# A Systematic Search and Mapping Review of Studies on Intracerebral Microdialysis of Amino Acids, and Systematized Review of Studies on Circadian Rhythms

**DOI:** 10.5334/jcr.172

**Published:** 2018-10-09

**Authors:** Cathalijn H. C. Leenaars, Jennifer Freymann, Koen Jakobs, Julia M. L. Menon, Thomas J. Van Ee, Janneke Elzinga, Rosalie W. M. Kempkes, Bea Zoer, Pim W. H. I. M. Drinkenburg

**Affiliations:** 1 RadboudUMC, Utrecht University, Utrecht and Nijmegen, NL; 2 Hannover Medical School, Hannover, DE; 3 University of Veterinary Medicine, DE; 4 Janssen Research and Development, A Division of Janssen Pharmaceutica NV, BE

**Keywords:** Amino acids, Histamine, Microdialysis, Systematic map, circadian rhythm

## Abstract

**Background:**

Microdialysis can be used to measure amino acids in the extracellular space in vivo, based on the principle of diffusion. Variations in experimental set-up result in variations in baseline levels of the compounds measured. Variations may also be due to circadian rhythms.

**Method:**

We systematically searched and mapped the literature on all studies reporting baseline microdialysis measurements of histamine and the amino acids asparagine, aspartate, GABA, glutamate, glutamine, glycine, proline and taurine. We fully reviewed the studies describing circadian rhythms for histamine and the selected amino acids.

**Results:**

We retrieved 2331 papers describing baseline measurements of one or more of the compounds of interest. We provide a numerical summary and lists of the publications by compound. We retrieved 11 references describing studies on the circadian rhythms of the compounds of interest. Aspartate, glutamate and histamine are generally higher during the dark than during the light phase in nocturnal rodents. For glutamine, no rhythmicity was observed. For GABA, the results were too inconsistent to generalise. For asparagine, glycine, proline and taurine, insufficient data are available.

**Conclusion:**

The literature on intracerebral microdialysis measurements of the amino acids is vast, but certain primary studies are still warranted. Future systematic reviews on the individual compounds can shed light on the effects of experimental variations on baseline concentrations.

## Introduction

Microdialysis is an elegant and convenient method to study the biochemistry of the extracellular space *in vivo.* It is based on simple diffusion; molecules diffuse from the tissue of interest into a continuously flowing isotonic fluid through a semi-permeable membrane. Microdialysis can be used to measure a variety of compounds. Concentrations of these molecules in the dialysate are not only affected by biological variations, but also by flow rate, membrane type and membrane surface area [1].

Amino acids are a set of compounds often analysed by microdialysis. Besides their function as building blocks of proteins, amino acids can function as excitatory (e.g. glutamate and aspartate) and inhibitory (e.g. GABA, glycine and taurine) neurotransmitters and neuromodulators. Amino acids are actually the most widely used neurotransmitters in the mammalian brain [2]. As neuroactive amino acids are also involved in intermediary neurotransmitter metabolism, it is difficult to analyse their transmitter role separate from their other biochemical roles [3]. Besides, brain concentration of amino acids can be altered by dietary intake [4].

Amino acids have been implied in the pathology of several neuropsychological disorders, including addiction to various compounds [5,6,7], ischaemia [8,9], depression [10], Tourette’s syndrome [11], Parkinson’s disease [12], and (neuropathic) pain [13]. Histamine and the amino acids asparagine, aspartate, GABA, glutamate, glutamine, glycine, proline and taurine can be measured in a single high-pressure liquid chromatography (HPLC) run. As we are using this HPLC method to analyse microdialysis samples, we are interested in the available literature on microdialysis studies measuring these specific compounds.

In clinical medicine, systematic reviews (SRs) are considered to provide the highest level of research evidence. In animal research, SRs are, so far, less common, while SRs are the optimal method to acquire a complete overview of the past use of a technique. While many interesting narrative reviews on microdialysis are available (refer to [1] for an interesting selection), only few SRs of microdialysis have been published to date. We thus set out to perform an SR to describe all available evidence on the observed range of baseline levels of histamine and the amino acids asparagine, aspartate, GABA, glutamate, glutamine, glycine, proline and taurine in microdialysates.

With increasing numbers of literature reviews of scientific findings, the types of reviews published are also increasing [14]. Most review types do not implement an explicit methodology, resulting in limited reliability of their findings. However, several review types transparently describe their methodology and strive for an approach that is still systematic: mapping reviews, scoping reviews, and rapid reviews. According to a recent SR of published evidence maps, evidence maps comprise a systematic search of a broad field that present results in a user-friendly format [15].

The differences between SRs and systematic maps (SMs) are outlined by [16]. In general, the objective of an SM is wider and more descriptive than answering a specific research question with an SR. For an SM, data extraction is limited, and critical appraisal is optional. The SM will identify knowledge clusters and knowledge gaps but will not synthesise data from the included studies. Besides these differences, systematic mapping mainly follows the same process as systematic reviewing. Systematic evidence mapping is increasingly encouraged in the environmental sciences [17], healthcare, and social sciences [18]. Systematic mapping of animal studies has so far hardly been explored.

The number of references retrieved by our systematic search proved to be too high to complete the planned full systematic review. We thus decided to use our search results for another systematic review approach, and created a systematic map of the available literature on studies measuring histamine and the amino acids asparagine, aspartate, GABA, glutamate, glutamine, glycine, proline and taurine in animal models using microdialysis. Besides, we fully reviewed the studies describing circadian rhythms for histamine and the selected amino acids, as circadian rhythms can affect the outcomes of any microdialysis experiment.

## Materials and Methods

On 12 January 2017, we published a protocol [19] for an SR addressing the research question: “What is the reported range of concentrations in intracerebral microdialysates for histamine and the amino acids asparagine, aspartate, GABA, glutamate, glutamine, glycine, proline and taurine?”. The protocol describes the used methodology for this SR up to the selection of papers.

### Search

We developed a comprehensive search strategy for studies on histamine and the amino acids asparagine, aspartate, GABA, glutamate, glutamine, glycine, proline and taurine for 2 databases: PubMed and Embase. We extended our previous search strategy for microdialysis [20] by adding the terms “chemitrode”, “dialytrode”, “brain dialysis”, “intracerebral dialysis”, “intracranial dialysis”, “transcranial dialysis” and “implanted perfused hollow fibre”. We used previously published filters to identify animal studies [21,22].

The full search strategies are described in our protocol [19]. Searches were performed on 12 and 14 December 2016. Duplicates were removed manually. We did not check reference lists of included papers or relevant reviews for additional papers, as this labour-intensive process would have retrieved only one additional paper using our improved search for microdialysis in our comparable review of adenosine [20].

### Selection

#### Selection for mapping review

Screening of titles and abstracts was performed in EROS (Early Review Organising Software; Institute of Clinical Effectiveness and Health Policy, Buenos Aires, Argentina) by 2 independent reviewers (2 out of CL, JF, KJ, TvE, JE, RK, JvL & BZ). Subsequent screening of full-text papers was also performed by 2 independent reviewers (2 out of CL, JF, KJ & TvE). Discrepancies were resolved by discussion among the reviewers. We excluded studies on other techniques than microdialysis (e.g. biosensors and studies using precursors to microdialysis such as ventricular, cortical cup and push-pull perfusion), studies measuring other substances than histamine and the amino acids asparagine, aspartate, GABA, glutamate, glutamine, glycine, proline and taurine, extra-cerebral microdialysis studies, human and in vitro studies, and (in the full-text screening phase only) papers not containing primary study data. In the full-text screening we further excluded retro-dialysis studies and other microdialysis studies that did not report baseline values without the specified compounds in the perfusion fluid.

#### Selection for systematised review of circadian rhythms

During full-text screening, tags were added by KJ and CL to all studies on circadian rhythms, sleep and sleep deprivation. Tagged studies were separately screened afterwards for inclusion based on the following criterium (besides being included in our mapping): studies measuring one or more of the compounds of interest during an undisturbed baseline period of minimally 6 hours including a light-dark transition. Most of the excluded tagged studies measured for shorter periods after pharmacological interventions, or after retrodialysis of various compounds.

To ensure capturing all relevant studies, we searched for studies with the terms “*circadi*” “*period*”, “*clock*”, “*oscill*”, “*basel*”, “*pacemaker*”, “*ultradian*”, “*diurnal*”and “*rhythm*” in the list of titles of the studies included in our mapping.

### Mapping

During full-text screening, labels were added to all included references by one of the screeners (CL or KJ) to reflect for which of the compound(s) of interest (histamine and the amino acids asparagine, aspartate, GABA, glutamate, glutamine, glycine, proline and taurine) baseline concentrations were provided. The EROS-file was exported to Excel, where references were sorted by these labels to create the tables provided in the appendices. An individual paper is included in multiple tables if it describes baseline dialysate concentrations of more than one compound of interest.

A subset of 140 studies was labelled by both screeners and analysed for concordance.

### Systematised review

The following data were extracted for papers included in the review on circadian rhythms: bibliographic details, location of laboratory, n, species, strain, age and weight, sex, flowrate, probe length, probe diameter, membrane type, probe reuse, brain region, wash-out, anaesthesia/freely behaving, post-surgical recovery, amino acids analysed, type of analysis, light-dark cycle and summary of findings. When a reference described measurements of several amino acids and/or histamine, findings were separately tabulated for all compounds. Likewise, when references described measurements in several brain regions (e.g. the nucleus accumbens and the striatum; [23]) or for various experimental conditions (e.g. freely behaving and anaesthetised; [24]), findings were separately tabulated.

### Risk of bias assessment

In our protocol, we explained that the available risk-of-bias tools are hardly applicable to baseline measurements. We thus planned to provide an indication of study quality, internal validity and risk of bias by tabulating the extracted study characteristics.

For the mapping review, only limited data were extracted, and all references are provided. We did not perform a formal risk of bias assessment for these 2331 included studies. For the studies included in our systematised review, the risk of bias was estimated by one experimenter. We added the following elements to the analysis: blinding of sample analysis for detection bias and incomplete outcome data for attrition bias, [25], and for other sources of bias: verification of probe placement, reporting of an a priori power analysis, approval of an ethical board, and conflicts of interest.

## Results

### Search and selection

**Figure 1:**
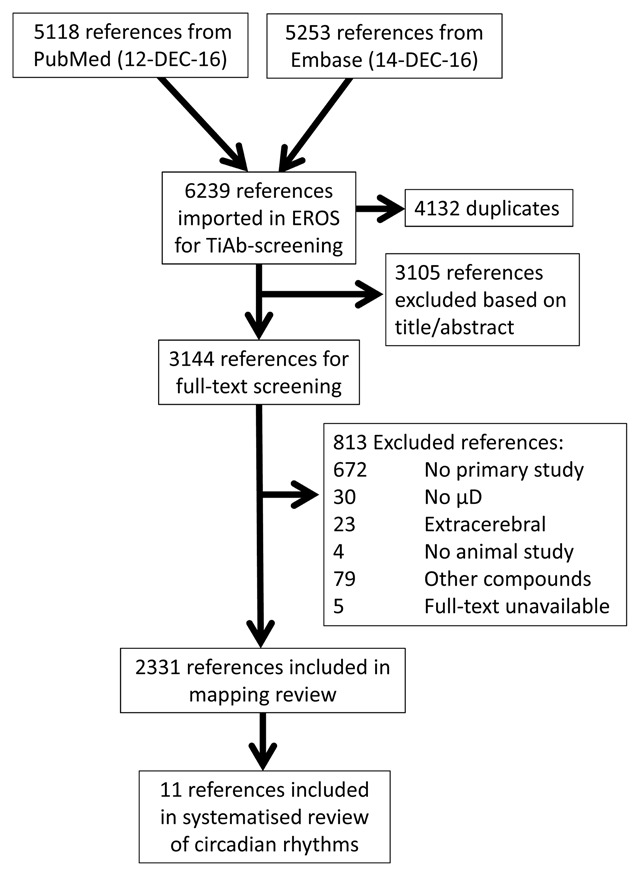
Flow scheme of retrieved and included references. TiAb – Title-Abstract; μD = microdialysis.

Our search in PubMed retrieved 5118 references, that in Embase 5253. After removal of duplicates, 6239 references remained for title-abstract screening. Of these, 3144 were screened full-text, and 2331 were included in the mapping review. An overview of the flow of papers is provided in Figure 1.

We tagged 47 references during full-text screening as relevant for circadian rhythms, sleep and sleep deprivation. Of these, 7 met the inclusion criterium for our systematised review on circadian rhythms. Our search for relevant title words added 4 references, resulting in 11 references included in our SR on circadian rhythms.

### Mapping review; retrieved references by compound

The number of references retrieved by compound is provided in Table 1. The number of references by compound varies from only 6 references for proline to 1876 references for glutamate. Concordance of labelling in the subset of 140 studies ranged from 93.6% for GABA to 100% for proline. Concordance percentages are also provided in Table 1.

**Table 1:** Number of references by compound and concordance between screeners.

Compound	Concordance in subset (%)	Number of references retrieved

Asparagine	98.6	60
Aspartate	95.7	600
GABA	93.6	918
Glutamine	95.7	253
Glutamate	93.6	1876
Glycine	99.3	338
Histamine	99.3	102
Proline	100	6
Taurine	94.3	411

Only one study described the baseline microdialysate levels for all our compounds of interest [26], and 4 more described 8 out of 9 [4,27,28,29]. Most of the studies reported only one (1246 studies) or two (548 studies) of these compounds. References for the included studies are provided by compound in appendix 1–9. The appendix tables can be used by scientists interested in intracerebral microdialysis of histamine and/or one or more of the analysed amino acids. The titles in a specific table can be screened one by one, or a search for title words of interest can be performed. When searching for title words, we highly recommend searching for all known synonyms of a term of interest, and searching for the first letters of the term to retrieve both the singular and the plural use and different spellings.

### Systematised review on circadian rhythms in microdialysate amino acids

The 11 included references described 25 studies in 1–15 animals (9 references on rats; 5× Wistar, 4× Sprague-Dawley, 1 on mice, 1 on hamsters) in different brain regions (frontal cortex, suprachiasmatic nucleus, striatum, nucleus accumbens, basal forebrain and hypothalamus), all in English. One reference did not mention the sex of the rats, the others all used male animals only. The light schedule was adequately described by 10 out of 11 references, one only mentioned a 12 h light-dark cycle without providing actual times, but expressed the data in Zeitgeber time [30]. For the 10 papers describing the light-dark cycle, the cycle was normal (12:12, lights on at 6:00–8:00) for 7, reversed for 2 (12:12, lights on at 20:00 or 20:30), or partially reversed (12:12, lights on at 24:00 h).

Microdialysis flow rate was reported by 10 out of 11 references, and ranged from 1 to 2 μl/min. Probe lengths (0.5–4 mm) were reported by 10 out of 11 references, probe diameters (0.2–0.5 mm) by 5 out of 11. Membrane type (e.g. Hospal or CMA7) and/or molecular weight cut off (1–50 kDa) was reported by 10 out of 12 references. None of the references mentioned probe reuse. All references described HPLC for sample analysis, 9 with fluorescence detection, 1 with electrochemical detection, one with both detection types. Either histamine only (6 references), or several amino acids (5 references) were measured.

Study characteristics are provided by study and amino acid in appendix 10.

### Aspartate

Table 2 lists the 2 studies on aspartate circadian rhythms. In rats, Honma et al. tested rhythmicity in freely behaving animals and under urethane anaesthesia on a 12:12 light-dark cycle [24]. Baseline dialysis commenced at different points of the light-dark cycle, and lasted 24 h. Group averages were calculated after smoothing of the individual animal data with a three-point running average. Circadian effects were tested with a two-way analysis of variance (ANOVA) which showed a significant effect. According to the authors, aspartate levels started to decrease before light onset, and increase before dark onset. In hamsters kept on a 14:10 light-dark cycle (lights on at 08:00) and measured for 24 h, Glass et al. standardised values to a percentage of the daily mean [31]. Time-of-day effects were tested by a multivariate time series analysis (MANOVA) followed by Student Newman Keuls tests. Aspartate peaked during the dark phase; 01:00–03:00 and 07:00–08:00 h.

**Table 2:** Microdialysis studies on aspartate circadian rhythms.

Study_ID	Species	Strain	Brain Area	Summary

Honma_1996b	Rats	Wistar	SCN	Asp is higher during the dark than during the light phase
Honma_1996d	Rats	Wistar	SCN	
Glass_1993b	Hamsters	Home bred	SCN	

SCN: SupraChiasmatic Nucleus; Asp: Aspartate.

To conclude, in the suprachiasmatic nucleus, aspartate is higher during the dark than during the light phase in both rats and hamsters, and the rhythm is also present under urethane anaesthesia [24,31].

### GABA

Table 3 lists the 3 studies on GABA circadian rhythms. Castaneda et al. sampled for 30 h starting at 14 h from rats on a 12:12 light-dark cycle; lights ON at 20:00 [23]. Values were expressed as a percentage of the average of the first 8 samples and somehow corrected for slow continuous changes in dialysate concentrations. Circadian rhythms were tested with the Cosinor method to fit the data to a sinusoidal model. If the fit was not significant, but differences between dark and light were apparent, samples 1 and 24 and samples 25 and 48 were compared with linear regression. GABA did not show circadian variation. Marquez de Prado et al. collected dialysates for 24 h after establishment of a stable baseline level [32]. Rats were on a 12:12 light-dark cycle; lights ON at 08:00. Repeated measure ANOVAs following linear regression analyses showed significant circadian variations; GABA decreased by 19% during the light period and rose to 100% of basal values at night. Meng et al. dialyzed rats on 12:12 light-dark cycle; lights ON at 07:00 [33]. Samples were collected at 2 h intervals over a 24 h period. Data were analyzed with paired-samples t-test and independent-samples t-test. The authors observed a circadian pattern with relatively high levels during the dark period and low levels during the light period.

**Table 3:** Microdialysis studies on GABA circadian rhythms.

Study_ID	Species	Strain	Brain Area	Summary

Castaneda_2004b	Rats	Wistar	Striatum	GABA shows no significant circadian variation, although it may be higher during dark than during light
Castaneda_2004e	Rats	Wistar	NAcc	
MarquezdePrado_2000b	Rats	Wistar	Neostriatum	GABA is higher during the dark than during the light phase
Meng_2015	Rats	Spraque-Dawley	Striatum	

NAcc: Nucleus Accumbens; GABA: gamma-aminobutyric acid.

To conclude, results for GABA are too inconsistent to generalise [23,32,33].

### Glutamate

Table 4 lists the 5 studies on glutamate circadian rhythms. These studies were all described above. According to Castaneda et al. glutamate followed a sinusoidal model [23]. Honma et al. state that glutamate levels started to decrease before light onset, and increase before dark onset [24]. Marquez de Prado et al. showed significant circadian variations; glutamate decreased by 37% during the light period and increased during the following dark period [32]. Glass et al. described an acrophase during the dark period [31]. Meng et al. observed a circadian pattern with relatively high levels during the dark and low levels during the light [33].

**Table 4:** Microdialysis studies on glutamate circadian rhythms.

Study_ID	Species	Strain	Brain Area	Summary

Castaneda_2004a	Rats	Wistar	Striatum	Glu is higher during the dark than during the light phase
Castaneda_2004d	Rats	Wistar	NAcc	
Honma_1996a	Rats	Wistar	SCN	
Honma_1996c	Rats	Wistar	SCN	
MarquezdePrado_2000a	Rats	Wistar	Neostriatum	
Glass_1993a	Hamsters	Home bred	SCN	
Meng_2015	Rats	Spraque-Dawley	Striatum	

NAcc: Nucleus Accumbens; SCN: SupraChiasmatic Nucleus; Glu: glutamate.

To conclude, glutamate seems consistently higher during the dark than during the light phase in the suprachiasmatic nucleus in hamsters and in several brain regions in freely behaving and anaesthetised rats [23,24,31,32,33].

### Glutamine

Table 5 lists the 3 studies on glutamine circadian rhythms. These studies were all described above. Castaneda et al. found no circadian variation in glutamine concentrations [23]. The study by Marquez de Prado et al. also showed no circadian rhythm for glutamine [32]. Glass et al. did not describe the glutamine findings in the text, but their figure shows that there is no significant rhythmicity for glutamine [31].

**Table 5:** Microdialysis studies on glutamine circadian rhythms.

Study_ID	Species	Strain	Brain Area	Summary

Castaneda_2004c	Rats	Wistar	Striatum	Gln shows no circadian rhythm
Castaneda_2004f	Rats	Wistar	NAcc	
MarquezdePrado_2000c	Rats	Wistar	Neostriatum	
Glass_1993c	Hamsters	homebred	SCN	

NAcc: Nucleus Accumbens; SCN: SupraChiasmatic Nucleus; Gln: glutamine.

To conclude, no rhythmicity is observed in glutamine in different brain regions and different species [23,31,32].

### Histamine

Table 6 lists the 6 studies on histamine circadian rhythms. Fell et al. describe several experiments, of which one met the inclusion criteria of our systematised review [34]. Rats were kept on a 12:12 light-dark cycle; lights ON at 24:00. The circadian rhythm was not specifically analysed, but from the figures it is clear that baseline histamine increases during the dark compared to the light period. Chu et al. kept rats on a 12:12 light-dark cycle; lights ON at 08:00, and collected dialysate over 24 h [35]. The circadian rhythm was not specifically analysed, but the figure shows that baseline histamine increases during the dark compared to the light period. Hong et al. also kept rats on a 12:12 light-dark cycle; lights ON at 08:00 [36]. Dialysates were collected for 14-h starting at 20:00. The circadian rhythm was again not specifically analysed, but the figure shows that histamine is higher at the onset of the dark phase compared to the light phase. Zant et al. kept rats on an unspecified 12:12 light-dark cycle and collected dialysate over a 24 h control day [30]. The authors observed a strong correlation (r = 0.84, p < 0.001) between histamine levels and time awake, which is higher during the dark. Individual animal data also show higher histamine during dark than during light. Mochizuki et al. kept rats on a 12:12 light-dark cycle; lights ON at 08:00 [37]. They collected dialysates over variable periods of time over the circadian cycle. Averages for the light and the dark period were compared with a paired, two-tailed Student’s t-test. Average histamine concentrations were higher during dark than during light. Rozov et al. kept mice on a 12:12 light-dark cycle; lights ON at 20:30, and collected dialysate for five consecutive days [38]. Data were analysed with the Lomb–Scargle method using LSP software. The authors found 24-h and overlaid 8-h periodicities in histamine release, with an orthophase at 17:63.

**Table 6:** Microdialysis studies on histamine circadian rhythms.

Study_ID	Species	Strain	Brain Area	Summary

Fell_2015	Rat	Spraque-Dawley	mPFC	Hist is higher during the dark than during the light phase
Chu_2004	Rats	Spraque-Dawley	Frontal cortex	
Hong_2005	Rats	Spraque-Dawley	Frontal cortex	
Zant_2012	Rat	Han-Wistar	BF	
Mochizuki_1992	Rats	Wistar	Hypothalamus	
Rozov_2014	Mice	C57BL6/J	Hypothalamus	

mPFC: medial Prefrontal Cortex; BF: Basal Forebrain; Hist: histamine.

To conclude, histamine seems consistently higher during the dark than during the light phase in the hypothalamus in mice and in several brain regions in rats [34,35,36,37,38,39].

### Taurine

Only one included reference measured taurine; in the neostriatum of Wistar rats. This study by Marquez de Prado et al. was described above [32]. Taurine showed no circadian rhythm.

### Asparagine, Glycine and Proline

Our search retrieved no studies of circadian rhythms in asparagine, glycine and proline.

### Risk of Bias

Reporting of study characteristics was analysed by reference. None of the included references described blinding of sample analysis, missing samples or power analyses. Only 1 out of 11 references contained a statement on conflicts of interest. Approval of the protocol by an ethical board was reported in 6 out of 11 references. Methods for verification of probe placement were reported in 8 out of 11 references, but only 3 showed the results.

Concluding, the risk of bias is unclear for all included studies.

## Discussion

Our systematic map provides an overview of the papers describing baseline microdialysis measurements of histamine and the amino acids asparagine, aspartate, GABA, glutamate, glutamine, glycine, proline and taurine. In our results section, we describe how scientists can use this systematic map, to save the time involved in performing a systematic search and selection for one or more of the analysed compounds themselves. In our systematized review, we provide an example.

The map shows that many more microdialysis studies have measured glutamate and GABA than proline and histamine. This partially echoes researchers interests in compounds as a neurotransmitter but is probably also reflecting the relative ease of measuring glutamate in dialysates. Included studies describe analytical methods [40,41], functional neuroanatomy [42,43], basic pharmacology [44,45], the neurochemistry of behaviour [46,47,48], and the neurochemistry of several disorders (described in the introduction).

The 11 references on circadian rhythms show a relative abundance of studies on histamine, but none of them addressed asparagine, glycine or proline, and only one was on taurine. Aspartate, glutamate and histamine are generally higher during the dark than during the light phase in nocturnal rodents. For glutamine, no rhythmicity was observed. For GABA, the results were too inconsistent to generalise. For asparagine, GABA, glycine, proline and taurine, further primary studies on circadian rhythms are warranted.

Several authors have published SRs on the microdialysis technique, but we are aware of only one SR on amino acids [49]. Fliegel et al. performed an SR followed by several meta-analyses of glutamate and GABA in relation to ethanol exposure. We do not know if they followed a prespecified review protocol. Their search was more focussed than ours, they used key search terms and searched one database, which resulted in a selection of the available literature; 245 studies reporting baseline glutamate and or GABA levels. While methodology of this SR was not described to the extent that we can evaluate its quality, it provides extremely useful overviews of the baseline concentrations of glutamate and GABA for several brain regions.

Our comprehensive search strategy makes this the most complete review of amino acids in microdialysates up to date. We based our search for microdialysis studies on previous work [20] and extended it by including the terms “chemitrode”, “dialytrode”, “brain dialysis”, “intracerebral dialysis”, “intracranial dialysis”, “transcranial dialysis” and “implanted perfused hollow fibre” to improve retrieval of older studies.

Because the size of the review was larger than anticipated, we had to adapt our protocol. By our labelling approach we managed to create a map of the literature by amino acid of interest. We only labelled studies for measurements of asparagine, aspartate, GABA, glutamate, glutamine, glycine, histamine, proline and taurine. In the included papers, we regularly came across microdialysis measurements of other amino acids, e.g. alanine, serine, and threonine. Future systematic reviews should summarise the findings for these compounds.

Labelling was performed by one of the screeners. We analysed concordance in a subset of 140 papers. Concordance ranged from 93.6% to 100%. We did not reanalyse the discordant papers to determine if they are erroneously included or if they were missed by one of the screeners. There thus is a risk of both missing relevant studies and errors of overinclusion. However, the margin of error is quite likely smaller than when using non-systematic search approaches.

Tagging and/or labelling of references on specific topics during screening for large reviews is an interesting approach to increase the output and relevance of review efforts. Our labelling for amino acids seems rather reliable, with concordance >93%. Our tagging for studies on circadian rhythms was less successful, we tagged only 7 of the 11 references included in our systematised review. Possibly, this was due to a loss of focus. Tagging did have added value; we included 4 references via tags that would not have been found with our title search. Of note, our searches also retrieved publications on other than circadian biological rhythms, e.g. circannual rhythms, [50] reflecting the limited specificity of searching for text words in general. When using the tagging/labelling strategy for future reviews, we advise against labelling for 2 topics simultaneously, and to restrict labelling to straightforward concepts (e.g. well-defined chemical compounds).

The provided lists of references in our mapping review can be used as the basis for systematic reviews for future projects. We envision for example rapid review strategies for certain fields based on searches for title words within the larger tables (e.g. for glutamate), or on screening of all the titles in one of the smaller tables (e.g. histamine).

We provide such a systematized review of circadian rhythms as a proof of concept. This review shows circadian rhythms for aspartate, glutamate and histamine, and no rhythmicity for glutamine. Next to this review, a second review of the studies about natural sleep and sleep deprivation is in progress. Our systematic map is relevant to all neurochemists; it is not specific to the field of chronobiology. We chose to present it with our systematised review of the circadian data as systematic reviews are still relatively scarce in animal chronobiology. We thus hope to inspire chronobiologists to use more systematised review strategies. We highly encourage anyone performing a meta-analysis on microdialysis data to address the effects of variations in experimental design, as done, for example, in [20,51,52].

## Data Accessibility Statement

Data in this publication are already in the public domain. Extracted data will be made available for reuse to individual scientists upon reasonable requests.

## Additional Files

The additional files for this article can be found as follows:

10.5334/jcr.172.s1 Appendix 1 Studies describing baseline dialysate concentrations for asparagine.

10.5334/jcr.172.s1 Appendix 2 Studies describing baseline dialysate concentrations for aspartate.

10.5334/jcr.172.s1 Appendix 3 Studies describing baseline dialysate concentrations for GABA.

10.5334/jcr.172.s1 Appendix 4 Studies describing baseline dialysate concentrations for glutamine.

10.5334/jcr.172.s1 Appendix 5 Studies describing baseline dialysate concentrations for glutamate.

10.5334/jcr.172.s1 Appendix 6 Studies describing baseline dialysate concentrations for glycine.

10.5334/jcr.172.s1 Appendix 7 Studies describing baseline dialysate concentrations for histamine.

10.5334/jcr.172.s1 Appendix 8 Studies describing baseline dialysate concentrations for proline.

10.5334/jcr.172.s1 Appendix 9 Studies describing baseline dialysate concentrations for taurine.

10.5334/jcr.172.s1 Appendix 10 Study characteristics for microdialysis studies on circadian rhythms of aspartate, GABA, glutamine, glutamate and histamine.
