# Supplementary material for: A Systematic Search and Mapping Review of Studies on Intracerebral Microdialysis of Amino Acids, and Systematized Review of Studies on Circadian Rhythms
Source: J Circadian Rhythms. 2018 Oct 9;16:12. doi: 10.5334/jcr.172 (PMC6196574; doi:10.5334/jcr.172)
Supplement: Appendix 1 — Studies describing baseline dialysate concentrations for asparagine. [file jcr-16-172-s1.pdf]

Appendix 1: Studies describing baseline dialysate concentrations for asparagine

**Asparagine**					
**Authors**	**Title**	**Year**	**Vol**	**Pages**	**Journal**
Abraham, I.; Juhasz, G.; Kekesi, K. A.; Kovacs, K. J.	Corticosterone peak is responsible for stress-induced elevation of glutamate in the hippocampus	1998	2	171-181	Stress
Abraham, I.; Juhasz, G.; Kekesi, K. A.; Kovacs, K. J.	Effect of intrahippocampal dexamethasone on the levels of amino acid transmitters and neuronal excitability	1996	733	56-63	Brain Res
Acworth, I. N.; Yu, J.; Ryan, E.; Gariepy, K. C.; Gamache, P.; Hull, K.; Maher, T.	Simultaneous measurement of monoamine, amino acid, and drug levels, using high performance liquid chromatography and coulometric array technology: Application to in vivo microdialysis perfusate analysis	1994	17	685-705	J Liq Chromatogr
Andine, P.; Orwar, O.; Jacobson, I.; Sandberg, M.; Hagberg, H.	Changes in extracellular amino acids and spontaneous neuronal activity during ischemia and extended reflow in the CA1 of the rat hippocampus	1991	57	222-229	J Neurochem
Bereiter, D. A.; Shen, S.; Benetti, A. P.	Sex differences in amino acid release from rostral trigeminal subnucleus caudalis after acute injury to the TMJ region	2002	98	89-99	Pain
Boyd, B. W.; Witowski, S. R.; Kennedy, R. T.	Trace-level amino acid analysis by capillary liquid chromatography and application to in vivo microdialysis sampling with 10-s temporal resolution	2000	72	865-871	Anal Chem
Buisson, A.; Callebert, J.; Mathieu, E.; Plotkine, M.; Boulu, R. G.	Striatal protection induced by lesioning the substantia nigra of rats subjected to focal ischemia	1992	59	1153-1157	J Neurochem
Butcher, S. P.; Hamberger, A.	In vivo studies on the extracellular, and veratrine-releasable, pools of endogenous amino acids in the rat striatum: effects of corticostriatal deafferentation and kainic acid lesion	1987	48	713-721	J Neurochem
Butcher, S. P.; Jacobson, I.; Hamberger, A.	On the epileptogenic effects of kainic acid and dihydrokainic acid in the dentate gyrus of the rat	1988	27	375-381	Neuropharmacol
Butcher, S. P.; Lazarewicz, J. W.; Hamberger, A.	In vivo microdialysis studies on the effects of decortication and excitotoxic lesions on kainic acid-induced calcium fluxes, and endogenous amino acid release, in the rat striatum	1987	49	1355-1360	J Neurochem
Butcher, S. P.; Sandberg, M.; Hagberg, H.; Hamberger, A.	Cellular origins of endogenous amino acids released into the extracellular fluid of the rat striatum during severe insulin-induced hypoglycemia	1987	48	722-728	J Neurochem
Choi, Y. H.; Chang, N.; Fletcher, P. J.; Anderson, G. H.	Dietary protein content affects the profiles of extracellular amino acids in the medial preoptic area of freely moving rats	2000	66	1105-1118	Life Sci

Asparagine					
**Authors**	**Title**	**Year**	**Vol**	**Pages**	**Journal**
Choi, Y. H.; Fletcher, P. J.; Anderson, G. H.	Extracellular amino acid profiles in the paraventricular nucleus of the rat hypothalamus are influenced by diet composition	2001	892	320-328	Brain Res
Currie, P. J.; Chang, N.; Luo, S.; Anderson, G. H.	Microdialysis as a tool to measure dietary and regional effects on the complete profile of extracellular amino acids in the hypothalamus of rats	1995	57	1911-1923	Life Sci
David, C. N.; Frias, E. S.; Szu, J. I.; Vieira, P. A.; Hubbard, J. A.; Lovelace, J.; Michael, M.; Worth, D.; McGovern, K. E.; Ethell, I. M.; Stanley, B. G.; Korzus, E.; Fiacco, T. A.; Binder, D. K.; Wilson, E. H.	GLT-1-Dependent Disruption of CNS Glutamate Homeostasis and Neuronal Function by the Protozoan Parasite Toxoplasma gondii	2016	12	e1005643	PLoS Pathog
Dolgodilina, E.; Imobersteg, S.; Laczko, E.; Welt, T.; Verrey, F.; Makrides, V.	Brain interstitial fluid glutamine homeostasis is controlled by blood-brain barrier SLC7A5/LAT1 amino acid transporter	2016	36	1929-1941	J Cereb Blood Flow Metab
Fedele, E.; Foster, A. C.	An evaluation of the role of extracellular amino acids in the delayed neurodegeneration induced by quinolinic acid in the rat striatum	1993	52	911-917	Neuroscience
Fraser, M.; Bennet, L.; Van Zijl, P. L.; Mocatta, T. J.; Williams, C. E.; Gluckman, P. D.; Winterbourn, C. C.; Gunn, A. J.	Extracellular amino acids and lipid peroxidation products in periventricular white matter during and after cerebral ischemia in preterm fetal sheep	2008	105	2214-2223	J Neurochem
Gobert, A.; Rivet, J. M.; Billiras, R.; Parsons, F.; Millan, M. J.	Simultaneous quantification of D- vs. L-serine, taurine, kynurenate, phosphoethanolamine and diverse amino acids in frontocortical dialysates of freely-moving rats: differential modulation by N-methyl-D-aspartate (NMDA) and other pharmacological agents	2011	202	143-157	J Neurosci Methods
Goldsmith, J. D.; Kujawa, S. G.; McLaren, J. D.; Bledsoe Jr, S. C.	In vivo release of neuroactive amino acids from the inferior colliculus of the guinea pig using brain microdialysis	1995	83	80-88	Hear Res
Gordon, K. E.; Simpson, J.; Statman, D.; Silverstein, F. S.	Effects of perinatal stroke on striatal amino acid efflux in rats studied with in vivo microdialysis	1991	22	928-932	Stroke
Guerra-Romero, L.; Tureen, J. H.; Fournier, M. A.; Makrides, V.; Tauber, M. G.	Amino acids in cerebrospinal and brain interstitial fluid in experimental pneumococcal meningitis	1993	33	510-513	Pediatr Res
Haskew-Layton, R. E.; Rudkouskaya, A.; Jin, Y.; Feustel, P. J.; Kimelberg, H. K.; Mongin, A. A.	Two distinct modes of hypoosmotic medium-induced release of excitatory amino acids and taurine in the rat brain in vivo	2008	3	e3543	PLoS One
Jacobson, I.; Sandberg, M.; Hamberger, A.	Mass transfer in brain dialysis devices--a new method for the estimation of extracellular amino acids concentration	1985	15	263-268	J Neurosci Methods
Largo, C.; Cuevas, P.; Somjen, G. G.; Martin	The effect of depressing glial function in rat brain in situ on ion	1996	16	1219-	J Neurosci

Asparagine					
**Authors**	**Title**	**Year**	**Vol**	**Pages**	**Journal**
del Rio, R.; Herreras, O.	homeostasis, synaptic transmission, and neuron survival			1229	
Lehmann, A.	Alterations in hippocampal extracellular amino acids and purine catabolites during limbic seizures induced by folate injections into the rabbit amygdala	1987	22	573-578	Neuroscience
Lerma, J.; Herranz, A. S.; Herreras, O.	In vivo determination of extracellular concentration of amino acids in the rat hippocampus. A method based on brain dialysis and computerized analysis	1986	384	145-155	Brain Research
Li, H.; Yan, Z. Y.	Analysis of amino acid neurotransmitters in hypothalamus of rats during cerebral ischemia-reperfusion by microdialysis and capillary electrophoresis	2010	24	1185-1192	Biomed Chromatogr
Li, Z. P.; Zhang, X. Y.; Lu, X.; Zhong, M. K.; Ji, Y. H.	Dynamic release of amino acid transmitters induced by valproate in PTZ-kindled epileptic rat hippocampus	2004	44	263-270	Neurochem Int
Lo, E. H.; Steinberg, G. K.; Panahian, N.; Maidment, N. T.; Newcomb, R.	Profiles of extracellular amino acid changes in focal cerebral ischaemia: effects of mild hypothermia	1993	15	281-287	Neurol Res
Malmberg, A. B.; Hamberger, A.; Hedner, T.	Effects of prostaglandin E2 and capsaicin on behavior and cerebrospinal fluid amino acid concentrations of unanesthetized rats: a microdialysis study	1995	65	2185-2193	J Neurochem
Mena, F. V.; Baab, P. J.; Zielke, C. L.; Zielke, H. R.	In vivo glutamine hydrolysis in the formation of extracellular glutamate in the injured rat brain	2000	60	632-641	J Neurosci Res
Miyashita, K.; Abe, H.; Nakajima, T.; Ishikawa, A.; Nishiura-Suzuki, M.; Naritomi, H.; Tanaka, R.; Sawada, T.	Glutamate release in the gerbil hippocampus after middle cerebral artery occlusion	1994	5	945-948	Neuroreport
Molchanova, S.; Kbi, P.; Oja, S. S.; Saransaari, P.	Interstitial concentrations of amino acids in the rat striatum during global forebrain ischemia and potassium-evoked spreading depression	2004	29	1519-1527	Neurochem Res
Morita, T.; Takahashi, M.; Takeuchi, T.; Hikasa, Y.; Ikeda, S.; Sawada, M.; Sato, K.; Shibahara, T.; Shimada, A.	Changes in extracellular neurotransmitters in the cerebrum of familial idiopathic epileptic shetland sheepdogs using an intracerebral microdialysis technique and immunohistochemical study for glutamate metabolism	2005	67	1119-1126	J Vet Med Sci
Newcomb, R.; Palma, A.	Effects of diverse omega-conopeptides on the in vivo release of glutamic and gamma-aminobutyric acids	1994	638	95-102	Brain Res
Nilsson, P.; Hillered, L.; Ponten, U.; Ungerstedt, U.	Changes in cortical extracellular levels of energy-related metabolites and amino acids following concussive brain injury in rats	1990	10	631-637	J Cereb Blood Flow Metab

Asparagine					
**Authors**	**Title**	**Year**	**Vol**	**Pages**	**Journal**
Nilsson, P.; Ronne-Engstrom, E.; Flink, R.; Ungerstedt, U.; Carlson, H.; Hillered, L.	Epileptic seizure activity in the acute phase following cortical impact trauma in rat	1994	637	227-232	Brain Res
Qu, Y.; Li, Y.; Vandenbussche, E.; Vandesande, F.; Arckens, L.	In vivo microdialysis in the visual cortex of awake cat. II: sample analysis by microbore HPLC-electrochemical detection and capillary electrophoresis-laser-induced fluorescence detection	2001	7	45-51	Brain Res Brain Res Protoc
Qureshi, A. I.; Ali, Z.; Suri, M. F.; Shuaib, A.; Baker, G.; Todd, K.; Guterman, L. R.; Hopkins, L. N.	Extracellular glutamate and other amino acids in experimental intracerebral hemorrhage: an in vivo microdialysis study	2003	31	182-189	Crit Care Med
Radhakrishnan, R.; Sluka, K. A.	Increased glutamate and decreased glycine release in the rostral ventromedial medulla during induction of a pre-clinical model of chronic widespread muscle pain	2009	457	141-145	Neurosci Lett
Renno, W. M.; Mullet, M. A.; Williams, F. G.; Beitz, A. J.	Construction of 1 mm microdialysis probe for amino acids dialysis in rats	1998	79	217-228	J Neurosci Methods
Sandberg, M.; Butcher, S. P.; Hagberg, H.	Extracellular overflow of neuroactive amino acids during severe insulin-induced hypoglycemia: in vivo dialysis of the rat hippocampus	1986	47	178-184	J Neurochem
Sheng, W.; Hang, H. W.; Ruan, D. Y.	In vivo microdialysis study of the relationship between lead-induced impairment of learning and neurotransmitter changes in the hippocampus	2005	20	233-240	Environ Toxicol Pharmacol
Silverstein, F. S.; Naik, B.; Simpson, J.	Hypoxia-ischemia stimulates hippocampal glutamate efflux in perinatal rat brain: an in vivo microdialysis study	1991	30	587-590	Pediatr Res
Sved, A. F.; Curtis, J. T.	Amino acid neurotransmitters in nucleus tractus solitarius: an in vivo microdialysis study	1993	61	2089-2098	J Neurochem
Szeg, E. M.; Kekesi, K. A.; Szabo, Z.; Janaky, T.; Juhasz, G. D.	Estrogen regulates cytoskeletal flexibility, cellular metabolism and synaptic proteins: A proteomic study	2010	35	807-819	Psychoneuroendocrinology
Taylor, D. L.; Davies, S. E.; Obrenovitch, T. P.; Doheny, M. H.; Patsalos, P. N.; Clark, J. B.; Symon, L.	Investigation into the role of N-acetylaspartate in cerebral osmoregulation	1995	65	275-281	J Neurochem
Tseng, E. E.; Brock, M. V.; Lange, M. S.; Troncoso, J. C.; Blue, M. E.; Lowenstein, C. J.; Johnston, M. V.; Baumgartner, W. A.	Monosialoganglioside GM1 inhibits neurotoxicity after hypothermic circulatory arrest	1998	124	289-306	Surgery
Uutela, P.; Ketola, R. A.; Piepponen, P.; Kostiainen, R.	Comparison of different amino acid derivatives and analysis of rat brain microdialysates by liquid chromatography tandem mass	2009	633	223-231	Anal Chim Acta

Asparagine					
Authors	Title	Year	Vol	Pages	Journal
	spectrometry				
Vallee, N.; Rostain, J. C.; Boussuges, A.; Risso, J. J.	Comparison of nitrogen narcosis and helium pressure effects on striatal amino acids: a microdialysis study in rats	2009	34	835-844	Neurochem Res
Vallee, N.; Rostain, J. C.; Risso, J. J.	A pressurized nitrogen counterbalance to cortical glutamatergic pathway stimulation	2010	35	718-726	Neurochem Res
Vallee, N.; Rostain, J. C.; Risso, J. J.	How can an inert gas counterbalance a NMDA-induced glutamate release?	2009	107	1951-1958	J Appl Physiol (1985)
Wade, J. V.; Olson, J. P.; Samson, F. E.; Nelson, S. R.; Pazdernik, T. L.	A possible role for taurine in osmoregulation within the brain	1988	51	740-745	J Neurochem
West, A. R.; Galloway, M. P.	Inhibition of glutamate reuptake potentiates endogenous nitric oxide-facilitated dopamine efflux in the rat striatum: an in vivo microdialysis study	1997	230	21-24	Neurosci Lett
Westergren, I.; Nystrom, B.; Hamberger, A.; Johansson, B. B.	Intracerebral dialysis and the blood-brain barrier	1995	64	229-234	J Neurochem
Westerink, B. H.; Damsma, G.; de Vries, J. B.	Effect of ouabain applied by intrastriatal microdialysis on the in vivo release of dopamine, acetylcholine, and amino acids in the brain of conscious rats	1989	52	705-712	J Neurochem
Westerink, B. H.; Hofsteede, H. M.; Damsma, G.; de Vries, J. B.	The significance of extracellular calcium for the release of dopamine, acetylcholine and amino acids in conscious rats, evaluated by brain microdialysis	1988	337	373-378	Naunyn Schmiedebergs Arch Pharmacol
Wong, J. M.; Malec, P. A.; Mabrouk, O. S.; Ro, J.; Dus, M.; Kennedy, R. T.	Benzoyl chloride derivatization with liquid chromatography-mass spectrometry for targeted metabolomics of neurochemicals in biological samples	2016	78-90	78-90	J Chromatogr A
Xu, G. Y.; McAdoo, D. J.; Hughes, M. G.; Robak, G.; De Castro Jr, R.	Considerations in the determination by microdialysis of resting extracellular amino acid concentrations and release upon spinal cord injury	1998	86(3)	1011-1021	Neuroscience

Appendix 2: Studies describing baseline dialysate concentrations for aspartate

Aspartate					
**Authors**	**Title**	**Year**	**Vol.**	**Pages**	**Journal**
Abarca, C.; Silva, E.; Sepulveda, M. J.; Oliva, P.; Contreras, E.	Neurochemical changes after morphine, dizocilpine or riluzole in the ventral posterolateral thalamic nuclei of rats with hyperalgesia	2000	403	67-74	Eur J Pharmacol
Abarca, J.; Bustos, G.	Differential regulation of glutamate, aspartate and gamma-amino-butyrate release by N-methyl-D-aspartate receptors in rat striatum after partial and extensive lesions to the nigro-striatal dopamine pathway	1999	35	19-33	Neurochem Int
Abarca, J.; Gysling, K.; Roth, R. H.; Bustos, G.	Changes in extracellular levels of glutamate and aspartate in rat substantia nigra induced by dopamine receptor ligands: in vivo microdialysis studies	1995	20	159-169	Neurochem Res
Abraham, I.; Juhasz, G.; Kekesi, K. A.; Kovacs, K. J.	Corticosterone peak is responsible for stress-induced elevation of glutamate in the hippocampus	1998	2	171-181	Stress
Abraham, I.; Juhasz, G.; Kekesi, K. A.; Kovacs, K. J.	Effect of intrahippocampal dexamethasone on the levels of amino acid transmitters and neuronal excitability	1996	733	56-63	Brain Res
Acworth, I. N.; Yu, J.; Ryan, E.; Gariepy, K. C.; Gamache, P.; Hull, K.; Maher, T.	Simultaneous measurement of monoamine, amino acid, and drug levels, using high performance liquid chromatography and coulometric array technology: Application to in vivo microdialysis perfusate analysis	1994	17	685-705	J Liq Chromatogr
Adachi, N.; Chen, J.; Liu, K.; Nagaro, T.; Arai, T.	Metyrapone alleviates ischemic neuronal damage in the gerbil hippocampus	1999	373	147-152	Eur J Pharmacol
Adachi, N.; Chen, J.; Nakanishi, K.; Arai, T.	Pre-ischaemic administration of procaine suppresses ischaemic glutamate release and reduces neuronal damage in the gerbil hippocampus	1999	83	472-474	Br J Anaesth
Adachi, N.; Seyfried, F. J.; Arai, T.	Blockade of central histaminergic H2 receptors aggravates ischemic neuronal damage in gerbil hippocampus	2001	29	1189-1194	Crit Care Med
Aghajanian, G. K.; Kogan, J. H.; Moghaddam, B.	Opiate withdrawal increases glutamate and aspartate efflux in the locus coeruleus: an in vivo microdialysis study	1994	636	126-30	Brain Res
Ahmad, S.; Fowler, L. J.; Whitton, P. S.	Lamotrigine, carbamazepine and phenytoin differentially alter extracellular levels of 5-hydroxytryptamine, dopamine and amino acids	2005	63	141-149	Epilepsy Res
Ahmad, S.; Fowler, L. J.; Whitton, P. S.	Effects of combined lamotrigine and valproate on basal and	2005	371	1-8	Naunyn Schmiedebergs

Aspartate					
**Authors**	**Title**	**Year**	**Vol.**	**Pages**	**Journal**
	stimulated extracellular amino acids and monoamines in the hippocampus of freely moving rats				Arch Pharmacol
Ahmad, S.; Fowler, L. J.; Whitton, P. S.	Effects of acute and chronic lamotrigine treatment on basal and stimulated extracellular amino acids in the hippocampus of freely moving rats	2004	1029	41-47	Brain Res
Albrecht, J.; Hilgier, W.; Zielinska, M.; Januszewski, S.; Hesselink, M.; Quack, G.	Extracellular concentrations of taurine, glutamate, and aspartate in the cerebral cortex of rats at the asymptomatic stage of thioacetamide-induced hepatic failure: modulation by ketamine anesthesia	2000	25	1497-14502	Neurochem Res
Alexander, G. M.; Deitch, J. S.; Seeburger, J. L.; Del Valle, L.; Heiman-Patterson, T. D.	Elevated cortical extracellular fluid glutamate in transgenic mice expressing human mutant (G93A) Cu/Zn superoxide dismutase	2000	74	1666-1673	J Neurochem
Almaguer-Melian, W.; Cruz-Aguado, R.; Riva Cde, L.; Kendrick, K. M.; Frey, J. U.; Bergado, J.	Effect of LTP-reinforcing paradigms on neurotransmitter release in the dentate gyrus of young and aged rats	2005	327	877-883	Biochem Biophys Res Commun
Amakawa, K.; Adachi, N.; Liu, K.; Ikemune, K.; Fujitani, T.; Arai, T.	Effects of pre- and postischemic administration of thiopental on transmitter amino acid release and histologic outcome in gerbils	1996	85	1422-1430	Anesthesiology
Amantea, D.; Fratto, V.; Maida, S.; Rotiroti, D.; Ragusa, S.; Nappi, G.; Bagetta, G.; Corasaniti, M. T.	Prevention of Glutamate Accumulation and Upregulation of Phospho-Akt may Account for Neuroprotection Afforded by Bergamot Essential Oil against Brain Injury Induced by Focal Cerebral Ischemia in Rat	2009	85	389-405	Int Rev Neurobiol
Anderson, J. J.; DiMicco, J. A.	The use of microdialysis for studying the regional effects of pharmacological manipulation on extracellular levels of amino acids--some methodological aspects	1992	51	623-630	Life Sci
Anderson, J. J.; DiMicco, J. A.	Effect of local inhibition of gamma-aminobutyric acid uptake in the dorsomedial hypothalamus on extracellular levels of gamma-aminobutyric acid and on stress-induced tachycardia: a study using microdialysis	1990	255	1399-1407	J Pharmacol Exp Ther
Anderzhanova, E.; Rayevsky, K. S.; Saransaari, P.; Riitamaa, E.; Oja, S. S.	Effects of acute toxic doses of psychostimulants on extracellular levels of excitatory amino acids and taurine in rats: comparison of d-amphetamine and sydnocarb	2002	965	193-203	Ann N Y Acad Sci
Anderzhanova, E.; Rayevsky, K. S.; Saransaari, P.; Riitamaa, E.; Oja, S. S.	Effects of sydnocarb and D-amphetamine on the extracellular levels of amino acids in the rat caudate-putamen	2001	428	87-95	Eur J Pharmacol
Andiarzhanova, E. A.; Saransaari, R.;	[Extracellular level of neuroactive amino acids in the rat	2002	65	7-13	Eksp Klin Farmakol

Aspartate					
**Authors**	**Title**	**Year**	**Vol.**	**Pages**	**Journal**
Riitamaa, E.; Oiia, S. S.; Raevskii, K. S.	neostriatum after treatment with psychostimulants (microdialysis study)]				
Andine, P.; Orwar, O.; Jacobson, I.; Sandberg, M.; Hagberg, H.	Changes in extracellular amino acids and spontaneous neuronal activity during ischemia and extended reflow in the CA1 of the rat hippocampus	1991	57	2222-2229	J Neurochem
Andine, P.; Rudolphi, K. A.; Fredholm, B. B.; Hagberg, H.	Effect of propentofylline (HWA 285) on extracellular purines and excitatory amino acids in CA1 of rat hippocampus during transient ischaemia	1990	100	814-818	Br J Pharmacol
Arvin, B.; Lekieffre, D.; Graham, J. L.; Moncada, C.; Chapman, A. G.; Meldrum, B. S.	Effect of the non-NMDA receptor antagonist GYKI 52466 on the microdialysate and tissue concentrations of amino acids following transient forebrain ischaemia	1994	62	1458-1467	J Neurochem
Arvin, B.; Moncada, C.; Le Peillet, E.; Chapman, A.; Meldrum, B. S.	GYKI 52466 blocks the increase in extracellular glutamate induced by ischaemia	1992	3	235-238	Neuroreport
Ashworth-Preece, M. A.; Jarrott, B.; Lawrence, A. J.	Nicotinic acetylcholine receptor mediated modulation of evoked excitatory amino acid release in the nucleus tractus solitarius of the rat: evidence from in vivo microdialysis	1998	806	287-291	Brain Res
Ashworth-Preece, M. A.; Jarrott, B.; Lawrence, A. J.	5-Hydroxytryptamine3 receptor modulation of excitatory amino acid release in the rat nucleus tractus solitarius	1995	191	75-78	Neurosci Lett
Ashworth-Preece, M.; Jarrott, B.; Lawrence, A. J.	Nicotinic acetylcholine receptors in the rat and primate nucleus tractus solitarius and on rat and human inferior vagal (nodose) ganglia: evidence from in vivo microdialysis and [125I]alpha-bungarotoxin autoradiography	1998	83	1113-1122	Neuroscience
Badiani, A.; Oates, M. M.; Fraioli, S.; Browman, K. E.; Ostrander, M. M.; Xue, C. J.; Wolf, M. E.; Robinson, T. E.	Environmental modulation of the response to amphetamine: dissociation between changes in behavior and changes in dopamine and glutamate overflow in the rat striatal complex	2000	151	166-174	Psychopharmacology (Berl)
Baker, A. J.; Zornow, M. H.; Grafe, M. R.; Scheller, M. S.; Skilling, S. R.; Smullin, D. H.; Larson, A. A.	Hypothermia prevents ischemia-induced increases in hippocampal glycine concentrations in rabbits	1991	22	666-673	Stroke
Baker, A. J.; Zornow, M. H.; Scheller, M. S.; Yaksh, T. L.; Skilling, S. R.; Smullin, D. H.; Larson, A. A.; Kuczenski, R.	Changes in extracellular concentrations of glutamate, aspartate, glycine, dopamine, serotonin, and dopamine metabolites after transient global ischemia in the rabbit brain	1991	57	1370-1379	J Neurochem
Bakkelund, A. H.; Fonnum, F.; Paulsen, R. E.	Evidence using in vivo microdialysis that aminotransferase activities are important in the regulation of the pools of	1993	18	411-415	Neurochem Res

Aspartate					
**Authors**	**Title**	**Year**	**Vol.**	**Pages**	**Journal**
	transmitter amino acids				
Baldwin, H. A.; Williams, J. L.; Snares, M.; Ferreira, T.; Cross, A. J.; Green, A. R.	Attenuation by chlormethiazole administration of the rise in extracellular amino acids following focal ischaemia in the cerebral cortex of the rat	1994	112	188-194	Br J Pharmacol
Ballini, C.; Corte, L. D.; Pazzagli, M.; Colivicchi, M. A.; Pepeu, G.; Tipton, K. F.; Giovannini, M. G.	Extracellular levels of brain aspartate, glutamate and GABA during an inhibitory avoidance response in the rat	2008	106	1035-1043	J Neurochem
Battaglia, G.; Monn, J. A.; Schoepp, D. D.	In vivo inhibition of veratridine-evoked release of striatal excitatory amino acids by the group II metabotropic glutamate receptor agonist LY354740 in rats	1997	229	161-164	Neurosci Lett
Bealer, S. L.; Crowley, W. R.	Histaminergic control of oxytocin release in the paraventricular nucleus during lactation in rats	2001	171	317-322	Exp Neurol
Behrens, P. F.; Langemann, H.; Strohschein, R.; Draeger, J.; Hennig, J.	Extracellular glutamate and other metabolites in and around RG2 rat glioma: an intracerebral microdialysis study	2000	47	11-22	J Neurooncol
Beitz, A. J.	Relationship of glutamate and aspartate to the periaqueductal gray-raphe magnus projection: analysis using immunocytochemistry and microdialysis	1990	38	1755-1765	J Histochem Cytochem
Beitz, A. J.; Saxon, D.	Harmaline-induced climbing fiber activation causes amino acid and peptide release in the rodent cerebellar cortex and a unique temporal pattern of Fos expression in the olivo-cerebellar pathway	2004	33	49-74	J Neurocytol
Benturquia, N.; Parrot, S.; Sauvinet, V.; Renaud, B.; Denoroy, L.	Simultaneous determination of vigabatrin and amino acid neurotransmitters in brain microdialysates by capillary electrophoresis with laser-induced fluorescence detection	2004	806	237-244	J Chromatogr B Analyt Technol Biomed Life Sci
Benveniste, H.; Drejer, J.; Schousboe, A.; Diemer, N. H.	Elevation of the extracellular concentrations of glutamate and aspartate in rat hippocampus during transient cerebral ischemia monitored by intracerebral microdialysis	1984	43	1369-1374	J Neurochem
Bereiter, D. A.; Benetti, A. P.	Excitatory amino release within spinal trigeminal nucleus after mustard oil injection into the temporomandibular joint region of the rat	1996	67	451-459	Pain
Bereiter, D. A.; Shen, S.; Benetti, A. P.	Sex differences in amino acid release from rostral trigeminal subnucleus caudalis after acute injury to the TMJ region	2002	98	89-99	Pain
Bergquist, J.; Vona, M. J.; Stiller, C. O.;	Capillary electrophoresis with laser-induced fluorescence	1996	65	33-42	J Neurosci Methods

Aspartate					
**Authors**	**Title**	**Year**	**Vol.**	**Pages**	**Journal**
Connor, W. T.; Falkenberg, T.; Ekman, R.	detection: a sensitive method for monitoring extracellular concentrations of amino acids in the periaqueductal grey matter				
Bert, L.; Parrot, S.; Robert, F.; Desvignes, C.; Denoroy, L.; Suaud-Chagny, M. F.; Renaud, B.	In vivo temporal sequence of rat striatal glutamate, aspartate and dopamine efflux during apomorphine, nomifensine, NMDA and PDC in situ administration	2002	43	825-835	Neuropharmacol
Bianchi, L.; Ballini, C.; Colivicchi, M. A.; Della Corte, L.; Giovannini, M. G.; Pepeu, G.	Investigation on acetylcholine, aspartate, glutamate and GABA extracellular levels from ventral hippocampus during repeated exploratory activity in the rat	2003	28	565-573	Neurochem Res
Bianchi, L.; Colivicchi, M. A.; Bolam, J. P.; Della Corte, L.	The release of amino acids from rat neostriatum and substantia nigra in vivo: a dual microdialysis probe analysis	1998	87	171-180	Neuroscience
Bianchi, L.; Della Corte, L.; Tipton, K. F.	Simultaneous determination of basal and evoked output levels of aspartate, glutamate, taurine and 4-aminobutyric acid during microdialysis and from superfused brain slices	1999	723	47-59	J Chromatogr B Biomed Sci Appl
Bie, X.; Chen, Y.; Han, J.; Dai, H.; Wan, H.; Zhao, T.	Effects of gastrodin on amino acids after cerebral ischemia-reperfusion injury in rat striatum	2007	16 Suppl1	305-308	Asia Pac J Clin Nutr
Biggs, C. S.; Fowler, L. J.; Whitton, P. S.; Starr, M. S.	Extracellular levels of glutamate and aspartate in the entopeduncular nucleus of the rat determined by microdialysis: regulation by striatal dopamine D2 receptors via the indirect striatal output pathway?	1997	753	163-175	Brain Res
Biggs, C. S.; Pearce, B. R.; Fowler, L. J.; Whitton, P. S.	Effect of isonicotinic acid hydrazide on extracellular amino acids and convulsions in the rat: Reversal of neurochemical and behavioural deficits by sodium valproate	1994	63	2197-2201	J Neurochem
Biggs, C. S.; Pearce, B. R.; Fowler, L. J.; Whitton, P. S.	The effect of sodium valproate on extracellular GABA and other amino acids in the rat ventral hippocampus: an in vivo microdialysis study	1992	594	138-142	Brain Res
Bland, S. T.; Gonzales, R. A.; Schallert, T.	Movement-related glutamate levels in rat hippocampus, striatum, and sensorimotor cortex	1999	277	119-122	Neurosci Lett
Bloc, A.; Samuel, D.; Forni, C.; Dusticier, N.; Goff, L. K. L.	Effects of ionotropic excitatory amino acid receptor antagonists on glutamate transport and transport-mediated changes in extracellular excitatory amino acids in the rat striatum	1995	64	1598-1604	J Neurochem
Boatell, M. L.; Bendahan, G.; Mahy, N.	Time-related cortical amino acid changes after basal forebrain lesion: a microdialysis study	1995	64	285-291	J Neurochem
Bockelmann, R.; Reiser, M.; Horn, T. F.;	Influence of nitric oxide synthase activity on amino acid	2000	19	423-437	Amino Acids

Aspartate					
**Authors**	**Title**	**Year**	**Vol.**	**Pages**	**Journal**
Wolf, G.	concentration in the quinolinate lesioned rat striatum: a microdialysis and histochemical study				
Bolea, I.; Colivicchi, M. A.; Ballini, C.; Fattori, M.; Marco-Contelles, J. L.; Unzeta, M.; Della Corte, L.	Protective effect of the MAO-B inhibitor PF9601N in a microdialysis model of excitotoxicity	2011	8	/	Neurodegener Dis
Bolea, I.; Colivicchi, M. A.; Ballini, C.; Marco-Contelles, J.; Tipton, K. F.; Unzeta, M.; Della Corte, L.	Neuroprotective effects of the MAO-B inhibitor, PF9601N, in an in vivo model of excitotoxicity	2014	20	641-650	CNS Neurosci Ther
Boris-Moller, F.; Wieloch, T.	The effect of 4 beta-phorbol-12,13-dibutyrate and staurosporine on the extracellular glutamate levels during ischemia in the rat striatum	1998	35	133-147	Mol Chem Neuropathol
Boris-Moller, F.; Wieloch, T.	Changes in the extracellular levels of glutamate and aspartate during ischemia and hypoglycemia. Effects of hypothermia	1998	121	277-284	Exp Brain Res
Bosch, O. J.; Sartori, S. B.; Singewald, N.; Neumann, I. D.	Extracellular amino acid levels in the paraventricular nucleus and the central amygdala in high- and low-anxiety dams rats during maternal aggression: regulation by oxytocin	2007	10	261-270	Stress
Bosman, D. K.; Deutz, N. E.; Maas, M. A.; van Eijk, H. M.; Smit, J. J.; de Haan, J. G.; Chamuleau, R. A.	Amino acid release from cerebral cortex in experimental acute liver failure, studied by in vivo cerebral cortex microdialysis	1992	59	591-599	J Neurochem
Bottcher, T.; Goiny, M.; Bering, J.; Domhof, S.; Nau, R.; Ungerstedt, U.	Regional differences in glutamine synthetase inhibition by L-methionine sulfoximine: a microdialysis study in the rabbit brain	2003	150	194-200	Exp Brain Res
Bourne, J. A.; Fosbraey, P.; Halliday, J.	Changes in striatal electroencephalography and neurochemistry induced by kainic acid seizures are modified by dopamine receptor antagonists	2001	413	189-198	Eur J Pharmacol
Bowser, M. T.; Kennedy, R. T.	In vivo monitoring of amine neurotransmitters using microdialysis with on-line capillary electrophoresis	2001	22	3668-3676	Electrophoresis
Boyd, B. W.; Witowski, S. R.; Kennedy, R. T.	Trace-level amino acid analysis by capillary liquid chromatography and application to in vivo microdialysis sampling with 10-s temporal resolution	2000	72	865-871	Anal Chem
Bradford, H. F.; Young, A. M.; Crowder, J. M.	Continuous glutamate leakage from brain cells is balanced by compensatory high-affinity reuptake transport	1987	81	296-302	Neurosci Lett
Brennan, P. A.; Kendrick, K. M.; Keverne, E. B.	Neurotransmitter release in the accessory olfactory bulb during and after the formation of an olfactory memory in mice	1995	69	1075-1086	Neuroscience

Aspartate					
**Authors**	**Title**	**Year**	**Vol.**	**Pages**	**Journal**
Britton, P.; Whitton, P. S.; Fowler, L. J.; Bowery, N. G.	Tetanus toxin-induced effects on extracellular amino acid levels in rat hippocampus: an in vivo microdialysis study	1996	67	324-329	J Neurochem
Brodie, M. E.; Opacka-Juffry, J.; Peterson, D. W.; Brown, A. W.	Neurochemical changes in hippocampal and caudate dialysates associated with early trimethyltin neurotoxicity in rats	1990	11	35-46	NeuroToxicology
Bruhn, T.; Christensen, T.; Diemer, N. H.	Evidence for increased cellular uptake of glutamate and aspartate in the rat hippocampus during kainic acid seizures. A microdialysis study using the "indicator diffusion" method	1997	26	363-371	Epilepsy Res
Brust, P.; Christensen, T.; Diemer, N. H.	Decrease of extracellular taurine in the rat dorsal hippocampus after central nervous administration of vasopressin	1992	58	1427-1431	J Neurochem
Buisson, A.; Callebert, J.; Mathieu, E.; Plotkine, M.; Boulu, R. G.	Striatal protection induced by lesioning the substantia nigra of rats subjected to focal ischemia	1992	59	1153-1157	J Neurochem
Bullock, R.; Butcher, S. P.; Chen, M. H.; Kendall, L.; McCulloch, J.	Correlation of the extracellular glutamate concentration with extent of blood flow reduction after subdural hematoma in the rat	1991	74	794-802	J Neurosurg
Buratta, S.; Hamberger, A.; Ryberg, H.; Nystrom, B.; Sandberg, M.; Mozzi, R.	Effect of serine and ethanolamine administration on phospholipid-related compounds and neurotransmitter amino acids in the rabbit hippocampus	1998	71	2145-2150	J Neurochem
Bustamante, D.; You, Z. B.; Castel, M. N.; Johansson, S.; Goiny, M.; Terenius, L.; Hokfelt, T.; Herrera-Marschitz, M.	Effect of single and repeated methamphetamine treatment on neurotransmitter release in substantia nigra and neostriatum of the rat	2002	83	645-654	J Neurochem
Bustos, G.; Abarca, J.; Forray, M. I.; Gysling, K.; Bradberry, C. W.; Roth, R. H.	Regulation of excitatory amino acid release by N-methyl-D-aspartate receptors in rat striatum: in vivo microdialysis studies	1992	585	105-115	Brain Res
Butcher, S. P.; Bullock, R.; Graham, D. I.; McCulloch, J.	Correlation between amino acid release and neuropathologic outcome in rat brain following middle cerebral artery occlusion	1990	21	1727-1733	Stroke
Butcher, S. P.; Cameron, D.; Kendall, L.; Griffiths, R.	Homocysteine-induced alterations in extracellular amino acids in rat hippocampus	1992	20	75-80	Neurochem Int
Butcher, S. P.; Hamberger, A.	In vivo studies on the extracellular, and veratrine-releasable, pools of endogenous amino acids in the rat striatum: effects of corticostriatal deafferentation and kainic acid lesion	1987	48	713-721	J Neurochem
Butcher, S. P.; Jacobson, I.; Hamberger, A.	On the epileptogenic effects of kainic acid and dihydrokainic acid in the dentate gyrus of the rat	1988	27	375-381	Neuropharmacology
Butcher, S. P.; Lazarewicz, J. W.; Hamberger, A.	In vivo microdialysis studies on the effects of decortication and excitotoxic lesions on kainic acid-induced calcium fluxes, and	1987	49	1355-1360	J Neurochem

Aspartate					
**Authors**	**Title**	**Year**	**Vol.**	**Pages**	**Journal**
	endogenous amino acid release, in the rat striatum				
Butcher, S. P.; Sandberg, M.; Hagberg, H.; Hamberger, A.	Cellular origins of endogenous amino acids released into the extracellular fluid of the rat striatum during severe insulin-induced hypoglycemia	1987	48	722-728	J Neurochem
Calvo, J. C.; Gisolfi, C. V.; Blazquez, E.; Mora, F.	Glucagon-like peptide-1(7-36)amide induces the release of aspartic acid and glutamine by the ventromedial hypothalamus of the conscious rat	1995	38	435-439	Brain Res Bull
Carboni, S.; Melis, F.; Pani, L.; Hadjiconstantinou, M.; Rossetti, Z. L.	The non-competitive NMDA-receptor antagonist MK-801 prevents the massive release of glutamate and aspartate from rat striatum induced by 1-methyl-4-phenylpyridinium (MPP)	1990	117	129-133	Neurosci Lett
Carboni, S.; Melis, F.; Vaccari, A.; Rossetti, Z. L.	NMDA receptor activation mediates glutamate and aspartate release from rat striatum: prevention by MK-801	1990	22	49	Pharmacol Res
Cassel, G. E.; Fosbraey, P.	Measurement of the oxime HI-6 after peripheral administration in tandem with neurotransmitter levels in striatal dialysates: effects of soman intoxication	1996	35	159-166	J Pharmacol Toxicol Methods
Castillo-Melendez, M.; Krstew, E.; Lawrence, A. J.; Jarrott, B.	Presynaptic adenosine A2a receptors on soma and central terminals of rat vagal afferent neurons	1994	652	137-144	Brain Res
Cellar, N. A.; Burns, S. T.; Meiners, J. C.; Chen, H.; Kennedy, R. T.	Microfluidic chip for low-flow push-pull perfusion sampling in vivo with on-line analysis of amino acids	2005	77	7067-7073	Anal Chem
Chen, J. C.; Liang, K. W.; Huang, Y. K.; Liang, C. S.; Chiang, Y. C.	Significance of glutamate and dopamine neurons in the ventral pallidum in the expression of behavioral sensitization to amphetamine	2001	68	973-983	Life Sci
Chen, J.; Adachi, N.; Tsubota, S.; Nagaro, T.; Arai, T.	Dexamethasone augments ischemia-induced extracellular accumulation of glutamate in gerbil hippocampus	1998	347	67-70	Eur J Pharmacol
Chen, J.; Xu, W.; Jiang, H.	17 beta-estradiol protects neurons from ischemic damage and attenuates accumulation of extracellular excitatory amino acids	2001	92	1520-1523	Anesth Analg
Chen, R.; Deng, Y.; Yao, J.; Kamal, G. M.; Wang, J.; Xu, F.	Assessment of amino acid neurotransmitters in rat brain microdialysis samples by high-performance liquid chromatography with coulometric detection	2015	38	1439-1447	J Liq Chromatogr Related Technol
Choi, Y. H.; Chang, N.; Fletcher, P. J.; Anderson, G. H.	Dietary protein content affects the profiles of extracellular amino acids in the medial preoptic area of freely moving rats	2000	66	1105-1118	Life Sci
Choi, Y. H.; Fletcher, P. J.; Anderson, G. H.	Extracellular amino acid profiles in the paraventricular nucleus of the rat hypothalamus are influenced by diet composition	2001	892	320-328	Brain Res

Aspartate					
**Authors**	**Title**	**Year**	**Vol.**	**Pages**	**Journal**
Conroy, B. P.; Black, D.	Lamotrigine attenuates cortical glutamate release during global cerebral ischemia in pigs on cardiopulmonary bypass	1999	90	844-854	Anesthesiology
Conroy, B. P.; Lin, C. Y.; Jenkins, L. W.; DeWitt, D. S.; Zornow, M. H.; Uchida, T.; Johnston, W. E.	Hypothermic modulation of cerebral ischemic injury during cardiopulmonary bypass in pigs	1998	88	390-402	Anesthesiology
Corsi, C.; Melani, A.; Bianchi, L.; Pedata, F.	Striatal A2A adenosine receptor antagonism differentially modifies striatal glutamate outflow in vivo in young and aged rats	2000	11	2591-2595	Neuroreport
Corsi, C.; Melani, A.; Bianchi, L.; Pepeu, G.; Pedata, F.	Striatal A2A adenosine receptors differentially regulate spontaneous and K+-evoked glutamate release in vivo in young and aged rats	1999	10	687-691	Neuroreport
Corsi, C.; Pazzagli, M.; Bianchi, L.; Della Corte, L.; Pepeu, G.; Pedata, F.	In vivo amino acid release from the striatum of aging rats: adenosine modulation	1997	18	243-250	Neurobiol Aging
Currie, P. J.; Chang, N.; Luo, S.; Anderson, G. H.	Microdialysis as a tool to measure dietary and regional effects on the complete profile of extracellular amino acids in the hypothalamus of rats	1995	57	1911-1923	Life Sci
Czell, D.; Efe, T.; Preuss, M.; Schofer, M. D.; Becker, R.	Influence of Intraventricular Application of Baclofen on Arterial Blood Pressure and Neurotransmitter Concentrations in the Hypothalamic Paraventricular Nucleus of Rats	2011	/	1-6	Neurochem Res
Da Costa, A. P.; De La Riva, C.; Guevara-Guzman, R.; Kendrick, K. M.	C-fos and c-jun in the paraventricular nucleus play a role in regulating peptide gene expression, oxytocin and glutamate release, and maternal behaviour	1999	11	2199-2210	Eur J Neurosci
Da Costa, A. P.; Guevara-Guzman, R. G.; Ohkura, S.; Goode, J. A.; Kendrick, K. M.	The role of oxytocin release in the paraventricular nucleus in the control of maternal behaviour in the sheep	1996	8	163-177	J Neuroendocrinol
Dahchour, A.; De Witte, P.	Effect of repeated ethanol withdrawal on glutamate microdialysate in the hippocampus	1999	23	1698-1703	Alcohol Clin Exp Res
Dahchour, A.; De Witte, P.	Excitatory and inhibitory amino acid changes during repeated episodes of ethanol withdrawal: an in vivo microdialysis study	2003	459	171-178	Eur J Pharmacol
Dahchour, A.; De Witte, P.	Effects of acamprosate on excitatory amino acids during multiple ethanol withdrawal periods	2003	27	465-470	Alcohol Clin Exp Res
Dahchour, A.; Hoffman, A.; Deitrich, R.; de Witte, P.	Effects of ethanol on extracellular amino acid levels in high-and low-alcohol sensitive rats: a microdialysis study	2000	35	548-553	Alcohol Alcohol
Daisley, J. N.; Gruss, M.; Rose, S. P.;	Passive avoidance training and recall are associated with	1998	6	53-61	Neural Plast

Aspartate					
**Authors**	**Title**	**Year**	**Vol.**	**Pages**	**Journal**
Braun, K.	increased glutamate levels in the intermediate medial hyperstriatum ventrale of the day-old chick				
Dalley, J. W.	Effects of excitotoxic lesions of the rat prefrontal cortex on CREB regulation and presynaptic markers of dopamine and amino acid function in the nucleus accumbens	1999	11	1265-1274	Eur J Neurosci
Daly, D. A.; Moghaddam, B.	Actions of clozapine and haloperidol on the extracellular levels of excitatory amino acids in the prefrontal cortex and striatum of conscious rats	1993	152	61-64	Neurosci Lett
Darling, B. K.; Abdel-Rahim, M.; Moores, R. R.; Chang, A. S.; Howard, R. S.; Neill, J. T.	Brain excitatory amino acid concentrations are lower in the neonatal pig: a buffer against excitotoxicity?	2001	80	305-312	Biol Neonate
David, C. N.; Frias, E. S.; Szu, J. I.; Vieira, P. A.; Hubbard, J. A.; Lovelace, J.; Michael, M.; Worth, D.; McGovern, K. E.; Ethell, I. M.; Stanley, B. G.; Korzus, E.; Fiacco, T. A.; Binder, D. K.; Wilson, E. H.	GLT-1-Dependent Disruption of CNS Glutamate Homeostasis and Neuronal Function by the Protozoan Parasite Toxoplasma gondii	2016	12	e1005643	PLoS Pathog
Dawson, L. A.; Djali, S.; Gonzales, C.; Vinegra, M. A.; Zaleska, M. M.	Characterization of transient focal ischemia-induced increases in extracellular glutamate and aspartate in spontaneously hypertensive rats	2000	53	767-776	Brain Res Bull
Dawson, L. A.; Nguyen, H. Q.; Li, P.	In vivo effects of the 5-HT(6) antagonist SB-271046 on striatal and frontal cortex extracellular concentrations of noradrenaline, dopamine, 5-HT, glutamate and aspartate	2000	130	23-26	Br J Pharmacol
Dawson, L. A.; Organ, A. J.; Winter, P.; Lacroix, L. P.; Shilliam, C. S.; Heidbreder, C.; Shah, A. J.	Rapid high-throughput assay for the measurement of amino acids from microdialysates and brain tissue using monolithic C18-bonded reversed-phase columns	2004	807	235-241	J Chromatogr B Analyt Technol Biomed Life Sci
Dawson, L. A.; Stow, J. M.; Palmer, A. M.	Improved method for the measurement of glutamate and aspartate using capillary electrophoresis with laser induced fluorescence detection and its application to brain microdialysis	1997	694	455-460	J Chromatogr B Biomed Sci Appl
Del Arco, A.; Gonzalez-Mora, J. L.; Armas, V. R.; Mora, F.	Amphetamine increases the extracellular concentration of glutamate in striatum of the awake rat: involvement of high affinity transporter mechanisms	1999	38	943-954	Neuropharmacology
Delgado, J. M.; DeFeudis, F. V.; Roth, R. H.; Ryugo, D. K.; Mitruka, B. M.	Dialytrode for long term intracerebral perfusion in awake monkeys	1972	198	9-21	Arch Int Pharmacodyn Ther

Aspartate					
**Authors**	**Title**	**Year**	**Vol.**	**Pages**	**Journal**
Deng, Y.; Wang, M.; Jiang, L.; Ma, C.; Xi, Z.; Li, X.; He, N.	A comparison of extracellular excitatory amino acids release inhibition of acute lamotrigine and topiramate treatment in the hippocampus of PTZ-kindled epileptic rats	2013	9	1123-1128	J Biomed Nanotechnol
Deng, Y.; Wang, M.; Wang, W.; Ma, C.; He, N.	Comparison and effects of acute lamotrigine treatment on extracellular excitatory amino acids in the hippocampus of PTZ-kindled epileptic and PTZ-induced status epilepticus rats	2013	38	504-511	Neurochem Res
Devall, A. J.; Blake, R.; Langman, N.; Smith, C. G.; Richards, D. A.; Whitehead, K. J.	Monolithic column-based reversed-phase liquid chromatography separation for amino acid assay in microdialysates and cerebral spinal fluid	2007	848	323-328	J Chromatogr B Analyt Technol Biomed Life Sci
Di Cara, B.; Dusticier, N.; Forni, C.; Lievens, J. C.; Daszuta, A.	Serotonin depletion produces long lasting increase in striatal glutamatergic transmission	2001	78	240-248	J Neurochem
Di, X.; Harpold, T.; Watson, J. C.; Bullock, M. R.	Excitotoxic damage in neurotrauma: fact or fiction	1996	9	231-241	Restor Neurol Neurosci
Dijk, S. N.; Francis, P. T.; Stratmann, G. C.; Bowen, D. M.	NMDA-induced glutamate and aspartate release from rat cortical pyramidal neurones: evidence for modulation by a 5-HT1A antagonist	1995	115	1169-1174	Br J Pharmacol
Dijk, S. N.; Francis, P. T.; Stratmann, G. C.; Bowen, D. M.	Cholinomimetics increase glutamate outflow via an action on the corticostriatal pathway: implications for Alzheimer's disease	1995	65	2165-2219	J Neurochem
Donnell, J. C.; McDonough, J. H.; Shih, T. M.	In vivo microdialysis and electroencephalographic activity in freely moving guinea pigs exposed to organophosphorus nerve agents sarin and VX: analysis of acetylcholine and glutamate	2011	85	1607-1616	Arch Toxicol
Donzanti, B. A.; Yamamoto, B. K.	An improved and rapid HPLC-EC method for the isocratic separation of amino acid neurotransmitters from brain tissue and microdialysis perfusates	1988	43	913-922	Life Sci
Doretto, M. C.; Burger, R. L.; Mishra, P. K.; Garcia-Cairasco, N.; Dailey, J. W.; Jobe, P. C.	A microdialysis study of amino acid concentrations in the extracellular fluid of the substantia nigra of freely behaving GEPR-9s: relationship to seizure predisposition	1994	17	157-165	Epilepsy Res
During, M. J.; Craig, J. S.; Hernandez, T. D.; Anderson, G. M.; Gallager, D. W.	Effect of amygdala kindling on the in vivo release of GABA and 5-HT in the dorsal raphe nucleus in freely moving rats	1992	584	36-44	Brain Res
Dzahini, K.; Dentresangle, C.; Le Cavorsin, M.; Bertrand, A.; Detraz, I.; Savasta, M.; Leviel, V.	Pre-synaptic glutamate-induced activation of DA release in the striatum after partial nigral lesion	2010	113	1459-1470	J Neurochem
Ebner, K.; Bosch, O. J.; Kromer, S. A.;	Release of oxytocin in the rat central amygdala modulates	2005	30	223-230	Neuropsychopharmacology

Aspartate					
**Authors**	**Title**	**Year**	**Vol.**	**Pages**	**Journal**
Singewald, N.; Neumann, I. D.	stress-coping behavior and the release of excitatory amino acids				
Ehlen, J. C.; Albers, H. E.; Breyer, E. D.	MEKC-LIF of gamma-amino butyric acid in microdialysate: Systematic optimization of the separation conditions by factorial analysis	2005	147	36-47	J Neurosci Methods
el-Yamany, N. A.; Horn, E.	Time courses of aspartate and glutamate concentrations in the focus area during penicillin induced epileptiform activity in awake rats	2002	140	13-30	Arch Ital Biol
Engelmann, M.; Ludwig, M.; Singewald, N.; Ebner, K.; Sabatier, N.; Lubec, G.; Landgraf, R.; Wotjak, C. T.	Taurine selectively modulates the secretory activity of vasopressin neurons in conscious rats	2001	14	1047-1055	Eur J Neurosci
Engelmann, M.; Wolf, G.; Horn, T. F.	Release patterns of excitatory and inhibitory amino acids within the hypothalamic supraoptic nucleus in response to direct nitric oxide administration during forced swimming in rats	2002	324	252-254	Neurosci Lett
Espey, M. G.; Kustova, Y.; Sei, Y.; Basile, A. S.	Extracellular glutamate levels are chronically elevated in the brains of LP-BM5-infected mice: a mechanism of retrovirus-induced encephalopathy	1998	71	2079-2087	J Neurochem
Fabre-Nys, C.; Blache, D.; Hinton, M. R.; Goode, J. A.; Kendrick, K. M.	Microdialysis measurement of neurochemical changes in the mediobasal hypothalamus of ovariectomized ewes during oestrus	1994	649	282-296	Brain Res
Fabricius, M.; Jensen, L. H.; Lauritzen, M.	Microdialysis of interstitial amino acids during spreading depression and anoxic depolarization in rat neocortex	1993	612	61-69	Brain Res
Faden, A. I.	Dynorphin increases extracellular levels of excitatory amino acids in the brain through a non-opioid mechanism	1992	12	425-429	J Neurosci
Fallgren, A. B.; Paulsen, R. E.	A microdialysis study in rat brain of dihydrokainate, a glutamate uptake inhibitor	1996	21	19-25	Neurochem Res
Favalli, L.; Rozza, A.; Frattini, P.; Masoero, E.; Scelsi, R.; Pascale, A.; Govoni, S.	Ischemia-induced glutamate release in rat frontoparietal cortex after chronic alcohol and withdrawal	2002	326	183-186	Neurosci Lett
Fedele, E.; Foster, A. C.	An evaluation of the role of extracellular amino acids in the delayed neurodegeneration induced by quinolinic acid in the rat striatum	1993	52	911-917	Neuroscience
Fedele, E.; Varnier, G.; Ansaldo, M. A.; Raiteri, M.	Nicotine administration stimulates the in vivo N-methyl-D-aspartate receptor/nitric oxide/cyclic GMP pathway in rat	1998	125	1042-1048	Br J Pharmacol

Aspartate					
**Authors**	**Title**	**Year**	**Vol.**	**Pages**	**Journal**
	hippocampus through glutamate release				
Feet, B. A.; Gilland, E.; Groenendaal, F.; Brun, N. C.; Hellstrom-Westas, L.; Hagberg, H.; Saugstad, O. D.	Cerebral excitatory amino acids and Na+ K+ -ATPase activity during resuscitation of severely hypoxic newborn piglets	1998	87	889-895	Acta Paediatr
Feng, Y. Z.; Zhang, T.; Tokuyama, S.; Zhu, H.; Rockhold, R. W.; Ho, I. K.	Mu- and delta-opioid receptor antagonists precipitate similar withdrawal phenomena in butorphanol and morphine dependence	1996	21	63-71	Neurochem Res
Feng, Y.; Rockhold, R. W.; Ho, I. K.	Nor-binaltorphimine precipitates withdrawal and excitatory amino acid release in the locus ceruleus of butorphanol--but not morphine-dependent rats	1997	283	932-938	J Pharmacol Exp Ther
Foster, S. B.; Tang, H.; Miller, K. E.; Dryhurst, G.	Increased extracellular glutamate evoked by 1-methyl-4-phenylpyridinium [MPP(+)] in the rat striatum is not essential for dopaminergic neurotoxicity and is not derived from released glutathione	2005	7	251-263	Neurotox Res
Frantz, K.; Harte, M.; Ungerstedt, U.; Connor, W. T.	A dual probe characterization of dialysate amino acid levels in the medial prefrontal cortex and ventral tegmental area of the awake freely moving rat	2002	119	109-119	J Neurosci Methods
Fraser, M.; Bennet, L.; Van Zijl, P. L.; Mocatta, T. J.; Williams, C. E.; Gluckman, P. D.; Winterbourn, C. C.; Gunn, A. J.	Extracellular amino acids and lipid peroxidation products in periventricular white matter during and after cerebral ischemia in preterm fetal sheep	2008	105	2214-2223	J Neurochem
Freedman, S. B.; Patel, S.; Smith, A. J.; Chapman, K.; Fletcher, A.; Kemp, J. A.; Marshall, G. R.; Hargreaves, R. J.; Scholey, K.; Mellin, E. C.; DiPardo, R. M.; Bock, M. G.; Freidinger, R. M.	A second generation of non-peptide cholecystokinin receptor antagonists and their possible therapeutic potential	1994	713	312-318	Ann N Y Acad Sci
Freinbichler, W.; Colivicchi, M. A.; Fattori, M.; Ballini, C.; Tipton, K. F.; Linert, W.; Della Corte, L.	Validation of a robust and sensitive method for detecting hydroxyl radical formation together with evoked neurotransmitter release in brain microdialysis	2008	105	738-749	J Neurochem
Fu, Y.; He, F.; Zhang, S.; Huang, J.; Zhang, J.; Jiao, X.	3-Nitropropionic acid produces indirect excitotoxic damage to rat striatum	1995	17	333-339	Neurotoxicol Teratol
Fujikawa, D. G.	Effects of N-methyl-D-aspartate-receptor blockade on high-potassium-induced neuronal death and glutamate release	1997	226	25-28	Neurosci Lett
Fujikawa, D. G.; Kim, J. S.; Daniels, A. H.;	In vivo elevation of extracellular potassium in the rat amygdala	1996	74	695-706	Neuroscience

Aspartate					
**Authors**	**Title**	**Year**	**Vol.**	**Pages**	**Journal**
Alcaraz, A. F.; Sohn, T. B.	increases extracellular glutamate and aspartate and damages neurons				
Fujisawa, H.; Koizumi, H.; Ito, H.; Yamashita, K.; Maekawa, T.	Effects of mild hypothermia on the cortical release of excitatory amino acids and nitric oxide synthesis following hypoxia	1999	16	1083-1093	J Neurotrauma
Fujita, T.; Kamisaki, Y.; Yonehara, N.	Nitric oxide-induced increase of excitatory amino acid levels in the trigeminal nucleus caudalis of the rat with tactile hypersensitivity evoked by the loose-ligation of the inferior alveolar nerves	2004	91	558-567	J Neurochem
Fujitani, T.; Adachi, N.; Miyazaki, H.; Liu, K.; Nakamura, Y.; Kataoka, K.; Arai, T.	Lidocaine protects hippocampal neurons against ischemic damage by preventing increase of extracellular excitatory amino acids: a microdialysis study in Mongolian gerbils	1994	179	91-94	Neurosci Lett
Galeffi, F.; Bianchi, L.; Bolam, J. P.; Della Corte, L.	The effect of 6-hydroxydopamine lesions on the release of amino acids in the direct and indirect pathways of the basal ganglia: a dual microdialysis probe analysis	2003	18	856-868	Eur J Neurosci
Gallegos-Sanchez, J.; Picard, S.; Delaleu, B.; Malpaux, B.; Thiery, J. C.	Initiation of the oestradiol-induced inhibition of pulsatile LH secretion in ewes under long days: comparison of peripheral versus central treatment and neurochemical correlates	1996	151	19-28	J Endocrinol
Ganguly, P. K.; Maddaford, T. G.; Edel, A. L.; O, K.; Smeda, J. S.; Pierce, G. N.	Increased homocysteine-induced release of excitatory amino acids in the striatum of spontaneously hypertensive stroke-prone rats	2008	1226	192-198	Brain Res
Ge, J.; Long, S. K.; Kilpatrick, I. C.	Preferential blockade of cholecystokinin-8S-induced increases in aspartate and glutamate levels by the CCK(B) receptor antagonist, L-365,260, in rat brain	1998	345	163-170	Eur J Pharmacol
Ghijsen, W. E.; da Silva Aresta Belo, A. I.; Zuiderwijk, M.; Lopez da Silva, F. H.	Compensatory change in EAAC1 glutamate transporter in rat hippocampus CA1 region during kindling epileptogenesis	1999	276	157-160	Neurosci Lett
Ghribi, O.; Callebert, J.; Plotkine, M.; Boulu, R. G.	L-NAME modulates glutamate accumulation induced by K(+)-depolarization but not by forebrain ischaemia in the rat striatum	1994	174	34-38	Neurosci Lett
Ghribi, O.; Callebert, J.; Plotkine, M.; Boulu, R. G.	Effect of kynurenic acid on the ischaemia-induced accumulation of glutamate in rat striatum	1994	5	435-437	Neuroreport
Ghribi, O.; Callebert, J.; Plotkine, M.; Boulu, R. G.	Competitive NMDA receptor blockers reduce striatal glutamate accumulation in ischaemia	1994	5	1253-1255	Neuroreport
Ghribi, O.; Callebert, J.; Verrecchia, C.; Plotkine, M.; Boulu, R. G.	Blockers of NMDA-operated channels decrease glutamate and aspartate extracellular accumulation in striatum during forebrain	1995	9	141-146	Fundam Clin Pharmacol

Aspartate					
**Authors**	**Title**	**Year**	**Vol.**	**Pages**	**Journal**
	ischaemia in rats				
Glass, J. D.; Hauser, U. E.; Blank, J. L.; Selim, M.; Rea, M. A.	Differential timing of amino acid and 5-HIAA rhythms in suprachiasmatic hypothalamus	1993	265	R504-R511	Am J Physiol
Gobert, A.; Rivet, J. M.; Billiras, R.; Parsons, F.; Millan, M. J.	Simultaneous quantification of D- vs. L-serine, taurine, kynurenate, phosphoethanolamine and diverse amino acids in frontocortical dialysates of freely-moving rats: differential modulation by N-methyl-D-aspartate (NMDA) and other pharmacological agents	2011	202	143-157	J Neurosci Methods
Goda, H.; Ooboshi, H.; Nakane, H.; Ibayashi, S.; Sadoshima, S.; Fujishima, M.	Modulation of ischemia-evoked release of excitatory and inhibitory amino acids by adenosine A1 receptor agonist	1998	357	149-155	Eur J Pharmacol
Godukhin, O.	Effect of local cholecystokinin-8 administration on extracellular levels of amino acids and glycolytic products monitored by in vivo microdialysis in the fronto-parietal cortex of the rat	1995	194	29-32	Neurosci Lett
Goldsmith, J. D.; Kujawa, S. G.; McLaren, J. D.; Bledsoe Jr, S. C.	In vivo release of neuroactive amino acids from the inferior colliculus of the guinea pig using brain microdialysis	1995	83	80-88	Hear Res
Golembiowska, K.; Dziubina, A.	Effect of acute and chronic administration of citalopram on glutamate and aspartate release in the rat prefrontal cortex	2000	52	441-448	Pol J Pharmacol
Golembiowska, K.; Dziubina, A.	Inhibition of amino acid release by 5-HT1B receptor agonist in the rat prefrontal cortex	2002	54	625-631	Pol J Pharmacol
Golembiowska, K.; Dziubina, A.	Involvement of adenosine in the effect of antidepressants on glutamate and aspartate release in the rat prefrontal cortex	2001	363	663-670	Naunyn Schmiedebergs Arch Pharmacol
Golembiowska, K.; Zylewska, A.	Effect of antidepressant drugs on veratridine-evoked glutamate and aspartate release in rat prefrontal cortex	1999	51	63-70	Pol J Pharmacol
Gong, J.; Gong, S.; Zhang, M.; Zhang, L.; Hu, Y.; Liu, Y.; Li, W.	Cerebral ischemic preconditioning reduces glutamate excitotoxicity by up-regulating the uptake activity of GLT-1 in rats	2014	46	1537-1545	Amino Acids
Grabb, M. C.; Sciotti, V. M.; Gidday, J. M.; Cohen, S. A.; van Wylen, D. G.	Neurochemical and morphological responses to acutely and chronically implanted brain microdialysis probes	1998	82	25-34	J Neurosci Methods
Graham, S. H.; Shimizu, H.; Newman, A.; Weinstein, P.; Faden, A. I.	Opioid receptor antagonist nalmefene stereospecifically inhibits glutamate release during global cerebral ischemia	1993	632	346-350	Brain Res
Graham, S. H.; Shiraishi, K.; Panter, S. S.; Simon, R. P.; Faden, A. I.	Changes in extracellular amino acid neurotransmitters produced by focal cerebral ischemia	1990	110	124-130	Neurosci Lett
Gruss, M.; Braun, K.	Stimulus-evoked increase of glutamate in the mediorostral	1996	66	1167-	J Neurochem

Aspartate					
**Authors**	**Title**	**Year**	**Vol.**	**Pages**	**Journal**
	neostriatum/hyperstriatum ventrale of domestic chick after auditory filial imprinting: an in vivo microdialysis study			1173	
Guerra-Romero, L.; Tureen, J. H.; Fournier, M. A.; Makrides, V.; Tauber, M. G.	Amino acids in cerebrospinal and brain interstitial fluid in experimental pneumococcal meningitis	1993	33	510-513	Pediatr Res
Guevara-Guzman, R.; Barrera-Mera, B.; De La Riva, C.; Kendrick, K. M.	Release of classical transmitters and nitric oxide in the rat olfactory bulb, evoked by vaginocervical stimulation and potassium, varies with the oestrus cycle	2000	12	80-88	Eur J Neurosci
Guevara-Guzman, R.; Emson, P. C.; Kendrick, K. M.	Modulation of in vivo striatal transmitter release by nitric oxide and cyclic GMP	1994	62	807-810	J Neurochem
Hamann, M.; Sohr, R.; Morgenstern, R.; Richter, A.	Extracellular amino acid levels in the striatum of the dt(sz) mutant, a model of paroxysmal dystonia	2008	157	188-195	Neuroscience
Han, J.; Wan, H. T.; Yang, J. H.; Zhang, Y. Y.; Ge, L. J.; Bie, X. D.	Effect of ligustrazine on levels of amino acid neurotransmitters in rat striatum after cerebral ischemia-reperfusion injury	2014	16	1060-1067	J Asian Nat Prod Res
Han, Y.; Zuo, M.; Qi, L.; Liu, K.; Mao, L.; Chen, Y.	Online acid barrage stacking anti-salt injection for capillary electrophoresis of 9-fluorenylmethylchloroformate-derivatized amino acids in high ionic strength solutions by UV detection	2006	27	4240-4248	Electrophoresis
Harkany, T.; Abraham, I.; Laskay, G.; Timmerman, W.; Jost, K.; Zarandi, M.; Penke, B.; Nyakas, C.; Luiten, P. G.	Propionyl-IIGL tetrapeptide antagonizes beta-amyloid excitotoxicity in rat nucleus basalis	1999	10	1693-1698	Neuroreport
Harkany, T.; Abraham, I.; Timmerman, W.; Laskay, G.; Toth, B.; Sasvari, M.; Konya, C.; Sebens, J. B.; Korf, J.; Nyakas, C.; Zarandi, M.; Soos, K.; Penke, B.; Luiten, P. G.	beta-amyloid neurotoxicity is mediated by a glutamate-triggered excitotoxic cascade in rat nucleus basalis	2000	12	2735-2745	Eur J Neurosci
Hasegawa, H.; Yazawa, T.; Yasumatsu, M.; Otokawa, M.; Aihara, Y.	Alteration in dopamine metabolism in the thermoregulatory center of exercising rats	2000	289	161-164	Neurosci Lett
Haskew-Layton, R. E.; Rudkouskaya, A.; Jin, Y.; Feustel, P. J.; Kimelberg, H. K.; Mongin, A. A.	Two distinct modes of hypoosmotic medium-induced release of excitatory amino acids and taurine in the rat brain in vivo	2008	3	e3543	PLoS One
Hassel, B.; Dahlberg, D.; Mariussen, E.; Goverud, I. L.; Antal, E. A.; Tonjum, T.; Maehlen, J.	Brain infection with Staphylococcus aureus leads to high extracellular levels of glutamate, aspartate, gamma-aminobutyric acid, and zinc	2014	92	1792-1800	J Neurosci Res
Hathway, G. J.; Emson, P. C.; Humphrey,	Somatostatin potently stimulates in vivo striatal dopamine and	1998	70	1740-9	J Neurochem

Aspartate					
**Authors**	**Title**	**Year**	**Vol.**	**Pages**	**Journal**
P. P.; Kendrick, K. M.	gamma-aminobutyric acid release by a glutamate-dependent action				
Hazell, A. S.; Butterworth, R. F.; Hakim, A. M.	Cerebral vulnerability is associated with selective increase in extracellular glutamate concentration in experimental thiamine deficiency	1993	61	1155-1158	J Neurochem
Hehre, D. A.; Devia, C. J.; Bancalari, E.; Suguihara, C.	Brainstem amino acid neurotransmitters and ventilatory response to hypoxia in piglets	2008	63	46-50	Pediatr Res
Heim, C.; Zhang, J.; Lan, J.; Sieklucka, M.; Kurz, T.; Riederer, P.; Gerlach, M.; Sontag, K. H.	Cerebral oligaemia episode triggers free radical formation and late cognitive deficiencies	2000	12	715-725	Eur J Neurosci
Hemmati, P.; Shilliam, C. S.; Hughes, Z. A.; Shah, A. J.; Roberts, J. C.; Atkins, A. R.; Hunter, A. J.; Heidbreder, C. A.	In vivo characterization of basal amino acid levels in subregions of the rat nucleus accumbens: effect of a dopamine D(3)/D(2) agonist	2001	39	199-208	Neurochem Int
Herbison, A. E.; Heavens, R. P.; Dyer, R. G.	Endogenous release of gamma-aminobutyric acid from the medial preoptic area measured by microdialysis in the anaesthetised rat	1990	55	1617-1623	J Neurochem
Herbison, A. E.; Voisin, D. L.; Douglas, A. J.; Chapman, C.	Profile of monoamine and excitatory amino acid release in rat supraoptic nucleus over parturition	1997	138	33-40	Endocrinology
Herrera-Marschitz, M.; Javier Meana, J.; Hokfelt, T.; You, Z. B.; Morino, P.; Brodin, E.; Ungerstedt, U.	Cholecystokinin is released from a crossed corticostriatal pathway	1992	3	905-908	NeuroReport
Herrera-Marschitz, M.; Meana, J. J.; Connor, W. T.; Goiny, M.; Reid, M. S.; Ungerstedt, U.	Neuronal dependence of extracellular dopamine, acetylcholine, glutamate, aspartate and gamma-aminobutyric acid (GABA) measured simultaneously from rat neostriatum using in vivo microdialysis: reciprocal interactions	1992	2	157-179	Amino Acids
Herrera-Marschitz, M.; You, Z. B.; Goiny, M.; Meana, J. J.; Silveira, R.; Godukhin, O. V.; Chen, Y.; Espinoza, S.; Pettersson, E.; Loidl, C. F.; Lubec, G.; Andersson, K.; Nylander, I.; Terenius, L.; Ungerstedt, U.	On the origin of extracellular glutamate levels monitored in the basal ganglia of the rat by in vivo microdialysis	1996	66	1726-1735	J Neurochem
Herz, R. C.; Gaillard, P. J.; de Wildt, D. J.; Versteeg, D. H.	Differences in striatal extracellular amino acid concentrations between Wistar and Fischer 344 rats after middle cerebral artery occlusion	1996	715	163-171	Brain Res

Aspartate					
**Authors**	**Title**	**Year**	**Vol.**	**Pages**	**Journal**
Hilgier, W.; Zielinska, M.; Borkowska, H. D.; Gadamski, R.; Walski, M.; Oja, S. S.; Saransaari, P.; Albrecht, J.	Changes in the extracellular profiles of neuroactive amino acids in the rat striatum at the asymptomatic stage of hepatic failure	1999	56	76-84	J Neurosci Res
Hillered, L.; Hallstrom, A.; Segersvard, S.; Persson, L.; Ungerstedt, U.	Dynamics of extracellular metabolites in the striatum after middle cerebral artery occlusion in the rat monitored by intracerebral microdialysis	1989	9	607-616	J Cereb Blood Flow Metab
Honma, S.; Katsuno, Y.; Shinohara, K.; Abe, H.; Honma, K.	Circadian rhythm and response to light of extracellular glutamate and aspartate in rat suprachiasmatic nucleus	1996	271	R579-585	Am J Physiol
Hoop, B.; Beagle, J. L.; Maher, T. J.; Kazemi, H.	Brainstem amino acid neurotransmitters and hypoxic ventilatory response	1999	118	117-129	Respir Physiol
Horn, T.; Bauce, L.; Landgraf, R.; Pittman, Q. J.	Microdialysis with high NaCl causes central release of amino acids and dopamine	1995	64	1632-1644	J Neurochem
Horn, T.; Smith, P. M.; McLaughlin, B. E.; Bauce, L.; Marks, G. S.; Pittman, Q. J.; Ferguson, A. V.	Nitric oxide actions in paraventricular nucleus: cardiovascular and neurochemical implications	1994	266	R306-R313	Am J Physiol
Hoshi, K.; Ma, T.; Ho, I. K.	Precipitated kappa-opioid receptor agonist withdrawal increase glutamate in rat locus coeruleus	1996	314	301-306	Eur J Pharmacol
Hoshi, K.; Ma, T.; Oh, S.; Ho, I. K.	Increased release of excitatory amino acids in rat locus coeruleus in kappa-opioid agonist dependent rats precipitated by nor-binaltorphimine	1997	753	63-68	Brain Res
Hoshi, K.; Yamamoto, A.; Ishizuki, S.; Fujihira, E.; Ichihara, K.	Excitatory amino acid release in the locus coeruleus during naloxone-precipitated morphine withdrawal in adjuvant arthritic rats	2000	49	36-41	Inflamm Res
Huo, T.; Zhang, Y.; Li, W.; Yang, H.; Jiang, H.; Sun, G.	Effect of realgar on extracellular amino acid neurotransmitters in hippocampal CA1 region determined by online microdialysis-dansyl chloride derivatization-high-performance liquid chromatography and fluorescence detection	2014	28	1254-1262	Biomed Chromatogr
Hylland, P.; Nilsson, G. E.	Extracellular levels of amino acid neurotransmitters during anoxia and forced energy deficiency in crucian carp brain	1999	823	49-58	Brain Res
Hylland, P.; Nilsson, G. E.; Johansson, D.	Anoxic brain failure in an ectothermic vertebrate: release of amino acids and K+ in rainbow trout thalamus	1995	269	R1077-1084	Am J Physiol
Hyzinski-Garcia, M. C.; Vincent, M. Y.; Haskew-Layton, R. E.; Dohare, P.; Keller	Hypo-osmotic swelling modifies glutamate-glutamine cycle in the cerebral cortex and in astrocyte cultures	2011	118	140-152	J Neurochem

Aspartate					
**Authors**	**Title**	**Year**	**Vol.**	**Pages**	**Journal**
Jr, R. W.; Mongin, A. A.					
Ichord, R. N.; Johnston, M. V.; Traystman, R. J.	MK801 decreases glutamate release and oxidative metabolism during hypoglycemic coma in piglets	2001	128	139-148	Brain Res Dev Brain Res
Ichord, R. N.; Northington, F. J.; van Wylen, D.; Johnston, M. V.; Kwon, C.; Traystman, R. J.	Brain O2 consumption and glutamate release during hypoglycemic coma in piglets are temperature sensitive	1999	276	H2053-H2062	Am J Physiol
Iijima, T.; Iwao, Y.; Sankawa, H.	Amino acid release during spreading depression in a flow-compromised cortical area	1999	818	553-555	Brain Res
Iino, T.; Katsura, M.; Kuriyama, K.	Protective effect of vinconate on ischemia-induced neuronal damage in the rat hippocampus	1992	224	117-124	Eur J Pharmacol
Inglefield, J. R.; Perry, J. M.; Schwartz, R. D.	Postischemic inhibition of GABA reuptake by tiagabine slows neuronal death in the gerbil hippocampus	1995	5	460-468	Hippocampus
Irifune, M.; Kikuchi, N.; Saida, T.; Takarada, T.; Shimizu, Y.; Endo, C.; Morita, K.; Dohi, T.; Sato, T.; Kawahara, M.	Riluzole, a glutamate release inhibitor, induces loss of righting reflex, antinociception, and immobility in response to noxious stimulation in mice	2007	104	1415-1421	Anesth Analg
Irimia, C.; Buczynski, M.; Laredo, S.; Alvaros, N.; Parsons, L.	Dysregulated NMDA NR1 signaling in the infralimbic cortex contributes to increased impulsivity during protracted alcohol abstinence	2015	40	S600-S601	Neuropsychopharmacology
Jacobson, I.; Hagberg, H.; Sandberg, M.; Hamberger, A.	Ouabain-induced changes in extracellular aspartate, glutamate and GABA levels in the rabbit olfactory bulb in vivo	1986	64	211-215	Neurosci Lett
Jacobson, I.; Sandberg, M.; Hamberger, A.	Mass transfer in brain dialysis devices--a new method for the estimation of extracellular amino acids concentration	1985	15	263-268	J Neurosci Methods
Jacobsson, S. O.; Cassel, G. E.; Karlsson, B. M.; Sellstrom, A.; Persson, S. A.	Release of dopamine, GABA and EAA in rats during intrastriatal perfusion with kainic acid, NMDA and soman: a comparative microdialysis study	1997	71	756-765	Arch Toxicol
Jacobsson, S. O.; Sellstrom, A.; Persson, S. A.; Cassel, G. E.	Correlation between cortical EEG and striatal microdialysis in soman-intoxicated rats	1997	231	155-158	Neurosci Lett
Jellish, W. S.; Murdoch, J.; Kindel, G.; Zhang, X.; White, F. A.	The effect of clonidine on cell survival, glutamate, and aspartate release in normo- and hyperglycemic rats after near complete forebrain ischemia	2005	167	526-534	Exp Brain Res
Jin, Y.; Zhang, B. Y.; Yao, S. Y.; Liu, Z.; Liu, W. Z.; Zhao, Y. J.	Effect of activation or blocking of N-methyl-D-aspartate receptors on release of excitatory amino acids in rat brain hippocampus. [Chinese]	2005	9	141-143	Zhongguo lin chuang kang fu

Aspartate					
**Authors**	**Title**	**Year**	**Vol.**	**Pages**	**Journal**
Jones, N. M.; Lawrence, A. J.; Beart, P. M.	In vivo microdialysis reveals facilitatory metabotropic glutamate receptors regulating excitatory amino acid release in rat nucleus tractus solitarius	1998	32	31-38	Neurochem Int
Juhasz, G.; Kekesi, K. A.; Nyitrai, G.; Dobolyi, A.; Krogsgaard-Larsen, P.; Schousboe, A.	Differential effects of nipecotic acid and 4,5,6,7-tetrahydroisoxazolo[4,5-c]pyridin-3-ol on extracellular gamma-aminobutyrate levels in rat thalamus	1997	331	139-144	Eur J Pharmacol
Kahn, R. A.; Panah, M.; Kiffel, S.; Weinberger, J.	Modulation of ischemic excitatory neurotransmitter and gamma-aminobutyric acid release during global temporary cerebral ischemia by local nitric oxide synthase inhibition	1997	84	1004-1010	Anesth Analg
Kahn, R. A.; Panah, M.; Weinberger, J.	Modulation of ischemic excitatory neurotransmitter and gamma-aminobutyric acid release during global temporary cerebral ischemia by selective neuronal nitric oxide synthase inhibition	1997	84	997-1003	Anesth Analg
Kaku, T.; Jiang, M. H.; Hada, J.; Morimoto, K.; Hayashi, Y.	Sodium nitroprusside-induced seizures and adenosine release in rat hippocampus	2001	413	199-205	Eur J Pharmacol
Kanda, T.; Kurokawa, M.; Tamura, S.; Nakamura, J.; Ishii, A.; Kuwana, Y.; Serikawa, T.; Yamada, J.; Ishihara, K.; Sasa, M.	Topiramate reduces abnormally high extracellular levels of glutamate and aspartate in the hippocampus of spontaneously epileptic rats (SER)	1996	59	1607-1616	Life Sci
Kapoor, V.; Minson, J.; Chalmers, J.	Ventral medulla stimulation increases blood pressure and spinal cord amino acid release	1992	3	55-58	Neuroreport
Kapoor, V.; Nakahara, D.; Blood, R. J.; Chalmers, J. P.	Preferential release of neuroactive amino acids from the ventrolateral medulla of the rat in vivo as measured by microdialysis	1990	37	187-191	Neuroscience
Kashkin, V. A.; De Witte, P.	Nicotine increases microdialysate brain amino acid concentrations and induces conditioned place preference	2005	15	625-632	Eur Neuropsychopharmacol
Kasper, J. M.; Booth, R. G.; Peris, J.	Serotonin-2C receptor agonists decrease potassium-stimulated GABA release in the nucleus accumbens	2015	69	78-85	Synapse
Katayama, Y.; Kawamata, T.; Tamura, T.; Hovda, D. A.; Becker, D. P.; Tsubokawa, T.	Calcium-dependent glutamate release concomitant with massive potassium flux during cerebral ischemia in vivo	1991	558	136-140	Brain Res
Kaura, S.; Bradford, H. F.; Young, A. M.; Croucher, M. J.; Hughes, P. D.	Effect of amygdaloid kindling on the content and release of amino acids from the amygdaloid complex: in vivo and in vitro studies	1995	65	1240-1249	J Neurochem
Keck, M. E.; Sillaber, I.; Ebner, K.; Welt, T.;	Acute transcranial magnetic stimulation of frontal brain regions	2000	12	3713-	Eur J Neurosci

Aspartate					
**Authors**	**Title**	**Year**	**Vol.**	**Pages**	**Journal**
Toschi, N.; Kaehler, S. T.; Singewald, N.; Philippu, A.; Elbel, G. K.; Wotjak, C. T.; Holsboer, F.; Landgraf, R.; Engelmann, M.	selectively modulates the release of vasopressin, biogenic amines and amino acids in the rat brain			3720	
Keefe, K. A.; Sved, A. F.; Zigmond, M. J.; Abercrombie, E. D.	Stress-induced dopamine release in the neostriatum: evaluation of the role of action potentials in nigrostriatal dopamine neurons or local initiation by endogenous excitatory amino acids	1993	61	1943-1952	J Neurochem
Kehr, J.	Determination of glutamate and aspartate in microdialysis samples by reversed-phase column liquid chromatography with fluorescence and electrochemical detection	1998	708	27-38	J Chromatogr B Biomed Sci Appl
Kekesi, K. A.; Dobolyi, A.; Salfay, O.; Nyitrai, G.; Juhasz, G.	Slow wave sleep is accompanied by release of certain amino acids in the thalamus of cats	1997	8	1183-1186	Neuroreport
Kekesi, K. A.; Szilagyi, N.; Nyitrai, G.; Dobolyi, A.; Skuban, N.; Kardos, J.	Persistent depolarization and Glu uptake inhibition operate distinct osmoregulatory mechanisms in the mammalian brain	2000	37	171-178	Neurochem Int
Kendrick, K. M.; Guevara-Guzman, R.; de la Riva, C.; Christensen, J.; Ostergaard, K.; Emson, P. C.	NMDA and kainate-evoked release of nitric oxide and classical transmitters in the rat striatum: in vivo evidence that nitric oxide may play a neuroprotective role	1996	8	2619-2634	Eur J Neurosci
Kendrick, K. M.; Guevara-Guzman, R.; Zorrilla, J.; Hinton, M. R.; Broad, K. D.; Mimmack, M.; Ohkura, S.	Formation of olfactory memories mediated by nitric oxide	1997	388	670-674	Nature
Kendrick, K. M.; Hinton, M. R.	Microdialysis sampling of in vivo neurotransmitter release in the brain of the conscious sheep: Measurement of acetylcholine, amino acid and monoamine concentrations by high performance liquid chromatography (HPLC)	1992	446	66P	J Physiol
Kendrick, K. M.; Keverne, E. B.; Chapman, C.; Baldwin, B. A.	Microdialysis measurement of oxytocin, aspartate, gamma-aminobutyric acid and glutamate release from the olfactory bulb of the sheep during vaginocervical stimulation	1988	442	171-174	Brain Res
Kennedy, R. T.; Thompson, J. E.; Vickroy, T. W.	In vivo monitoring of amino acids by direct sampling of brain extracellular fluid at ultralow flow rates and capillary electrophoresis	2002	114	39-49	J Neurosci Methods
Kirschner, D. L.; Jaramillo, M.; Green, T. K.	Enantioseparation and stacking of Cyanobenz[f]isoindole-amino acids by reverse polarity capillary electrophoresis and sulfated beta-cyclodextrin	2007	79	736-743	Anal Chem
Kling, A. S.; Tachiki, K.; Lloyd, R.	Neurochemical correlates of the Kluver-Bucy syndrome by in	1993	56	161-170	Behav Brain Res

Aspartate					
**Authors**	**Title**	**Year**	**Vol.**	**Pages**	**Journal**
	vivo microdialysis in monkey				
Koetzner, L.; Hua, X. Y.; Lai, J.; Porreca, F.; Yaksh, T.	Nonopioid actions of intrathecal dynorphin evoke spinal excitatory amino acid and prostaglandin E2 release mediated by cyclooxygenase-1 and -2	2004	24	1451-1458	J Neurosci
Koizumi, H.; Fujisawa, H.; Ito, H.; Maekawa, T.; Di, X.; Bullock, R.	Effects of mild hypothermia on cerebral blood flow-independent changes in cortical extracellular levels of amino acids following contusion trauma in the rat	1997	747	304-312	Brain Res
Koizumi, H.; Fujisawa, H.; Suehiro, E.; Shirao, S.; Suzuki, M.	Neuroprotective effects of ebselen following forebrain ischemia: involvement of glutamate and nitric oxide	2011	51	337-343	Neurol Med Chir (Tokyo)
Kovacs, A.; Mihaly, A.; Komaromi, A.; Gyengesi, E.; Szente, M.; Weiczner, R.; Krisztin-Peva, B.; Szabo, G.; Telegdy, G.	Seizure, neurotransmitter release, and gene expression are closely related in the striatum of 4-aminopyridine-treated rats	2003	55	117-129	Epilepsy Res
Kretschmer, B. D.; Goiny, M.; Herrera-Marschitz, M.	Effect of intracerebral administration of NMDA and AMPA on dopamine and glutamate release in the ventral pallidum and on motor behavior	2000	74	2049-2057	J Neurochem
Kuntz, A.; Clement, H. W.; Lehnert, W.; van Calker, D.; Hennighausen, K.; Gerlach, M.; Schulz, E.	Effects of secretin on extracellular amino acid concentrations in rat hippocampus	2004	111	931-939	J Neural Transm (Vienna)
Kusaka, T.; Matsuura, S.; Fujikawa, Y.; Okubo, K.; Kawada, K.; Namba, M.; Okada, H.; Imai, T.; Isobe, K.; Itoh, S.	Relationship between cerebral interstitial levels of amino acids and phosphorylation potential during secondary energy failure in hypoxic-ischemic newborn piglets	2004	55	273-279	Pediatr Res
La Bella, V.; Piccoli, F.	Differential effect of beta-N-oxalylamino-L-alanine, the Lathyrus sativus neurotoxin, and (+/-)-alpha-amino-3-hydroxy-5-methylisoxazole-4-propionate on the excitatory amino acid and taurine levels in the brain of freely moving rats	2000	36	523-530	Neurochem Int
Lada, M. W.; Kennedy, R. T.	Quantitative in vivo monitoring of primary amines in rat caudate nucleus using microdialysis coupled by a flow-gated interface to capillary electrophoresis with laser-induced fluorescence detection	1996	68	2790-2797	Anal Chem
Lada, M. W.; Vickroy, T. W.; Kennedy, R. T.	Evidence for neuronal origin and metabotropic receptor-mediated regulation of extracellular glutamate and aspartate in rat striatum in vivo following electrical stimulation of the prefrontal cortex	1998	70	617-625	J Neurochem

Aspartate					
**Authors**	**Title**	**Year**	**Vol.**	**Pages**	**Journal**
Lada, M. W.; Vickroy, T. W.; Kennedy, R. T.	High temporal resolution monitoring of glutamate and aspartate in vivo using microdialysis on-line with capillary electrophoresis with laser-induced fluorescence detection	1997	69	4560-4565	Anal Chem
Lallement, G.; Carpentier, P.; Collet, A.; Pernot-Marino, I.; Baubichon, D.; Blanchet, G.	Effects of soman-induced seizures on different extracellular amino acid levels and on glutamate uptake in rat hippocampus	1991	563	234-240	Brain Res
Langlais, P. J.; Shu Xing, Zhang	Extracellular glutamate is increased in thalamus during thiamine deficiency-induced lesions and is blocked by MK-801	1993	61	2175-2182	J Neurochem
Largo, C.; Cuevas, P.; Herreras, O.	Is glia disfunction the initial cause of neuronal death in ischemic penumbra?	1996	18	445-448	Neurol Res
Largo, C.; Cuevas, P.; Somjen, G. G.; Martin del Rio, R.; Herreras, O.	The effect of depressing glial function in rat brain in situ on ion homeostasis, synaptic transmission, and neuron survival	1996	16	1219-1229	J Neurosci
Lasley, S. M.; Yan, Q. S.	Diminished potassium-stimulated GABA release in vivo in genetically epilepsy-prone rats	1994	175	145-148	Neurosci Lett
Lawrence, A. J.	Neurotransmitter mechanisms of rat vagal afferent neurons	1995	22	869-873	Clin Exp Pharmacol Physiol
Lawrence, A. J.; Jarrott, B.	L-glutamate as a neurotransmitter at baroreceptor afferents: evidence from in vivo microdialysis	1994	58	585-591	Neuroscience
Lawrence, A. J.; Jarrott, B.	Nitric oxide increases interstitial excitatory amino acid release in the rat dorsomedial medulla oblongata	1993	151	126-129	Neurosci Lett
Lazarewicz, J. W.; Salinska, E.; Puka, M.	Glycine enhances extracellular 45Ca2+ response to NMDA application investigated with microdialysis of rabbit hippocampus in vivo	1992	52	83-91	Acta Neurobiol Exp (Wars)
Lee, T. F.; Jantzie, L. L.; Todd, K. G.; Cheung, P. Y.	Postresuscitation N-acetylcysteine treatment reduces cerebral hydrogen peroxide in the hypoxic piglet brain	2008	34	190-197	Intensive Care Med
Lehmann, A.	Alterations in hippocampal extracellular amino acids and purine catabolites during limbic seizures induced by folate injections into the rabbit amygdala	1987	22	573-578	Neuroscience
Lehmann, A.	Effects of microdialysis-perfusion with anisoosmotic media on extracellular amino acids in the rat hippocampus and skeletal muscle	1989	53	525-535	J Neurochem
Lehmann, A.	Abnormalities in the levels of extracellular and tissue amino acids in the brain of the seizure-susceptible rat	1989	3	130-137	Epilepsy Res
Lehmann, A.	Evidence for a direct action of N-methylaspartate on non-	1987	411	95-101	Brain Res

Aspartate					
**Authors**	**Title**	**Year**	**Vol.**	**Pages**	**Journal**
	neuronal cells				
Lehmann, A.; Hagberg, H.; Huxtable, R. J.; Sandberg, M.	Reduction of brain taurine: Effects on neurotoxic and metabolic actions of kainate	1987	10	265-274	Neurochem Int
Lekieffre, D.; Callebert, J.; Plotkine, M.; Allix, M.; Boulu, R. G.	Enhancement of endogenous excitatory amino acids by theophylline does not modify the behavioral and histological consequences of forebrain ischemia	1991	565	353-357	Brain Res
Lekieffre, D.; Callebert, J.; Plotkine, M.; Boulu, R. G.	Concomitant increases in the extracellular concentrations of excitatory and inhibitory amino acids in the rat hippocampus during forebrain ischemia	1992	137	78-82	Neurosci Lett
Lekieffre, D.; Ghribi, O.; Callebert, J.; Allix, M.; Plotkine, M.; Boulu, R. G.	Inhibition of glutamate release in rat hippocampus by kynurenic acid does not protect CA1 cells from forebrain ischemia	1992	592	333-337	Brain Res
Lekieffre, D.; Meldrum, B. S.	The pyrimidine-derivative, BW1003C87, protects CA1 and striatal neurons following transient severe forebrain ischaemia in rats. A microdialysis and histological study	1993	56	93-99	Neuroscience
Lena, I.; Parrot, S.; Deschaux, O.; Muffat-Joly, S.; Sauvinet, V.; Renaud, B.; Suaud-Chagny, M. F.; Gottesmann, C.	Variations in extracellular levels of dopamine, noradrenaline, glutamate, and aspartate across the sleep--wake cycle in the medial prefrontal cortex and nucleus accumbens of freely moving rats	2005	81	891-899	J Neurosci Res
Lerma, J.; Herranz, A. S.; Herreras, O.	In vivo determination of extracellular concentration of amino acids in the rat hippocampus. A method based on brain dialysis and computerized analysis	1986	384	145-155	Brain Research
Leung, L. Y.; Tong, K. Y.; Zhang, S. M.; Zeng, X. H.; Zhang, K. P.; Zheng, X. X.	Neurochemical effects of exercise and neuromuscular electrical stimulation on brain after stroke: a microdialysis study using rat model	2006	397	135-139	Neurosci Lett
Li, H.; Li, C.; Yan, Z. Y.; Yang, J.; Chen, H.	Simultaneous monitoring multiple neurotransmitters and neuromodulators during cerebral ischemia/reperfusion in rats by microdialysis and capillary electrophoresis	2010	189	162-168	J Neurosci Methods
Li, H.; Yan, Z. Y.	Analysis of amino acid neurotransmitters in hypothalamus of rats during cerebral ischemia-reperfusion by microdialysis and capillary electrophoresis	2010	24	1185-1192	Biomed Chromatogr
Li, J.; Yang, Q.; Hu, S.	The effect of Wuto Granules on the levels of excitatory amino acids in extracellular fluid of rat hippocampus following cerebral ischemia. [Chinese]	1997	22	19-21	Hunan Yi Ke Da Xue Xue Bao

Aspartate					
**Authors**	**Title**	**Year**	**Vol.**	**Pages**	**Journal**
Li, T. F.; Xue, W.; Li, M.; Shi, A. X.; Li, K. X.	Establishment of an LC-MS/MS method to determine content of six amino acids in rat brain dialysate. [Chinese]	2015	24	1659-1664	Zhongguo xin yao za zhi
Li, Y. M.; Qu, Y.; Vandenbussche, E.; Arckens, L.; Vandesande, F.	Analysis of extracellular gamma-aminobutyric acid, glutamate and aspartate in cat visual cortex by in vivo microdialysis and capillary electrophoresis-laser induced fluorescence detection	2001	105	211-215	J Neurosci Methods
Li, Z. P.; Zhang, X. Y.; Lu, X.; Zhong, M. K.; Ji, Y. H.	Dynamic release of amino acid transmitters induced by valproate in PTZ-kindled epileptic rat hippocampus	2004	44	263-270	Neurochem Int
Lillrank, S. M.; Connor, W. T.; Oja, S. S.; Ungerstedt, U.	Systemic phencyclidine administration is associated with increased dopamine, GABA, and 5-HIAA levels in the dorsolateral striatum of conscious rats: an in vivo microdialysis study	1994	95	145-155	J Neural Transm Gen Sect
Liu, H. G.; Yang, A. C.; Meng, D. W.; Chen, N.; Zhang, J. G.	Stimulation of the anterior nucleus of the thalamus induces changes in amino acids in the hippocampi of epileptic rats	2012	1477	37-44	Brain Res
Liu, P.; He, X. R.; Zhou, W. B.; Shen, R. R.; Feng, F.	[Research for dependablity of administration of platycodi radix in Tianwang Buxinwan decoction with change of brain inhibitive neurotransmitte in rats by microdialysis]	2008	33	2830-2833	Zhongguo Zhong Yao Za Zhi
Liu, P.; He, X.; Guo, M.	[Single and combining effects of Calculus Bovis and zolpidem on inhibitive neurotransmitter of rat striatum corpora]	2010	35	904-907	Zhongguo Zhong Yao Za Zhi
Liu, Y. X.; Zhang, M.; Liu, L. Z.; Cui, X.; Hu, Y. Y.; Li, W. B.	The role of glutamate transporter-1a in the induction of brain ischemic tolerance in rats	2012	60	112-124	Glia
Liu, Z.; Zhang, L.; He, Q.; Liu, X.; Chukwunweike Ikechukwu, O.; Tong, L.; Guo, L.; Yang, H.; Zhang, Q.; Zhao, H.; Gu, X.	Effect of Baicalin-loaded PEGylated cationic solid lipid nanoparticles modified by OX26 antibody on regulating the levels of baicalin and amino acids during cerebral ischemia-reperfusion in rats	2015	489	131-138	Int J Pharmaceutics
Loidl, C. F.; Herrera-Marschitz, M.; Andersson, K.; You, Z. B.; Goiny, M.; Connor, W. T.; Silveira, R.; Rawal, R.; Bjelke, B.; Chen, Y.; et al.	Long-term effects of perinatal asphyxia on basal ganglia neurotransmitter systems studied with microdialysis in rat	1994	175	9-12	Neurosci Lett
Lombardi, G.; Moroni, F.	GM1 ganglioside reduces ischemia-induced excitatory amino acid output: a microdialysis study in the gerbil hippocampus	1992	134	171-174	Neurosci Lett
Ludvig, N.; Mishra, P. K.; Yan, Q. S.; Lasley, S. M.; Burger, R. L.; Jobe, P. C.	The paradoxical effect of NMDA receptor stimulation on electrical activity of the sensorimotor cortex in freely behaving rats: analysis by combined EEG-intracerebral microdialysis	1992	12	87-98	Synapse

Aspartate					
**Authors**	**Title**	**Year**	**Vol.**	**Pages**	**Journal**
Ludvig, N.; Potter, P. E.; Fox, S. E.	Simultaneous single-cell recording and microdialysis within the same brain site in freely behaving rats: a novel neurobiological method	1994	55	31-40	J Neurosci Methods
Luna-Munguia, H.; Meneses, A.; Pena-Ortega, F.; Gaona, A.; Rocha, L.	Effects of hippocampal high-frequency electrical stimulation in memory formation and their association with amino acid tissue content and release in normal rats	2012	22	98-105	Hippocampus
Maciejak, P.; Szyndler, J.; Turzynska, D.; Sobolewska, A.; Plaznik, A.	Kynurenic acid: A new effector of valproate action?	2011	63	1569-1573	Pharmacol Rep
MacKay, K. B.; Patel, T. R.; Galbraith, S. L.; Woodruff, G. N.; McCulloch, J.	The relationship between glutamate release and cerebral blood flow after focal cerebral ischaemia in the cat: effect of pretreatment with enadoline (a kappa receptor agonist)	1996	712	329-334	Brain Res
Malmberg, A. B.; Hamberger, A.; Hedner, T.	Effects of prostaglandin E2 and capsaicin on behavior and cerebrospinal fluid amino acid concentrations of unanesthetized rats: a microdialysis study	1995	65	2185-2193	J Neurochem
Marsala, M.; Sorkin, L. S.; Yaksh, T. L.	Transient spinal ischemia in rat: characterization of spinal cord blood flow, extracellular amino acid release, and concurrent histopathological damage	1994	14	604-614	J Cereb Blood Flow Metab
Masoero, E.; Frattini, P.; Favalli, L.; Rozza, A.; Scelsi, R.; Govoni, S.	Effect of acute alcohol on ischemia-induced glutamate release and brain damage	2000	22	173-177	Alcohol
Mason, P. A.; Escarciga, R.; Doyle, J. M.; Romano, W. F.; Berger, R. E.; Donnellan, J. P.	Amino acid concentrations in hypothalamic and caudate nuclei during microwave-induced thermal stress: analysis by microdialysis	1997	18	277-283	Bioelectromagnetics
Massieu, L.; Gomez-Roman, N.; Montiel, T.	In vivo potentiation of glutamate-mediated neuronal damage after chronic administration of the glycolysis inhibitor iodoacetate	2000	165	257-267	Exp Neurol
Matsumoto, K.; Lo, E. H.; Pierce, A. R.; Halpern, E. F.; Newcomb, R.	Secondary elevation of extracellular neurotransmitter amino acids in the reperfusion phase following focal cerebral ischemia	1996	16	114-124	J Cereb Blood Flow Metab
Matsumoto, K.; Ueda, S.; Hashimoto, T.; Kuriyama, K.	Ischemic neuronal injury in the rat hippocampus following transient forebrain ischemia: evaluation using in vivo microdialysis	1991	543	236-242	Brain Res
Mauler, F.; Fahrig, T.; Horvath, E.; Jork, R.	Inhibition of evoked glutamate release by the neuroprotective 5-HT(1A) receptor agonist BAY x 3702 in vitro and in vivo	2001	888	150-157	Brain Res
McKenzie, J. A.; Watson, C. J.; Rostand,	Automated capillary liquid chromatography for simultaneous	2002	962	105-115	J Chromatogr A

Aspartate					
**Authors**	**Title**	**Year**	**Vol.**	**Pages**	**Journal**
R. D.; German, I.; Witowski, S. R.; Kennedy, R. T.	determination of neuroactive amines and amino acids				
Meana, J. J.; Herrera-Marschitz, M.; Brodin, E.; Hokfelt, T.; Ungerstedt, U.	Simultaneous determination of cholecystokinin, dopamine, glutamate and aspartate in cortex and striatum of the rat using in vivo microdialysis	1991	1	365-373	Amino Acids
Meana, J. J.; Johansson, B.; Herrera-Marschitz, M.; Connor, W. T.; Goiny, M.; Parkinson, F. E.; Fredholm, B. B.; Ungerstedt, U.	Effect of the neurotoxin AF64A on intrinsic and extrinsic neuronal systems of rat neostriatum measured by in vivo microdialysis	1992	596	65-72	Brain Res
Melani, A.; Pantoni, L.; Bordoni, F.; Gianfriddo, M.; Bianchi, L.; Vannucchi, M. G.; Bertorelli, R.; Monopoli, A.; Pedata, F.	The selective A2A receptor antagonist SCH 58261 reduces striatal transmitter outflow, turning behavior and ischemic brain damage induced by permanent focal ischemia in the rat	2003	959	243-250	Brain Res
Melani, A.; Pantoni, L.; Corsi, C.; Bianchi, L.; Monopoli, A.; Bertorelli, R.; Pepeu, G.; Pedata, F.	Striatal outflow of adenosine, excitatory amino acids, gamma-aminobutyric acid, and taurine in awake freely moving rats after middle cerebral artery occlusion: correlations with neurological deficit and histopathological damage	1999	30	2448-2455	Stroke
Melis, M. R.; Succu, S.; Mascia, M. S.; Cortis, L.; Argiolas, A.	Extracellular excitatory amino acids increase in the paraventricular nucleus of male rats during sexual activity: main role of N-methyl-d-aspartic acid receptors in erectile function	2004	19	2569-2575	Eur J Neurosci
Mena, F. V.; Baab, P. J.; Zielke, C. L.; Huang, Y.; Zielke, H. R.	Formation of extracellular glutamate from glutamine: exclusion of pyroglutamate as an intermediate	2005	1052	88-96	Brain Res
Mena, F. V.; Baab, P. J.; Zielke, C. L.; Zielke, H. R.	In vivo glutamine hydrolysis in the formation of extracellular glutamate in the injured rat brain	2000	60	632-641	J Neurosci Res
Messam, C. A.; Greene, J. G.; Greenamyre, J. T.; Robinson, M. B.	Intrastriatal injections of the succinate dehydrogenase inhibitor, malonate, cause a rise in extracellular amino acids that is blocked by MK-801	1995	684	221-224	Brain Res
Michalak, A.; Rose, C.; Butterworth, J.; Butterworth, R. F.	Neuroactive amino acids and glutamate (NMDA) receptors in frontal cortex of rats with experimental acute liver failure	1996	24	908-913	Hepatology
Miele, M.; Mura, M. A.; Enrico, P.; Esposito, G.; Serra, P. A.; Migheli, R.; Zangani, D.; Miele, E.; Desole, M. S.	On the mechanism of d-amphetamine-induced changes in glutamate, ascorbic acid and uric acid release in the striatum of freely moving rats	2000	129	582-588	Br J Pharmacol
Mignon, L. J.; Wolf, W. A.	8-hydroxy-2-(di-n-propylamino)tetralin reduces striatal glutamate in an animal model of Parkinson's disease	2005	16	699-703	Neuroreport

Aspartate					
**Authors**	**Title**	**Year**	**Vol.**	**Pages**	**Journal**
Miguens, M.; Del Olmo, N.; Higuera-Matas, A.; Torres, I.; Garcia-Lecumberri, C.; Ambrosio, E.	Glutamate and aspartate levels in the nucleus accumbens during cocaine self-administration and extinction: a time course microdialysis study	2008	196	303-313	Psychopharmacology (Berl)
Millan, M. H.; Chapman, A. G.; Meldrum, B. S.	Dual inhibitory action of enadoline (CI977) on release of amino acids in the rat hippocampus	1995	279	75-81	Eur J Pharmacol
Millan, M. H.; Chapman, A. G.; Meldrum, B. S.	Contrasting effects of D- and L-(E)-4-(3-phosphono-2-propenyl)piperazine-2-carboxylic acid as anticonvulsants and as inhibitors of potassium-evoked increases in hippocampal extracellular glutamate and aspartate levels in freely moving rats	1994	310	217-222	J Neurochem
Millan, M. H.; Chapman, A. G.; Meldrum, B. S.	Extracellular amino acid levels in hippocampus during pilocarpine-induced seizures	1993	14	139-148	Epilepsy Res
Minamoto, Y.; Itano, T.; Tokuda, M.; Matsui, H.; Janjua, N. A.; Hosokawa, K.; Okada, Y.; Murakami, T. H.; Negi, T.; Hatase, O.	In vivo microdialysis of amino acid neurotransmitters in the hippocampus in amygdaloid kindled rat	1992	573	345-348	Brain Res
Miyashita, K.; Abe, H.; Nakajima, T.; Ishikawa, A.; Nishiura-Suzuki, M.; Naritomi, H.; Tanaka, R.; Sawada, T.	Glutamate release in the gerbil hippocampus after middle cerebral artery occlusion	1994	5	945-948	Neuroreport
Miyashita, K.; Nakajima, T.; Ishikawa, A.; Miyatake, T.	An adenosine uptake blocker, propentofylline, reduces glutamate release in gerbil hippocampus following transient forebrain ischemia	1992	17	147-150	Neurochem Res
Moghaddam, B.	Stress preferentially increases extraneuronal levels of excitatory amino acids in the prefrontal cortex: comparison to hippocampus and basal ganglia	1993	60	1650-1657	J Neurochem
Moghaddam, B.; Bunney, B. S.	Depolarization inactivation of dopamine neurons: terminal release characteristics	1993	14	195-200	Synapse
Monda, M.; Viggiano, A.; De Luca, V.	An aversive diet as thiamine-free food blocks food-induced release of excitatory amino acids in the accumbens	2003	178	197-203	Acta Physiol Scand
Monda, M.; Viggiano, A.; Sullo, A.; De Luca, V.	Aspartic and glutamic acids increase in the frontal cortex during prostaglandin E1 hyperthermia	1998	83	1239-1243	Neuroscience
Morales-Villagran, A.; Tapia, R.	Preferential stimulation of glutamate release by 4-aminopyridine in rat striatum in vivo	1996	28	35-40	Neurochem Int

Aspartate					
**Authors**	**Title**	**Year**	**Vol.**	**Pages**	**Journal**
Morita, T.; Takahashi, M.; Takeuchi, T.; Hikasa, Y.; Ikeda, S.; Sawada, M.; Sato, K.; Shibahara, T.; Shimada, A.	Changes in extracellular neurotransmitters in the cerebrum of familial idiopathic epileptic shetland sheepdogs using an intracerebral microdialysis technique and immunohistochemical study for glutamate metabolism	2005	67	1119-1126	J Vet Med Sci
Morrone, L. A.; Rombola, L.; Pelle, C.; Corasaniti, M. T.; Zappettini, S.; Paudice, P.; Bonanno, G.; Bagetta, G.	The essential oil of bergamot enhances the levels of amino acid neurotransmitters in the hippocampus of rat: implication of monoterpene hydrocarbons	2007	55	255-262	Pharmacol Res
Mura, E.; Zappettini, S.; Preda, S.; Biundo, F.; Lanni, C.; Grilli, M.; Cavallero, A.; Olivero, G.; Salamone, A.; Govoni, S.; Marchi, M.	Dual effect of beta-amyloid on alpha7 and alpha4beta2 nicotinic receptors controlling the release of glutamate, aspartate and GABA in rat hippocampus	2012	7	e29661	PLoS One
Nakane, H.; Yao, H.; Ibayashi, S.; Kitazono, T.; Ooboshi, H.; Uchimura, H.; Fujishima, M.	Protein kinase C modulates ischemia-induced amino acids release in the striatum of hypertensive rats	1998	782	290-296	Brain Res
Nakasa, H.; Tada, T.; Eguchi, T.; Hirabayashi, H.; Morimoto, T.; Sakaki, T.	Sequential changes in content of excitatory amino acids in the epileptic focus during seizure. [Japanese]	1991	43	451-454	Brain and Nerve
Nakase, H.; Tada, T.; Eguchi, T.; Hirabayashi, H.; Mirimoto, T.; Sakaki, T.	Kindling seizure induction and excitatory amino acid	1992	46	357-360	Jpn J Psychiatry Neurol
Nakase, H.; Tada, T.; Hashimoto, H.; Kurokawa, S.; Hirabayashi, H.; Hoshida, T.; Sakaki, T.; Ohnishi, H.	Experimental study of the mechanism of seizure induction: changes in the concentrations of excitatory amino acids in the epileptic focus of the cat amygdaloid kindling model	1994	34	418-422	Neurol Med Chir (Tokyo)
Nakashima, K.; Todd, M. M.	Effects of hypothermia, pentobarbital, and isoflurane on postdepolarization amino acid release during complete global cerebral ischemia	1996	85	161-168	Anesthesiology
Nakashima, K.; Todd, M. M.	Effects of hypothermia on the rate of excitatory amino acid release after ischemic depolarization	1996	27	913-918	Stroke
Nakata, N.; Kato, H.; Kogure, K.	Protective effects of serotonin reuptake inhibitors, citalopram and clomipramine, against hippocampal CA1 neuronal damage following transient ischemia in the gerbil	1992	590	48-52	Brain Res
Nash, J. F.; Yamamoto, B. K.	Methamphetamine neurotoxicity and striatal glutamate release: comparison to 3,4-methylenedioxymethamphetamine	1992	581	237-243	Brain Res
Neumann, I.; Landgraf, R.; Bauce, L.; Pittman, Q. J.	Osmotic responsiveness and cross talk involving oxytocin, but not vasopressin or amino acids, between the supraoptic nuclei	1995	15	3408-3417	J Neurosci

Aspartate					
**Authors**	**Title**	**Year**	**Vol.**	**Pages**	**Journal**
	in virgin and lactating rats				
Newcomb, R.; Palma, A.	Effects of diverse omega-conopeptides on the in vivo release of glutamic and gamma-aminobutyric acids	1994	638	95-102	Brain Res
Nicniocaill, B.; Haraldsson, B.; Hansson, O.; Connor, W. T.; Brundin, P.	Altered striatal amino acid neurotransmitter release monitored using microdialysis in R6/1 Huntington transgenic mice	2001	13	206-210	Eur J Neurosci
Nielsen, E. O.; Aarslew-Jensen, M.; Diemer, N. H.; Krogsgaard-Larsen, P.; Schousboe, A.	Baclofen-induced, calcium-dependent stimulation of in vivo release of D-[3H]aspartate from rat hippocampus monitored by intracerebral microdialysis	1989	14	321-326	Neurochem Res
Nilsson, P.; Hillered, L.; Ponten, U.; Ungerstedt, U.	Changes in cortical extracellular levels of energy-related metabolites and amino acids following concussive brain injury in rats	1990	10	631-637	J Cereb Blood Flow Metab
Nilsson, P.; Ronne-Engstrom, E.; Flink, R.; Ungerstedt, U.; Carlson, H.; Hillered, L.	Epileptic seizure activity in the acute phase following cortical impact trauma in rat	1994	637	227-232	Brain Res
Nyitrai, G.; Emri, Z.; Crunelli, V.; Kekesi, K. A.; Dobolyi, A.; Juhasz, G.	In vivo blockade of thalamic GABA(B) receptors increases excitatory amino-acid levels	1996	318	295-300	Eur J Pharmacol
Nyitrai, G.; Kekesi, K. A.; Szilagyi, N.; Papp, A.; Juhasz, G.; Kardos, J.	Neurotoxicity of lindane and picrotoxin: neurochemical and electrophysiological correlates in the rat hippocampus in vivo	2002	27	139-145	Neurochem Res
Nyitrai, G.; Kovacs, I.; Szarics, E.; Skuban, N.; Juhasz, G.; Kardos, J.	Role of intracellular Ca(2+) stores shaping normal activity in brain	1999	57	906-915	J Neurosci Res
Nyitrai, G.; Szarics, E.; Kovacs, I.; Kekesi, K. A.; Juhasz, G.; Kardos, J.	Effect of CGP 36742 on the extracellular level of neurotransmitter amino acids in the thalamus	1999	34	391-398	Neurochem Int
Obrenovitch, T. P.; Richards, D. A.	Extracellular neurotransmitter changes in cerebral ischaemia	1995	7	1-54	Cerebrovasc Brain Metab Rev
Obrenovitch, T. P.; Urenjak, J.; Richards, D. A.; Ueda, Y.; Curzon, G.; Symon, L.	Extracellular neuroactive amino acids in the rat striatum during ischaemia: comparison between penumbral conditions and ischaemia with sustained anoxic depolarisation	1993	61	178-186	J Neurochem
Ohta, H.; Li, X.; Talman, W. T.	Release of glutamate in the nucleus tractus solitarii in response to baroreflex activation in rats	1996	74	29-37	Neuroscience
Ohta, K.; Fukuuchi, Y.; Shimazu, K.; Komatsumoto, S.; Ichijo, M.; Araki, N.; Shibata, M.	Presynaptic glutamate receptors facilitate release of norepinephrine and 5-hydroxytryptamine as well as dopamine in the normal and ischemic striatum	1994	49 Suppl	S195-S202	J Auton Nerv Syst
Ooboshi, H.; Ibayashi, S.; Takano, K.; Sadoshima, S.; Kondo, A.; Uchimura, H.;	Hypothermia inhibits ischemia-induced efflux of amino acids and neuronal damage in the hippocampus of aged rats	2000	884	23-30	Brain Research

Aspartate					
**Authors**	**Title**	**Year**	**Vol.**	**Pages**	**Journal**
Fujishima, M.					
Ooboshi, H.; Sadoshima, S.; Yao, H.; Ibayashi, S.; Matsumoto, T.; Uchimura, H.; Fujishima, M.	Ischemia-induced release of amino acids in the hippocampus of aged hypertensive rats	1995	15	227-234	J Cereb Blood Flow Metab
Oreiro-Garcia, M. T.; Vazquez-Illanes, M. D.; Sierra-Paredes, G.; Sierra-Marcuno, G.	Changes in extracellular amino acid concentrations in the rat hippocampus after in vivo actin depolymerization with latrunculin A	2007	50	734-740	Neurochem Int
Oreiro-Garcia, M. T.; Vazquez-Illanes, M. D.; Sierra-Paredes, G.; Sierra-Marcuno, G.	Analysis of neuroactive amino acids from microdialysate samples by fluorescence detection using a modification of the 6-aminoquinolyl-N-hydroxysuccinimidyl carbamate method	2005	19	720-724	Biomed Chromatogr
Orwar, O.; Li, X.; Andine, P.; Bergstrom, C. M.; Hagberg, H.; Folestad, S.; Sandberg, M.	Increased intra- and extracellular concentrations of gamma-glutamylglutamate and related dipeptides in the ischemic rat striatum: involvement of glutamyl transpeptidase	1994	63	1371-1376	J Neurochem
Osborne, P. G.; Hashimoto, M.	Mammalian cerebral metabolism and amino acid neurotransmission during hibernation	2008	106	1888-1899	J Neurochem
Oskarsson, A.; Ljungberg, T.; Stahle, L.	Behavioral and neurochemical effects after combined perinatal treatment of rats with lead and disulfiram	1986	8	591-599	Neurobehav Toxicol Teratol
Ovanesov, M. V.; Vogel, M. W.; Moran, T. H.; Pletnikov, M. V.	Neonatal Borna disease virus infection in rats is associated with increased extracellular levels of glutamate and neurodegeneration in the striatum	2007	13	185-194	J Neurovirol
Palmer, A. M.; Hutson, P. H.; Lowe, S. L.; Bowen, D. M.	Extracellular concentrations of aspartate and glutamate in rat neostriatum following chemical stimulation of frontal cortex	1989	75	659-663	Exp Brain Res
Palmer, A. M.; Marion, D. W.; Botscheller, M. L.; Redd, E. E.	Therapeutic hypothermia is cytoprotective without attenuating the traumatic brain injury-induced elevations in interstitial concentrations of aspartate and glutamate	1993	10	363-372	J Neurotrauma
Palmer, A. M.; Marion, D. W.; Botscheller, M. L.; Swedlow, P. E.; Styren, S. D.; DeKosky, S. T.	Traumatic brain injury-induced excitotoxicity assessed in a controlled cortical impact model	1993	61	2015-2024	J Neurochem
Panter, S. S.; Faden, A. I.	Pretreatment with NMDA antagonists limits release of excitatory amino acids following traumatic brain injury	1992	136	165-168	Neurosci Lett
Parrot, S.; Bert, L.; Renaud, B.; Denoroy, L.	Glutamate and aspartate do not exhibit the same changes in their extracellular concentrations in the rat striatum after N-methyl-D-aspartate local administration	2003	71	445-454	J Neurosci Res

Aspartate					
**Authors**	**Title**	**Year**	**Vol.**	**Pages**	**Journal**
Parrot, S.; Bert, L.; Renaud, B.; Denoroy, L.	Large inter-experiment variations in microdialysate aspartate and glutamate in rat striatum may reflect a circannual rhythm	2001	39	267-269	Synapse
Parrot, S.; Sauvinet, V.; Riban, V.; Depaulis, A.; Renaud, B.; Denoroy, L.	High temporal resolution for in vivo monitoring of neurotransmitters in awake epileptic rats using brain microdialysis and capillary electrophoresis with laser-induced fluorescence detection	2004	140	29-38	J Neurosci Methods
Paulsen, R. E.; Fonnum, F.	Role of glial cells for the basal and Ca2+-dependent K+-evoked release of transmitter amino acids investigated by microdialysis	1989	52	1823-1829	J Neurochem
Pena, F.; Tapia, R.	Relationships among seizures, extracellular amino acid changes, and neurodegeneration induced by 4-aminopyridine in rat hippocampus: a microdialysis and electroencephalographic study	1999	72	2006-2014	J Neurochem
Perez-Pinzon, M. A.; Nilsson, G. E.; Lutz, P. L.	Relationship between ion gradients and neurotransmitter release in the newborn rat striatum during anoxia	1993	602	228-233	Brain Res
Perry, V. L.; Young, R. S.; Aquila, W. J.; During, M. J.	Effect of experimental Escherichia coli meningitis on concentrations of excitatory and inhibitory amino acids in the rabbit brain: in vivo microdialysis study	1993	34	187-191	Pediatr Res
Petkova-Kirova, P.; Rakovska, A.; Della Corte, L.; Zaekova, G.; Radomirov, R.; Mayer, A.	Neurotensin modulation of acetylcholine, GABA, and aspartate release from rat prefrontal cortex studied in vivo with microdialysis	2008	77	129-135	Brain Res Bull
Phillis, J. W.; Song, D.; Guyot, L. L.; Regan, M. H.	Lactate reduces amino acid release and fuels recovery of function in the ischemic brain	1999	272	195-198	Neurosci Lett
Pierard, C.; Lagarde, D.; Barrere, B.; Duret, P.; Cordeiro, C.; Guezennec, C. Y.; Peres, M.	Effects of a vigilance enhancing drug, modafinil, on rat brain cortex amino acids: A microdialysis study	1997	25	51-54	Med Sci Res
Pierard, C.; Peres, M.; Satabin, P.; Guezennec, C. Y.; Lagarde, D.	Effects of GABA-transaminase inhibition on brain metabolism and amino-acid compartmentation: an in vivo study by 2D 1H-NMR spectroscopy coupled with microdialysis	1999	127	321-327	Exp Brain Res
Pietraszek, M.; Golembiowska, K.; Bijak, M.; Ossowska, K.; Wolfarth, S.	Differential effects of chronic haloperidol and clozapine administration on glutamatergic transmission in the fronto-parietal cortex in rats: microdialysis and electrophysiological studies	2002	336	417-424	Naunyn Schmiedebergs Arch Pharmacol
Ping, L.; Mahesh, V. B.; Wiedmeier, V. T.;	Release of glutamate and aspartate from the preoptic area	1994	59	318-324	Neuroendocrinology

Aspartate					
**Authors**	**Title**	**Year**	**Vol.**	**Pages**	**Journal**
Brann, D. W.	during the progesterone-induced LH surge: in vivo microdialysis studies				
Puka-Sundvall, M.; Sandberg, M.; Hagberg, H.	Brain injury after hypoxia-ischemia in newborn rats: relationship to extracellular levels of excitatory amino acids and cysteine	1997	750	325-328	Brain Res
Qu, Y.; Arckens, L.; Vandenbussche, E.; Geeraerts, S.; Vandesande, F.	Simultaneous determination of total and extracellular concentrations of the amino acid neurotransmitters in cat visual cortex by microbore liquid chromatography and electrochemical detection	1998	798	19-26	J Chromatogr A
Qu, Y.; Arckens, L.; Vandesande, F.; Vandenbussche, E.	Sampling extracellular aspartate, glutamate and gamma-aminobutyric acid in striate cortex of awake cat by in vivo microdialysis: surgical and methodological aspects	2000	866	116-127	Brain Res
Qu, Y.; Li, Y.; Vandenbussche, E.; Vandesande, F.; Arckens, L.	In vivo microdialysis in the visual cortex of awake cat. II: sample analysis by microbore HPLC-electrochemical detection and capillary electrophoresis-laser-induced fluorescence detection	2001	7	45-51	Brain Res Brain Res Protoc
Quertemont, E.; Dahchour, A.; Ward, R. J.; De Witte, P.	Ethanol induces taurine release in the amygdala: An in vivo microdialysis study	1999	4	47-54	Addict Biol
Quertemont, E.; de Neuville, J.; De Witte, P.	Changes in the amygdala amino acid microdialysate after conditioning with a cue associated with ethanol	1998	139	71-78	Psychopharmacology (Berl)
Quertemont, E.; Linotte, S.; de Witte, P.	Differential taurine responsiveness to ethanol in high- and low-alcohol sensitive rats: a brain microdialysis study	2002	444	143-150	Eur J Pharmacol
Qureshi, A. I.; Ali, Z.; Suri, M. F.; Shuaib, A.; Baker, G.; Todd, K.; Guterman, L. R.; Hopkins, L. N.	Extracellular glutamate and other amino acids in experimental intracerebral hemorrhage: an in vivo microdialysis study	2003	31	1482-1489	Crit Care Med
Rabouan, S.; Olivier, J. C.; Guillemin, H.; Barthes, D.	Validation of HPLC analysis of aspartate and glutamate neurotransmitters following o-phthaldialdehyde-mercaptoethanol derivatization	2003	26	1797-1808	J Liq Chromatogr Related Technol
Radhakrishnan, R.; Sluka, K. A.	Increased glutamate and decreased glycine release in the rostral ventromedial medulla during induction of a pre-clinical model of chronic widespread muscle pain	2009	457	141-145	Neurosci Lett
Rahman, S.; Bardo, M. T.	Environmental enrichment increases amphetamine-induced glutamate neurotransmission in the nucleus accumbens: a neurochemical study	2008	1197	40-46	Brain Res
Rakovska, A.; Giovannini, M. G.; Corte, L.	Neurotensin modulation of acetylcholine and GABA release	1998	33	335-340	Neurochem Int

Aspartate					
**Authors**	**Title**	**Year**	**Vol.**	**Pages**	**Journal**
D.; Kalfin, R.; Bianchi, L.; Pepeu, G.	from the rat hippocampus: An in vivo microdialysis study				
Ramirez-Munguia, N.; Vera, G.; Tapia, R.	Epilepsy, neurodegeneration, and extracellular glutamate in the hippocampus of awake and anesthetized rats treated with okadaic acid	2003	28	1517-1524	Neurochem Res
Rao, V. L.; Audet, R. M.; Butterworth, R. F.	Selective alterations of extracellular brain amino acids in relation to function in experimental portal-systemic encephalopathy: results of an in vivo microdialysis study	1995	65	1221-1228	J Neurochem
Rea, M. A.; Ferriera, S.; Randolph, W.; Glass, J. D.	Daily profile of the extracellular concentration of glutamate in the suprachiasmatic region of the Siberian hamster	1993	204	104-109	Proc Soc Exp Biol Med
Reid, M. S.; Fox, L.; Ho, L. B.; Berger, S. P.	Nicotine stimulation of extracellular glutamate levels in the nucleus accumbens: neuropharmacological characterization	2000	35	129-136	Synapse
Reid, M. S.; Hsu Jr, K.; Berger, S. P.	Cocaine and amphetamine preferentially stimulate glutamate release in the limbic system: Studies on the involvement of dopamine	1997	27	95-105	Synapse
Renno, W. M.	Microdialysis of excitatory amino acids in the periaqueductal gray of the rat after unilateral peripheral inflammation	1998	14	319-331	Amino Acids
Renno, W. M.; Alkhalaf, M.; Mousa, A.; Kanaan, R. A.	A comparative study of excitatory and inhibitory amino acids in three different brainstem nuclei	2008	33	150-159	Neurochem Res
Renno, W. M.; Mullet, M. A.; Williams, F. G.; Beitz, A. J.	Construction of 1 mm microdialysis probe for amino acids dialysis in rats	1998	79	217-228	J Neurosci Methods
Renno, W. M.; Mullett, M. A.; Beitz, A. J.	Systemic morphine reduces GABA release in the lateral but not the medial portion of the midbrain periaqueductal gray of the rat	1992	594	221-232	Brain Res
Richards, D. A.; Lemos, T.; Whitton, P. S.; Bowery, N. G.	Extracellular GABA in the ventrolateral thalamus of rats exhibiting spontaneous absence epilepsy: a microdialysis study	1995	65	1674-1680	J Neurochem
Richards, D. A.; Morrone, L. A.; Bagetta, G.; Bowery, N. G.	Effects of alpha-dendrotoxin and dendrotoxin K on extracellular excitatory amino acids and on electroencephalograph spectral power in the hippocampus of anaesthetised rats	2000	293	183-186	Neurosci Lett
Richards, D. A.; Morrone, L. A.; Bowery, N. G.	Hippocampal extracellular amino acids and EEG spectral analysis in a genetic rat model of absence epilepsy	2000	39	2433-2441	Neuropharmacol
Ritz, M. F.; Curin, Y.; Mendelowitsch, A.; Andriantsitohaina, R.	Acute treatment with red wine polyphenols protects from ischemia-induced excitotoxicity, energy failure and oxidative stress in rats	2008	1239	226-234	Brain Res
Ritz, M. F.; Schmidt, P.; Mendelowitsch, A.	Acute effects of 17beta-estradiol on the extracellular	2004	1022	157-163	Brain Res

Aspartate					
**Authors**	**Title**	**Year**	**Vol.**	**Pages**	**Journal**
	concentration of excitatory amino acids and energy metabolites during transient cerebral ischemia in male rats				
Ritz, M. F.; Schmidt, P.; Mendelowitsch, A.	17beta-estradiol effect on the extracellular concentration of amino acids in the glutamate excitotoxicity model in the rat	2002	27	1677-1683	Neurochem Res
Robert, F.; Bert, L.; Lambas-Senas, L.; Denoroy, L.; Renaud, B.	In vivo monitoring of extracellular noradrenaline and glutamate from rat brain cortex with 2-min microdialysis sampling using capillary electrophoresis with laser-induced fluorescence detection	1996	70	153-162	J Neurosci Methods
Robinson, S. E.; Kunko, P. M.; Smith, J. A.; Wallace, M. J.; Mo, Q.; Maher, J. R.	Extracellular aspartate concentration increases in nucleus accumbens after cocaine sensitization	1997	319	31-36	Eur J Pharmacol
Rocha, L.; Briones, M.; Ackermann, R. F.; Anton, B.; Maidment, N. T.; Evans, C. J.; Engel, J., Jr.	Pentylenetetrazol-induced kindling: early involvement of excitatory and inhibitory systems	1996	26	105-113	Epilepsy Res
Rocher, C.; Bert, L.; Robert, F.; Trouvin, J. H.; Renaud, B.; Jacquot, C.; Gardier, A. M.	Microdialysis monitoring of variations in extracellular levels of serotonin, GABA and excitatory amino acids in the frontal cortex of awake rats in response to a single peripheral or central administration of dexfenfluramine	1996	737	221-230	Brain Res
Rodriguez Diaz, M.; Alonso, T. J.; Perdomo Diaz, J.; Gonzalez Hernandez, T.; Castro Fuentes, R.; Sabate, M.; Garcia Dopico, J.	Glial regulation of nonsynaptic extracellular glutamate in the substantia nigra	2005	49	134-142	Glia
Ronne Engstrom, E.; Hillered, L.; Flink, R.; Kihlstrom, L.; Lindquist, C.; Nie, J. X.; Olsson, Y.; Silander, H. C.	Extracellular amino acid levels measured with intracerebral microdialysis in the model of posttraumatic epilepsy induced by intracortical iron injection	2001	43	135-144	Epilepsy Res
Ronne-Engstrom, E.; Carlson, H.; Liu, Y.; Ungerstedt, U.; Hillered, L.	Influence of perfusate glucose concentration on dialysate lactate, pyruvate, aspartate, and glutamate levels under basal and hypoxic conditions: a microdialysis study in rat brain	1995	65	257-262	J Neurochem
Rose, M. E.; Huerbin, M. B.; Melick, J.; Marion, D. W.; Palmer, A. M.; Schiding, J. K.; Kochanek, P. M.; Graham, S. H.	Regulation of interstitial excitatory amino acid concentrations after cortical contusion injury	2002	935	40-46	Brain Res
Rozza, A.; Masoero, E.; Favalli, L.; Lanza, E.; Govoni, S.; Rizzo, V.; Montalbetti, L.	Influence of different anaesthetics on extracellular aminoacids in rat brain	2000	101	165-169	J Neurosci Methods
Saellstroem Baum, S.; Huebner, A.; Krimphove, M.; Morgenstern, R.; Badawy,	Nicotine stimulation on extracellular glutamate levels in the nucleus accumbens of ethanol-withdrawn rats in vivo	2006	30	1414-1421	Alcohol Clin Exp Res

Aspartate					
**Authors**	**Title**	**Year**	**Vol.**	**Pages**	**Journal**
A. A.; Spies, C. D.					
Saito, H.; Matsumoto, M.; Togashi, H.; Yoshioka, M.	Ischemia stimulates cortical 5-hydroxytryptamine efflux in rat brain: An in vivo microdialysis study	1994	10	91-97	Biog Amines
Salamone, A.; Mura, E.; Zappettini, S.; Grilli, M.; Olivero, G.; Preda, S.; Govoni, S.; Marchi, M.	Inhibitory effects of beta-amyloid on the nicotinic receptors which stimulate glutamate release in rat hippocampus: the glial contribution	2014	723	314-321	Eur J Pharmacol
Salazar, P.; Tapia, R.	Allopregnanolone potentiates the glutamate-mediated seizures induced by 4-aminopyridine in rat hippocampus in vivo	2012	37	596-603	Neurochem Res
Saleh, T. M.; Connell, B. J.; Legge, C.; Cribb, A. E.	Estrogen attenuates neuronal excitability in the insular cortex following middle cerebral artery occlusion	2004	1018	119-129	Brain Res
Saleh, T. M.; Connell, B. J.; McQuaid, T.; Cribb, A. E.	Estrogen-induced neurochemical and electrophysiological changes in the parabrachial nucleus of the male rat	2003	990	58-65	Brain Res
Samuel, D.; Pisano, P.; Forni, C.; Nieoullon, A.; Goff, L. K. L.	Involvement of the glutamatergic metabotropic receptors in the regulation of glutamate uptake and extracellular excitatory amino acid levels in the striatum of chloral hydrate-anesthetized rats	1996	739	156-162	Brain Res
Sanchez-Carbente, M. D. R.; Massieu, L.	Transient inhibition of glutamate uptake in vivo induces neurodegeneration when energy metabolism is impaired	1999	72	129-138	J Neurochem
Sanchez-Huerta, K. B.; Montes, S.; Perez-Severiano, F.; Alva-Sanchez, C.; Rios, C.; Pacheco-Rosado, J.	Hypothyroidism reduces glutamate-synaptic release by ouabain depolarization in rat CA3-hippocampal region	2012	90	905-912	J Neurosci Res
Sandberg, M.; Butcher, S. P.; Hagberg, H.	Extracellular overflow of neuroactive amino acids during severe insulin-induced hypoglycemia: in vivo dialysis of the rat hippocampus	1986	47	178-184	J Neurochem
Sanz, B.; Exposito, I.; Mora, F.	Effects of neurotensin on the release of glutamic acid in the prefrontal cortex and striatum of the rat	1993	4	1194-1196	NeuroReport
Schaefer, F.; Vogel, M.; Kerkhoff, G.; Woitzik, J.; Daschner, M.; Mehls, O.	Experimental uremia affects hypothalamic amino acid neurotransmitter milieu	2001	12	1218-1227	J Am Soc Nephrol
Scheller, D.; Szathmary, S.; Kolb, J.; Tegtmeier, F.	Observations on the relationship between the extracellular changes of taurine and glutamate during cortical spreading depression, during ischemia, and within the area surrounding a thrombotic infarct	2000	19	571-583	Amino Acids
Schilstrom, B.; Fagerquist, M. V.; Zhang,	Putative role of presynaptic alpha7* nicotinic receptors in	2000	38	375-383	Synapse

Aspartate					
**Authors**	**Title**	**Year**	**Vol.**	**Pages**	**Journal**
X.; Hertel, P.; Panagis, G.; Nomikos, G. G.; Svensson, T. H.	nicotine stimulated increases of extracellular levels of glutamate and aspartate in the ventral tegmental area				
Schubert, G. A.; Poli, S.; Mendelowitsch, A.; Schilling, L.; Thome, C.	Hypothermia reduces early hypoperfusion and metabolic alterations during the acute phase of massive subarachnoid hemorrhage: a laser-Doppler-flowmetry and microdialysis study in rats	2008	25	539-548	J Neurotrauma
Sciotti, V. M.; Roche, F. M.; Grabb, M. C.; Van Wylen, D. G.	Adenosine receptor blockade augments interstitial fluid levels of excitatory amino acids during cerebral ischemia	1992	12	646-655	J Cereb Blood Flow Metab
Segieth, J.; Getting, S. J.; Biggs, C. S.; Whitton, P. S.	Nitric oxide regulates excitatory amino acid release in a biphasic manner in freely moving rats	1995	200	101-104	Neurosci Lett
Seki, Y.; Feustel, P. J.; Keller Jr, R. W.; Tranmer, B. I.; Kimelberg, H. K.	Inhibition of ischemia-induced glutamate release in rat striatum by dihydrokinate and an anion channel blocker	1999	30	433-440	Stroke
Semba, J.; Miyoshi, R.; Kanazawa, M.; Kito, S.	Effect of systemic dexamethasone on extracellular amino acids, GABA and acetylcholine release in rat hippocampus using in vivo microdialysis	1995	10	17-21	Funct Neurol
Semba, J.; Wakuta, M. S.	Regional differences in the effects of glutamate uptake inhibitor L-trans-pyrrolidine-2,4-dicarboxylic acid on extracellular amino acids and dopamine in rat brain: an in vivo microdialysis study	1998	31	399-404	Gen Pharmacol
Sepulveda, M. J.; Hernandez, L.; Rada, P.; Tucci, S.; Contreras, E.	Effect of precipitated withdrawal on extracellular glutamate and aspartate in the nucleus accumbens of chronically morphine-treated rats: an in vivo microdialysis study	1998	60	255-262	Pharmacol Biochem Behav
Shah, A. J.; de Biasi, V.; Taylor, S. G.; Roberts, C.; Hemmati, P.; Munton, R.; West, A.; Routledge, C.; Camilleri, P.	Development of a protocol for the automated analysis of amino acids in brain tissue samples and microdialysates	1999	735	133-40	J Chromatogr B Biomed Sci Appl
Shea, S. D.; Smith, I. M.; McCabe, O. M.; Cronin, M. M.; Walsh, D. M.; Connor, W. T.	Intracerebroventricular Administration of Amyloid beta-protein Oligomers Selectively Increases Dorsal Hippocampal Dialysate Glutamate Levels in the Awake Rat	2008	8	7428-7437	Sensors (Basel)
Shea, T. J.; Weber, P. L.; Bammel, B. P.; Lunte, C. E.; Lunte, S. M.; Smyth, M. R.	Monitoring excitatory amino acid release in vivo by microdialysis with capillary electrophoresis-electrochemistry	1992	608	189-195	J Chromatogr
Shen, E. Y.; Lai, Y. J.	In vivo microdialysis study of excitatory and inhibitory amino acid levels in the hippocampus following penicillin-induced seizures in mature rats	2002	43	313-318	Acta Paediatr Taiwan
Sheng, W.; Hang, H. W.; Ruan, D. Y.	In vivo microdialysis study of the relationship between lead-	2005	20	233-240	Environ Toxicol Pharmacol

Aspartate					
**Authors**	**Title**	**Year**	**Vol.**	**Pages**	**Journal**
	induced impairment of learning and neurotransmitter changes in the hippocampus				
Shi, L.; Yang, A. C.; Li, J. J.; Meng, D. W.; Jiang, B.; Zhang, J. G.	Favorable modulation in neurotransmitters: effects of chronic anterior thalamic nuclei stimulation observed in epileptic monkeys	2015	265	94-101	Exp Neurol
Shibanoki, S.; Kogure, M.; Sugahara, M.; Ishikawa, K.	Effect of systemic administration of N-methyl-D-aspartic acid on extracellular taurine level measured by microdialysis in the hippocampal CA1 field and striatum of rats	1993	61	1698-1704	J Neurochem
Shimada, N.; Graf, R.; Rosner, G.; Heiss, W. D.	Ischemia-induced accumulation of extracellular amino acids in cerebral cortex, white matter, and cerebrospinal fluid	1993	60	66-71	J Neurochem
Shimizu, H.; Graham, S. H.; Chang, L. H.; Mintorovitch, J.; James, T. L.; Faden, A. I.; Weinstein, P. R.	Relationship between extracellular neurotransmitter amino acids and energy metabolism during cerebral ischemia in rats monitored by microdialysis and in vivo magnetic resonance spectroscopy	1993	605	33-42	Brain Res
Shin, R. S.; Anisman, H.; Merali, Z.; McIntyre, D. C.	Amygdala amino acid and monoamine levels in genetically Fast and Slow kindling rat strains during massed amygdala kindling: a microdialysis study	2004	20	185-194	Eur J Neurosci
Shou, M.; Smith, A. D.; Shackman, J. G.; Peris, J.; Kennedy, R. T.	In vivo monitoring of amino acids by microdialysis sampling with on-line derivatization by naphthalene-2,3-dicarboxyaldehyde and rapid micellar electrokinetic capillary chromatography	2004	138	189-197	J Neurosci Methods
Sierra-Paredes, G.; Galan-Valiente, J.; Vazquez-Illanes, M. D.; Aguilar-Veiga, E.; Sierra-Marcuno, G.	Effect of ionotropic glutamate receptors antagonists on the modifications in extracellular glutamate and aspartate levels during picrotoxin seizures: a microdialysis study in freely moving rats	2000	37	377-86	Neurochem Int
Sierra-Paredes, G.; Galan-Valiente, J.; Vazquez-Illanes, M. D.; Aguilar-Veiga, E.; Soto-Otero, R.; Mendez-Alvarez, E.; Sierra-Marcuno, G.	Extracellular amino acids in the rat hippocampus during picrotoxin threshold seizures in chronic microdialysis experiments	1998	248	53-6	Neurosci Lett
Sierra-Paredes, G.; Loureiro, A. I.; Wright, L. C.; Sierra-Marcuno, G.; Soares-da-Silva, P.	Effects of eslicarbazepine acetate on acute and chronic latrunculin A-induced seizures and extracellular amino acid levels in the mouse hippocampus	2014	15	134	BMC Neurosci
Sierra-Paredes, G.; Oreiro-Garcia, M. T.; Vazquez-Illanes, M. D.; Sierra-Marcuno, G.	Effect of eslicarbazepine acetate (BIA 2-093) on latrunculin A-induced seizures and extracellular amino acid concentrations in	2007	77	36-43	Epilepsy Res

Aspartate					
**Authors**	**Title**	**Year**	**Vol.**	**Pages**	**Journal**
	the rat hippocampus				
Silva, E.; Hernandez, L.	[Extracellular aminoacids in the amygdala and nucleus accumbens in the rat during acute pain]	2007	48	213-224	Invest Clin
Silva, E.; Hernandez, L.; Quinonez, B.; Gonzalez, L. E.; Colasante, C.	Selective amino acids changes in the medial and lateral preoptic area in the formalin test in rats	2004	124	395-404	Neuroscience
Silva, E.; Quinones, B.; Freund, N.; Gonzalez, L. E.; Hernandez, L.	Extracellular glutamate, aspartate and arginine increase in the ventral posterolateral thalamic nucleus during nociceptive stimulation	2001	923	45-49	Brain Res
Silva, E.; Quinones, B.; Paez, X.; Hernandez, L.	[Effect of a simple morphine system injection in some aminoacids in the anterior cingulate cortex during acute pain]	2008	49	511-522	Invest Clin
Silverstein, F. S.; Naik, B.	Effect of depolarization on striatal amino acid efflux in perinatal rats: an in vivo microdialysis study	1991	128	133-136	Neurosci Lett
Silverstein, F. S.; Naik, B.; Simpson, J.	Hypoxia-ischemia stimulates hippocampal glutamate efflux in perinatal rat brain: an in vivo microdialysis study	1991	30	587-590	Pediatr Res
Silverstein, F. S.; Simpson, J.; Gordon, K. E.	Hypoglycemia alters striatal amino acid efflux in perinatal rats: an in vivo microdialysis study	1990	28	516-521	Ann Neurol
Simon, R. P.; Chen, J.; Graham, S. H.	GM1 ganglioside treatment of focal ischemia: a dose-response and microdialysis study	1993	265	24-29	J Pharmacol Exp Ther
Simpson Jr, R. K.; Robertson, C. S.; Goodman, J. C.	Segmental release of amino acid neurotransmitters from transcranial stimulation	1991	16	89-94	Neurochem Res
Skorzewska, A.; Bidzinski, A.; Hamed, A.; Lehner, M.; Turzynska, D.; Sobolewska, A.; Szyndler, J.; Maciejak, P.; Wislowska-Stanek, A.; Plaznik, A.	The effect of CRF and alpha-helical CRF((9-41)) on rat fear responses and amino acids release in the central nucleus of the amygdala	2009	57	148-156	Neuropharmacol
Slaney, T. R.; Mabrouk, O. S.; Porter-Stransky, K. A.; Aragona, B. J.; Kennedy, R. T.	Chemical gradients within brain extracellular space measured using low flow push-pull perfusion sampling in vivo	2013	4	321-329	ACS Chem Neurosci
Smith, J. A.; Mo, Q.; Guo, H.; Kunko, P. M.; Robinson, S. E.	Cocaine increases extraneuronal levels of aspartate and glutamate in the nucleus accumbens	1995	683	264-269	Brain Res
Smolders, I.; Sarre, S.; Ebinger, G.; Michotte, Y.	Microbore liquid chromatography analysis of amino acid transmitters	1997	72	197-204	Methods Mol Biol
Smolders, I.; Sarre, S.; Michotte, Y.; Ebinger, G.	The analysis of excitatory, inhibitory and other amino acids in rat brain microdialysates using microbore liquid	1995	57	47-53	J Neurosci Methods

Aspartate					
**Authors**	**Title**	**Year**	**Vol.**	**Pages**	**Journal**
	chromatography				
Smolders, I.; Sarre, S.; Vanhaesendonck, C.; Ebinger, G.; Michotte, Y.	Extracellular striatal dopamine and glutamate after decortication and kainate receptor stimulation, as measured by microdialysis	1996	66	2373-2380	J Neurochem
Smolders, I.; Van Belle, K.; Ebinger, G.; Michotte, Y.	Hippocampal and cerebellar extracellular amino acids during pilocarpine-induced seizures in freely moving rats	1997	319	21-29	Eur J Pharmacol
Solas, A. B.; Kutzsche, S.; Vinje, M.; Saugstad, O. D.	Cerebral hypoxemia-ischemia and reoxygenation with 21% or 100% oxygen in newborn piglets: effects on extracellular levels of excitatory amino acids and microcirculation	2001	2	340-345	Pediatr Crit Care Med
Song, P.; Mabrouk, O. S.; Hershey, N. D.; Kennedy, R. T.	In vivo neurochemical monitoring using benzoyl chloride derivatization and liquid chromatography-mass spectrometry	2012	84	412-419	Anal Chem
Stein-Behrens, B. A.; Elliott, E. M.; Miller, C. A.; Schilling, J. W.; Newcombe, R.; Sapolsky, R. M.	Glucocorticoids exacerbate kainic acid-induced extracellular accumulation of excitatory amino acids in the rat hippocampus	1992	58	1730-1735	J Neurochem
Stiller, C. O.; Linderoth, B.; Connor, W. T.; Franck, J.; Falkenberg, T.; Ungerstedt, U.; Brodin, E.	Repeated spinal cord stimulation decreases the extracellular level of gamma-aminobutyric acid in the periaqueductal gray matter of freely moving rats	1995	699	231-241	Brain Res
Stoffel, M.; Eriskat, J.; Plesnila, M.; Aggarwal, N.; Baethmann, A.	The penumbra zone of a traumatic cortical lesion: a microdialysis study of excitatory amino acid release	1997	70	91-93	Acta Neurochir Suppl
Stoffel, M.; Plesnila, N.; Eriskat, J.; Furst, M.; Baethmann, A.	Release of excitatory amino acids in the penumbra of a focal cortical necrosis	2002	19	467-477	J Neurotrauma
Storozheva, Z. I.; Proshin, A. T.	Selective involvement of the neurotransmitter systems of the cerebellum in the mechanisms forming different types of defensive behavior	2011	41	946-972	Neurosci Behav Physiol
Storozheva, Z. I.; Proshin, A. T.	[Selective involvement of cerebellar neurotransmitter systems in mechanisms of various types of defensive behavior]	2010	60	474-485	Zh Vyssh Nerv Deiat Im I P Pavlova
Sullivan, M. E.; Hall, S. R.; Milne, B.; Jhamandas, K.	Suppression of acute and chronic opioid withdrawal by a selective soluble guanylyl cyclase inhibitor	2000	859	45-56	Brain Res
Sustkova-Fiserova, M.; Vavrova, J.; Krsiak, M.	Brain levels of GABA, glutamate and aspartate in sociable, aggressive and timid mice: an in vivo microdialysis study	2009	30	79-84	Neuro Endocrinol Lett
Sved, A. F.; Curtis, J. T.	Amino acid neurotransmitters in nucleus tractus solitarius: an in vivo microdialysis study	1993	61	2089-2098	J Neurochem
Swanson, R. A.; Chen, J.; Graham, S. H.	Glucose can fuel glutamate uptake in ischemic brain	1994	14	1-6	J Cereb Blood Flow Metab
Szeg, E. M.; Kekesi, K. A.; Szabo, Z.;	Estrogen regulates cytoskeletal flexibility, cellular metabolism	2010	35	807-819	Psychoneuroendocrinology

Aspartate					
**Authors**	**Title**	**Year**	**Vol.**	**Pages**	**Journal**
Janaky, T.; Juhasz, G. D.	and synaptic proteins: A proteomic study				
Szyndler, J.; Maciejak, P.; Turzynska, D.; Sobolewska, A.; Lehner, M.; Taracha, E.; Walkowiak, J.; Skorzewska, A.; Wislowska-Stanek, A.; Hamed, A.; Bidzinski, A.; Plaznik, A.	Changes in the concentration of amino acids in the hippocampus of pentylenetetrazole-kindled rats	2008	439	245-249	Neurosci Lett
Takeda, A.; Itoh, H.; Tamano, H.; Yuzurihara, M.; Oku, N.	Suppressive effect of Yokukansan on excessive release of glutamate and aspartate in the hippocampus of zinc-deficient rats	2008	11	41-46	Nutr Neurosci
Takeda, A.; Minami, A.; Sakurada, N.; Nakajima, S.; Oku, N.	Response of hippocampal mossy fiber zinc to excessive glutamate release	2007	50	322-327	Neurochem Int
Takeda, A.; Minami, A.; Seki, Y.; Nakajima, S.; Oku, N.	Release of amino acids by zinc in the hippocampus	2004	63	253-257	Brain Res Bull
Takeda, A.; Sakurada, N.; Kanno, S.; Ando, M.; Oku, N.	Vulnerability to seizures induced by potassium dyshomeostasis in the hippocampus in aged rats	2008	54	37-42	J Health Sci
Takeda, A.; Sotogaku, N.; Oku, N.	Influence of manganese on the release of neurotransmitters in rat striatum	2003	965	279-282	Brain Res
Takeda, A.; Sotogaku, N.; Oku, N.	Manganese influences the levels of neurotransmitters in synapses in rat brain	2002	114	669-674	Neuroscience
Takeda, A.; Tamano, H.; Kan, F.; Hanajima, T.; Yamada, K.; Oku, N.	Enhancement of social isolation-induced aggressive behavior of young mice by zinc deficiency	2008	82	909-914	Life Sci
Takeda, A.; Tamano, H.; Oku, N.	Involvement of unusual glutamate release in kainate-induced seizures in zinc-deficient adult rats	2005	66	137-143	Epilepsy Res
Tan, W. K.; Williams, C. E.; During, M. J.; Mallard, C. E.; Gunning, M. I.; Gunn, A. J.; Gluckman, P. D.	Accumulation of cytotoxins during the development of seizures and edema after hypoxic-ischemic injury in late gestation fetal sheep	1996	39	791-797	Pediatr Res
Tanaka, S.; Kiuchi, Y.; Tsuchida, A.; Yamamoto, T.; Oguchi, K.; Yoshida, T.; Kuroiwa, Y.	Possible involvement of an increase in brain extracellular concentration of excitatory amino acids in convulsions in phenobarbital-withdrawn rats	1996	17	91-102	Res Commun Alcohol S
Taylor, D. L.; Davies, S. E.; Obrenovitch, T. P.; Doheny, M. H.; Patsalos, P. N.; Clark, J. B.; Symon, L.	Investigation into the role of N-acetylaspartate in cerebral osmoregulation	1995	65	275-281	J Neurochem
Thomas, L. S.; Jane, D. E.; Gasparini, F.;	Glutamate release inhibiting properties of the novel mGlu(5)	2001	41	523-527	Neuropharmacol

Aspartate					
Authors	Title	Year	Vol.	Pages	Journal
Croucher, M. J.	receptor antagonist 2-methyl-6-(phenylethynyl)-pyridine (MPEP): complementary in vitro and in vivo evidence				
Togashi, H.; Nakamura, K.; Matsumoto, M.; Ueno, K.; Ohashi, S.; Saito, H.; Yoshioka, M.	Aniracetam enhances glutamatergic transmission in the prefrontal cortex of stroke-prone spontaneously hypertensive rats	2002	320	109-112	Neurosci Lett
Torp, R.; Arvin, B.; Le Peillet, E.; Chapman, A. G.; Ottersen, O. P.; Meldrum, B. S.	Effect of ischaemia and reperfusion on the extra- and intracellular distribution of glutamate, glutamine, aspartate and GABA in the rat hippocampus, with a note on the effect of the sodium channel blocker BW1003C87	1993	96	365-376	Exp Brain Res
Tossman, U.; Delin, A.; Eriksson, L. S.; Ungerstedt, U.	Brain cortical amino acids measured by intracerebral dialysis in portacaval shunted rats	1987	12	265-269	Neurochem Res
Tossman, U.; Eriksson, S.; Delin, A.; Hagenfeldt, L.; Law, D.; Ungerstedt, U.	Brain amino acids measured by intracerebral dialysis in portacaval shunted rats	1983	41	1046-1051	J Neurochem
Tossman, U.; Jonsson, G.; Ungerstedt, U.	Regional distribution and extracellular levels of amino acids in rat central nervous system	1986	127	533-545	Acta Physiol Scand
Tossman, U.; Segovia, J.; Ungerstedt, U.	Extracellular levels of amino acids in striatum and globus pallidus of 6-hydroxydopamine-lesioned rats measured with microdialysis	1986	127	547-551	Acta Physiol Scand
Toth, E.	Effect of nicotine on the level of extracellular amino acids in the hippocampus of rat	1996	21	903-907	Neurochem Res
Toth, E.; Harsing Jr, L. G.; Sershen, H.; Ramacci, M. T.; Lajtha, A.	Effect of acetyl-L-carnitine on extracellular amino acid levels in vivo in rat brain regions	1993	18	573-578	Neurochem Res
Toth, E.; Vizi, E. S.; Lajtha, A.	Effect of nicotine on levels of extracellular amino acids in regions of the rat brain in vivo	1993	32	827-832	Neuropharmacol
Trabace, L.; Cassano, T.; Cagiano, R.; Tattoli, M.; Pietra, C.; Steardo, L.; Kendrick, K. M.; Cuomo, V.	Effects of ENA713 and CHF2819, two anti-Alzheimer's disease drugs, on rat amino acid levels	2001	910	182-186	Brain Res
Tsai, G.; Stauch, B. L.; Vornov j, J.; Deshpande, J. K.; Coyle, J. T.	Selective release of N-acetylaspartylglutamate from rat optic nerve terminals in vivo	1990	518	313-316	Brain Research
Tseng, E. E.; Brock, M. V.; Kwon, C. C.; Annanata, M.; Lange, M. S.; Troncoso, J. C.; Johnston, M. V.; Baumgartner, W. A.	Increased intracerebral excitatory amino acids and nitric oxide after hypothermic circulatory arrest	1999	67	371-376	Ann Thorac Surg
Tseng, E. E.; Brock, M. V.; Lange, M. S.;	Monosialoganglioside GM1 inhibits neurotoxicity after	1998	124	298-306	Surgery

Aspartate					
**Authors**	**Title**	**Year**	**Vol.**	**Pages**	**Journal**
Troncoso, J. C.; Blue, M. E.; Lowenstein, C. J.; Johnston, M. V.; Baumgartner, W. A.	hypothermic circulatory arrest				
Tsuchida, E.; Harms, J. F.; Woodward, J. J.; Bullock, R.	A use-dependent sodium channel antagonist, 619C89, in reduction of ischemic brain damage and glutamate release after acute subdural hematoma in the rat	1996	85	104-111	J Neurosurg
Uchiyama-Tsuyuki, Y.; Araki, H.; Yae, T.; Otomo, S.	Changes in the extracellular concentrations of amino acids in the rat striatum during transient focal cerebral ischemia	1994	62	1074-1078	J Neurochem
Ueda, Y.; Mitsuyama, Y.	Nitroprusside inhibits kindling development and potentiates excitatory amino acid exocytosis in the kindling rat	1997	23	49-60	J Brain Sci
Vallee, N.; Rostain, J. C.; Boussuges, A.; Risso, J. J.	Comparison of nitrogen narcosis and helium pressure effects on striatal amino acids: a microdialysis study in rats	2009	34	835-844	Neurochem Res
Vallee, N.; Rostain, J. C.; Risso, J. J.	A pressurized nitrogen counterbalance to cortical glutamatergic pathway stimulation	2010	35	718-726	Neurochem Res
Vallee, N.; Rostain, J. C.; Risso, J. J.	How can an inert gas counterbalance a NMDA-induced glutamate release?	2009	107	1951-1958	J Appl Physiol (1985)
Valtonen, P.; Haapalinna, A.; Riekkinen, P., Sr.; Halonen, T.	Effect of alpha 2-adrenergic drugs dexmedetomidine and atipamezole on extracellular amino acid levels in vivo	1995	285	239-246	Eur J Pharmacol
Van Hemelrijck, A.; Sarre, S.; Smolders, I.; Michotte, Y.	Determination of amino acids associated with cerebral ischaemia in rat brain microdialysates using narrowbore liquid chromatography and fluorescence detection	2005	144	63-71	J Neurosci Methods
van Os, S.; Ruitenbeek, W.; Hopman, J.; Klaessens, J.; van de Bor, M.	Cortical excitatory amino acid release and cell function during hypotension in near-term born lambs	2006	90	128-134	Biol Neonate
Varga, V.; Kekesi, A.; Juhasz, G.; Kocsis, B.	Reduction of the extracellular level of glutamate in the median raphe nucleus associated with hippocampal theta activity in the anaesthetized rat	1998	84	49-57	Neuroscience
Vasylieva, N.; Maucler, C.; Meiller, A.; Viscogliosi, H.; Lieutaud, T.; Barbier, D.; Marinesco, S.	Immobilization method to preserve enzyme specificity in biosensors: consequences for brain glutamate detection	2013	85	2507-2515	Anal Chem
Venero, C.; Borrell, J.	Rapid glucocorticoid effects on excitatory amino acid levels in the hippocampus: a microdialysis study in freely moving rats	1999	11	2465-2473	Eur J Neurosci
Venton, B. J.; Robinson, T. E.; Kennedy, R. T.	Transient changes in nucleus accumbens amino acid concentrations correlate with individual responsivity to the predator fox odor 2,5-dihydro-2,4,5-trimethylthiazoline	2006	96	236-246	J Neurochem

**Aspartate**					
**Authors**	**Title**	**Year**	**Vol.**	**Pages**	**Journal**
Venton, B. J.; Robinson, T. E.; Kennedy, R. T.; Maren, S.	Dynamic amino acid increases in the basolateral amygdala during acquisition and expression of conditioned fear	2006	23	3391-3398	Eur J Neurosci
Vogels, B. A.; Karlsen, O. T.; Mass, M. A.; Bovee, W. M.; Chamuleau, R. A.	L-ornithine vs. L-ornithine-L-aspartate as a treatment for hyperammonemia-induced encephalopathy in rats	1997	26	174-182	J Hepatol
Vondrakova, K.; Syslova, K.; Mikoska, M.; Kacer, P.; Burchfiel, J.; Kubova, H.; Vales, K.; Tsenov, G.	The role of endothelin receptors in neurochemical changes during focal cerebral ischemia induced by intrahippocampal injection of the endothelin-1 in immature rats	2016	36	477	J Cereb Blood Flow Metab
Wade, J. V.; Olson, J. P.; Samson, F. E.; Nelson, S. R.; Pazdernik, T. L.	A possible role for taurine in osmoregulation within the brain	1988	51	740-745	J Neurochem
Wagner, Z.; Tabi, T.; Zachar, G.; Csillag, A.; Szoko, E.	Comparison of quantitative performance of three fluorescence labels in CE/LIF analysis of aspartate and glutamate in brain microdialysate	2011	32	2816-2822	Electrophoresis
Waldmeier, P. C.; Martin, P.; Stocklin, K.; Portet, C.; Schmutz, M.	Effect of carbamazepine, oxcarbazepine and lamotrigine on the increase in extracellular glutamate elicited by veratridine in rat cortex and striatum	1996	354	164-172	Naunyn Schmiedebergs Arch Pharmacol
Waldmeier, P. C.; Stocklin, K.; Feldtrauer, J. J.	Systemic administration of baclofen and the GABAB antagonist, CGP 35348, does not affect GABA, glutamate or aspartate in microdialysates of the striatum of conscious rats	1992	345	548-552	Naunyn Schmiedebergs Arch Pharmacol
Wang, L.; Huang, Y.; Wu, J.; Lv, G.; Zhou, L.; Jia, J.	Effect of Buyang Huanwu decoction on amino acid content in cerebrospinal fluid of rats during ischemic/reperfusion injury	2013	86	143-150	J Pharm Biomed Anal
Wang, M.; Hershey, N. D.; Mabrouk, O. S.; Kennedy, R. T.	Collection, storage, and electrophoretic analysis of nanoliter microdialysis samples collected from awake animals in vivo	2011	400	2013-2023	Anal Bioanal Chem
Wang, Y. L.; He, R. R.	Effects of dipfluzine on cortical somatosensory evoked potentials and amino acid contents in ischemic rat brain	1996	17	41-44	Zhongguo Yao Li Xue Bao
Ward, R. J.; Colantuoni, C.; Dahchour, A.; Quertemont, E.; De Witte, P.	Acetaldehyde-induced changes in monoamine and amino acid extracellular microdialysate content of the nucleus accumbens	1997	36	225-232	Neuropharmacology
Watson, C. J.	Insular balance of glutamatergic and GAB Aergic signaling modulates pain processing	2016	157	2194-2207	Pain
Wei, J.; Quast, M. J.	Effect of nitric oxide synthase inhibitor on a hyperglycemic rat model of reversible focal ischemia: detection of excitatory amino acids release and hydroxyl radical formation	1998	791	146-156	Brain Res
Wen, Z. H.; Wu, G. J.; Chang, Y. C.; Wang, J. J.; Wong, C. S.	Dexamethasone modulates the development of morphine tolerance and expression of glutamate transporters in rats	2005	133	807-817	Neuroscience

Aspartate					
**Authors**	**Title**	**Year**	**Vol.**	**Pages**	**Journal**
Westergren, I.; Nystrom, B.; Hamberger, A.; Johansson, B. B.	Intracerebral dialysis and the blood-brain barrier	1995	64	229-234	J Neurochem
Westergren, I.; Nystrom, B.; Hamberger, A.; Johansson, B. B.	Amino acids in extracellular fluid in vasogenic brain edema	1994	60	124-127	Acta Neurochir Suppl (Wien)
Westergren, I.; Nystrom, B.; Hamberger, A.; Nordborg, C.; Johansson, B. B.	Concentrations of amino acids in extracellular fluid after opening of the blood-brain barrier by intracarotid infusion of protamine sulfate	1994	62	159-165	J Neurochem
Westerink, B. H.; Damsma, G.; de Vries, J. B.	Effect of ouabain applied by intrastriatal microdialysis on the in vivo release of dopamine, acetylcholine, and amino acids in the brain of conscious rats	1989	52	705-712	J Neurochem
Westerink, B. H.; Hofsteede, H. M.; Damsma, G.; de Vries, J. B.	The significance of extracellular calcium for the release of dopamine, acetylcholine and amino acids in conscious rats, evaluated by brain microdialysis	1988	337	373-378	Naunyn Schmiedebergs Arch Pharmacol
Wilson, C. L.; Maidment, N. T.; Shomer, M. H.; Behnke, E. J.; Ackerson, L.; Fried, I.; Engel, J., Jr.	Comparison of seizure related amino acid release in human epileptic hippocampus versus a chronic, kainate rat model of hippocampal epilepsy	1996	26	245-254	Epilepsy Res
Wolf, M. E.; Xue, C. J.	Amphetamine and D1 dopamine receptor agonists produce biphasic effects on glutamate efflux in rat ventral tegmental area: modification by repeated amphetamine administration	1998	70	198-209	J Neurochem
Wong, J. M.; Malec, P. A.; Mabrouk, O. S.; Ro, J.; Dus, M.; Kennedy, R. T.	Benzoyl chloride derivatization with liquid chromatography-mass spectrometry for targeted metabolomics of neurochemicals in biological samples	2016	1446	78-90	J Chromatogr A
Wu, G.; Kim, H. K.; Zornow, M. H.	Transient brain ischemia in rabbits: the effect of omega-conopeptide MVIIC on hippocampal excitatory amino acids	1995	692	118-122	Brain Res
Xu, G. Y.; McAdoo, D. J.; Hughes, M. G.; Robak, G.; De Castro Jr, R.	Considerations in the determination by microdialysis of resting extracellular amino acid concentrations and release upon spinal cord injury	1998	86	1011-1021	Neuroscience
Xu, P.; Li, J.; Chen, B.; Wang, X.; Cai, X.; Jiang, H.; Wang, C.; Zhang, H.	The real-time neurotoxicity analysis of Fe3O4 nanoparticles combined with daunorubicin for rat brain in vivo	2012	8	417-423	J Biomed Nanotechnol
Xu, X. H.; Zheng, X. X.; Li, H.	Effects of puerarin on excitatory amino acid levels in cerebral ischemic striatum of rat. [Chinese]	2008	43	675-679	Zhongguo yao li xue tong bao
Xue, C. J.; Ng, J. P.; Li, Y.; Wolf, M. E.	Acute and repeated systemic amphetamine administration: effects on extracellular glutamate, aspartate, and serine levels	1996	67	352-363	J Neurochem

Aspartate					
**Authors**	**Title**	**Year**	**Vol.**	**Pages**	**Journal**
	in rat ventral tegmental area and nucleus accumbens				
Yamada, T.; Terashima, T.; Kawano, S.; Furuno, R.; Okubo, T.; Juneja, L. R.; Yokogoshi, H.	Theanine, gamma-glutamylethylamide, a unique amino acid in tea leaves, modulates neurotransmitter concentrations in the brain striatum interstitium in conscious rats	2009	36	21-27	Amino Acids
Yamada, T.; Terashima, T.; Okubo, T.; Juneja, L. R.; Yokogoshi, H.	Effects of theanine, r-glutamylethylamide, on neurotransmitter release and its relationship with glutamic acid neurotransmission	2005	8	219-226	Nutr Neurosci
Yamamoto, B. K.; Davy, S.	Dopaminergic modulation of glutamate release in striatum as measured by microdialysis	1992	58	1736-1742	J Neurochem
Yang, C. S.; Tsai, P. J.; Chen, W. Y.; Tsai, W. J.; Kuo, J. S.	On-line derivatization for continuous and automatic monitoring of brain extracellular glutamate levels in anesthetized rats: a microdialysis study	1999	734	1-6	J Chromatogr B Biomed Sci Appl
Yang, Z. Z.; Li, J.; Li, S. X.; Feng, W.; Wang, H.	Effect of ginkgolide B on striatal extracellular amino acids in middle cerebral artery occluded rats	2011	136	117-122	J Ethnopharmacol
Yao, H.; Ooboshi, H.; Ibayashi, S.; Uchimura, H.; Fujishima, M.	Cerebral blood flow and ischemia-induced neurotransmitter release in the striatum of aged spontaneously hypertensive rats	1993	24	577-580	Stroke
Yasumatsu, M.; Yazawa, T.; Otokawa, M.; Kuwasawa, K.; Hasegawa, H.; Aihara, Y.	Monoamines, amino acids and acetylcholine in the preoptic area and anterior hypothalamus of rats: measurements of tissue extracts and in vivo microdialysates	1998	121	13-23	Comp Biochem Physiol A Mol Integr Physiol
Yin, P. B.; Li, B. M.; Ye, W. L.; Mei, Z. T.	[A microdialysis study of excitatory amino acid levels of the monkeys' caudate nucleus during the delayed go/no-go task]	1997	49	128-134	Sheng Li Xue Bao
Yonezawa, Y.; Hondo, H.; Hashimoto, K.; Matsumoto, T.; Hirano, M.; Uchimura, H.; Kuroki, T.	Effect of phencyclidine on endogenous excitatory amino acid release from the rat anterior cingulate cortex--an in vivo microdialysis study	1993	94	325-340	J Neural Transm Gen Sect
You, Z. B.; Godukhin, O.; Goiny, M.; Nylander, I.; Ungerstedt, U.; Terenius, L.; Hokfelt, T.; Herrera-Marschitz, M.	Cholecystokinin-8S increases dynorphin B, aspartate and glutamate release in the fronto-parietal cortex of the rat via different receptor subtypes	1997	355	576-581	Naunyn Schmiedebergs Arch Pharmacol
You, Z. B.; Herrera-Marschitz, M.; Brodin, E.; Meana, J. J.; Morino, P.; Hokfelt, T.; Silveira, R.; Goiny, M.; Ungerstedt, U.	On the origin of striatal cholecystokinin release: studies with in vivo microdialysis	1994	62	76-85	J Neurochem
You, Z. B.; Herrera-Marschitz, M.; Nylander, I.; Goiny, M.; Kehr, J.; Ungerstedt, U.; Terenius, L.	Effect of morphine on dynorphin B and GABA release in the basal ganglia of rats	1996	710	241-248	Brain Res

Aspartate					
**Authors**	**Title**	**Year**	**Vol.**	**Pages**	**Journal**
You, Z. B.; Herrera-Marschitz, M.; Pettersson, E.; Nylander, I.; Goiny, M.; Shou, H. Z.; Kehr, J.; Godukhin, O.; Hokfelt, T.; Terenius, L.; Ungerstedt, U.	Modulation of neurotransmitter release by cholecystokinin in the neostriatum and substantia nigra of the rat: regional and receptor specificity	1996	74	793-804	Neuroscience
You, Z. B.; Herrera-Marschitz, M.; Terenius, L.	Modulation of neurotransmitter release in the basal ganglia of the rat brain by dynorphin peptides	1999	290	1307-1315	J Pharmacol Exp Ther
You, Z. B.; Pettersson, E.; Herrera-Marschitz, M.; Hokfelt, T.; Terenius, L.; Nylander, I.; Goiny, M.; Hughes, J.; Connor, W. T.; Ungerstedt, U.	Modulation of striatal aspartate and dynorphin B release by cholecystokinin (CCK-8) studied in vivo with microdialysis	1994	5	2301-2304	Neuroreport
You, Z. B.; Saria, A.; Fischer-Colbrie, R.; Terenius, L.; Goiny, M.; Herrera-Marschitz, M.	Effects of secretogranin II-derived peptides on the release of neurotransmitters monitored in the basal ganglia of the rat with in vivo microdialysis	1996	354	717-724	Naunyn Schmiedebergs Arch Pharmacol
Young, A. M. J.; Crowder, J. M.; Bradford, H. F.	Kainate potentiates excitatory amino acid release in striatum	1987	15	635-636	Biochem Soc Trans
Young, A. M.; Bradford, H. F.	N-methyl-D-aspartate releases excitatory amino acids in rat corpus striatum in vivo	1991	56	1677-1683	J Neurochem
Young, A. M.; Bradford, H. F.	Excitatory amino acid neurotransmitters in the corticostriate pathway: studies using intracerebral microdialysis in vivo	1986	47	1399-1404	J Neurochem
Young, A. M.; Foley, P. M.; Bradford, H. F.	Preloading in vivo: a rapid and reliable method for measuring gamma-aminobutyric acid and glutamate fluxes by microdialysis	1990	55	1060-1063	J Neurochem
Young, R. S.; During, M. J.; Aquila, W. J.; Tendler, D.; Ley, E.	Hypoxia increases extracellular concentrations of excitatory and inhibitory neurotransmitters in subsequently induced seizure: in vivo microdialysis study in the rabbit	1992	117	204-209	Exp Neurol
Yu, H. L.; An, Y.; Jiang, H. Y.; Jin, Q. H.; Jin, Y. Z.	Changes of amino acid concentrations in the rat medial vestibular nucleus following unilateral labyrinthectomy	2007	59	71-78	Sheng Li Xue Bao
Zachar, G.; Wagner, Z.; Tabi, T.; Balint, E.; Szoko, E.; Csillag, A.	Differential changes of extracellular aspartate and glutamate in the striatum of domestic chicken evoked by high potassium or distress: an in vivo microdialysis study	2012	37	1730-1737	Neurochem Res
Zappettini, S.; Mura, E.; Grilli, M.; Preda, S.; Salamone, A.; Olivero, G.; Govoni, S.; Marchi, M.	Different presynaptic nicotinic receptor subtypes modulate in vivo and in vitro the release of glycine in the rat hippocampus	2011	59	729-738	Neurochem Int

Aspartate					
**Authors**	**Title**	**Year**	**Vol.**	**Pages**	**Journal**
Zhang, D.; Zhang, J.; Ma, W.; Chen, D.; Han, H.; Shu, H.; Liu, G.	Analysis of trace amino acid neurotransmitters in hypothalamus of rats after exhausting exercise using microdialysis	2001	758	277-282	J Chromatogr B Biomed Sci Appl
Zhang, J.; Niu, X.	Changes of monoamines, purines and amino acids in rat striatum as measured by intercerebral microdialysis during ischemia/reperfusion	1994	9	225-229	Chin Med Sci J
Zhang, N.; Liu, P.; He, X.	[Effect of single-used borneol and combining it with diazepam on content of neurotransmitter in corpus striatum of rats]	2011	36	3180-3183	Zhongguo Zhong Yao Za Zhi
Zhang, N.; Liu, P.; He, X.	Effect of single-use versus combined-use moschus and diazepam on expression of amino acid neurotransmitters in the rat corpus striatum	2012	7	182-186	Neural Regen Res
Zhang, N.; Liu, P.; He, X.	Effect of borneol, moschus, storax, and acorus tatarinowii on expression levels of four amino acid neurotransmitters in the rat corpus striatum	2012	7	440-444	Neural Regen Res
Zhang, T.; Feng, Y.; Rockhold, R. W.; Ho, I. K.	Naloxone-precipitated morphine withdrawal increases pontine glutamate levels in the rat	1994	55	Pl25-Pl31	Life Sci
Zhang, W. Q.; Hudson, P. M.; Sobotka, T. J.; Hong, J. S.; Tilson, H. A.	Extracellular concentrations of amino acid transmitters in ventral hippocampus during and after the development of kindling	1991	540	315-318	Brain Res
Zhang, W. Q.; Rogers, B. C.; Tandon, P.; Hudson, P. M.; Sobotka, T. J.; Hong, J. S.; Tilson, H. A.	Systemic administration of kainic acid increases GABA levels in perfusate from the hippocampus of rats in vivo	1990	11	593-600	Neurotoxicology
Zhang, X. J.; Chen, H. L.; Li, Z.; Zhang, H. Q.; Xu, H. X.; Sung, J. J.; Bian, Z. X.	Analgesic effect of paeoniflorin in rats with neonatal maternal separation-induced visceral hyperalgesia is mediated through adenosine A(1) receptor by inhibiting the extracellular signal-regulated protein kinase (ERK) pathway	2009	94	88-97	Pharmacol Biochem Behav
Zhang, X.; Rauch, A.; Lee, H.; Xiao, H.; Rainer, G.; Logothetis, N. K.	Capillary hydrophilic interaction chromatography/mass spectrometry for simultaneous determination of multiple neurotransmitters in primate cerebral cortex	2007	21	3621-3628	Rapid Commun Mass Spectrom
Zhang, Y.; Zhang, H.; Feustel, P. J.; Kimelberg, H. K.	DCPIB, a specific inhibitor of volume regulated anion channels (VRACs), reduces infarct size in MCAo and the release of glutamate in the ischemic cortical penumbra	2008	210	514-520	Exp Neurol
Zhao, P.; Cheng, J.	Effects of electroacupuncture on extracellular contents of amino acid neurotransmitters in rat striatum following transient focal	1997	22	119-126	Acupunct Electrother Res

**Aspartate**					
**Authors**	**Title**	**Year**	**Vol.**	**Pages**	**Journal**
	cerebral ischemia				
Zhong, C.; Zhao, X.; Van, K. C.; Bzdega, T.; Smyth, A.; Zhou, J.; Kozikowski, A. P.; Jiang, J.; Connor, W. T.; Berman, R. F.; Neale, J. H.; Lyeth, B. G.	NAAG peptidase inhibitor increases dialysate NAAG and reduces glutamate, aspartate and GABA levels in the dorsal hippocampus following fluid percussion injury in the rat	2006	97	1015-1025	J Neurochem
Zhong, M.; Chen, H.; Jiang, Y.; Tu, P. F.; Liu, C. L.; Zhang, W. X.; Ma, J. Y.; Ding, H.	Effects of echinacoside on striatal extracellular levels of amino acid neurotransmitter in cerebral ischemia rats. [Chinese]	2012	28	361-365	Zhongguo yao li xue tong bao
Zhou, J.; Lunte, S. M.	Direct determination of amino acids by capillary electrophoresis/electrochemistry using a copper microelectrode and zwitterionic buffers	1995	16	498-503	Electrophoresis
Zhou, Q. M.; Lu, Y. L.; Hu, G.; Zeng, Y. M.; Tu, W. F.	Effect of aquaporin-4 deficiency on intravenous anaesthetic induced hypnotic effects in mice	2013	65	569-576	Sheng Li Xue Bao
Zhou, S. Y.; Zuo, H.; Stobaugh, J. F.; Lunte, C. E.; Lunte, S. M.	Continuous in vivo monitoring of amino acid neurotransmitters by microdialysis sampling with on-line derivatization and capillary electrophoresis separation	1995	67	594-599	Anal Chem
Zietlow, R.; Sinclair, S. R.; Schwiening, C. J.; Dunnett, S. B.; Fawcett, J. W.	The release of excitatory amino acids, dopamine, and potassium following transplantation of embryonic mesencephalic dopaminergic grafts to the rat striatum, and their effects on dopaminergic neuronal survival in vitro	2002	11	637-652	Cell Transplant
Zuiderwijk, M.; Veenstra, E.; Lopes da Silva, F. H.; Ghijsen, W. E.	Effects of uptake carrier blockers SK & F 89976-A and L-trans-PDC on in vivo release of amino acids in rat hippocampus	1996	307	275-282	Eur J Pharmacol

Appendix 3: Studies describing baseline dialysate concentrations for GABA

GABA					
**Authors**	**Title**	**Year**	**Vol**	**Pages**	**Journal**
Abarca, J.; Bustos, G.	Differential regulation of glutamate, aspartate and gamma-amino-butyrate release by N-methyl-D-aspartate receptors in rat striatum after partial and extensive lesions to the nigro-striatal dopamine pathway	1999	35	19-23	Neurochem Int
Abekawa, T.; Ohmori, T.; Ito, K.; Koyama, T.	D1 dopamine receptor activation reduces extracellular glutamate and GABA concentrations in the medial prefrontal cortex	2000	867	250-254	Brain Res
Abi-Saab, W. M.; Bubser, M.; Roth, R. H.; Deutch, A. Y.	5-HT2 receptor regulation of extracellular GABA levels in the prefrontal cortex	1999	20	92-96	Neuropsychopharmacology
Abraham, I.; Juhasz, G.; Kekesi, K. A.; Kovacs, K. J.	Effect of intrahippocampal dexamethasone on the levels of amino acid transmitters and neuronal excitability	1996	733	56-63	Brain Res
Acworth, I. N.; Yu, J.; Ryan, E.; Gariepy, K. C.; Gamache, P.; Hull, K.; Maher, T.	Simultaneous measurement of monoamine, amino acid, and drug levels, using high performance liquid chromatography and coulometric array technology: Application to in vivo microdialysis perfusate analysis	1994	17	685-705	J Liq Chromatogr
Ahmad, S.; Fowler, L. J.; Whitton, P. S.	Lamotrigine, carbamazepine and phenytoin differentially alter extracellular levels of 5-hydroxytryptamine, dopamine and amino acids	2005	63	141-149	Epilepsy Res
Ahmad, S.; Fowler, L. J.; Whitton, P. S.	Effects of combined lamotrigine and valproate on basal and stimulated extracellular amino acids and monoamines in the hippocampus of freely moving rats	2005	371	1-8	Naunyn Schmiedebergs Arch Pharmacol
Ahmad, S.; Fowler, L. J.; Whitton, P. S.	Effects of acute and chronic lamotrigine treatment on basal and stimulated extracellular amino acids in the hippocampus of freely moving rats	2004	1029	41-47	Brain Res
Aihara, N.; Mizukawa, K.; Koide, K.; Mabe, H.; Nishino, H.	Striatal grafts in infarct striatopallidum increase GABA release, reorganize GABAA receptor and improve water-maze learning in the rat	1994	33	483-488	Brain Res Bull
Allers, K. A.; Dremencov, E.; Ceci, A.; Flik, G.; Ferger, B.; Cremers, T. I.; Ittrich, C.; Sommer, B.	Acute and repeated flibanserin administration in female rats modulates monoamines differentially across brain areas: a microdialysis study	2010	7	1757-1767	J Sex Med
Ally, A.; Nauli, S. M.; Maher, T. J.	Cardiovascular responses and neurotransmission in the ventrolateral medulla during skeletal muscle contraction following	2002	952	176-187	Brain Res

GABA					
**Authors**	**Title**	**Year**	**Vol**	**Pages**	**Journal**
	transient middle cerebral artery occlusion and reperfusion				
Ally, A.; Phattanarudee, S.; Shafique, E.; Maher, T.	Effects of medullary administration of nitric oxide precursor on cardiovascular responses and neurotransmission during static exercise in stroke rats	2012	26	/	FASEB J. Conference: Experimental Biology
Ally, A.; Tedesco, A.; Chaitoff, K. A.	Effects of inducible nitric oxide synthase (iNOS) blockade within the periaqueductal gray on cardiovascular responses during mechanical, heat, and cold nociception	2009	23	/	FASEB J. Conference: Experimental Biology
Almaguer-Melian, W.; Cruz-Aguado, R.; Riva Cde, L.; Kendrick, K. M.; Frey, J. U.; Bergado, J.	Effect of LTP-reinforcing paradigms on neurotransmitter release in the dentate gyrus of young and aged rats	2005	327	877-883	Biochem Biophys Res Commun
Amakawa, K.; Adachi, N.; Liu, K.; Ikemune, K.; Fujitani, T.; Arai, T.	Effects of pre- and postischemic administration of thiopental on transmitter amino acid release and histologic outcome in gerbils	1996	85	1422-1430	Anesthesiology
Amantea, D.; Fratto, V.; Maida, S.; Rotiroti, D.; Ragusa, S.; Nappi, G.; Bagetta, G.; Corasaniti, M. T.	Prevention of Glutamate Accumulation and Upregulation of Phospho-Akt may Account for Neuroprotection Afforded by Bergamot Essential Oil against Brain Injury Induced by Focal Cerebral Ischemia in Rat	2009	85	389-405	Int Rev Neurobiol
Ampe, B.; Massie, A.; Haens, J.; Ebinger, G.; Michotte, Y.; Sarre, S.	NMDA-mediated release of glutamate and GABA in the subthalamic nucleus is mediated by dopamine: an in vivo microdialysis study in rats	2007	103	1063-1074	J Neurochem
Anderson, J. G.; Fordahl, S. C.; Cooney, P. T.; Weaver, T. L.; Colyer, C. L.; Erikson, K. M.	Manganese exposure alters extracellular GABA, GABA receptor and transporter protein and mRNA levels in the developing rat brain	2008	29	1044-1053	Neurotoxicology
Anderson, J. J.; DiMicco, J. A.	The use of microdialysis for studying the regional effects of pharmacological manipulation on extracellular levels of amino acids--some methodological aspects	1992	51	623-630	Life Sci
Anderson, J. J.; DiMicco, J. A.	Effect of local inhibition of gamma-aminobutyric acid uptake in the dorsomedial hypothalamus on extracellular levels of gamma-aminobutyric acid and on stress-induced tachycardia: a study using microdialysis	1990	255	1399-1407	J Pharmacol Exp Ther
Andine, P.; Orwar, O.; Jacobson, I.; Sandberg, M.; Hagberg, H.	Changes in extracellular amino acids and spontaneous neuronal activity during ischemia and extended reflow in the CA1 of the rat hippocampus	1991	57	222-229	J Neurochem
Andolina, D.; Di Segni, M.; Bisicchia, E.;	Effects of lack of microRNA-34 on the neural circuitry underlying	2016	107	305-	Neuropharmacology

GABA					
**Authors**	**Title**	**Year**	**Vol**	**Pages**	**Journal**
Alessandro, F.; Cestari, V.; Ventura, A.; Concepcion, C.; Puglisi-Allegra, S.; Ventura, R.	the stress response and anxiety			316	
Andolina, D.; Maran, D.; Viscomi, M. T.; Puglisi-Allegra, S.	Strain-dependent variations in stress coping behavior are mediated by a 5-HT/GABA interaction within the prefrontal corticolimbic system	2014	18	pyu074	Int J Neuropsycholpharmacol
Andrews, N.; Davis, B.; Gonzalez, M. I.; Oles, R.; Singh, L.; McKnight, A. T.	Effect of gastrin-releasing peptide on rat hippocampal extracellular GABA levels and seizures in the audiogenic seizure-prone DBA/2 mouse	2000	859	386-389	Brain Res
Antonelli, T.; Ferioli, V.; Gallo, G. L.; Tomasini, M. C.; Fernandez, M.; Connor, W. T.; Glennon, J. C.; Tanganelli, S.; Ferraro, L.	Differential effects of acute and short-term lithium administration on dialysate glutamate and GABA levels in the frontal cortex of the conscious rat	2000	38	355-362	Synapse
Antonelli, T.; Fuxe, K.; Agnati, L.; Mazzoni, E.; Tanganelli, S.; Tomasini, M. C.; Ferraro, L.	Experimental studies and theoretical aspects on A2A/D2 receptor interactions in a model of Parkinson's disease. Relevance for L-dopa induced dyskinesias	2006	248	16-22	J Neurol Sci
Arriaga-Avila, V.; Martinez-Abundis, E.; Cardenas-Morales, B.; Mercado-Gomez, O.; Aburto-Arciniega, E.; Miranda-Martinez, A.; Kendrick, K. M.; Guevara-Guzman, R.	Lactation reduces stress-caused dopaminergic activity and enhances GABAergic activity in the rat medial prefrontal cortex	2014	52	515-524	J Mol Neurosci
Arvin, B.; Lekieffre, D.; Graham, J. L.; Moncada, C.; Chapman, A. G.; Meldrum, B. S.	Effect of the non-NMDA receptor antagonist GYKI 52466 on the microdialysate and tissue concentrations of amino acids following transient forebrain ischaemia	1994	62	1458-1467	J Neurochem
Arvin, B.; Moncada, C.; Le Peillet, E.; Chapman, A.; Meldrum, B. S.	GYKI 52466 blocks the increase in extracellular glutamate induced by ischaemia	1992	3	235-238	Neuroreport
Bagley, E. E.; Hacker, J.; Chefer, V. I.; Mallet, C.; McNally, G. P.; Chieng, B. C.; Perroud, J.; Shippenberg, T. S.; Christie, M. J.	Drug-induced GABA transporter currents enhance GABA release to induce opioid withdrawal behaviors	2011	14	1548-1554	Nat Neurosci
Bakkelund, A. H.; Fonnum, F.; Paulsen, R. E.	Evidence using in vivo microdialysis that aminotransferase activities are important in the regulation of the pools of transmitter amino acids	1993	18	411-415	Neurochem Res

GABA					
**Authors**	**Title**	**Year**	**Vol**	**Pages**	**Journal**
Baldwin, H. A.; Williams, J. L.; Snares, M.; Ferreira, T.; Cross, A. J.; Green, A. R.	Attenuation by chlormethiazole administration of the rise in extracellular amino acids following focal ischaemia in the cerebral cortex of the rat	1994	112	188-194	Br J Pharmacol
Ballini, C.; Corte, L. D.; Pazzagli, M.; Colivicchi, M. A.; Pepeu, G.; Tipton, K. F.; Giovannini, M. G.	Extracellular levels of brain aspartate, glutamate and GABA during an inhibitory avoidance response in the rat	2008	106	1035-1043	J Neurochem
Bankson, M. G.; Yamamoto, B. K.	Serotonin-GABA interactions modulate MDMA-induced mesolimbic dopamine release	2004	91	852-859	J Neurochem
Baroncelli, L.; Sale, A.; Viegi, A.; Maya Vetencourt, J. F.; De Pasquale, R.; Baldini, S.; Maffei, L.	Experience-dependent reactivation of ocular dominance plasticity in the adult visual cortex	2010	226	100-109	Exp Neurol
Basaran, N. F.; Buyukuysal, R. L.; Sertac Yilmaz, M.; Aydin, S.; Cavun, S.; Millington, W. R.	The effect of Gly-Gln [ss-endorphin30-31] on morphine-evoked serotonin and GABA efflux in the nucleus accumbens of conscious rats	2016	58	23-29	Neuropeptides
Battaglia, G.; Bruno, V.; Pisani, A.; Centonze, D.; Catania, M. V.; Calabresi, P.; Nicoletti, F.	Selective blockade of type-1 metabotropic glutamate receptors induces neuroprotection by enhancing gabaergic transmission	2001	17	1071-1083	Mol Cell Neurosci
Beal, M. F.; Brouillet, E.; Jenkins, B. G.; Ferrante, R. J.; Kowall, N. W.; Miller, J. M.; Storey, E.; Srivastava, R.; Rosen, B. R.; Hyman, B. T.	Neurochemical and histologic characterization of striatal excitotoxic lesions produced by the mitochondrial toxin 3-nitropropionic acid	1993	13	4181-4192	J Neurosci
Bedse, G.; Romano, A.; Tempesta, B.; Lavecchia, M. A.; Pace, L.; Bellomo, A.; Duranti, A.; Micioni Di Bonaventura, M. V.; Cifani, C.; Cassano, T.; Gaetani, S.	Inhibition of anandamide hydrolysis enhances noradrenergic and GABAergic transmission in the prefrontal cortex and basolateral amygdala of rats subjected to acute swim stress	2015	93	777-787	J Neurosci Res
Beggiato, S.; Antonelli, T.; Tomasini, M. C.; Tanganelli, S.; Fuxe, K.; Schwarcz, R.; Ferraro, L.	Kynurenic acid, by targeting alpha7 nicotinic acetylcholine receptors, modulates extracellular GABA levels in the rat striatum in vivo	2013	37	1470-1477	Eur J Neurosci
Beggiato, S.; Connor, W. T.; Tomasini, M. C.; Antonelli, T.; Loche, A.; Tanganelli, S.; Cacciaglia, R.; Ferraro, L.	GET73 increases rat extracellular hippocampal CA1 GABA levels through a possible involvement of local mGlu5 receptor	2013	67	678-691	Synapse
Beggiato, S.; Tanganelli, S.; Fuxe, K.;	Endogenous kynurenic acid regulates extracellular GABA levels in	2014	82	11-18	Neuropharmacology

GABA					
**Authors**	**Title**	**Year**	**Vol**	**Pages**	**Journal**
Antonelli, T.; Schwarcz, R.; Ferraro, L.	the rat prefrontal cortex				
Beggiato, S.; Tomasini, M. C.; Borelli, A. C.; Borroto-Escuela, D. O.; Fuxe, K.; Antonelli, T.; Tanganelli, S.; Ferraro, L.	Functional role of striatal A2A, D2, and mGlu5 receptor interactions in regulating striatopallidal GABA neuronal transmission	2016	138	254-264	J Neurochem
Benturquia, N.; Parrot, S.; Sauvinet, V.; Renaud, B.; Denoroy, L.	Simultaneous determination of vigabatrin and amino acid neurotransmitters in brain microdialysates by capillary electrophoresis with laser-induced fluorescence detection	2004	806	237-244	J Chromatogr B Analyt Technol Biomed Life Sci
Bergquist, F.; Ludwig, M.; Dutia, M. B.	Neuroscience I: Commissural GABA release during vestibular compensation in the rat	2009	37	S28	Amino Acids
Bergquist, F.; Ludwig, M.; Dutia, M. B.	Role of the commissural inhibitory system in vestibular compensation in the rat	2008	586	4441-4452	J Physiol
Bergquist, J.; Vona, M. J.; Stiller, C. O.; Connor, W. T.; Falkenberg, T.; Ekman, R.	Capillary electrophoresis with laser-induced fluorescence detection: a sensitive method for monitoring extracellular concentrations of amino acids in the periaqueductal grey matter	1996	65	33-42	J Neurosci Methods
Bernay, B.; Gaillard, M. C.; Guryca, V.; Emadali, A.; Kuhn, L.; Bertrand, A.; Detraz, I.; Carcenac, C.; Savasta, M.; Brouillet, E.; Garin, J.; Elalouf, J. M.	Discovering new bioactive neuropeptides in the striatum secretome using in vivo microdialysis and versatile proteomics	2009	8	946-958	Mol Cell Proteomics
Beverly, J. L.; Beverly, M. F.; Meguid, M. M.	Alterations in extracellular GABA in the ventral hypothalamus of rats in response to acute glucoprivation	1995	269	R1174-R1178	Am J Physiol
Beverly, J. L.; de Vries, M. G.; Beverly, M. F.; Arseneau, L. M.	Norepinephrine mediates glucoprivic-induced increase in GABA in the ventromedial hypothalamus of rats	2000	279	R990-R996	Am J Physiol Regul Integr Comp Physiol
Beverly, J. L.; De Vries, M. G.; Bouman, S. D.; Arseneau, L. M.	Noradrenergic and GABAergic systems in the medial hypothalamus are activated during hypoglycemia	2001	280	R563-R569	Am J Physiol Regul Integr Comp Physiol
Bianchi, L.; Ballini, C.; Colivicchi, M. A.; Della Corte, L.; Giovannini, M. G.; Pepeu, G.	Investigation on acetylcholine, aspartate, glutamate and GABA extracellular levels from ventral hippocampus during repeated exploratory activity in the rat	2003	28	565-573	Neurochem Res
Bianchi, L.; Colivicchi, M. A.; Bolam, J. P.; Della Corte, L.	The release of amino acids from rat neostriatum and substantia nigra in vivo: a dual microdialysis probe analysis	1998	87	171-180	Neuroscience
Bianchi, L.; Della Corte, L.; Tipton, K. F.	Simultaneous determination of basal and evoked output levels of aspartate, glutamate, taurine and 4-aminobutyric acid during microdialysis and from superfused brain slices	1999	723	47-59	J Chromatogr B Biomed Sci Appl
Bianchi, L.; Sharp, T.; Bolam, J. P.;	The effect of kainic acid on the release of GABA in rat neostriatum	1994	5	1233-	Neuroreport

GABA					
**Authors**	**Title**	**Year**	**Vol**	**Pages**	**Journal**
Della Corte, L.	and substantia nigra			1236	
Bido, S.; Marti, M.; Morari, M.	Amantadine attenuates levodopa-induced dyskinesia in mice and rats preventing the accompanying rise in nigral GABA levels	2011	118	1043-1055	J Neurochem
Bie, X.; Chen, Y.; Han, J.; Dai, H.; Wan, H.; Zhao, T.	Effects of gastrodin on amino acids after cerebral ischemia-reperfusion injury in rat striatum	2007	16 Suppl 1	305-308	Asia Pac J Clin Nutr
Biggs, C. S.; Fowler, L. J.; Whitton, P. S.; Starr, M. S.	Impulse-dependent and tetrodotoxin-sensitive release of GABA in the rat's substantia nigra measured by microdialysis	1995	684	172-178	Brain Res
Biggs, C. S.; Pearce, B. R.; Fowler, L. J.; Whitton, P. S.	Effect of isonicotinic acid hydrazide on extracellular amino acids and convulsions in the rat: Reversal of neurochemical and behavioural deficits by sodium valproate	1994	63	2197-2201	J Neurochem
Biggs, C. S.; Pearce, B. R.; Fowler, L. J.; Whitton, P. S.	The effect of sodium valproate on extracellular GABA and other amino acids in the rat ventral hippocampus: an in vivo microdialysis study	1992	594	138-142	Brain Res
Blanco, L.; Lorigados, L.; Martinez, L.; Pavon, N.; Gonzalez, M. E.; Serrano, T.; Blanco, V.	Neuroprotective effects of MK-801 systemic administration on the pedunculopontine nucleus of hemiparkinsonian rats. [Spanish, English]	2009	26	53-64	Biotecnologia Aplicada
Bledsoe Jr, S. C.; Nagase, S.; Miller, J. M.; Altschuler, R. A.	Deafness-induced plasticity in the mature central auditory system	1995	7	225-229	NeuroReport
Bogaert, L.; Scheller, D.; Moonen, J.; Sarre, S.; Smolders, I.; Ebinger, G.; Michotte, Y.	Neurochemical changes and laser Doppler flowmetry in the endothelin-1 rat model for focal cerebral ischemia	2000	887	266-275	Brain Res
Bolea, I.; Colivicchi, M. A.; Ballini, C.; Fattori, M.; Marco-Contelles, J. L.; Unzeta, M.; Della Corte, L.	Protective effect of the MAO-B inhibitor PF9601N in a microdialysis model of excitotoxicity	2011	8	/	Neurodegener Dis
Bolea, I.; Colivicchi, M. A.; Ballini, C.; Marco-Contelles, J.; Tipton, K. F.; Unzeta, M.; Della Corte, L.	Neuroprotective effects of the MAO-B inhibitor, PF9601N, in an in vivo model of excitotoxicity	2014	20	641-650	CNS Neurosci Ther
Bortel, A.; Nowak, P.; Brus, R.	Neonatal DSP-4 treatment modifies GABAergic neurotransmission in the prefrontal cortex of adult rats	2008	13	247-252	Neurotox Res
Bosch, O. J.; Sartori, S. B.; Singewald, N.; Neumann, I. D.	Extracellular amino acid levels in the paraventricular nucleus and the central amygdala in high- and low-anxiety dams rats during maternal aggression: regulation by oxytocin	2007	10	261-270	Stress
Bosman, D. K.; Deutz, N. E.; Maas, M.	Amino acid release from cerebral cortex in experimental acute liver	1992	59	591-	J Neurochem

GABA					
**Authors**	**Title**	**Year**	**Vol**	**Pages**	**Journal**
A.; van Eijk, H. M.; Smit, J. J.; de Haan, J. G.; Chamuleau, R. A.	failure, studied by in vivo cerebral cortex microdialysis			599	
Bouchez, G.; Millan, M. J.; Rivet, J. M.; Billiras, R.; Boulanger, R.; Gobert, A.	Quantification of extracellular levels of corticosterone in the basolateral amygdaloid complex of freely-moving rats: a dialysis study of circadian variation and stress-induced modulation	2012	1452	47-60	Brain Res
Boulet, S.; Lacombe, E.; Carcenac, C.; Feuerstein, C.; Sgambato-Faure, V.; Poupard, A.; Savasta, M.	Subthalamic stimulation-induced forelimb dyskinesias are linked to an increase in glutamate levels in the substantia nigra pars reticulata	2006	26	10768-10776	J Neurosci
Boulet, S.; Mounayar, S.; Poupard, A.; Bertrand, A.; Jan, C.; Pessiglione, M.; Hirsch, E. C.; Feuerstein, C.; Francois, C.; Feger, J.; Savasta, M.; Tremblay, L.	Behavioral recovery in MPTP-treated monkeys: neurochemical mechanisms studied by intrastriatal microdialysis	2008	28	9575-9584	J Neurosci
Bourdelais, A. J.; Deutch, A. Y.	The effects of haloperidol and clozapine on extracellular GABA levels in the prefrontal cortex of the rat: an in vivo microdialysis study	1994	4	69-77	Cereb Cortex
Bourdelais, A. J.; Kalivas, P. W.	Modulation of extracellular gamma-aminobutyric acid in the ventral pallidum using in vivo microdialysis	1992	58	2311-2320	J Neurochem
Bourdelais, A.; Kalivas, P. W.	High sensitivity HPLC assay for GABA in brain dialysis studies	1991	39	115-121	J Neurosci Methods
Bourdelais, A.; Kalivas, P. W.	Apomorphine decreases extracellular GABA in the ventral pallidum of rats with 6-OHDA lesions in the nucleus accumbens	1992	577	306-311	Brain Res
Bourdelais, A.; Kalivas, P. W.	Amphetamine lowers extracellular GABA concentration in the ventral pallidum	1990	516	132-136	Brain Res
Bourne, J. A.; Fosbraey, P.; Halliday, J.	Changes in striatal electroencephalography and neurochemistry induced by kainic acid seizures are modified by dopamine receptor antagonists	2001	413	189-198	Eur J Pharmacol
Bowser, M. T.; Kennedy, R. T.	In vivo monitoring of amine neurotransmitters using microdialysis with on-line capillary electrophoresis	2001	22	3668-3676	Electrophoresis
Boyd, B. W.; Witowski, S. R.; Kennedy, R. T.	Trace-level amino acid analysis by capillary liquid chromatography and application to in vivo microdialysis sampling with 10-s temporal resolution	2000	72	865-871	Anal Chem
Bragina, L.; Marchionni, I.; Omrani, A.; Cozzi, A.; Pellegrini-Giampietro, D. E.;	GAT-1 regulates both tonic and phasic GABA(A) receptor-mediated inhibition in the cerebral cortex	2008	105	1781-1793	J Neurochem

GABA					
**Authors**	**Title**	**Year**	**Vol**	**Pages**	**Journal**
Cherubini, E.; Conti, F.					
Bredewold, R.; Schiavo, J. K.; van der Hart, M.; Verreij, M.; Veenema, A. H.	Dynamic changes in extracellular release of GABA and glutamate in the lateral septum during social play behavior in juvenile rats: Implications for sex-specific regulation of social play behavior	2015	307	117-127	Neuroscience
Brennan, P. A.; Kendrick, K. M.; Keverne, E. B.	Neurotransmitter release in the accessory olfactory bulb during and after the formation of an olfactory memory in mice	1995	69	1075-1086	Neuroscience
Britton, P.; Whitton, P. S.; Fowler, L. J.; Bowery, N. G.	Tetanus toxin-induced effects on extracellular amino acid levels in rat hippocampus: an in vivo microdialysis study	1996	67	324-349	J Neurochem
Brodie, M. E.; Opacka-Juffry, J.; Peterson, D. W.; Brown, A. W.	Neurochemical changes in hippocampal and caudate dialysates associated with early trimethyltin neurotoxicity in rats	1990	11	35-46	Neurotoxicology
Bruening, S.; Oh, E.; Hetzenauer, A.; Escobar-Alvarez, S.; Westphalen, R. I.; Hemmings, H. C., Jr.; Singewald, N.; Shippenberg, T.; Toth, M.	The anxiety-like phenotype of 5-HT receptor null mice is associated with genetic background-specific perturbations in the prefrontal cortex GABA-glutamate system	2006	99	892-899	J Neurochem
Bruet, N.; Windels, F.; Carcenac, C.; Feuerstein, C.; Bertrand, A.; Poupard, A.; Savasta, M.	Neurochemical mechanisms induced by high frequency stimulation of the subthalamic nucleus: increase of extracellular striatal glutamate and GABA in normal and hemiparkinsonian rats	2003	62	1228-1240	J Neuropathol Exo Neurol
Brugnoli, A.; Napolitano, F.; Usiello, A.; Morari, M.	Genetic deletion of Rhes or pharmacological blockade of mTORC1 prevent striato-nigral neurons activation in levodopa-induced dyskinesia	2016	85	155-163	Neurobiol Dis
Bruhn, T.; Cobo, M.; Berg, M.; Diemer, N. H.	Limbic seizure-induced changes in extracellular amino acid levels in the hippocampal formation: a microdialysis study of freely moving rats	1992	86	455-461	Acta Neurol Scand
Bubser, M.; de Brabander, J. M.; Timmerman, W.; Feenstra, M. G.; Erdtsieck-Ernste, E. B.; Rinkens, A.; van Uum, J. F.; Westerink, B. H.	Disinhibition of the mediodorsal thalamus induces fos-like immunoreactivity in both pyramidal and GABA-containing neurons in the medial prefrontal cortex of rats, but does not affect prefrontal extracellular GABA levels	1998	30	156-165	Synapse
Buck, K.; Voehringer, P.; Ferger, B.	Rapid analysis of GABA and glutamate in microdialysis samples using high performance liquid chromatography and tandem mass spectrometry	2009	182	78-84	J Neurosci Methods
Bustamante, D.; You, Z. B.; Castel, M. N.; Johansson, S.; Goiny, M.; Terenius, L.; Hokfelt, T.; Herrera-Marschitz, M.	Effect of single and repeated methamphetamine treatment on neurotransmitter release in substantia nigra and neostriatum of the rat	2002	83	645-654	J Neurochem

GABA					
Authors	Title	Year	Vol	Pages	Journal
Butcher, S. P.; Bullock, R.; Graham, D. I.; McCulloch, J.	Correlation between amino acid release and neuropathologic outcome in rat brain following middle cerebral artery occlusion	1990	21	1727-1733	Stroke
Butcher, S. P.; Cameron, D.; Kendall, L.; Griffiths, R.	Homocysteine-induced alterations in extracellular amino acids in rat hippocampus	1992	20	75-80	Neurochem Int
Butcher, S. P.; Hamberger, A.	In vivo studies on the extracellular, and veratrine-releasable, pools of endogenous amino acids in the rat striatum: effects of corticostriatal deafferentation and kainic acid lesion	1987	48	713-721	J Neurochem
Butcher, S. P.; Lazarewicz, J. W.; Hamberger, A.	In vivo microdialysis studies on the effects of decortication and excitotoxic lesions on kainic acid-induced calcium fluxes, and endogenous amino acid release, in the rat striatum	1987	49	1355-1360	J Neurochem
Butcher, S. P.; Sandberg, M.; Hagberg, H.; Hamberger, A.	Cellular origins of endogenous amino acids released into the extracellular fluid of the rat striatum during severe insulin-induced hypoglycemia	1987	48	722-728	J Neurochem
Byrnes, E. M.; Reilly, A.; Bruno, J. P.	Effects of AMPA and D1 receptor activation on striatal and nigral GABA efflux	1997	26	254-268	Synapse
Caille, S.; Parsons, L. H.	Cannabinoid modulation of opiate reinforcement through the ventral striatopallidal pathway	2006	31	804-813	Neuropsychopharmacology
Caille, S.; Parsons, L. H.	Intravenous heroin self-administration decreases GABA efflux in the ventral pallidum: an in vivo microdialysis study in rats	2004	20	593-596	Eur J Neurosci
Calatrava-Ferreras, L.; Gonzalo-Gobernado, R.; Reimers, D.; Herranz, A. S.; Jimenez-Escrig, A.; Diaz-Gil, J. J.; Casarejos, M. J.; Montero-Vega, M. T.; Bazan, E.	Neuroprotective role of liver growth factor "LGF" in an experimental model of cerebellar ataxia	2014	15	19056-19073	Int J Mol Sci
Calcagno, E.; Invernizzi, R. W.	Strain-dependent serotonin neuron feedback control: role of serotonin 2C receptors	2010	114	1701-1710	J Neurochem
Campbell, K.; Kalen, P.; Lundberg, C.; Wictorin, K.; Rosengren, E.; Bjorklund, A.	Extracellular gamma-aminobutyric acid levels in the rat caudate-putamen: monitoring the neuronal and glial contribution by intracerebral microdialysis	1993	614	241-250	Brain Res
Campbell, K.; Kalen, P.; Wictorin, K.; Lundberg, C.; Mandel, R. J.; Bjorklund, A.	Characterization of GABA release from intrastriatal striatal transplants: dependence on host-derived afferents	1993	53	403-415	Neuroscience
Campos, F.; Alfonso, M.; Duran, R.	In vivo modulation of alpha7 nicotinic receptors on striatal	2010	56	850-	Neurochem Int

GABA					
Authors	Title	Year	Vol	Pages	Journal
	glutamate release induced by anatoxin-A			855	
Cassel, G. E.; Fosbraey, P.	Measurement of the oxime HI-6 after peripheral administration in tandem with neurotransmitter levels in striatal dialysates: effects of soman intoxication	1996	35	159-166	J Pharmacol Toxicol Methods
Castelli, M. P.; Ferraro, L.; Mocci, I.; Carta, F.; Carai, M. A.; Antonelli, T.; Tanganelli, S.; Cignarella, G.; Gessa, G. L.	Selective gamma-hydroxybutyric acid receptor ligands increase extracellular glutamate in the hippocampus, but fail to activate G protein and to produce the sedative/hypnotic effect of gamma-hydroxybutyric acid	2003	87	722-732	J Neurochem
Castro-Alamancos, M. A.; Torres-Aleman, I.	Long-term depression of glutamate-induced gamma-aminobutyric acid release in cerebellum by insulin-like growth factor I	1993	90	7386-7390	Proc Natl Acad Sci U S A
Cauli, O.; Agusti, A.; Felipo, V.	Hyperammonemia increases gabaergic tone in cerebellum but decreases it in cortex of rats. Role in cognitive impairment	2010	52	S205-S206	J Hepatol
Cauli, O.; Llansola, M.; Erceg, S.; Felipo, V.	Hypolocomotion in rats with chronic liver failure is due to increased glutamate and activation of metabotropic glutamate receptors in substantia nigra	2006	45	654-661	J Hepatol
Cauli, O.; Mlili, N.; Llansola, M.; Felipo, V.	Motor activity is modulated via different neuronal circuits in rats with chronic liver failure than in normal rats	2007	25	2112-2222	Eur J Neurosci
Cauli, O.; Mlili, N.; Rodrigo, R.; Felipo, V.	Hyperammonaemia alters the mechanisms by which metabotropic glutamate receptors in nucleus accumbens modulate motor function	2007	103	38-46	J Neurochem
Chaitoff, K. A.; Patel, D.; Ally, A.	Effects of endothelial NOS antagonism within the periaqueductal gray on cardiovascular responses and neurotransmission during mechanical, heat, and cold nociception	2008	1236	93-104	Brain Res
Chan, O.; Cheng, H.; Herzog, R.; Czyzyk, D.; Zhu, W.; Wang, A.; McCrimmon, R. J.; Seashore, M. R.; Sherwin, R. S.	Increased GABAergic tone in the ventromedial hypothalamus contributes to suppression of counterregulatory responses after antecedent hypoglycemia	2008	57	1363-1370	Diabetes
Chan, O.; Lawson, M.; Zhu, W.; Beverly, J. L.; Sherwin, R. S.	ATP-sensitive K(+) channels regulate the release of GABA in the ventromedial hypothalamus during hypoglycemia	2007	56	1120-1126	Diabetes
Chan, O.; Paranjape, S. A.; Horblitt, A.; Zhu, W.; Sherwin, R. S.	Lactate-induced release of GABA in the ventromedial hypothalamus contributes to counterregulatory failure in recurrent hypoglycemia and diabetes	2013	62	4239-4246	Diabetes
Chan, O.; Paranjape, S.; Czyzyk, D.;	Increased GABAergic output in the ventromedial hypothalamus	2011	60	1582-	Diabetes

GABA					
**Authors**	**Title**	**Year**	**Vol**	**Pages**	**Journal**
Horblitt, A.; Zhu, W.; Ding, Y.; Fan, X.; Seashore, M.; Sherwin, R.	contributes to impaired hypoglycemic counterregulation in diabetic rats			1589	
Chapman, M. A.; See, R. E.	The neurotensin receptor antagonist SR 48692 decreases extracellular striatal GABA in rats	1996	729	124-126	Brain Res
Chapman, M. A.; See, R. E.	Differential effects of unique profile antipsychotic drugs on extracellular amino acids in the ventral pallidum and globus pallidus of rats	1996	277	1586-1594	J Pharmacol Exp Ther
Chefer, V. I.; Denoroy, L.; Zapata, A.; Shippenberg, T. S.	Mu opioid receptor modulation of somatodendritic dopamine overflow: GABAergic and glutamatergic mechanisms	2009	30	272-278	Eur J Neurosci
Chefer, V. I.; Wang, R.; Shippenberg, T. S.	Basolateral amygdala-driven augmentation of medial prefrontal cortex GABAergic neurotransmission in response to environmental stimuli associated with cocaine administration	2011	36	2018-2029	Neuropsychopharmacology
Chefer, V.; Meis, J.; Wang, G.; Kuzmin, A.; Bakalkin, G.; Shippenberg, T.	Repeated exposure to moderate doses of ethanol augments hippocampal glutamate neurotransmission by increasing release	2011	16	229-237	Addict Biol
Chen, N.; Meng, F. G.; Zhang, J. G.; Yang, A. C.; Liu, H. G.; Hu, W. H.; Meng, D. W.; Zhang, X.; Liu, C.; Ge, Y.	[Changes of glutamate and gamma-aminobutyric acid of extracellular fluid in hippocampus during electrical stimulation of anterior nucleus thalamus in rats]	2012	92	3371-3373	Zhonghua Yi Xue Za Zhi
Chen, R.; Deng, Y.; Yao, J.; Kamal, G. M.; Wang, J.; Xu, F.	Assessment of amino acid neurotransmitters in rat brain microdialysis samples by high-performance liquid chromatography with coulometric detection	2015	38	1439-1447	J Liq Chromatogr Related Tech
Cho, C.	Striatal glutamate and GABA after high frequency subthalamic stimulation in parkinsonian rat	2015	15	1313	Neurodegener Dis
Choi, Y. H.; Chang, N.; Fletcher, P. J.; Anderson, G. H.	Dietary protein content affects the profiles of extracellular amino acids in the medial preoptic area of freely moving rats	2000	66	1105-1118	Life Sci
Choi, Y. H.; Fletcher, P. J.; Anderson, G. H.	Extracellular amino acid profiles in the paraventricular nucleus of the rat hypothalamus are influenced by diet composition	2001	892	320-328	Brain Res
Choi, Y. H.; Fletcher, P. J.; Wong, C. C.; Anderson, G. H.	Measurement of blood-brain barrier permeability of rats with alpha-aminoisobutyric acid during microdialysis: possible application to behavioral studies	1999	67	587-598	Physiol Behav
Ciriacks, C. M.; Bowser, M. T.	Measuring the effect of glutamate receptor agonists on extracellular D-serine concentrations in the rat striatum using online microdialysis-capillary electrophoresis	2006	393	200-205	Neurosci Lett
Ciriacks, C. M.; Bowser, M. T.	Monitoring D-serine dynamics in the rat brain using online	2004	76	6582-	Anal Chem

GABA					
Authors	Title	Year	Vol	Pages	Journal
	microdialysis-capillary electrophoresis			6587	
Clement, H. W.; Pschibul, A.; Schulz, E.	Effects of secretin on extracellular GABA and other amino acid concentrations in the rat hippocampus	2005	71	239-271	Int Rev Neurobiol
Cleton, A.; Altorf, B. A.; Voskuyl, R. A.; Danhof, M.	Effect of amygdala kindling on the central nervous system effects of tiagabine: EEG effects versus brain GABA levels	2000	130	1037-1044	Br J Pharmacol
Clinckers, R.; Zgavc, T.; Vermoesen, K.; Meurs, A.; Michotte, Y.; Smolders, I.	Pharmacological and neurochemical characterization of the involvement of hippocampal adrenoreceptor subtypes in the modulation of acute limbic seizures	2010	115	1595-1607	J Neurochem
Cobo, M.; Bruhn, T.; Berg, M.; Diemer, N. H.	Phenylsuccinate reduces KCL-induced release of GABA evidence for the participation of the ketodicarboxylate carrier in the biosynthesis of transmitter-GABA	1993	5	377-388	Amino Acids
Collins-Praino, L. E.; Podurgiel, S. J.; Kovner, R.; Randall, P. A.; Salamone, J. D.	Extracellular GABA in globus pallidus increases during the induction of oral tremor by haloperidol but not by muscarinic receptor stimulation	2012	234	129-135	Behav Brain Res
Connor, W. T.	Functional neuroanatomy of the ventral striopallidal GABA pathway. New sites of intervention in the treatment of schizophrenia	2001	109	31-39	J Neurosci Methods
Connor, W. T.	Functional neuroanatomy of the basal ganglia as studied by dual-probe microdialysis	1998	25	743-746	Nucl Med Biol
Connor, W. T.; Lindefors, N.; Brene, S.; Herrera-Marschitz, M.; Persson, H.; Ungerstedt, U.	Short-term dopaminergic regulation of GABA release in dopamine deafferented caudate-putamen is not directly associated with glutamic acid decarboxylase gene expression	1991	128	66-70	Neurosci Lett
Connor, W. T.; Osborne, P. G.; Ungerstedt, U.	Tolerance to catalepsy following chronic haloperidol is not associated with changes in GABA release in the globus pallidus	1998	787	299-303	Brain Res
Connor, W. T.; Tanganelli, S.; Ungerstedt, U.; Fuxe, K.	The effects of neurotensin on GABA and acetylcholine release in the dorsal striatum of the rat: an in vivo microdialysis study	1992	573	209-216	Brain Res
Cook, C. J.	Stress induces CRF release in the paraventricular nucleus, and both CRF and GABA release in the amygdala	2000	82	751-762	Physiol Behav
Cook, C. J.	Monitoring on-line of extracellular gamma-amino-4-butyric acid using microdialysis coupled to immunosensor analysis	1998	82	145-150	J Neurosci Methods
Cook, C. J.; Maasland, S. A.; Devine, C. E.	Social behaviour in sheep relates to behaviour and neurotransmitter responses to nociceptive stimuli	1996	60	741-751	Physiol Behav
Corsi, C.; Melani, A.; Bianchi, L.; Pepeu,	Effect of adenosine A2A receptor stimulation on GABA release	1999	10	3933-	Neuroreport

GABA					
**Authors**	**Title**	**Year**	**Vol**	**Pages**	**Journal**
G.; Pedata, F.	from the striatum of young and aged rats in vivo			3937	
Corsi, C.; Pazzagli, M.; Bianchi, L.; Della Corte, L.; Pepeu, G.; Pedata, F.	In vivo amino acid release from the striatum of aging rats: adenosine modulation	1997	18	243-250	Neurobiol Aging
Cowen, M.; Chen, F.; Jarrott, B.; Lawrence, A. J.	Effects of acute ethanol on GABA release and GABA(A) receptor density in the rat mesolimbic system	1998	59	51-57	Pharmacol Biochem Behav
Da Costa, A. P.; De La Riva, C.; Guevara-Guzman, R.; Kendrick, K. M.	C-fos and c-jun in the paraventricular nucleus play a role in regulating peptide gene expression, oxytocin and glutamate release, and maternal behaviour	1999	11	2199-2210	Eur J Neurosci
Dahchour, A.; De Witte, P.	Effect of repeated ethanol withdrawal on glutamate microdialysate in the hippocampus	1999	23	1698-1703	Alcohol Clin Exp Res
Dahchour, A.; De Witte, P.	Taurine blocks the glutamate increase in the nucleus accumbens microdialysate of ethanol-dependent rats	2000	65	345-350	Pharmacol Biochem Behav
Dahchour, A.; De Witte, P.	Acamprosate decreases the hypermotility during repeated ethanol withdrawal	1999	18	77-81	Alcohol
Dahchour, A.; Hoffman, A.; Deitrich, R.; de Witte, P.	Effects of ethanol on extracellular amino acid levels in high-and low-alcohol sensitive rats: a microdialysis study	2000	35	548-553	Alcohol Alcohol
Dahchour, A.; Quertemont, E.; De Witte, P.	Taurine increases in the nucleus accumbens microdialysate after acute ethanol administration to naive and chronically alcoholised rats	1996	735	9-19	Brain Res
Dalby, N. O.	GABA-level increasing and anticonvulsant effects of three different GABA uptake inhibitors	2000	39	2399-2407	Neuropharmacology
Dave, K. R.; Lange-Asschenfeldt, C.; Raval, A. P.; Prado, R.; Busto, R.; Saul, I.; Perez-Pinzon, M. A.	Ischemic preconditioning ameliorates excitotoxicity by shifting glutamate/gamma-aminobutyric acid release and biosynthesis	2055	82	665-673	J Neurosci Res
Dawson, L. A.; Organ, A. J.; Winter, P.; Lacroix, L. P.; Shilliam, C. S.; Heidbreder, C.; Shah, A. J.	Rapid high-throughput assay for the measurement of amino acids from microdialysates and brain tissue using monolithic C18-bonded reversed-phase columns	2004	807	235-241	J Chromatogr B Analyt Technol Biomed Life Sci
De Bundel, D.; Aourz, N.; Kiagiadaki, F.; Clinckers, R.; Hoyer, D.; Kastellakis, A.; Michotte, Y.; Thermos, K.; Smolders, I.	Hippocampal sst(1) receptors are autoreceptors and do not affect seizures in rats	2010	21	254-258	Neuroreport
de Groote, L.; Linthorst, A. C.	Exposure to novelty and forced swimming evoke stressor-dependent changes in extracellular GABA in the rat hippocampus	2007	148	794-805	Neuroscience
De Novellis, V.; Marabese, I.; Palazzo,	Group I metabotropic glutamate receptors modulate glutamate and	2003	462	73-81	Eur J Pharmacol

GABA					
**Authors**	**Title**	**Year**	**Vol**	**Pages**	**Journal**
E.; Berrino, L.; Rodella, L.; Bianchi, R.; Rossi, F.; Maione, S.	gamma-aminobutyric acid release in the periaqueductal grey of rats				
de Novellis, V.; Negri, L.; Lattanzi, R.; Rossi, F.; Palazzo, E.; Marabese, I.; Giannini, E.; Vita, D.; Melchiorri, P.; Maione, S.	The prokineticin receptor agonist Bv8 increases GABA release in the periaqueductal grey and modifies RVM cell activities and thermoceptive reflexes in the rat	2007	26	3068-3078	Eur J Neurosci
Del Arco, A.; Gonzalez-Mora, J. L.; Armas, V. R.; Mora, F.	Amphetamine increases the extracellular concentration of glutamate in striatum of the awake rat: involvement of high affinity transporter mechanisms	1999	38	943-954	Neuropharmacology
Del Arco, A.; Mora, F.	NMDA and AMPA/kainate glutamatergic agonists increase the extracellular concentrations of GABA in the prefrontal cortex of the freely moving rat: modulation by endogenous dopamine	2002	57	623-630	Brain Res Bull
Del Arco, A.; Mora, F.	Endogenous dopamine potentiates the effects of glutamate on extracellular GABA in the prefrontal cortex of the freely moving rat	2000	53	339-345	Brain Res Bull
Del Arco, A.; Mora, F.	Effects of endogenous glutamate on extracellular concentrations of GABA, dopamine, and dopamine metabolites in the prefrontal cortex of the freely moving rat: involvement of NMDA and AMPA/KA receptors	1999	24	1027-1035	Neurochem Res
Dell, L. E.; Parsons, L. H.	Serotonin1B receptors in the ventral tegmental area modulate cocaine-induced increases in nucleus accumbens dopamine levels	2004	311	711-719	J Pharmacol Exp Ther
Devall, A. J.; Blake, R.; Langman, N.; Smith, C. G.; Richards, D. A.; Whitehead, K. J.	Monolithic column-based reversed-phase liquid chromatography separation for amino acid assay in microdialysates and cerebral spinal fluid	2007	848	323-328	J Chromatogr B Technol Biomed Life Sci
Di Cara, B.; Samuel, D.; Salin, P.; Kerkerian-Le Goff, L.; Daszuta, A.	Serotonergic regulation of the GABAergic transmission in the rat basal ganglia	2003	50	144-150	Synapse
Diaz-Cabiale, Z.; Vivo, M.; Del Arco, A.; Connor, W. T.; Harte, M. K.; Muller, C. E.; Martinez, E.; Popoli, P.; Fuxe, K.; Ferre, S.	Metabotropic glutamate mGlu5 receptor-mediated modulation of the ventral striopallidal GABA pathway in rats. Interactions with adenosine A(2A) and dopamine D(2) receptors	2002	324	154-158	Neurosci Lett
Ding, R.; Asada, H.; Obata, K.	Changes in extracellular glutamate and GABA levels in the hippocampal CA3 and CA1 areas and the induction of glutamic acid decarboxylase-67 in dentate granule cells of rats treated with kainic acid	1998	800	105-113	Brain Res

GABA					
**Authors**	**Title**	**Year**	**Vol**	**Pages**	**Journal**
Dodge, J. C.; Illig, A. M.; Snyder, P. J.; Badura, L. L.	GABA levels within the medial preoptic area: effects of chronic administration of sodium valproic acid	2000	25	519-534	Psychoneuroendocrinology
Dohmen, C.; Kumura, E.; Rosner, G.; Heiss, W. D.; Graf, R.	Adenosine in relation to calcium homeostasis: comparison between gray and white matter ischemia	2001	21	503-510	J Cereb Blood Flow Metab
Dong, H. I.; Fukuda, S.; Murata, E.; Higuchi, T.	Excitatory and inhibitory actions of isoflurane on the cholinergic ascending arousal system of the rat	2006	104	122-132	Anesthesiology
Donnell, J. C.; McDonough, J. H.; Shih, T. M.	In vivo microdialysis and electroencephalographic activity in freely moving guinea pigs exposed to organophosphorus nerve agents sarin and VX: analysis of acetylcholine and glutamate	2011	85	1607-1616	Arch Toxicol
Donzanti, B. A.; Yamamoto, B. K.	An improved and rapid HPLC-EC method for the isocratic separation of amino acid neurotransmitters from brain tissue and microdialysis perfusates	1988	43	913-922	Life Sci
Doretto, M. C.; Burger, R. L.; Mishra, P. K.; Garcia-Cairasco, N.; Dailey, J. W.; Jobe, P. C.	A microdialysis study of amino acid concentrations in the extracellular fluid of the substantia nigra of freely behaving GEPR-9s: relationship to seizure predisposition	1994	17	157-165	Epilepsy Res
Drew, K. L.; Connor, W. T.; Kehr, J.; Ungerstedt, U.	Regional specific effects of clozapine and haloperidol on GABA and dopamine release in rat basal ganglia	1990	187	385-397	Eur J Pharmacol
Drew, K. L.; Connor, W. T.; Kehr, J.; Ungerstedt, U.	Characterization of gamma-aminobutyric acid and dopamine overflow following acute implantation of a microdialysis probe	1989	45	1307-1317	Life Sci
Drew, K. L.; Fitka, T.; Hu, Y.; Ungerstedt, U.	Non-exocytotic GABA overflow in rat striatum inhibits gnawing	1997	61	1593-1601	Life Sci
Drew, K. L.; Ungerstedt, U.	Pergolide presynaptically inhibits calcium-stimulated release of gamma-aminobutyric acid	1991	57	1927-1930	J Neurochem
During, M. J.; Leone, P.; Davis, K. E.; Kerr, D.; Sherwin, R. S.	Glucose modulates rat substantia nigra GABA release in vivo via ATP-sensitive potassium channels	1995	95	2403-2408	J Clin Invest
During, M. J.; Ryder, K. M.; Spencer, D. D.	Hippocampal GABA transporter function in temporal-lobe epilepsy	1995	376	174-177	Nature
Dzahini, K.; Dentresangle, C.; Le Cavorsin, M.; Bertrand, A.; Detraz, I.; Savasta, M.; Leviel, V.	Pre-synaptic glutamate-induced activation of DA release in the striatum after partial nigral lesion	2010	113	1459-1470	J Neurochem
Ebner, K.; Bosch, O. J.; Kromer, S. A.; Singewald, N.; Neumann, I. D.	Release of oxytocin in the rat central amygdala modulates stress-coping behavior and the release of excitatory amino acids	2005	30	223-230	Neuropsychopharmacology
Ehlen, J. C.; Albers, H. E.; Breyer, E. D.	MEKC-LIF of gamma-amino butyric acid in microdialysate:	2005	147	36-47	J Neurosci Methods

GABA					
**Authors**	**Title**	**Year**	**Vol**	**Pages**	**Journal**
	Systematic optimization of the separation conditions by factorial analysis				
Engel, M.; Snikeris, P.; Jenner, A.; Karl, T.; Huang, X. F.; Frank, E.	Neuregulin 1 prevents phencyclidine-induced behavioral impairments and disruptions to GABAergic signaling in mice	2015	18	1-11	Int J Neuropsychop
Engelmann, M.; Bull, P. M.; Brown, C. H.; Landgraf, R.; Horn, T. F.; Singewald, N.; Ludwig, M.; Wotjak, C. T.	GABA selectively controls the secretory activity of oxytocin neurons in the rat supraoptic nucleus	2004	19	601-608	Eur J Neurosci
Engelmann, M.; Ludwig, M.; Singewald, N.; Ebner, K.; Sabatier, N.; Lubec, G.; Landgraf, R.; Wotjak, C. T.	Taurine selectively modulates the secretory activity of vasopressin neurons in conscious rats	2001	14	1047-1055	Eur J Neurosci
Engelmann, M.; Wolf, G.; Horn, T. F.	Release patterns of excitatory and inhibitory amino acids within the hypothalamic supraoptic nucleus in response to direct nitric oxide administration during forced swimming in rats	2002	324	252-254	Neurosci Lett
Exposito, I.; Del Arco, A.; Segovia, G.; Mora, F.	Endogenous dopamine increases extracellular concentrations of glutamate and GABA in striatum of the freely moving rat: involvement of D1 and D2 dopamine receptors	1999	24	849-856	Neurochem
Fabre-Nys, C.; Blache, D.; Hinton, M. R.; Goode, J. A.; Kendrick, K. M.	Microdialysis measurement of neurochemical changes in the mediobasal hypothalamus of ovariectomized ewes during oestrus	1994	649	282-296	Brain Res
Faiman, M. D.; Kaul, S.; Latif, S. A.; Williams, T. D.; Lunte, C. E.	S-(N, N-diethylcarbamoyl)glutathione (carbamathione), a disulfiram metabolite and its effect on nucleus accumbens and prefrontal cortex dopamine, GABA, and glutamate: a microdialysis study	2013	75	95-105	Neuropharmacology
Falkenberg, T.; Lindefors, N.; Connor, W. T.; Zachrisson, O.; Camilli, F.; Ungerstedt, U.	GABA release and GAD67 mRNA expression in rat hippocampus following entorhinal cortex activation	1997	48	413-416	Brain Res Mol Brain Res
Fallgren, A. B.; Paulsen, R. E.	A microdialysis study in rat brain of dihydrokainate, a glutamate uptake inhibitor	1996	21	19-25	Neurochem Res
Fantin, M.; Auberson, Y. P.; Morari, M.	Differential effect of NR2A and NR2B subunit selective NMDA receptor antagonists on striato-pallidal neurons: relationship to motor response in the 6-hydroxydopamine model of parkinsonism	2008	106	957-968	J Neurochem
Fantin, M.; Marti, M.; Auberson, Y. P.; Morari, M.	NR2A and NR2B subunit containing NMDA receptors differentially regulate striatal output pathways	2007	103	2200-2211	J Neurochem
Farahmandfar, M.; Zarrindast, M. R.; Kadivar, M.; Karimian, S. M.; Naghdi, N.	The effect of morphine sensitization on extracellular concentrations of GABA in dorsal hippocampus of male rats	2011	669	66-70	Eur J Pharmacol

GABA					
**Authors**	**Title**	**Year**	**Vol**	**Pages**	**Journal**
Fedele, E.; Varnier, G.; Ansaldo, M. A.; Raiteri, M.	Nicotine administration stimulates the in vivo N-methyl-D-aspartate receptor/nitric oxide/cyclic GMP pathway in rat hippocampus through glutamate release	1998	125	1042-1048	Br J Pharmacol
Fenelon, V. S.; Herbison, A. E.	Progesterone regulation of GABAA receptor plasticity in adult rat supraoptic nucleus	2000	12	1617-1623	Eur J Neurosci
Ferraro, L.; Antonelli, T.; Connor, W. T.; Fuxe, K.; Soubrie, P.; Tanganelli, S.	The striatal neurotensin receptor modulates striatal and pallidal glutamate and GABA release: functional evidence for a pallidal glutamate-GABA interaction via the pallidal-subthalamic nucleus loop	1998	18	6977-6989	J Neurosci
Ferraro, L.; Antonelli, T.; Connor, W. T.; Tanganelli, S.; Rambert, F. A.; Fuxe, K.	The effects of modafinil on striatal, pallidal and nigral GABA and glutamate release in the conscious rat: evidence for a preferential inhibition of striato-pallidal GABA transmission	1998	253	135-138	Neurosci Lett
Ferraro, L.; Antonelli, T.; Connor, W. T.; Tanganelli, S.; Rambert, F. A.; Fuxe, K.	Modafinil: an antinarcoleptic drug with a different neurochemical profile to d-amphetamine and dopamine uptake blockers	1997	42	1181-1183	Biol Psychiatry
Ferraro, L.; Antonelli, T.; Connor, W. T.; Tanganelli, S.; Rambert, F.; Fuxe, K.	The antinarcoleptic drug modafinil increases glutamate release in thalamic areas and hippocampus	1997	8	2883-2887	Neuroreport
Ferraro, L.; Antonelli, T.; Tanganelli, S.; Connor, W. T.; Perez de la Mora, M.; Mendez-Franco, J.; Rambert, F. A.; Fuxe, K.	The vigilance promoting drug modafinil increases extracellular glutamate levels in the medial preoptic area and the posterior hypothalamus of the conscious rat: prevention by local GABAA receptor blockade	1999	20	346-356	Neuropsychopharmacology
Ferraro, L.; Connor, W. T.; Antonelli, T.; Fuxe, K.; Tanganelli, S.	Differential effects of intrastriatal neurotensin(1-13) and neurotensin(8-13) on striatal dopamine and pallidal GABA release. A dual-probe microdialysis study in the awake rat	1997	9	1838-1846	Eur J Neurosci
Ferraro, L.; Connor, W. T.; Beggiato, S.; Tomasini, M. C.; Fuxe, K.; Tanganelli, S.; Antonelli, T.	Striatal NTS1 , dopamine D2 and NMDA receptor regulation of pallidal GABA and glutamate release--a dual-probe microdialysis study in the intranigral 6-hydroxydopamine unilaterally lesioned rat	2012	35	207-220	Eur J Neurosci
Ferraro, L.; Connor, W. T.; Glennon, J.; Tomasini, M. C.; Bebe, B. W.; Tanganelli, S.; Antonelli, T.	Evidence for a nucleus accumbens CCK2 receptor regulation of rat ventral pallidal GABA levels: a dual probe microdialysis study	2000	68	483-496	Life Sci
Ferraro, L.; Connor, W. T.; Li, X. M.; Rimondini, R.; Beani, L.; Ungerstedt, U.; Fuxe, K.; Tanganelli, S.	Evidence for a differential cholecystokinin-B and -A receptor regulation of GABA release in the rat nucleus accumbens mediated via dopaminergic and cholinergic mechanisms	1996	73	941-950	Neuroscience
Ferraro, L.; Tanganelli, S.; Connor, W.	The vigilance promoting drug modafinil decreases GABA release	1996	220	5-8	Neurosci Lett

GABA					
**Authors**	**Title**	**Year**	**Vol**	**Pages**	**Journal**
T.; Antonelli, T.; Rambert, F.; Fuxe, K.	in the medial preoptic area and in the posterior hypothalamus of the awake rat: possible involvement of the serotonergic 5-HT3 receptor				
Ferraro, L.; Tanganelli, S.; Connor, W. T.; Francesconi, W.; Loche, A.; Gessa, G. L.; Antonelli, T.	gamma-Hydroxybutyrate modulation of glutamate levels in the hippocampus: an in vivo and in vitro study	2001	78	929-939	J Neurochem
Ferraro, L.; Tomasini, M. C.; Cassano, T.; Bebe, B. W.; Siniscalchi, A.; Connor, W. T.; Magee, P.; Tanganelli, S.; Cuomo, V.; Antonelli, T.	Cannabinoid receptor agonist WIN 55,212-2 inhibits rat cortical dialysate gamma-aminobutyric acid levels	2001	66	298-302	J Neurosci Res
Ferraro, L.; Tomasini, M. C.; Fernandez, M.; Bebe, B. W.; Connor, W. T.; Fuxe, K.; Glennon, J. C.; Tanganelli, S.; Antonelli, T.	Nigral neurotensin receptor regulation of nigral glutamate and nigroventral thalamic GABA transmission: a dual-probe microdialysis study in intact conscious rat brain	2001	102	113-120	Neuroscience
Ferre, S.; Connor, W. T.; Fuxe, K.; Ungerstedt, U.	The striopallidal neuron: a main locus for adenosine-dopamine interactions in the brain	1993	13	5402-5406	J Neurosci
Ferre, S.; Oconnor, W. T.; Snaprud, P.; Ungerstedt, U.; Fuxe, K.	Antagonistic interaction between adenosine A(2a) receptors and dopamine D2 receptors in the ventral striopallidal system. Implications for the treatment of schizophrenia	1994	63	765-773	Neuroscience
Fink-Jensen, A.; Suzdak, P. D.; Swedberg, M. D.; Judge, M. E.; Hansen, L.; Nielsen, P. G.	The gamma-aminobutyric acid (GABA) uptake inhibitor, tiagabine, increases extracellular brain levels of GABA in awake rats	1992	220	197-201	Eur J Pharmacol
Fordahl, S. C.; Anderson, J. G.; Cooney, P. T.; Weaver, T. L.; Colyer, C. L.; Erikson, K. M.	Manganese exposure inhibits the clearance of extracellular GABA and influences taurine homeostasis in the striatum of developing rats	2010	31	639-646	Neurotoxicology
Forray, M. I.; Bustos, G.; Gysling, K.	Noradrenaline inhibits glutamate release in the rat bed nucleus of the stria terminalis: in vivo microdialysis studies	1999	55	311-320	J Neurosci Res
Frantz, K.; Harte, M.; Ungerstedt, U.; Connor, W. T.	A dual probe characterization of dialysate amino acid levels in the medial prefrontal cortex and ventral tegmental area of the awake freely moving rat	2002	119	109-119	J Neurosci Methods
Fraser, M.; Bennet, L.; Van Zijl, P. L.; Mocatta, T. J.; Williams, C. E.; Gluckman, P. D.; Winterbourn, C. C.;	Extracellular amino acids and lipid peroxidation products in periventricular white matter during and after cerebral ischemia in preterm fetal sheep	2008	105	2214-2223	J Neurochem

GABA					
**Authors**	**Title**	**Year**	**Vol**	**Pages**	**Journal**
Gunn, A. J.					
Freinbichler, W.; Colivicchi, M. A.; Fattori, M.; Ballini, C.; Tipton, K. F.; Linert, W.; Della Corte, L.	Validation of a robust and sensitive method for detecting hydroxyl radical formation together with evoked neurotransmitter release in brain microdialysis	2008	105	738-749	J Neurochem
Fu, L. W.; Longhurst, J. C.	Electroacupuncture modulates vlPAG release of GABA through presynaptic cannabinoid CB1 receptors	2009	106	1800-1809	J Appl Physiol (1985)
Fukuyama, K.; Tanahashi, S.; Nakagawa, M.; Yamamura, S.; Motomura, E.; Shiroyama, T.; Tanii, H.; Okada, M.	Levetiracetam inhibits neurotransmitter release associated with CICR	2012	518	69-74	Neurosci Lett
Fuxe, K.; Connor, W. T.; Antonelli, T.; Osborne, P. G.; Tanganelli, S.; Agnati, L. F.; Ungerstedt, U.	Evidence for a substrate of neuronal plasticity based on pre- and postsynaptic neurotensin-dopamine receptor interactions in the neostriatum	1992	89	5591-5595	Proc Natl Acad Sci U S A
Gabriel, E. M.; Inglefield, J. R.; Chadwick, L. E.; Schwartz-Bloom, R. D.	Ischemic injury and extracellular amino acid accumulation in hippocampal area CA1 are not dependent upon an intact septo-hippocampal pathway	1998	785	279-286	Brain Res
Galeffi, F.; Bianchi, L.; Bolam, J. P.; Della Corte, L.	The effect of 6-hydroxydopamine lesions on the release of amino acids in the direct and indirect pathways of the basal ganglia: a dual microdialysis probe analysis	2003	18	856-868	Eur J Neurosci
Galindo, A.; Del Arco, A.; Mora, F.	Endogenous GABA potentiates the potassium-induced release of dopamine in striatum of the freely moving rat: a microdialysis study	1999	50	209-214	Brain Res Bull
Gallegos-Sanchez, J.; Picard, S.; Delaleu, B.; Malpaux, B.; Thiery, J. C.	Initiation of the oestradiol-induced inhibition of pulsatile LH secretion in ewes under long days: comparison of peripheral versus central treatment and neurochemical correlates	1996	151	19-28	J Endocrinol
Galvan, A.; Villalba, R. M.; West, S. M.; Maidment, N. T.; Ackerson, L. C.; Smith, Y.; Wichmann, T.	GABAergic modulation of the activity of globus pallidus neurons in primates: in vivo analysis of the functions of GABA receptors and GABA transporters	2005	94	990-1000	J Neurophysiol
Geddes, J. W.; Chang, N. G.; Ackley, D. C.; Soultanian, N. S.; McGillis, J. P.; Yokel, R. A.	Postmortem elevation in extracellular glutamate in the rat hippocampus when brain temperature is maintained at physiological levels: implications for the use of human brain autopsy tissues	1999	8631	104-112	Brain Res
Getting, S. J.; Segieth, J.; Ahmad, S.; Biggs, C. S.; Whitton, P. S.	Biphasic modulation of GABA release by nitric oxide in the hippocampus of freely moving rats in vivo	1996	717	196-199	Brain Res

GABA					
**Authors**	**Title**	**Year**	**Vol**	**Pages**	**Journal**
Gianfriddo, M.; Melani, A.; Turchi, D.; Giovannini, M. G.; Pedata, F.	Adenosine and glutamate extracellular concentrations and mitogen-activated protein kinases in the striatum of Huntington transgenic mice. Selective antagonism of adenosine A2A receptors reduces transmitter outflow	2004	17	77-88	Neurobiol Dis
Giorgetti, M.; Bacciottini, L.; Giovannini, M. G.; Colivicchi, M. A.; Goldfarb, J.; Blandina, P.	Local GABAergic modulation of acetylcholine release from the cortex of freely moving rats	2000	12	1941-1948	Eur J Neurosci
Giovannini, M. G.; Camilli, F.; Mundula, A.; Bianchi, L.; Colivicchi, M. A.; Pepeu, G.	Differential regulation by N-methyl-D-aspartate and non-N-methyl-D-aspartate receptors of acetylcholine release from the rat striatum in vivo	1995	65	409-415	Neuroscience
Giovannini, M. G.; Rakovska, A.; Benton, R. S.; Pazzagli, M.; Bianchi, L.; Pepeu, G.	Effects of novelty and habituation on acetylcholine, GABA, and glutamate release from the frontal cortex and hippocampus of freely moving rats	2001	106	43-53	Neuroscience
Globus, M. Y.; Busto, R.; Dietrich, W. D.; Martinez, E.; Valdes, I.; Ginsberg, M. D.	Effect of ischemia on the in vivo release of striatal dopamine, glutamate, and gamma-aminobutyric acid studied by intracerebral microdialysis	1988	51	1455-1464	J Neurochem
Globus, M. Y.; Busto, R.; Martinez, E.; Valdes, I.; Dietrich, W. D.; Ginsberg, M. D.	Comparative effect of transient global ischemia on extracellular levels of glutamate, glycine, and gamma-aminobutyric acid in vulnerable and nonvulnerable brain regions in the rat	1991	57	470-478	J Neurochem
Globus, M. Y.; Ginsberg, M. D.; Busto, R.	Excitotoxic index--a biochemical marker of selective vulnerability	1991	127	39-42	Neurosci Lett
Gobaille, S.; Hechler, V.; Andriamampandry, C.; Kemmel, V.; Maitre, M.	gamma-Hydroxybutyrate modulates synthesis and extracellular concentration of gamma-aminobutyric acid in discrete rat brain regions in vivo	1999	290	303-309	J Pharmacol Exp Ther
Gobert, A.; Rivet, J. M.; Billiras, R.; Parsons, F.; Millan, M. J.	Simultaneous quantification of D- vs. L-serine, taurine, kynurenate, phosphoethanolamine and diverse amino acids in frontocortical dialysates of freely-moving rats: differential modulation by N-methyl-D-aspartate (NMDA) and other pharmacological agents	2011	202	143-157	J Neurosci Methods
Goda, H.; Ooboshi, H.; Nakane, H.; Ibayashi, S.; Sadoshima, S.; Fujishima, M.	Modulation of ischemia-evoked release of excitatory and inhibitory amino acids by adenosine A1 receptor agonist	1998	357	149-155	Eur J Pharmacol
Godukhin, O.	Effect of local cholecystokinin-8 administration on extracellular levels of amino acids and glycolytic products monitored by in vivo	1995	194	29-32	Neurosci Lett

GABA					
**Authors**	**Title**	**Year**	**Vol**	**Pages**	**Journal**
	microdialysis in the fronto-parietal cortex of the rat				
Goldsmith, J. D.; Kujawa, S. G.; McLaren, J. D.; Bledsoe Jr, S. C.	In vivo release of neuroactive amino acids from the inferior colliculus of the guinea pig using brain microdialysis	1995	83	80-88	Hear Res
Gomez, R.; Vargas, C. R.; Wajner, M.; Barros, H. M.	Lower in vivo brain extracellular GABA concentration in diabetic rats during forced swimming	2003	968	281-284	Brain Res
Gonzalez-Usano, A.; Cauli, O.; Agusti, A.; Felipo, V.	Pregnenolone sulfate restores the glutamate-nitric-oxide-cGMP pathway and extracellular GABA in cerebellum and learning and motor coordination in hyperammonemic rats	2014	5	100-105	ASC Chem Neurosci
Goodman, R. L.; Robinson, J. E.; Kendrick, K. M.; Dyer, R. G.	Is the inhibitory action of estradiol on luteinizing hormone pulse frequency in anestrous ewes mediated by noradrenergic neurons in the preoptic area?	1995	61	284-292	Neuroendocrinology
Goren, M. Z.; Aker, R.; Onat, F.; Ergun, A.	The effects of partial bilateral lesioning of substantia nigra in a genetic absence epilepsy rat model	2002	15	79-85	Marmara Med J
Goren, M. Z.; Aker, R.; Yananli, H. R.; Onat, F. Y.	Extracellular concentrations of catecholamines and amino acids in the dorsomedial hypothalamus of kindled rats. A microdialysis study	2003	68	190-197	Pharmacology
Goren, M. Z.; Kucukibrahimoglu, E.; Berkman, K.; Terzioglu, B.	Fluoxetine partly exerts its actions through GABA: a neurochemical evidence	2007	32	1559-1565	Neurochem Res
Goren, M. Z.; Yananli, H. R.; Berkman, K.; Onat, F.; Aker, R.	The influence of dorsomedial hypothalamic nucleus on contralateral paraventricular nucleus in NMDA-mediated cardiovascular responses	2003	968	219-226	Brain Res
Graham, S. H.; Chen, J.; Lan, J.; Leach, M. J.; Simon, R. P.	Neuroprotective effects of a use-dependent blocker of voltage-dependent sodium channels, BW619C89, in rat middle cerebral artery occlusion	1994	269	854-859	J Pharmacol Exp Ther
Graham, S. H.; Shimizu, H.; Newman, A.; Weinstein, P.; Faden, A. I.	Opioid receptor antagonist nalmefene stereospecifically inhibits glutamate release during global cerebral ischemia	1993	632	346-350	Brain Res
Graham, S. H.; Shiraishi, K.; Panter, S. S.; Simon, R. P.; Faden, A. I.	Changes in extracellular amino acid neurotransmitters produced by focal cerebral ischemia	1990	110	124-130	Neurosci Lett
Grimm, J. W.; See, R. E.	Chronic haloperidol-induced alterations in pallidal GABA and striatal D(1)-mediated dopamine turnover as measured by dual probe microdialysis in rats	2000	100	507-514	Neuroscience
Grimm, J. W.; See, R. E.	Unique activation of extracellular striato-pallidal neurotransmitters in rats following acute risperidone	1998	801	182-189	Brain Res

GABA					
**Authors**	**Title**	**Year**	**Vol**	**Pages**	**Journal**
Grobin, A. C.; Deutch, A. Y.	Dopaminergic regulation of extracellular gamma-aminobutyric acid levels in the prefrontal cortex of the rat	1998	285	350-357	J Pharmacol Exp Ther
Gronli, J.; Fiske, E.; Murison, R.; Bjorvatn, B.; Sorensen, E.; Ursin, R.; Portas, C. M.	Extracellular levels of serotonin and GABA in the hippocampus after chronic mild stress in rats. A microdialysis study in an animal model of depression	2007	181	42-51	Behav Brain Res
Gruss, M.; Braun, K.	Stimulus-evoked increase of glutamate in the mediorostral neostriatum/hyperstriatum ventrale of domestic chick after auditory filial imprinting: an in vivo microdialysis study	1996	66	1167-1173	J Neurochem
Guevara-Guzman, R.; Barrera-Mera, B.; De La Riva, C.; Kendrick, K. M.	Release of classical transmitters and nitric oxide in the rat olfactory bulb, evoked by vaginocervical stimulation and potassium, varies with the oestrus cycle	2000	12	80-88	Eur J Neurosci
Guevara-Guzman, R.; Buzo, E.; Larrazolo, A.; de la Riva, C.; Da Costa, A. P.; Kendrick, K. M.	Vaginocervical stimulation-induced release of classical neurotransmitters and nitric oxide in the nucleus of the solitary tract varies as a function of the oestrus cycle	2001	898	303-313	Brain Res
Guevara-Guzman, R.; Emson, P. C.; Kendrick, K. M.	Modulation of in vivo striatal transmitter release by nitric oxide and cyclic GMP	1994	62	807-810	J Neurochem
Guo, Y.; Cai, H.; Chen, L.; Liang, D.; Yang, R.; Dang, R.; Jiang, P.	Quantitative profiling of neurotransmitter abnormalities in the hippocampus of rats treated with lipopolysaccharide: Focusing on kynurenine pathway and implications for depression	2016	295-296	41-46	J Neuroimmunol
Hada, J.; Kaku, T.; Jiang, M. H.; Morimoto, K.; Hayashi, Y.	Inhibition of high K+-evoked gamma-aminobutyric acid release by sodium nitroprusside in rat hippocampus	2003	467	119-123	Eur J Pharmacol
Hamann, M.; Sohr, R.; Morgenstern, R.; Richter, A.	Extracellular amino acid levels in the striatum of the dt(sz) mutant, a model of paroxysmal dystonia	2008	157	188-195	Neuroscience
Han, J.; Wan, H. T.; Yang, J. H.; Zhang, Y. Y.; Ge, L. J.; Bie, X. D.	Effect of ligustrazine on levels of amino acid neurotransmitters in rat striatum after cerebral ischemia-reperfusion injury	2014	16	1060-1067	J Asian Nat Prod Res
Han, J.; Wan, H.; Li, J.; Ge, L.	[Study on 3-methoxy puerarin to dynamic changes of amino acids in rat brain ischemia-reperfusion]	2012	37	1023-1027	Zhongguo Zhong Yao Za Zhi
Han, W.; Qi, J.; Wang, F.; Zhang, L.; Zhao, S.; Song, M.; Wu, C.; Yang, J.	NMDA receptors in the medial prefrontal cortex and the dorsal hippocampus regulate methamphetamine-induced hyperactivity and extracellular amino acid release in mice	2012	232	44-52	Behav Brain Res
Harte, M.; Connor, W. T.	Evidence for a differential medial prefrontal dopamine D1 and D2 receptor regulation of local and ventral tegmental glutamate and GABA release: a dual probe microdialysis study in the awake rat	2004	1017	120-129	Brain Res

GABA					
Authors	Title	Year	Vol	Pages	Journal
Hashimoto, T.; Kuriyama, K.	GABAA receptor-mediated K(+)-evoked GABA release from globus pallidus--analysis using microdialysis	1997	30	247-252	Neurochem Int
Hassel, B.; Dahlberg, D.; Mariussen, E.; Goverud, I. L.; Antal, E. A.; Tonjum, T.; Maehlen, J.	Brain infection with Staphylococcus aureus leads to high extracellular levels of glutamate, aspartate, gamma-aminobutyric acid, and zinc	2014	92	1792-1800	J Neurosci Res
Hata, H.; Sugahara, M.; Shibanoki, S.; Ishikawa, K.	Characterization of NMDA-induced GABA release in rat hippocampus and striatum	1997	23	155-166	J Brain Sci
Hathway, G. J.; Emson, P. C.; Humphrey, P. P.; Kendrick, K. M.	Somatostatin potently stimulates in vivo striatal dopamine and gamma-aminobutyric acid release by a glutamate-dependent action	1998	70	1740-1749	J Neurochem
Haxhiu, M. A.; Yamamoto, B. K.; Dreshaj, I. A.; Ferguson, D. G.	Activation of the midbrain periaqueductal gray induces airway smooth muscle relaxation	2002	93	440-449	J Appl Physiol (1985)
Hegoburu, C.; Sevelinges, Y.; Thevenet, M.; Gervais, R.; Parrot, S.; Mouly, A. M.	Differential dynamics of amino acid release in the amygdala and olfactory cortex during odor fear acquisition as revealed with simultaneous high temporal resolution microdialysis	2009	16	687-697	Learn Mem
Hehre, D. A.; Devia, C. J.; Bancalari, E.; Suguihara, C.	Brainstem amino acid neurotransmitters and ventilatory response to hypoxia in piglets	2008	63	46-50	Pediatr Res
Heidbreder, C.; De Witte, P.	Ethanol differentially affects extracellular monoamines and GABA in the nucleus accumbens	1993	46	477-481	Pharmacol Biochem Behav
Heim, C.; Zhang, J.; Lan, J.; Sieklucka, M.; Kurz, T.; Riederer, P.; Gerlach, M.; Sontag, K. H.	Cerebral oligaemia episode triggers free radical formation and late cognitive deficiencies	2000	12	715-725	Eur J Neurosci
Hemmati, P.; Shilliam, C. S.; Hughes, Z. A.; Shah, A. J.; Roberts, J. C.; Atkins, A. R.; Hunter, A. J.; Heidbreder, C. A.	In vivo characterization of basal amino acid levels in subregions of the rat nucleus accumbens: effect of a dopamine D(3)/D(2) agonist	2001	39	199-208	Neurochem Int
Herbison, A. E.; Chapman, C.; Dyer, R. G.	Role of medial preoptic GABA neurones in regulating luteinising hormone secretion in the ovariectomised rat	1991	87	345-352	Exp Brain Res
Herbison, A. E.; Heavens, R. P.; Dye, S.; Dyer, R. G.	Acute action of oestrogen on medial preoptic gamma-aminobutyric Acid neurons: correlation with oestrogen negative feedback on luteinizing hormone secretion	1991	3	101-106	J Neuroendocrinol
Herbison, A. E.; Heavens, R. P.; Dyer, R. G.	Endogenous release of gamma-aminobutyric acid from the medial preoptic area measured by microdialysis in the anaesthetised rat	1990	55	1617-1623	J Neurochem
Herbison, A. E.; Heavens, R. P.; Dyer,	Oestrogen modulation of excitatory A1 noradrenergic input to rat	1990	52	161-	Neuroendocrinology

GABA					
Authors	Title	Year	Vol	Pages	Journal
R. G.	medial preoptic gamma aminobutyric acid neurones demonstrated by microdialysis			168	
Hernandez, L. F.; Segovia, G.; Mora, F.	Chronic treatment with a dopamine uptake blocker changes dopamine and acetylcholine but not glutamate and GABA concentrations in prefrontal cortex, striatum and nucleus accumbens of the awake rat	2008	52	457-469	Neurochem Int
Hernandez, L. F.; Segovia, G.; Mora, F.	Effects of activation of NMDA and AMPA glutamate receptors on the extracellular concentrations of dopamine, acetylcholine, and GABA in striatum of the awake rat: a microdialysis study	2003	28	1819-1827	Neurochem Res
Hernandez-Rabaza, V.; Cabrera-Pastor, A.; Taoro-Gonzalez, L.; Gonzalez-Usano, A.; Agusti, A.; Balzano, T.; Llansola, M.; Felipo, V.	Neuroinflammation increases GABAergic tone and impairs cognitive and motor function in hyperammonemia by increasing GAT-3 membrane expression. Reversal by sulforaphane by promoting M2 polarization of microglia	2016	13	83	J Neuroinflammation
Heron, A.; Lasbennes, F.; Seylaz, J.	Adenosine modulation of amino acid release in rat hippocampus during ischemia and veratridine depolarization	1993	608	27-32	Brain Res
Higuera-Matas, A.; Miguens, M.; Coria, S. M.; Assis, M. A.; Borcel, E.; del Olmo, N.; Ambrosio, E.	Sex-specific disturbances of the glutamate/GABA balance in the hippocampus of adult rats subjected to adolescent cannabinoid exposure	2012	62	1975-1984	Neuropharmacology
Hiller, A.; Loeffler, S.; Haupt, C.; Litza, M.; Hofmann, U.; Moser, A.	Electrical high frequency stimulation of the caudate nucleus induces local GABA outflow in freely moving rats	2007	159	286-290	J Neurosci Methods
Hirose, N.; Yoshida, Y.; Koide, S.; Takada, K.; Saigusa, T.; Koshikawa, N.	Effects of propofol and fentanyl on extracellular levels of gamma-aminobutyric acid in the rat nucleus accumbens: an in vivo microdialysis study	1998	40	165-170	J Oral Sci
Hondo, H.; Nakahara, T.; Nakamura, K.; Hirano, M.; Uchimura, H.; Tashiro, N.	The effect of phencyclidine on the basal and high potassium evoked extracellular GABA levels in the striatum of freely-moving rats: an in vivo microdialysis study	1995	671	54-62	Brain Res
Hong, Z. Y.; Huang, Z. L.; Qu, W. M.; Eguchi, N.; Urade, Y.; Hayaishi, O.	An adenosine A receptor agonist induces sleep by increasing GABA release in the tuberomammillary nucleus to inhibit histaminergic systems in rats	2005	92	1542-1549	J Neurochem
Hoop, B.; Beagle, J. L.; Maher, T. J.; Kazemi, H.	Brainstem amino acid neurotransmitters and hypoxic ventilatory response	1999	118	117-129	Respir Physiol
Horn, T.; Bauce, L.; Landgraf, R.; Pittman, Q. J.	Microdialysis with high NaCl causes central release of amino acids and dopamine	1995	64	1632-1644	J Neurochem

GABA					
**Authors**	**Title**	**Year**	**Vol**	**Pages**	**Journal**
Horn, T.; Smith, P. M.; McLaughlin, B. E.; Bauce, L.; Marks, G. S.; Pittman, Q. J.; Ferguson, A. V.	Nitric oxide actions in paraventricular nucleus: cardiovascular and neurochemical implications	1994	266	R306-R313	Am J Physiol
Hossain, M. M.; Suzuki, T.; Unno, T.; Komori, S.; Kobayashi, H.	Differential presynaptic actions of pyrethroid insecticides on glutamatergic and GABAergic neurons in the hippocampus	2008	243	155-163	Toxicology
Hu, M.; Watson, C. J.; Kennedy, R. T.; Becker, J. B.	Estradiol attenuates the K+-induced increase in extracellular GABA in rat striatum	2006	59	122-124	Synapse
Hu, R. Q.; Banerjee, P. K.; Snead, O. C., 3rd	Regulation of gamma-aminobutyric acid (GABA) release in cerebral cortex in the gamma-hydroxybutyric acid (GHB) model of absence seizures in rat	2000	39	427-439	Neuropharmacology
Hu, X. J.; Wang, F. H.; Stenfors, C.; Ogren, S. O.; Kehr, J.	Effects of the 5-HT1B receptor antagonist NAS-181 on extracellular levels of acetylcholine, glutamate and GABA in the frontal cortex and ventral hippocampus of awake rats: a microdialysis study	2007	17	580-586	Eur Neuropsychopharmacol
Huang, M.; Felix, A. R.; Flood, D. G.; Bhuvaneswaran, C.; Hilt, D.; Koenig, G.; Meltzer, H. Y.	The novel alpha7 nicotinic acetylcholine receptor agonist EVP-6124 enhances dopamine, acetylcholine, and glutamate efflux in rat cortex and nucleus accumbens	2014	231	4541-4551	Psychopharmacology (Berl)
Huang, M.; Li, Z.; Dai, J.; Shahid, M.; Wong, E. H.; Meltzer, H. Y.	Asenapine increases dopamine, norepinephrine, and acetylcholine efflux in the rat medial prefrontal cortex and hippocampus	2008	33	2934-2945	Neuropsychopharmacology
Huang, M.; Panos, J. J.; Kwon, S.; Oyamada, Y.; Rajagopal, L.; Meltzer, H. Y.	Comparative effect of lurasidone and blonanserin on cortical glutamate, dopamine, and acetylcholine efflux: role of relative serotonin (5-HT)2A and DA D2 antagonism and 5-HT1A partial agonism	2014	128	938-949	J Neurochem
Huo, T.; Zhang, Y.; Li, W.; Yang, H.; Jiang, H.; Sun, G.	Effect of realgar on extracellular amino acid neurotransmitters in hippocampal CA1 region determined by online microdialysis-dansyl chloride derivatization-high-performance liquid chromatography and fluorescence detection	2014	28	1254-1262	Biomed Chromatogr
Hwa, L. S.; Nathanson, A. J.; Shimamoto, A.; Tayeh, J. K.; Wilens, A. R.; Holly, E. N.; Newman, E. L.; DeBold, J. F.; Miczek, K. A.	Aggression and increased glutamate in the mPFC during withdrawal from intermittent alcohol in outbred mice	2015	232	2889-2902	Psychopharmacology (Berl)
Hylland, P.; Nilsson, G. E.	Extracellular levels of amino acid neurotransmitters during anoxia and forced energy deficiency in crucian carp brain	1999	823	49-58	Brain Res

GABA					
**Authors**	**Title**	**Year**	**Vol**	**Pages**	**Journal**
Hylland, P.; Nilsson, G. E.; Johansson, D.	Anoxic brain failure in an ectothermic vertebrate: release of amino acids and K+ in rainbow trout thalamus	1995	269	R1077-R1084	Am J Physiol
Iino, T.; Katsura, M.; Kuriyama, K.	Protective effect of vinconate on ischemia-induced neuronal damage in the rat hippocampus	1992	224	117-124	Eur J Pharmacol
Inglefield, J. R.; Perry, J. M.; Schwartz, R. D.	Postischemic inhibition of GABA reuptake by tiagabine slows neuronal death in the gerbil hippocampus	1995	5	460-468	Hippocampus
Inui, T.; Yamamoto, T.; Shimura, T.	GABAergic transmission in the rat ventral pallidum mediates a saccharin palatability shift in conditioned taste aversion	2009	30	110-115	Eur J Neurosci
Invernizzi, R. W.; Pierucci, M.; Calcagno, E.; Di Giovanni, G.; Di Matteo, V.; Benigno, A.; Esposito, E.	Selective activation of 5-HT(2C) receptors stimulates GABA-ergic function in the rat substantia nigra pars reticulata: a combined in vivo electrophysiological and neurochemical study	2007	144	1523-1535	Neuroscience
Ipponi, A.; Lamberti, C.; Medica, A.; Bartolini, A.; Malmberg-Aiello, P.	Tiagabine antinociception in rodents depends on GABA(B) receptor activation: parallel antinociception testing and medial thalamus GABA microdialysis	1999	368	205-211	Eur J Pharmacol
Ishide, T.; Hara, Y.; Maher, T. J.; Ally, A.	Glutamate neurotransmission and nitric oxide interaction within the ventrolateral medulla during cardiovascular responses to muscle contraction	2000	874	107-115	Brain Res
Ishide, T.; Maher, T. J.; Pearce, W. J.; Nauli, S. M.; Chaiyakul, P.; Ally, A.	Simultaneous glutamate and gamma-aminobutyric acid release within ventrolateral medulla during skeletal muscle contraction in intact and barodenervated rats	2001	923	137-146	Brain Res
Ishide, T.; Preuss, C. V.; Maher, T. J.; Ally, A.	Neurochemistry within ventrolateral medulla and cardiovascular effects during static exercise following eNOS antagonism	2005	52	21-30	Neurosci Res
Ishiwari, K.; Mingote, S.; Correa, M.; Trevitt, J. T.; Carlson, B. B.; Salamone, J. D.	The GABA uptake inhibitor beta-alanine reduces pilocarpine-induced tremor and increases extracellular GABA in substantia nigra pars reticulata as measured by microdialysis	2004	140	39-46	J Neurosci Methods
Ishiwata, S.; Umino, A.; Balu, D. T.; Coyle, J. T.; Nishikawa, T.	Neuronal serine racemase regulates extracellular D-serine levels in the adult mouse hippocampus	2015	122	1099-1103	J Neural Transm (Vienna)
Ishiwata, T.; Saito, T.; Hasegawa, H.; Yazawa, T.; Kotani, Y.; Otokawa, M.; Aihara, Y.	Changes of body temperature and thermoregulatory responses of freely moving rats during GABAergic pharmacological stimulation to the preoptic area and anterior hypothalamus in several ambient temperatures	2005	1048	32-40	Brain Res
Izurieta-Sanchez, P.; Sarre, S.; Ebinger, G.; Michotte, Y.	Muscarinic antagonists in substantia nigra influence the decarboxylation of L-dopa in striatum	2000	399	151-160	Eur J Pharmacol

GABA					
**Authors**	**Title**	**Year**	**Vol**	**Pages**	**Journal**
Jacobson, I.; Hagberg, H.; Sandberg, M.; Hamberger, A.	Ouabain-induced changes in extracellular aspartate, glutamate and GABA levels in the rabbit olfactory bulb in vivo	1986	64	211-215	Neurosci Lett
Jacobsson, S. O.; Cassel, G. E.; Karlsson, B. M.; Sellstrom, A.; Persson, S. A.	Release of dopamine, GABA and EAA in rats during intrastriatal perfusion with kainic acid, NMDA and soman: a comparative microdialysis study	1997	71	756-765	Arch Toxicol
Jacobsson, S. O.; Sellstrom, A.; Persson, S. A.; Cassel, G. E.	Correlation between cortical EEG and striatal microdialysis in soman-intoxicated rats	1997	231	155-158	Neurosci Lett
Jayaram, P.; Steketee, J. D.	Cocaine-induced increases in medial prefrontal cortical GABA transmission involves glutamatergic receptors	2006	531	74-79	Eur J Pharmacol
Jayaram, P.; Steketee, J. D.	Effects of cocaine-induced behavioural sensitization on GABA transmission within rat medial prefrontal cortex	2005	21	2035-2039	Eur J Neurosci
Jayaram, P.; Steketee, J. D.	Effects of repeated cocaine on medial prefrontal cortical GABAB receptor modulation of neurotransmission in the mesocorticolimbic dopamine system	2004	90	839-847	J Neurochem
Jia, J.; Hu, Y. S.; Wu, Y.; Liu, G.; Yu, H. X.; Zheng, Q. P.; Zhu, D. N.; Xia, C. M.; Cao, Z. J.	Pre-ischemic treadmill training affects glutamate and gamma aminobutyric acid levels in the striatal dialysate of a rat model of cerebral ischemia	2009	84	505-511	Life Sci
Jiao, Y.; Wang, D. Q.; Wu, Z. E.; Fan, B.; Peng, J.; Wang, X. Y.	The effect of extracellular amino acid neurotransmitters in striatum of rats by intracerebral perfusion of 6-OHDA and Madopar intervene. [Chinese]	2009	25	1055-1059	Zhongguo yao li xue tong bao
Jimenez-Diaz, L.; Gruart, A.; Minano, F. J.; Delgado-Garcia, J. M.	An experimental study of posterior interpositus involvement in the genesis and control of conditioned eyelid responses	2002	978	106-118	Ann N Y Acad Sci
Johansen, F. F.; Christensen, T.; Jensen, M. S.; Valente, E.; Jensen, C. V.; Nathan, T.; Lambert, J. D.; Diemer, N. H.	Inhibition in postischemic rat hippocampus: GABA receptors, GABA release, and inhibitory postsynaptic potentials	1991	84	529-537	Exp Brain Res
John, J.; Wu, M. F.; Kodama, T.; Siegel, J. M.	Intravenously administered hypocretin-1 alters brain amino acid release: an in vivo microdialysis study in rats	2003	548	557-562	J Physiol
Jolkkonen, J.; Mazurkiewicz, M.; Lahtinen, H.; Riekkinen, P.	Acute effects of gamma-vinyl GABA on the GABAergic system in rats as studied by microdialysis	1992	229	269-272	Eur J Pharmacol
Juhasz, G.; Kekesi, K. A.; Nyitrai, G.; Dobolyi, A.; Krogsgaard-Larsen, P.; Schousboe, A.	Differential effects of nipecotic acid and 4,5,6,7-tetrahydroisoxazolo[4,5-c]pyridin-3-ol on extracellular gamma-aminobutyrate levels in rat thalamus	1997	331	139-144	Eur J Pharmacol

GABA					
**Authors**	**Title**	**Year**	**Vol**	**Pages**	**Journal**
Kahn, R. A.; Panah, M.; Kiffel, S.; Weinberger, J.	Modulation of ischemic excitatory neurotransmitter and gamma-aminobutyric acid release during global temporary cerebral ischemia by local nitric oxide synthase inhibition	1997	84	1004-1010	Anesth Analg
Kahn, R. A.; Panah, M.; Weinberger, J.	Modulation of ischemic excitatory neurotransmitter and gamma-aminobutyric acid release during global temporary cerebral ischemia by selective neuronal nitric oxide synthase inhibition	1997	84	997-1003	Anesth Analg
Kalivas, P. W.; Duffy, P.	D1 receptors modulate glutamate transmission in the ventral tegmental area	1995	15	5379-5388	J Neurosci
Kang, L.; Dai, Z. Z.; Li, H. H.; Ma, L.	Environmental cues associated with morphine modulate release of glutamate and gamma-aminobutyric acid in ventral subiculum	2006	22	255-260	Neurosci Bull
Kano, T.; Shimizu-Sasamata, M.; Huang, P. L.; Moskowitz, M. A.; Lo, E. H.	Effects of nitric oxide synthase gene knockout on neurotransmitter release in vivo	1998	86	695-699	Neuroscience
Kapasova, Z.; Szumlinski, K. K.	Strain differences in alcohol-induced neurochemical plasticity: a role for accumbens glutamate in alcohol intake	2008	32	617-631	Alcohol Clin Exp Res
Kapoor, V.; Nakahara, D.; Blood, R. J.; Chalmers, J. P.	Preferential release of neuroactive amino acids from the ventrolateral medulla of the rat in vivo as measured by microdialysis	1990	37	187-191	Neuroscience
Karlsson, G. A.; Chaitoff, K. A.; Hossain, S.; Bohlke, M.; Maher, T. J.; Ally, A.	Modulation of cardiovascular responses and neurotransmission during peripheral nociception following nNOS antagonism within the periaqueductal gray	2007	1143	150-160	Brain Res
Kasper, J. M.; Booth, R. G.; Peris, J.	Serotonin-2C receptor agonists decrease potassium-stimulated GABA release in the nucleus accumbens	2015	69	78-85	Synapse
Kasper, J. M.; McCue, D. L.; Milton, A. J.; Szwed, A.; Sampson, C. M.; Huang, M.; Carlton, S.; Meltzer, H. Y.; Cunningham, K. A.; Hommel, J. D.	Gamma-Aminobutyric Acidergic Projections From the Dorsal Raphe to the Nucleus Accumbens Are Regulated by Neuromedin U	2016	80	878-887	Biol Psychiatry
Katayama, S.; Irifune, M.; Kikuchi, N.; Takarada, T.; Shimizu, Y.; Endo, C.; Takata, T.; Dohi, T.; Sato, T.; Kawahara, M.	Increased gamma-aminobutyric acid levels in mouse brain induce loss of righting reflex, but not immobility, in response to noxious stimulation	2007	104	1422-1429	Anesth Analg
Katoh, H.; Shima, K.; Nawashiro, H.; Wada, K.; Chigasaki, H.	Selective hippocampal damage to hypoxia after mild closed head injury in the rat	1998	supplement. 71	247-249	Acta neurochirurgica

GABA					
**Authors**	**Title**	**Year**	**Vol**	**Pages**	**Journal**
Katoh, H.; Sima, K.; Nawashiro, H.; Wada, K.; Chigasaki, H.	The effect of MK-801 on extracellular neuroactive amino acids in hippocampus after closed head injury followed by hypoxia in rats	1997	758	153-162	Brain Res
Kaul, S.; Faiman, M. D.; Lunte, C. E.	Determination of GABA, glutamate and carbamathione in brain microdialysis samples by capillary electrophoresis with fluorescence detection	2011	32	284-291	Electrophoresis
Kaura, S.; Bradford, H. F.; Young, A. M.; Croucher, M. J.; Hughes, P. D.	Effect of amygdaloid kindling on the content and release of amino acids from the amygdaloid complex: in vivo and in vitro studies	1995	65	1240-1249	J Neurochem
Keck, M. E.; Sillaber, I.; Ebner, K.; Welt, T.; Toschi, N.; Kaehler, S. T.; Singewald, N.; Philippu, A.; Elbel, G. K.; Wotjak, C. T.; Holsboer, F.; Landgraf, R.; Engelmann, M.	Acute transcranial magnetic stimulation of frontal brain regions selectively modulates the release of vasopressin, biogenic amines and amino acids in the rat brain	2000	12	3713-3720	Eur J Neurosci
Keefe, K. A.; Sved, A. F.; Zigmond, M. J.; Abercrombie, E. D.	Stress-induced dopamine release in the neostriatum: evaluation of the role of action potentials in nigrostriatal dopamine neurons or local initiation by endogenous excitatory amino acids	1993	61	1943-1952	J Neurochem
Kehr, J.; Ungerstedt, U.	Fast HPLC estimation of gamma-aminobutyric acid in microdialysis perfusates: effect of nipecotic and 3-mercaptopropionic acids	1988	51	1308-1310	J Neurochem
Kekesi, K. A.; Dobolyi, A.; Salfay, O.; Nyitrai, G.; Juhasz, G.	Slow wave sleep is accompanied by release of certain amino acids in the thalamus of cats	1997	8	1183-1186	Neuroreport
Kelly, C. J.; Huang, M.; Meltzer, H.; Martina, M.	Reduced Glutamatergic Currents and Dendritic Branching of Layer 5 Pyramidal Cells Contribute to Medial Prefrontal Cortex Deactivation in a Rat Model of Neuropathic Pain	2016	10	133	Front Cell Neurosci
Kemppainen, H.; Raivio, N.; Nurmi, H.; Kiianmaa, K.	GABA and glutamate overflow in the VTA and ventral pallidum of alcohol-preferring AA and alcohol-avoiding ANA rats after ethanol	2010	45	111-118	Alcohol Alcohol
Kendrick, K. M.; Fabre-Nys, C.; Blache, D.; Goode, J. A.; Broad, K. D.	The role of oxytocin release in the mediobasal hypothalamus of the sheep in relation to female sexual receptivity	1993	5	13-21	J Neuroendocrinol
Kendrick, K. M.; Guevara-Guzman, R.; de la Riva, C.; Christensen, J.; Ostergaard, K.; Emson, P. C.	NMDA and kainate-evoked release of nitric oxide and classical transmitters in the rat striatum: in vivo evidence that nitric oxide may play a neuroprotective role	1996	8	2619-2634	Eur J Neurosci
Kendrick, K. M.; Guevara-Guzman, R.; Zorrilla, J.; Hinton, M. R.; Broad, K. D.; Mimmack, M.; Ohkura, S.	Formation of olfactory memories mediated by nitric oxide	1997	388	670-674	Nature
Kendrick, K. M.; Hinton, M. R.	Microdialysis sampling of in vivo neurotransmitter release in the	1992	446	66P	J Physiol (Lond)

GABA					
**Authors**	**Title**	**Year**	**Vol**	**Pages**	**Journal**
	brain of the conscious sheep: Measurement of acetylcholine, amino acid and monoamine concentrations by high performance liquid chromatography (HPLC)				
Kendrick, K. M.; Hinton, M. R.; Baldwin, B. A.	GABA release in the zona incerta of the sheep in response to the sight and ingestion of food and salt	1991	550	165-168	Brain Res
Kendrick, K. M.; Keverne, E. B.; Chapman, C.; Baldwin, B. A.	Microdialysis measurement of oxytocin, aspartate, gamma-aminobutyric acid and glutamate release from the olfactory bulb of the sheep during vaginocervical stimulation	1988	442	171-174	Brain Res
Kendrick, K. M.; Keverne, E. B.; Hinton, M. R.; Goode, J. A.	Oxytocin, amino acid and monoamine release in the region of the medial preoptic area and bed nucleus of the stria terminalis of the sheep during parturition and suckling	1992	569	199-209	Brain Res
Kennedy, R. T.; Thompson, J. E.; Vickroy, T. W.	In vivo monitoring of amino acids by direct sampling of brain extracellular fluid at ultralow flow rates and capillary electrophoresis	2002	114	39-49	J Neurosci Methods
Keverne, E. B.; Levy, F.; Guevara-Guzman, R.; Kendrick, K. M.	Influence of birth and maternal experience on olfactory bulb neurotransmitter release	1993	56	557-565	Neuroscience
Khan, G. M.; Smolders, I.; Ebinger, G.; Michotte, Y.	Anticonvulsant effect and neurotransmitter modulation of focal and systemic 2-chloroadenosine against the development of pilocarpine-induced seizures	2000	39	2418-2432	Neuropharmacology
Khan, G. M.; Smolders, I.; Lindekens, H.; Manil, J.; Ebinger, G.; Michotte, Y.	Effects of diazepam on extracellular brain neurotransmitters in pilocarpine-induced seizures in rats	1999	373	153-161	Eur J Pharmacol
Kishi, T.; Hirooka, Y.; Sakai, K.; Shigematsu, H.; Shimokawa, H.; Takeshita, A.	Overexpression of eNOS in the RVLM causes hypotension and bradycardia via GABA release	2001	38	896-901	Hypertension
Kishi, T.; Hirooka, Y.; Sunagawa, K.	Telmisartan inhibits sympathetic nerve activity through the inhibition of oxidative stress and increase in NO and GABA in the brain of hypertensive rats	2010	22	S60	Nitric Oxide
Kishi, T.; Hirooka, Y.; Sunagawa, K.	Telmisartan inhibits sympathetic nerve activity through the reduction of oxidative stress and release of GABA in RVLM of hypertensive rats	2010	24	/	FASEB J. Conference: Experimental Biology
Kitta, T.; Matsumoto, M.; Tanaka, H.; Mitsui, T.; Yoshioka, M.; Nonomura, K.	GABAergic mechanism mediated via D receptors in the rat periaqueductal gray participates in the micturition reflex: an in vivo microdialysis study	2008	27	3216-3225	Eur J Neurosci

GABA					
**Authors**	**Title**	**Year**	**Vol**	**Pages**	**Journal**
Kliem, M. A.; Maidment, N. T.; Ackerson, L. C.; Chen, S.; Smith, Y.; Wichmann, T.	Activation of nigral and pallidal dopamine D1-like receptors modulates basal ganglia outflow in monkeys	2007	98	1489-1500	J Neurophysiol
Klinker, C. C.; Bowser, M. T.	4-fluoro-7-nitro-2,1,3-benzoxadiazole as a fluorogenic labeling reagent for the in vivo analysis of amino acid neurotransmitters using online microdialysis-capillary electrophoresis	2007	79	8747-8754	Anal Chem
Klitenick, M. A.; DeWitte, P.; Kalivas, P. W.	Regulation of somatodendritic dopamine release in the ventral tegmental area by opioids and GABA: an in vivo microdialysis study	1992	12	2623-2632	J Neurosci
Kodama, T.; Lai, Y. Y.; Siegel, J. M.	Changes in inhibitory amino acid release linked to pontine-induced atonia: an in vivo microdialysis study	2003	23	1548-1554	J Neurosci
Kokaia, M.; Aebischer, P.; Elmer, E.; Bengzon, J.; Kalen, P.; Kokaia, Z.; Lindvall, O.	Seizure suppression in kindling epilepsy by intracerebral implants of GABA- but not by noradrenaline-releasing polymer matrices	1994	100	385-394	Exp Brain Res
Kolachana, B. S.; Saunders, R. C.; Weinberger, D. R.	In vivo characterization of extracellular GABA release in the caudate nucleus and prefrontal cortex of the rhesus monkey	1997	25	285-292	Synapse
Kolosowska, K.; Maciejak, P.; Szyndler, J.; Turzynska, D.; Sobolewska, A.; Plaznik, A.	The role of IL-1beta and glutamate in the effects of lipopolysaccharide on the hippocampal electrical kindling of seizures	2016	298	146-152	J Neuroimmunol
Korkmaz, O. T.; Tuncel, N.; Tuncel, M.; Oncu, E. M.; Sahinturk, V.; Celik, M.	Vasoactive intestinal peptide (VIP) treatment of Parkinsonian rats increases thalamic gamma-aminobutyric acid (GABA) levels and alters the release of nerve growth factor (NGF) by mast cells	2010	41	278-287	J Mol Neurosci
Kovacs, A.; Mihaly, A.; Komaromi, A.; Gyengesi, E.; Szente, M.; Weiczner, R.; Krisztin-Peva, B.; Szabo, G.; Telegdy, G.	Seizure, neurotransmitter release, and gene expression are closely related in the striatum of 4-aminopyridine-treated rats	2003	55	117-129	Epilepsy Res
Krebs-Kraft, D. L.; Rauw, G.; Baker, G. B.; Parent, M. B.	Zero net flux estimates of septal extracellular glucose levels and the effects of glucose on septal extracellular GABA levels	2009	611	44-52	Eur J Pharmacol
Kuntz, A.; Clement, H. W.; Lehnert, W.; van Calker, D.; Hennighausen, K.; Gerlach, M.; Schulz, E.	Effects of secretin on extracellular amino acid concentrations in rat hippocampus	2004	111	931-939	J Neural Transm (Vienna)
Kusaka, T.; Matsuura, S.; Fujikawa, Y.; Okubo, K.; Kawada, K.; Namba, M.;	Relationship between cerebral interstitial levels of amino acids and phosphorylation potential during secondary energy failure in	2004	55	273-279	Pediatr Res

GABA					
**Authors**	**Title**	**Year**	**Vol**	**Pages**	**Journal**
Okada, H.; Imai, T.; Isobe, K.; Itoh, S.	hypoxic-ischemic newborn piglets				
Lallement, G.; Carpentier, P.; Pernot-Marino, I.; Baubichon, D.; Collet, A.; Blanchet, G.	Transient impairment of the gabaergic function during initiation of soman-induced seizures	1993	629	239-244	Brain Res
Langlais, P. J.; Shu Xing, Zhang	Extracellular glutamate is increased in thalamus during thiamine deficiency-induced lesions and is blocked by MK-801	1993	61	2175-2182	J Neurochem
Largo, C.; Cuevas, P.; Somjen, G. G.; Martin del Rio, R.; Herreras, O.	The effect of depressing glial function in rat brain in situ on ion homeostasis, synaptic transmission, and neuron survival	1996	16	1219-1229	J Neurosci
Lasley, S. M.; Gilbert, M. E.	Rat hippocampal glutamate and GABA release exhibit biphasic effects as a function of chronic lead exposure level	2002	66	139-147	Toxicol Sci
Lasley, S. M.; Gilbert, M. E.	Presynaptic glutamatergic function in dentate gyrus in vivo is diminished by chronic exposure to inorganic lead	1996	736	125-134	Brain Res
Lasley, S. M.; Yan, Q. S.	Diminished potassium-stimulated GABA release in vivo in genetically epilepsy-prone rats	1994	175	145-148	Neurosci Lett
Lehmann, A.	Alterations in hippocampal extracellular amino acids and purine catabolites during limbic seizures induced by folate injections into the rabbit amygdala	1987	22	573-578	Neuroscience
Lehmann, A.	Abnormalities in the levels of extracellular and tissue amino acids in the brain of the seizure-susceptible rat	1989	3	130-137	Epilepsy Res
Lehmann, A.	Evidence for a direct action of N-methylaspartate on non-neuronal cells	1987	411	95-101	Brain Res
Lehmann, A.; Hagberg, H.; Huxtable, R. J.; Sandberg, M.	Reduction of brain taurine: Effects on neurotoxic and metabolic actions of kainate	1987	10	265-274	Neurochem Int
Lehner, M.; Taracha, E.; Skorzewska, A.; Turzynska, D.; Sobolewska, A.; Maciejak, P.; Szyndler, J.; Hamed, A.; Bidzinski, A.; Wislowska-Stanek, A.; Plaznik, A.	Expression of c-Fos and CRF in the brains of rats differing in the strength of a fear response	2008	188	154-167	Behav Brain Res
Lehner, M.; Taracha, E.; Turzynska, D.; Sobolewska, A.; Hamed, A.; Kolomanska, P.; Skorzewska, A.; Maciejak, P.; Szyndler, J.; Bidzinski, A.; Plaznik, A.	The role of the dorsomedial part of the prefrontal cortex serotonergic innervation in rat responses to the aversively conditioned context: behavioral, biochemical and immunocytochemical studies	2008	192	203-215	Behav Brain Res

GABA					
**Authors**	**Title**	**Year**	**Vol**	**Pages**	**Journal**
Lekieffre, D.; Callebert, J.; Plotkine, M.; Boulu, R. G.	Concomitant increases in the extracellular concentrations of excitatory and inhibitory amino acids in the rat hippocampus during forebrain ischemia	1992	137	78-82	Neurosci Lett
Len, W. B.; Chan, J. Y.	Rostral ventrolateral medulla suppresses reflex bradycardia by the release of gamma-aminobutyric acid in nucleus tractus solitarii of the rat	2001	39	23-31	Synapse
Len, W. B.; Chan, J. Y.	GABAergic neurotransmission at the nucleus tractus solitarii in the suppression of reflex bradycardia by parabrachial nucleus	2001	42	27-39	Synapse
Leng, G.; Brown, C. H.; Bull, P. M.; Brown, D.; Scullion, S.; Currie, J.; Blackburn-Munro, R. E.; Feng, J.; Onaka, T.; Verbalis, J. G.; Russell, J. A.; Ludwig, M.	Responses of magnocellular neurons to osmotic stimulation involves coactivation of excitatory and inhibitory input: an experimental and theoretical analysis	2001	21	6967-6977	J Neurosci
Lerma, J.; Herranz, A. S.; Herreras, O.	In vivo determination of extracellular concentration of amino acids in the rat hippocampus. A method based on brain dialysis and computerized analysis	1986	384	145-155	Brain Research
Lerma, J.; Herreras, O.; Herranz, A. S.; Munoz, D.; del Rio, R. M.	In vivo effects of nipecotic acid on levels of extracellular GABA and taurine, and hippocampal excitability	1984	23	595-598	Neuropharmacology
Leung, L. Y.; Tong, K. Y.; Zhang, S. M.; Zeng, X. H.; Zhang, K. P.; Zheng, X. X.	Neurochemical effects of exercise and neuromuscular electrical stimulation on brain after stroke: a microdialysis study using rat model	2006	397	135-139	Neurosci Lett
Levy, F.; Kendrick, K. M.; Goode, J. A.; Guevara-Guzman, R.; Keverne, E. B.	Oxytocin and vasopressin release in the olfactory bulb of parturient ewes: changes with maternal experience and effects on acetylcholine, gamma-aminobutyric acid, glutamate and noradrenaline release	1995	669	197-206	Brain Res
Li, G.; Lv, J.; Wang, J.; Wan, P.; Li, Y.; Jiang, H.; Jin, Q.	GABAB receptors in the hippocampal dentate gyrus are involved in spatial learning and memory impairment in a rat model of vascular dementia	2016	124	190-197	Brain Res Bull
Li, H.; Li, C.; Yan, Z. Y.; Yang, J.; Chen, H.	Simultaneous monitoring multiple neurotransmitters and neuromodulators during cerebral ischemia/reperfusion in rats by microdialysis and capillary electrophoresis	2010	189	162-168	J Neurosci Methods
Li, H.; Yan, Z. Y.	Analysis of amino acid neurotransmitters in hypothalamus of rats during cerebral ischemia-reperfusion by microdialysis and capillary	2010	24	1185-1192	Biomed Chromatogr

GABA					
Authors	Title	Year	Vol	Pages	Journal
	electrophoresis				
Li, P. A.; He, Q. P.; Miyashita, H.; Howllet, W.; Siesjo, B. K.; Shuaib, A.	Hypothermia ameliorates ischemic brain damage and suppresses the release of extracellular amino acids in both normo- and hyperglycemic subjects	1999	158	242-253	Exp Neurol
Li, T. F.; Xue, W.; Li, M.; Shi, A. X.; Li, K. X.	Establishment of an LC-MS/MS method to determine content of six amino acids in rat brain dialysate. [Chinese]	2015	24	1659-1664	Zhongguo xin yao za zhi
Li, X. L.; An, Y.; Jin, Q. H.; Kim, M. S.; Park, B. R.; Jin, Y. Z.	Changes of some amino acid concentrations in the medial vestibular nucleus of conscious rats following acute hypotension	2010	477	11-14	Neurosci Lett
Li, X. M.; Ferraro, L.; Tanganelli, S.; Connor, W. T.; Hasselrot, U.; Ungerstedt, U.; Fuxe, K.	Neurotensin peptides antagonistically regulate postsynaptic dopamine D2 receptors in rat nucleus accumbens: a receptor binding and microdialysis study	1995	102	125-137	J Neural Transm Gen Sect
Li, X.; Gardner, E. L.; Xi, Z. X.	The metabotropic glutamate receptor 7 (mGluR7) allosteric agonist AMN082 modulates nucleus accumbens GABA and glutamate, but not dopamine, in rats	2008	54	542-551	Neuropharmacology
Li, Y. M.; Qu, Y.; Vandenbussche, E.; Arckens, L.; Vandesande, F.	Analysis of extracellular gamma-aminobutyric acid, glutamate and aspartate in cat visual cortex by in vivo microdialysis and capillary electrophoresis-laser induced fluorescence detection	2001	105	211-215	J Neurosci Methods
Li, Z. P.; Zhang, X. Y.; Lu, X.; Zhong, M. K.; Ji, Y. H.	Dynamic release of amino acid transmitters induced by valproate in PTZ-kindled epileptic rat hippocampus	2004	44	263-270	Neurochem Int
Li, Z.; Zhao, Z. X.; Huang, L. Q.; Xiang, Z. H.; Zhang, L.; Zhang, P.; Li, Y. P.; Wu, H. J.; Zhuang, J. H.; Wang, W. Z.; He, B.	Effects of rapid eye movement sleep deprivation and GABAergic drug intervention on cognition in rats. [Chinese]	2011	44	538-543	Zhonghua shen jing ke za zhi
Lillrank, S. M.; Connor, W. T.; Oja, S. S.; Ungerstedt, U.	Systemic phencyclidine administration is associated with increased dopamine, GABA, and 5-HIAA levels in the dorsolateral striatum of conscious rats: an in vivo microdialysis study	1994	95	145-155	J Neural Transm Gen Sect
Lillrank, S. M.; Connor, W. T.; Saransaari, P.; Ungerstedt, U.	In vivo effects of local and systemic phencyclidine on the extracellular levels of catecholamines and transmitter amino acids in the dorsolateral striatum of anaesthetized rats	1994	150	109-115	Acta Physiol Scand
Lin, B.; Globus, M. Y.; Dietrich, W. D.; Busto, R.; Martinez, E.; Ginsberg, M. D.	Differing neurochemical and morphological sequelae of global ischemia: comparison of single- and multiple-insult paradigms	1992	59	2213-2223	J Neurochem
Lin, W. H.; Huang, H. P.; Lin, M. X.; Chen, S. G.	[The effect of high frequency stimulation of epileptic foci on the release of glutamate and gamma-aminobutyric acid in	2011	27	88-92	Zhongguo Ying Yong Sheng Li Xue Za Zhi

GABA					
**Authors**	**Title**	**Year**	**Vol**	**Pages**	**Journal**
	hippocampus of the kainic acid-kindled rats]				
Lindefors, N.; Barati, S.; Connor, W. T.	Differential effects of single and repeated ketamine administration on dopamine, serotonin and GABA transmission in rat medial prefrontal cortex	1997	759	205-212	Brain Res
Lindefors, N.; Brodin, E.; Tossman, U.; Segovia, J.; Ungerstedt, U.	Tissue levels and in vivo release of tachykinins and GABA in striatum and substantia nigra of rat brain after unilateral striatal dopamine denervation	1989	74	527-534	Exp Brain Res
Lindefors, N.; Hurd, Y. L.; Connor, W. T.; Brene, S.; Persson, H.; Ungerstedt, U.	Amphetamine regulation of acetylcholine and gamma-aminobutyric acid in nucleus accumbens	1992	48	439-448	Neuroscience
Lindekens, H.; Smolders, I.; Khan, G. M.; Bialer, M.; Ebinger, G.; Michotte, Y.	In vivo study of the effect of valpromide and valnoctamide in the pilocarpine rat model of focal epilepsy	2000	17	1408-1413	Pharm Res
Littlewood, C. L.; Jones, N.; Neill, M. J.; Mitchell, S. N.; Tricklebank, M.; Williams, S. C.	Mapping the central effects of ketamine in the rat using pharmacological MRI	2006	186	64-81	Psychopharmacology (Berl)
Liu, H. G.; Yang, A. C.; Meng, D. W.; Chen, N.; Zhang, J. G.	Stimulation of the anterior nucleus of the thalamus induces changes in amino acids in the hippocampi of epileptic rats	2012	1477	37-44	Brain Res
Liu, H.; Zhang, G.; Bie, X.; Liu, M.; Yang, J.; Wan, H.; Zhang, Y.	[Effect of polydatin on dynamic changes of excitatory amino acids in cerebrospinal fluid of cerebral hemorrhage rats]	2010	35	3038-3042	Zhongguo Zhong Yao Za Zhi
Liu, P.; He, X. R.; Zhou, W. B.; Shen, R. R.; Feng, F.	[Research for dependablity of administration of platycodi radix in Tianwang Buxinwan decoction with change of brain inhibitive neurotransmitte in rats by microdialysis]	2008	33	2830-2833	Zhongguo Zhong Yao Za Zhi
Liu, P.; Wu, C.; Song, W.; Yu, L.; Yang, X.; Xiang, R.; Wang, F.; Yang, J.	Uridine decreases morphine-induced behavioral sensitization by decreasing dorsal striatal dopamine release possibly via agonistic effects at GABAA receptors	2014	24	1557-1566	Eur Neuropsychopharmacol
Liu, Z.; Zhang, L.; He, Q.; Liu, X.; Chukwunweike Ikechukwu, O.; Tong, L.; Guo, L.; Yang, H.; Zhang, Q.; Zhao, H.; Gu, X.	Effect of Baicalin-loaded PEGylated cationic solid lipid nanoparticles modified by OX26 antibody on regulating the levels of baicalin and amino acids during cerebral ischemia-reperfusion in rats	2015	489	131-138	Int J Pharm
Loidl, C. F.; Herrera-Marschitz, M.; Andersson, K.; You, Z. B.; Goiny, M.; Connor, W. T.; Silveira, R.; Rawal, R.; Bjelke, B.; Chen, Y.; et al.	Long-term effects of perinatal asphyxia on basal ganglia neurotransmitter systems studied with microdialysis in rat	1994	175	9-12	Neurosci Lett

GABA					
**Authors**	**Title**	**Year**	**Vol**	**Pages**	**Journal**
Luna-Munguia, H.; Meneses, A.; Pena-Ortega, F.; Gaona, A.; Rocha, L.	Effects of hippocampal high-frequency electrical stimulation in memory formation and their association with amino acid tissue content and release in normal rats	2012	22	98-105	Hippocampus
Luparini, M. R.; Garrone, B.; Pazzagli, M.; Pinza, M.; Pepeu, G.	A cortical GABA-5HT interaction in the mechanism of action of the antidepressant trazodone	2004	28	1117-1127	Prog Neuropsychopharmacol Biol Psychiatry
Lupinsky, D.; Moquin, L.; Gratton, A.	Interhemispheric regulation of the rat medial prefrontal cortical glutamate stress response: role of local GABA- and dopamine-sensitive mechanisms	2017	234	353-363	Psychopharmacolgy (Berl)
Lupinsky, D.; Moquin, L.; Gratton, A.	Interhemispheric regulation of the rat medial prefrontal cortical glutamate stress response: role of local GABA- and dopamine-sensitive mechanisms	2016	234	1-11	Psychopharmacology
Mabrouk, O. S.; Li, Q.; Song, P.; Kennedy, R. T.	Microdialysis and mass spectrometric monitoring of dopamine and enkephalins in the globus pallidus reveal reciprocal interactions that regulate movement	2011	118	24-33	J Neurochem
Mabrouk, O. S.; Marti, M.; Morari, M.	Endogenous nociceptin/orphanin FQ (N/OFQ) contributes to haloperidol-induced changes of nigral amino acid transmission and parkinsonism: a combined microdialysis and behavioral study in naive and nociceptin/orphanin FQ receptor knockout mice	2010	166	40-48	Neuroscience
Mabrouk, O. S.; Marti, M.; Salvadori, S.; Morari, M.	The novel delta opioid receptor agonist UFP-512 dually modulates motor activity in hemiparkinsonian rats via control of the nigro-thalamic pathway	2009	164	360-369	Neuroscience
Mabrouk, O. S.; Mela, F.; Calcagno, M.; Budri, M.; Viaro, R.; Dekundy, A.; Parsons, C. G.; Auberson, Y. P.; Morari, M.	GluN2A and GluN2B NMDA receptor subunits differentially modulate striatal output pathways and contribute to levodopa-induced abnormal involuntary movements in dyskinetic rats	2013	4	808-816	ACS Chem Neurosci
Mabrouk, O. S.; Semaan, D. Z.; Mikelman, S.; Gnegy, M. E.; Kennedy, R. T.	Amphetamine stimulates movement through thalamocortical glutamate release	2014	128	152-161	J Neurochem
Mabrouk, O. S.; Viaro, R.; Volta, M.; Ledonne, A.; Mercuri, N.; Morari, M.	Stimulation of delta opioid receptor and blockade of nociceptin/orphanin FQ receptor synergistically attenuate parkinsonism	2014	34	12953-12962	J Neurosci
Mabrouk, O. S.; Volta, M.; Marti, M.; Morari, M.	Stimulation of delta opioid receptors located in substantia nigra reticulata but not globus pallidus or striatum restores motor activity	2008	107	1647-1659	J Neurochem

GABA					
**Authors**	**Title**	**Year**	**Vol**	**Pages**	**Journal**
	in 6-hydroxydopamine lesioned rats: new insights into the role of delta receptors in parkinsonism				
Maciejak, P.; Lehner, M.; Turzynska, D.; Szyndler, J.; Bidzinski, A.; Taracha, E.; Sobolewska, A.; Walkowiak, J.; Skorzewska, A.; Wislowska, A.; Hamed, A.; Plaznik, A.	The opposite role of hippocampal mGluR1 in fear conditioning in kindled and non-kindled rats	2008	1187	184-193	Brain Res
Maciejak, P.; Szyndler, J.; Turzynska, D.; Sobolewska, A.; Plaznik, A.	Kynurenic acid: A new effector of valproate action?	2011	63	1569-1573	Pharmacological Reports
Maciejak, P.; Szyndler, J.; Turzynska, D.; Sobolewska, A.; Taracha, E.; Skorzewska, A.; Lehner, M.; Bidzinski, A.; Hamed, A.; Wislowska-Stanek, A.; Plaznik, A.	The effects of group III mGluR ligands on pentylenetetrazol-induced kindling of seizures and hippocampal amino acids concentration	2009	1282	20-27	Brain Res
Macinnes, N.; Duty, S.	Group III metabotropic glutamate receptors act as hetero-receptors modulating evoked GABA release in the globus pallidus in vivo	2008	580	95-99	Eur J Pharmacol
MacKay, K. B.; Patel, T. R.; Galbraith, S. L.; Woodruff, G. N.; McCulloch, J.	The relationship between glutamate release and cerebral blood flow after focal cerebral ischaemia in the cat: effect of pretreatment with enadoline (a kappa receptor agonist)	1996	712	329-334	Brain Res
Maione, S.; Marabese, I.; Oliva, P.; De Novellis, V.; Stella, L.; Filippelli, A.; Rossi, F.	Periaqueductal gray matter glutamate and GABA decrease following subcutaneous formalin injection in rat	1999	10	1403-1407	Neuroreport
Malmberg, A. B.; Hamberger, A.; Hedner, T.	Effects of prostaglandin E2 and capsaicin on behavior and cerebrospinal fluid amino acid concentrations of unanesthetized rats: a microdialysis study	1995	65	2185-2193	J Neurochem
Manto, M.; Laute, M. A.; Pandolfo, M.	Depression of extra-cellular GABA and increase of NMDA-induced nitric oxide following acute intra-nuclear administration of alcohol in the cerebellar nuclei of the rat	2005	4	230-238	Cerebellum
Marabese, I.; de Novellis, V.; Palazzo, E.; Mariani, L.; Siniscalco, D.; Rodella, L.; Rossi, F.; Maione, S.	Differential roles of mGlu8 receptors in the regulation of glutamate and gamma-aminobutyric acid release at periaqueductal grey level	2005	49 Suppl 1	157-166	Neuropharmacology
Mark, K. A.; Soghomonian, J. J.; Yamamoto, B. K.	High-dose methamphetamine acutely-activates the striatonigral pathway to increase striatal glutamate and mediate long-term	2004	24	11449-11456	J Neurosci

GABA					
**Authors**	**Title**	**Year**	**Vol**	**Pages**	**Journal**
	dopamine toxicity				
Marquez de Prado, B.; Castaneda, T. R.; Galindo, A.; del Arco, A.; Segovia, G.; Reiter, R. J.; Mora, F.	Melatonin disrupts circadian rhythms of glutamate and GABA in the neostriatum of the aware rat: a microdialysis study	2000	29	209-216	J Pineal Res
Marti, M.; Rodi, D.; Li, Q.; Guerrini, R.; Fasano, S.; Morella, I.; Tozzi, A.; Brambilla, R.; Calabresi, P.; Simonato, M.; Bezard, E.; Morari, M.	Nociceptin/orphanin FQ receptor agonists attenuate L-DOPA-induced dyskinesias	2012	32	16106-16119	J Neurosci
Marti, M.; Trapella, C.; Morari, M.	The novel nociceptin/orphanin FQ receptor antagonist Trap-101 alleviates experimental parkinsonism through inhibition of the nigro-thalamic pathway: positive interaction with L-DOPA	2008	107	1683-1696	J Neurochem
Marti, M.; Trapella, C.; Viaro, R.; Morari, M.	The nociceptin/orphanin FQ receptor antagonist J-113397 and L-DOPA additively attenuate experimental parkinsonism through overinhibition of the nigrothalamic pathway	2007	27	1297-1307	J Neurosci
Martin, P.; Carlsson, M. L.; Hjorth, S.	Systemic PCP treatment elevates brain extracellular 5-HT: a microdialysis study in awake rats	1998	9	2985-2988	Neuroreport
Martinez-Martos, J. M.; Iribar, M. C.; Peinado, J. M.	Evoked GABA release is not mediated by N-type VDCC in the frontal cortex of awake rats: effects of neomycin	1997	43	441-445	Brain Res Bull
Massie, A.; Cnops, L.; Smolders, I.; Van Damme, K.; Vandenbussche, E.; Vandesande, F.; Eysel, U. T.; Arckens, L.	Extracellular GABA concentrations in area 17 of cat visual cortex during topographic map reorganization following binocular central retinal lesioning	2003	976	100-108	Brain Res
Matsui, M.; Yokoo, H.; Yoshida, M.; Yamada, S.; Tanaka, T.; Mizoguchi, K.; Emoto, H.; Ishii, H.; Nakahara, K.; Kawahara, H.; Anno, N.; Tanaka, M.	Measurement and pharmacological manipulation of extracellular GABA levels in the nucleus accumbens of the rat	1998	14	163-176	Biogenic Amines
Matsui-Okuno, M.; Yokoo, H.; Yoshida, M.; Emoto, H.; Ishii, H.; Annoh, N.; Morinaga, D.; Tanaka, M.	Corticotropin releasing-factor (CRF) increases extracellular GABA levels in the rat hypothalamus	2002	17	141-148	Biogenic Amines
Matsumoto, K.; Lo, E. H.; Pierce, A. R.; Halpern, E. F.; Newcomb, R.	Secondary elevation of extracellular neurotransmitter amino acids in the reperfusion phase following focal cerebral ischemia	1996	16	114-124	J Cereb Blood Flow Metab
Matsumoto, K.; Ueda, S.; Hashimoto, T.; Kuriyama, K.	Ischemic neuronal injury in the rat hippocampus following transient forebrain ischemia: evaluation using in vivo microdialysis	1991	543	236-242	Brain Res

GABA					
**Authors**	**Title**	**Year**	**Vol**	**Pages**	**Journal**
Matsumoto, M.; Togashi, H.; Kaku, A.; Kanno, M.; Tahara, K.; Yoshioka, M.	Cortical GABAergic regulation of dopaminergic responses to psychological stress in the rat dorsolateral striatum	2005	56	117-121	Synapse
Matuszewich, L.; Yamamoto, B. K.	Modulation of GABA release by dopamine in the substantia nigra	1999	32	29-36	Synapse
Maya-Vetencourt, J. F.; Baroncelli, L.; Viegi, A.; Tiraboschi, E.; Castren, E.; Cattaneo, A.; Maffei, L.	IGF-1 restores visual cortex plasticity in adult life by reducing local GABA levels	2012	2012	250421	Neural Plast
McKee, A. G.; Loscher, J. S.; Sullivan, N. C.; Chadderton, N.; Palfi, A.; Batti, L.; Sheridan, G. K.; Shea, S.; Moran, M.; McCabe, O.; Fernandez, A. B.; Pangalos, M. N.; Connor, J. J.; Regan, C. M.; Connor, W. T.; Humphries, P.; Farrar, G. J.; Murphy, K. J.	AAV-mediated chronic over-expression of SNAP-25 in adult rat dorsal hippocampus impairs memory-associated synaptic plasticity	2010	112	991-1004	J Neurohem
McKenzie, J. A.; Watson, C. J.; Rostand, R. D.; German, I.; Witowski, S. R.; Kennedy, R. T.	Automated capillary liquid chromatography for simultaneous determination of neuroactive amines and amino acids	2002	962	105-115	J Chromatogr A
Meana, J. J.; Johansson, B.; Herrera-Marschitz, M.; Connor, W. T.; Goiny, M.; Parkinson, F. E.; Fredholm, B. B.; Ungerstedt, U.	Effect of the neurotoxin AF64A on intrinsic and extrinsic neuronal systems of rat neostriatum measured by in vivo microdialysis	1992	596	65-72	Brain Res
Meeusen, R.; Smolders, I.; Sarre, S.; de Meirleir, K.; Keizer, H.; Serneels, M.; Ebinger, G.; Michotte, Y.	Endurance training effects on neurotransmitter release in rat striatum: an in vivo microdialysis study	1997	159	335-341	Acta Physiol Scand
Mejia-Toiber, J.; Limon-Pacheco, J. H.; Gonzalez-Gallardo, A.; Giordano, M.	In vivo GABA release and kinetics of transgene loss in a GABAergic cell line after long-term transplantation into the rat brain	2012	203	244-254	Neuroscience
Mela, F.; Marti, M.; Bido, S.; Cenci, M. A.; Morari, M.	In vivo evidence for a differential contribution of striatal and nigral D1 and D2 receptors to L-DOPA induced dyskinesia and the accompanying surge of nigral amino acid levels	2012	45	573-582	Neurobiol Dis
Melani, A.; Pantoni, L.; Bordoni, F.; Gianfriddo, M.; Bianchi, L.; Vannucchi, M. G.; Bertorelli, R.; Monopoli, A.; Pedata, F.	The selective A2A receptor antagonist SCH 58261 reduces striatal transmitter outflow, turning behavior and ischemic brain damage induced by permanent focal ischemia in the rat	2003	959	243-250	Brain Res
Melani, A.; Pantoni, L.; Corsi, C.;	Striatal outflow of adenosine, excitatory amino acids, gamma-	1999	30	2448-	Stroke

GABA					
**Authors**	**Title**	**Year**	**Vol**	**Pages**	**Journal**
Bianchi, L.; Monopoli, A.; Bertorelli, R.; Pepeu, G.; Pedata, F.	aminobutyric acid, and taurine in awake freely moving rats after middle cerebral artery occlusion: correlations with neurological deficit and histopathological damage			2455	
Mele, A.; Thomas, D. N.; Pert, A.	Different neural mechanisms underlie dizocilpine maleate- and dopamine agonist-induced locomotor activity	1997	82	43-58	Neuroscience
Mena, F. V.; Baab, P. J.; Zielke, C. L.; Zielke, H. R.	In vivo glutamine hydrolysis in the formation of extracellular glutamate in the injured rat brain	2000	60	632-641	J Neurosci Res
Meng, T.; Yuan, S.; Zheng, Z.; Liu, T.; Lin, L.	Effects of endogenous melatonin on glutamate and GABA rhythms in the striatum of unilateral 6-hydroxydopamine-lesioned rats	2015	286	308-315	Neuroscience
Messam, C. A.; Greene, J. G.; Greenamyre, J. T.; Robinson, M. B.	Intrastriatal injections of the succinate dehydrogenase inhibitor, malonate, cause a rise in extracellular amino acids that is blocked by MK-801	1995	684	221-224	Brain Res
Meurs, A.; Clinckers, R.; Ebinger, G.; Michotte, Y.; Smolders, I.	Seizure activity and changes in hippocampal extracellular glutamate, GABA, dopamine and serotonin	2008	78	50-59	Epilepsy Res
Meurs, A.; Clinckers, R.; Ebinger, G.; Michotte, Y.; Smolders, I.	Substantia nigra is an anticonvulsant site of action of topiramate in the focal pilocarpine model of limbic seizures	2006	47	1519-1535	Epilepsia
Michalak, A.; Rose, C.; Butterworth, J.; Butterworth, R. F.	Neuroactive amino acids and glutamate (NMDA) receptors in frontal cortex of rats with experimental acute liver failure	1996	24	908-913	Hepatology
Miguens, M.; Botreau, F.; Olias, O.; Del Olmo, N.; Coria, S. M.; Higuera-Matas, A.; Ambrosio, E.	Genetic differences in the modulation of accumbal glutamate and gamma-amino butyric acid levels after cocaine-induced reinstatement	2013	18	623-632	Addict Biol
Mileykovskiy, B. Y.; Kiyashchenko, L. I.; Kodama, T.; Lai, Y. Y.; Siegel, J. M.	Activation of pontine and medullary motor inhibitory regions reduces discharge in neurons located in the locus coeruleus and the anatomical equivalent of the midbrain locomotor region	2000	20	8551-8558	J Neurosci
Millan, M. H.; Chapman, A. G.; Meldrum, B. S.	Dual inhibitory action of enadoline (CI977) on release of amino acids in the rat hippocampus	1995	279	75-81	Eur J Pharmacol
Milton, S. L.; Thompson, J. W.; Lutz, P. L.	Mechanisms for maintaining extracellular glutamate levels in the anoxic turtle striatum	2002	282	R1317-R1323	Am J Physiol Regul Integr Comp Physiol
Minamoto, Y.; Itano, T.; Tokuda, M.; Matsui, H.; Janjua, N. A.; Hosokawa, K.; Okada, Y.; Murakami, T. H.; Negi, T.; Hatase, O.	In vivo microdialysis of amino acid neurotransmitters in the hippocampus in amygdaloid kindled rat	1992	573	345-348	Brain Res
Mitsushima, D.; Shwe, T. T.; Funabashi,	GABA release in the medial preoptic area of cyclic female rats	2002	113	109-	Neuroscience

GABA					
**Authors**	**Title**	**Year**	**Vol**	**Pages**	**Journal**
T.; Shinohara, K.; Kimura, F.				114	
Morari, M.; Connor, W. T.; Darvelid, M.; Ungerstedt, U.; Bianchi, C.; Fuxe, K.	Functional neuroanatomy of the nigrostriatal and striatonigral pathways as studied with dual probe microdialysis in the awake rat--I. Effects of perfusion with tetrodotoxin and low-calcium medium	1996	72	79-87	Neuroscience
Morari, M.; Connor, W. T.; Ungerstedt, U.; Bianchi, C.; Fuxe, K.	Functional neuroanatomy of the nigrostriatal and striatonigral pathways as studied with dual probe microdialysis in the awake rat--II. Evidence for striatal N-methyl-D-aspartate receptor regulation of striatonigral GABAergic transmission and motor function	1996	72	89-97	Neuroscience
Morari, M.; Connor, W. T.; Ungerstedt, U.; Fuxe, K.	Dopamine D1 and D2 receptor antagonism differentially modulates stimulation of striatal neurotransmitter levels by N-methyl-D-aspartic acid	1994	256	23-30	Eur J Pharmacol
Morari, M.; Connor, W. T.; Ungerstedt, U.; Fuxe, K.	N-methyl-D-aspartic acid differentially regulates extracellular dopamine, GABA, and glutamate levels in the dorsolateral neostriatum of the halothane-anesthetized rat: an in vivo microdialysis study	1993	60	1884-1893	J Neurochem
Morari, M.; Fantin, M.	Loss of the preferential control over the striato-nigral direct pathway by striatal NMDA receptors in a rat model of Parkinson's disease	2015	140	3830-3839	Analyst
Morrone, L. A.; Rombola, L.; Pelle, C.; Corasaniti, M. T.; Zappettini, S.; Paudice, P.; Bonanno, G.; Bagetta, G.	The essential oil of bergamot enhances the levels of amino acid neurotransmitters in the hippocampus of rat: implication of monoterpene hydrocarbons	2007	55	255-262	Pharmacol Res
Muere, C.; Neumueller, S.; Miller, J.; Olesiak, S.; Hodges, M. R.; Pan, L.; Forster, H. V.	Atropine microdialysis within or near the pre-Botzinger Complex increases breathing frequency more during wakefulness than during NREM sleep	2013	114	694-704	J Appl Physiol (1985)
Mura, E.; Zappettini, S.; Preda, S.; Biundo, F.; Lanni, C.; Grilli, M.; Cavallero, A.; Olivero, G.; Salamone, A.; Govoni, S.; Marchi, M.	Dual effect of beta-amyloid on alpha7 and alpha4beta2 nicotinic receptors controlling the release of glutamate, aspartate and GABA in rat hippocampus	2012	7	e29661	PLoS One
Murai, T.; Yoshida, Y.; Koide, S.; Takada, K.; Misaki, T.; Koshikawa, N.; Cools, A. R.	Clonidine reduces dopamine and increases GABA in the nucleus accumbens: an in vivo microdialysis study	1998	60	695-701	Pharmacol Biochem Behav

GABA					
**Authors**	**Title**	**Year**	**Vol**	**Pages**	**Journal**
Murphy, K. J.; Ter Horst, J. P.; Cassidy, A. W.; DeSouza, I. E.; Morgunova, M.; Li, C.; Connole, L. M.; Sullivan, N. C.; Loscher, J. S.; Brady, A. T.; Rombach, N.; Connellan, J.; McGettigan, P. A.; Scully, D.; Fedriani, R.; Lukasz, B.; Moran, M. P.; McCabe, O. M.; Wantuch, C. M.; Hughes, Z. A.; Mulvany, S. K.; Higgins, D. G.; Pangalos, M. N.; Marquis, K. L.; Connor, W. T.; Ring, R. H.; von Schack, D.; Regan, C. M.	Temporal dysregulation of cortical gene expression in the isolation reared Wistar rat	2010	113	601-614	J Neurochem
Murphy, N. P.; Maidment, N. T.	Orphanin FQ/nociceptin modulation of mesolimbic dopamine transmission determined by microdialysis	1999	73	179-186	J Neurochem
Nagai, T.; Takata, N.; Shinohara, Y.; Hirase, H.	Adaptive changes of extracellular amino acid concentrations in mouse dorsal striatum by 4-AP-induced cortical seizures	2015	295	229-236	Neuroscience
Nail-Boucherie, K.; Le-Pham, B. T.; Gobaille, S.; Maitre, M.; Aunis, D.; Depaulis, A.	Evidence for a role of the parafascicular nucleus of the thalamus in the control of epileptic seizures by the superior colliculus	2005	46	141-145	Epilepsia
Nakashima, K.; Todd, M. M.	Effects of hypothermia, pentobarbital, and isoflurane on postdepolarization amino acid release during complete global cerebral ischemia	1996	85	161-168	Anesthesiology
Nakashima, K.; Todd, M. M.	Effects of hypothermia on the rate of excitatory amino acid release after ischemic depolarization	1996	27	913-918	Stroke
Nakata, N.; Kato, H.; Kogure, K.	Ischemic tolerance and extracellular amino acid concentrations in gerbil hippocampus measured by intracerebral microdialysis	1994	35	247-251	Brain Res Bull
Nakata, N.; Kato, H.; Kogure, K.; DeGraba, T. J.	Effects of repeated cerebral ischemia on extracellular amino acid concentrations measured with intracerebral microdialysis in the gerbil hippocampus	1993	24	458-464	Stroke
Nanda, B.; Galvan, A.; Smith, Y.; Wichmann, T.	Effects of stimulation of the centromedian nucleus of the thalamus on the activity of striatal cells in awake rhesus monkeys	2009	29	588-598	Eur J Neurosci
Narita, M.; Niikura, K.; Nanjo-Niikura, K.; Narita, M.; Furuya, M.; Yamashita, A.; Saeki, M.; Matsushima, Y.; Imai, S.;	Sleep disturbances in a neuropathic pain-like condition in the mouse are associated with altered GABAergic transmission in the cingulate cortex	2011	152	1358-1372	Pain

GABA					
**Authors**	**Title**	**Year**	**Vol**	**Pages**	**Journal**
Shimizu, T.; Asato, M.; Kuzumaki, N.; Okutsu, D.; Miyoshi, K.; Suzuki, M.; Tsukiyama, Y.; Konno, M.; Yomiya, K.; Matoba, M.; Suzuki, T.					
Natividad, L. A.; Buczynski, M. W.; Parsons, L. H.; Torres, O. V.; Dell, L. E.	Adolescent rats are resistant to adaptations in excitatory and inhibitory mechanisms that modulate mesolimbic dopamine during nicotine withdrawal	2012	123	578-588	J Neurochem
Nauli, S. M.; Pearce, W. J.; Amer, A.; Maher, T. J.; Ally, A.	Effects of nitric oxide and GABA interaction within ventrolateral medulla on cardiovascular responses during static muscle contraction	2001	922	234-242	Brain Res
Neal-Perry, G. S.; Zeevalk, G. D.; Shu, J.; Etgen, A. M.	Restoration of the luteinizing hormone surge in middle-aged female rats by altering the balance of GABA and glutamate transmission in the medial preoptic area	2008	79	878-888	Biol Reprod
Nencioni, A. L. A.; Barreto, S. A.; Lebrun, I.; Florio, J. C.; Lourenco, G. A.; Dorce, V. A. C.	Neurotransmitter evaluation in the hippocampus of rats after intracerebral injection of TsTX scorpion toxin	2009	15	236-254	J Venom Anim Toxins Incl Trop Dis
Nencioni, A. L.; Lourenco, G. A.; Lebrun, I.; Florio, J. C.; Dorce, V. A.	Central effects of Tityus serrulatus and Tityus bahiensis scorpion venoms after intraperitoneal injection in rats	2009	463	234-238	Neurosci Lett
Neumann, I.; Landgraf, R.; Bauce, L.; Pittman, Q. J.	Osmotic responsiveness and cross talk involving oxytocin, but not vasopressin or amino acids, between the supraoptic nuclei in virgin and lactating rats	1995	15	3408-3417	J Neurosci
Newcomb, R.; Palma, A.	Effects of diverse omega-conopeptides on the in vivo release of glutamic and gamma-aminobutyric acids	1994	638	95-102	Brain Res
Newcomb, R.; Pierce, A. R.; Kano, T.; Meng, W.; Bosque-Hamilton, P.; Taylor, L.; Curthoys, N.; Lo, E. H.	Characterization of mitochondrial glutaminase and amino acids at prolonged times after experimental focal cerebral ischemia	1998	813	103-111	Brain Res
Nicniocaill, B.; Haraldsson, B.; Hansson, O.; Connor, W. T.; Brundin, P.	Altered striatal amino acid neurotransmitter release monitored using microdialysis in R6/1 Huntington transgenic mice	2001	13	206-210	Eur J Neurosci
Nicol, A. U.; Sanchez-Andrade, G.; Collado, P.; Segonds-Pichon, A.; Kendrick, K. M.	Olfactory bulb encoding during learning under anesthesia	2014	8	193	Front Behav Neurosci
Nilsson, P.; Hillered, L.; Ponten, U.; Ungerstedt, U.	Changes in cortical extracellular levels of energy-related metabolites and amino acids following concussive brain injury in	1990	10	631-637	J Cereb Blood Flow Metab

GABA					
**Authors**	**Title**	**Year**	**Vol**	**Pages**	**Journal**
	rats				
Nilsson, P.; Ronne-Engstrom, E.; Flink, R.; Ungerstedt, U.; Carlson, H.; Hillered, L.	Epileptic seizure activity in the acute phase following cortical impact trauma in rat	1994	637	227-232	Brain Res
Nishino, H.; Aihara, N.; Czurko, A.; Hashitani, T.; Isobe, Y.; Ichikawa, O.; Watari, H.	Reconstruction of GABAergic transmission and behavior by striatal cell grafts in rats with ischemic infarcts in the middle cerebral artery	1993	4	147-155	J Neural Transplant Plast
Nishino, H.; Koide, K.; Aihara, N.; Kumazaki, M.; Sakurai, T.; Nagai, H.	Striatal grafts in the ischemic striatum improve pallidal GABA release and passive avoidance	1993	32	517-520	Brain Res Bull
Nitz, D.; Siegel, J.	GABA release in the dorsal raphe nucleus: role in the control of REM sleep	1997	273	R451-R455	Am J Physiol
Nitz, D.; Siegel, J. M.	GABA release in the locus coeruleus as a function of sleep/wake state	1997	78	795-801	Neuroscience
Nitz, D.; Siegel, J. M.	GABA release in posterior hypothalamus across sleep-wake cycle	1996	271	R1707-R1712	Am J Physiol
Nyitrai, G.; Szarics, E.; Kovacs, I.; Kekesi, K. A.; Juhasz, G.; Kardos, J.	Effect of CGP 36742 on the extracellular level of neurotransmitter amino acids in the thalamus	1999	34	391-398	Neurochem Int
Obata, K.; Fukuda, T.; Konishi, S.; Ji, F. Y.; Mitoma, H.; Kosaka, T.	Synaptic localization of the 67,000 mol. wt isoform of glutamate decarboxylase and transmitter function of GABA in the mouse cerebellum lacking the 65,000 mol. wt isoform	1999	93	1475-1482	Neuroscience
Obrenovitch, T. P.; Richards, D. A.	Extracellular neurotransmitter changes in cerebral ischaemia	1995	7	1-54	Cerebrovasc Brain Metab Rev
Obrenovitch, T. P.; Urenjak, J.; Richards, D. A.; Ueda, Y.; Curzon, G.; Symon, L.	Extracellular neuroactive amino acids in the rat striatum during ischaemia: comparison between penumbral conditions and ischaemia with sustained anoxic depolarisation	1993	61	178-186	J Neurochem
Ochi, M.; Koga, K.; Kurokawa, M.; Kase, H.; Nakamura, J.; Kuwana, Y.	Systemic administration of adenosine A(2A) receptor antagonist reverses increased GABA release in the globus pallidus of unilateral 6-hydroxydopamine-lesioned rats: a microdialysis study	2000	100	53-62	Neuroscience
Ochi, M.; Shiozaki, S.; Kase, H.	Adenosine A(2A) receptor-mediated modulation of GABA and glutamate release in the output regions of the basal ganglia in a rodent model of Parkinson's disease	2004	127	223-231	Neuroscience
Ohoyama, K.; Yamamura, S.; Hamaguchi, T.; Nakagawa, M.; Motomura, E.; Shiroyama, T.; Tanii, H.;	Effect of novel atypical antipsychotic, blonanserin, on extracellular neurotransmitter level in rat prefrontal cortex	2011	653	47-57	Eur J Pharmacol

GABA					
**Authors**	**Title**	**Year**	**Vol**	**Pages**	**Journal**
Okada, M.					
Ohtaki, K.; Matsubara, K.; Fujimaru, S.; Shimizu, K.; Awaya, T.; Suno, M.; Chiba, K.; Hayase, N.; Shiono, H.	Cefoselis, a beta-lactam antibiotic, easily penetrates the blood-brain barrier and causes seizure independently by glutamate release	2004	111	1523-1535	J Neural Transm (Vienna)
Ohtani, N.; Ohta, M.; Sugano, T.	Microdialysis study of modification of hypothalamic neurotransmitters in streptozotocin-diabetic rats	1997	69	1622-1628	J Neurochem
Ohtani, N.; Sugano, T.; Ohta, M.	Alterations in monoamines and GABA in the ventromedial and paraventricular nuclei of the hypothalamus following cold exposure: a reduction in noradrenaline induces hyperphagia	1999	842	6-14	Brain Res
Ojanen, S. P.; Palmen, M.; Hyytia, P.; Kiianmaa, K.	Extracellular glutamate and GABA in the ventral tegmental area of alcohol-preferring AA and alcohol-avoiding ANA rats treated repeatedly with morphine	2007	559	38-45	Eur J Pharmacol
Okada, M.; Zhu, G.; Yoshida, S.; Hirose, S.; Kaneko, S.	Protein kinase associated with gating and closing transmission mechanisms in temporoammonic pathway	2004	47	485-504	Neuropharmacology
Okuda, N.; Kohara, K.; Mikami, H.; Moriguchi, A.; Yamada, K.; Higaki, J.; Ogihara, T.	Effect of propranolol on central neurotransmitter release in Wistar rats analysed by brain microdialysis	1999	26	220-224	Clin Exp Pharmacol Physiol
Oliva, P.; Berrino, L.; de Novellis, V.; Palazzo, E.; Marabese, I.; Siniscalco, D.; Scafuro, M.; Mariani, L.; Rossi, F.; Maione, S.	Role of periaqueductal grey prostaglandin receptors in formalin-induced hyperalgesia	2006	530	40-47	Eur J Pharmacol
Ooboshi, H.; Sadoshima, S.; Yao, H.; Ibayashi, S.; Matsumoto, T.; Uchimura, H.; Fujishima, M.	Ischemia-induced release of amino acids in the hippocampus of aged hypertensive rats	1995	15	227-234	J Cereb Blood Flow Metab
Opacka-Juffry, J.; Ashworth, S.; Ahier, R. G.; Hume, S. P.	Modulatory effects of L-DOPA on D2 dopamine receptors in rat striatum, measured using in vivo microdialysis and PET	1998	105	349-364	J Neural Transm (Vienna)
Oreiro-Garcia, M. T.; Vazquez-Illanes, M. D.; Sierra-Paredes, G.; Sierra-Marcuno, G.	Changes in extracellular amino acid concentrations in the rat hippocampus after in vivo actin depolymerization with latrunculin A	2007	50	734-740	Neurochem Int
Orwar, O.; Li, X.; Andine, P.; Bergstrom, C. M.; Hagberg, H.; Folestad, S.; Sandberg, M.	Increased intra- and extracellular concentrations of gamma-glutamylglutamate and related dipeptides in the ischemic rat striatum: involvement of glutamyl transpeptidase	1994	63	1371-1376	J Neurochem
Osborne, P. G.; Connor, W. T.; Beck,	Acute versus chronic haloperidol: relationship between tolerance to	1994	634	20-30	Brain Res

GABA					
**Authors**	**Title**	**Year**	**Vol**	**Pages**	**Journal**
O.; Ungerstedt, U.	catalepsy and striatal and accumbens dopamine, GABA and acetylcholine release				
Osborne, P. G.; Connor, W. T.; Drew, K. L.; Ungerstedt, U.	An in vivo microdialysis characterization of extracellular dopamine and GABA in dorsolateral striatum of awake freely moving and halothane anaesthetised rats	1990	34	99-105	J Neurosci Methods
Osborne, P. G.; Connor, W. T.; Kehr, J.; Ungerstedt, U.	In vivo characterisation of extracellular dopamine, GABA and acetylcholine from the dorsolateral striatum of awake freely moving rats by chronic microdialysis	1991	37	93-102	J Neurosci Methods
Osborne, P. G.; Hashimoto, M.	Mammalian cerebral metabolism and amino acid neurotransmission during hibernation	2008	106	1888-1899	J Neurochem
Osborne, P. G.; Hu, Y.; Covey, D. N.; Barnes, B. N.; Katz, Z.; Drew, K. L.	Determination of striatal extracellular gamma-aminobutyric acid in non-hibernating and hibernating arctic ground squirrels using quantitative microdialysis	1999	839	1-6	Brain Res
Oshinsky, M. L.; Luo, J.	Neurochemistry of trigeminal activation in an animal model of migraine	2006	46 Suppl 1	S39-S44	Headache
Oshinsky, M. L.; Murphy, A. L.; Hekierski, H., Jr.; Cooper, M.; Simon, B. J.	Noninvasive vagus nerve stimulation as treatment for trigeminal allodynia	2014	155	1037-1042	Pain
Oskarsson, A.; Ljungberg, T.; Stahle, L.	Behavioral and neurochemical effects after combined perinatal treatment of rats with lead and disulfiram	1986	8	591-599	Neurobehav Toxicol Teratol
Ossanai, L. T.; Lourenco, G. A.; Nencioni, A. L.; Lebrun, I.; Yamanouye, N.; Dorce, V. A.	Effects of a toxin isolated from Tityus bahiensis scorpion venom on the hippocampus of rats	2012	91	230-236	Life Sci
Palazzo, E.; Marabese, I.; Soukupova, M.; Luongo, L.; Boccella, S.; Giordano, C.; de Novellis, V.; Rossi, F.; Maione, S.	Metabotropic glutamate receptor subtype 8 in the amygdala modulates thermal threshold, neurotransmitter release, and rostral ventromedial medulla cell activity in inflammatory pain	2011	31	4687-4697	J Neurosci
Paolone, G.; Brugnoli, A.; Arcuri, L.; Mercatelli, D.; Morari, M.	Eltoprazine prevents levodopa-induced dyskinesias by reducing striatal glutamate and direct pathway activity	2015	30	1728-1738	Mov Disord
Paredes, D. A.; Cartford, M. C.; Catlow, B. J.; Samec, A.; Avilas, M.; George, A.; Schlunck, A.; Small, B.; Bickford, P. C.	Neurotransmitter release during delay eyeblink classical conditioning: role of norepinephrine in consolidation and effect of age	2009	92	267-282	Neurobiol Learn Mem
Parent, M. B.; Master, S.; Kashlub, S.; Baker, G. B.	Effects of the antidepressant/antipanic drug phenelzine and its putative metabolite phenylethylidenehydrazine on extracellular	2002	63	57-64	Biochem Pharmacol

GABA					
Authors	Title	Year	Vol	Pages	Journal
	gamma-aminobutyric acid levels in the striatum				
Parrot, S.; Sauvinet, V.; Riban, V.; Depaulis, A.; Renaud, B.; Denoroy, L.	High temporal resolution for in vivo monitoring of neurotransmitters in awake epileptic rats using brain microdialysis and capillary electrophoresis with laser-induced fluorescence detection	2004	140	29-38	J Neurosci Methods
Parsell, D. M.; Laimon-Thomson, E.; Northcott, C. A.	GABA and glutamate in the paraventricular nucleus during the onset of renal wrap rat hypertension	2011	21(4)	258	Clin Auton Res
Pasumarthi, R. K.; Fadel, J.	Stimulation of lateral hypothalamic glutamate and acetylcholine efflux by nicotine: implications for mechanisms of nicotine-induced activation of orexin neurons	2010	113	1023-1035	J Neurochem
Patel, D. R.; Young, A. M.; Croucher, M. J.	Presynaptic alpha-amino-3-hydroxy-5-methyl-4-isoxazole propionate receptor-mediated stimulation of glutamate and GABA release in the rat striatum in vivo: a dual-label microdialysis study	2001	102	101-111	Neuroscience
Patel, D.; Bohlke, M.; Phattanarudee, S.; Kabadi, S.; Maher, T. J.; Ally, A.	Cardiovascular responses and neurotransmitter changes during blockade of angiotensin II receptors within the ventrolateral medulla	2008	60	340-348	Neurosci Res
Paulsen, R. E.; Fonnum, F.	Role of glial cells for the basal and Ca2+-dependent K+-evoked release of transmitter amino acids investigated by microdialysis	1989	52	1823-1829	J Neurochem
Pellegrini-Giampietro, D. E.; Peruginelli, F.; Meli, E.; Cozzi, A.; Albani-Torregrossa, S.; Pellicciari, R.; Moroni, F.	Protection with metabotropic glutamate 1 receptor antagonists in models of ischemic neuronal death: time-course and mechanisms	1999	38	1607-1619	Neuropharmacology
Pena, F.; Tapia, R.	Relationships among seizures, extracellular amino acid changes, and neurodegeneration induced by 4-aminopyridine in rat hippocampus: a microdialysis and electroencephalographic study	1999	72	2006-2014	J Neurochem
Pena, F.; Tapia, R.	Seizures and neurodegeneration induced by 4-aminopyridine in rat hippocampus in vivo: role of glutamate- and GABA-mediated neurotransmission and of ion channels	2000	101	547-561	Neuroscience
Peng, J. F.; Wu, Z. T.; Wang, Y. K.; Yuan, W. J.; Sun, T.; Ni, X.; Su, D. F.; Wang, W.; Xu, M. J.; Wang, W. Z.	GABAergic mechanism in the rostral ventrolateral medulla contributes to the hypotension of moxonidine	2011	89	473-481	Cardiovasc Res
Perez-Pinzon, M. A.; Nilsson, G. E.; Lutz, P. L.	Relationship between ion gradients and neurotransmitter release in the newborn rat striatum during anoxia	1993	602	228-233	Brain Res
Perry, V. L.; Young, R. S.; Aquila, W. J.;	Effect of experimental Escherichia coli meningitis on	1993	34	187-	Pediatr Res

GABA					
**Authors**	**Title**	**Year**	**Vol**	**Pages**	**Journal**
During, M. J.	concentrations of excitatory and inhibitory amino acids in the rabbit brain: in vivo microdialysis study			191	
Petkova-Kirova, P.; Rakovska, A.; Della Corte, L.; Zaekova, G.; Radomirov, R.; Mayer, A.	Neurotensin modulation of acetylcholine, GABA, and aspartate release from rat prefrontal cortex studied in vivo with microdialysis	2008	77	129-135	Brain Res Bull
Petrie, K. A.; Schmidt, D.; Bubser, M.; Fadel, J.; Carraway, R. E.; Deutch, A. Y.	Neurotensin activates GABAergic interneurons in the prefrontal cortex	2005	25	1629-1936	J Neurosci
Phattanarudee, S.; Towiwat, P.; Maher, T. J.; Ally, A.	Effects of medullary administration of a nitric oxide precursor on cardiovascular responses and neurotransmission during static exercise following ischemic stroke	2013	91	510-520	Can J Physiol Pharmacol
Phillis, J. W.; Song, D.; Guyot, L. L.; Regan, M. H.	Lactate reduces amino acid release and fuels recovery of function in the ischemic brain	1999	272	195-198	Neurosci Lett
Piepponen, T. P.; Kiianmaa, K.; Ahtee, L.	Effects of ethanol on the accumbal output of dopamine, GABA and glutamate in alcohol-tolerant and alcohol-nontolerant rats	2002	74	21-30	Pharmacol Biochem Behav
Piepponen, T. P.; Skujins, A.	Rapid and sensitive step gradient assays of glutamate, glycine, taurine and gamma-aminobutyric acid by high-performance liquid chromatography-fluorescence detection with o-phthalaldehyde-mercaptoethanol derivatization with an emphasis on microdialysis samples	2001	757	277-283	J Chromatogr B Biomed Sci Appl
Pierard, C.; Lagarde, D.; Barrere, B.; Duret, P.; Cordeiro, C.; Guezennec, C. Y.; Peres, M.	Effects of a vigilance enhancing drug, modafinil, on rat brain cortex amino acids: A microdialysis study	1997	25	51-54	Med Sci Res
Pierard, C.; Peres, M.; Satabin, P.; Guezennec, C. Y.; Lagarde, D.	Effects of GABA-transaminase inhibition on brain metabolism and amino-acid compartmentation: an in vivo study by 2D 1H-NMR spectroscopy coupled with microdialysis	1999	127	321-327	Exp Brain Res
Pistis, M.; Ferraro, L.; Pira, L.; Flore, G.; Tanganelli, S.; Gessa, G. L.; Devoto, P.	Delta(9)-tetrahydrocannabinol decreases extracellular GABA and increases extracellular glutamate and dopamine levels in the rat prefrontal cortex: an in vivo microdialysis study	2002	948	155-158	Brain Res
Portelli, J.; Aourz, N.; De Bundel, D.; Meurs, A.; Smolders, I.; Michotte, Y.; Clinckers, R.	Intrastrain differences in seizure susceptibility, pharmacological response and basal neurochemistry of Wistar rats	2009	87	234-246	Epilepsy Res
Portelli, J.; Smolders, I.	Intracerebral microdialysis in the study of limbic seizure	2013	75	321-	Microdialy Tech Neurosci

GABA					
**Authors**	**Title**	**Year**	**Vol**	**Pages**	**Journal**
	mechanisms and antiepileptic drug action using freely moving rats			337	
Qi, J.; Han, W. Y.; Yang, J. Y.; Wang, L. H.; Dong, Y. X.; Wang, F.; Song, M.; Wu, C. F.	Oxytocin regulates changes of extracellular glutamate and GABA levels induced by methamphetamine in the mouse brain	2012	17	758-769	Addict Biol
Qian, T.; Chen, R.; Nakamura, M.; Furukawa, T.; Kumada, T.; Akita, T.; Kilb, W.; Luhmann, H. J.; Nakahara, D.; Fukuda, A.	Activity-dependent endogenous taurine release facilitates excitatory neurotransmission in the neocortical marginal zone of neonatal rats	2014	8	33	Front Cell Neurosci
Qu, Y.; Arckens, L.; Vandenbussche, E.; Geeraerts, S.; Vandesande, F.	Simultaneous determination of total and extracellular concentrations of the amino acid neurotransmitters in cat visual cortex by microbore liquid chromatography and electrochemical detection	1998	798	19-26	J Chromatogr A
Qu, Y.; Arckens, L.; Vandesande, F.; Vandenbussche, E.	Sampling extracellular aspartate, glutamate and gamma-aminobutyric acid in striate cortex of awake cat by in vivo microdialysis: surgical and methodological aspects	2000	866	116-127	Brain Res
Qu, Y.; Li, Y.; Vandenbussche, E.; Vandesande, F.; Arckens, L.	In vivo microdialysis in the visual cortex of awake cat. II: sample analysis by microbore HPLC-electrochemical detection and capillary electrophoresis-laser-induced fluorescence detection	2001	7	45-51	Brain Res Brain Res Protoc
Qu, Y.; Saint Marie, R. L.; Breier, M. R.; Ko, D.; Stouffer, D.; Parsons, L. H.; Swerdlow, N. R.	Neural basis for a heritable phenotype: Differences in the effects of apomorphine on startle gating and ventral pallidal GABA efflux in male Sprague-Dawley and Long-Evans rats	2009	207	271-280	Psychopharmacology
Qu, Y.; Swerdlow, N. R.; Weber, M.; Stouffer, D.; Parsons, L. H.	Quinelorane, a dopamine D3/D2 receptor agonist, reduces prepulse inhibition of startle and ventral pallidal GABA efflux: Time course studies	2008	90	686-690	Pharmacol Biochem Behav
Quertemont, E.; Dahchour, A.; Ward, R. J.; De Witte, P.	Ethanol induces taurine release in the amygdala: An in vivo microdialysis study	1999	4	47-54	Addict Biol
Qume, M.; Whitton, P. S.; Fowler, L. J.	The effect of chronic treatment with the GABA transaminase inhibitors gamma-vinyl-GABA and ethanolamine-O-sulphate on the in vivo release of GABA from rat hippocampus	1995	64	2256-2261	J Neurochem
Qureshi, A. I.; Ali, Z.; Suri, M. F.; Shuaib, A.; Baker, G.; Todd, K.; Guterman, L. R.; Hopkins, L. N.	Extracellular glutamate and other amino acids in experimental intracerebral hemorrhage: an in vivo microdialysis study	2003	31	1482-1489	Crit Care Med
Rada, P.; Mendialdua, A.; Hernandez,	Extracellular glutamate increases in the lateral hypothalamus	2003	117	222-	Behav Neurosci

GABA					
Authors	Title	Year	Vol	Pages	Journal
L.; Hoebel, B. G.	during meal initiation, and GABA peaks during satiation: microdialysis measurements every 30 s			227	
Raedt, R.; Clinckers, R.; Mollet, L.; Vonck, K.; El Tahry, R.; Wyckhuys, T.; De Herdt, V.; Carrette, E.; Wadman, W.; Michotte, Y.; Smolders, I.; Boon, P.; Meurs, A.	Increased hippocampal noradrenaline is a biomarker for efficacy of vagus nerve stimulation in a limbic seizure model	2011	117	461-469	J Neurochem
Rajarao, S. J.; Platt, B.; Sukoff, S. J.; Lin, Q.; Bender, C. N.; Nieuwenhuijsen, B. W.; Ring, R. H.; Schechter, L. E.; Rosenzweig-Lipson, S.; Beyer, C. E.	Anxiolytic-like activity of the non-selective galanin receptor agonist, galnon	2007	41	307-320	Neuropeptides
Rakovska, A.; Giovannini, M. G.; Corte, L. D.; Kalfin, R.; Bianchi, L.; Pepeu, G.	Neurotensin modulation of acetylcholine and GABA release from the rat hippocampus: An in vivo microdialysis study	1998	33	335-340	Neurochem Int
Rao, V. L.; Audet, R. M.; Butterworth, R. F.	Selective alterations of extracellular brain amino acids in relation to function in experimental portal-systemic encephalopathy: results of an in vivo microdialysis study	1995	65	1221-1228	J Neurochem
Ravindran, J.; Shuaib, A.; Ijaz, S.; Galazka, P.; Waqar, T.; Ishaqzay, R.; Miyashita, H.; Liu, L.	High extracellular GABA levels in hippocampus--as a mechanism of neuronal protection in cerebral ischemia in adrenalectomized gerbils	1994	176	209-211	Neurosci Lett
Rea, K.; Cremers, T. I.; Westerink, B. H.	HPLC conditions are critical for the detection of GABA by microdialysis	2005	94	672-679	J Neurochem
Rea, K.; Lang, Y.; Finn, D. P.	Alterations in extracellular levels of gamma-aminobutyric acid in the rat basolateral amygdala and periaqueductal gray during conditioned fear, persistent pain and fear-conditioned analgesia	2009	10	1088-1098	J Pain
Reagan, L. P.; Reznikov, L. R.; Evans, A. N.; Gabriel, C.; Mocaer, E.; Fadel, J. R.	The antidepressant agomelatine inhibits stress-mediated changes in amino acid efflux in the rat hippocampus and amygdala	2012	1446	91-98	Brain Res
Reinhoud, N. J.; Brouwer, H. J.; van Heerwaarden, L. M.; Korte-Bouws, G. A.	Analysis of glutamate, GABA, noradrenaline, dopamine, serotonin, and metabolites using microbore UHPLC with electrochemical detection	2013	4	888-894	ACS Chem Neurosci
Reisi, P.; Alaei, H.; Babri, S.; Sharifi, M. R.; Mohaddes, G.; Soleimannejad, E.	Determination of the extracellular basal levels of glutamate and GABA at dentate gyrus of streptozotocin-induced diabetic rats	2009	16	63-66	Pathophysiology
Reisi, P.; Alaei, H.; Babri, S.; Sharifi, M.	Effects of treadmill running on extracellular basal levels of	2010	15	172-	J Res Med Sci

GABA					
**Authors**	**Title**	**Year**	**Vol**	**Pages**	**Journal**
R.; Mohaddes, G.; Soleimannejad, E.; Rashidi, B.	glutamate and GABA at dentate gyrus of streptozotocin-induced diabetic rats			174	
Renno, W. M.; Alkhalaf, M.; Mousa, A.; Kanaan, R. A.	A comparative study of excitatory and inhibitory amino acids in three different brainstem nuclei	2008	33	150-159	Neurochem Res
Renno, W. M.; Beitz, A. J.	Peripheral inflammation is associated with decreased veratridine-induced release of GABA in the rat ventrocaudal periaqueductal gray: microdialysis study	1999	163	105-110	J Neurol Sci
Renno, W. M.; Mullet, M. A.; Williams, F. G.; Beitz, A. J.	Construction of 1 mm microdialysis probe for amino acids dialysis in rats	1998	79	217-228	J Neurosci Methods
Renno, W. M.; Mullett, M. A.; Beitz, A. J.	Systemic morphine reduces GABA release in the lateral but not the medial portion of the midbrain periaqueductal gray of the rat	1992	594	221-232	Brain Res
Reynolds Jr, N. C.; Lin, W.; Cameron, C. M.; Roerig, D. L.	Extracellular perfusion of rat brain nuclei using microdialysis: A method for studying differential neurotransmitter release in response to neurotoxins	1999	4	124-131	Brain Res
Reynolds Jr, N. C.; Lin, W.; Meyer Cameron, C.; Roerig, D. L.	Differential responses of extracellular GABA to intrastriatal perfusions of 3-nitropropionic acid and quinolinic acid in the rat	1997	778	140-149	Brain Res
Reznikov, L. R.; Reagan, L. P.; Fadel, J. R.	Effects of acute and repeated restraint stress on GABA efflux in the rat basolateral and central amygdala	2009	1256	61-68	Brain Res
Richards, D. A.; Bowery, N. G.	Comparative effects of the GABA uptake inhibitors, tiagabine and NNC-711, on extracellular GABA levels in the rat ventrolateral thalamus	1996	21	135-140	Neurochem Res
Richards, D. A.; Lemos, T.; Whitton, P. S.; Bowery, N. G.	Extracellular GABA in the ventrolateral thalamus of rats exhibiting spontaneous absence epilepsy: a microdialysis study	1995	65	1674-1680	J Neurochem
Richards, D. A.; Morrone, L. A.; Bowery, N. G.	Hippocampal extracellular amino acids and EEG spectral analysis in a genetic rat model of absence epilepsy	2000	39	2433-2441	Neuropharmacology
Richter, D. W.; Schmidt-Garcon, P.; Pierrefiche, O.; Bischoff, A. M.; Lalley, P. M.	Neurotransmitters and neuromodulators controlling the hypoxic respiratory response in anaesthetized cats	1999	514 (Pt 2)	567-578	J Physiol
Rimondini, R.; Connor, W. T.; Ferre, S.; Sillard, R.; Agerberth, B.; Mutt, V.; Ungerstedt, U.; Fuxe, K.	PEC-60 increases dopamine but not GABA release in the dorsolateral neostriatum of the halothane anaesthetized rat. An in vivo microdialysis study	1994	177	53-57	Neurosci Lett
Rimondini, R.; Connor, W. T.; Sillard, R.; Mutt, V.; Ungerstedt, U.; Fuxe, K.	The secretory trypsin inhibitor like-peptide, PEC-60 increases dopamine D2 receptor agonist induced inhibition of GABA release	1996	61	111-117	Regul Pept

GABA					
**Authors**	**Title**	**Year**	**Vol**	**Pages**	**Journal**
	in the dorsolateral neostriatum of the awake freely moving rat. An in vivo microdialysis study				
Ritz, M. F.; Schmidt, P.; Mendelowitsch, A.	17beta-estradiol effect on the extracellular concentration of amino acids in the glutamate excitotoxicity model in the rat	2002	27	1677-1683	Neurochem Res
Rivas-Arancibia, S.; Dorado-Martinez, C.; Colin-Barenque, L.; Kendrick, K. M.; De La Riva, C.; Guevara-Guzman, R.	Effect of acute ozone exposure on locomotor behavior and striatal function	2003	74	891-900	Pharmacol Biochem Behav
Roberto, M.; Cruz, M. T.; Gilpin, N. W.; Sabino, V.; Schweitzer, P.; Bajo, M.; Cottone, P.; Madamba, S. G.; Stouffer, D. G.; Zorrilla, E. P.; Koob, G. F.; Siggins, G. R.; Parsons, L. H.	Corticotropin releasing factor-induced amygdala gamma-aminobutyric Acid release plays a key role in alcohol dependence	2010	67	831-839	Biol Psychiatry
Roberto, M.; Madamba, S. G.; Stouffer, D. G.; Parsons, L. H.; Siggins, G. R.	Increased GABA release in the central amygdala of ethanol-dependent rats	2004	24	10159-10166	J Neurosci
Robertson, R. G.; Graham, W. C.; Sambrook, M. A.; Crossman, A. R.	Further investigations into the pathophysiology of MPTP-induced parkinsonism in the primate: an intracerebral microdialysis study of gamma-aminobutyric acid in the lateral segment of the globus pallidus	1991	563	278-280	Brain Res
Robinson, J. E.	Gamma amino-butyric acid and the control of GnRH secretion in sheep	1995	49	221-230	J Reprod Fertil Suppl
Robinson, J. E.; Kendrick, K. M.	Inhibition of luteinizing hormone secretion in the ewe by progesterone: associated changes in the release of gamma-aminobutyric Acid and noradrenaline in the preoptic area as measured by intracranial microdialysis	1992	4	231-236	J Neuroendocrinol
Robinson, J. E.; Kendrick, K. M.; Lambart, C. E.	Changes in the release of gamma-aminobutyric Acid and catecholamines in the preoptic/septal area prior to and during the preovulatory surge of luteinizing hormone in the ewe	1991	3	393-399	J Neuroendocrinol
Rodriguez Diaz, M.; Alonso, T. J.; Perdomo Diaz, J.; Gonzalez Hernandez, T.; Castro Fuentes, R.; Sabate, M.; Garcia Dopico, J.	Glial regulation of nonsynaptic extracellular glutamate in the substantia nigra	2005	49	134-142	Glia
Rodriguez, M. J.; Robledo, P.; Andrade, C.; Mahy, N.	In vivo co-ordinated interactions between inhibitory systems to control glutamate-mediated hippocampal excitability	2005	95	651-661	J Neurochem

GABA					
**Authors**	**Title**	**Year**	**Vol**	**Pages**	**Journal**
Rosales, M. G.; Martinez-Fong, D.; Morales, R.; Nunez, A.; Flores, G.; Gongora-Alfaro, J. L.; Floran, B.; Aceves, J.	Reciprocal interaction between glutamate and dopamine in the pars reticulata of the rat substantia nigra: a microdialysis study	1997	80	803-810	Neuroscience
Rosi, S.; Giovannini, M. G.; Lestage, P. J.; Munoz, C.; Corte, L. D.; Pepeu, G.	S 18986, a positive modulator of AMPA receptors with cognition-enhancing properties, increases ACh release in the hippocampus of young and aged rat	2004	361	120-123	Neurosci Lett
Rowley, H. L.; Marsden, C. A.; Martin, K. F.	Generalised seizure-induced changes in rat hippocampal glutamate but not GABA release are potentiated by repeated seizures	1997	234	143-146	Neurosci Lett
Rowley, H. L.; Marsden, C. A.; Martin, K. F.	Differential effects of phenytoin and sodium valproate on seizure-induced changes in gamma-aminobutyric acid and glutamate release in vivo	1995	294	541-546	Eur J Pharmacol
Rowley, H. L.; Martin, K. F.; Marsden, C. A.	Determination of in vivo amino acid neurotransmitters by high-performance liquid chromatography with o-phthalaldehyde-sulphite derivatisation	1995	57	93-99	J Neurosci Methods
Rowley, H. L.; Martin, K. F.; Marsden, C. A.	Decreased GABA release following tonic-clonic seizures is associated with an increase in extracellular glutamate in rat hippocampus in vivo	1995	68	415-422	Neuroscience
Ruda-Kucerova, J.; Amchova, P.; Havlickova, T.; Jerabek, P.; Babinska, Z.; Kacer, P.; Syslova, K.; Sulcova, A.; Sustkova-Fiserova, M.	Reward related neurotransmitter changes in a model of depression: An in vivo microdialysis study	2015	16	521-535	World J Biol Psychiatry
Runnerstam, M.; Bao, F.; Huang, Y.; Shi, J.; Gutierrez, E.; Hamberger, A.; Hansson, H. A.; Viano, D.; Haglid, K.	A new model for diffuse brain injury by rotational acceleration: II. Effects on extracellular glutamate, intracranial pressure, and neuronal apoptosis	2001	18	259-273	J Neurotrauma
Saito, H.; Matsumoto, M.; Togashi, H.; Yoshioka, M.	Ischemia stimulates cortical 5-hydroxytryptamine efflux in rat brain: An in vivo microdialysis study	1994	10	91-97	Biog Amines
Salamone, A.; Mura, E.; Zappettini, S.; Grilli, M.; Olivero, G.; Preda, S.; Govoni, S.; Marchi, M.	Inhibitory effects of beta-amyloid on the nicotinic receptors which stimulate glutamate release in rat hippocampus: the glial contribution	2014	723	314-321	Eur J Pharmacol
Salazar, P.; Tapia, R.	Allopregnanolone potentiates the glutamate-mediated seizures induced by 4-aminopyridine in rat hippocampus in vivo	2012	37	596-603	Neurochem Res

GABA					
**Authors**	**Title**	**Year**	**Vol**	**Pages**	**Journal**
Salazar, P.; Tapia, R.	Epilepsy and hippocampal neurodegeneration induced by glutamate decarboxylase inhibitors in awake rats	2015	116	27-33	Epilepsy Res
Sale, A.; Maya Vetencourt, J. F.; Medini, P.; Cenni, M. C.; Baroncelli, L.; De Pasquale, R.; Maffei, L.	Environmental enrichment in adulthood promotes amblyopia recovery through a reduction of intracortical inhibition	2007	10	679-681	Nat Neurosci
Saleh, T. M.; Connell, B. J.; Legge, C.; Cribb, A. E.	Estrogen attenuates neuronal excitability in the insular cortex following middle cerebral artery occlusion	2004	1018	119-129	Brain Res
Saleh, T. M.; Connell, B. J.; McQuaid, T.; Cribb, A. E.	Estrogen-induced neurochemical and electrophysiological changes in the parabrachial nucleus of the male rat	2003	990	58-65	Brain Res
Samoudi, G.; Nissbrandt, H.; Dutia, M. B.; Bergquist, F.	Noisy galvanic vestibular stimulation promotes GABA release in the substantia nigra and improves locomotion in hemiparkinsonian rats	2012	7	e29308	PLoS One
Samuel, D.; Pisano, P.; Forni, C.; Nieoullon, A.; Goff, L. K. L.	Involvement of the glutamatergic metabotropic receptors in the regulation of glutamate uptake and extracellular excitatory amino acid levels in the striatum of chloral hydrate-anesthetized rats	1996	739	156-162	Brain Research
Sandberg, M.; Butcher, S. P.; Hagberg, H.	Extracellular overflow of neuroactive amino acids during severe insulin-induced hypoglycemia: in vivo dialysis of the rat hippocampus	1986	47	178-184	J Neurochem
Santana Gomez, C.	Evaluation of the hippocampal release of gaba and glutamate as result of status epilepticus induced by lithium-pilocarpine administration in rats	2014	14	148	Epilepsy Curr
Santana-Gomez, C. E.; Alcantara-Gonzalez, D.; Luna-Munguia, H.; Banuelos-Cabrera, I.; Magdaleno-Madrigal, V.; Fernandez-Mas, R.; Besio, W.; Rocha, L.	Transcranial focal electrical stimulation reduces the convulsive expression and amino acid release in the hippocampus during pilocarpine-induced status epilepticus in rats	2015	49	33-39	Epilepsy Behav
Santos-Fandila, A.; Zafra-Gomez, A.; Barranco, A.; Navalon, A.; Rueda, R.; Ramirez, M.	Quantitative determination of neurotransmitters, metabolites and derivates in microdialysates by UHPLC-tandem mass spectrometry	2013	114	79-89	Talanta
Saul; skaia, N. B.; Gorbachevskaia, A. I.	[Participation of the hippocampus in regulation of GABA release in the nucleus accumbens during emotional conditioning]	1998	84	443-449	Ross Fiziol Zh Im I M Sechenova
Saul; skaya, N. B.; Gorbachevskaya, A. I.	The role of the hippocampal formation in controlling GABA release in the nucleus accumbens during an emotional conditioned	1999	29	461-466	Neurosci Behav Physiol

GABA					
**Authors**	**Title**	**Year**	**Vol**	**Pages**	**Journal**
	response				
Saul; skaya, N. B.; Marsden, C. A.	gamma-Aminobutyric acid levels in the intercellular space in the nucleus accumbens of the rat brain during a nociceptive conditioned response	1998	28	60-64	Neurosci Behav Physiol
Saulskaya, N.; Marsden, C. A.	Extracellular glutamate in the nucleus accumbens during a conditioned emotional response in the rat	1995	698	114-120	Brain Res
Sayin, U.; Timmerman, W.; Westerink, B. H.	The significance of extracellular GABA in the substantia nigra of the rat during seizures and anticonvulsant treatments	1995	669	67-72	Brain Res
Schaefer, F.; Vogel, M.; Kerkhoff, G.; Woitzik, J.; Daschner, M.; Mehls, O.	Experimental uremia affects hypothalamic amino acid neurotransmitter milieu	2001	12	1218-1227	J Am Soc Nephrol
Schallier, A.; Vermoesen, K.; Loyens, E.; Van Liefferinge, J.; Michotte, Y.; Smolders, I.; Massie, A.	L-Theanine intake increases threshold for limbic seizures but decreases threshold for generalized seizures	2013	16	78-82	Nutr Neurosci
Schechter, L. E.; Lin, Q.; Smith, D. L.; Zhang, G.; Shan, Q.; Platt, B.; Brandt, M. R.; Dawson, L. A.; Cole, D.; Bernotas, R.; Robichaud, A.; Rosenzweig-Lipson, S.; Beyer, C. E.	Neuropharmacological profile of novel and selective 5-HT6 receptor agonists: WAY-181187 and WAY-208466	2008	33	1323-1335	Neuropsychopharmacology
Schepers, R. J.; Mahoney, J. L.; Zapata, A.; Chefer, V.; Shippenberg, T. S.	The effects of local perfusion of DAMGO on extracellular GABA and glutamate concentrations in the rostral ventromedial medulla	2008	104	806-817	J Neurochem
Schoenfeld, T. J.; Rada, P.; Pieruzzini, P. R.; Hsueh, B.; Gould, E.	Physical exercise prevents stress-induced activation of granule neurons and enhances local inhibitory mechanisms in the dentate gyrus	2013	33	7770-7777	J Neurosci
Schroeder, J. A.; Schneider, J. S.	GABA-opioid interactions in the globus pallidus: [D-Ala2]-Met-enkephalinamide attenuates potassium-evoked GABA release after nigrostriatal lesion	2002	82	666-673	J Neurochem
Schubert, G. A.; Poli, S.; Mendelowitsch, A.; Schilling, L.; Thome, C.	Hypothermia reduces early hypoperfusion and metabolic alterations during the acute phase of massive subarachnoid hemorrhage: a laser-Doppler-flowmetry and microdialysis study in rats	2008	25	539-548	J Neurotrauma
Schultz, K. N.; von Esenwein, S. A.; Hu, M.; Bennett, A. L.; Kennedy, R. T.;	Viral vector-mediated overexpression of estrogen receptor-alpha in striatum enhances the estradiol-induced motor activity in female	2009	29	1897-1903	J Neurosci

GABA					
**Authors**	**Title**	**Year**	**Vol**	**Pages**	**Journal**
Musatov, S.; Toran-Allerand, C. D.; Kaplitt, M. G.; Young, L. J.; Becker, J. B.	rats and estradiol-modulated GABA release				
See, R. E.; Berglind, W. J.	Decreased pallidal GABA following reverse microdialysis with clozapine, but not haloperidol	2001	12	3655-3658	Neuroreport
See, R. E.; Berglind, W. J.; Krentz, L.; Meshul, C. K.	Convergent evidence from microdialysis and presynaptic immunolabeling for the regulation of gamma-aminobutyric acid release in the globus pallidus following acute clozapine or haloperidol administration in rats	2002	82	172-180	J Neurochem
See, R. E.; Chapman, M. A.	Chronic haloperidol, but not clozapine, produces altered oral movements and increased extracellular glutamate in rats	1994	263	269-276	Eur J Pharmacol
See, R. E.; Chapman, M. A.; Klitenick, M. A.	Chronic neuroleptic administration decreases extracellular GABA in the nucleus accumbens but not in the caudate-putamen of rats	1992	588	177-180	Brain Res
See, R. E.; Lynch, A. M.	Chronic haloperidol potentiates stimulated glutamate release in caudate putamen, but not prefrontal cortex	1995	6	1795-1798	Neuroreport
Segovia, G.; Mora, F.	Effects of the metabotropic glutamate receptor agonist, ACPD, on the extracellular concentrations of GABA and acetylcholine in the prefrontal cortex of the rat during the normal process of aging	2005	65	11-16	Brain Res Bull
Segovia, G.; Mora, F.	Dopamine and GABA increases produced by activation of glutamate receptors in the nucleus accumbens are decreased during aging	2005	26	91-101	Neurobiol Aging
Segovia, G.; Mora, F.	Involvement of NMDA and AMPA/kainate receptors in the effects of endogenous glutamate on extracellular concentrations of dopamine and GABA in the nucleus accumbens of the awake rat	2001	54	153-157	Brain Res Bull
Segovia, G.; Mora, F.	Role of nitric oxide in modulating the release of dopamine, glutamate, and GABA in striatum of the freely moving rat	1998	45	275-279	Brain Res Bull
Segovia, G.; Yague, A. G.; Garcia-Verdugo, J. M.; Mora, F.	Environmental enrichment promotes neurogenesis and changes the extracellular concentrations of glutamate and GABA in the hippocampus of aged rats	2006	70	8-14	Brain Res Bull
Segovia, J.; Tossman, U.; Herrera-Marschitz, M.	gamma-Aminobutyric acid release in the globus pallidus in vivo after a 6-hydroxydopamine lesion in the substantia nigra of the rat	1986	70	364-368	Neurosci Lett
Semba, J.; Miyoshi, R.; Kanazawa, M.; Kito, S.	Effect of systemic dexamethasone on extracellular amino acids, GABA and acetylcholine release in rat hippocampus using in vivo	1995	10	17-21	Funct Neurol

GABA					
**Authors**	**Title**	**Year**	**Vol**	**Pages**	**Journal**
	microdialysis				
Semba, J.; Sakai, M.; Miyoshi, R.; Kito, S.	NG-monomethyl-L-arginine, an inhibitor of nitric oxide synthase, increases extracellular GABA in the striatum of the freely moving rat	1995	6	1426-1428	Neuroreport
Shah, A. J.; de Biasi, V.; Taylor, S. G.; Roberts, C.; Hemmati, P.; Munton, R.; West, A.; Routledge, C.; Camilleri, P.	Development of a protocol for the automated analysis of amino acids in brain tissue samples and microdialysates	1999	735	133-140	J Chromatogr B Biomed Sci Appl
Shea, S. D.; Smith, I. M.; McCabe, O. M.; Cronin, M. M.; Walsh, D. M.; Connor, W. T.	Intracerebroventricular Administration of Amyloid beta-protein Oligomers Selectively Increases Dorsal Hippocampal Dialysate Glutamate Levels in the Awake Rat	2008	8	7428-7437	Sensors (Basel)
Shen, E. Y.; Lai, Y. J.	In vivo microdialysis study of excitatory and inhibitory amino acid levels in the hippocampus following penicillin-induced seizures in mature rats	2002	43	313-318	Acta Paediatr Taiwan
Shen, L. M.; Chen, Y. C.	[Changes of inhibitory amino acid in the monkey frontal cortex during a figure recognition guided sequential movement task]	1999	51(6)	681-686	Sheng Li Xue Bao
Sheng, W.; Hang, H. W.; Ruan, D. Y.	In vivo microdialysis study of the relationship between lead-induced impairment of learning and neurotransmitter changes in the hippocampus	2005	20	233-240	Environ Toxicol Pharmacol
Shi, H.; Yang, X.; Zhao, H.; Zhang, S.; Zu, J.; Zhang, W.; Shen, X.; Cui, G.; Hua, F.; Yan, C.	Ranitidine reduced levodopa-induced dyskinesia by remodeling neurochemical changes in hemiparkinsonian model of rats	2015	11	1331-1337	Neuropsychiatr Dis Treat
Shi, L.; Yang, A. C.; Li, J. J.; Meng, D. W.; Jiang, B.; Zhang, J. G.	Favorable modulation in neurotransmitters: effects of chronic anterior thalamic nuclei stimulation observed in epileptic monkeys	2015	265	94-101	Exp Neurol
Shima, K.; Shirotani, T.; Chigasaki, H.	Delayed neuronal damage following focal ischemic injury in stroke-prone spontaneously hypertensive rats	1996	67	24-27	Acta Neurochir Suppl
Shimada, N.; Graf, R.; Rosner, G.; Heiss, W. D.	Ischemia-induced accumulation of extracellular amino acids in cerebral cortex, white matter, and cerebrospinal fluid	1993	60	66-71	J Neurochem
Shimizu-Sasamata, M.	Attenuated neurotransmitter release and spreading depression-like depolarizations after focal ischemia in mutant mice with disrupted type I nitric oxide synthase gene	1998	18(22)	9564-9571	J Neurosci
Shin, R. S.; Anisman, H.; Merali, Z.; McIntyre, D. C.	Amygdala amino acid and monoamine levels in genetically Fast and Slow kindling rat strains during massed amygdala kindling: a microdialysis study	2004	20	185-194	Eur J Neurosci

GABA					
**Authors**	**Title**	**Year**	**Vol**	**Pages**	**Journal**
Shin, R. S.; Anisman, H.; Merali, Z.; McIntyre, D. C.	Changes in extracellular levels of amygdala amino acids in genetically fast and slow kindling rat strains	2002	946	31-42	Brain Res
Shinohara, K.; Hirooka, Y.; Kishi, T.; Sunagawa, K.	Inhibition of no-mediated gaba release by reactive oxygen species in the rostral ventrolateral medulla results in sympathoexcitation in hypertensive rats	2011	124	/	Circulation. Conference: American Heart Association's Scientific Sessions
Shinohara, K.; Hirooka, Y.; Kishi, T.; Sunagawa, K.	Reduction of nitric oxide-mediated gamma-amino butyric acid release in rostral ventrolateral medulla is involved in superoxide-induced sympathoexcitation of hypertensive rats	2012	76	2814-2821	Circ J
Shirokawa, T.; Ogawa, T.	Release of GABA following a single whisker stimulation in the adjacent barrels of rat somatosensory cortex	1994	44 Suppl 2	S119-S123	Jpn J Physiol
Shirokawa, T.; Ogawa, T.	Regulation of basal release of GABA by noradrenaline in the kitten visual cortex	1994	5	2037-2040	Neuroreport
Shirokawa, T.; Ogawa, T.	Release of gamma-aminobutyric acid by visual stimulation in the kitten visual cortex	1992	589	157-160	Brain Res
Shirotani, T.; Shima, K.; Chigasaki, H.	In vivo studies of extracellular metabolites in the striatum after distal middle cerebral artery occlusion in stroke-prone spontaneously hypertensive rats	1995	26	878-884	Stroke
Shou, M.; Smith, A. D.; Shackman, J. G.; Peris, J.; Kennedy, R. T.	In vivo monitoring of amino acids by microdialysis sampling with on-line derivatization by naphthalene-2,3-dicarboxyaldehyde and rapid micellar electrokinetic capillary chromatography	2004	138	189-197	J Neurosci Methods
Shuaib, A.; Ijaz, M. S.; Miyashita, H.; Hussain, S.; Kanthan, R.	GABA and glutamate levels in the substantia nigra reticulata following repetitive cerebral ischemia in gerbils	1997	147	311-315	Exp Neurol
Shuaib, A.; Ijaz, M. S.; Waqar, T.; Voll, C.; Kanthan, R.; Miyashita, H.; Liu, L.	Insulin elevates hippocampal GABA levels during ischemia. This is independent of its hypoglycemic effect	1995	67	809-814	Neuroscience
Shuaib, A.; Ijaz, S.; Miyashita, H.; Mainprize, T.; Kanthan, R.	Progressive decrease in extracellular GABA concentrations in the post-ischemic period in the striatum: a microdialysis study	1994	666	99-103	Brain Res
Sierra-Paredes, G.; Galan-Valiente, J.; Vazquez-Illanes, M. D.; Aguilar-Veiga, E.; Soto-Otero, R.; Mendez-Alvarez, E.; Sierra-Marcuno, G.	Extracellular amino acids in the rat hippocampus during picrotoxin threshold seizures in chronic microdialysis experiments	1998	248	53-56	Neurosci Lett
Sierra-Paredes, G.; Loureiro, A. I.; Wright, L. C.; Sierra-Marcuno, G.;	Effects of eslicarbazepine acetate on acute and chronic latrunculin A-induced seizures and extracellular amino acid levels in the	2014	15	134	BMC Neurosci

GABA					
**Authors**	**Title**	**Year**	**Vol**	**Pages**	**Journal**
Soares-da-Silva, P.	mouse hippocampus				
Sierra-Paredes, G.; Oreiro-Garcia, M. T.; Vazquez-Illanes, M. D.; Sierra-Marcuno, G.	Effect of eslicarbazepine acetate (BIA 2-093) on latrunculin A-induced seizures and extracellular amino acid concentrations in the rat hippocampus	2007	77	36-43	Epilepsy Res
Silva, E.; Quinones, B.; Paez, X.; Hernandez, L.	[Effect of a simple morphine system injection in some aminoacids in the anterior cingulate cortex during acute pain]	2008	49	511-522	Invest Clin
Simon, R. P.; Chen, J.; Graham, S. H.	GM1 ganglioside treatment of focal ischemia: a dose-response and microdialysis study	1993	265	24-29	J Pharmacol Exp Ther
Sizemore, G. M.; Co, C.; Smith, J. E.	Ventral pallidal extracellular fluid levels of dopamine, serotonin, gamma amino butyric acid, and glutamate during cocaine self-administration in rats	2000	150	391-398	Psychopharmacology (Berl)
Skirzewski, M.; Lopez, W.; Mosquera, E.; Betancourt, L.; Catlow, B.; Chiurillo, M.; Loureiro, N.; Hernandez, L.; Rada, P.	Enhanced GABAergic tone in the ventral pallidum: memory of unpleasant experiences?	2011	196	131-146	Neuroscience
Skorzewska, A.; Bidzinski, A.; Hamed, A.; Lehner, M.; Turzynska, D.; Sobolewska, A.; Szyndler, J.; Maciejak, P.; Wislowska-Stanek, A.; Plaznik, A.	The effect of CRF and alpha-helical CRF((9-41)) on rat fear responses and amino acids release in the central nucleus of the amygdala	2009	57	148-156	Neuropharmacology
Skorzewska, A.; Bidzinski, A.; Hamed, A.; Lehner, M.; Turzynska, D.; Sobolewska, A.; Walkowiak, J.; Plaznik, A.	Changes in hippocampal amino acid concentrations after chronic administration of corticosterone	2007	59	763-772	Pharmacol Rep
Slaney, T. R.; Mabrouk, O. S.; Porter-Stransky, K. A.; Aragona, B. J.; Kennedy, R. T.	Chemical gradients within brain extracellular space measured using low flow push-pull perfusion sampling in vivo	2013	4	321-329	ACS Chem Neurosci
Smith, A.; Watson, C. J.; Frantz, K. J.; Eppler, B.; Kennedy, R. T.; Peris, J.	Differential increase in taurine levels by low-dose ethanol in the dorsal and ventral striatum revealed by microdialysis with on-line capillary electrophoresis	2004	28	1028-1038	Alcohol Clin Exp Res
Smith, S. E.; Sharp, T.	An investigation of the origin of extracellular GABA in rat nucleus accumbens measured in vivo by microdialysis	1994	97	161-171	J Neural Transm Gen Sect
Smith, S. L.; Thompson, K. S. J.; Sargent, B. J.; Heal, D. J.	BTS 72664 - A novel CNS drug with potential anticonvulsant, neuroprotective, and antimigraine properties	2001	7	146-171	CNS Drug Reviews

GABA					
**Authors**	**Title**	**Year**	**Vol**	**Pages**	**Journal**
Smith, S.; Sharp, T.	Measurement of GABA in rat brain microdialysates using o-phthaldialdehyde-sulphite derivatization and high-performance liquid chromatography with electrochemical detection	1994	652	228-233	J Chromatogr
Smolders, I.; Bogaert, L.; Ebinger, G.; Michotte, Y.	Muscarinic modulation of striatal dopamine, glutamate, and GABA release, as measured with in vivo microdialysis	1997	68	1942-1948	J Neurochem
Smolders, I.; Gousseau, C.; Marchand, S.; Couet, W.; Ebinger, G.; Michotte, Y.	Convulsant and subconvulsant doses of norfloxacin in the presence and absence of biphenylacetic acid alter extracellular hippocampal glutamate but not gamma-aminobutyric acid levels in conscious rats	2002	46	471-477	Antimicrob Agents Chemother
Smolders, I.; Khan, G. M.; Lindekens, H.; Prikken, S.; Marvin, C. A.; Manil, J.; Ebinger, G.; Michotte, Y.	Effectiveness of vigabatrin against focally evoked pilocarpine-induced seizures and concomitant changes in extracellular hippocampal and cerebellar glutamate, gamma-aminobutyric acid and dopamine levels, a microdialysis-electrocorticography study in freely moving rats	1997	283	1239-1248	J Pharmacol Exp Ther
Smolders, I.; Khan, G. M.; Manil, J.; Ebinger, G.; Michotte, Y.	NMDA receptor-mediated pilocarpine-induced seizures: characterization in freely moving rats by microdialysis	1997	121	1171-1179	Br J Pharmacol
Smolders, I.; Lindekens, H.; Clinckers, R.; Meurs, A.; Neill, M. J.; Lodge, D.; Ebinger, G.; Michotte, Y.	In vivo modulation of extracellular hippocampal glutamate and GABA levels and limbic seizures by group I and II metabotropic glutamate receptor ligands	2004	88	1068-1077	J Neurochem
Smolders, I.; Loo, J. V.; Sarre, S.; Ebinger, G.; Michotte, Y.	Effects of dietary sucrose on hippocampal serotonin release: a microdialysis study in the freely-moving rat	2001	86	151-155	Br J Nutr
Smolders, I.; Sarre, S.; Ebinger, G.; Michotte, Y.	Microbore liquid chromatography analysis of amino acid transmitters	1997	72	197-204	Methods Mol Biol
Smolders, I.; Sarre, S.; Michotte, Y.; Ebinger, G.	The analysis of excitatory, inhibitory and other amino acids in rat brain microdialysates using microbore liquid chromatography	1995	57	47-53	J Neurosci Methods
Smolders, I.; Van Belle, K.; Ebinger, G.; Michotte, Y.	Hippocampal and cerebellar extracellular amino acids during pilocarpine-induced seizures in freely moving rats	1997	319	21-29	Eur J Pharmacol
Soares, J.; Kliem, M. A.; Betarbet, R.; Greenamyre, J. T.; Yamamoto, B.; Wichmann, T.	Role of external pallidal segment in primate parkinsonism: comparison of the effects of 1-methyl-4-phenyl-1,2,3,6-tetrahydropyridine-induced parkinsonism and lesions of the external pallidal segment	2004	24	6417-6426	J Neurosci
Sommer, W.; Rimondini, R.; Connor, W.; Hansson, A. C.; Ungerstedt, U.;	Intrastriatally injected c-fos antisense oligonucleotide interferes with striatonigral but not striatopallidal gamma-aminobutyric acid	1996	93	14134-14139	Proc Natl Acad Sci U S A

GABA					
**Authors**	**Title**	**Year**	**Vol**	**Pages**	**Journal**
Fuxe, D. K.	transmission in the conscious rat				
Song, P.; Mabrouk, O. S.; Hershey, N. D.; Kennedy, R. T.	In vivo neurochemical monitoring using benzoyl chloride derivatization and liquid chromatography-mass spectrometry	2012	84	412-419	Anal Chem
Sotomayor, R.; Forray, M. I.; Gysling, K.	Acute morphine administration increases extracellular DA levels in the rat lateral septum by decreasing the GABAergic inhibitory tone in the ventral tegmental area	2005	81	132-139	J Neurosci Res
Sotomayor-Zarate, R.; Araya, K. A.; Pereira, P.; Blanco, E.; Quiroz, G.; Pozo, S.; Carreno, P.; Andres, M. E.; Forray, M. I.; Gysling, K.	Activation of GABA-B receptors induced by systemic amphetamine abolishes dopamine release in the rat lateral septum	2010	114	1678-1686	J Neurochem
Soukupova, M.; Binaschi, A.; Falcicchia, C.; Zucchini, S.; Roncon, P.; Palma, E.; Magri, E.; Grandi, E.; Simonato, M.	Impairment of GABA release in the hippocampus at the time of the first spontaneous seizure in the pilocarpine model of temporal lobe epilepsy	2014	257	39-49	Exp Neurol
Specter, S. E.; Horwitz, B. A.; Beverly, J. L.	Basal and glucoprivic-induced changes in extracellular GABA in the ventral hypothalamus of Zucker rats	1996	271	R388-R392	Am J Physiol
Stanley, E. M.; Fadel, J. R.	Aging-related alterations in orexin/hypocretin modulation of septo-hippocampal amino acid neurotransmission	2011	195	70-79	Neuroscience
Stanley, E. M.; Fadel, J. R.; Mott, D. D.	Interneuron loss reduces dendritic inhibition and GABA release in hippocampus of aged rats	2012	33	431.e1-431.e13	Neurobiol Aging
Starowicz, K.; Maione, S.; Cristino, L.; Palazzo, E.; Marabese, I.; Rossi, F.; de Novellis, V.; Di Marzo, V.	Tonic endovanilloid facilitation of glutamate release in brainstem descending antinociceptive pathways	2007	27	13739-13749	J Neurosci
Stengard, K.; Connor, W. T.	Acute toluene exposure decreases extracellular gamma-aminobutyric acid in the globus pallidus but not in striatum: a microdialysis study in awake, freely moving rats	1994	292	43-46	Eur J Pharmacol
Stengard, K.; Tham, R.; Connor, W. T.; Hoglund, G.; Ungerstedt, U.	Acute toluene exposure increases extracellular GABA in the cerebellum of rat: a microdialysis study	1993	73	315-318	Pharmacol Toxicol
Stiller, C. O.; Bergquist, J.; Beck, O.; Ekman, R.; Brodin, E.	Local administration of morphine decreases the extracellular level of GABA in the periaqueductal gray matter of freely moving rats	1996	209	165-168	Neurosci Lett
Stiller, C. O.; Linderoth, B.; Connor, W. T.; Franck, J.; Falkenberg, T.; Ungerstedt, U.; Brodin, E.	Repeated spinal cord stimulation decreases the extracellular level of gamma-aminobutyric acid in the periaqueductal gray matter of freely moving rats	1995	699	231-241	Brain Res
Stork, O.; Ji, F. Y.; Obata, K.	Reduction of extracellular GABA in the mouse amygdala during	2002	327	138-	Neurosci Lett

GABA					
Authors	Title	Year	Vol	Pages	Journal
	and following confrontation with a conditioned fear stimulus			142	
Storozheva, Z. I.; Proshin, A. T.	Selective involvement of the neurotransmitter systems of the cerebellum in the mechanisms forming different types of defensive behavior	2011	41	964-972	Neurosci Behav Physiol
Storozheva, Z. I.; Proshin, A. T.	[Selective involvement of cerebellar neurotransmitter systems in mechanisms of various types of defensive behavior]	2010	60	474-485	Zh Vyssh Nerv Deiat Im I P Pavlova
Stragier, B.; Hristova, I.; Sarre, S.; Ebinger, G.; Michotte, Y.	In vivo characterization of the angiotensin-(1-7)-induced dopamine and gamma-aminobutyric acid release in the striatum of the rat	2005	22	658-664	Eur J Neurosci
Suarez, L. M.; Munoz, M. D.; Gonzalez, J. C.; Bustamante, J.; Del Rio, R. M.; Solis, J. M.	The taurine transporter substrate guanidinoethyl sulfonate mimics the action of taurine on long-term synaptic potentiation	2016	48	2647-2656	Amino Acids
Sun, J. Y.; Yang, J. Y.; Wang, F.; Hou, Y.; Dong, Y. X.; Wu, C. F.	GABA receptors in VTA mediate the morphine-induced release of ascorbic acid in rat nucleus accumbens	2011	1368	52-58	Brain Res
Sun, J. Y.; Yang, J. Y.; Wang, F.; Wang, J. Y.; Song, W.; Su, G. Y.; Dong, Y. X.; Wu, C. F.	Lesions of nucleus accumbens affect morphine-induced release of ascorbic acid and GABA but not of glutamate in rats	2011	16	540-550	Addict Biol
Sun, Z.; Jia, J.; Gong, X.; Jia, Y.; Deng, J.; Wang, X.	Inhibition of glutamate and acetylcholine release in behavioral improvement induced by electroacupuncture in parkinsonian rats	2012	520	32-37	Neurosci Lett
Sun, Z.; You, J.; Li, G.; Zhao, X.; Suo, Y.; Wang, X.	Determination of amino acids in rat brain microdialysate with 1,2,5,6-dibenzocarbazole-9-ethyl chloroformate as labeling reagent by high performance liquid chromatographic fluorescence detection and mass spectrometric identification	2011	879	1367-1374	J Chromatogr B Analyt Technol Biomed Life Sci
Sustkova-Fiserova, M.; Vavrova, J.; Krsiak, M.	Brain levels of GABA, glutamate and aspartate in sociable, aggressive and timid mice: an in vivo microdialysis study	2009	30	79-84	Neuro Endocrinol Lett
Sved, A. F.; Curtis, J. T.	Amino acid neurotransmitters in nucleus tractus solitarius: an in vivo microdialysis study	1993	61	2089-2098	J Neurochem
Swanson, R. A.; Chen, J.; Graham, S. H.	Glucose can fuel glutamate uptake in ischemic brain	1994	14	1-6	J Cereb Blood Flow Metab
Szeg, E. M.; Kekesi, K. A.; Szabo, Z.; Janaky, T.; Juhasz, G. D.	Estrogen regulates cytoskeletal flexibility, cellular metabolism and synaptic proteins: A proteomic study	2010	35	807-819	Psychoneuroendocrinology
Szumlinski, K. K.; Diab, M. E.; Friedman, R.; Henze, L. M.; Lominac, K. D.; Bowers, M. S.	Accumbens neurochemical adaptations produced by binge-like alcohol consumption	2007	190	415-431	Psychopharmacology (Berl)

GABA					
**Authors**	**Title**	**Year**	**Vol**	**Pages**	**Journal**
Szumlinski, K. K.; Liu, A.; Penzner, J. H.; Lominac, K. D.	Protracted pro-addictive phenotype produced in mice by pre-adolescent phenylpropanolamine	2007	32	1760-1773	Neuropsychopharmacology
Szyndler, J.; Maciejak, P.; Turzynska, D.; Sobolewska, A.; Lehner, M.; Taracha, E.; Walkowiak, J.; Skorzewska, A.; Wislowska-Stanek, A.; Hamed, A.; Bidzinski, A.; Plaznik, A.	Changes in the concentration of amino acids in the hippocampus of pentylenetetrazole-kindled rats	2008	439	245-249	Neurosci Lett
Tabata, M.; Kurosawa, H.; Kikuchi, Y.; Hida, W.; Ogawa, H.; Okabe, S.; Tun, Y.; Hattori, T.; Shirato, K.	Role of GABA within the nucleus tractus solitarii in the hypoxic ventilatory decline of awake rats	2001	281	R1411-R1419	Am J Physiol Regul Integr Comp Physiol
Takagi, K.; Ginsberg, M. D.; Globus, M. Y.; Dietrich, W. D.; Martinez, E.; Kraydieh, S.; Busto, R.	Changes in amino acid neurotransmitters and cerebral blood flow in the ischemic penumbral region following middle cerebral artery occlusion in the rat: correlation with histopathology	1993	13	575-585	J Cereb Blood Flow Metab
Takazawa, A.; Murashima, Y. L.; Minatogawa, Y.; Kojima, T.; Tanaka, K.; Yamauchi, T.	In vivo microdialysis monitoring for extracellular glutamate and GABA in the ventral hippocampus of the awake rat during kainate-induced seizures	1995	49	S275-S277	Psychiatry Clin Neurosci
Takazawa, A.; Murashima, Y. L.; Minatogawa, Y.; Kojima, T.; Yamazaki, O.; Yamauchi, T.	Changes of extracellular GABA concentrations in the ventral hippocampus during kainate-induced seizures in rats	1993	47	403-405	Jpn J Psychiatry Neurol
Takeda, A.; Hirate, M.; Tamano, H.; Oku, N.	Release of glutamate and GABA in the hippocampus under zinc deficiency	2003	72	537-542	J Neurosci Res
Takeda, A.; Itoh, H.; Imano, S.; Oku, N.	Impairment of GABAergic neurotransmitter system in the amygdala of young rats after 4-week zinc deprivation	2006	49	746-750	Neurochem Int
Takeda, A.; Minami, A.; Seki, Y.; Oku, N.	Differential effects of zinc on glutamatergic and GABAergic neurotransmitter systems in the hippocampus	2004	75	225-229	J Neurosci Res
Takeda, A.; Minami, A.; Seki, Y.; Oku, N.	Inhibitory function of zinc against excitation of hippocampal glutamatergic neurons	2003	57	169-174	Epilepsy Res
Takeda, A.; Sakurada, N.; Kanno, S.; Ando, M.; Oku, N.	Vulnerability to seizures induced by potassium dyshomeostasis in the hippocampus in aged rats	2008	54	37-42	J Health Sci
Takeda, A.; Sotogaku, N.; Oku, N.	Influence of manganese on the release of neurotransmitters in rat striatum	2003	965	279-282	Brain Res
Takeda, A.; Sotogaku, N.; Oku, N.	Manganese influences the levels of neurotransmitters in synapses in rat brain	2002	114	669-674	Neuroscience

GABA					
**Authors**	**Title**	**Year**	**Vol**	**Pages**	**Journal**
Takeda, A.; Tamano, H.; Kan, F.; Hanajima, T.; Yamada, K.; Oku, N.	Enhancement of social isolation-induced aggressive behavior of young mice by zinc deficiency	2008	82	909-914	Life Sci
Takeda, A.; Tamano, H.; Oku, N.	Involvement of unusual glutamate release in kainate-induced seizures in zinc-deficient adult rats	2005	66	137-143	Epilepsy Res
Tamano, H.; Takeda, A.	Suppressive effect of Saiko-ka-ryukotsu-borei-to, a herbal medicine, on excessive release of glutamate in the hippocampus	2004	64	273-277	Brain Res Bull
Tan, W. K.; Williams, C. E.; During, M. J.; Mallard, C. E.; Gunning, M. I.; Gunn, A. J.; Gluckman, P. D.	Accumulation of cytotoxins during the development of seizures and edema after hypoxic-ischemic injury in late gestation fetal sheep	1996	39	791-797	Pediatr Res
Tanahashi, S.; Ueda, Y.; Nakajima, A.; Yamamura, S.; Nagase, H.; Okada, M.	Novel delta1-receptor agonist KNT-127 increases the release of dopamine and L-glutamate in the striatum, nucleus accumbens and median pre-frontal cortex	2012	62	2057-2067	Neuropharmacology
Tanahashi, S.; Yamamura, S.; Nakagawa, M.; Motomura, E.; Okada, M.	Dopamine D2 and serotonin 5-HT1A receptors mediate the actions of aripiprazole in mesocortical and mesoaccumbens transmission	2012	62	765-774	Neuropharmacology
Tanaka, S.; Kiuchi, Y.; Tsuchida, A.; Yamamoto, T.; Oguchi, K.; Yoshida, T.; Kuroiwa, Y.	Possible involvement of an increase in brain extracellular concentration of excitatory amino acids in convulsions in phenobarbital-withdrawn rats	1996	17	91-102	Res Commun Alcohol S
Tang, X. C.; McFarland, K.; Cagle, S.; Kalivas, P. W.	Cocaine-induced reinstatement requires endogenous stimulation of mu-opioid receptors in the ventral pallidum	2005	25	4512-4520	J Neurosci
Tang, Y. B.; Sun, F.; Teng, L.; Li, W. B.; An, S. M.; Zhang, C.; Yang, X. J.; Lv, H. Y.; Ding, X. P.; Zhu, L.; Chen, H. Z.	Simultaneous determination of the repertoire of classical neurotransmitters released from embryonal carcinoma stem cells using online microdialysis coupled with hydrophilic interaction chromatography-tandem mass spectrometry	2014	849	70-79	Anal Chim Acta
Tanganelli, S.; Connor, W. T.; Ferraro, L.; Bianchi, C.; Beani, L.; Ungerstedt, U.; Fuxe, K.	Facilitation of GABA release by neurotensin is associated with a reduction of dopamine release in rat nucleus accumbens	1994	60	649-657	Neuroscience
Tedesco, A.; Ally, A.	Angiotensin II type-2 (AT2) receptor antagonism alters cardiovascular responses to static exercise and simultaneously changes glutamate/GABA levels within the ventrolateral medulla	2009	64	372-379	Neurosci Res
Tejeda, H. A.; Counotte, D. S.; Oh, E.; Ramamoorthy, S.; Schultz-Kuszak, K. N.; Backman, C. M.; Chefer, V.;	Prefrontal cortical kappa-opioid receptor modulation of local neurotransmission and conditioned place aversion	2013	38	1770-1779	Neuropsychopharmacology

GABA					
**Authors**	**Title**	**Year**	**Vol**	**Pages**	**Journal**
Donnell, P.; Shippenberg, T. S.					
Terzioglu, B.; Aypak, C.; Onat, F. Y.; Kucukibrahimoglu, E.; Ozkaynakci, A. E.; Goren, M. Z.	The effects of ethosuximide on amino acids in genetic absence epilepsy rat model	2006	100	227-233	J Pharmacol Sci
Terzioglu, B.; Karaalp, A.; Zafer Goren, M.	A linear relationship between lamotrigine and GABA in cerebrospinal fluid	2011	15	1-6	Marmara Pharm J
Thorpe, A. J.; Doane, D. F.; Sweet, D. C.; Beverly, J. L.; Kotz, C. M.	Orexin A in the rostrolateral hypothalamic area induces feeding by modulating GABAergic transmission	2006	1125	60-66	Brain Res
Timmerman, W.; Bouma, M.; De Vries, J. B.; Davis, M.; Westerink, B. H.	A microdialysis study on the mechanism of action of gabapentin	2000	398	53-57	Eur J Pharmacol
Timmerman, W.; Westerink, B. H.	Electrical stimulation of the substantia nigra reticulata: detection of neuronal extracellular GABA in the ventromedial thalamus and its regulatory mechanism using microdialysis in awake rats	1997	26	62-71	Synapse
Timmerman, W.; Westerink, B. H.	Extracellular gamma-aminobutyric acid in the substantia nigra reticulata measured by microdialysis in awake rats: effects of various stimulants	1995	197	21-24	Neurosci Lett
Timmerman, W.; Zwaveling, J.; Westerink, B. H.	Characterization of extracellular GABA in the substantia nigra reticulata by means of brain microdialysis	1992	345	661-665	Naunyn Schmiedebergs Arch Pharmacol
Tin Tin Win, Shwe; Mitsushima, D.; Kimura, F.	Profiles of in vivo gamma-aminobutyric acid release in the medial preoptic area of intact and castrated male rats	2002	76	290-296	Neuroendocrinology
Tin Tin Win, Shwe; Mitsushima, D.; Shinohara, K.; Kimura, F.	Sexual dimorphism of GABA release in the medial preoptic area and luteinizing hormone release in gonadectomized estrogen-primed rats	2004	127	243-250	Neuroscience
Tinimerman, W.; Westerink, B. H. C.	Extracellular gamma-aminobutyric acid in the substantia nigra reticulata measured by microdialysis in awake rats: Effects of various stimulants	1995	197	21-24	Neurosci Lett
Togashi, H.; Nakamura, K.; Matsumoto, M.; Ueno, K.; Ohashi, S.; Saito, H.; Yoshioka, M.	Aniracetam enhances glutamatergic transmission in the prefrontal cortex of stroke-prone spontaneously hypertensive rats	2002	320	109-112	Neurosci Lett
Torregrossa, M. M.; Kalivas, P. W.	Neurotensin in the ventral pallidum increases extracellular gamma-aminobutyric acid and differentially affects cue- and cocaine-primed reinstatement	2008	325	556-566	J Pharmacol Exp Ther
Tossman, U.; Eriksson, S.; Delin, A.;	Brain amino acids measured by intracerebral dialysis in portacaval	1983	41	1046-	J Neurochem

GABA					
**Authors**	**Title**	**Year**	**Vol**	**Pages**	**Journal**
Hagenfeldt, L.; Law, D.; Ungerstedt, U.	shunted rats			1051	
Tossman, U.; Segovia, J.; Ungerstedt, U.	Extracellular levels of amino acids in striatum and globus pallidus of 6-hydroxydopamine-lesioned rats measured with microdialysis	1986	127	547-551	Acta Physiol Scand
Tossman, U.; Ungerstedt, U.	The effect of apomorphine and pergolide on the potassium-evoked overflow of GABA in rat striatum studied by microdialysis	1986	123	295-298	Eur J Pharmacol
Tossman, U.; Wieloch, T.; Ungerstedt, U.	gamma-Aminobutyric acid and taurine release in the striatum of the rat during hypoglycemic coma, studied by microdialysis	1985	62	231-235	Neurosci Lett
Touret, M.; Parrot, S.; Denoroy, L.; Belin, M. F.; Didier-Bazes, M.	Glutamatergic alterations in the cortex of genetic absence epilepsy rats	2007	8	69	BMC Neurosci
Toya, S.; Takatsuru, Y.; Kokubo, M.; Amano, I.; Shimokawa, N.; Koibuchi, N.	Early-life-stress affects the homeostasis of glutamatergic synapses	2014	40	3627-3634	Eur J Neurosci
Toyota, S.; Graf, R.; Valentino, M.; Yoshimine, T.; Heiss, W. D.	Malignant infarction in cats after prolonged middle cerebral artery occlusion: glutamate elevation related to decrease of cerebral perfusion pressure	2002	33	1383-1391	Stroke
Trabace, L.; Kendrick, K. M.	Nitric oxide can differentially modulate striatal neurotransmitter concentrations via soluble guanylate cyclase and peroxynitrite formation	2000	75	1664-1674	J Neurochem
Trevitt, J.; Carlson, B.; Correa, M.; Keene, A.; Morales, M.; Salamone, J.	Interactions between dopamine D1 receptors and gamma-aminobutyric acid mechanisms in substantia nigra pars reticulata of the rat: Neurochemical and behavioral studies	2002	159	229-237	Psychopharmacology
Tsukada, Y.; Kanamatsu, T.; Takahara, H.	Neurotransmitter release from the medial hyperstriatum ventrale of the chick forebrain accompanying filial imprinting behavior, measured by in vivo microdialysis	1999	24	315-320	Neurochem Res
Tucci, S.; Contreras, Q.; Paez, X.; Gonzalez, L.; Rada, P.; Hernandez, L.	Medial prefrontal transection enhances social interaction. II: neurochemical studies	2000	887	259-265	Brain Res
Tuma, P.; Sustkova-Fiserova, M.; Opekar, F.; Pavlicek, V.; Malkova, K.	Large-volume sample stacking for in vivo monitoring of trace levels of gamma-aminobutyric acid, glycine and glutamate in microdialysates of periaqueductal gray matter by capillary electrophoresis with contactless conductivity detection	2013	1303	94-99	Jr Chromatogr A
Uchiyama-Tsuyuki, Y.; Araki, H.; Yae, T.; Otomo, S.	Changes in the extracellular concentrations of amino acids in the rat striatum during transient focal cerebral ischemia	1994	62	1074-1078	J Neurochem
Ueda, Y.; Mitsuyama, Y.	Nitroprusside inhibits kindling development and potentiates excitatory amino acid exocytosis in the kindling rat	1997	23	49-60	J Brain Sci

GABA					
**Authors**	**Title**	**Year**	**Vol**	**Pages**	**Journal**
Ueda, Y.; Tsuru, N.	Simultaneous monitoring of the seizure-related changes in extracellular glutamate and gamma-aminobutyric acid concentration in bilateral hippocampi following development of amygdaloid kindling	1995	20	213-219	Epilepsy Res
Uutela, P.; Ketola, R. A.; Piepponen, P.; Kostiainen, R.	Comparison of different amino acid derivatives and analysis of rat brain microdialysates by liquid chromatography tandem mass spectrometry	2009	633	223-231	Anal Chim Acta
Valle-Dorado, M. G.; Santana-Gomez, C. E.; Orozco-Suarez, S. A.; Rocha, L.	The mast cell stabilizer sodium cromoglycate reduces histamine release and status epilepticus-induced neuronal damage in the rat hippocampus	2015	92	49-55	Neuropharmacology
Valtonen, P.; Haapalinna, A.; Riekkinen, P., Sr.; Halonen, T.	Effect of alpha 2-adrenergic drugs dexmedetomidine and atipamezole on extracellular amino acid levels in vivo	1995	285	239-246	Eur J Pharmacol
Vanini, G.; Lydic, R.; Baghdoyan, H. A.	GABA-to-ACh ratio in basal forebrain and cerebral cortex varies significantly during sleep	2012	35	1325-1334	Sleep
Vanini, G.; Nemanis, K.; Baghdoyan, H. A.; Lydic, R.	GABAergic transmission in rat pontine reticular formation regulates the induction phase of anesthesia and modulates hyperalgesia caused by sleep deprivation	2014	40	2264-2273	Eur J Neurosci
Vanini, G.; Wathen, B. L.; Lydic, R.; Baghdoyan, H. A.	Endogenous GABA levels in the pontine reticular formation are greater during wakefulness than during rapid eye movement sleep	2011	31	2649-2656	J Neurosci
Varatharajan, R.; Joseph, K.; Neto, S. C.; Hofmann, U. G.; Moser, A.; Tronnier, V.	Electrical high frequency stimulation modulates GABAergic activity in the nucleus accumbens of freely moving rats	2015	90	255-260	Neurochem Int
Vazquez-DeRose, J.; Schwartz, M. D.; Nguyen, A. T.; Warrier, D. R.; Gulati, S.; Mathew, T. K.; Neylan, T. C.; Kilduff, T. S.	Hypocretin/orexin antagonism enhances sleep-related adenosine and GABA neurotransmission in rat basal forebrain	2016	221	923-940	Brain Struct Funct
Venton, B. J.; Robinson, T. E.; Kennedy, R. T.	Transient changes in nucleus accumbens amino acid concentrations correlate with individual responsivity to the predator fox odor 2,5-dihydro-2,4,5-trimethylthiazoline	2006	96	236-246	J Neurochem
Venton, B. J.; Robinson, T. E.; Kennedy, R. T.; Maren, S.	Dynamic amino acid increases in the basolateral amygdala during acquisition and expression of conditioned fear	2006	23	3391-3398	Eur J Neurosci
Viggiano, A.; Monda, M.; Viggiano, A.; Chiefari, M.; Aurilio, C.; De Luca, B.	Evidence that GABAergic neurons in the spinal trigeminal nucleus are involved in the transmission of inflammatory pain in the rat: a	2004	496	87-92	Eur J Pharmacol

GABA					
**Authors**	**Title**	**Year**	**Vol**	**Pages**	**Journal**
	microdialysis and pharmacological study				
Viggiano, A.; Monda, M.; Viggiano, A.; Fuccio, F.; De Luca, B.	Extracellular GABA in the medial hypothalamus is increased following hypocretin-1 administration	2004	182	89-94	Acta Physiol Scand
Vihavainen, T.; Relander, T. R.; Leiviska, R.; Airavaara, M.; Tuominen, R. K.; Ahtee, L.; Piepponen, T. P.	Chronic nicotine modifies the effects of morphine on extracellular striatal dopamine and ventral tegmental GABA	2008	107	844-854	J Neurochem
Voisin, D. L.; Chapman, C.; Poulain, D. A.; Herbison, A. E.	Extracellular GABA concentrations in rat supraoptic nucleus during lactation and following haemodynamic changes: an in vivo microdialysis study	1994	63	547-558	Neuroscience
Voisin, D. L.; Herbison, A. E.; Chapman, C.; Poulain, D. A.	Effects of central GABAB receptor modulation upon the milk ejection reflex in the rat	1996	63	368-376	Neuroendocrinology
Volta, M.; Marti, M.; McDonald, J.; Molinari, S.; Camarda, V.; Pela, M.; Trapella, C.; Morari, M.	Pharmacological profile and antiparkinsonian properties of the novel nociceptin/orphanin FQ receptor antagonist 1-[1-cyclooctylmethyl-5-(1-hydroxy-1-methyl-ethyl)-1,2,3,6-tetrahydro-pyridin-4-y l]-3-ethyl-1,3-dihydro-benzoimidazol-2-one (GF-4)	2010	31	1194-1204	Peptides
Volta, M.; Viaro, R.; Trapella, C.; Marti, M.; Morari, M.	Dopamine-nociceptin/orphanin FQ interactions in the substantia nigra reticulata of hemiparkinsonian rats: Involvement of D2/D3 receptors and impact on nigro-thalamic neurons and motor activity	2011	228	126-137	Exp Neurol
Vondrakova, K.; Syslova, K.; Mikoska, M.; Kacer, P.; Burchfiel, J.; Kubova, H.; Vales, K.; Tsenov, G.	The role of endothelin receptors in neurochemical changes during focal cerebral ischemia induced by intrahippocampal injection of the endothelin-1 in immature rats	2016	36	477	J Cereb Blood Flow Met
Waldmeier, P. C.; Stocklin, K.; Feldtrauer, J. J.	Systemic administration of baclofen and the GABAB antagonist, CGP 35348, does not affect GABA, glutamate or aspartate in microdialysates of the striatum of conscious rats	1992	345	548-552	Naunyn Schmiedebergs Arch Pharmacol
Waldmeier, P. C.; Stocklin, K.; Feldtrauer, J. J.	Weak effects of local and systemic administration of the GABA uptake inhibitor, SK&F 89976, on extracellular GABA in the rat striatum	1992	345	544-547	Naunyn Schmiedebergs Arch Pharmacol
Wang, F.; Chen, H.; Sharp, B. M.	Neuroadaptive changes in the mesocortical glutamatergic system during chronic nicotine self-administration and after extinction in rats	2008	106	943-956	J Neurochem
Wang, J.; Shen, L. L.; Cao, Y. X.; Sun, Z. J.; Wang, Q.; Zhu, D. N.	[Role of angiotensin-(1-7) in amino-acid-neurotransmitter-mediated blood pressure regulation in rat rostral ventrolateral medulla]	2001	53	1-6	Sheng Li Xue Bao
Wang, J.; Shen, L. L.; Cao, Y. X.; Zhu,	Effects of electroacupuncture on pressor response to angiotensin-	2003	28	25-34	Acupunct Electrother Res

GABA					
**Authors**	**Title**	**Year**	**Vol**	**Pages**	**Journal**
D. N.	(1-7) by amino acid release in the rostral ventrolateral medulla				
Wang, L.; Huang, Y.; Wu, J.; Lv, G.; Zhou, L.; Jia, J.	Effect of Buyang Huanwu decoction on amino acid content in cerebrospinal fluid of rats during ischemic/reperfusion injury	2013	86	143-150	J Pharm Biomed Anal
Wang, L.; Maher, T. J.; Wurtman, R. J.	Oral L-glutamine increases GABA levels in striatal tissue and extracellular fluid	2007	21	1227-1232	Faseb J
Wang, M.; Hershey, N. D.; Mabrouk, O. S.; Kennedy, R. T.	Collection, storage, and electrophoretic analysis of nanoliter microdialysis samples collected from awake animals in vivo	2011	400	2013-2023	Anal Bioanal Chem
Wang, S.; Fu, F. H.; Zhu, H.	The effects of chronic exercise on the autonomic balance of the cardiovascular system in hypertensive rats	2009	168	273-281	Gazz Med Ital Edizioni Minerva Medica
Wang, X.; Ai, J.; Hampson, D. R.; Snead, Iii O. C.	Altered glutamate and GABA release within thalamocortical circuitry in metabotropic glutamate receptor 4 knockout mice	2005	134	1195-1203	Neuroscience
Wang, X.; Shimizu-Sasamata, M.; Moskowitz, M. A.; Newcomb, R.; Lo, E. H.	Profiles of glutamate and GABA efflux in core versus peripheral zones of focal cerebral ischemia in mice	2001	313	121-124	Neurosci Lett
Ward, R. J.; Colantuoni, C.; Dahchour, A.; Quertemont, E.; De Witte, P.	Acetaldehyde-induced changes in monoamine and amino acid extracellular microdialysate content of the nucleus accumbens	1997	36	225-232	Neuropharmacology
Ward, R. J.; Colivicchi, M. A.; Allen, R.; Schol, F.; Lallemand, F.; de Witte, P.; Ballini, C.; Corte, L. D.; Dexter, D.	Neuro-inflammation induced in the hippocampus of binge drinking rats may be mediated by elevated extracellular glutamate content	2009	111	1119-1128	J Neurochem
Watson, C. J.; Lydic, R.; Baghdoyan, H. A.	Sleep duration varies as a function of glutamate and GABA in rat pontine reticular formation	2011	118	571-580	J Neurochem
Watson, C. J.; Lydic, R.; Baghdoyan, H. A.	Sleep and GABA levels in the oral part of rat pontine reticular formation are decreased by local and systemic administration of morphine	2007	144	375-386	Neuroscience
Watson, C. J.; Soto-Calderon, H.; Lydic, R.; Baghdoyan, H. A.	Pontine reticular formation (PnO) administration of hypocretin-1 increases PnO GABA levels and wakefulness	2008	31	453-464	Sleep
Wei, J.; Yao, L.; Yang, L.; Zhao, W.; Shi, S.; Cai, Q.; Chen, D.; Li, W.; Wang, Q.	Alteration of glutamate/GABA balance during acute alcohol intoxication in rats: effect of Xingnaojing injection	2015	166	333-339	J Ethnopharmacol
Wei, N.; Wang, Y.; Wang, X.; He, Z.; Zhang, M.; Zhang, X.; Pan, Y.; Zhang, J.; Qin, Z.; Zhang, K.	The different effects of high-frequency stimulation of the nucleus accumbens shell and core on food consumption are possibly associated with different neural responses in the lateral hypothalamic area	2015	301	312-322	Neuroscience

GABA					
**Authors**	**Title**	**Year**	**Vol**	**Pages**	**Journal**
Welsch-Kunze, S.; Kleim, J.; Dietze, S.; Kuschinsky, K.	Recordings of extracellular glutamate and gamma-aminobutyric acid in the striatum of non-anesthetized rats. K(+)-stimulation, its Ca(2+)-dependence and lack of effects of drugs acting on dopamine receptors	1993	43	85-91	Arzneimittelforschung
Welty, N.; Shoblock, J. R.	The effects of thioperamide on extracellular levels of glutamate and GABA in the rat prefrontal cortex	2009	207	433-438	Psychopharmacoly (Berl)
Westergren, I.; Nystrom, B.; Hamberger, A.; Johansson, B. B.	Intracerebral dialysis and the blood-brain barrier	1995	64	229-234	J Neurochem
Westergren, I.; Nystrom, B.; Hamberger, A.; Johansson, B. B.	Amino acids in extracellular fluid in vasogenic brain edema	1994	60	124-127	Acta Neurochir Suppl (Wien)
Westergren, I.; Nystrom, B.; Hamberger, A.; Nordborg, C.; Johansson, B. B.	Concentrations of amino acids in extracellular fluid after opening of the blood-brain barrier by intracarotid infusion of protamine sulfate	1994	62	159-165	J Neurochem
Westerink, B. H.; de Vries, J. B.	On the origin of extracellular GABA collected by brain microdialysis and assayed by a simplified on-line method	1989	339	603-607	Naunyn Schmiedebergs Arch Pharmacol
Wilson, C. L.; Maidment, N. T.; Shomer, M. H.; Behnke, E. J.; Ackerson, L.; Fried, I.; Engel, J., Jr.	Comparison of seizure related amino acid release in human epileptic hippocampus versus a chronic, kainate rat model of hippocampal epilepsy	1996	26	245-254	Epilepsy Res
Windels, F.; Bruet, N.; Poupard, A.; Feuerstein, C.; Bertrand, A.; Savasta, M.	Influence of the frequency parameter on extracellular glutamate and gamma-aminobutyric acid in substantia nigra and globus pallidus during electrical stimulation of subthalamic nucleus in rats	2003	72	259-267	J Neurosci Res
Windels, F.; Bruet, N.; Poupard, A.; Urbain, N.; Chouvet, G.; Feuerstein, C.; Savasta, M.	Effects of high frequency stimulation of subthalamic nucleus on extracellular glutamate and GABA in substantia nigra and globus pallidus in the normal rat	2000	12	4141-4146	Eur J Neurosci
Windels, F.; Carcenac, C.; Poupard, A.; Savasta, M.	Pallidal origin of GABA release within the substantia nigra pars reticulata during high-frequency stimulation of the subthalamic nucleus	2005	25	5079-5086	J Neurosci
Win-Shwe, T. T.; Mitsushima, D.; Nakajima, D.; Ahmed, S.; Yamamoto, S.; Tsukahara, S.; Kakeyama, M.; Goto, S.; Fujimaki, H.	Toluene induces rapid and reversible rise of hippocampal glutamate and taurine neurotransmitter levels in mice	2007	168	75-82	Toxicol Lett
Wislowska-Stanek, A.; Hamed, A.; Lehner, M.; Bidzinski, A.; Turzynska, D.;	Effects of midazolam and buspirone on in vivo concentration of amino acids and monoamine metabolites in the rat hippocampus	2008	60	209-218	Pharmacol Rep

GABA					
**Authors**	**Title**	**Year**	**Vol**	**Pages**	**Journal**
Sobolewska, A.; Walkowiak, J.; Plaznik, A.					
Wlodarczyk, A. I.; Sylantyev, S.; Herd, M. B.; Kersante, F.; Lambert, J. J.; Rusakov, D. A.; Linthorst, A. C.; Semyanov, A.; Belelli, D.; Pavlov, I.; Walker, M. C.	GABA-independent GABAA receptor openings maintain tonic currents	2013	33	3905-3914	J Neurosci
Wolfram-Aduan, A.; Altemus, M.; Wickwire, J. H.; Sandstrom, M. I.	Presymptomatic glutamate levels in prefrontal cortex in the Hdh(CAG150) mouse model of Huntington's disease	2014	3	387-399	J Huntingtons Dis
Wong, J. M.; Malec, P. A.; Mabrouk, O. S.; Ro, J.; Dus, M.; Kennedy, R. T.	Benzoyl chloride derivatization with liquid chromatography-mass spectrometry for targeted metabolomics of neurochemicals in biological samples	2016	1446	78-90	J Chromatogr A
Wozniak, M.; Golembiowska, K.; Noworyta-Sokolowska, K.; Acher, F.; Cieslik, P.; Kusek, M.; Tokarski, K.; Pilc, A.; Wieronska, J. M.	Neurochemical and behavioral studies on the 5-HT1A-dependent antipsychotic action of the mGlu4 receptor agonist LSP4-2022	2016	115	149-165	Neuropharmacology
Wu, G.; Liu, X.; Hong, Z.; Tang, T.	Low-frequency hippocampal stimulation increases the extracellular y-aminobutyrate level in the brain, inhibits the epileptic seizures and after discharges of the amygdala in pharmacoresistant temporal lobe epileptic rats. [Chinese]	2014	47	542-547	Zhonghua Shen Jing Ge Za Zhi
Wu, Z. E.; Wang, D. Q.; Li, P.; Sun, X. F.; Wang, W.	The method of simultaneous measurement of the plasma concentration of L-DOPA and extracellular amino acids in striatum of the Parkinson's disease rats induced by 6-OHDA. [Chinese]	2010	26	1395-1399	Zhongguo yao li xue tong bao
Wydra, K.; Golembiowska, K.; Suder, A.; Kaminska, K.; Fuxe, K.; Filip, M.	On the role of adenosine 2A receptors in cocaine-induced reward: A pharmacological and neurochemical analysis in rats	2015	232	421-435	Psychopharmacology
Wydra, K.; Golembiowska, K.; Zaniewska, M.; Kaminska, K.; Ferraro, L.; Fuxe, K.; Filip, M.	Accumbal and pallidal dopamine, glutamate and GABA overflow during cocaine self-administration and its extinction in rats	2013	18	307-324	Addict Biol
Xi, Z. X.; Ramamoorthy, S.; Shen, H.; Lake, R.; Samuvel, D. J.; Kalivas, P. W.	GABA transmission in the nucleus accumbens is altered after withdrawal from repeated cocaine	2003	23	3498-3505	J Neurosci
Xia, C. M.; Shao, C. H.; Xin, L.; Wang, Y. R.; Ding, C. N.; Wang, J.; Shen, L. L.; Li, L.; Cao, Y. X.; Zhu, D. N.	Effects of melatonin on blood pressure in stress-induced hypertension in rats	2008	35	1258-1264	Clin Exp Pharmacol Physiol

GABA					
**Authors**	**Title**	**Year**	**Vol**	**Pages**	**Journal**
Xie, F.; Li, X.; Bao, M.; Shi, R.; Yue, Y.; Guan, Y.; Wang, Y.	Anesthetic propofol normalized the increased release of glutamate and gamma-amino butyric acid in hippocampus after paradoxical sleep deprivation in rats	2015	37	1102-1107	Neurol Res
Xu, P.; Li, J.; Chen, B.; Wang, X.; Cai, X.; Jiang, H.; Wang, C.; Zhang, H.	The real-time neurotoxicity analysis of Fe3O4 nanoparticles combined with daunorubicin for rat brain in vivo	2012	8	417-423	J Biomed Nanotechnol
Xu, X. H.; Zheng, X. X.; Li, H.	Effects of puerarin on excitatory amino acid levels in cerebral ischemic striatum of rat. [Chinese]	2008	43	675-679	Chin Pharmaceutical J
Yager, J. Y.; Armstrong, E. A.; Miyashita, H.; Wirrell, E. C.	Prolonged neonatal seizures exacerbate hypoxic-ischemic brain damage: correlation with cerebral energy metabolism and excitatory amino acid release	2002	24	367-381	Dev Neurosci
Yamada, K.; Moriguchi, A.; Mikami, H.; Okuda, N.; Higaki, J.; Ogihara, T.	The effect of central amino acid neurotransmitters on the antihypertensive response to angiotensin blockade in spontaneous hypertension	1995	13	1624-1630	J Hypertens
Yamamoto, B. K.; Davy, S.	Dopaminergic modulation of glutamate release in striatum as measured by microdialysis	1992	58	1736-1742	J Neurochem
Yamamoto, B. K.; Nash, J. F.; Gudelsky, G. A.	Modulation of methylenedioxymethamphetamine-induced striatal dopamine release by the interaction between serotonin and gamma-aminobutyric acid in the substantia nigra	1995	273	1063-1070	J Pharmacol Exp Ther
Yamamoto, B. K.; Pehek, E. A.; Meltzer, H. Y.	Brain region effects of clozapine on amino acid and monoamine transmission	1994	55 Suppl B	8-14	J Clin Psychiatry
Yamamoto, N.; Pierce, R. C.; Soghomonian, J. J.	Subchronic administration of L-DOPA to adult rats with a unilateral 6-hydroxydopamine lesion of dopamine neurons results in a sensitization of enhanced GABA release in the substantia nigra, pars reticulata	2006	1123	196-200	Brain Res
Yamamoto, Y.; Kakigi, T.; Maeda, K.	Intra-striatal phencyclidine inhibits N-methyl-D-aspartic acid-stimulated increase in glutamate levels of freely moving rats	1999	23	161-174	Prog Neuropsychopharmacol Biol Psychiatry
Yamamoto, Y.; Mochizuki, T.; Okakura-Mochizuki, K.; Uno, A.; Yamatodani, A.	Thioperamide, a histamine H3 receptor antagonist, increases GABA release from the rat hypothalamus	1997	19	289-298	Methods Find Exp Clin Pharmacol
Yamamura, S.; Hamaguchi, T.; Ohoyama, K.; Sugiura, Y.; Suzuki, D.; Kanehara, S.; Nakagawa, M.; Motomura, E.; Matsumoto, T.; Tanii, H.; Shiroyama, T.; Okada, M.	Topiramate and zonisamide prevent paradoxical intoxication induced by carbamazepine and phenytoin	2009	84	172-186	Epilepsy Res

GABA					
**Authors**	**Title**	**Year**	**Vol**	**Pages**	**Journal**
Yamamura, S.; Hoshikawa, M.; Dai, K.; Saito, H.; Suzuki, N.; Niwa, O.; Okada, M.	ONO-2506 inhibits spike-wave discharges in a genetic animal model without affecting traditional convulsive tests via gliotransmission regulation	2013	168	1088-1100	Br J Pharmacol
Yamamura, S.; Ohoyama, K.; Hamaguchi, T.; Kashimoto, K.; Nakagawa, M.; Kanehara, S.; Suzuki, D.; Matsumoto, T.; Motomura, E.; Shiroyama, T.; Okada, M.	Effects of quetiapine on monoamine, GABA, and glutamate release in rat prefrontal cortex	2009	206	243-258	Psychopharmacology (Berl)
Yamamura, S.; Ohoyama, K.; Hamaguchi, T.; Nakagawa, M.; Suzuki, D.; Matsumoto, T.; Motomura, E.; Tanii, H.; Shiroyama, T.; Okada, M.	Effects of zotepine on extracellular levels of monoamine, GABA and glutamate in rat prefrontal cortex	2009	157	656-665	Br Jpharmacol
Yamamura, S.; Ohoyama, K.; Nagase, H.; Okada, M.	Zonisamide enhances delta receptor-associated neurotransmitter release in striato-pallidal pathway	2009	57	322-331	Neuropharmacology
Yamamura, S.; Saito, H.; Suzuki, N.; Kashimoto, S.; Hamaguchi, T.; Ohoyama, K.; Suzuki, D.; Kanehara, S.; Nakagawa, M.; Shiroyama, T.; Okada, M.	Effects of zonisamide on neurotransmitter release associated with inositol triphosphate receptors	2009	454	91-96	Neurosci Lett
Yamanaka, T.; Sasa, M.; Matsunaga, T.	Glutamate as a primary afferent neurotransmitter in the medial vestibular nucleus as detected by in vivo microdialysis	1997	762	243-246	Brain Res
Yamashita, A.; Hamada, A.; Suhara, Y.; Kawabe, R.; Yanase, M.; Kuzumaki, N.; Matsui, R.; Okano, H.; Narita, M.	Astrocytic activation in the anterior cingulate cortex is critical for sleep disorder under neuropathic pain	2014	68	235-247	Synapse
Yan, N.; Chen, N.; Zhu, H.; Zhang, J.; Sim, M.; Ma, Y.; Wang, W.	High-frequency stimulation of nucleus accumbens changes in dopaminergic reward circuit	2013	8	e79318	PLoS One
Yan, Q. S.; Zheng, S. Z.; Feng, M. J.; Yan, S. E.	Involvement of 5-HT1B receptors within the ventral tegmental area in ethanol-induced increases in mesolimbic dopaminergic transmission	2005	1060	126-137	Brain Res
Yan, Q. S.; Zheng, S. Z.; Yan, S. E.	Involvement of 5-HT1B receptors within the ventral tegmental area in regulation of mesolimbic dopaminergic neuronal activity via GABA mechanisms: a study with dual-probe microdialysis	2004	1021	82-91	Brain Res
Yananli, H. R.; Terzioglu, B.; Goren, M.	Extracellular hypothalamic gamma-aminobutyric acid (GABA) and	2008	106	301-	J Pharmacol Sci

GABA					
**Authors**	**Title**	**Year**	**Vol**	**Pages**	**Journal**
Z.; Aker, R. G.; Aypak, C.; Onat, F. Y.	L-glutamic acid concentrations in response to bicuculline in a genetic absence epilepsy rat model			309	
Yang, C. S.; Tsai, P. J.; Chen, W. Y.; Tsai, W. J.; Kuo, J. S.	On-line derivatization for continuous and automatic monitoring of brain extracellular glutamate levels in anesthetized rats: a microdialysis study	1999	734	1-6	J Chromatogr B Biomed Sci Appl
Yang, P. S.; Kim, H.; Lee, W.; Bohlke, M.; Park, S.; Maher, T. J.; Yoo, S. S.	Transcranial focused ultrasound to the thalamus is associated with reduced extracellular GABA levels in rats	2012	65	153-160	Neuropsychobiology
Yang, Z. Z.; Li, J.; Li, S. X.; Feng, W.; Wang, H.	Effect of ginkgolide B on striatal extracellular amino acids in middle cerebral artery occluded rats	2011	136	117-122	J Ethnopharmacol
Yao, H.; Matsumoto, T.; Hirano, M.; Uchimura, H.; Ooboshi, H.; Sadoshima, S.; Fujishima, M.	Striatal glutamic acid and gamma-aminobutyric acid in transient cerebral ischemia in spontaneously hypertensive rats	1990	31	385-392	Jpn Heart J
Yasumatsu, M.; Yazawa, T.; Otokawa, M.; Kuwasawa, K.; Hasegawa, H.; Aihara, Y.	Monoamines, amino acids and acetylcholine in the preoptic area and anterior hypothalamus of rats: measurements of tissue extracts and in vivo microdialysates	1998	121	13-23	Comp Biochem Physiol A Mol Integr Physiol
Yelkenli, I. H.; Ulupinar, E.; Korkmaz, O. T.; Sener, E.; Kus, G.; Filiz, Z.; Tuncel, N.	Modulation of Corpus Striatal Neurochemistry by Astrocytes and Vasoactive Intestinal Peptide (VIP) in Parkinsonian Rats	2016	59	280-289	J Mol Neurosci
Yonezawa, Y.; Kuroki, T.; Kawahara, T.; Tashiro, N.; Uchimura, H.	Involvement of gamma-aminobutyric acid neurotransmission in phencyclidine-induced dopamine release in the medial prefrontal cortex	1998	341	45-56	Eur J Pharmacol
Yoshida, S.; Okada, M.; Zhu, G.; Kaneko, S.	Carbamazepine prevents breakdown of neurotransmitter release induced by hyperactivation of ryanodine receptor	2007	52	1538-1546	Neuropharmacology
Yoshida, S.; Okada, M.; Zhu, G.; Kaneko, S.	Effects of zonisamide on neurotransmitter exocytosis associated with ryanodine receptors	2005	67	153-162	Epilepsy Res
Yoshida, S.; Yamamura, S.; Ohoyama, K.; Nakagawa, M.; Motomura, E.; Kaneko, S.; Okada, M.	Effects of valproate on neurotransmission associated with ryanodine receptors	2010	68	322-328	Neurosci Res
Yoshizumi, M.; Parker, R. A.; Eisenach, J. C.; Hayashida, K.	Gabapentin inhibits gamma-amino butyric acid release in the locus coeruleus but not in the spinal dorsal horn after peripheral nerve injury in rats	2012	116	1347-1353	Anesthesiology
You, Z. B.; Godukhin, O.; Goiny, M.; Nylander, I.; Ungerstedt, U.; Terenius,	Cholecystokinin-8S increases dynorphin B, aspartate and glutamate release in the fronto-parietal cortex of the rat via	1997	355	576-581	Naunyn Schmiedebergs Arch Pharmacol

GABA					
**Authors**	**Title**	**Year**	**Vol**	**Pages**	**Journal**
L.; Hokfelt, T.; Herrera-Marschitz, M.	different receptor subtypes				
You, Z. B.; Herrera-Marschitz, M.; Nylander, I.; Goiny, M.; Conner, W. T.; Ungerstedt, U.; Terenius, L.	Nigral dynorphin release studied with in vivo microdialysis in rat	1994	54	339-340	Regul Pept
You, Z. B.; Herrera-Marschitz, M.; Nylander, I.; Goiny, M.; Connor, W. T.; Ungerstedt, U.; Terenius, L.	The striatonigral dynorphin pathway of the rat studied with in vivo microdialysis--II. Effects of dopamine D1 and D2 receptor agonists	1994	63	427-434	Neuroscience
You, Z. B.; Herrera-Marschitz, M.; Nylander, I.; Goiny, M.; Kehr, J.; Ungerstedt, U.; Terenius, L.	Effect of morphine on dynorphin B and GABA release in the basal ganglia of rats	1996	710	241-248	Brain Res
You, Z. B.; Herrera-Marschitz, M.; Pettersson, E.; Nylander, I.; Goiny, M.; Shou, H. Z.; Kehr, J.; Godukhin, O.; Hokfelt, T.; Terenius, L.; Ungerstedt, U.	Modulation of neurotransmitter release by cholecystokinin in the neostriatum and substantia nigra of the rat: regional and receptor specificity	1996	74	783-804	Neuroscience
You, Z. B.; Nylander, I.; Herrera-Marschitz, M.; Connor, W. T.; Goiny, M.; Terenius, L.	The striatonigral dynorphin pathway of the rat studied with in vivo microdialysis--I. Effects of K(+)-depolarization, lesions and peptidase inhibition	1994	63	415-425	Neuroscience
You, Z. B.; Pettersson, E.; Herrera-Marschitz, M.; Hokfelt, T.; Terenius, L.; Nylander, I.; Goiny, M.; Hughes, J.; Connor, W. T.; Ungerstedt, U.	Modulation of striatal aspartate and dynorphin B release by cholecystokinin (CCK-8) studied in vivo with microdialysis	1994	5	2301-2304	Neuroreport
You, Z. B.; Saria, A.; Fischer-Colbrie, R.; Terenius, L.; Goiny, M.; Herrera-Marschitz, M.	Effects of secretogranin II-derived peptides on the release of neurotransmitters monitored in the basal ganglia of the rat with in vivo microdialysis	1996	354	717-724	Naunyn Schmiedebergs Arch Pharmacol
You, Z. B.; Wang, B.; Zitzman, D.; Azari, S.; Wise, R. A.	A role for conditioned ventral tegmental glutamate release in cocaine seeking	2007	27	10546-10555	J Neurosci
Young, A. M.; Bradford, H. F.	N-methyl-D-aspartate releases gamma-aminobutyric acid from rat striatum in vivo: a microdialysis study using a novel preloading method	1993	60	487-492	J Neurochem
Young, A. M.; Foley, P. M.; Bradford, H. F.	Preloading in vivo: a rapid and reliable method for measuring gamma-aminobutyric acid and glutamate fluxes by microdialysis	1990	55	1060-1063	J Neurochem
Young, B. J.; Cook, C. J.	Reduced basal GABA concentrations in the rat amygdala during pregnancy	2006	87	817-820	Physiol Behav

GABA					
**Authors**	**Title**	**Year**	**Vol**	**Pages**	**Journal**
Young, R. S.; During, M. J.; Aquila, W. J.; Tendler, D.; Ley, E.	Hypoxia increases extracellular concentrations of excitatory and inhibitory neurotransmitters in subsequently induced seizure: in vivo microdialysis study in the rabbit	1992	117	204-209	Exp Neurol
Zeng, X.; Zhang, Y.; Zhang, S.; Zheng, X.	A microdialysis study of effects of gastrodin on neurochemical changes in the ischemic/reperfused rat cerebral hippocampus	2007	30	801-804	Bio Pharm Bull
Zestos, A. G.; Mikelman, S. R.; Kennedy, R. T.; Gnegy, M. E.	PKCbeta Inhibitors Attenuate Amphetamine-Stimulated Dopamine Efflux	2016	7	757-766	ASC Chem Neurosci
Zhang, B.; Chu, J.; Zhang, J.; Ma, Y.	Change of extracellular glutamate and gamma-aminobutyric acid in substantia nigra and globus pallidus during electrical stimulation of subthalamic nucleus in epileptic rats	2008	86	208-215	Stereotact Funct Neurosurg
Zhang, C. Y.; Du, G. Y.; Wang, W.; Ye, Z. G.; Wang, D. Q.; Sun, X. F.; Zhao, D. Z.	[Effects of tianma gouteng fang on transmitter amino acids in the hippocampus extracellular liquids in freely moving rats subjected to brain ischemia]	2004	29	1061-1065	Zhongguo Zhong Yao Za Zhi
Zhang, D.; Zhang, J.; Ma, W.; Chen, D.; Han, H.; Shu, H.; Liu, G.	Analysis of trace amino acid neurotransmitters in hypothalamus of rats after exhausting exercise using microdialysis	2001	758	277-282	J Chromatogr B Biomed Sci Appl
Zhang, J.; Niu, X.	Changes of monoamines, purines and amino acids in rat striatum as measured by intercerebral microdialysis during ischemia/reperfusion	1994	9	225-229	Chin Med Sci J
Zhang, N.; Liu, P.; He, X.	[Effect of single-used borneol and combining it with diazepam on content of neurotransmitter in corpus striatum of rats]	2011	36	3180-3183	Zhongguo Zhong Yao Za Zhi
Zhang, N.; Liu, P.; He, X.	Effect of single-use versus combined-use moschus and diazepam on expression of amino acid neurotransmitters in the rat corpus striatum	2012	7	182-186	Neural Regen Res
Zhang, N.; Liu, P.; He, X.	Effect of borneol, moschus, storax, and acorus tatarinowii on expression levels of four amino acid neurotransmitters in the rat corpus striatum	2012	7	440-444	Neural Regen Res
Zhang, S.; Takeda, Y.; Hagioka, S.; Goto, K.; Morita, K.	The close relationship between decreases in extracellular GABA concentrations and increases in the incidence of hyperbaric oxygen-induced electrical discharge	2004	58	91-95	Acta Med Okayama
Zhang, S.; Takeda, Y.; Hagioka, S.; Takata, K.; Aoe, H.; Nakatsuka, H.; Yokoyama, M.; Morita, K.	Measurement of GABA and glutamate in vivo levels with high sensitivity and frequency	2005	14	61-66	Brain Res Brain Res Protoc
Zhang, W. Q.; Rogers, B. C.; Tandon,	Systemic administration of kainic acid increases GABA levels in	1990	11	593-	Neurotoxicology

GABA					
Authors	Title	Year	Vol	Pages	Journal
P.; Hudson, P. M.; Sobotka, T. J.; Hong, J. S.; Tilson, H. A.	perfusate from the hippocampus of rats in vivo			600	
Zhang, W.; Wei, L.; Yu, W.; Cui, X.; Liu, X.; Wang, Q.; Wang, S.	Effect of Jian-Pi-Zhi-Dong Decoction on striatal glutamate and gamma-aminobutyric acid levels detected using microdialysis in a rat model of Tourette syndrome	2016	12	1233-1242	Neuropsychiatr Dis Treat
Zhang, X.; Rauch, A.; Lee, H.; Xiao, H.; Rainer, G.; Logothetis, N. K.	Capillary hydrophilic interaction chromatography/mass spectrometry for simultaneous determination of multiple neurotransmitters in primate cerebral cortex	2007	21	3621-3628	Rapid Commun Mass Spectrom
Zhao, P.; Cheng, J.	Effects of electroacupuncture on extracellular contents of amino acid neurotransmitters in rat striatum following transient focal cerebral ischemia	1997	22	119-126	Acupunct Electrother Res
Zhao, W. H.; Huang, H. L.; Huang, Y.; Xu, X.; Mo, L. D.	Experimental study on the correlation between the cerebral energy metabolism and cerebral blood flow changes in rats with subarachnoid hemorrhage-induced delayed cerebral vasospasm. [Chinese]	2007	7	146-151	Zhongguo Xian Dai Shen Jing Ji Bing Za Zhi
Zhao, X. D.; Zhou, X. P.; Liu, H. H.; Li, B. M.; Hu, X. W.; Li, F. Q.; You, B. M.	[Changes of amino acids neurotransmitters in striatum of hemi-parkinsonian rhesus monkey after high frequency stimulation of subthalamic nucleus]	2007	45	1682-1684	Zhonghua Wai Ke Za Zhi
Zhong, C.; Zhao, X.; Van, K. C.; Bzdega, T.; Smyth, A.; Zhou, J.; Kozikowski, A. P.; Jiang, J.; Connor, W. T.; Berman, R. F.; Neale, J. H.; Lyeth, B. G.	NAAG peptidase inhibitor increases dialysate NAAG and reduces glutamate, aspartate and GABA levels in the dorsal hippocampus following fluid percussion injury in the rat	2006	97	1015-1025	J Neurochem
Zhong, M.; Chen, H.; Jiang, Y.; Tu, P. F.; Liu, C. L.; Zhang, W. X.; Ma, J. Y.; Ding, H.	Effects of echinacoside on striatal extracellular levels of amino acid neurotransmitter in cerebral ischemia rats. [Chinese]	2012	28	361-365	Zhongguo yao li xue tong bao
Zhou, Q. M.; Hu, G.; Ding, J. H.; Zeng, Y. M.	Aquaporin-4 deficiency changes propofol-induced hypnotic effects in mice. [Chinese]	2011	27	440-441	Zhongguo yao li xue tong bao
Zhou, Q. M.; Lu, Y. L.; Hu, G.; Zeng, Y. M.; Tu, W. F.	Effect of aquaporin-4 deficiency on intravenous anaesthetic induced hypnotic effects in mice	2013	65	569-576	Sheng Li Xue Bao
Zhu, D. N.; Moriguchi, A.; Mikami, H.; Higaki, J.; Ogihara, T.	Central amino acids mediate cardiovascular response to angiotensin II in the rat	1998	45	189-197	Brain Res Bull
Zhu, G.; Okada, M.; Murakami, T.;	Dysfunction of M-channel enhances propagation of neuronal	2000	294	53-57	Neurosci Lett

GABA					
**Authors**	**Title**	**Year**	**Vol**	**Pages**	**Journal**
Kamata, A.; Kawata, Y.; Wada, K.; Kaneko, S.	excitability in rat hippocampus monitored by multielectrode dish and microdialysis systems				
Zhu, G.; Okada, M.; Uchiyama, D.; Ohkubo, T.; Yoshida, S.; Kaneko, S.	Hyperactivity of endoplasmic reticulum associated exocytosis mechanism contributes to acute phencyclidine intoxication	2004	95	214-227	J Pharmacol Sci
Zhu, W.; Czyzyk, D.; Paranjape, S. A.; Zhou, L.; Horblitt, A.; Szabo, G.; Seashore, M. R.; Sherwin, R. S.; Chan, O.	Glucose prevents the fall in ventromedial hypothalamic GABA that is required for full activation of glucose counterregulatory responses during hypoglycemia	2010	298	E971-E977	Am J Physiol Endocrinol Metab
Zuiderwijk, M.; Ghijsen, W. E.	Monitoring amino acid neurotransmitter release in the brain by in vivo microdialysis	1997	72	239-250	Methods Mol Biol
Zuiderwijk, M.; Veenstra, E.; Lopes da Silva, F. H.; Ghijsen, W. E.	Effects of uptake carrier blockers SK & F 89976-A and L-trans-PDC on in vivo release of amino acids in rat hippocampus	1996	307	275-282	Eur J Pharmacol

Appendix 4: Studies describing baseline dialysate concentrations for glutamine

Glutamine					
**Authors**	**Title**	**Year**	**Vol.**	**Pages**	**Journal**
Abekawa, T.	[Experimental study of methamphetamine psychosis--role of glutamate and nitric oxide in methamphetamine-induced dopaminergic and serotonergic neurotoxicity in the rat brain]	1997	72	113-126	Hokkaido Igaku Zasshi
Abekawa, T.; Ohmori, T.; Koyama, T.	Effects of repeated administration of a high dose of methamphetamine on dopamine and glutamate release in rat striatum and nucleus accumbens	1994	643	276-281	Brain Res
Abraham, I.; Juhasz, G.; Kekesi, K. A.; Kovacs, K. J.	Effect of intrahippocampal dexamethasone on the levels of amino acid transmitters and neuronal excitability	1996	733	56-63	Brain Res
Abraham, I.; Juhasz, G.; Kekesi, K. A.; Kovacs, K. J.	Corticosterone peak is responsible for stress-induced elevation of glutamate in the hippocampus	1998	2	171-181	Stress
Acworth, I. N.; Yu, J.; Ryan, E.; Gariepy, K. C.; Gamache, P.; Hull, K.; Maher, T.	Simultaneous measurement of monoamine, amino acid, and drug levels, using high performance liquid chromatography and coulometric array technology: Application to in vivo microdialysis perfusate analysis	1994	17	685-705	J Liq Chromatogr
Alexander, G. M.; Deitch, J. S.; Seeburger, J. L.; Del Valle, L.; Heiman-Patterson, T. D.	Elevated cortical extracellular fluid glutamate in transgenic mice expressing human mutant (G93A) Cu/Zn superoxide dismutase	2000	74	1666-1673	J Neurochem
Alexander, G. M.; Grothusen, J. R.; Gordon, S. W.; Schwartzman, R. J.	Intracerebral microdialysis study of glutamate reuptake in awake, behaving rats	1997	766	1-10	Brain Res
Amantea, D.; Fratto, V.; Maida, S.; Rotiroti, D.; Ragusa, S.; Nappi, G.; Bagetta, G.; Corasaniti, M. T.	Prevention of Glutamate Accumulation and Upregulation of Phospho-Akt may Account for Neuroprotection Afforded by Bergamot Essential Oil against Brain Injury Induced by Focal Cerebral Ischemia in Rat	2009	85	389-405	Int Rev Neurobiol
Andine, P.; Orwar, O.; Jacobson, I.; Sandberg, M.; Hagberg, H.	Changes in extracellular amino acids and spontaneous neuronal activity during ischemia and extended reflow in the CA1 of the rat hippocampus	1991	57	222-229	J Neurochem
Arvin, B.; Moncada, C.; Le Peillet, E.; Chapman, A.; Meldrum, B. S.	GYKI 52466 blocks the increase in extracellular glutamate induced by ischaemia	1992	3	235-238	Neuroreport
Bakkelund, A. H.; Fonnum, F.; Paulsen, R. E.	Evidence using in vivo microdialysis that aminotransferase activities are important in the regulation of the pools of	1993	18	411-415	Neurochem Res

Glutamine					
**Authors**	**Title**	**Year**	**Vol.**	**Pages**	**Journal**
	transmitter amino acids				
Behrens, P. F.; Franz, P.; Woodman, B.; Lindenberg, K. S.; Landwehrmeyer, G. B.	Impaired glutamate transport and glutamate-glutamine cycling: downstream effects of the Huntington mutation	2002	125	1908-1922	Brain
Behrens, P. F.; Langemann, H.; Strohschein, R.; Draeger, J.; Hennig, J.	Extracellular glutamate and other metabolites in and around RG2 rat glioma: an intracerebral microdialysis study	2000	47	11-22	J Neurooncol
Benveniste, H.; Drejer, J.; Schousboe, A.; Diemer, N. H.	Elevation of the extracellular concentrations of glutamate and aspartate in rat hippocampus during transient cerebral ischemia monitored by intracerebral microdialysis	1984	43	1369-1374	J Neurochem
Bereiter, D. A.; Shen, S.; Benetti, A. P.	Sex differences in amino acid release from rostral trigeminal subnucleus caudalis after acute injury to the TMJ region	2002	98	89-99	Pain
Bergquist, F.; Ludwig, M.; Dutia, M. B.	Neuroscience I: Commissural GABA release during vestibular compensation in the rat	2009	37	S28	Amino Acids
Bergquist, F.; Ludwig, M.; Dutia, M. B.	Role of the commissural inhibitory system in vestibular compensation in the rat	2008	586	4441-4452	J Physiol
Bergquist, J.; Vona, M. J.; Stiller, C. O.; Connor, W. T.; Falkenberg, T.; Ekman, R.	Capillary electrophoresis with laser-induced fluorescence detection: a sensitive method for monitoring extracellular concentrations of amino acids in the periaqueductal grey matter	1996	65	33-42	J Neurosci Methods
Biggs, C. S.; Pearce, B. R.; Fowler, L. J.; Whitton, P. S.	Effect of isonicotinic acid hydrazide on extracellular amino acids and convulsions in the rat: Reversal of neurochemical and behavioural deficits by sodium valproate	1994	63	2197-2201	J Neurochem
Biggs, C. S.; Pearce, B. R.; Fowler, L. J.; Whitton, P. S.	The effect of sodium valproate on extracellular GABA and other amino acids in the rat ventral hippocampus: an in vivo microdialysis study	1992	594	138-142	Brain Res
Boatell, M. L.; Bendahan, G.; Mahy, N.	Time-related cortical amino acid changes after basal forebrain lesion: a microdialysis study	1995	64	285-291	J Neurochem
Bockelmann, R.; Reiser, M.; Horn, T. F.; Wolf, G.	Influence of nitric oxide synthase activity on amino acid concentration in the quinolinate lesioned rat striatum: a microdialysis and histochemical study	2000	19	423-437	Amino Acids

Glutamine					
**Authors**	**Title**	**Year**	**Vol.**	**Pages**	**Journal**
Bosman, D. K.; Deutz, N. E.; Maas, M. A.; van Eijk, H. M.; Smit, J. J.; de Haan, J. G.; Chamuleau, R. A.	Amino acid release from cerebral cortex in experimental acute liver failure, studied by in vivo cerebral cortex microdialysis	1992	59	591-599	J Neurochem
Bottcher, T.; Goiny, M.; Bering, J.; Domhof, S.; Nau, R.; Ungerstedt, U.	Regional differences in glutamine synthetase inhibition by L-methionine sulfoximine: a microdialysis study in the rabbit brain	2003	150	194-200	Exp Brain Res
Bradford, H. F.; Young, A. M.; Crowder, J. M.	Continuous glutamate leakage from brain cells is balanced by compensatory high-affinity reuptake transport	1987	81	296-302	Neurosci Lett
Brodie, M. E.; Opacka-Juffry, J.; Peterson, D. W.; Brown, A. W.	Neurochemical changes in hippocampal and caudate dialysates associated with early trimethyltin neurotoxicity in rats	1990	11	35-46	Neurotoxicology
Brown, A. M.; Skamarauskas, J.; Lister, T.; Madjd, A.; Ray, D. E.	Differential susceptibility of astrocytic and neuronal function to 3-chloropropanediol in the rat inferior colliculus	2011	116	996-1004	J Neurochem
Bruhn, T.; Christensen, T.; Diemer, N. H.	In vivo cellular uptake of glutamate is impaired in the rat hippocampus during and after transient cerebral ischemia: a microdialysis extraction study	2001	66	1118-1126	J Neurosci Res
Bruhn, T.; Cobo, M.; Berg, M.; Diemer, N. H.	Limbic seizure-induced changes in extracellular amino acid levels in the hippocampal formation: a microdialysis study of freely moving rats	1992	86	455-461	Acta Neurol Scand
Brust, P.; Christensen, T.; Diemer, N. H.	Decrease of extracellular taurine in the rat dorsal hippocampus after central nervous administration of vasopressin	1992	58	1427-1431	J Neurochem
Buisson, A.; Callebert, J.; Mathieu, E.; Plotkine, M.; Boulu, R. G.	Striatal protection induced by lesioning the substantia nigra of rats subjected to focal ischemia	1992	59	1153-1157	J Neurochem
Buratta, S.; Hamberger, A.; Ryberg, H.; Nystrom, B.; Sandberg, M.; Mozzi, R.	Effect of serine and ethanolamine administration on phospholipid-related compounds and neurotransmitter amino acids in the rabbit hippocampus	1998	71	2145-2150	J Neurochem
Butcher, S. P.; Cameron, D.; Kendall, L.; Griffiths, R.	Homocysteine-induced alterations in extracellular amino acids in rat hippocampus	1992	20	75-80	Neurochem Int
Butcher, S. P.; Hamberger, A.	In vivo studies on the extracellular, and veratrine-releasable, pools of endogenous amino acids in the rat striatum: effects of corticostriatal deafferentation and kainic acid lesion	1987	48	713-721	J Neurochem

Glutamine					
**Authors**	**Title**	**Year**	**Vol.**	**Pages**	**Journal**
Butcher, S. P.; Jacobson, I.; Hamberger, A.	On the epileptogenic effects of kainic acid and dihydrokainic acid in the dentate gyrus of the rat	1988	27	375-381	Neuropharmacology
Butcher, S. P.; Lazarewicz, J. W.; Hamberger, A.	In vivo microdialysis studies on the effects of decortication and excitotoxic lesions on kainic acid-induced calcium fluxes, and endogenous amino acid release, in the rat striatum	1987	49	1355-1360	J Neurochem
Butcher, S. P.; Sandberg, M.; Hagberg, H.; Hamberger, A.	Cellular origins of endogenous amino acids released into the extracellular fluid of the rat striatum during severe insulin-induced hypoglycemia	1987	48	722-728	J Neurochem
Camacho, A.; Montiel, T.; Massieu, L.	The anion channel blocker, 4,4'-dinitrostilbene-2,2'-disulfonic acid prevents neuronal death and excitatory amino acid release during glycolysis inhibition in the hippocampus in vivo	2006	143	1005-1017	Neuroscience
Cassel, G. E.; Fosbraey, P.	Measurement of the oxime HI-6 after peripheral administration in tandem with neurotransmitter levels in striatal dialysates: effects of soman intoxication	1996	35	159-166	J Pharmacol Toxicol Methods
Chen, Y. L.; Li, D.; Wang, Z. Z.; Xu, W. G.; Li, R. P.; Zhang, J. D.	Glutamate metabolism of astrocytes during hyperbaric oxygen exposure and its effects on central nervous system oxygen toxicity	2016	27	73-79	Neuroreport
Choi, Y. H.; Chang, N.; Fletcher, P. J.; Anderson, G. H.	Dietary protein content affects the profiles of extracellular amino acids in the medial preoptic area of freely moving rats	2000	66	1105-1118	Life Sci
Choi, Y. H.; Fletcher, P. J.; Anderson, G. H.	Extracellular amino acid profiles in the paraventricular nucleus of the rat hypothalamus are influenced by diet composition	2001	892	320-328	Brain Res
Choi, Y. H.; Fletcher, P. J.; Wong, C. C.; Anderson, G. H.	Measurement of blood-brain barrier permeability of rats with alpha-aminoisobutyric acid during microdialysis: possible application to behavioral studies	1999	67	587-598	Physiol Behav
Cobo, M.; Bruhn, T.; Berg, M.; Diemer, N. H.	Phenylsuccinate reduces KCL-induced release of GABA evidence for the participation of the ketodicarboxylate carrier in the biosynthesis of transmitter-GABA	1993	5	377-388	Amino Acids
Currie, P. J.; Chang, N.; Luo, S.; Anderson, G. H.	Microdialysis as a tool to measure dietary and regional effects on the complete profile of extracellular amino acids	1995	57	1911-1923	Life Sci

Glutamine					
**Authors**	**Title**	**Year**	**Vol.**	**Pages**	**Journal**
	in the hypothalamus of rats				
Daisley, J. N.; Gruss, M.; Rose, S. P.; Braun, K.	Passive avoidance training and recall are associated with increased glutamate levels in the intermediate medial hyperstriatum ventrale of the day-old chick	1998	6	53-61	Neural Plast
Darbin, O.; Risso, J. J.; Rostain, J. C.	Helium-oxygen pressure induces striatal glutamate increase: a microdialysis study in freely-moving rats	2001	297	37-40	Neurosci Lett
David, C. N.; Frias, E. S.; Szu, J. I.; Vieira, P. A.; Hubbard, J. A.; Lovelace, J.; Michael, M.; Worth, D.; McGovern, K. E.; Ethell, I. M.; Stanley, B. G.; Korzus, E.; Fiacco, T. A.; Binder, D. K.; Wilson, E. H.	GLT-1-Dependent Disruption of CNS Glutamate Homeostasis and Neuronal Function by the Protozoan Parasite Toxoplasma gondii	2016	12	e1005643	PLoS Pathog
Dawson, L. A.; Organ, A. J.; Winter, P.; Lacroix, L. P.; Shilliam, C. S.; Heidbreder, C.; Shah, A. J.	Rapid high-throughput assay for the measurement of amino acids from microdialysates and brain tissue using monolithic C18-bonded reversed-phase columns	2004	807	235-241	J Chromatogr B Analyt Technol Biomed Life Sci
de Knegt, R. J.; Schalm, S. W.; van der Rijt, C. C.; Fekkes, D.; Dalm, E.; Hekking-Weyma, I.	Extracellular brain glutamate during acute liver failure and during acute hyperammonemia simulating acute liver failure: an experimental study based on in vivo brain dialysis	1994	20	19-26	J Hepatol
De Novellis, V.; Marabese, I.; Palazzo, E.; Berrino, L.; Rodella, L.; Bianchi, R.; Rossi, F.; Maione, S.	Group I metabotropic glutamate receptors modulate glutamate and gamma-aminobutyric acid release in the periaqueductal grey of rats	2003	462	73-81	Eur J Pharmacol
de Novellis, V.; Negri, L.; Lattanzi, R.; Rossi, F.; Palazzo, E.; Marabese, I.; Giannini, E.; Vita, D.; Melchiorri, P.; Maione, S.	The prokineticin receptor agonist Bv8 increases GABA release in the periaqueductal grey and modifies RVM cell activities and thermoceptive reflexes in the rat	2007	26	3068-3078	Eur J Neurosci
Del Arco, A.; Castaneda, T. R.; Mora, F.	Amphetamine releases GABA in striatum of the freely moving rat: involvement of calcium and high affinity transporter mechanisms	1998	37	199-205	Neuropharmacology
Del Arco, A.; Gonzalez-Mora, J. L.; Armas, V. R.; Mora, F.	Amphetamine increases the extracellular concentration of glutamate in striatum of the awake rat: involvement of high affinity transporter mechanisms	1999	38	943-954	Neuropharmacology

Glutamine					
**Authors**	**Title**	**Year**	**Vol.**	**Pages**	**Journal**
Del Arco, A.; Martinez, R.; Mora, F.	Amphetamine increases extracellular concentrations of glutamate in the prefrontal cortex of the awake rat: a microdialysis study	1998	23	1153-1158	Neurochem Res
Delgado, J. M.; DeFeudis, F. V.; Roth, R. H.; Ryugo, D. K.; Mitruka, B. M.	Dialytrode for long term intracerebral perfusion in awake monkeys	1972	198	9-21	Arch Int Pharmacodyn Ther
Delgado, J. M.; Lerma, J.; Martin del Rio, R.; Solis, J. M.	Dialytrode technology and local profiles of amino acids in the awake cat brain	1984	42	1218-1228	J Neurochem
Devall, A. J.; Blake, R.; Langman, N.; Smith, C. G.; Richards, D. A.; Whitehead, K. J.	Monolithic column-based reversed-phase liquid chromatography separation for amino acid assay in microdialysates and cerebral spinal fluid	2007	848	323-328	J Chromatogr B Analyt Technol Biomed Life Sci
Di Cara, B.; Dusticier, N.; Forni, C.; Lievens, J. C.; Daszuta, A.	Serotonin depletion produces long lasting increase in striatal glutamatergic transmission	2001	78	240-248	J Neurochem
Dolgodilina, E.; Imobersteg, S.; Laczko, E.; Welt, T.; Verrey, F.; Makrides, V.	Brain interstitial fluid glutamine homeostasis is controlled by blood-brain barrier SLC7A5/LAT1 amino acid transporter	2016	36	1929-1941	J Cereb Blood Flow Metab
Espey, M. G.; Kustova, Y.; Sei, Y.; Basile, A. S.	Extracellular glutamate levels are chronically elevated in the brains of LP-BM5-infected mice: a mechanism of retrovirus-induced encephalopathy	1998	71	2079-2087	J Neurochem
Fallgren, A. B.; Paulsen, R. E.	A microdialysis study in rat brain of dihydrokainate, a glutamate uptake inhibitor	1996	21	19-25	Neurochem Res
Fedele, E.; Foster, A. C.	An evaluation of the role of extracellular amino acids in the delayed neurodegeneration induced by quinolinic acid in the rat striatum	1993	52	911-917	Neuroscience
Fedele, E.; Varnier, G.; Ansaldo, M. A.; Raiteri, M.	Nicotine administration stimulates the in vivo N-methyl-D-aspartate receptor/nitric oxide/cyclic GMP pathway in rat hippocampus through glutamate release	1998	125	1042-1048	Br J Pharmacol
Fraser, M.; Bennet, L.; Van Zijl, P. L.; Mocatta, T. J.; Williams, C. E.; Gluckman, P. D.; Winterbourn, C. C.; Gunn, A. J.	Extracellular amino acids and lipid peroxidation products in periventricular white matter during and after cerebral ischemia in preterm fetal sheep	2008	105	2214-2223	J Neurochem
Fujikawa, D. G.	Effects of N-methyl-D-aspartate-receptor blockade on high-potassium-induced neuronal death and glutamate	1997	226	25-28	Neurosci Lett

Glutamine					
**Authors**	**Title**	**Year**	**Vol.**	**Pages**	**Journal**
	release				
Fujikawa, D. G.; Kim, J. S.; Daniels, A. H.; Alcaraz, A. F.; Sohn, T. B.	In vivo elevation of extracellular potassium in the rat amygdala increases extracellular glutamate and aspartate and damages neurons	1996	74	695-706	Neuroscience
Fumero, B.; Guadalupe, T.; Valladares, F.; Mora, F.; Neill, R. D.; Mas, M.; Gonzalez-Mora, J. L.	Fixed versus removable microdialysis probes for in vivo neurochemical analysis: implications for behavioral studies	1994	63	1407-1415	J Neurochem
Gemba, T.; Matsunaga, K.; Ueda, M.	Changes in extracellular concentration of amino acids in the hippocampus during cerebral ischemia in stroke-prone SHR, stroke-resistant SHR and normotensive rats	1992	135	184-188	Neurosci Lett
Glass, J. D.; Hauser, U. E.; Blank, J. L.; Selim, M.; Rea, M. A.	Differential timing of amino acid and 5-HIAA rhythms in suprachiasmatic hypothalamus	1993	265	R504-R511	Am J Physiol
Gobert, A.; Rivet, J. M.; Billiras, R.; Parsons, F.; Millan, M. J.	Simultaneous quantification of D- vs. L-serine, taurine, kynurenate, phosphoethanolamine and diverse amino acids in frontocortical dialysates of freely-moving rats: differential modulation by N-methyl-D-aspartate (NMDA) and other pharmacological agents	2011	202	143-157	J Neurosci Methods
Goldsmith, J. D.; Kujawa, S. G.; McLaren, J. D.; Bledsoe Jr, S. C.	In vivo release of neuroactive amino acids from the inferior colliculus of the guinea pig using brain microdialysis	1995	83	80-88	Hear Res
Gordon, K. E.; Simpson, J.; Statman, D.; Silverstein, F. S.	Effects of perinatal stroke on striatal amino acid efflux in rats studied with in vivo microdialysis	1991	22	928-932	Stroke
Gruenbaum, S.; Dhaher, R.; Eid, T.	The role of increased branched-chain amino acids in the blood on brain extracellular fluid glutamate concentrations in naive rats	2015	27(4)	407-408	J Neurosurg Anesthesiol
Gruss, M.; Braun, K.	Stimulus-evoked increase of glutamate in the mediorostral neostriatum/hyperstriatum ventrale of domestic chick after auditory filial imprinting: an in vivo microdialysis study	1996	66	1167-1173	J Neurochem
Guo, Y.; Cai, H.; Chen, L.; Liang, D.; Yang, R.; Dang, R.; Jiang, P.	Quantitative profiling of neurotransmitter abnormalities in the hippocampus of rats treated with lipopolysaccharide: Focusing on kynurenine pathway and implications for depression	2016	295-296	41-46	J Neuroimmunol
Gwag, B. J.; Sessler, F. M.; Robine, V.; Springer, J. E.	Endogenous glutamate levels regulate nerve growth factor mRNA expression in the rat dentate gyrus	1997	7	425-430	Mol Cells

Glutamine					
**Authors**	**Title**	**Year**	**Vol.**	**Pages**	**Journal**
Hamann, M.; Sohr, R.; Morgenstern, R.; Richter, A.	Extracellular amino acid levels in the striatum of the dt(sz) mutant, a model of paroxysmal dystonia	2008	157	188-195	Neuroscience
Hasegawa, T.; Azum, S.; Inoue, S.	Amino acid release from the rat oral pontine reticular nucleus across the sleep-wakefulness cycle	2000	47	87-93	J Med Dent Sci
Hashimoto, K.; Fujita, Y.; Ishima, T.; Chaki, S.; Iyo, M.	Phencyclidine-induced cognitive deficits in mice are improved by subsequent subchronic administration of the glycine transporter-1 inhibitor NFPS and D-serine	2008	18	414-221	Eur Neuropsychopharmacol
Haskew-Layton, R. E.; Rudkouskaya, A.; Jin, Y.; Feustel, P. J.; Kimelberg, H. K.; Mongin, A. A.	Two distinct modes of hypoosmotic medium-induced release of excitatory amino acids and taurine in the rat brain in vivo	2008	3	e3543	PLoS One
Hassel, B.; Dahlberg, D.; Mariussen, E.; Goverud, I. L.; Antal, E. A.; Tonjum, T.; Maehlen, J.	Brain infection with Staphylococcus aureus leads to high extracellular levels of glutamate, aspartate, gamma-aminobutyric acid, and zinc	2014	92	1792-1800	J Neurosci Res
Hemmati, P.; Shilliam, C. S.; Hughes, Z. A.; Shah, A. J.; Roberts, J. C.; Atkins, A. R.; Hunter, A. J.; Heidbreder, C. A.	In vivo characterization of basal amino acid levels in subregions of the rat nucleus accumbens: effect of a dopamine D(3)/D(2) agonist	2001	39	199-208	Neurochem Int
Hilgier, W.; Lazarewicz, J. W.; Struzynska, L.; Frontczak-Baniewicz, M.; Albrecht, J.	Repeated exposure of adult rats to Aroclor 1254 induces neuronal injury and impairs the neurochemical manifestations of the NMDA receptor-mediated intracellular signaling in the hippocampus	2012	33	16-22	Neurotoxicology
Hilgier, W.; Zielinska, M.; Borkowska, H. D.; Gadamski, R.; Walski, M.; Oja, S. S.; Saransaari, P.; Albrecht, J.	Changes in the extracellular profiles of neuroactive amino acids in the rat striatum at the asymptomatic stage of hepatic failure	1999	56	76-84	J Neurosci Res
Hillered, L.; Hallstrom, A.; Segersvard, S.; Persson, L.; Ungerstedt, U.	Dynamics of extracellular metabolites in the striatum after middle cerebral artery occlusion in the rat monitored by intracerebral microdialysis	1989	9	607-616	J Cereb Blood Flow Metab
Horio, M.; Kohno, M.; Fujita, Y.; Ishima, T.; Inoue, R.; Mori, H.; Hashimoto, K.	Levels of D-serine in the brain and peripheral organs of serine racemase (Srr) knock-out mice	2011	59	853-859	Neurochem Int

Glutamine					
**Authors**	**Title**	**Year**	**Vol.**	**Pages**	**Journal**
Horn, T.; Bauce, L.; Landgraf, R.; Pittman, Q. J.	Microdialysis with high NaCl causes central release of amino acids and dopamine	1995	64	1632-1644	J Neurochem
Huo, T.; Zhang, Y.; Li, W.; Yang, H.; Jiang, H.; Sun, G.	Effect of realgar on extracellular amino acid neurotransmitters in hippocampal CA1 region determined by online microdialysis-dansyl chloride derivatization-high-performance liquid chromatography and fluorescence detection	2014	28	1254-1262	Biomed Chromatogr
Hwa, L. S.; Nathanson, A. J.; Shimamoto, A.; Tayeh, J. K.; Wilens, A. R.; Holly, E. N.; Newman, E. L.; DeBold, J. F.; Miczek, K. A.	Aggression and increased glutamate in the mPFC during withdrawal from intermittent alcohol in outbred mice	2015	232	2889-2902	Psychopharmacology (Berl)
Hylland, P.; Nilsson, G. E.	Extracellular levels of amino acid neurotransmitters during anoxia and forced energy deficiency in crucian carp brain	1999	823	49-58	Brain Res
Hylland, P.; Nilsson, G. E.; Johansson, D.	Anoxic brain failure in an ectothermic vertebrate: release of amino acids and K+ in rainbow trout thalamus	1995	269	R1077-R1084	Am J Physiol
Hyzinski-Garcia, M. C.; Vincent, M. Y.; Haskew-Layton, R. E.; Dohare, P.; Keller Jr, R. W.; Mongin, A. A.	Hypo-osmotic swelling modifies glutamate-glutamine cycle in the cerebral cortex and in astrocyte cultures	2011	118	140-152	J Neurochem
Ichord, R. N.; Northington, F. J.; van Wylen, D.; Johnston, M. V.; Kwon, C.; Traystman, R. J.	Brain O2 consumption and glutamate release during hypoglycemic coma in piglets are temperature sensitive	1999	276	H2053-H2062	Am J Physiol
Ishiwata, S.; Umino, A.; Balu, D. T.; Coyle, J. T.; Nishikawa, T.	Neuronal serine racemase regulates extracellular D-serine levels in the adult mouse hippocampus	2015	122	1099-1103	J Neural Transm (Vienna)
Itoh, T.; Saito, T.; Fujimura, M.; Watanabe, S.; Saito, K.	Restraint stress-induced changes in endogenous zinc release from the rat hippocampus	1993	618	318-322	Brain Res
Jacobson, I.; Hagberg, H.; Sandberg, M.; Hamberger, A.	Ouabain-induced changes in extracellular aspartate, glutamate and GABA levels in the rabbit olfactory bulb in vivo	1986	64	211-215	Neurosci Lett
Jacobson, I.; Sandberg, M.; Hamberger, A.	Mass transfer in brain dialysis devices--a new method for the estimation of extracellular amino acids concentration	1985	15	263-268	J Neurosci Methods
Kanamori, K.	Disinhibition reduces extracellular glutamine and elevates extracellular glutamate in rat hippocampus in vivo	2015	114	32-46	Epilepsy Res

Glutamine					
**Authors**	**Title**	**Year**	**Vol.**	**Pages**	**Journal**
Kanamori, K.; Ross, B. D.	Electrographic seizures are significantly reduced by in vivo inhibition of neuronal uptake of extracellular glutamine in rat hippocampus	2013	107	20-36	Epilepsy Res
Kanamori, K.; Ross, B. D.	Suppression of glial glutamine release to the extracellular fluid studied in vivo by NMR and microdialysis in hyperammonemic rat brain	2005	94	74-85	J Neurochem
Kanamori, K.; Ross, B. D.	Quantitative determination of extracellular glutamine concentration in rat brain, and its elevation in vivo by system A transport inhibitor, alpha-(methylamino)isobutyrate	2004	90	203-210	J Neurochem
Kapoor, V.; Minson, J.; Chalmers, J.	Ventral medulla stimulation increases blood pressure and spinal cord amino acid release	1992	3	55-58	Neuroreport
Kasper, J. M.; Booth, R. G.; Peris, J.	Serotonin-2C receptor agonists decrease potassium-stimulated GABA release in the nucleus accumbens	2015	69	78-85	Synapse
Kaura, S.; Bradford, H. F.; Young, A. M.; Croucher, M. J.; Hughes, P. D.	Effect of amygdaloid kindling on the content and release of amino acids from the amygdaloid complex: in vivo and in vitro studies	1995	65	1240-1249	J Neurochem
Keck, M. E.; Sillaber, I.; Ebner, K.; Welt, T.; Toschi, N.; Kaehler, S. T.; Singewald, N.; Philippu, A.; Elbel, G. K.; Wotjak, C. T.; Holsboer, F.; Landgraf, R.; Engelmann, M.	Acute transcranial magnetic stimulation of frontal brain regions selectively modulates the release of vasopressin, biogenic amines and amino acids in the rat brain	2000	12	3713-3720	Eur J Neurosci
Kekesi, K. A.; Dobolyi, A.; Salfay, O.; Nyitrai, G.; Juhasz, G.	Slow wave sleep is accompanied by release of certain amino acids in the thalamus of cats	1997	8	1183-1186	Neuroreport
Kurtz, P.; Vargas-Lopes, C.; Madeira, C.; Mello, I.; Panizzutti, R.; Azevedo, L. C.; Bozza, F. A.	Pathophysiology of sepsis-associated brain dysfunction: An experimental study of cerebral microdialysis and mitochondrial function	2013	17	S8-S9	Crit Care
Kusaka, T.; Matsuura, S.; Fujikawa, Y.; Okubo, K.; Kawada, K.; Namba, M.; Okada, H.; Imai, T.; Isobe, K.; Itoh, S.	Relationship between cerebral interstitial levels of amino acids and phosphorylation potential during secondary energy failure in hypoxic-ischemic newborn piglets	2004	55	273-279	Pediatr Res
Lallement, G.; Carpentier, P.; Collet, A.; Pernot-Marino, I.;	Effects of soman-induced seizures on different extracellular amino acid levels and on glutamate uptake in	1991	563	234-240	Brain Res

Glutamine					
**Authors**	**Title**	**Year**	**Vol.**	**Pages**	**Journal**
Baubichon, D.; Blanchet, G.	rat hippocampus				
Langer, T. M.; Neumueller, S.; Burgraff, N.; Crumley, E.; Pan, L.; Hodges, M. R.; Forster, H. V.	Ventilatory and neurochemical effects of microdialysis of the ionotropic GABA A receptor agonist muscimol into the ventral respiratory column of awake goats	2016	30	/	FASEB J. Conference: Experiimental Biology
Langlais, P. J.; Shu Xing, Zhang	Extracellular glutamate is increased in thalamus during thiamine deficiency-induced lesions and is blocked by MK-801	1993	61	2175-2182	J Neurochem
Largo, C.; Cuevas, P.; Somjen, G. G.; Martin del Rio, R.; Herreras, O.	The effect of depressing glial function in rat brain in situ on ion homeostasis, synaptic transmission, and neuron survival	1996	16	1219-1229	J Neurosci
Lazarewicz, J. W.; Salinska, E.; Puka, M.	Glycine enhances extracellular 45Ca2+ response to NMDA application investigated with microdialysis of rabbit hippocampus in vivo	1992	52	83-91	Acta Neurobiol Exp (Wars)
Lehmann, A.	Alterations in hippocampal extracellular amino acids and purine catabolites during limbic seizures induced by folate injections into the rabbit amygdala	1987	22	573-578	Neuroscience
Lehmann, A.	Evidence for a direct action of N-methylaspartate on non-neuronal cells	1987	411	95-101	Brain Res
Lehmann, A.	Effects of microdialysis-perfusion with anisoosmotic media on extracellular amino acids in the rat hippocampus and skeletal muscle	1989	53	525-535	J Neurochem
Lehmann, A.; Hagberg, H.; Huxtable, R. J.; Sandberg, M.	Reduction of brain taurine: Effects on neurotoxic and metabolic actions of kainate	1987	10	265-274	Neurochem Int
Lekieffre, D.; Callebert, J.; Plotkine, M.; Boulu, R. G.	Concomitant increases in the extracellular concentrations of excitatory and inhibitory amino acids in the rat hippocampus during forebrain ischemia	1992	137	78-82	Neurosci Lett
Lerma, J.; Herranz, A. S.; Herreras, O.	In vivo determination of extracellular concentration of amino acids in the rat hippocampus. A method based on brain dialysis and computerized analysis	1986	384	145-155	Brain Res
Lerma, J.; Herranz, A. S.; Herreras, O.; Munoz, D.; Solis, J. M.; del Rio, R. M.; Delgado, J. M.	gamma-Aminobutyric acid greatly increases the in vivo extracellular taurine in the rat hippocampus	1985	44	983-986	J Neurochem
Li, H.; Yan, Z. Y.	Analysis of amino acid neurotransmitters in hypothalamus	2010	24	1185-	Biomed Chromatogr

Glutamine					
Authors	Title	Year	Vol.	Pages	Journal
	of rats during cerebral ischemia-reperfusion by microdialysis and capillary electrophoresis			1192	
Li, P. A.; He, Q. P.; Miyashita, H.; Howllet, W.; Siesjo, B. K.; Shuaib, A.	Hypothermia ameliorates ischemic brain damage and suppresses the release of extracellular amino acids in both normo- and hyperglycemic subjects	1999	158	242-253	Exp Neurol
Li, T. F.; Xue, W.; Li, M.; Shi, A. X.; Li, K. X.	Establishment of an LC-MS/MS method to determine content of six amino acids in rat brain dialysate. [Chinese]	2015	24	1659-1664	Zhongguo xin yao za zhi
Li, Z. P.; Zhang, X. Y.; Lu, X.; Zhong, M. K.; Ji, Y. H.	Dynamic release of amino acid transmitters induced by valproate in PTZ-kindled epileptic rat hippocampus	2004	44	263-270	Neurochem Int
Lo, E. H.; Steinberg, G. K.; Panahian, N.; Maidment, N. T.; Newcomb, R.	Profiles of extracellular amino acid changes in focal cerebral ischaemia: effects of mild hypothermia	1993	15	281-287	Neurol Res
Luna-Munguia, H.; Meneses, A.; Pena-Ortega, F.; Gaona, A.; Rocha, L.	Effects of hippocampal high-frequency electrical stimulation in memory formation and their association with amino acid tissue content and release in normal rats	2012	22	98-105	Hippocampus
Maione, S.; Marabese, I.; Rossi, F.; Berrino, L.; Palazzo, E.; Trabace, L.	Effects of persistent nociception on periaqueductal gray glycine release	2000	97	311-316	Neuroscience
Maki, O.; Ryuji, N.; Sotaro, M.; Kazuhiko, Y.; Ming-Rong, Z.; Toshimitsu, F.; Osamu, I.	In vivo measurement of glial metabolism in the rat brain with [carbonyl-11]benzyl acetate by PET and microdialysis	2011	54	S300	J Labelled Compd Radiopharm
Malmberg, A. B.; Hamberger, A.; Hedner, T.	Effects of prostaglandin E2 and capsaicin on behavior and cerebrospinal fluid amino acid concentrations of unanesthetized rats: a microdialysis study	1995	65	2185-2193	J Neurochem
Marabese, I.; de Novellis, V.; Palazzo, E.; Mariani, L.; Siniscalco, D.; Rodella, L.; Rossi, F.; Maione, S.	Differential roles of mGlu8 receptors in the regulation of glutamate and gamma-aminobutyric acid release at periaqueductal grey level	2005	49 Suppl1	157-166	Neuropharmacology
Marquez de Prado, B.; Castaneda, T. R.; Galindo, A.; del Arco, A.; Segovia, G.; Reiter, R. J.; Mora, F.	Melatonin disrupts circadian rhythms of glutamate and GABA in the neostriatum of the aware rat: a microdialysis study	2000	29	209-216	J Pineal Res
Mason, P. A.; Escarciga, R.; Doyle, J. M.; Romano, W. F.; Berger, R.	Amino acid concentrations in hypothalamic and caudate nuclei during microwave-induced thermal stress: analysis	1997	18	277-83	Bioelectromagnetics

Glutamine					
**Authors**	**Title**	**Year**	**Vol.**	**Pages**	**Journal**
E.; Donnellan, J. P.	by microdialysis				
Massieu, L.; Gomez-Roman, N.; Montiel, T.	In vivo potentiation of glutamate-mediated neuronal damage after chronic administration of the glycolysis inhibitor iodoacetate	2000	165	257-267	Exp Neurol
Massieu, L.; Morales-Villagran, A.; Tapia, R.	Accumulation of extracellular glutamate by inhibition of its uptake is not sufficient for inducing neuronal damage: an in vivo microdialysis study	1995	64	2262-2272	J Neurochem
Medina-Ceja, L.; Morales-Villagran, A.; Tapia, R.	Action of 4-aminopyridine on extracellular amino acids in hippocampus and entorhinal cortex: a dual microdialysis and electroencehalographic study in awake rats	2000	53	255-262	Brain Res Bull
Melon, C.; Chassain, C.; Bielicki, G.; Renou, J. P.; Kerkerian-Le Goff, L.; Salin, P.; Durif, F.	Progressive brain metabolic changes under deep brain stimulation of subthalamic nucleus in parkinsonian rats	2015	132	703-712	J Neurochem
Mena, F. V.; Baab, P. J.; Zielke, C. L.; Huang, Y.; Zielke, H. R.	Formation of extracellular glutamate from glutamine: exclusion of pyroglutamate as an intermediate	2005	1052	88-96	Brain Res
Mena, F. V.; Baab, P. J.; Zielke, C. L.; Zielke, H. R.	In vivo glutamine hydrolysis in the formation of extracellular glutamate in the injured rat brain	2000	60	632-641	J Neurosci Res
Michalak, A.; Rose, C.; Butterworth, J.; Butterworth, R. F.	Neuroactive amino acids and glutamate (NMDA) receptors in frontal cortex of rats with experimental acute liver failure	1996	24	908-913	Hepatology
Millan, M. H.; Chapman, A. G.; Meldrum, B. S.	Dual inhibitory action of enadoline (CI977) on release of amino acids in the rat hippocampus	1995	279	75-81	Eur J Pharmacol
Millan, M. H.; Chapman, A. G.; Meldrum, B. S.	Contrasting effects of D- and L-(E)-4-(3-phosphono-2-propenyl)piperazine-2-carboxylic acid as anticonvulsants and as inhibitors of potassium-evoked increases in hippocampal extracellular glutamate and aspartate levels in freely moving rats	1994	62	217-222	J Neurochem
Millan, M. H.; Chapman, A. G.; Meldrum, B. S.	Extracellular amino acid levels in hippocampus during pilocarpine-induced seizures	1993	14	139-148	Epilepsy Res
Miyashita, K.; Abe, H.; Nakajima, T.; Ishikawa, A.; Nishiura-Suzuki, M.; Naritomi, H.; Tanaka, R.; Sawada, T.	Glutamate release in the gerbil hippocampus after middle cerebral artery occlusion	1994	5	945-948	Neuroreport
Miyashita, K.; Nakajima, T.;	An adenosine uptake blocker, propentofylline, reduces	1992	17	147-150	Neurochem Res

Glutamine					
**Authors**	**Title**	**Year**	**Vol.**	**Pages**	**Journal**
Ishikawa, A.; Miyatake, T.	glutamate release in gerbil hippocampus following transient forebrain ischemia				
Molchanova, S.; Kbi, P.; Oja, S. S.; Saransaari, P.	Interstitial concentrations of amino acids in the rat striatum during global forebrain ischemia and potassium-evoked spreading depression	2004	29	1519-1527	Neurochem Res
Montiel, T.; Camacho, A.; Estrada-Sanchez, A. M.; Massieu, L.	Differential effects of the substrate inhibitor l-trans-pyrrolidine-2,4-dicarboxylate (PDC) and the non-substrate inhibitor DL-threo-beta-benzyloxyaspartate (DL-TBOA) of glutamate transporters on neuronal damage and extracellular amino acid levels in rat brain in vivo	2005	133	667-678	Neuroscience
Morales-Villagran, A.; Tapia, R.	Preferential stimulation of glutamate release by 4-aminopyridine in rat striatum in vivo	1996	28	35-40	Neurochem Int
Morita, T.; Takahashi, M.; Takeuchi, T.; Hikasa, Y.; Ikeda, S.; Sawada, M.; Sato, K.; Shibahara, T.; Shimada, A.	Changes in extracellular neurotransmitters in the cerebrum of familial idiopathic epileptic shetland sheepdogs using an intracerebral microdialysis technique and immunohistochemical study for glutamate metabolism	2005	67	1119-1126	J Vet Med Sci
Munoz, M. D.; Herreras, O.; Herranz, A. S.	Effects of dihydrokainic acid of extracellular amino acids and neuronal excitability in the in vivo rat hippocampus	1987	26	1-8	Neuropharmacology
Nakata, N.; Kato, H.; Kogure, K.	Ischemic tolerance and extracellular amino acid concentrations in gerbil hippocampus measured by intracerebral microdialysis	1994	35	247-251	Brain Res Bull
Nakata, N.; Kato, H.; Kogure, K.	Protective effects of serotonin reuptake inhibitors, citalopram and clomipramine, against hippocampal CA1 neuronal damage following transient ischemia in the gerbil	1992	590	48-52	Brain Res
Neumann, I.; Landgraf, R.; Bauce, L.; Pittman, Q. J.	Osmotic responsiveness and cross talk involving oxytocin, but not vasopressin or amino acids, between the supraoptic nuclei in virgin and lactating rats	1995	15	3408-3417	J Neurosci
Newcomb, R.; Palma, A.	Effects of diverse omega-conopeptides on the in vivo release of glutamic and gamma-aminobutyric acids	1994	638	95-102	Brain Res
Newcomb, R.; Pierce, A. R.; Kano, T.; Meng, W.; Bosque-Hamilton, P.; Taylor, L.; Curthoys, N.; Lo, E. H.	Characterization of mitochondrial glutaminase and amino acids at prolonged times after experimental focal cerebral ischemia	1998	813	103-111	Brain Res

Glutamine					
**Authors**	**Title**	**Year**	**Vol.**	**Pages**	**Journal**
Nilsson, P.; Hillered, L.; Ponten, U.; Ungerstedt, U.	Changes in cortical extracellular levels of energy-related metabolites and amino acids following concussive brain injury in rats	1990	10	631-637	J Cereb Blood Flow Metab
Nyitrai, G.; Kekesi, K. A.; Szilagyi, N.; Papp, A.; Juhasz, G.; Kardos, J.	Neurotoxicity of lindane and picrotoxin: neurochemical and electrophysiological correlates in the rat hippocampus in vivo	2002	27	139-145	Neurochem Res
Obrenovitch, T. P.; Urenjak, J.; Richards, D. A.; Ueda, Y.; Curzon, G.; Symon, L.	Extracellular neuroactive amino acids in the rat striatum during ischaemia: comparison between penumbral conditions and ischaemia with sustained anoxic depolarisation	1993	61	178-186	J Neurochem
Ohta, K.; Araki, N.; Shibata, M.; Hamada, J.; Komatsumoto, S.; Shimazu, K.; Fukuuchi, Y.	Correlation of in vivo nitric oxide and cGMP with glutamate/glutamine metabolism in the rat striatum	1996	25	379-384	Neurosci Res
Okada, M.; Nakao, R.; Hosoi, R.; Zhang, M. R.; Fukumura, T.; Suzuki, K.; Inoue, O.	In vivo monitoring of extracellular 13N-glutamine derived from blood-borne 13N-ammonia in rat striatum using microdialysis with radio-LC method	2009	184	37-41	J Neurosci Methods
Oliva, P.; Berrino, L.; de Novellis, V.; Palazzo, E.; Marabese, I.; Siniscalco, D.; Scafuro, M.; Mariani, L.; Rossi, F.; Maione, S.	Role of periaqueductal grey prostaglandin receptors in formalin-induced hyperalgesia	2006	530	40-47	Eur J Pharmacol
Ooboshi, H.; Sadoshima, S.; Yao, H.; Ibayashi, S.; Matsumoto, T.; Uchimura, H.; Fujishima, M.	Ischemia-induced release of amino acids in the hippocampus of aged hypertensive rats	1995	15	227-234	J Cereb Blood Flow Metab
Oria, M.; Chatauret, N.; Chavarria, L.; Romero-Gimenez, J.; Palenzuela, L.; Pardo-Yules, B.; Arranz, J. A.; Bodega, G.; Raguer, N.; Cordoba, J.	Motor-evoked potentials in awake rats are a valid method of assessing hepatic encephalopathy and of studying its pathogenesis	2010	52	2077-2085	Hepatology
Orwar, O.; Li, X.; Andine, P.; Bergstrom, C. M.; Hagberg, H.; Folestad, S.; Sandberg, M.	Increased intra- and extracellular concentrations of gamma-glutamylglutamate and related dipeptides in the ischemic rat striatum: involvement of glutamyl transpeptidase	1994	63	1371-1376	J Neurochem
Osborne, P. G.; Hashimoto, M.	Mammalian cerebral metabolism and amino acid	2008	106	1888-	J Neurochem

Glutamine					
**Authors**	**Title**	**Year**	**Vol.**	**Pages**	**Journal**
	neurotransmission during hibernation			1899	
Oshinsky, M. L.; Luo, J.	Neurochemistry of trigeminal activation in an animal model of migraine	2006	46 Suppl 1	S39-S44	Headache
Ovanesov, M. V.; Vogel, M. W.; Moran, T. H.; Pletnikov, M. V.	Neonatal Borna disease virus infection in rats is associated with increased extracellular levels of glutamate and neurodegeneration in the striatum	2007	13	185-194	J Neurovirol
Palmer, A. M.; Marion, D. W.; Botscheller, M. L.; Swedlow, P. E.; Styren, S. D.; DeKosky, S. T.	Traumatic brain injury-induced excitotoxicity assessed in a controlled cortical impact model	1993	61	2015-2024	J Neurochem
Paulsen, R. E.; Fonnum, F.	Role of glial cells for the basal and Ca2+-dependent K+-evoked release of transmitter amino acids investigated by microdialysis	1989	52	1823-1829	J Neurochem
Pena, F.; Tapia, R.	Relationships among seizures, extracellular amino acid changes, and neurodegeneration induced by 4-aminopyridine in rat hippocampus: a microdialysis and electroencephalographic study	1999	72	2006-2014	J Neurochem
Pierard, C.; Peres, M.; Satabin, P.; Guezennec, C. Y.; Lagarde, D.	Effects of GABA-transaminase inhibition on brain metabolism and amino-acid compartmentation: an in vivo study by 2D 1H-NMR spectroscopy coupled with microdialysis	1999	127	321-327	Exp Brain Res
Prazak, J.; Laszikova, E.; Pantoflicek, T.; Ryska, O.; Koblihova, E.; Ryska, M.	Cerebral microdialysis reflects the neuroprotective effect of fractionated plasma separation and adsorption in acute liver failure better and earlier than intracranial pressure: a controlled study in pigs	2013	13	98	BMC Gastroenterol
Puka, M.; Lehmann, A.	In vivo acidosis reduces extracellular concentrations of taurine and glutamate in the rat hippocampus	1994	37	641-646	J Neurosci Res
Puka-Sundvall, M.; Eriksson, P.; Nilsson, M.; Sandberg, M.; Lehmann, A.	Neurotoxicity of cysteine: interaction with glutamate	1995	705	65-70	Brain Res
Qu, Y.; Li, Y.; Vandenbussche, E.; Vandesande, F.; Arckens, L.	In vivo microdialysis in the visual cortex of awake cat. II: sample analysis by microbore HPLC-electrochemical detection and capillary electrophoresis-laser-induced	2001	7	45-51	Brain Res Brain Res Protoc

Glutamine					
**Authors**	**Title**	**Year**	**Vol.**	**Pages**	**Journal**
	fluorescence detection				
Radhakrishnan, R.; Sluka, K. A.	Increased glutamate and decreased glycine release in the rostral ventromedial medulla during induction of a pre-clinical model of chronic widespread muscle pain	2009	457	141-145	Neurosci Lett
Rao, V. L.; Audet, R. M.; Butterworth, R. F.	Selective alterations of extracellular brain amino acids in relation to function in experimental portal-systemic encephalopathy: results of an in vivo microdialysis study	1995	65	1221-1228	J Neurochem
Rappeneau, V.; Blaker, A.; Petro, J. R.; Yamamoto, B. K.; Shimamoto, A.	Disruption of the Glutamate-Glutamine Cycle Involving Astrocytes in an Animal Model of Depression for Males and Females	2016	10	231	Front Behav Neurosci
Renno, W. M.; Mullet, M. A.; Williams, F. G.; Beitz, A. J.	Construction of 1 mm microdialysis probe for amino acids dialysis in rats	1998	79	217-228	J Neurosci Methods
Richards, D. A.; Lemos, T.; Whitton, P. S.; Bowery, N. G.	Extracellular GABA in the ventrolateral thalamus of rats exhibiting spontaneous absence epilepsy: a microdialysis study	1995	35	1674-1680	J Neurochem
Rodriguez Diaz, M.; Alonso, T. J.; Perdomo Diaz, J.; Gonzalez Hernandez, T.; Castro Fuentes, R.; Sabate, M.; Garcia Dopico, J.	Glial regulation of nonsynaptic extracellular glutamate in the substantia nigra	2005	49	134-142	Glia
Rodriguez, M. J.; Robledo, P.; Andrade, C.; Mahy, N.	In vivo co-ordinated interactions between inhibitory systems to control glutamate-mediated hippocampal excitability	2005	95	651-661	J Neurochem
Rodriguez-Navarro, J. A.; Gonzalo-Gobernado, R.; Herranz, A. S.; Gonzlez-Vigueras, J. M.; Solis, J. M.	High potassium induces taurine release by osmosensitive and osmoresistant mechanisms in the rat hippocampus in vivo	2009	87	208-217	J Neurosci Res
Rosdahl, H.; Samuelsson, A. C.; Ungerstedt, U.; Henriksson, J.	Influence of adrenergic agonists on the release of amino acids from rat skeletal muscle studied by microdialysis	1998	163	349-360	Acta Physiol Scand
Rose, C.; Ytrebo, L. M.; Davies, N. A.; Sen, S.; Nedredal, G. I.; Belanger, M.; Revhaug, A.; Jalan, R.	Association of reduced extracellular brain ammonia, lactate, and intracranial pressure in pigs with acute liver failure	2007	46	1883-1892	Hepatology
Runnerstam, M.; Bao, F.; Huang,	A new model for diffuse brain injury by rotational	2001	18	259-273	J Neurotrauma

Glutamine					
**Authors**	**Title**	**Year**	**Vol.**	**Pages**	**Journal**
Y.; Shi, J.; Gutierrez, E.; Hamberger, A.; Hansson, H. A.; Viano, D.; Haglid, K.	acceleration: II. Effects on extracellular glutamate, intracranial pressure, and neuronal apoptosis				
Runnerstam, M.; Stephensen, H.; von Essen, C.; Nystrom, B.; Hamberger, A.	Subarachnoid blood infusion versus raised intracranial pressure: effects on the amino acid pattern in the extracellular fluid of the rabbit hippocampus	1999	21	404-408	Neurol Res
Samuelsson, C.; Kumlien, E.; Elfving, A.; Lindholm, D.; Ronne-Engstrom, E.	The effects of PBN (phenyl-butyl-nitrone) on GLT-1 levels and on the extracellular levels of amino acids and energy metabolites in a model of iron-induced posttraumatic epilepsy	2003	56	165-173	Epilepsy Res
Sandberg, M.; Butcher, S. P.; Hagberg, H.	Extracellular overflow of neuroactive amino acids during severe insulin-induced hypoglycemia: in vivo dialysis of the rat hippocampus	1986	47	178-184	J Neurochem
Santos-Fandila, A.; Zafra-Gomez, A.; Barranco, A.; Navalon, A.; Rueda, R.; Ramirez, M.	Quantitative determination of neurotransmitters, metabolites and derivates in microdialysates by UHPLC-tandem mass spectrometry	2013	114	79-89	Talanta
Scheller, D. K.; De Ryck, M.; Kolb, J.; Szathmary, S.; van Reempts, J.; Clincke, G.; Tegtmeier, F.	Lubeluzole blocks increases in extracellular glutamate and taurine in the peri-infarct zone in rats	1997	338	243-251	Eur J Pharmacol
Schubert, G. A.; Poli, S.; Mendelowitsch, A.; Schilling, L.; Thome, C.	Hypothermia reduces early hypoperfusion and metabolic alterations during the acute phase of massive subarachnoid hemorrhage: a laser-Doppler-flowmetry and microdialysis study in rats	2008	25	539-548	J Neurotrauma
Segovia, G.; Del Arco, A.; Mora, F.	Effects of aging on the interaction between glutamate, dopamine, and GABA in striatum and nucleus accumbens of the awake rat	1999	73	2063-2072	J Neurochem
Segovia, G.; Del Arco, A.; Mora, F.	Endogenous glutamate increases extracellular concentrations of dopamine, GABA, and taurine through NMDA and AMPA/kainate receptors in striatum of the freely moving rat: a microdialysis study	1997	69	1476-1483	J Neurochem
Segovia, G.; Del Arco, A.; Prieto, L.; Mora, F.	Glutamate-glutamine cycle and aging in striatum of the awake rat: effects of a glutamate transporter blocker	2001	26	37-41	Neurochem Res
Segovia, G.; Mora, F.	Role of nitric oxide in modulating the release of dopamine,	1998	45	275-279	Brain Res Bull

Glutamine					
**Authors**	**Title**	**Year**	**Vol.**	**Pages**	**Journal**
	glutamate, and GABA in striatum of the freely moving rat				
Segovia, G.; Porras, A.; Mora, F.	Effects of 4-aminopyridine on extracellular concentrations of glutamate in striatum of the freely moving rat	1997	22	1491-1497	Neurochem Res
Shah, A. J.; de Biasi, V.; Taylor, S. G.; Roberts, C.; Hemmati, P.; Munton, R.; West, A.; Routledge, C.; Camilleri, P.	Development of a protocol for the automated analysis of amino acids in brain tissue samples and microdialysates	1999	735	133-140	J Chromatogr B Biomed Sci Appl
Sheng, W.; Hang, H. W.; Ruan, D. Y.	In vivo microdialysis study of the relationship between lead-induced impairment of learning and neurotransmitter changes in the hippocampus	2005	20	233-240	Environ Toxicol Pharmacol
Shimizu, H.; Graham, S. H.; Chang, L. H.; Mintorovitch, J.; James, T. L.; Faden, A. I.; Weinstein, P. R.	Relationship between extracellular neurotransmitter amino acids and energy metabolism during cerebral ischemia in rats monitored by microdialysis and in vivo magnetic resonance spectroscopy	1993	605	33-42	Brain Res
Shirotani, T.; Shima, K.; Chigasaki, H.	In vivo studies of extracellular metabolites in the striatum after distal middle cerebral artery occlusion in stroke-prone spontaneously hypertensive rats	1995	26	878-884	Stroke
Silverstein, F. S.; Naik, B.; Simpson, J.	Hypoxia-ischemia stimulates hippocampal glutamate efflux in perinatal rat brain: an in vivo microdialysis study	1991	30	587-590	Pediatr Res
Silverstein, F. S.; Simpson, J.; Gordon, K. E.	Hypoglycemia alters striatal amino acid efflux in perinatal rats: an in vivo microdialysis study	1990	28	516-521	Ann Neurol
Slezia, A.; Kekesi, A. K.; Szikra, T.; Papp, A. M.; Nagy, K.; Szente, M.; Magloczky, Z.; Freund, T. F.; Juhasz, G.	Uridine release during aminopyridine-induced epilepsy	2004	16	490-499	Neurobiol Dis
Solis, J. M.; Herranz, A. S.; Herreras, O.; Lerma, J.; Martin Del Rio, R.	Low chloride-dependent release of taurine by a furosemide-sensitive process in the in vivo rat hippocampus	1988	24	885-891	Neuroscience
Solis, J. M.; Herranz, A. S.; Herreras, O.; Munoz, M. D.; Martin del Rio, R.; Lerma, J.	Variation of potassium ion concentrations in the rat hippocampus specifically affects extracellular taurine levels	1986	66	263-268	Neurosci Lett
Stein-Behrens, B. A.; Elliott, E. M.; Miller, C. A.; Schilling, J. W.;	Glucocorticoids exacerbate kainic acid-induced extracellular accumulation of excitatory amino acids in the	1992	58	1730-1735	J Neurochem

Glutamine					
**Authors**	**Title**	**Year**	**Vol.**	**Pages**	**Journal**
Newcombe, R.; Sapolsky, R. M.	rat hippocampus				
Suarez, L. M.; Munoz, M. D.; Gonzalez, J. C.; Bustamante, J.; Del Rio, R. M.; Solis, J. M.	The taurine transporter substrate guanidinoethyl sulfonate mimics the action of taurine on long-term synaptic potentiation	2016	48	2647-2656	Amino Acids
Suzuki, K.; Matsuo, N.; Moriguchi, T.; Takeyama, N.; Kitazawa, Y.; Tanaka, T.	Changes in brain ECF amino acids in rats with experimentally induced hyperammonemia	1992	7	63-75	Metab Brain Dis
Sved, A. F.; Curtis, J. T.	Amino acid neurotransmitters in nucleus tractus solitarius: an in vivo microdialysis study	1993	61	2089-2098	J Neurochem
Swanson, R. A.; Chen, J.; Graham, S. H.	Glucose can fuel glutamate uptake in ischemic brain	1994	14	1-6	J Cereb Blood Flow Metab
Szeg, E. M.; Kekesi, K. A.; Szabo, Z.; Janaky, T.; Juhasz, G. D.	Estrogen regulates cytoskeletal flexibility, cellular metabolism and synaptic proteins: A proteomic study	2010	35	807-819	Psychoneuroendocrinology
Szerb, J. C.	Glutamate release and spreading depression in the fascia dentata in response to microdialysis with high K+: role of glia	1991	542	259-265	Brain Res
Szerb, J. C.; Redondo, I. M.	Astrocytes and the entry of circulating ammonia into the brain: effect of fluoroacetate	1993	8	217-234	Metab Brain Dis
Takatsuru, Y.; Eto, K.; Kaneko, R.; Masuda, H.; Shimokawa, N.; Koibuchi, N.; Nabekura, J.	Critical role of the astrocyte for functional remodeling in contralateral hemisphere of somatosensory cortex after stroke	2013	33	4683-4692	J Neurosci
Togashi, H.; Nakamura, K.; Matsumoto, M.; Ueno, K.; Ohashi, S.; Saito, H.; Yoshioka, M.	Aniracetam enhances glutamatergic transmission in the prefrontal cortex of stroke-prone spontaneously hypertensive rats	2002	320	109-112	Neurosci Lett
Tossman, U.; Delin, A.; Eriksson, L. S.; Ungerstedt, U.	Brain cortical amino acids measured by intracerebral dialysis in portacaval shunted rats	1987	12	265-269	Neurochem Res
Tossman, U.; Eriksson, S.; Delin, A.; Hagenfeldt, L.; Law, D.; Ungerstedt, U.	Brain amino acids measured by intracerebral dialysis in portacaval shunted rats	1983	41	1046-1051	J Neurochem
Tossman, U.; Jonsson, G.; Ungerstedt, U.	Regional distribution and extracellular levels of amino acids in rat central nervous system	1986	127	533-545	Acta Physiol Scand
Tossman, U.; Segovia, J.; Ungerstedt, U.	Extracellular levels of amino acids in striatum and globus pallidus of 6-hydroxydopamine-lesioned rats measured	1986	127	547-551	Acta Physiol Scand

Glutamine					
**Authors**	**Title**	**Year**	**Vol.**	**Pages**	**Journal**
	with microdialysis				
Toth, E.; Harsing Jr, L. G.; Sershen, H.; Ramacci, M. T.; Lajtha, A.	Effect of acetyl-L-carnitine on extracellular amino acid levels in vivo in rat brain regions	1993	18	573-578	Neurochem Res
Toya, S.; Takatsuru, Y.; Kokubo, M.; Amano, I.; Shimokawa, N.; Koibuchi, N.	Early-life-stress affects the homeostasis of glutamatergic synapses	2014	40	3627-3634	Eur J Neurosci
Tseng, E. E.; Brock, M. V.; Kwon, C. C.; Annanata, M.; Lange, M. S.; Troncoso, J. C.; Johnston, M. V.; Baumgartner, W. A.	Increased intracerebral excitatory amino acids and nitric oxide after hypothermic circulatory arrest	1999	67	371-376	Ann Thorac Surg
Tseng, E. E.; Brock, M. V.; Lange, M. S.; Troncoso, J. C.; Blue, M. E.; Lowenstein, C. J.; Johnston, M. V.; Baumgartner, W. A.	Monosialoganglioside GM1 inhibits neurotoxicity after hypothermic circulatory arrest	1998	124	298-306	Surgery
Tseng, E. E.; Brock, M. V.; Lange, M. S.; Troncoso, J. C.; Blue, M. E.; Lowenstein, C. J.; Johnston, M. V.; Baumgartner, W. A.	Glutamate excitotoxicity mediates neuronal apoptosis after hypothermic circulatory arrest	2010	89	440-445	Ann Thorac Surg
Tsukada, Y.; Kanamatsu, T.; Takahara, H.	Neurotransmitter release from the medial hyperstriatum ventrale of the chick forebrain accompanying filial imprinting behavior, measured by in vivo microdialysis	1999	24	315-320	Neurochem Res
Ueda, Y.; Mitsuyama, Y.	Nitroprusside inhibits kindling development and potentiates excitatory amino acid exocytosis in the kindling rat	1997	23	49-60	J Brain Sci
Vallee, N.; Rostain, J. C.; Boussuges, A.; Risso, J. J.	Comparison of nitrogen narcosis and helium pressure effects on striatal amino acids: a microdialysis study in rats	2009	34	835-844	Neurochem Res
Vallee, N.; Rostain, J. C.; Risso, J. J.	A pressurized nitrogen counterbalance to cortical glutamatergic pathway stimulation	2010	35	718-726	Neurochem Res
Varga, V.; Kekesi, A.; Juhasz, G.; Kocsis, B.	Reduction of the extracellular level of glutamate in the median raphe nucleus associated with hippocampal theta activity in the anaesthetized rat	1998	84	49-57	Neuroscience
Vasylieva, N.; Maucler, C.; Meiller, A.; Viscogliosi, H.; Lieutaud, T.;	Immobilization method to preserve enzyme specificity in biosensors: consequences for brain glutamate detection	2013	85	2507-2515	Anal Chem

Glutamine					
**Authors**	**Title**	**Year**	**Vol.**	**Pages**	**Journal**
Barbier, D.; Marinesco, S.					
Vogels, B. A.; Karlsen, O. T.; Mass, M. A.; Bovee, W. M.; Chamuleau, R. A.	L-ornithine vs. L-ornithine-L-aspartate as a treatment for hyperammonemia-induced encephalopathy in rats	1997	26	174-182	J Hepatol
Wade, J. V.; Samson, F. E.; Nelson, S. R.; Pazdernik, T. L.	Changes in extracellular amino acids during soman- and kainic acid-induced seizures	1987	49	645-650	J Neurochem
Wang, L.; Maher, T. J.; Wurtman, R. J.	Oral L-glutamine increases GABA levels in striatal tissue and extracellular fluid	2007	21	1227-1232	FASEB J.
Watts, J.; Fowler, L.; Whitton, P. S.; Pearce, B.	Release of arginine, glutamate and glutamine in the hippocampus of freely moving rats: Involvement of nitric oxide	2005	65	521-528	Brain Res Bull
Westergren, I.; Nystrom, B.; Hamberger, A.; Johansson, B. B.	Intracerebral dialysis and the blood-brain barrier	1995	64	229-234	J Neurochem
Westerink, B. H.; Damsma, G.; de Vries, J. B.	Effect of ouabain applied by intrastriatal microdialysis on the in vivo release of dopamine, acetylcholine, and amino acids in the brain of conscious rats	1989	52	705-712	J Neurochem
Westerink, B. H.; Hofsteede, H. M.; Damsma, G.; de Vries, J. B.	The significance of extracellular calcium for the release of dopamine, acetylcholine and amino acids in conscious rats, evaluated by brain microdialysis	1988	337	373-378	Naunyn Schmiedebergs Arch Pharmacol
Wong, J. M.; Malec, P. A.; Mabrouk, O. S.; Ro, J.; Dus, M.; Kennedy, R. T.	Benzoyl chloride derivatization with liquid chromatography-mass spectrometry for targeted metabolomics of neurochemicals in biological samples	2016	1446	78-90	J Chromatogr A
Xu, G. Y.; McAdoo, D. J.; Hughes, M. G.; Robak, G.; De Castro Jr, R.	Considerations in the determination by microdialysis of resting extracellular amino acid concentrations and release upon spinal cord injury	1998	86	1011-1021	Neuroscience
Yager, J. Y.; Armstrong, E. A.; Miyashita, H.; Wirrell, E. C.	Prolonged neonatal seizures exacerbate hypoxic-ischemic brain damage: correlation with cerebral energy metabolism and excitatory amino acid release	2002	24	367-381	Dev Neurosci
Yamada, K.; Moriguchi, A.; Mikami, H.; Okuda, N.; Higaki, J.; Ogihara, T.	The effect of central amino acid neurotransmitters on the antihypertensive response to angiotensin blockade in spontaneous hypertension	1995	13	1624-1630	J Hypertens
Yang, C. S.; Tsai, P. J.; Chen, W. Y.; Tsai, W. J.; Kuo, J. S.	On-line derivatization for continuous and automatic monitoring of brain extracellular glutamate levels in	1999	734	1-6	J Chromatogr B Biomed Sci Appl

Glutamine					
**Authors**	**Title**	**Year**	**Vol.**	**Pages**	**Journal**
	anesthetized rats: a microdialysis study				
Yin, P. B.; Li, B. M.; Ye, W. L.; Mei, Z. T.	[A microdialysis study of excitatory amino acid levels of the monkey's caudate nucleus during the delayed go/no-go task]	1997	49	128-134	Sheng Li Xue Bao
Young, A. M.; Bradford, H. F.	N-methyl-D-aspartate releases excitatory amino acids in rat corpus striatum in vivo	1991	56	1677-1683	J Neurochem
Young, A. M.; Bradford, H. F.	Excitatory amino acid neurotransmitters in the corticostriate pathway: studies using intracerebral microdialysis in vivo	1986	47	1399-1404	J Neurochem
Young, A. M.; Foley, P. M.; Bradford, H. F.	Preloading in vivo: a rapid and reliable method for measuring gamma-aminobutyric acid and glutamate fluxes by microdialysis	1990	55	1060-1063	J Neurochem
Yu, H. L.; An, Y.; Jiang, H. Y.; Jin, Q. H.; Jin, Y. Z.	Changes of amino acid concentrations in the rat medial vestibular nucleus following unilateral labyrinthectomy	2007	59	71-78	Sheng Li Xue Bao
Zhang, D.; Zhang, J.; Ma, W.; Chen, D.; Han, H.; Shu, H.; Liu, G.	Analysis of trace amino acid neurotransmitters in hypothalamus of rats after exhausting exercise using microdialysis	2001	758	277-282	J Chromatogr B Biomed Sci Appl
Zhang, W. Q.; Rogers, B. C.; Tandon, P.; Hudson, P. M.; Sobotka, T. J.; Hong, J. S.; Tilson, H. A.	Systemic administration of kainic acid increases GABA levels in perfusate from the hippocampus of rats in vivo	1990	11	593-600	Neurotox
Zhu, D. N.; Moriguchi, A.; Mikami, H.; Higaki, J.; Ogihara, T.	Central amino acids mediate cardiovascular response to angiotensin II in the rat	1998	45	189-197	Brain Res Bull

Appendix 5: Studies describing baseline dialysate concentrations for glutamate

Glutamate					
**Authors**	**Title**	**Year**	**Vol.**	**Pages**	**Journal**
Abarca, C.; Silva, E.; Sepulveda, M. J.; Oliva, P.; Contreras, E.	Neurochemical changes after morphine, dizocilpine or riluzole in the ventral posterolateral thalamic nuclei of rats with hyperalgesia	2000	403	67-74	Eur J Pharmacol
Abarca, J.; Bustos, G.	Differential regulation of glutamate, aspartate and gamma-amino-butyrate release by N-methyl-D-aspartate receptors in rat striatum after partial and extensive lesions to the nigro-striatal dopamine pathway	1999	35	19-33	Neurochem Int
Abarca, J.; Gysling, K.; Roth, R. H.; Bustos, G.	Changes in extracellular levels of glutamate and aspartate in rat substantia nigra induced by dopamine receptor ligands: in vivo microdialysis studies	1995	20	159-169	Neurochem Res
Abekawa, T.	[Experimental study of methamphetamine psychosis--role of glutamate and nitric oxide in methamphetamine-induced dopaminergic and serotonergic neurotoxicity in the rat brain]	1997	72	113-126	Hokkaido Igaku Zasshi
Abekawa, T.; Honda, M.; Ito, K.; Koyama, T.	Effects of NRA0045, a novel potent antagonist at dopamine D4, 5-HT2A, and alpha1 adrenaline receptors, and NRA0160, a selective D4 receptor antagonist, on phencyclidine-induced behavior and glutamate release in rats	2003	169	247-256	Psychopharmacology (Berl)
Abekawa, T.; Ito, K.; Koyama, T.	Different effects of a single and repeated administration of clozapine on phencyclidine-induced hyperlocomotion and glutamate releases in the rat medial prefrontal cortex at short- and long-term withdrawal from this antipsychotic	2007	375	261-271	Naunyn Schmiedebergs Arch Pharmacol
Abekawa, T.; Ito, K.; Koyama, T.	Role of the simultaneous enhancement of NMDA and dopamine D1 receptor-mediated neurotransmission in the effects of clozapine on phencyclidine-induced acute increases in glutamate levels in the rat medial prefrontal cortex	2006	374	177-193	Naunyn Schmiedebergs Arch Pharmacol
Abekawa, T.; Ito, K.; Nakagawa, S.; Nakato, Y.; Koyama, T.	Effects of aripiprazole and haloperidol on progression to schizophrenia-like behavioural abnormalities and apoptosis in rodents	2011	125	77-87	Schizophr Res
Abekawa, T.; Ohmori, T.; Ito, K.; Koyama, T.	D1 dopamine receptor activation reduces extracellular glutamate and GABA concentrations in the medial prefrontal cortex	2000	867	250-254	Brain Res
Abekawa, T.; Ohmori, T.; Koyama, T.	Effects of repeated administration of a high dose of methamphetamine on dopamine and glutamate release in rat	1994	643	276-281	Brain Res

Glutamate					
**Authors**	**Title**	**Year**	**Vol.**	**Pages**	**Journal**
	striatum and nucleus accumbens				
Abraham, I.; Juhasz, G.; Kekesi, K. A.; Kovacs, K. J.	Effect of intrahippocampal dexamethasone on the levels of amino acid transmitters and neuronal excitability	1996	733	56-63	Brain Res
Abraham, I.; Juhasz, G.; Kekesi, K. A.; Kovacs, K. J.	Corticosterone peak is responsible for stress-induced elevation of glutamate in the hippocampus	1998	2	171-181	Stress
Acworth, I. N.; Yu, J.; Ryan, E.; Gariepy, K. C.; Gamache, P.; Hull, K.; Maher, T.	Simultaneous measurement of monoamine, amino acid, and drug levels, using high performance liquid chromatography and coulometric array technology: Application to in vivo microdialysis perfusate analysis	1994	17	685-705	J Liq Chromatogr
Adachi, H.; Fujisawa, H.; Maekawa, T.; Yamashita, T.; Ito, H.	Changes in the extracellular glutamate concentrations in the rat cortex following localized by hyperthermia	1995	11	587-599	Int J Hyperthermia
Adachi, N.; Chen, J.; Liu, K.; Nagaro, T.; Arai, T.	Metyrapone alleviates ischemic neuronal damage in the gerbil hippocampus	1999	373	147-152	Eur J Pharmacol
Adachi, N.; Chen, J.; Nakanishi, K.; Arai, T.	Pre-ischaemic administration of procaine suppresses ischaemic glutamate release and reduces neuronal damage in the gerbil hippocampus	1999	83	472-474	Br J Anaesth
Adachi, N.; Seyfried, F. J.; Arai, T.	Blockade of central histaminergic H2 receptors aggravates ischemic neuronal damage in gerbil hippocampus	2001	29	1189-1194	Crit Care Med
Adams, B. W.; Moghaddam, B.	Effect of clozapine, haloperidol, or M100907 on phencyclidine-activated glutamate efflux in the prefrontal cortex	2001	50	750-757	Biol Psychiatry
Adams, B.; Moghaddam, B.	Corticolimbic dopamine neurotransmission is temporally dissociated from the cognitive and locomotor effects of phencyclidine	1998	18	5545-5554	J Neurosci
Agarwal, S.; Wang, M.; Joshi, S.	Comparison of the effect of propofol-Induced EEG silence with hypothermia and normothermic controls with an active EEG on early ischemic markers during transient global cerebral hypoperfusion: A microdialysis study in the rabbit brain	2011	23(4)	393	J Neurosurg Anesthesiol
Agarwal, S.; Wang, M.; Joshi, S.	Comparison of the effect of propofol induced eeg silence with hypothermia on early ischemic markers during transient global cerebral hypoperfusion: A microdialysis study in the rabbit brain	2011	1	S40	Neurocrit Care
Agarwal, S.; Wang, M.; Joshi, S.	Characterization of ischemia thresholds as evidenced by optically measured NADH concentrations and microdialysis study during transient cerebral hypoperfusion in anesthetized rabbits	2011	23(4)	428	J Neurosurg Anesthesiol

Glutamate					
**Authors**	**Title**	**Year**	**Vol.**	**Pages**	**Journal**
Aghajanian, G. K.; Kogan, J. H.; Moghaddam, B.	Opiate withdrawal increases glutamate and aspartate efflux in the locus coeruleus: an in vivo microdialysis study	1994	636	126-130	Brain Res
Ago, Y.; Araki, R.; Yano, K.; Hiramatsu, N.; Kawasaki, T.; Chaki, S.; Nakazato, A.; Onoe, H.; Hashimoto, H.; Baba, A.; Takuma, K.; Matsuda, T.	Activation of metabotropic glutamate 2/3 receptors attenuates methamphetamine-induced hyperlocomotion and increase in prefrontal serotonergic neurotransmission	2011	217	443-452	Psychopharmacology (Berl)
Ahmad, S.; Fowler, L. J.; Whitton, P. S.	Lamotrigine, carbamazepine and phenytoin differentially alter extracellular levels of 5-hydroxytryptamine, dopamine and amino acids	2005	63	141-149	Epilepsy Res
Ahmad, S.; Fowler, L. J.; Whitton, P. S.	Effects of combined lamotrigine and valproate on basal and stimulated extracellular amino acids and monoamines in the hippocampus of freely moving rats	2005	371	1-8	Naunyn Schmiedebergs Arch Pharmacol
Ahmad, S.; Fowler, L. J.; Whitton, P. S.	Effects of acute and chronic lamotrigine treatment on basal and stimulated extracellular amino acids in the hippocampus of freely moving rats	2004	1029	41-47	Brain Res
Alabadi, J. A.; Thibault, J. L.; Pinard, E.; Seylaz, J.; Lasbennes, F.	7-Nitroindazole, a selective inhibitor of nNOS, increases hippocampal extracellular glutamate concentration in status epilepticus induced by kainic acid in rats	1999	839	305-312	Brain Res
Albrecht, J.; Hilgier, W.; Zielinska, M.; Januszewski, S.; Hesselink, M.; Quack, G.	Extracellular concentrations of taurine, glutamate, and aspartate in the cerebral cortex of rats at the asymptomatic stage of thioacetamide-induced hepatic failure: modulation by ketamine anesthesia	2000	25	1497-1502	Neurochem Res
Alessandri, B.; Heimann, A.; Filippi, R.; Kopacz, L.; Kempski, O.	Moderate controlled cortical contusion in pigs: effects on multi-parametric neuromonitoring and clinical relevance	2003	20	1293-1305	J Neurotrauma
Alessandri, B.; Schwandt, E.; Kamada, Y.; Nagata, M.; Heimann, A.; Kempski, O.	The neuroprotective effect of lactate is not due to improved glutamate uptake after controlled cortical impact in rats	2012	29	2181-2191	J Neurotrauma
Alexander, G. M.; Deitch, J. S.; Seeburger, J. L.; Del Valle, L.; Heiman-Patterson, T. D.	Elevated cortical extracellular fluid glutamate in transgenic mice expressing human mutant (G93A) Cu/Zn superoxide dismutase	2000	74	1666-1673	J Neurochem
Alexander, G. M.; Grothusen, J. R.; Gordon, S. W.; Schwartzman, R. J.	Intracerebral microdialysis study of glutamate reuptake in awake, behaving rats	1997	776	1-10	Brain Res
Alexander, K. S.; Pocivavsek, A.; Wu, H. Q.; Pershing, M. L.; Schwarcz, R.; Bruno,	Early developmental elevations of brain kynurenic acid impair cognitive flexibility in adults: reversal with galantamine	2013	238	19-28	Neuroscience

Glutamate					
**Authors**	**Title**	**Year**	**Vol.**	**Pages**	**Journal**
J. P.					
Allchin, R. E.; Batten, T. F. C.; McWilliam, P. N.; Vaughan, P. F. T.	Release of glutamate from the dorsal horn of the anaesthetized cat on sciatic nerve stimulation: An in vivo microdialysis study	1993	467	185P	J Physiol
Allchin, R. E.; Batten, T. F.; McWilliam, P. N.; Vaughan, P. F.	Electrical stimulation of the vagus increases extracellular glutamate recovered from the nucleus tractus solitarii of the cat by in vivo microdialysis	1994	79	265-268	Exp Physiol
Allen, J. P.; Hathway, G. J.; Clarke, N. J.; Jowett, M. I.; Topps, S.; Kendrick, K. M.; Humphrey, P. P.; Wilkinson, L. S.; Emson, P. C.	Somatostatin receptor 2 knockout/lacZ knockin mice show impaired motor coordination and reveal sites of somatostatin action within the striatum	2003	17	1881-1895	Eur J Neurosci
Allers, K. A.; Dremencov, E.; Ceci, A.; Flik, G.; Ferger, B.; Cremers, T. I.; Ittrich, C.; Sommer, B.	Acute and repeated flibanserin administration in female rats modulates monoamines differentially across brain areas: a microdialysis study	2010	7	1757-1767	J Sex Med
Ally, A.; Nauli, S. M.; Maher, T. J.	Cardiovascular responses and neurotransmission in the ventrolateral medulla during skeletal muscle contraction following transient middle cerebral artery occlusion and reperfusion	2002	952	176-187	Brain Research
Ally, A.; Phattanarudee, S.; Shafique, E.; Maher, T.	Effects of medullary administration of nitric oxide precursor on cardiovascular responses and neurotransmission during static exercise in stroke rats	2012	26	/	FASEB J Conference: Experimental Biology
Ally, A.; Tedesco, A.; Chaitoff, K. A.	Effects of inducible nitric oxide synthase (iNOS) blockade within the periaqueductal gray on cardiovascular responses during mechanical, heat, and cold nociception	2009	23	/	FASEB J Conference: Experimental Biology
Almaguer-Melian, W.; Cruz-Aguado, R.; Riva Cde, L.; Kendrick, K. M.; Frey, J. U.; Bergado, J.	Effect of LTP-reinforcing paradigms on neurotransmitter release in the dentate gyrus of young and aged rats	2005	327	877-883	Biochem Biophys Res Commun
Alves, O. L.; Bullock, R.; Clausen, T.; Reinert, M.; Reeves, T. M.	Concurrent monitoring of cerebral electrophysiology and metabolism after traumatic brain injury: an experimental and clinical study	2005	22	733-749	J Neurotrauma
Amakawa, K.; Adachi, N.; Liu, K.; Ikemune, K.; Fujitani, T.; Arai, T.	Effects of pre- and postischemic administration of thiopental on transmitter amino acid release and histologic outcome in gerbils	1996	85	1422-1430	Anesthesiology
Amantea, D.; Fratto, V.; Maida, S.; Rotiroti, D.; Ragusa, S.; Nappi, G.; Bagetta, G.; Corasaniti, M. T.	Prevention of Glutamate Accumulation and Upregulation of Phospho-Akt may Account for Neuroprotection Afforded by Bergamot Essential Oil against Brain Injury Induced by Focal	2009	85	389-405	Int Rev Neurobiol

Glutamate					
**Authors**	**Title**	**Year**	**Vol.**	**Pages**	**Journal**
	Cerebral Ischemia in Rat				
Amer-Wahlin, I.; Nord, A.; Bottalico, B.; Hansson, S. R.; Ley, D.; Marsal, K.; Ungerstedt, U.; Nordstrom, C. H.	Fetal cerebral energy metabolism and electrocardiogram during experimental umbilical cord occlusion and resuscitation	2010	23	158-166	J Matern Fetal Neonatal Med
Amitai, N.; Kuczenski, R.; Behrens, M. M.; Markou, A.	Repeated phencyclidine administration alters glutamate release and decreases GABA markers in the prefrontal cortex of rats	2012	62	1422-1431	Neuropharmacology
Ampe, B.; Massie, A.; Haens, J.; Ebinger, G.; Michotte, Y.; Sarre, S.	NMDA-mediated release of glutamate and GABA in the subthalamic nucleus is mediated by dopamine: an in vivo microdialysis study in rats	2007	103	1063-1074	J Neurochem
Anderson, J. J.; DiMicco, J. A.	The use of microdialysis for studying the regional effects of pharmacological manipulation on extracellular levels of amino acids--some methodological aspects	1992	51	623-630	Life Sci
Anderson, J. J.; DiMicco, J. A.	Effect of local inhibition of gamma-aminobutyric acid uptake in the dorsomedial hypothalamus on extracellular levels of gamma-aminobutyric acid and on stress-induced tachycardia: a study using microdialysis	1990	255	1399-1407	J Pharmacol Exp Ther
Anderzhanova, E.; Rayevsky, K. S.; Saransaari, P.; Riitamaa, E.; Oja, S. S.	Effects of acute toxic doses of psychostimulants on extracellular levels of excitatory amino acids and taurine in rats: comparison of d-amphetamine and sydnocarb	2002	965	193-203	Ann N Y Acad Sci
Anderzhanova, E.; Rayevsky, K. S.; Saransaari, P.; Riitamaa, E.; Oja, S. S.	Effects of sydnocarb and D-amphetamine on the extracellular levels of amino acids in the rat caudate-putamen	2001	428	87-95	Eur J Pharmacol
Anderzhanova, E.; Saransaari, P.; Oja, S. S.	Neuroprotective mechanisms of taurine in vivo	2006	583	377-387	Adv Exp Med Biol
Andiarzhanova, E. A.; Saransaari, R.; Riitamaa, E.; Oiia, S. S.; Raevskii, K. S.	[Extracellular level of neuroactive amino acids in the rat neostriatum after treatment with psychostimulants (microdialysis study)]	2002	65	7-13	Eksp Klin Farmakol
Andine, P.; Orwar, O.; Jacobson, I.; Sandberg, M.; Hagberg, H.	Changes in extracellular amino acids and spontaneous neuronal activity during ischemia and extended reflow in the CA1 of the rat hippocampus	1991	57	222-229	J Neurochem
Andine, P.; Rudolphi, K. A.; Fredholm, B. B.; Hagberg, H.	Effect of propentofylline (HWA 285) on extracellular purines and excitatory amino acids in CA1 of rat hippocampus during transient ischaemia	1990	100	814-818	Br J Pharmacol
Andreassen, O. A.; Jenkins, B. G.;	Increases in cortical glutamate concentrations in transgenic	2001	77	383-390	J Neurochem

Glutamate					
Authors	Title	Year	Vol.	Pages	Journal
Dedeoglu, A.; Ferrante, K. L.; Bogdanov, M. B.; Kaddurah-Daouk, R.; Beal, M. F.	amyotrophic lateral sclerosis mice are attenuated by creatine supplementation				
Andresen, J. H.; Saugstad, O. D.	Effects of nicotine infusion on striatal glutamate and cortical non-protein-bound iron in hypoxic newborn piglets	2008	94	284-292	Neonatology
Anneken, J. H.; Cunningham, J. I.; Collins, S. A.; Yamamoto, B. K.; Gudelsky, G. A.	MDMA increases glutamate release and reduces parvalbumin-positive GABAergic cells in the dorsal hippocampus of the rat: role of cyclooxygenase	2013	8	58-65	J Neuroimmune Pharmacol
Anneken, J. H.; Gudelsky, G. A.	MDMA produces a delayed and sustained increase in the extracellular concentration of glutamate in the rat hippocampus	2012	63	1022-1027	Neuropharmacology
Ansah, T. A.; Ferguson, M. C.; Nayyar, T.	The 5-HT(2A) Receptor Antagonist M100907 Produces Antiparkinsonian Effects and Decreases Striatal Glutamate	2011	5	48	Front Syst Neurosci
Antonelli, T.; Ferioli, V.; Gallo, G. L.; Tomasini, M. C.; Fernandez, M.; Connor, W. T.; Glennon, J. C.; Tanganelli, S.; Ferraro, L.	Differential effects of acute and short-term lithium administration on dialysate glutamate and GABA levels in the frontal cortex of the conscious rat	2000	38	355-362	Synapse
Antonelli, T.; Fuxe, K.; Agnati, L.; Mazzoni, E.; Tanganelli, S.; Tomasini, M. C.; Ferraro, L.	Experimental studies and theoretical aspects on A2A/D2 receptor interactions in a model of Parkinson's disease. Relevance for L-dopa induced dyskinesias	2006	248	16-22	J Neurol Sci
Antonelli, T.; Fuxe, K.; Tomasini, M. C.; Bartoszyk, G. D.; Seyfried, C. A.; Tanganelli, S.; Ferraro, L.	Effects of sarizotan on the corticostriatal glutamate pathways	2005	58	193-199	Synapse
Antonelli, T.; Tanganelli, S.; Tomasini, M. C.; Finetti, S.; Trabace, L.; Steardo, L.; Sabino, V.; Carratu, M. R.; Cuomo, V.; Ferraro, L.	Long-term effects on cortical glutamate release induced by prenatal exposure to the cannabinoid receptor agonist (R)-(+)-[2,3-dihydro-5-methyl-3-(4-morpholinyl-methyl)pyrrolo[1,2,3-de]-1,4-benzo xazin-6-yl]-1-naphthalenylmethanone: an in vivo microdialysis study in the awake rat	2004	124	367-375	Neuroscience
Arai, N.; Furukawa, N.; Miyamae, T.; Goshima, Y.; Sasaki, Y.; Ohshima, E.; Suzuki, F.; Fujita, K.; Misu, Y.	DOPA cyclohexyl ester, a competitive DOPA antagonist, protects glutamate release and resultant delayed neuron death by transient ischemia in hippocampus CA1 of conscious rats	2001	299	213-216	Neurosci Lett
Ardestani, M. S.; Fathi-Moghaddam, H.	Betamethasone can significantly decrease level of striatal glutamate in animal model of Parkinson's disease	2008	7	229-232	Iran J Pharm Res
Arriaga-Avila, V.; Martinez-Abundis, E.; Cardenas-Morales, B.; Mercado-Gomez,	Lactation reduces stress-caused dopaminergic activity and enhances GABAergic activity in the rat medial prefrontal cortex	2014	52	515-524	J Mol Neurosci

Glutamate					
**Authors**	**Title**	**Year**	**Vol.**	**Pages**	**Journal**
O.; Aburto-Arciniega, E.; Miranda-Martinez, A.; Kendrick, K. M.; Guevara-Guzman, R.					
Arvin, B.; Lekieffre, D.; Graham, J. L.; Moncada, C.; Chapman, A. G.; Meldrum, B. S.	Effect of the non-NMDA receptor antagonist GYKI 52466 on the microdialysate and tissue concentrations of amino acids following transient forebrain ischaemia	1994	62	1458-1467	J Neurochem
Arvin, B.; Moncada, C.; Le Peillet, E.; Chapman, A.; Meldrum, B. S.	GYKI 52466 blocks the increase in extracellular glutamate induced by ischaemia	1992	3	235-238	Neuroreport
Asai, S.; Kohno, T.; Katayama, Y.; Iribe, Y.; Hosoi, I.; Kanematsu, K.; Kunimatsu, T.; Ishikawa, K.	Oxygen-independent real-time monitoring of distinct biphasic glutamate release using dialysis electrode in rat striatum during anoxia: In vivo evaluation of glutamate release and reversed uptake	2000	17	1105-1114	J Neurotrauma
Asakura, K.; Matsuo, Y.; Oshima, T.; Kihara, T.; Minagawa, K.; Araki, Y.; Kagawa, K.; Kanemasa, T.; Ninomiya, M.	omega-agatoxin IVA-sensitive Ca(2+) channel blocker, alpha-eudesmol, protects against brain injury after focal ischemia in rats	2000	394	57-65	Eur J Pharmacol
Ash, E. S.; Heal, D. J.; Clare Stanford, S.	Contrasting changes in extracellular dopamine and glutamate along the rostrocaudal axis of the anterior cingulate cortex of the rat following an acute d-amphetamine or dopamine challenge	2014	87	180-187	Neuropharmacology
Ashworth-Preece, M. A.; Jarrott, B.; Lawrence, A. J.	Nicotinic acetylcholine receptor mediated modulation of evoked excitatory amino acid release in the nucleus tractus solitarius of the rat: evidence from in vivo microdialysis	1998	806	287-291	Brain Res
Ashworth-Preece, M. A.; Jarrott, B.; Lawrence, A. J.	5-Hydroxytryptamine3 receptor modulation of excitatory amino acid release in the rat nucleus tractus solitarius	1995	191	75-78	Neurosci Lett
Ashworth-Preece, M.; Jarrott, B.; Lawrence, A. J.	Nicotinic acetylcholine receptors in the rat and primate nucleus tractus solitarius and on rat and human inferior vagal (nodose) ganglia: evidence from in vivo microdialysis and [125I]alpha-bungarotoxin autoradiography	1998	83	1113-1122	Neuroscience
Azuma, S.; Kodama, T.; Honda, K.; Inoue, S.	State-dependent changes of extracellular glutamate in the medial preoptic area in freely behaving rats	1996	214	179-182	Neurosci Lett
Bacher, A.; Zornow, M. H.	Lamotrigine inhibits extracellular glutamate accumulation during transient global cerebral ischemia in rabbits	1997	86	459-463	Anesthesiology
Badiani, A.; Oates, M. M.; Fraioli, S.; Browman, K. E.; Ostrander, M. M.; Xue,	Environmental modulation of the response to amphetamine: dissociation between changes in behavior and changes in	2000	151	166-174	Psychopharmacology(Berl)

Glutamate					
**Authors**	**Title**	**Year**	**Vol.**	**Pages**	**Journal**
C. J.; Wolf, M. E.; Robinson, T. E.	dopamine and glutamate overflow in the rat striatal complex				
Badr, A. E.; Yin, W.; Mychaskiw, G.; Zhang, J. H.	Effect of hyperbaric oxygen on striatal metabolites: a microdialysis study in awake freely moving rats after MCA occlusion	2001	916	85-90	Brain Res
Bagley, J.; Moghaddam, B.	Temporal dynamics of glutamate efflux in the prefrontal cortex and in the hippocampus following repeated stress: effects of pretreatment with saline or diazepam	1997	77	65-73	Neuroscience
Baker, A. J.; Zornow, M. H.; Grafe, M. R.; Scheller, M. S.; Skilling, S. R.; Smullin, D. H.; Larson, A. A.	Hypothermia prevents ischemia-induced increases in hippocampal glycine concentrations in rabbits	1991	22	666-673	Stroke
Baker, A. J.; Zornow, M. H.; Scheller, M. S.; Yaksh, T. L.; Skilling, S. R.; Smullin, D. H.; Larson, A. A.; Kuczenski, R.	Changes in extracellular concentrations of glutamate, aspartate, glycine, dopamine, serotonin, and dopamine metabolites after transient global ischemia in the rabbit brain	1991	57	1370-1379	J Neurochem
Baker, C. J.; Fiore, A. J.; Frazzini, V. I.; Choudhri, T. F.; Zubay, G. P.; Solomon, R. A.	Intraischemic hypothermia decreases the release of glutamate in the cores of permanent focal cerebral infarcts	1995	36	994-1002	Neurosurgery
Baker, D. A.; Madayag, A.; Kristiansen, L. V.; Meador-Woodruff, J. H.; Haroutunian, V.; Raju, I.	Contribution of cystine-glutamate antiporters to the psychotomimetic effects of phencyclidine	2008	33	1760-1772	Neuropsychopharmacology
Baker, D. A.; Shen, H.; Kalivas, P. W.	Cystine/glutamate exchange serves as the source for extracellular glutamate: modifications by repeated cocaine administration	2002	23	161-162	Amino Acids
Baker, D. A.; Xi, Z. X.; Shen, H.; Swanson, C. J.; Kalivas, P. W.	The origin and neuronal function of in vivo nonsynaptic glutamate	2002	22	9134-9141	J Neurosci
Bakkelund, A. H.; Fonnum, F.; Paulsen, R. E.	Evidence using in vivo microdialysis that aminotransferase activities are important in the regulation of the pools of transmitter amino acids	1993	18	411-415	Neurochem Res
Baldauf, K.; Braun, K.; Gruss, M.	Opiate modulation of monoamines in the chick forebrain: possible role in emotional regulation?	2005	62	149-163	J Neurobiol
Baldwin, H. A.; Williams, J. L.; Snares, M.; Ferreira, T.; Cross, A. J.; Green, A. R.	Attenuation by chlormethiazole administration of the rise in extracellular amino acids following focal ischaemia in the cerebral cortex of the rat	1994	112	188-94	Br J Pharmacol
Ballini, C.; Corte, L. D.; Pazzagli, M.;	Extracellular levels of brain aspartate, glutamate and GABA	2008	106	1035-	J Neurochem

Glutamate					
**Authors**	**Title**	**Year**	**Vol.**	**Pages**	**Journal**
Colivicchi, M. A.; Pepeu, G.; Tipton, K. F.; Giovannini, M. G.	during an inhibitory avoidance response in the rat			1043	
Bamford, N. S.; Robinson, S.; Palmiter, R. D.; Joyce, J. A.; Moore, C.; Meshul, C. K.	Dopamine modulates release from corticostriatal terminals	2004	24	9541-9552	J Neurosci
Baroncelli, L.; Sale, A.; Viegi, A.; Maya Vetencourt, J. F.; De Pasquale, R.; Baldini, S.; Maffei, L.	Experience-dependent reactivation of ocular dominance plasticity in the adult visual cortex	2010	226	100-109	Exp Neurol
Battaglia, G.; Bruno, V.; Pisani, A.; Centonze, D.; Catania, M. V.; Calabresi, P.; Nicoletti, F.	Selective blockade of type-1 metabotropic glutamate receptors induces neuroprotection by enhancing gabaergic transmission	2001	17	1071-1083	Mol Cell Neurosci
Battaglia, G.; Monn, J. A.; Schoepp, D. D.	In vivo inhibition of veratridine-evoked release of striatal excitatory amino acids by the group II metabotropic glutamate receptor agonist LY354740 in rats	1997	229	161-164	Neurosci Lett
Bazzu, G.; Biosa, A.; Farina, D.; Spissu, Y.; Dedola, S.; Calia, G.; Puggioni, G.; Rocchitta, G.; Migheli, R.; Desole, M. S.; Serra, P. A.	Dual asymmetric-flow microdialysis for in vivo monitoring of brain neurochemicals	2011	85	1933-1940	Talanta
Bealer, S. L.; Crowley, W. R.	Histaminergic control of oxytocin release in the paraventricular nucleus during lactation in rats	2001	171	317-322	Exp Neurol
Becker, H. C.; Griffin, W. C.	Effects of chronic intermittent ethanol exposure and drinking on extracellular glutamate levels in the accumbens of C57BL/6J mice	2010	34	80A	Alcohol Clin Exp Res
Becker, H. C.; Griffin, W. C.; Ramachandra, V.; Mulholland, P. J.	Increased glutamate tone in the nucleus accumbens mediates excessive ethanol drinking in dependent mice	2011	36	S187-S188	Neuropsychopharmacology
Beggiato, S.; Antonelli, T.; Tomasini, M. C.; Tanganelli, S.; Fuxe, K.; Schwarcz, R.; Ferraro, L.	Kynurenic acid, by targeting alpha7 nicotinic acetylcholine receptors, modulates extracellular GABA levels in the rat striatum in vivo	2013	37	1470-1477	Eur J Neurosci
Beggiato, S.; Giuliani, A.; Sivilia, S.; Lorenzini, L.; Antonelli, T.; Imbimbo, B. P.; Giardino, L.; Calza, L.; Ferraro, L.	CHF5074 and LY450139 sub-acute treatments differently affect cortical extracellular glutamate levels in pre-plaque Tg2576 mice	2014	266	13-22	Neuroscience
Beggiato, S.; Tanganelli, S.; Fuxe, K.; Antonelli, T.; Schwarcz, R.; Ferraro, L.	Endogenous kynurenic acid regulates extracellular GABA levels in the rat prefrontal cortex	2014	82	11-18	Neuropharmacology
Beggiato, S.; Tomasini, M. C.; Borelli, A.	Functional role of striatal A2A, D2, and mGlu5 receptor	2016	138	254-264	J Neurochem

Glutamate					
**Authors**	**Title**	**Year**	**Vol.**	**Pages**	**Journal**
C.; Borroto-Escuela, D. O.; Fuxe, K.; Antonelli, T.; Tanganelli, S.; Ferraro, L.	interactions in regulating striatopallidal GABA neuronal transmission				
Behrens, P. F.; Franz, P.; Woodman, B.; Lindenberg, K. S.; Landwehrmeyer, G. B.	Impaired glutamate transport and glutamate-glutamine cycling: downstream effects of the Huntington mutation	2002	125	1908-1922	Brain
Behrens, P. F.; Langemann, H.; Strohschein, R.; Draeger, J.; Hennig, J.	Extracellular glutamate and other metabolites in and around RG2 rat glioma: an intracerebral microdialysis study	2000	47	11-22	J Neurooncol
Beitz, A. J.	Relationship of glutamate and aspartate to the periaqueductal gray-raphe magnus projection: analysis using immunocytochemistry and microdialysis	1990	38	1755-1765	J Histochem Cytochem
Beitz, A. J.; Saxon, D.	Harmaline-induced climbing fiber activation causes amino acid and peptide release in the rodent cerebellar cortex and a unique temporal pattern of Fos expression in the olivo-cerebellar pathway	2004	33	49-74	J Neurocytol
Bell, K.; Duffy, P.; Kalivas, P. W.	Context-specific enhancement of glutamate transmission by cocaine	2000	23	335-344	Neuropsychopharmacology
Ben-Shahar, O. M.; Szumlinski, K. K.; Lominac, K. D.; Cohen, A.; Gordon, E.; Ploense, K. L.; Demartini, J.; Bernstein, N.; Rudy, N. M.; Nabhan, A. N.; Sacramento, A.; Pagano, K.; Carosso, G. A.; Woodward, N.	Extended access to cocaine self-administration results in reduced glutamate function within the medial prefrontal cortex	2012	17	746-757	Addict Biol
Benturquia, N.; Parrot, S.; Sauvinet, V.; Renaud, B.; Denoroy, L.	Simultaneous determination of vigabatrin and amino acid neurotransmitters in brain microdialysates by capillary electrophoresis with laser-induced fluorescence detection	2004	806	237-244	J Chromatogr B Analyt Technol Biomed Life Sci
Benveniste, H.; Drejer, J.; Schousboe, A.; Diemer, N. H.	Elevation of the extracellular concentrations of glutamate and aspartate in rat hippocampus during transient cerebral ischemia monitored by intracerebral microdialysis	1984	43	1369-1374	J Neurochem
Bereiter, D. A.; Benetti, A. P.	Excitatory amino release within spinal trigeminal nucleus after mustard oil injection into the temporomandibular joint region of the rat	1996	67	451-459	Pain
Bereiter, D. A.; Shen, S.; Benetti, A. P.	Sex differences in amino acid release from rostral trigeminal subnucleus caudalis after acute injury to the TMJ region	2002	98	89-99	Pain
Berger, C.; Schabitz, W. R.; Wolf, M.;	Hypothermia and brain-derived neurotrophic factor reduce	2004	185	305-312	Exp Neurol

Glutamate					
**Authors**	**Title**	**Year**	**Vol.**	**Pages**	**Journal**
Mueller, H.; Sommer, C.; Schwab, S.	glutamate synergistically in acute stroke				
Berger, C.; Schmid, P. C.; Schabitz, W. R.; Wolf, M.; Schwab, S.; Schmid, H. H.	Massive accumulation of N-acylethanolamines after stroke. Cell signalling in acute cerebral ischemia?	2004	88	1159-1167	J Neurochem
Berger, C.; Stauder, A.; Xia, F.; Sommer, C.; Schwab, S.	Neuroprotection and glutamate attenuation by acetylsalicylic acid in temporary but not in permanent cerebral ischemia	2008	210	543-548	Exp Neurol
Berger, C.; Xia, F.; Maurer, M. H.; Schwab, S.	Neuroprotection by pravastatin in acute ischemic stroke in rats	2008	58	48-56	Brain Res Rev
Berglind, W. J.; Whitfield Jr, T. W.; Lalumiere, R. T.; Kalivas, P. W.; McGinty, J. F.	A single intra-PFC infusion of BDNF prevents cocaineinduced alterations in extracellular glutamate within the nucleus accumbens	2009	29	3715-3719	J Neurosci
Bergquist, F.; Ludwig, M.; Dutia, M. B.	Neuroscience I: Commissural GABA release during vestibular compensation in the rat	2009	37	S28	Amino Acids
Bergquist, F.; Ludwig, M.; Dutia, M. B.	Role of the commissural inhibitory system in vestibular compensation in the rat	2008	586	4441-4452	J Physiol
Bergquist, J.; Vona, M. J.; Stiller, C. O.; Connor, W. T.; Falkenberg, T.; Ekman, R.	Capillary electrophoresis with laser-induced fluorescence detection: a sensitive method for monitoring extracellular concentrations of amino acids in the periaqueductal grey matter	1996	65	33-42	J Neurosci Methods
Bernay, B.; Gaillard, M. C.; Guryca, V.; Emadali, A.; Kuhn, L.; Bertrand, A.; Detraz, I.; Carcenac, C.; Savasta, M.; Brouillet, E.; Garin, J.; Elalouf, J. M.	Discovering new bioactive neuropeptides in the striatum secretome using in vivo microdialysis and versatile proteomics	2009	8	946-958	Mol Cell Proteomics
Bert, L.; Parrot, S.; Robert, F.; Desvignes, C.; Denoroy, L.; Suaud-Chagny, M. F.; Renaud, B.	In vivo temporal sequence of rat striatal glutamate, aspartate and dopamine efflux during apomorphine, nomifensine, NMDA and PDC in situ administration	2002	43	825-835	Neuropharmacology
Bianchi, L.; Ballini, C.; Colivicchi, M. A.; Della Corte, L.; Giovannini, M. G.; Pepeu, G.	Investigation on acetylcholine, aspartate, glutamate and GABA extracellular levels from ventral hippocampus during repeated exploratory activity in the rat	2003	28	565-73	Neurochem Res
Bianchi, L.; Colivicchi, M. A.; Bolam, J. P.; Della Corte, L.	The release of amino acids from rat neostriatum and substantia nigra in vivo: a dual microdialysis probe analysis	1998	87	171-180	Neuroscience
Bianchi, L.; Della Corte, L.; Tipton, K. F.	Simultaneous determination of basal and evoked output levels of aspartate, glutamate, taurine and 4-aminobutyric acid during microdialysis and from superfused brain slices	1999	723	47-59	J Chromatogr B Biomed Sci Appl
Bido, S.; Marti, M.; Morari, M.	Amantadine attenuates levodopa-induced dyskinesia in mice and	2011	118	1043-	J Neurochem

Glutamate					
**Authors**	**Title**	**Year**	**Vol.**	**Pages**	**Journal**
	rats preventing the accompanying rise in nigral GABA levels			1055	
Bie, X.; Chen, Y.; Han, J.; Dai, H.; Wan, H.; Zhao, T.	Effects of gastrodin on amino acids after cerebral ischemia-reperfusion injury in rat striatum	2007	16 Suppl1	305-308	Asia Pac J Clin Nutr
Bielavska, E.; Miksik, I.; Krivanek, J.	Glutamate in the parabrachial nucleus of rats during conditioned taste aversion	2000	887	413-417	Brain Res
Biggs, C. S.; Fowler, L. J.; Whitton, P. S.; Starr, M. S.	Extracellular levels of glutamate and aspartate in the entopeduncular nucleus of the rat determined by microdialysis: regulation by striatal dopamine D2 receptors via the indirect striatal output pathway?	1997	753	163-175	Brain Res
Biggs, C. S.; Pearce, B. R.; Fowler, L. J.; Whitton, P. S.	Effect of isonicotinic acid hydrazide on extracellular amino acids and convulsions in the rat: Reversal of neurochemical and behavioural deficits by sodium valproate	1994	63	2197-2201	J Neurochem
Biggs, C. S.; Pearce, B. R.; Fowler, L. J.; Whitton, P. S.	The effect of sodium valproate on extracellular GABA and other amino acids in the rat ventral hippocampus: an in vivo microdialysis study	1992	594	138-142	Brain Res
Biggs, C. S.; Starr, M. S.	Dopamine and glutamate control each other's release in the basal ganglia: a microdialysis study of the entopeduncular nucleus and substantia nigra	1997	21	497-504	Neurosci Biobehav Rev
Billet, F.; Costentin, J.; Dourmap, N.	Influence of glial cells in the dopamine releasing effect resulting from the stimulation of striatal delta-opioid receptors	2007	150	131-143	Neuroscience
Billet, F.; Dourmap, N.; Costentin, J.	Involvement of corticostriatal glutamatergic terminals in striatal dopamine release elicited by stimulation of delta-opioid receptors	2004	20	2629-2638	Eur J Neurosci
Blanco, L.; Lorigados, L.; Martinez, L.; Pavon, N.; Gonzalez, M. E.; Serrano, T.; Blanco, V.	Neuroprotective effects of MK-801 systemic administration on the pedunculopontine nucleus of hemiparkinsonian rats. [Spanish, English]	2009	26	53-64	Biotecnologia Aplicada
Bland, S. T.; Gonzales, R. A.; Schallert, T.	Movement-related glutamate levels in rat hippocampus, striatum, and sensorimotor cortex	1999	277	119-122	Neurosci Lett
Blanie, A.; Vigue, B.; Benhamou, D.; Duranteau, J.; Geeraerts, T.	The frontal lobe and thalamus have different sensitivities to hypoxia-hypotension after traumatic brain injury: a microdialysis study in rats	2012	29	2782-2790	J Neurotrauma
Bloc, A.; Samuel, D.; Forni, C.; Dusticier, N.; Goff, L. K. L.	Effects of ionotropic excitatory amino acid receptor antagonists on glutamate transport and transport-mediated changes in extracellular excitatory amino acids in the rat striatum	1995	64	1598-1604	J Neurochem

Glutamate					
**Authors**	**Title**	**Year**	**Vol.**	**Pages**	**Journal**
Blum, D.; Galas, M. C.; Pintor, A.; Brouillet, E.; Ledent, C.; Muller, C. E.; Bantubungi, K.; Galluzzo, M.; Gall, D.; Cuvelier, L.; Rolland, A. S.; Popoli, P.; Schiffmann, S. N.	A dual role of adenosine A2A receptors in 3-nitropropionic acid-induced striatal lesions: implications for the neuroprotective potential of A2A antagonists	2003	23	5361-5369	J Neurosci
Blutstein, T.; Baab, P. J.; Ronald Zielke, H.; Mong, J. A.	Hormonal modulation of amino acid neurotransmitter metabolism in the arcuate nucleus of the adult female rat: A novel action of estradiol	2009	150	3237-3244	Endocrinology
Boatell, M. L.; Bendahan, G.; Mahy, N.	Time-related cortical amino acid changes after basal forebrain lesion: a microdialysis study	1995	64	285-291	J Neurochem
Bockelmann, R.; Reiser, M.; Horn, T. F.; Wolf, G.	Influence of nitric oxide synthase activity on amino acid concentration in the quinolinate lesioned rat striatum: a microdialysis and histochemical study	2000	19	423-437	Amino Acids
Bogaert, L.; Neill, M. J.; Moonen, J.; Sarre, S.; Smolders, I.; Ebinger, G.; Michotte, Y.	The effects of LY393613, nimodipine and verapamil, in focal cerebral ischaemia	2001	411	71-83	Eur J Pharmacol
Bogaert, L.; Scheller, D.; Moonen, J.; Sarre, S.; Smolders, I.; Ebinger, G.; Michotte, Y.	Neurochemical changes and laser Doppler flowmetry in the endothelin-1 rat model for focal cerebral ischemia	2000	887	266-275	Brain Res
Bogdanov, M. B.; Tjurmina, O. A.; Wurtman, R. J.	Consumption of a high dietary dose of monosodium glutamate fails to affect extracellular glutamate levels in the hypothalamic arcuate nucleus of adult rats	1996	736	76-81	Brain Res
Bogdanov, M. B.; Wurtman, R. J.	Effects of systemic or oral ad libitum monosodium glutamate administration on striatal glutamate release, as measured using microdialysis in freely moving rats	1994	660	337-340	Brain Res
Boix, J.; Cauli, O.; Leslie, H.; Felipo, V.	Differential long-term effects of developmental exposure to polychlorinated biphenyls 52, 138 or 180 on motor activity and neurotransmission. Gender dependence and mechanisms involved	2011	58	69-77	Neurochem Int
Boix, J.; Llansola, M.; Cabrera-Pastor, A.; Felipo, V.	Metabotropic glutamate receptor 5 modulates the nitric oxide-cGMP pathway in cerebellum in vivo through activation of AMPA receptors	2011	58	599-604	Neurochem Int
Bolea, I.; Colivicchi, M. A.; Ballini, C.;	Protective effect of the MAO-B inhibitor PF9601N in a	2011	8	/	Neurodegener Dis

Glutamate					
**Authors**	**Title**	**Year**	**Vol.**	**Pages**	**Journal**
Fattori, M.; Marco-Contelles, J. L.; Unzeta, M.; Della Corte, L.	microdialysis model of excitotoxicity				Conference: 10th International Conference AD/PD Alzheimer's and Parkinson's Diseases: Advances, Concepts and New Challenges. Barcelona Spain. Conference Start
Bolea, I.; Colivicchi, M. A.; Ballini, C.; Marco-Contelles, J.; Tipton, K. F.; Unzeta, M.; Della Corte, L.	Neuroprotective effects of the MAO-B inhibitor, PF9601N, in an in vivo model of excitotoxicity	2014	20	641-650	CNS Neurosci Ther
Boris-Moller, F.; Wieloch, T.	The effect of 4 beta-phorbol-12,13-dibutyrate and staurosporine on the extracellular glutamate levels during ischemia in the rat striatum	1998	35	133-147	Mol Chem Neuropathol
Boris-Moller, F.; Wieloch, T.	Changes in the extracellular levels of glutamate and aspartate during ischemia and hypoglycemia. Effects of hypothermia	1998	121	277-284	Exp Brain Res
Boris-Moller, F.; Wieloch, T.	The effect of 4beta-phorbol-12,13-dibutyrate and staurosporine on the extracellular glutamate levels during ischemia in the rat striatum	1999	35	133-147	Mol Chem Neuropathol
Borycz, J.; Pereira, M. F.; Melani, A.; Rodrigues, R. J.; Kofalvi, A.; Panlilio, L.; Pedata, F.; Goldberg, S. R.; Cunha, R. A.; Ferre, S.	Differential glutamate-dependent and glutamate-independent adenosine A1 receptor-mediated modulation of dopamine release in different striatal compartments	2007	101	355-363	J Neurochem
Bosch, O. J.; Sartori, S. B.; Singewald, N.; Neumann, I. D.	Extracellular amino acid levels in the paraventricular nucleus and the central amygdala in high- and low-anxiety dams rats during maternal aggression: regulation by oxytocin	2007	10	261-270	Stress
Bosman, D. K.; Deutz, N. E.; Maas, M. A.; van Eijk, H. M.; Smit, J. J.; de Haan, J. G.; Chamuleau, R. A.	Amino acid release from cerebral cortex in experimental acute liver failure, studied by in vivo cerebral cortex microdialysis	1992	59	591-599	J Neurochem
Bottcher, T.; Goiny, M.; Bering, J.; Domhof, S.; Nau, R.; Ungerstedt, U.	Regional differences in glutamine synthetase inhibition by L-methionine sulfoximine: a microdialysis study in the rabbit brain	2003	150	194-200	Exp Brain Res
Bottcher, T.; Ren, H.; Goiny, M.; Gerber, J.; Lykkesfeldt, J.; Kuhn, U.; Lotz, M.; Bunkowski, S.; Werner, C.; Schau, I.; Spreer, A.; Christen, S.; Nau, R.	Clindamycin is neuroprotective in experimental Streptococcus pneumoniae meningitis compared with ceftriaxone	2004	91	1450-1460	J Neurochem

Glutamate					
**Authors**	**Title**	**Year**	**Vol.**	**Pages**	**Journal**
Boulet, S.; Lacombe, E.; Carcenac, C.; Feuerstein, C.; Sgambato-Faure, V.; Poupard, A.; Savasta, M.	Subthalamic stimulation-induced forelimb dyskinesias are linked to an increase in glutamate levels in the substantia nigra pars reticulata	2006	26	10768-10776	J Neurosci
Boulet, S.; Mounayar, S.; Poupard, A.; Bertrand, A.; Jan, C.; Pessiglione, M.; Hirsch, E. C.; Feuerstein, C.; Francois, C.; Feger, J.; Savasta, M.; Tremblay, L.	Behavioral recovery in MPTP-treated monkeys: neurochemical mechanisms studied by intrastriatal microdialysis	2008	28	9575-9584	J Neurosci
Bourne, J. A.; Fosbraey, P.; Halliday, J.	Changes in striatal electroencephalography and neurochemistry induced by kainic acid seizures are modified by dopamine receptor antagonists	2001	413	189-198	Eur J Pharmacol
Boutelle, M. G.; Fellows, L. K.; Cook, C.	Enzyme packed bed system for the on-line measurement of glucose, glutamate, and lactate in brain microdialysate	1992	64	1790-1794	Anal Chem
Bowser, M. T.; Kennedy, R. T.	In vivo monitoring of amine neurotransmitters using microdialysis with on-line capillary electrophoresis	2001	22	3668-3676	Electrophoresis
Bowyer, J. F.; Newport, G. D.; Slikker Jr, W.; Gough, B.; Ferguson, S. A.; Tor-Agbidye, J.	An evaluation of l-ephedrine neurotoxicity with respect to hyperthermia and caudate/putamen microdialysate levels of ephedrine, dopamine, serotonin, and glutamate	2000	55	133-142	Toxicol Sci
Boyd, B. W.; Witowski, S. R.; Kennedy, R. T.	Trace-level amino acid analysis by capillary liquid chromatography and application to in vivo microdialysis sampling with 10-s temporal resolution	2000	72	865-871	Anal Chem
Bradford, H. F.; Young, A. M.; Crowder, J. M.	Continuous glutamate leakage from brain cells is balanced by compensatory high-affinity reuptake transport	1987	81	296-302	Neurosci Lett
Bragina, L.; Marchionni, I.; Omrani, A.; Cozzi, A.; Pellegrini-Giampietro, D. E.; Cherubini, E.; Conti, F.	GAT-1 regulates both tonic and phasic GABA(A) receptor-mediated inhibition in the cerebral cortex	2008	105	1781-1793	J Neurochem
Bredewold, R.; Schiavo, J. K.; van der Hart, M.; Verreij, M.; Veenema, A. H.	Dynamic changes in extracellular release of GABA and glutamate in the lateral septum during social play behavior in juvenile rats: Implications for sex-specific regulation of social play behavior	2015	307	117-127	Neuroscience
Brennan, P. A.; Kendrick, K. M.; Keverne, E. B.	Neurotransmitter release in the accessory olfactory bulb during and after the formation of an olfactory memory in mice	1995	69	1075-1086	Neuroscience
Britton, P.; Whitton, P. S.; Fowler, L. J.; Bowery, N. G.	Tetanus toxin-induced effects on extracellular amino acid levels in rat hippocampus: an in vivo microdialysis study	1996	67	324-329	J Neurochem
Brodie, M. E.; Opacka-Juffry, J.; Peterson,	Neurochemical changes in hippocampal and caudate dialysates	1990	11	35-46	NeuroToxicology

Glutamate					
Authors	Title	Year	Vol.	Pages	Journal
D. W.; Brown, A. W.	associated with early trimethyltin neurotoxicity in rats				
Brown, A. M.; Skamarauskas, J.; Lister, T.; Madjd, A.; Ray, D. E.	Differential susceptibility of astrocytic and neuronal function to 3-chloropropanediol in the rat inferior colliculus	2011	116	996-1004	J Neurochem
Bruet, N.; Windels, F.; Carcenac, C.; Feuerstein, C.; Bertrand, A.; Poupard, A.; Savasta, M.	Neurochemical mechanisms induced by high frequency stimulation of the subthalamic nucleus: increase of extracellular striatal glutamate and GABA in normal and hemiparkinsonian rats	2003	62	1228-1240	J Neuropathol Exp Neurol
Brugnoli, A.; Napolitano, F.; Usiello, A.; Morari, M.	Genetic deletion of Rhes or pharmacological blockade of mTORC1 prevent striato-nigral neurons activation in levodopa-induced dyskinesia	2016	85	155-163	Neurobiol Dis
Bruhn, T.; Christensen, T.; Diemer, N. H.	In vivo cellular uptake of glutamate is impaired in the rat hippocampus during and after transient cerebral ischemia: a microdialysis extraction study	2001	66	1118-1126	J Neurosci Res
Bruhn, T.; Christensen, T.; Diemer, N. H.	Evidence for increased cellular uptake of glutamate and aspartate in the rat hippocampus during kainic acid seizures. A microdialysis study using the "indicator diffusion' method	1997	26	363-371	Epilepsy Res
Bruno, V.; Battaglia, G.; Ksiazek, I.; van der Putten, H.; Catania, M. V.; Giuffrida, R.; Lukic, S.; Leonhardt, T.; Inderbitzin, W.; Gasparini, F.; Kuhn, R.; Hampson, D. R.; Nicoletti, F.; Flor, P. J.	Selective activation of mGlu4 metabotropic glutamate receptors is protective against excitotoxic neuronal death	2000	20	6413-6420	J Neurosci
Buck, K.; Voehringer, P.; Ferger, B.	Rapid analysis of GABA and glutamate in microdialysis samples using high performance liquid chromatography and tandem mass spectrometry	2009	182	78-84	J Neurosci Methods
Buisson, A.; Callebert, J.; Mathieu, E.; Plotkine, M.; Boulu, R. G.	Striatal protection induced by lesioning the substantia nigra of rats subjected to focal ischemia	1992	59	1153-1157	J Neurochem
Bullock, R.; Butcher, S. P.; Chen, M. H.; Kendall, L.; McCulloch, J.	Correlation of the extracellular glutamate concentration with extent of blood flow reduction after subdural hematoma in the rat	1991	74	794-802	J Neurosurg
Buratta, S.; Hamberger, A.; Ryberg, H.; Nystrom, B.; Sandberg, M.; Mozzi, R.	Effect of serine and ethanolamine administration on phospholipid-related compounds and neurotransmitter amino acids in the rabbit hippocampus	1998	71	2145-2150	J Neurochem
Burgess, C.; Lai, D.; Siegel, J.; Peever, J.	An endogenous glutamatergic drive onto somatic motoneurons contributes to the stereotypical pattern of muscle tone across the sleep-wake cycle	2008	28	4649-4660	J Neurosci

Glutamate					
**Authors**	**Title**	**Year**	**Vol.**	**Pages**	**Journal**
Bustamante, D.; You, Z. B.; Castel, M. N.; Johansson, S.; Goiny, M.; Terenius, L.; Hokfelt, T.; Herrera-Marschitz, M.	Effect of single and repeated methamphetamine treatment on neurotransmitter release in substantia nigra and neostriatum of the rat	2002	83	645-654	J Neurochem
Busto, R.; Globus, M. Y.; Dietrich, W. D.; Martinez, E.; Valdes, I.; Ginsberg, M. D.	Effect of mild hypothermia on ischemia-induced release of neurotransmitters and free fatty acids in rat brain	1989	20	904-910	Stroke
Bustos, G.; Abarca, J.; Forray, M. I.; Gysling, K.; Bradberry, C. W.; Roth, R. H.	Regulation of excitatory amino acid release by N-methyl-D-aspartate receptors in rat striatum: in vivo microdialysis studies	1992	585	105-115	Brain Res
Butcher, S. P.; Bullock, R.; Graham, D. I.; McCulloch, J.	Correlation between amino acid release and neuropathologic outcome in rat brain following middle cerebral artery occlusion	1990	21	1727-1733	Stroke
Butcher, S. P.; Cameron, D.; Kendall, L.; Griffiths, R.	Homocysteine-induced alterations in extracellular amino acids in rat hippocampus	1992	20	75-80	Neurochem Int
Butcher, S. P.; Hamberger, A.	In vivo studies on the extracellular, and veratrine-releasable, pools of endogenous amino acids in the rat striatum: effects of corticostriatal deafferentation and kainic acid lesion	1987	48	713-721	J Neurochem
Butcher, S. P.; Jacobson, I.; Hamberger, A.	On the epileptogenic effects of kainic acid and dihydrokainic acid in the dentate gyrus of the rat	1988	27	375-381	Neuropharmacology
Butcher, S. P.; Lazarewicz, J. W.; Hamberger, A.	In vivo microdialysis studies on the effects of decortication and excitotoxic lesions on kainic acid-induced calcium fluxes, and endogenous amino acid release, in the rat striatum	1987	49	1355-1360	J Neurochem
Butcher, S. P.; Sandberg, M.; Hagberg, H.; Hamberger, A.	Cellular origins of endogenous amino acids released into the extracellular fluid of the rat striatum during severe insulin-induced hypoglycemia	1987	48	722-728	J Neurochem
Butts, K. A.; Phillips, A. G.	Glucocorticoid receptors in the prefrontal cortex regulate dopamine efflux to stress via descending glutamatergic feedback to the ventral tegmental area	2013	16	1799-1807	Int J Neuropsychopharmacol
Cabrera-Pastor, A.; Taoro, L.; Llansola, M.; Felipo, V.	Roles of the NMDA Receptor and EAAC1 Transporter in the Modulation of Extracellular Glutamate by Low and High Affinity AMPA Receptors in the Cerebellum in Vivo: Differential Alteration in Chronic Hyperammonemia	2015	6	1913-1921	ACS Chem Neurosci
Caille, S.; Parsons, L. H.	Intravenous heroin self-administration decreases GABA efflux in the ventral pallidum: an in vivo microdialysis study in rats	2004	20	593-596	Eur J Neurosci
Calatrava-Ferreras, L.; Gonzalo-Gobernado, R.; Reimers, D.; Herranz, A.	Neuroprotective role of liver growth factor "LGF" in an experimental model of cerebellar ataxia	2014	15	19056-19073	Int J Mol Sci

Glutamate					
Authors	Title	Year	Vol.	Pages	Journal
S.; Jimenez-Escrig, A.; Diaz-Gil, J. J.; Casarejos, M. J.; Montero-Vega, M. T.; Bazan, E.					
Calcagno, E.; Carli, M.; Baviera, M.; Invernizzi, R. W.	Endogenous serotonin and serotonin2C receptors are involved in the ability of M100907 to suppress cortical glutamate release induced by NMDA receptor blockade	2009	108	521-532	J Neurochem
Calcagno, E.; Carli, M.; Invernizzi, R. W.	The 5-HT(1A) receptor agonist 8-OH-DPAT prevents prefrontocortical glutamate and serotonin release in response to blockade of cortical NMDA receptors	2006	96	853-860	J Neurochem
Callaway, J. K.; Castillo-Melendez, M.; Giardina, S. F.; Krstew, E. K.; Beart, P. M.; Jarrott, B.	Sodium channel blocking activity of AM-36 and sipatrigine (BW619C89): in vitro and in vivo evidence	2004	47	146-155	Neuropharmacology
Camarini, R.; Griffin, Iii W. C.; Yanke, A. B.; Rosalina dos Santos, B.; Olive, M. F.	Effects of adolescent exposure to cocaine on locomotor activity and extracellular dopamine and glutamate levels in nucleus accumbens of DBA/2J mice	2008	1193	34-42	Brain Researc
Campos, F.; Alfonso, M.; Duran, R.	In vivo modulation of alpha7 nicotinic receptors on striatal glutamate release induced by anatoxin-A	2010	56	850-855	Neurochem Int
Canales, J. J.; Elayadi, A.; Errami, M.; Llansola, M.; Cauli, O.; Felipo, V.	Chronic hyperammonemia alters motor and neurochemical responses to activation of group I metabotropic glutamate receptors in the nucleus accumbens in rats in vivo	2003	14	380-390	Neurobiol Dis
Cantor, S. L.; Zornow, M. H.; Miller, L. P.; Yaksh, T. L.	The effect of cyclohexyladenosine on the periischemic increases of hippocampal glutamate and glycine in the rabbit	1992	59	1884-1892	J Neurochem
Cao, J. L.; Zhang, Y. H.; Gu, J.; Zhou, W. H.; Yang, G. D.; Zeng, Y. M.	[Application of combination of capillary electrophoresis with laser-induced fluorescence: measurement of glutamate and arginine in PAG microdialytes of conscions morphine-withdrawal rats]	2003	55	612-616	Sheng Li Xue Bao
Carboni, S.; Isola, R.; Gessa, G. L.; Rossetti, Z. L.	Ethanol prevents the glutamate release induced by N-methyl-D-aspartate in the rat striatum	1993	152	133-136	Neurosci Lett
Carboni, S.; Melis, F.; Pani, L.; Hadjiconstantinou, M.; Rossetti, Z. L.	The non-competitive NMDA-receptor antagonist MK-801 prevents the massive release of glutamate and aspartate from rat striatum induced by 1-methyl-4-phenylpyridinium (MPP+)	1990	117	129-133	Neurosci Lett
Carboni, S.; Melis, F.; Vaccari, A.; Rossetti, Z. L.	NMDA receptor activation mediates glutamate and aspartate release from rat striatum: prevention by MK-801	1990	22	49	Pharmacol Res
Carboni, S.; Rossetti, Z. L.	Increased extracellular glutamate in rat prefrontal cortex:	2001	890	189-191	Brain Res

Glutamate					
**Authors**	**Title**	**Year**	**Vol.**	**Pages**	**Journal**
	movement artifact contribution				
Caringi, D.; Maher, T. J.; Chaiyakul, P.; Asmundsson, G.; Ishide, T.; Ally, A.	Extracellular glutamate increases in rostral ventrolateral medulla during static muscle contraction	1998	435	465-471	Pflugers Arch
Carpenedo, R.; Pittaluga, A.; Cozzi, A.; Attucci, S.; Galli, A.; Raiteri, M.; Moroni, F.	Presynaptic kynurenate-sensitive receptors inhibit glutamate release	2001	13	2141-2147	Eur J Neurosci
Carrara-Nascimento, P. F.; Griffin, W. C., 3rd; Pastrello, D. M.; Olive, M. F.; Camarini, R.	Changes in extracellular levels of glutamate in the nucleus accumbens after ethanol-induced behavioral sensitization in adolescent and adult mice	2011	45	451-460	Alcohol
Cassel, G. E.; Fosbraey, P.	Measurement of the oxime HI-6 after peripheral administration in tandem with neurotransmitter levels in striatal dialysates: effects of soman intoxication	1996	35	159-166	J Pharmacol Toxicol Methods
Castaldo, P.; Magi, S.; Gaetani, S.; Cassano, T.; Ferraro, L.; Antonelli, T.; Amoroso, S.; Cuomo, V.	Prenatal exposure to the cannabinoid receptor agonist WIN 55,212-2 increases glutamate uptake through overexpression of GLT1 and EAAC1 glutamate transporter subtypes in rat frontal cerebral cortex	2007	53	369-378	Neuropharmacology
Castillo-Melendez, M.; Krstew, E.; Lawrence, A. J.; Jarrott, B.	Presynaptic adenosine A2a receptors on soma and central terminals of rat vagal afferent neurons	1994	652	137-144	Brain Res
Cauli, O.; Llansola, M.; Erceg, S.; Felipo, V.	Hypolocomotion in rats with chronic liver failure is due to increased glutamate and activation of metabotropic glutamate receptors in substantia nigra	2006	45	654-661	J Hepatol
Cauli, O.; Mlili, N.; Llansola, M.; Felipo, V.	Motor activity is modulated via different neuronal circuits in rats with chronic liver failure than in normal rats	2007	25	2112-2122	Eur J Neurosci
Cauli, O.; Mlili, N.; Rodrigo, R.; Felipo, V.	Hyperammonaemia alters the mechanisms by which metabotropic glutamate receptors in nucleus accumbens modulate motor function	2007	103	38-46	J Neurochem
Cauli, O.; Rodrigo, R.; Piedrafita, B.; Llansola, M.; Mansouri, M. T.; Felipo, V.	Neuroinflammation contributes to hypokinesia in rats with hepatic encephalopathy: ibuprofen restores its motor activity	2009	87	1369-1374	J Neurosci Res
Ceglia, I.; Carli, M.; Baviera, M.; Renoldi, G.; Calcagno, E.; Invernizzi, R. W.	The 5-HT receptor antagonist M100,907 prevents extracellular glutamate rising in response to NMDA receptor blockade in the mPFC	2004	91	189-199	J Neurochem
Cellar, N. A.; Burns, S. T.; Meiners, J. C.; Chen, H.; Kennedy, R. T.	Microfluidic chip for low-flow push-pull perfusion sampling in vivo with on-line analysis of amino acids	2005	77	7067-7073	Anal Chem
Chaitoff, K. A.; Patel, D.; Ally, A.	Effects of endothelial NOS antagonism within the periaqueductal	2008	1236	93-104	Brain Res

Glutamate					
Authors	Title	Year	Vol.	Pages	Journal
	gray on cardiovascular responses and neurotransmission during mechanical, heat, and cold nociception				
Chang, C. H.; Huang, W. T.; Kao, C. H.; Chen, S. H.; Lin, C. H.	Tetramethylpyrazine decreases hypothalamic glutamate, hydroxyl radicals and prostaglandin-E2 and has antipyretic effects	2013	62	527-535	Inflamm Res
Chang, H. P.; Ma, Y. L.; Wan, F. J.; Tsai, L. Y.; Lindberg, F. P.; Lee, E. H.	Functional blocking of integrin-associated protein impairs memory retention and decreases glutamate release from the hippocampus	2001	102	289-296	Neuroscience
Chapman, M. A.; See, R. E.	Differential effects of unique profile antipsychotic drugs on extracellular amino acids in the ventral pallidum and globus pallidus of rats	1996	277	1586-1594	J Pharmacol Exp Ther
Chefer, V. I.; Denoroy, L.; Zapata, A.; Shippenberg, T. S.	Mu opioid receptor modulation of somatodendritic dopamine overflow: GABAergic and glutamatergic mechanisms	2009	30	272-278	Eur J Neurosci
Chefer, V. I.; Wang, R.; Shippenberg, T. S.	Basolateral amygdala-driven augmentation of medial prefrontal cortex GABAergic neurotransmission in response to environmental stimuli associated with cocaine administration	2011	36	2018-2029	Neuropsypharmacology
Chefer, V.; Meis, J.; Wang, G.; Kuzmin, A.; Bakalkin, G.; Shippenberg, T.	Repeated exposure to moderate doses of ethanol augments hippocampal glutamate neurotransmission by increasing release	2011	16	229-237	Addict Biol
Chen, J. C.; Liang, K. W.; Huang, Y. K.; Liang, C. S.; Chiang, Y. C.	Significance of glutamate and dopamine neurons in the ventral pallidum in the expression of behavioral sensitization to amphetamine	2001	68	973-983	Life Sci
Chen, J.; Adachi, N.; Tsubota, S.; Nagaro, T.; Arai, T.	Dexamethasone augments ischemia-induced extracellular accumulation of glutamate in gerbil hippocampus	1998	347	67-70	Eur J Pharmacol
Chen, J.; Graham, S. H.; Simon, R. P.	A comparison of the effects of a sodium channel blocker and an NMDA antagonist upon extracellular glutamate in rat focal cerebral ischemia	1995	699	121-124	Brain Res
Chen, J.; Nam, H. W.; Lee, M. R.; Hinton, D. J.; Choi, S.; Kim, T.; Kawamura, T.; Janak, P. H.; Choi, D. S.	Altered glutamatergic neurotransmission in the striatum regulates ethanol sensitivity and intake in mice lacking ENT1	2010	208	636-642	Behav Brain Res
Chen, J.; Xu, W.; Jiang, H.	17 beta-estradiol protects neurons from ischemic damage and attenuates accumulation of extracellular excitatory amino acids	2001	92	1520-1523	Anesth Analg
Chen, N.; Meng, F. G.; Zhang, J. G.; Yang, A. C.; Liu, H. G.; Hu, W. H.; Meng,	[Changes of glutamate and gamma-aminobutyric acid of extracellular fluid in hippocampus during electrical stimulation of	2012	92	3371-3373	Zhonghua Yi Xue Za Zhi

Glutamate					
**Authors**	**Title**	**Year**	**Vol.**	**Pages**	**Journal**
D. W.; Zhang, X.; Liu, C.; Ge, Y.	anterior nucleus thalamus in rats]				
Chen, R.; Deng, Y.; Yang, L.; Wang, J.; Xu, F.	Determination of Histamine by High-Performance Liquid Chromatography After Precolumn Derivatization with o-Phthalaldehyde-Sulfite	2016	54	547-553	J Chromatogr Sci
Chen, R.; Deng, Y.; Yao, J.; Kamal, G. M.; Wang, J.; Xu, F.	Assessment of amino acid neurotransmitters in rat brain microdialysis samples by high-performance liquid chromatography with coulometric detection	2015	38	1439-1447	J Liq Chromatogr
Chen, Y. L.; Li, D.; Wang, Z. Z.; Xu, W. G.; Li, R. P.; Zhang, J. D.	Glutamate metabolism of astrocytes during hyperbaric oxygen exposure and its effects on central nervous system oxygen toxicity	2016	27	73-79	Neuroreport
Chen, Y.; Engidawork, E.; Loidl, F.; Dell; Anna, E.; Goiny, M.; Lubec, G.; Andersson, K.; Herrera-Marschitz, M.	Short- and long-term effects of perinatal asphyxia on monoamine, amino acid and glycolysis product levels measured in the basal ganglia of the rat	1997	104	19-30	Brain Res Dev Brain Res
Cherng, C. H.; Wong, C. S.; Wu, C. T.; Yeh, C. C.	Intrathecal glutamate release during hindlimb tourniquet inflation and femoral artery occlusion in rats	2013	112	259-262	J Formos Med Assoc
Chio, C. C.; Kuo, J. R.; Hsiao, S. H.; Chang, C. P.; Lin, M. T.	Effect of brain cooling on brain ischemia and damage markers after fluid percussion brain injury in rats	2007	28	284-290	Shock
Cho, C.	Striatal glutamate and GABA after high frequency subthalamic stimulation in parkinsonian rat	2015	15	1313	Neurodegener Dis
Choi, K. T.; Chung, J. K.; Kwak, C. S.; Kim, H. K.	Effect of hypocapnia on extracellular glutamate and glycine concentrations during the periischemic period in rabbit hippocampus	1995	765	86-97	Ann N Y Acad Sci
Choi, K. T.; Chung, J. K.; Kwak, C. S.; Kim, H. K.	Effect of hypocapnia on extracellular glutamate and glycine concentrations during peri-ischemic period in the rabbit hippocampus	1994	9	394-401	J Korean Med Sci
Choi, K. T.; Illievich, U. M.; Zornow, M. H.; Scheller, M. S.; Strnat, M. A.	Effect of hyperglycemia on peri-ischemic neurotransmitter levels in the rabbit hippocampus	1994	642	104-110	Brain Res
Choi, S. K.; Lee, G. J.; Choi, S.; Kim, Y. J.; Park, H. K.; Park, B. J.	Neuroprotective effects by nimodipine treatment in the experimental global ischemic rat model : real time estimation of glutamate	2011	49	1-7	J Korean Neurosurg Soc
Choi, Y. H.; Chang, N.; Fletcher, P. J.; Anderson, G. H.	Dietary protein content affects the profiles of extracellular amino acids in the medial preoptic area of freely moving rats	2000	66	1105-1118	Life Sci
Choi, Y. H.; Fletcher, P. J.; Anderson, G.	Extracellular amino acid profiles in the paraventricular nucleus of	2001	892	320-328	Brain Res

Glutamate					
**Authors**	**Title**	**Year**	**Vol.**	**Pages**	**Journal**
H.	the rat hypothalamus are influenced by diet composition				
Choi, Y. H.; Fletcher, P. J.; Wong, C. C.; Anderson, G. H.	Measurement of blood-brain barrier permeability of rats with alpha-aminoisobutyric acid during microdialysis: possible application to behavioral studies	1999	67	587-598	Physiol Behav
Christensen, T.; Bruhn, T.; Diemer, N. H.; Schousboe, A.	Effect of phenylsuccinate on potassium- and ischemia-induced release of glutamate in rat hippocampus monitored by microdialysis	1991	134	71-74	Neurosci Lett
Ciriacks, C. M.; Bowser, M. T.	Measuring the effect of glutamate receptor agonists on extracellular D-serine concentrations in the rat striatum using online microdialysis-capillary electrophoresis	2006	393	200-205	Neurosci Lett
Ciriacks, C. M.; Bowser, M. T.	Monitoring D-serine dynamics in the rat brain using online microdialysis-capillary electrophoresis	2004	76	6582-6587	Anal Chem
Clausen, T.; Scharf, A.; Menzel, M.; Soukup, J.; Holz, C.; Rieger, A.; Hanisch, F.; Brath, E.; Nemeth, N.; Miko, I.; Vajkoczy, P.; Radke, J.; Henze, D.	Influence of moderate and profound hyperventilation on cerebral blood flow, oxygenation and metabolism	2004	1019	113-123	Brain Res
Clausen, T.; Zauner, A.; Levasseur, J. E.; Rice, A. C.; Bullock, R.	Induced mitochondrial failure in the feline brain: implications for understanding acute post-traumatic metabolic events	2001	908	35-48	Brain Res
Clement, H. W.; Pschibul, A.; Schulz, E.	Effects of secretin on extracellular GABA and other amino acid concentrations in the rat hippocampus	2005	71	239-271	Int Rev Neruobiol
Clinckers, R.; Gheuens, S.; Smolders, I.; Meurs, A.; Ebinger, G.; Michotte, Y.	In vivo modulatory action of extracellular glutamate on the anticonvulsant effects of hippocampal dopamine and serotonin	2005	46	828-836	Epilepsia
Clinckers, R.; Zgavc, T.; Vermoesen, K.; Meurs, A.; Michotte, Y.; Smolders, I.	Pharmacological and neurochemical characterization of the involvement of hippocampal adrenoreceptor subtypes in the modulation of acute limbic seizures	2010	115	1595-1607	J Neurochem
Cobo, M.; Bruhn, T.; Berg, M.; Diemer, N. H.	Phenylsuccinate reduces KCL-induced release of GABA evidence for the participation of the ketodicarboxylate carrier in the biosynthesis of transmitter-GABA	1993	5	377-388	Amino Acids
Cohen Jr, M. M.	Effects of lubeluzole on the methamphetamine-induced increase in extracellular glutamate and the long-term depletion of striatal dopamine	2001	40	95-101	Synapse
Collins, S. A.; Gudelsky, G. A.; Yamamoto, B. K.	MDMA-induced loss of parvalbumin interneurons within the dentate gyrus is mediated by 5HT2A and NMDA receptors	2015	761	95-100	Eur J Pharmacol

Glutamate					
**Authors**	**Title**	**Year**	**Vol.**	**Pages**	**Journal**
Conroy, B. P.; Black, D.	Lamotrigine attenuates cortical glutamate release during global cerebral ischemia in pigs on cardiopulmonary bypass	1999	90	844-854	Anesthesiology
Conroy, B. P.; Lin, C. Y.; Jenkins, L. W.; DeWitt, D. S.; Zornow, M. H.; Uchida, T.; Johnston, W. E.	Hypothermic modulation of cerebral ischemic injury during cardiopulmonary bypass in pigs	1998	88	390-402	Anesthesiology
Cook, C. J.	Patterns of weaning and adult response to stress	1999	67	803-808	Physiol Behav
Cook, C. J.	Real-time extracellular measurement of neurotransmitters in conscious sheep	1997	72	161-166	J Neurosci Methods
Cook, C. J.; Maasland, S. A.; Devine, C. E.	Social behaviour in sheep relates to behaviour and neurotransmitter responses to nociceptive stimuli	1996	60	741-751	Physiol Behav
Corsi, C.; Melani, A.; Bianchi, L.; Pedata, F.	Striatal A2A adenosine receptor antagonism differentially modifies striatal glutamate outflow in vivo in young and aged rats	2000	11	2591-2595	Neuroreport
Corsi, C.; Melani, A.; Bianchi, L.; Pepeu, G.; Pedata, F.	Striatal A2A adenosine receptors differentially regulate spontaneous and K+-evoked glutamate release in vivo in young and aged rats	1999	10	687-691	Neuroreport
Corsi, C.; Pazzagli, M.; Bianchi, L.; Della Corte, L.; Pepeu, G.; Pedata, F.	In vivo amino acid release from the striatum of aging rats: adenosine modulation	1997	18	243-250	Neurobiol Aging
Corsi, C.; Pinna, A.; Gianfriddo, M.; Melani, A.; Morelli, M.; Pedata, F.	Adenosine A2A receptor antagonism increases striatal glutamate outflow in dopamine-denervated rats	2003	464	33-38	Eur J Pharmacol
Cozzi, A.; Attucci, S.; Peruginelli, F.; Marinozzi, M.; Luneia, R.; Pellicciari, R.; Moroni, F.	Type 2 metabotropic glutamate (mGlu) receptors tonically inhibit transmitter release in rat caudate nucleus: in vivo studies with (2S,1'S,2'S,3'R)-2-(2'-carboxy-3'-phenylcyclopropyl)glycine, a new potent and selective antagonist	1997	9	1350-1355	Eur J Neurosci
Currie, P. J.; Chang, N.; Luo, S.; Anderson, G. H.	Microdialysis as a tool to measure dietary and regional effects on the complete profile of extracellular amino acids in the hypothalamus of rats	1995	57	1911-1923	Life Sci
Czell, D.; Efe, T.; Preuss, M.; Schofer, M. D.; Becker, R.	Influence of Intraventricular Application of Baclofen on Arterial Blood Pressure and Neurotransmitter Concentrations in the Hypothalamic Paraventricular Nucleus of Rats	2012	37	381-386	Neurochem Res
Da Costa, A. P.; De La Riva, C.; Guevara-Guzman, R.; Kendrick, K. M.	C-fos and c-jun in the paraventricular nucleus play a role in regulating peptide gene expression, oxytocin and glutamate release, and maternal behaviour	1999	11	2199-2210	Eur J Neurosci
Da Costa, A. P.; Guevara-Guzman, R. G.;	The role of oxytocin release in the paraventricular nucleus in the	1996	8	163-177	J Neuroendocrinol

Glutamate					
**Authors**	**Title**	**Year**	**Vol.**	**Pages**	**Journal**
Ohkura, S.; Goode, J. A.; Kendrick, K. M.	control of maternal behaviour in the sheep				
Dahchour, A.; De Witte, P.	Effect of repeated ethanol withdrawal on glutamate microdialysate in the hippocampus	1999	23	1698-1703	Alcohol Clin Exp Res
Dahchour, A.; De Witte, P.	Taurine blocks the glutamate increase in the nucleus accumbens microdialysate of ethanol-dependent rats	2000	65	345-350	Pharmacol Biochem Behav
Dahchour, A.; De Witte, P.	Acamprosate decreases the hypermotility during repeated ethanol withdrawal	1999	18	77-81	Alcohol
Dahchour, A.; De Witte, P.	Excitatory and inhibitory amino acid changes during repeated episodes of ethanol withdrawal: an in vivo microdialysis study	2003	459	171-178	Eur J Pharmacol
Dahchour, A.; De Witte, P.	Effects of acamprosate on excitatory amino acids during multiple ethanol withdrawal periods	2003	27	465-470	Alcohol Clin Exp Res
Dahchour, A.; Hoffman, A.; Deitrich, R.; de Witte, P.	Effects of ethanol on extracellular amino acid levels in high-and low-alcohol sensitive rats: a microdialysis study	2000	35	548-553	Alcohol Alcohol
Dahchour, A.; Quertemont, E.; De Witte, P.	Taurine increases in the nucleus accumbens microdialysate after acute ethanol administration to naive and chronically alcoholised rats	1996	735	9-19	Brain Res
Dahlbacka, S.; Makela, J.; Kaakinen, T.; Alaoja, H.; Heikkinen, J.; Laurila, P.; Kiviluoma, K.; Salomaki, T.; Tuominen, H.; Ohtonen, P.; Lepola, P.; Biancari, F.; Juvonen, T.	Propofol is associated with impaired brain metabolism during hypothermic circulatory arrest: An experimental microdialysis study	2006	9	E710-E718	Heart Surg Forum
Dahlin, A. P.; Purins, K.; Clausen, F.; Chu, J.; Sedigh, A.; Lorant, T.; Enblad, P.; Lewen, A.; Hillered, L.	Refined microdialysis method for protein biomarker sampling in acute brain injury in the neurointensive care setting	2014	86	8671-8679	Anal Chem
Daisley, J. N.; Gruss, M.; Rose, S. P.; Braun, K.	Passive avoidance training and recall are associated with increased glutamate levels in the intermediate medial hyperstriatum ventrale of the day-old chick	1998	6	53-61	Neural Plast
Dalley, J. W.	Effects of excitotoxic lesions of the rat prefrontal cortex on CREB regulation and presynaptic markers of dopamine and amino acid function in the nucleus accumbens	1999	11	1265-1274	Eur J Neurosci
Daly, D. A.; Moghaddam, B.	Actions of clozapine and haloperidol on the extracellular levels of excitatory amino acids in the prefrontal cortex and striatum of conscious rats	1993	152	61-64	Neurosci Lett

Glutamate					
**Authors**	**Title**	**Year**	**Vol.**	**Pages**	**Journal**
Darbin, O.; Risso, J. J.; Rostain, J. C.	Helium-oxygen pressure induces striatal glutamate increase: a microdialysis study in freely-moving rats	2001	297	37-40	Neurosci Lett
Darling, B. K.; Abdel-Rahim, M.; Moores, R. R.; Chang, A. S.; Howard, R. S.; Neill, J. T.	Brain excitatory amino acid concentrations are lower in the neonatal pig: a buffer against excitotoxicity?	2001	80	305-312	Biol Neonate
Darna, M.; Beckmann, J. S.; Gipson, C. D.; Bardo, M. T.; Dwoskin, L. P.	Effect of environmental enrichment on dopamine and serotonin transporters and glutamate neurotransmission in medial prefrontal and orbitofrontal cortex	2015	1599	115-125	Brain Res
Darvesh, A. S.; Carroll, R. T.; Geldenhuys, W. J.; Gudelsky, G. A.; Klein, J.; Meshul, C. K.; Van der Schyf, C. J.	In vivo brain microdialysis: advances in neuropsychopharmacology and drug discovery	2011	6	109-127	Expert Opin Drug Discov
Das, S. C.; Yamamoto, B. K.; Hristov, A. M.; Sari, Y.	Ceftriaxone attenuates ethanol drinking and restores extracellular glutamate concentration through normalization of GLT-1 in nucleus accumbens of male alcohol-preferring rats	2015	97	67-74	Neuropharmacology
Dassesse, D.; Massie, A.; Ferrari, R.; Ledent, C.; Parmentier, M.; Arckens, L.; Zoli, M.; Schiffmann, S. N.	Functional striatal hypodopaminergic activity in mice lacking adenosine A(2A) receptors	2001	78	183-198	J Neurochem
Dave, K. R.; Lange-Asschenfeldt, C.; Raval, A. P.; Prado, R.; Busto, R.; Saul, I.; Perez-Pinzon, M. A.	Ischemic preconditioning ameliorates excitotoxicity by shifting glutamate/gamma-aminobutyric acid release and biosynthesis	2005	82	665-673	J Neurosci Res
David, C. N.; Frias, E. S.; Szu, J. I.; Vieira, P. A.; Hubbard, J. A.; Lovelace, J.; Michael, M.; Worth, D.; McGovern, K. E.; Ethell, I. M.; Stanley, B. G.; Korzus, E.; Fiacco, T. A.; Binder, D. K.; Wilson, E. H.	GLT-1-Dependent Disruption of CNS Glutamate Homeostasis and Neuronal Function by the Protozoan Parasite Toxoplasma gondii	2016	12	e1005643	PLoS Pathog
Dawson, L. A.; Djali, S.; Gonzales, C.; Vinegra, M. A.; Zaleska, M. M.	Characterization of transient focal ischemia-induced increases in extracellular glutamate and aspartate in spontaneously hypertensive rats	2000	53	767-776	Brain Res Bull
Dawson, L. A.; Li, P.	Effects of 5-HT(6) receptor blockade on the neurochemical outcome of antidepressant treatment in the frontal cortex of the rat	2003	110	577-590	J Neural Transm (Vienna)
Dawson, L. A.; Nguyen, H. Q.; Li, P.	In vivo effects of the 5-HT(6) antagonist SB-271046 on striatal and frontal cortex extracellular concentrations of noradrenaline,	2000	130	23-26	Br J Pharmacol

Glutamate					
Authors	Title	Year	Vol.	Pages	Journal
	dopamine, 5-HT, glutamate and aspartate				
Dawson, L. A.; Nguyen, H. Q.; Li, P.	The 5-HT(6) receptor antagonist SB-271046 selectively enhances excitatory neurotransmission in the rat frontal cortex and hippocampus	2001	25	662-668	Neuropsychopharmacology
Dawson, L. A.; Organ, A. J.; Winter, P.; Lacroix, L. P.; Shilliam, C. S.; Heidbreder, C.; Shah, A. J.	Rapid high-throughput assay for the measurement of amino acids from microdialysates and brain tissue using monolithic C18-bonded reversed-phase columns	2004	807	235-241	J Chromatogr B Analyt Technol Biomed Life Sci
Dawson, L. A.; Stow, J. M.; Dourish, C. T.; Routledge, C.	Analysis of glutamate in striatal microdialysates using capillary electrophoresis and laser-induced fluorescence detection	1995	700	81-87	Journal of Chromatography A
Dawson, L. A.; Stow, J. M.; Palmer, A. M.	Improved method for the measurement of glutamate and aspartate using capillary electrophoresis with laser induced fluorescence detection and its application to brain microdialysis	1997	694	455-460	J Chromatogr B Biomed Sci Appl
De Bundel, D.; Schallier, A.; Loyens, E.; Fernando, R.; Miyashita, H.; Van Liefferinge, J.; Vermoesen, K.; Bannai, S.; Sato, H.; Michotte, Y.; Smolders, I.; Massie, A.	Loss of system x(c)- does not induce oxidative stress but decreases extracellular glutamate in hippocampus and influences spatial working memory and limbic seizure susceptibility	2011	31	5792-5803	J Neurosci
de Knegt, R. J.; Schalm, S. W.; van der Rijt, C. C.; Fekkes, D.; Dalm, E.; Hekking-Weyma, I.	Extracellular brain glutamate during acute liver failure and during acute hyperammonemia simulating acute liver failure: an experimental study based on in vivo brain dialysis	1994	20	19-26	J Hepatol
De Novellis, V.; Marabese, I.; Palazzo, E.; Berrino, L.; Rodella, L.; Bianchi, R.; Rossi, F.; Maione, S.	Group I metabotropic glutamate receptors modulate glutamate and gamma-aminobutyric acid release in the periaqueductal grey of rats	2003	462	73-81	Eur J Pharmacol
de Novellis, V.; Marabese, I.; Uliano, R.; Palazzo, E.; Scafuro, A.; sca Rossi, F.; Maione, S.	Type I and II metabotropic glutamate receptors modulate periaqueductal grey glycine release: interaction between mGlu2/3 and A1 adenosine receptors	2002	43	1061-1069	Neuropharmacology
de Novellis, V.; Negri, L.; Lattanzi, R.; Rossi, F.; Palazzo, E.; Marabese, I.; Giannini, E.; Vita, D.; Melchiorri, P.; Maione, S.	The prokineticin receptor agonist Bv8 increases GABA release in the periaqueductal grey and modifies RVM cell activities and thermoceptive reflexes in the rat	2007	26	3068-3078	Eur J Neurosci
De Witte, P.; Dahchour, A.; Quertemont, E.	Acute and chronic alcohol injections increase taurine in the nucleus accumbens	1994	2	229-233	Alcohol Alcohol Suppl
Del Arco, A.; Castaneda, T. R.; Mora, F.	Amphetamine releases GABA in striatum of the freely moving rat:	1998	37	199-205	Neuropharmacology

Glutamate					
Authors	Title	Year	Vol.	Pages	Journal
	involvement of calcium and high affinity transporter mechanisms				
Del Arco, A.; Gonzalez-Mora, J. L.; Armas, V. R.; Mora, F.	Amphetamine increases the extracellular concentration of glutamate in striatum of the awake rat: involvement of high affinity transporter mechanisms	1999	38	943-954	Neuropharmacology
Del Arco, A.; Martinez, R.; Mora, F.	Amphetamine increases extracellular concentrations of glutamate in the prefrontal cortex of the awake rat: a microdialysis study	1998	23	1153-1158	Neurochem Res
Del Arco, A.; Mora, F.	NMDA and AMPA/kainate glutamatergic agonists increase the extracellular concentrations of GABA in the prefrontal cortex of the freely moving rat: modulation by endogenous dopamine	2002	57	623-630	Brain Res Bull
Del Arco, A.; Mora, F.	Endogenous dopamine potentiates the effects of glutamate on extracellular GABA in the prefrontal cortex of the freely moving rat	2000	53	339-345	Brain Res Bull
Del Arco, A.; Mora, F.	Effects of endogenous glutamate on extracellular concentrations of GABA, dopamine, and dopamine metabolites in the prefrontal cortex of the freely moving rat: involvement of NMDA and AMPA/KA receptors	1999	24	1027-1035	Neurochem Res
Del Arco, A.; Segovia, G.; Mora, F.	Effects of endogenous glutamate on extracellular concentrations of taurine in striatum and nucleus accumbens of the awake rat: involvement of NMDA and AMPA/kainate receptors	2000	19	729-738	Amino Acids
Del Arco, A.; Segovia, G.; Prieto, L.; Mora, F.	Endogenous glutamate-taurine interaction in striatum and nucleus accumbens of the freely moving rat: studies during the normal process of aging	2001	122	401-414	Mech Ageing Dev
Delgado, J. M.; DeFeudis, F. V.; Roth, R. H.; Ryugo, D. K.; Mitruka, B. M.	Dialytrode for long term intracerebral perfusion in awake monkeys	1972	198	9-21	Arch Int Pharmacodyn Ther
Delgado, J. M.; Lerma, J.; Martin del Rio, R.; Solis, J. M.	Dialytrode technology and local profiles of amino acids in the awake cat brain	1984	42	1218-1228	J Neurochem
Dell, L. E.; Parsons, L. H.	Serotonin1B receptors in the ventral tegmental area modulate cocaine-induced increases in nucleus accumbens dopamine levels	2004	311	711-719	J Pharmacol Exp Ther
Deng, Y.; Wang, M.; Jiang, L.; Ma, C.; Xi, Z.; Li, X.; He, N.	A comparison of extracellular excitatory amino acids release inhibition of acute lamotrigine and topiramate treatment in the hippocampus of PTZ-kindled epileptic rats	2013	9	1123-1128	J Biomed Nanotechnol
Deng, Y.; Wang, M.; Wang, W.; Ma, C.;	Comparison and effects of acute lamotrigine treatment on	2013	38	504-511	Neurochem Res

Glutamate					
**Authors**	**Title**	**Year**	**Vol.**	**Pages**	**Journal**
He, N.	extracellular excitatory amino acids in the hippocampus of PTZ-kindled epileptic and PTZ-induced status epilepticus rats				
Deransart, C.; Riban, V.; Le, B. T.; Hechler, V.; Marescaux, C.; Depaulis, A.	Evidence for the involvement of the pallidum in the modulation of seizures in a genetic model of absence epilepsy in the rat	1999	265	131-134	Neurosci Lett
Devall, A. J.; Blake, R.; Langman, N.; Smith, C. G.; Richards, D. A.; Whitehead, K. J.	Monolithic column-based reversed-phase liquid chromatography separation for amino acid assay in microdialysates and cerebral spinal fluid	2007	848	323-328	J Chromatogr B Analyt Technol Biomed Life Sci
Di Cara, B.; Dusticier, N.; Forni, C.; Lievens, J. C.; Daszuta, A.	Serotonin depletion produces long lasting increase in striatal glutamatergic transmission	2001	78	240-248	J Neurochem
Di, X.; Harpold, T.; Watson, J. C.; Bullock, M. R.	Excitotoxic damage in neurotrauma: fact or fiction	1996	9	231-241	Restor Neurol Neurosci
Dietze, S.; Kuschinsky, K.	Determination of extracellular glutamate after local K+ stimulation in the striatum of non-anaesthetised rats after treatment with dopaminergic drugs--studies using microdialysis	1992	90	1-11	J Neural Transm Gen Sect
Dijk, S. N.; Francis, P. T.; Stratmann, G. C.; Bowen, D. M.	NMDA-induced glutamate and aspartate release from rat cortical pyramidal neurones: evidence for modulation by a 5-HT1A antagonist	1995	115	1169-1174	Br J Pharmacol
Dijk, S. N.; Francis, P. T.; Stratmann, G. C.; Bowen, D. M.	Cholinomimetics increase glutamate outflow via an action on the corticostriatal pathway: implications for Alzheimer's disease	1995	65	2165-2169	J Neurochem
Dijk, S. N.; Krop-van Gastel, W.; Obrenovitch, T. P.; Korf, J.	Food deprivation protects the rat striatum against hypoxia-ischemia despite high extracellular glutamate	1994	62	1847-1851	J Neurochem
Ding, R.; Asada, H.; Obata, K.	Changes in extracellular glutamate and GABA levels in the hippocampal CA3 and CA1 areas and the induction of glutamic acid decarboxylase-67 in dentate granule cells of rats treated with kainic acid	1998	800	105-113	Brain Res
Ding, Z. M.; Engleman, E. A.; Rodd, Z. A.; McBride, W. J.	Ethanol increases glutamate neurotransmission in the posterior ventral tegmental area of female wistar rats	2012	36	633-640	Alcohol Clin Exp Res
Ding, Z. M.; Ingraham, C. M.; Lasek, A. W.; Hauser, S. R.; Bell, R. L.; McBride, W. J.	Differences in cortico-limbic MGLUR2 levels of alcohol-preferring (P) and wistar rats: Dissociation with glutamate transmission and alcohol drinking	2016	40	28A	Alcohol Clin Exp Res
Ding, Z. M.; Ingraham, C. M.; Rodd, Z. A.; McBride, W. J.	Alcohol drinking increases the dopamine-stimulating effects of ethanol and reduces D2 auto-receptor and group II metabotropic glutamate receptor function within the posterior ventral tegmental	2016	109	41-48	Neuropharmacology

Glutamate					
**Authors**	**Title**	**Year**	**Vol.**	**Pages**	**Journal**
	area of alcohol preferring (P) rats				
Ding, Z. M.; Rodd, Z. A.; Engleman, E. A.; Bailey, J. A.; Lahiri, D. K.; McBride, W. J.	Alcohol drinking and deprivation alter basal extracellular glutamate concentrations and clearance in the mesolimbic system of alcohol-preferring (P) rats	2013	18	297-306	Addict Biol
Ding, Z. M.; Rodd, Z. A.; Engleman, E. A.; Bailey, J. A.; Lahiri, D. K.; McBride, W. J.	Chronic alcohol drinking reduces glutamate clearance and enhances basal extracellular glutamate levels within the mesolimbic system	2012	36	95A	Alcohol Clin Exp Res
Doane, D. F.; Lawson, M. A.; Meade, J. R.; Kotz, C. M.; Beverly, J. L.	Orexin-induced feeding requires NMDA receptor activation in the perifornical region of the lateral hypothalamus	2007	293	R1022-R1026	Am J Physiol Regul Integr Comp Physiol
Doboszewska, U.; Szewczyk, B.; Sowa-Kucma, M.; Noworyta-Sokolowska, K.; Misztak, P.; Golebiowska, J.; Mlyniec, K.; Ostachowicz, B.; Krosniak, M.; Wojtanowska-Krosniak, A.; Golembiowska, K.; Lankosz, M.; Piekoszewski, W.; Nowak, G.	Alterations of Bio-elements, Oxidative, and Inflammatory Status in the Zinc Deficiency Model in Rats	2016	29	143-154	Neurotox Res
Dohmen, C.; Kumura, E.; Rosner, G.; Heiss, W. D.; Graf, R.	Adenosine in relation to calcium homeostasis: comparison between gray and white matter ischemia	2001	21	503-510	J Cereb Blood Flow Metab
Dohmen, C.; Kumura, E.; Rosner, G.; Heiss, W. D.; Graf, R.	Extracellular correlates of glutamate toxicity in short-term cerebral ischemia and reperfusion: a direct in vivo comparison between white and gray matter	2005	1037	43-51	Brain Res
Domin, H.; Golembiowska, K.; Jantas, D.; Kaminska, K.; Zieba, B.; Smialowska, M.	Group III mGlu receptor agonist, ACPT-I, exerts potential neuroprotective effects in vitro and in vivo	2014	26	99-113	Neurotox Res
Domin, H.; Zieba, B.; Golembiowska, K.; Kowalska, M.; Dziubina, A.; Smialowska, M.	Neuroprotective potential of mGluR5 antagonist MTEP: effects on kainate-induced excitotoxicity in the rat hippocampus	2010	62	1051-1061	Pharmacol Rep
Dominguez, J. M.; Gil, M.; Hull, E. M.	Preoptic glutamate facilitates male sexual behavior	2006	26	1699-1703	J Neurosci
Dong, H. I.; Fukuda, S.; Murata, E.; Higuchi, T.	Excitatory and inhibitory actions of isoflurane on the cholinergic ascending arousal system of the rat	2006	104	122-132	Anesthesiology
Donnell, J. C.; McDonough, J. H.; Shih, T. M.	In vivo microdialysis and electroencephalographic activity in freely moving guinea pigs exposed to organophosphorus nerve agents sarin and VX: analysis of acetylcholine and glutamate	2011	85	1607-1616	Arch Toxicol

Glutamate					
**Authors**	**Title**	**Year**	**Vol.**	**Pages**	**Journal**
Donzanti, B. A.; Hite, J. F.; Yamamoto, B. K.	Extracellular glutamate levels increase with age in the lateral striatum: potential involvement of presynaptic D-2 receptors	1993	13	376-382	Synapse
Donzanti, B. A.; Yamamoto, B. K.	An improved and rapid HPLC-EC method for the isocratic separation of amino acid neurotransmitters from brain tissue and microdialysis perfusates	1988	43	913-922	Life Sci
Doretto, M. C.; Burger, R. L.; Mishra, P. K.; Garcia-Cairasco, N.; Dailey, J. W.; Jobe, P. C.	A microdialysis study of amino acid concentrations in the extracellular fluid of the substantia nigra of freely behaving GEPR-9s: relationship to seizure predisposition	1994	17	157-165	Epilepsy Res
Douen, A. G.; Akiyama, K.; Hogan, M. J.; Wang, F.; Dong, L.; Chow, A. K.; Hakim, A.	Preconditioning with cortical spreading depression decreases intraischemic cerebral glutamate levels and down-regulates excitatory amino acid transporters EAAT1 and EAAT2 from rat cerebal cortex plasma membranes	2000	75	812-818	J Neurochem
Drejer, J.; Benveniste, H.; Diemer, N. H.; Schousboe, A.	Cellular origin of ischemia-induced glutamate release from brain tissue in vivo and in vitro	1985	45	145-151	J Neurochem
Dupre, K. B.; Ostock, C. Y.; Eskow Jaunarajs, K. L.; Button, T.; Savage, L. M.; Wolf, W.; Bishop, C.	Local modulation of striatal glutamate efflux by serotonin 1A receptor stimulation in dyskinetic, hemiparkinsonian rats	2011	229	288-299	Exp Neurol
During, M. J.; Craig, J. S.; Hernandez, T. D.; Anderson, G. M.; Gallager, D. W.	Effect of amygdala kindling on the in vivo release of GABA and 5-HT in the dorsal raphe nucleus in freely moving rats	1992	584	36-44	Brain Res
Durmuller, N.; Graham, J. L.; Sowinski, P.; Meldrum, B. S.	The vital dye Evans blue mimics limbic seizures induced by kainate or pilocarpine	1997	753	283-290	Brain Res
Duszczyk-Budhathoki, M.; Olczak, M.; Lehner, M.; Majewska, M. D.	Administration of thimerosal to infant rats increases overflow of glutamate and aspartate in the prefrontal cortex: protective role of dehydroepiandrosterone sulfate	2012	37	436-447	Neurochem Res
Dzahini, K.; Dentresangle, C.; Le Cavorsin, M.; Bertrand, A.; Detraz, I.; Savasta, M.; Leviel, V.	Pre-synaptic glutamate-induced activation of DA release in the striatum after partial nigral lesion	2010	113	1459-1470	J Neurochem
Ebner, K.; Bosch, O. J.; Kromer, S. A.; Singewald, N.; Neumann, I. D.	Release of oxytocin in the rat central amygdala modulates stress-coping behavior and the release of excitatory amino acids	2005	30	223-230	Neuropsychopharmacology
Ehlen, J. C.; Albers, H. E.; Breyer, E. D.	MEKC-LIF of gamma-amino butyric acid in microdialysate: Systematic optimization of the separation conditions by factorial analysis	2005	147	36-47	J Neurosci Methods
El Arfani, A.; Bentea, E.; Aourz, N.; Ampe,	NMDA receptor antagonism potentiates the L-DOPA-induced	2014	85	198-205	Neuropharmacology

Glutamate					
**Authors**	**Title**	**Year**	**Vol.**	**Pages**	**Journal**
B.; De Deurwaerdere, P.; Van Eeckhaut, A.; Massie, A.; Sarre, S.; Smolders, I.; Michotte, Y.	extracellular dopamine release in the subthalamic nucleus of hemi-parkinson rats				
el-Yamany, N. A.; Horn, E.	Time courses of aspartate and glutamate concentrations in the focus area during penicillin induced epileptiform activity in awake rats	2002	140	13-30	Arch Ital Biol
Enblad, P.; Frykholm, P.; Valtysson, J.; Silander, H. C.; Andersson, J.; Fasth, K. J.; Watanabe, Y.; Langstrom, B.; Hillered, L.; Persson, L.	Middle cerebral artery occlusion and reperfusion in primates monitored by microdialysis and sequential positron emission tomography	2001	32	1574-1580	Stroke
Engel, M.; Snikeris, P.; Jenner, A.; Karl, T.; Huang, X. F.; Frank, E.	Neuregulin 1 prevents phencyclidine-induced behavioral impairments and disruptions to GABAergic signaling in mice	2015	18	1-11	Int J Neuropsychopharmacol
Engelhard, K.; Werner, C.; Hoffman, W. E.; Matthes, B.; Blobner, M.; Kochs, E.	The effect of sevoflurane and propofol on cerebral neurotransmitter concentrations during cerebral ischemia in rats	2003	97	1155-1561	Anesth Analg
Engelmann, M.; Bull, P. M.; Brown, C. H.; Landgraf, R.; Horn, T. F.; Singewald, N.; Ludwig, M.; Wotjak, C. T.	GABA selectively controls the secretory activity of oxytocin neurons in the rat supraoptic nucleus	2004	19	601-608	Eur J Neurosci
Engelmann, M.; Ludwig, M.; Singewald, N.; Ebner, K.; Sabatier, N.; Lubec, G.; Landgraf, R.; Wotjak, C. T.	Taurine selectively modulates the secretory activity of vasopressin neurons in conscious rats	2001	14	1047-1055	Eur J Neurosci
Engelmann, M.; Wolf, G.; Horn, T. F.	Release patterns of excitatory and inhibitory amino acids within the hypothalamic supraoptic nucleus in response to direct nitric oxide administration during forced swimming in rats	2002	324	252-254	Neurosci Lett
Enrico, P.; Mura, M. A.; Esposito, G.; Serra, P.; Migheli, R.; De Natale, G.; Desole, M. S.; Miele, M.; Miele, E.	Effect of naloxone on morphine-induced changes in striatal dopamine metabolism and glutamate, ascorbic acid and uric acid release in freely moving rats	1998	797	94-102	Brain Res
Escartin, C.; Brouillet, E.; Gubellini, P.; Trioulier, Y.; Jacquard, C.; Smadja, C.; Knott, G. W.; Goff, L. K. L.; Deglon, N.; Hantraye, P.; Bonvento, G.	Ciliary neurotrophic factor activates astrocytes, redistributes their glutamate transporters GLAST and GLT-1 to raft microdomains, and improves glutamate handling in vivo	2006	26	5978-5989	J Neurosci
Escobar, A. P.; Cornejo, F. A.; Olivares-Costa, M.; Gonzalez, M.; Fuentealba, J. A.; Gysling, K.; Espana, R. A.; Andres, M.	Reduced dopamine and glutamate neurotransmission in the nucleus accumbens of quinpirole-sensitized rats hints at inhibitory D2 autoreceptor function	2015	134	1081-1090	J Neurochem

Glutamate					
**Authors**	**Title**	**Year**	**Vol.**	**Pages**	**Journal**
E.					
Espey, M. G.; Kustova, Y.; Sei, Y.; Basile, A. S.	Extracellular glutamate levels are chronically elevated in the brains of LP-BM5-infected mice: a mechanism of retrovirus-induced encephalopathy	1998	71	2079-2087	J Neurochem
Exposito, I.; Del Arco, A.; Segovia, G.; Mora, F.	Endogenous dopamine increases extracellular concentrations of glutamate and GABA in striatum of the freely moving rat: involvement of D1 and D2 dopamine receptors	1999	24	849-856	Neurochem Res
Fabre-Nys, C.; Blache, D.; Hinton, M. R.; Goode, J. A.; Kendrick, K. M.	Microdialysis measurement of neurochemical changes in the mediobasal hypothalamus of ovariectomized ewes during oestrus	1994	649	282-296	Brain Res
Fabre-Nys, C.; Ohkura, S.; Kendrick, K. M.	Male faces and odours evoke differential patterns of neurochemical release in the mediobasal hypothalamus of the ewe during oestrus: an insight into sexual motivation?	1997	9	1666-1677	Eur J Neurosci
Fabricius, M.; Jensen, L. H.; Lauritzen, M.	Microdialysis of interstitial amino acids during spreading depression and anoxic depolarization in rat neocortex	1993	612	61-69	Brain Res
Fadel, J.; Frederick-Duus, D.	Orexin/hypocretin modulation of the basal forebrain cholinergic system: insights from in vivo microdialysis studies	2008	90	156-162	Pharmacol Biochem Behav
Faden, A. I.	Dynorphin increases extracellular levels of excitatory amino acids in the brain through a non-opioid mechanism	1992	12	425-429	J Neurosci
Faiman, M. D.; Kaul, S.; Latif, S. A.; Williams, T. D.; Lunte, C. E.	S-(N, N-diethylcarbamoyl)glutathione (carbamathione), a disulfiram metabolite and its effect on nucleus accumbens and prefrontal cortex dopamine, GABA, and glutamate: a microdialysis study	2013	75	95-105	Neuropharmacology
Falkenberg, T.; Lindefors, N.; Camilli, F.; Metsis, M.; Ungerstedt, U.	Glutamate release correlates with brain-derived neurotrophic factor and trkB mRNA expression in the CA1 region of rat hippocampus	1996	42	317-327	Brain Res Mol Brain Res
Fallgren, A. B.; Paulsen, R. E.	A microdialysis study in rat brain of dihydrokainate, a glutamate uptake inhibitor	1996	21	19-25	Neurochem Res
Fang, Y. R.; Abekawa, T.; Li, X. B.; Wang, Z. C.; Inoue, T.; Koyama, T.	Effect of the protein kinase C inhibitor, staurosporine, on the high dose of methamphetamine-induced behavioral sensitization to dizocilpine (MK-801)	2005	180	100-106	Psychopharmacology(Berl)
Fantin, M.; Auberson, Y. P.; Morari, M.	Differential effect of NR2A and NR2B subunit selective NMDA receptor antagonists on striato-pallidal neurons: relationship to motor response in the 6-hydroxydopamine model of parkinsonism	2008	106	957-968	J Neurochem

Glutamate					
**Authors**	**Title**	**Year**	**Vol.**	**Pages**	**Journal**
Fantin, M.; Marti, M.; Auberson, Y. P.; Morari, M.	NR2A and NR2B subunit containing NMDA receptors differentially regulate striatal output pathways	2007	103	2200-2211	J Neurochem
Farahmandfar, M.; Karimian, S. M.; Zarrindast, M. R.; Kadivar, M.; Afrouzi, H.; Naghdi, N.	Morphine sensitization increases the extracellular level of glutamate in CA1 of rat hippocampus via mu-opioid receptor	2011	494	130-134	Neurosci Lett
Fatar, M.; Stroick, M.; Griebe, M.; Alonso, A.; Kreisel, S.; Kern, R.; Hennerici, M.; Meairs, S.	Effect of combined ultrasound and microbubbles treatment in an experimental model of cerebral ischemia	2008	34	1414-1420	Ultrasound Med Biol
Fattorini, G.; Bragina, L.; Candiracci, C.; Melone, M.; Cozzi, A.; Pellegrini-Giampietro, D. E.; Conti, F.	Acute phencyclidine administration reduces extracellular glutamate levels and the expression of synaptophysin and SNAP-25 in rat frontal cortex	2009	108	288-289	Schizophr Bull
Fattorini, G.; Melone, M.; Bragina, L.; Candiracci, C.; Cozzi, A.; Giampietro, D. E. P.; Torres-Ramos, M.; Perez-Samartin, A.; Matute, C.; Conti, F.	GLT-1 expression and Glu uptake in rat cerebral cortex are increased by phencyclidine	2008	56	1320-1327	Glia
Favalli, L.; Rozza, A.; Frattini, P.; Masoero, E.; Scelsi, R.; Pascale, A.; Govoni, S.	Ischemia-induced glutamate release in rat frontoparietal cortex after chronic alcohol and withdrawal	2002	326	183-186	Neurosci Lett
Fedele, E.; Foster, A. C.	An evaluation of the role of extracellular amino acids in the delayed neurodegeneration induced by quinolinic acid in the rat striatum	1993	52	911-917	Neuroscience
Fedele, E.; Varnier, G.; Ansaldo, M. A.; Raiteri, M.	Nicotine administration stimulates the in vivo N-methyl-D-aspartate receptor/nitric oxide/cyclic GMP pathway in rat hippocampus through glutamate release	1998	125	1042-1048	Br J Pharmacol
Feet, B. A.; Gilland, E.; Groenendaal, F.; Brun, N. C.; Hellstrom-Westas, L.; Hagberg, H.; Saugstad, O. D.	Cerebral excitatory amino acids and Na+/K+ -ATPase activity during resuscitation of severely hypoxic newborn piglets	1998	87	889-895	Int J Pediatr
Feng, Y. Z.; Zhang, T.; Rockhold, R. W.; Ho, I. K.	Increased locus coeruleus glutamate levels are associated with naloxone-precipitated withdrawal from butorphanol in the rat	1995	20	745-751	Neurochem Res
Feng, Y. Z.; Zhang, T.; Tokuyama, S.; Zhu, H.; Rockhold, R. W.; Ho, I. K.	Mu- and delta-opioid receptor antagonists precipitate similar withdrawal phenomena in butorphanol and morphine dependence	1996	21	63-71	Neurochem Res
Feng, Y.; LeBlanc, M. H.; Regunathan, S.	Agmatine reduces extracellular glutamate during	2005	390	129-133	Neurosci Lett

Glutamate					
**Authors**	**Title**	**Year**	**Vol.**	**Pages**	**Journal**
	pentylenetetrazole-induced seizures in rat brain: a potential mechanism for the anticonvulsive effects				
Feng, Y.; Rockhold, R. W.; Ho, I. K.	Nor-binaltorphimine precipitates withdrawal and excitatory amino acid release in the locus ceruleus of butorphanol--but not morphine-dependent rats	1997	283	932-938	J Pharmacol Exp Ther
Ferger, B.; Boonen, G.; Haberlein, H.; Kuschinsky, K.	In vivo microdialysis study of (+/-)-kavain on veratridine-induced glutamate release	1998	347	211-214	Eur J Pharmacol
Ferguson, M. C.; Nayyar, T.; Ansah, T. A.	Reverse microdialysis of a 5-HT2A receptor antagonist alters extracellular glutamate levels in the striatum of the MPTP mouse model of Parkinson's disease	2014	71	36-46	Neurochem Int
Ferrario, C. R.; Shou, M.; Samaha, A. N.; Watson, C. J.; Kennedy, R. T.; Robinson, T. E.	The rate of intravenous cocaine administration alters c-fos mRNA expression and the temporal dynamics of dopamine, but not glutamate, overflow in the striatum	2008	1209	151-156	Brain Res
Ferraro, L.; Antonelli, T.; Connor, W. T.; Fuxe, K.; Soubrie, P.; Tanganelli, S.	The striatal neurotensin receptor modulates striatal and pallidal glutamate and GABA release: functional evidence for a pallidal glutamate-GABA interaction via the pallidal-subthalamic nucleus loop	1998	18	6977-6989	J Neurosci
Ferraro, L.; Antonelli, T.; Connor, W. T.; Tanganelli, S.; Rambert, F. A.; Fuxe, K.	The effects of modafinil on striatal, pallidal and nigral GABA and glutamate release in the conscious rat: evidence for a preferential inhibition of striato-pallidal GABA transmission	1998	253	135-138	Neurosci Lett
Ferraro, L.; Antonelli, T.; Connor, W. T.; Tanganelli, S.; Rambert, F.; Fuxe, K.	The antinarcoleptic drug modafinil increases glutamate release in thalamic areas and hippocampus	1997	8	2883-2887	Neuroreport
Ferraro, L.; Antonelli, T.; Tanganelli, S.; Connor, W. T.; Perez de la Mora, M.; Mendez-Franco, J.; Rambert, F. A.; Fuxe, K.	The vigilance promoting drug modafinil increases extracellular glutamate levels in the medial preoptic area and the posterior hypothalamus of the conscious rat: prevention by local GABAA receptor blockade	1999	20	346-356	Neuropsychopharmacology
Ferraro, L.; Beggiato, S.; Tomasini, M. C.; Antonelli, T.; Loche, A.; Tanganelli, S.	GET73 modulates rat hippocampal glutamate transmission: evidence for a functional interaction with mGluR5	2011	63	1359-1371	Pharmacol Rep
Ferraro, L.; Beggiato, S.; Tomasini, M. C.; Fuxe, K.; Tanganelli, S.; Antonelli, T.	Neurotensin regulates cortical glutamate transmission by modulating N-methyl-D-aspartate receptor functional activity: an in vivo microdialysis study	2011	89	1618-1626	J Neurosci Res
Ferraro, L.; Connor, W. T.; Beggiato, S.; Tomasini, M. C.; Fuxe, K.; Tanganelli, S.;	Striatal NTS1 , dopamine D2 and NMDA receptor regulation of pallidal GABA and glutamate release--a dual-probe microdialysis	2012	35	207-220	Eur J Neurosci

Glutamate					
**Authors**	**Title**	**Year**	**Vol.**	**Pages**	**Journal**
Antonelli, T.	study in the intranigral 6-hydroxydopamine unilaterally lesioned rat				
Ferraro, L.; Frankowska, M.; Marcellino, D.; Zaniewska, M.; Beggiato, S.; Filip, M.; Tomasini, M. C.; Antonelli, T.; Tanganelli, S.; Fuxe, K.	A novel mechanism of cocaine to enhance dopamine d2-like receptor mediated neurochemical and behavioral effects. An in vivo and in vitro study	2012	37	1856-1866	Neuropsychopharmacology
Ferraro, L.; Tanganelli, S.; Connor, W. T.; Bianchi, C.; Ungerstedt, U.; Fuxe, K.	Neurotensin increases endogenous glutamate release in the neostriatum of the awake rat	1995	20	362-364	Synapse
Ferraro, L.; Tanganelli, S.; Connor, W. T.; Francesconi, W.; Loche, A.; Gessa, G. L.; Antonelli, T.	gamma-Hydroxybutyrate modulation of glutamate levels in the hippocampus: an in vivo and in vitro study	2001	78	929-939	J Neurochem
Ferraro, L.; Tomasini, M. C.; Fernandez, M.; Bebe, B. W.; Connor, W. T.; Fuxe, K.; Glennon, J. C.; Tanganelli, S.; Antonelli, T.	Nigral neurotensin receptor regulation of nigral glutamate and nigroventral thalamic GABA transmission: a dual-probe microdialysis study in intact conscious rat brain	2001	102	113-120	Neuroscience
Ferraro, L.; Tomasini, M. C.; Gessa, G. L.; Bebe, B. W.; Tanganelli, S.; Antonelli, T.	The cannabinoid receptor agonist WIN 55,212-2 regulates glutamate transmission in rat cerebral cortex: an in vivo and in vitro study	2001	11	728-733	Cereb Cortex
Ferraro, L.; Tomasini, M. C.; Mazza, R.; Fuxe, K.; Fournier, J.; Tanganelli, S.; Antonelli, T.	Neurotensin receptors as modulators of glutamatergic transmission	2008	58	365-373	Brain Res Brain Res Rev
Feustel, P. J.; Jin, Y.; Kimelberg, H. K.	Volume-regulated anion channels are the predominant contributors to release of excitatory amino acids in the ischemic cortical penumbra	2004	35	1164-1168	Stroke
Folkersma, H.; Foster Dingley, J. C.; van Berckel, B. N.; Rozemuller, A.; Boellaard, R.; Huisman, M. C.; Lammertsma, A. A.; Vandertop, W. P.; Molthoff, C. F.	Increased cerebral (R)-[(11)C]PK11195 uptake and glutamate release in a rat model of traumatic brain injury: a longitudinal pilot study	2011	8	67	J Neuroinflammation
Forray, M. I.; Bustos, G.; Gysling, K.	Noradrenaline inhibits glutamate release in the rat bed nucleus of the stria terminalis: in vivo microdialysis studies	1999	55	311-320	J Neurosci Res
Foster, S. B.; Tang, H.; Miller, K. E.; Dryhurst, G.	Increased extracellular glutamate evoked by 1-methyl-4-phenylpyridinium [MPP(+)] in the rat striatum is not essential for dopaminergic neurotoxicity and is not derived from released	2005	7	251-263	Neurotox Res

Glutamate					
**Authors**	**Title**	**Year**	**Vol.**	**Pages**	**Journal**
	glutathione				
Franke, H.; Grummich, B.; Hartig, W.; Grosche, J.; Regenthal, R.; Edwards, R. H.; Illes, P.; Krugel, U.	Changes in purinergic signaling after cerebral injury -- involvement of glutamatergic mechanisms?	2006	24	123-132	Int J Dev Neurosci
Frantz, K.; Harte, M.; Ungerstedt, U.; Connor, W. T.	A dual probe characterization of dialysate amino acid levels in the medial prefrontal cortex and ventral tegmental area of the awake freely moving rat	2002	119	109-119	J Neurosci Methods
Fraser, M.; Bennet, L.; Van Zijl, P. L.; Mocatta, T. J.; Williams, C. E.; Gluckman, P. D.; Winterbourn, C. C.; Gunn, A. J.	Extracellular amino acids and lipid peroxidation products in periventricular white matter during and after cerebral ischemia in preterm fetal sheep	2008	105	2214-2223	J Neurochem
Frederickson, C. J.; Giblin, L. J.; Krezel, A.; McAdoo, D. J.; Muelle, R. N.; Zeng, Y.; Balaji, R. V.; Masalha, R.; Thompson, R. B.; Fierke, C. A.; Sarvey, J. M.; de Valdenebro, M.; Prough, D. S.; Zornow, M. H.	Concentrations of extracellular free zinc (pZn) e in the central nervous system during simple anesthetization, ischemia and reperfusion	2006	198	285-293	Exp Neurol
Freinbichler, W.; Colivicchi, M. A.; Fattori, M.; Ballini, C.; Tipton, K. F.; Linert, W.; Della Corte, L.	Validation of a robust and sensitive method for detecting hydroxyl radical formation together with evoked neurotransmitter release in brain microdialysis	2008	105	738-749	J Neurochem
Froen, J. F.; Munkeby, B. H.; Stray-Pedersen, B.; Saugstad, O. D.	Interleukin-10 reverses acute detrimental effects of endotoxin-induced inflammation on perinatal cerebral hypoxia-ischemia	2002	942	87-94	Brain Res
Frykholm, P.; Hillered, L.; Langstrom, B.; Persson, L.; Valtysson, J.; Watanabe, Y.; Enblad, P.	Increase of interstitial glycerol reflects the degree of ischaemic brain damage: a PET and microdialysis study in a middle cerebral artery occlusion-reperfusion primate model	2001	71	455-461	J Neurol Neurosurg Psychiatry
Fu, L. W.; Longhurst, J. C.	Electroacupuncture modulates vlPAG release of GABA through presynaptic cannabinoid CB1 receptors	2009	106	1800-1809	J Appl Physiol (1985)
Fu, Y.; He, F.; Zhang, S.; Huang, J.; Zhang, J.; Jiao, X.	3-Nitropropionic acid produces indirect excitotoxic damage to rat striatum	1995	17	333-339	Neurotoxicol Teratol
Fu, Y.; Matta, S. G.; Gao, W.; Brower, V. G.; Sharp, B. M.	Systemic nicotine stimulates dopamine release in nucleus accumbens: re-evaluation of the role of N-methyl-D-aspartate receptors in the ventral tegmental area	2000	294	458-465	J Pharmacol Exp Ther
Fuentealba, J. A.; Forray, M. I.; Gysling, K.	Chronic morphine treatment and withdrawal increase extracellular levels of norepinephrine in the rat bed nucleus of the	2000	75	741-748	J Neurochem

Glutamate					
**Authors**	**Title**	**Year**	**Vol.**	**Pages**	**Journal**
	stria terminalis				
Fujikawa, D. G.	Effects of N-methyl-D-aspartate-receptor blockade on high-potassium-induced neuronal death and glutamate release	1997	226	25-28	Neurosci Lett
Fujikawa, D. G.; Kim, J. S.; Daniels, A. H.; Alcaraz, A. F.; Sohn, T. B.	In vivo elevation of extracellular potassium in the rat amygdala increases extracellular glutamate and aspartate and damages neurons	1996	74	695-706	Neuroscience
Fujisawa, H.; Koizumi, H.; Ito, H.; Yamashita, K.; Maekawa, T.	Effects of mild hypothermia on the cortical release of excitatory amino acids and nitric oxide synthesis following hypoxia	1999	16	1083-1093	J Neurotrauma
Fujisawa, H.; Sugimura, Y.; Takagi, H.; Mizoguchi, H.; Takeuchi, H.; Izumida, H.; Nakashima, K.; Ochiai, H.; Takeuchi, S.; Kiyota, A.; Fukumoto, K.; Iwama, S.; Takagishi, Y.; Hayashi, Y.; Arima, H.; Komatsu, Y.; Murata, Y.; Oiso, Y.	Chronic Hyponatremia Causes Neurologic and Psychologic Impairments	2016	27	766-780	J Am Soc Nephrol
Fujita, T.; Kamisaki, Y.; Yonehara, N.	Nitric oxide-induced increase of excitatory amino acid levels in the trigeminal nucleus caudalis of the rat with tactile hypersensitivity evoked by the loose-ligation of the inferior alveolar nerves	2004	91	558-567	J Neurochem
Fujitani, T.; Adachi, N.; Miyazaki, H.; Liu, K.; Nakamura, Y.; Kataoka, K.; Arai, T.	Lidocaine protects hippocampal neurons against ischemic damage by preventing increase of extracellular excitatory amino acids: a microdialysis study in Mongolian gerbils	1994	179	91-94	Neurosci Lett
Fukuyama, K.; Tanahashi, S.; Nakagawa, M.; Yamamura, S.; Motomura, E.; Shiroyama, T.; Tanii, H.; Okada, M.	Levetiracetam inhibits neurotransmitter release associated with CICR	2012	518	69-74	Neurosci Lett
Furukawa, N.; Arai, N.; Goshima, Y.; Miyamae, T.; Ohshima, E.; Suzuki, F.; Fujita, K.; Misu, Y.	Endogenously released DOPA is a causal factor for glutamate release and resultant delayed neuronal cell death by transient ischemia in rat striata	2001	76	815-824	J Neurochem
Furuse, M.; Ohta, T.; Ikenaga, T.; Liang, Y. M.; Isono, N.; Kuroiwa, T.; Preul, M. C.	Effects of intravascular perfusion of cooled crystalloid solution on cold-induced brain injury using an extracorporeal cooling-filtration system	2003	145	983-993	Acta Neurochir (Wien)
Gabriel, E. M.; Inglefield, J. R.; Chadwick, L. E.; Schwartz-Bloom, R. D.	Ischemic injury and extracellular amino acid accumulation in hippocampal area CA1 are not dependent upon an intact septo-hippocampal pathway	1998	785	279-286	Brain Res

Glutamate					
**Authors**	**Title**	**Year**	**Vol.**	**Pages**	**Journal**
Gabriele, A.; Pacchioni, A. M.; See, R. E.	Dopamine and glutamate release in the dorsolateral caudate putamen following withdrawal from cocaine self-administration in rats	2012	103	373-379	Pharmacol Biochem Behav
Gaisler-Salomon, I.; Miller, G. M.; Chuhma, N.; Lee, S.; Zhang, H.; Ghoddoussi, F.; Lewandowski, N.; Fairhurst, S.; Wang, Y.; Conjard-Duplany, A.; Masson, J.; Balsam, P.; Hen, R.; Arancio, O.; Galloway, M. P.; Moore, H. M.; Small, S. A.; Rayport, S.	Glutaminase-deficient mice display hippocampal hypoactivity, insensitivity to pro-psychotic drugs and potentiated latent inhibition: relevance to schizophrenia	2009	34	2305-2322	Neuropsychopharmacology
Galeffi, F.; Bianchi, L.; Bolam, J. P.; Della Corte, L.	The effect of 6-hydroxydopamine lesions on the release of amino acids in the direct and indirect pathways of the basal ganglia: a dual microdialysis probe analysis	2003	18	856-868	Eur J Neurosci
Gallegos-Sanchez, J.; Picard, S.; Delaleu, B.; Malpaux, B.; Thiery, J. C.	Initiation of the oestradiol-induced inhibition of pulsatile LH secretion in ewes under long days: comparison of peripheral versus central treatment and neurochemical correlates	1996	151	19-28	J Endocrinol
Galluzzo, M.; Pintor, A.; Pezzola, A.; Grieco, R.; Borsini, F.; Popoli, P.	Behavioural and neurochemical characterization of the adenosine A2A receptor antagonist ST1535	2008	579	149-152	Eur J Pharmacol
Galvan, A.; Smith, Y.; Wichmann, T.	Continuous monitoring of intracerebral glutamate levels in awake monkeys using microdialysis and enzyme fluorometric detection	2003	126	175-185	J Neurosci Methods
Ganguly, P. K.; Maddaford, T. G.; Edel, A. L.; O, K.; Smeda, J. S.; Pierce, G. N.	Increased homocysteine-induced release of excitatory amino acids in the striatum of spontaneously hypertensive stroke-prone rats	2008	1226	192-198	Brain Res
Gao-Yu, C.; Cong-Yina, D.; Li-Jun, Z.; Fei, L.; Hua, F.	Effects of hyperbaric oxygen preconditioning on energy metabolism and glutamate level in the peri-infarct area following permanent MCAO	2011	38	91-99	Undersea Hyperb Med
Garcia Dopico, J.; Perdomo Diaz, J.; Alonso, T. J.; Gonzalez Hernandez, T.; Castro Fuentes, R.; Rodriguez Diaz, M.	Extracellular taurine in the substantia nigra: taurine-glutamate interaction	2004	76	528-538	J Neurosci Res
Garcia-Keller, C.; Martinez, S. A.; Esparza, M. A.; Bollati, F.; Kalivas, P. W.; Cancela, L. M.	Cross-sensitization between cocaine and acute restraint stress is associated with sensitized dopamine but not glutamate release in the nucleus accumbens	2013	37	982-995	Eur J Neurosci
Garcia-Medina, N. E.; Vera, G.; Miranda,	Chemical stimulation or glutamate injections in the nucleus of	2015	278	202-209	Behav Brain Res

Glutamate					
**Authors**	**Title**	**Year**	**Vol.**	**Pages**	**Journal**
M. I.	solitary tract enhance conditioned taste aversion				
Gardenfors, A.; Nilsson, F.; Skagerberg, G.; Ungerstedt, U.; Nordstrom, C. H.	Cerebral physiological and biochemical changes during vasogenic brain oedema induced by intrathecal injection of bacterial lipopolysaccharides in piglets	2002	144	601-609	Acta Neurochir (Wien)
Gaspary, H. L.; Simon, R. P.; Graham, S. H.	BW1003C87 and NBQX but not CGS19755 reduce glutamate release and cerebral ischemic necrosis	1994	262	197-203	Eur J Pharmacol
Ge, J.; Long, S. K.; Kilpatrick, I. C.	Preferential blockade of cholecystokinin-8S-induced increases in aspartate and glutamate levels by the CCK(B) receptor antagonist, L-365,260, in rat brain	1998	345	163-170	Eur J Pharmacol
Geddes, J. W.; Chang, N. G.; Ackley, D. C.; Soultanian, N. S.; McGillis, J. P.; Yokel, R. A.	Postmortem elevation in extracellular glutamate in the rat hippocampus when brain temperature is maintained at physiological levels: implications for the use of human brain autopsy tissues	1999	831	104-112	Brain Res
Geeraerts, T.; Ract, C.; Tardieu, M.; Fourcade, O.; Mazoit, J. X.; Benhamou, D.; Duranteau, J.; Vigue, B.	Changes in cerebral energy metabolites induced by impact-acceleration brain trauma and hypoxic-hypotensive injury in rats	2006	23	1059-1071	J Neurotrauma
Gemba, T.; Matsunaga, K.; Ueda, M.	Changes in extracellular concentration of amino acids in the hippocampus during cerebral ischemia in stroke-prone SHR, stroke-resistant SHR and normotensive rats	1992	135	184-188	Neurosci Lett
Gemba, T.; Ninomiya, M.; Matsunaga, K.; Ueda, M.	Effects of a novel calcium antagonist, S-(+)-methyl-4,7-dihydro-3-isobutyl-6- methyl-4-(3-nitrophenyl)thieno[2,3-b]pyridine-5-carboxylate (S-312-d), on ischemic amino acid release and neuronal injury in stroke-prone spontaneously hypertensive rats	1993	265	463-467	J Pharmacol Exp Ther
Georgescu, M.; Afonso, V. M.; Graham, M. D.; Pfaus, J. G.	Glutamate release in the ventromedial hypothalamus of the female rat during copulation: modulation by estradiol	2014	65	119-126	Horm Behav
Ghijsen, W. E.; da Silva Aresta Belo, A. I.; Zuiderwijk, M.; Lopez da Silva, F. H.	Compensatory change in EAAC1 glutamate transporter in rat hippocampus CA1 region during kindling epileptogenesis	1999	276	157-160	Neurosci Lett
Ghribi, O.; Callebert, J.; Plotkine, M.; Boulu, R. G.	L-NAME modulates glutamate accumulation induced by K(+)-depolarization but not by forebrain ischaemia in the rat striatum	1994	174	34-38	Neurosci Lett
Ghribi, O.; Callebert, J.; Plotkine, M.; Boulu, R. G.	Effect of kynurenic acid on the ischaemia-induced accumulation of glutamate in rat striatum	1994	5	435-437	Neuroreport
Ghribi, O.; Callebert, J.; Plotkine, M.; Boulu, R. G.	Competitive NMDA receptor blockers reduce striatal glutamate accumulation in ischaemia	1994	5	1253-1255	Neuroreport

Glutamate					
**Authors**	**Title**	**Year**	**Vol.**	**Pages**	**Journal**
Ghribi, O.; Callebert, J.; Verrecchia, C.; Plotkine, M.; Boulu, R. G.	Blockers of NMDA-operated channels decrease glutamate and aspartate extracellular accumulation in striatum during forebrain ischaemia in rats	1995	9	141-146	Fundam Clin Pharmacol
Gianfriddo, M.; Corsi, C.; Melani, A.; Pezzola, A.; Reggio, R.; Popoli, P.; Pedata, F.	Adenosine A(2A) antagonism increases striatal glutamate outflow in the quinolinic acid rat model of Huntington's disease	2003	979	225-229	Brain Res
Gianfriddo, M.; Melani, A.; Turchi, D.; Giovannini, M. G.; Pedata, F.	Adenosine and glutamate extracellular concentrations and mitogen-activated protein kinases in the striatum of Huntington transgenic mice. Selective antagonism of adenosine A2A receptors reduces transmitter outflow	2004	17	77-88	Neurobiol Dis
Giorgetti, M.; Hotsenpiller, G.; Froestl, W.; Wolf, M. E.	In vivo modulation of ventral tegmental area dopamine and glutamate efflux by local GABA(B) receptors is altered after repeated amphetamine treatment	2002	109	585-595	Neuroscience
Giorgetti, M.; Hotsenpiller, G.; Ward, P.; Teppen, T.; Wolf, M. E.	Amphetamine-induced plasticity of AMPA receptors in the ventral tegmental area: effects on extracellular levels of dopamine and glutamate in freely moving rats	2001	21	6362-6369	J Neurosci
Giovannini, M. G.; Pazzagli, M.; Malmberg-Aiello, P.; Della Corte, L.; Rakovska, A. D.; Cerbai, F.; Casamenti, F.; Pepeu, G.	Inhibition of acetylcholine-induced activation of extracellular regulated protein kinase prevents the encoding of an inhibitory avoidance response in the rat	2005	136	15-32	Neuroscience
Giovannini, M. G.; Rakovska, A.; Benton, R. S.; Pazzagli, M.; Bianchi, L.; Pepeu, G.	Effects of novelty and habituation on acetylcholine, GABA, and glutamate release from the frontal cortex and hippocampus of freely moving rats	2001	106	43-53	Neuroscience
Gipson, C. D.; Reissner, K. J.; Kupchik, Y. M.; Smith, A. C.; Stankeviciute, N.; Hensley-Simon, M. E.; Kalivas, P. W.	Reinstatement of nicotine seeking is mediated by glutamatergic plasticity	2013	110	9124-9129	Proc Natl Acad Sci U S A
Glass, J. D.; Hauser, U. E.; Blank, J. L.; Selim, M.; Rea, M. A.	Differential timing of amino acid and 5-HIAA rhythms in suprachiasmatic hypothalamus	1993	265	R504-R511	Am J Physiol
Glass, J. D.; Selim, M.; Srkalovic, G.; Rea, M. A.	Tryptophan loading modulates light-induced responses in the mammalian circadian system	1995	10	80-90	J Biol Rhythms
Globus, M. Y.; Alonso, O.; Dietrich, W. D.; Busto, R.; Ginsberg, M. D.	Glutamate release and free radical production following brain injury: effects of posttraumatic hypothermia	1995	65	1704-1711	J Neurochem
Globus, M. Y.; Busto, R.; Dietrich, W. D.;	Effect of ischemia on the in vivo release of striatal dopamine,	1988	51	1455-	J Neurochem

Glutamate					
**Authors**	**Title**	**Year**	**Vol.**	**Pages**	**Journal**
Martinez, E.; Valdes, I.; Ginsberg, M. D.	glutamate, and gamma-aminobutyric acid studied by intracerebral microdialysis			1464	
Globus, M. Y.; Busto, R.; Dietrich, W. D.; Martinez, E.; Valdes, I.; Ginsberg, M. D.	Intra-ischemic extracellular release of dopamine and glutamate is associated with striatal vulnerability to ischemia	1988	91	36-40	Neurosci Lett
Globus, M. Y.; Busto, R.; Martinez, E.; Valdes, I.; Dietrich, W. D.	Ischemia induces release of glutamate in regions spared from histopathologic damage in the rat	1990	21	43-46	Stroke
Globus, M. Y.; Busto, R.; Martinez, E.; Valdes, I.; Dietrich, W. D.; Ginsberg, M. D.	Comparative effect of transient global ischemia on extracellular levels of glutamate, glycine, and gamma-aminobutyric acid in vulnerable and nonvulnerable brain regions in the rat	1991	57	470-478	J Neurochem
Globus, M. Y.; Ginsberg, M. D.; Busto, R.	Excitotoxic index--a biochemical marker of selective vulnerability	1991	127	39-42	Neurosci Lett
Gobert, A.; Rivet, J. M.; Billiras, R.; Parsons, F.; Millan, M. J.	Simultaneous quantification of D- vs. L-serine, taurine, kynurenate, phosphoethanolamine and diverse amino acids in frontocortical dialysates of freely-moving rats: differential modulation by N-methyl-D-aspartate (NMDA) and other pharmacological agents	2011	202	143-157	J Neurosci Methods
Goda, H.; Ooboshi, H.; Nakane, H.; Ibayashi, S.; Sadoshima, S.; Fujishima, M.	Modulation of ischemia-evoked release of excitatory and inhibitory amino acids by adenosine A1 receptor agonist	1998	357	149-155	Eur J Pharmacol
Godukhin, O.	Effect of local cholecystokinin-8 administration on extracellular levels of amino acids and glycolytic products monitored by in vivo microdialysis in the fronto-parietal cortex of the rat	1995	194	29-32	Neurosci Lett
Goldsmith, J. D.; Kujawa, S. G.; McLaren, J. D.; Bledsoe Jr, S. C.	In vivo release of neuroactive amino acids from the inferior colliculus of the guinea pig using brain microdialysis	1995	83	80-88	Hear Res
Golembiowska, K.; Berghauzen-Maciejewska, K.; Gorska, A.; Kaminska, K.; Ossowska, K.	A partial lesion of the substantia nigra pars compacta and retrorubral field decreases the harmaline-induced glutamate release in the rat cerebellum	2013	1537	303-311	Brain Res
Golembiowska, K.; Dziubina, A.	Effect of acute and chronic administration of citalopram on glutamate and aspartate release in the rat prefrontal cortex	2000	52	441-448	Pol J Pharmacol
Golembiowska, K.; Dziubina, A.	Inhibition of amino acid release by 5-HT1B receptor agonist in the rat prefrontal cortex	2002	54	625-631	Pol J Pharmacol
Golembiowska, K.; Dziubina, A.	Involvement of adenosine in the effect of antidepressants on glutamate and aspartate release in the rat prefrontal cortex	2001	363	663-670	Naunyn Schmiedebergs Arch Pharmacol
Golembiowska, K.; Dziubina, A.	The effect of adenosine A(2A) receptor antagonists on hydroxyl radical, dopamine, and glutamate in the striatum of rats with	2012	22	150-157	Neurotox Res

Glutamate					
**Authors**	**Title**	**Year**	**Vol.**	**Pages**	**Journal**
	altered function of VMAT2				
Golembiowska, K.; Dziubina, A.	Effect of adenosine A(2A) receptor antagonists and L-DOPA on hydroxyl radical, glutamate and dopamine in the striatum of 6-OHDA-treated rats	2012	21	222-230	Neurotox Res
Golembiowska, K.; Zylewska, A.	Effect of antidepressant drugs on veratridine-evoked glutamate and aspartate release in rat prefrontal cortex	1999	51	63-70	Pol J Pharmacol
Golembiowska, K.; Zylewska, A.	N6-2-(4-aminophenyl)ethyladenosine (APNEA), a putative adenosine A3 receptor agonist, enhances methamphetamine-induced dopamine outflow in rat striatum	1998	50	299-305	Pol J Pharmacol
Golembiowska, K.; Zylewska, A.	Adenosine receptors--the role in modulation of dopamine and glutamate release in the rat striatum	1997	49	317-322	Pol J Pharmacol
Gong, C. L.; Chiu, Y. T.; Lin, N. N.; Lin, S. Z.; Cheng, F. C.; Kuo, J. S.	Inhibitory actions of serotonin on glutamate release in dorsal medulla suppress systemic arterial pressure of cats	2004	335	73-76	Neurosci Lett
Gong, J.; Gong, S.; Zhang, M.; Zhang, L.; Hu, Y.; Liu, Y.; Li, W.	Cerebral ischemic preconditioning reduces glutamate excitotoxicity by up-regulating the uptake activity of GLT-1 in rats	2014	46	1537-1545	Amino Acids
Gordon, K. E.; Simpson, J.; Statman, D.; Silverstein, F. S.	Effects of perinatal stroke on striatal amino acid efflux in rats studied with in vivo microdialysis	1991	22	928-932	Stroke
Goren, M. Z.; Aker, R.; Onat, F.; Ergun, A.	The effects of partial bilateral lesioning of substantia nigra in a genetic absence epilepsy rat model	2002	15	79-85	Marmara Med J
Goren, M. Z.; Kucukibrahimoglu, E.; Berkman, K.; Terzioglu, B.	Fluoxetine partly exerts its actions through GABA: a neurochemical evidence	2007	32	1559-1565	Neurochem Res
Goshima, Y.; Furukawa, N.; Arai, N.; Miyamae, T.; Sasaki, Y.; Hashimoto, M.; Yamamoto, I.; Fujita, K.; Akaike, A.; Misu, Y.	[A DOPA antagonist, DOPA cyclohexyl ester inhibits transient brain ischemia-induced release of glutamate and delayed neuronal cell death in striatal and hippocampal region of in vivo rats]	1999	114 Suppl 1	180-185	Nihon Yakurigaku Zasshi
Goyagi, T.; Nishikawa, T.; Tobe, Y.	Neuroprotective effects and suppression of ischemia-induced glutamate elevation by beta1-adrenoreceptor antagonists administered before transient focal ischemia in rats	2011	23	131-137	J Neurosurg Anesthesiol
Goyagi, T.; Nishikawa, T.; Tobe, Y.; Masaki, Y.	The combined neuroprotective effects of lidocaine and dexmedetomidine after transient forebrain ischemia in rats	2009	53	1176-1183	Acta Anaesthesiol Scand
Grabb, M. C.; Sciotti, V. M.; Gidday, J. M.; Cohen, S. A.; van Wylen, D. G.	Neurochemical and morphological responses to acutely and chronically implanted brain microdialysis probes	1998	82	25-34	J Neurosci Methods
Graham, S. H.; Chen, J.; Lan, J.; Leach,	Neuroprotective effects of a use-dependent blocker of voltage-	1994	269	854-859	J Pharmacol Exp Ther

Glutamate					
**Authors**	**Title**	**Year**	**Vol.**	**Pages**	**Journal**
M. J.; Simon, R. P.	dependent sodium channels, BW619C89, in rat middle cerebral artery occlusion				
Graham, S. H.; Shimizu, H.; Newman, A.; Weinstein, P.; Faden, A. I.	Opioid receptor antagonist nalmefene stereospecifically inhibits glutamate release during global cerebral ischemia	1993	632	346-350	Brain Res
Graham, S. H.; Shiraishi, K.; Panter, S. S.; Simon, R. P.; Faden, A. I.	Changes in extracellular amino acid neurotransmitters produced by focal cerebral ischemia	1990	110	124-130	Neurosci Lett
Gray, A. M.; Rawls, S. M.; Shippenberg, T. S.; McGinty, J. F.	The kappa-opioid agonist, U-69593, decreases acute amphetamine-evoked behaviors and calcium-dependent dialysate levels of dopamine and glutamate in the ventral striatum	1999	73	1066-1074	J Neurochem
Gray, T. K.; Lewis, E., 3rd; Maher, T. J.; Ally, A.	AMPA-receptor blockade within the RVLM modulates cardiovascular responses via glutamate during peripheral stimuli	2001	43	47-54	Pharmacol Res
Griffin Iii, W. C.; Haun, H. L.; Hazelbaker, C. L.; Ramachandra, V. S.; Becker, H. C.	Increased extracellular glutamate in the nucleus accumbens promotes excessive ethanol drinking in ethanol dependent mice	2014	39	707-717	Neuropsychopharmacology
Griffin, W. C.; Hazelbaker, C. L.; Sanchez, J. T.; Randall, J. S.; Becker, H. C.	Effects of chronic ethanol exposure and ethanol drinking on glutamate levels in the accumbens of C57BL/6J mice	2010	34 (6)	195A	Alcohol Clin Exp Res
Griffin, W. C.; Mulholland, P. J.; Deegan, D. L.; Sanchez, J. T.; Randall, J. S.; Chandler, L. J.; Becker, H. C.	Effects of chronic intermittent ethanol exposure on extracellular glutamate levels in the Accumbens of C57BL/6J mice	2009	33	82A	Alcohol Clin Exp Res
Griffin, W. C.; Ramachandra, V. S.; Knackstedt, L. A.; Becker, H. C.	Repeated cycles of chronic intermittent ethanol exposure increases basal glutamate in the nucleus accumbens of mice without affecting glutamate transport	2015	6	27	Front Pharmacol
Gruenbaum, S.; Dhaher, R.; Eid, T.	The role of increased branched-chain amino acids in the blood on brain extracellular fluid glutamate concentrations in naive rats	2015	27(4)	407-408	J Neurosurg Anesthesiol
Grupe, M.; Paolone, G.; Jensen, A. A.; Sandager-Nielsen, K.; Sarter, M.; Grunnet, M.	Selective potentiation of (alpha4)3(beta2)2 nicotinic acetylcholine receptors augments amplitudes of prefrontal acetylcholine- and nicotine-evoked glutamatergic transients in rats	2013	86	1487-1496	Biochem Pharmacol
Gruss, M.; Bock, J.; Braun, K.	Haloperidol impairs auditory filial imprinting and modulates monoaminergic neurotransmission in an imprinting-relevant forebrain area of the domestic chick	2003	87	686-696	J Neurochem
Gruss, M.; Braun, K.	Stimulus-evoked increase of glutamate in the mediorostral neostriatum/hyperstriatum ventrale of domestic chick after auditory filial imprinting: an in vivo microdialysis study	1996	66	1167-1173	J Neurochem
Gruss, M.; Bredenkotter, M.; Braun, K.	N-methyl-D-aspartate receptor-mediated modulation of	1999	40	116-135	J Neurobiol

Glutamate					
Authors	Title	Year	Vol.	Pages	Journal
	monoaminergic metabolites and amino acids in the chick forebrain: an in vivo microdialysis and electrophysiology study				
Gu, P. F.; Shang, Y.; Li, Y.	[Penehyclidine hydrochloride inhibits glutamate release and related research in global brain ischemia/reperfusion rats]. [Chinese]	2011	27	353-356	Zhongguo Ying Yong Sheng Li Xue Za Zhi
Gudelsky, G. A.; Roenker, N.; Ahlbrand, R.; Horn, P.; Richtand, N. M.	Evidence for involvement of nitric oxide and gaba-b receptors in mk-801-stimulated glutamate efflux in the rat prefrontal cortex	2012	38	S166-S167	Neuropsychopharmacology
Guerra-Romero, L.; Tureen, J. H.; Fournier, M. A.; Makrides, V.; Tauber, M. G.	Amino acids in cerebrospinal and brain interstitial fluid in experimental pneumococcal meningitis	1993	33	510-513	Pediatr Res
Guevara-Guzman, R.; Barrera-Mera, B.; De La Riva, C.; Kendrick, K. M.	Release of classical transmitters and nitric oxide in the rat olfactory bulb, evoked by vaginocervical stimulation and potassium, varies with the oestrus cycle	2000	12	80-88	Eur J Neurosci
Guevara-Guzman, R.; Buzo, E.; Larrazolo, A.; de la Riva, C.; Da Costa, A. P.; Kendrick, K. M.	Vaginocervical stimulation-induced release of classical neurotransmitters and nitric oxide in the nucleus of the solitary tract varies as a function of the oestrus cycle	2001	898	303-313	Brain Res
Guevara-Guzman, R.; Emson, P. C.; Kendrick, K. M.	Modulation of in vivo striatal transmitter release by nitric oxide and cyclic GMP	1994	62	807-810	J Neurochem
Gui, L.; Duan, W.; Tian, H.; Li, C.; Zhu, J.; Chen, J. F.; Zheng, J.	Adenosine A 2A receptor deficiency reduces striatal glutamate outflow and attenuates brain injury induced by transient focal cerebral ischemia in mice	2009	1297	185-193	Brain Res
Guo, Y.; Cai, H.; Chen, L.; Liang, D.; Yang, R.; Dang, R.; Jiang, P.	Quantitative profiling of neurotransmitter abnormalities in the hippocampus of rats treated with lipopolysaccharide: Focusing on kynurenine pathway and implications for depression	2016	295-296	41-46	J Neuroimmunol
Guo, Z.; Zhang, L.; Wu, Y.; Li, M.; Yang, X.; He, Z.; Wu, Z.; Hu, Y.; Jia, J.	The role of glutamate transporter-1 in the acquisition of brain ischaemic tolerance in rats induced by electro-acupuncture pre-treatment	2015	29	396-402	Brain Inj
Guzman-Ramos, K.; Moreno-Castilla, P.; Castro-Cruz, M.; McGaugh, J. L.; Martinez-Coria, H.; LaFerla, F. M.; Bermudez-Rattoni, F.	Restoration of dopamine release deficits during object recognition memory acquisition attenuates cognitive impairment in a triple transgenic mice model of Alzheimer's disease	2012	49	453-460	Learn Mem
Guzman-Ramos, K.; Osorio-Gomez, D.; Moreno-Castilla, P.; Bermudez-Rattoni, F.	Post-acquisition release of glutamate and norepinephrine in the amygdala is involved in taste-aversion memory consolidation	2012	19	231-238	Learn Mem

Glutamate					
**Authors**	**Title**	**Year**	**Vol.**	**Pages**	**Journal**
Guzman-Ramos, K.; Osorio-Gomez, D.; Moreno-Castilla, P.; Bermudez-Rattoni, F.	Off-line concomitant release of dopamine and glutamate involvement in taste memory consolidation	2010	114	226-236	J Neurochem
Gwag, B. J.; Sessler, F. M.; Robine, V.; Springer, J. E.	Endogenous glutamate levels regulate nerve growth factor mRNA expression in the rat dentate gyrus	1997	7	425-430	Mol Cells
Hada, J.; Kaku, T.; Morimoto, K.; Hayashi, Y.; Nagai, K.	Adenosine transport inhibitors enhance high K(+)-evoked taurine release from rat hippocampus	1996	305	101-107	Eur J Pharmacol
Haider, A.; Woodward, N. C.; Lominac, K. D.; Sacramento, A. D.; Klugmann, M.; Bell, R. L.; Szumlinski, K. K.	Homer2 within the nucleus accumbens core bidirectionally regulates alcohol intake by both P and Wistar rats	2015	49	533-542	Alcohol
Halpin, L. E.; Northrop, N. A.; Yamamoto, B. K.	Ammonia mediates methamphetamine-induced increases in glutamate and excitotoxicity	2014	39	1031-1038	Neurospychopharmacology
Hamann, M.; Sohr, R.; Morgenstern, R.; Richter, A.	Extracellular amino acid levels in the striatum of the dt(sz) mutant, a model of paroxysmal dystonia	2008	157	188-195	Neuroscience
Han, F.; Shioda, N.; Moriguchi, S.; Qin, Z. H.; Fukunaga, K.	Downregulation of glutamate transporters is associated with elevation in extracellular glutamate concentration following rat microsphere embolism	2008	430	275-280	Neurosci Lett
Han, J. L.; Blank, T.; Schwab, S.; Kollmar, R.	Inhibited glutamate release by granulocyte-colony stimulating factor after experimental stroke	2008	432	167-169	Neurosci Lett
Han, J.; Wan, H. T.; Yang, J. H.; Zhang, Y. Y.; Ge, L. J.; Bie, X. D.	Effect of ligustrazine on levels of amino acid neurotransmitters in rat striatum after cerebral ischemia-reperfusion injury	2014	16	1060-1067	J Asian Nat Prod Res
Han, J.; Wan, H.; Li, J.; Ge, L.	[Study on 3'-methoxy puerarin to dynamic changes of amino acids in rat brain ischemia-reperfusion]	2012	37	1023-1027	Zhongguo Zhong Yao Za Zhi
Han, W.; Qi, J.; Wang, F.; Zhang, L.; Zhao, S.; Song, M.; Wu, C.; Yang, J.	NMDA receptors in the medial prefrontal cortex and the dorsal hippocampus regulate methamphetamine-induced hyperactivity and extracellular amino acid release in mice	2012	232	44-52	Behav Brain Res
Han, Y.; Zuo, M.; Qi, L.; Liu, K.; Mao, L.; Chen, Y.	Online acid barrage stacking anti-salt injection for capillary electrophoresis of 9-fluorenylmethylchloroformate-derivatized amino acids in high ionic strength solutions by UV detection	2006	27	4240-4248	Electrophoresis
Hand, G. A.; Potts, J. T.; Treuhaft, B. S.; Wilson, L. B.; Petty, F.; Mitchell, J. H.	Static muscle contraction elicits a baroreflex-dependent increase in glutamate concentration in the ventrolateral medulla	1997	748	211-218	Brain Res
Hao, Y.; Yang, J. Y.; Guo, M.; Wu, C. F.; Wu, M. F.	Morphine decreases extracellular levels of glutamate in the anterior cingulate cortex: an in vivo microdialysis study in freely moving rats	2005	1040	191-196	Brain Res

Glutamate					
**Authors**	**Title**	**Year**	**Vol.**	**Pages**	**Journal**
Hao, Y.; Yang, J. Y.; Wu, C. F.; Wu, M. F.	Pseudoginsenoside-F11 decreases morphine-induced behavioral sensitization and extracellular glutamate levels in the medial prefrontal cortex in mice	2007	86	660-666	Pharmacol Biochem Behav
Hara, S.; Mukai, T.; Kurosaki, K.; Kuriiwa, F.; Endo, T.	Characterization of hydroxyl radical generation in the striatum of free-moving rats due to carbon monoxide poisoning, as determined by in vivo microdialysis	2004	1016	281-284	Brain Res
Harai, T.; Inoue, R.; Fujita, Y.; Tanaka, A.; Horio, M.; Hashimoto, K.; Hongou, K.; Miyawaki, T.; Mori, H.	Decreased susceptibility to seizures induced by pentylenetetrazole in serine racemase knockout mice	2012	102	180-187	Epilepsy Res
Harkany, T.; Abraham, I.; Laskay, G.; Timmerman, W.; Jost, K.; Zarandi, M.; Penke, B.; Nyakas, C.; Luiten, P. G.	Propionyl-IIGL tetrapeptide antagonizes beta-amyloid excitotoxicity in rat nucleus basalis	1999	10	1693-1698	Neuroreport
Harkany, T.; Abraham, I.; Timmerman, W.; Laskay, G.; Toth, B.; Sasvari, M.; Konya, C.; Sebens, J. B.; Korf, J.; Nyakas, C.; Zarandi, M.; Soos, K.; Penke, B.; Luiten, P. G.	beta-amyloid neurotoxicity is mediated by a glutamate-triggered excitotoxic cascade in rat nucleus basalis	2000	12	2735-2745	Eur J Neurosci
Harro, J.; Raudkivi, K.; Alttoa, A.	Extracellular glutamate levels in rats with high or low exploratory behaviour	2011	21	S376	European Neuropsychopharmacol
Harsing, L. G., Jr.; Matyus, P.	Mechanisms of glycine release, which build up synaptic and extrasynaptic glycine levels: the role of synaptic and non-synaptic glycine transporters	2013	93	110-119	Brain Res Bull
Harte, M.; Connor, W. T.	Evidence for a differential medial prefrontal dopamine D1 and D2 receptor regulation of local and ventral tegmental glutamate and GABA release: a dual probe microdialysis study in the awake rat	2004	1017	120-129	Brain Res
Harte, M.; Connor, W. T.	Evidence for a selective prefrontal cortical GABA(B) receptor-mediated inhibition of glutamate release in the ventral tegmental area: a dual probe microdialysis study in the awake rat	2005	130	215-222	Neuroscience
Hartmann, J.; Kiewert, C.; Klein, J.	Neurotransmitters and energy metabolites in amyloid-bearing APP(SWE)xPSEN1dE9 Mouse Brain	2010	332	364-370	J Pharmacol Exp Ther
Hasegawa, H.; Yazawa, T.; Yasumatsu, M.; Otokawa, M.; Aihara, Y.	Alteration in dopamine metabolism in the thermoregulatory center of exercising rats	2000	289	161-164	Neurosci Lett
Hasegawa, T.; Azum, S.; Inoue, S.	Amino acid release from the rat oral pontine reticular nucleus	2000	47	87-93	J Med Dent Sci

Glutamate					
**Authors**	**Title**	**Year**	**Vol.**	**Pages**	**Journal**
	across the sleep-wakefulness cycle				
Hashimoto, A.; Kanda, J.; Oka, T.	Effects of N-methyl-D-aspartate, kainate or veratridine on extracellular concentrations of free D-serine and L-glutamate in rat striatum: an in vivo microdialysis study	2000	53	347-351	Brain Res Bull
Hashimoto, A.; Oka, T.; Nishikawa, T.	Extracellular concentration of endogenous free D-serine in the rat brain as revealed by in vivo microdialysis	1995	66	635-643	Neuroscience
Hashimoto, K.; Fujita, Y.; Ishima, T.; Chaki, S.; Iyo, M.	Phencyclidine-induced cognitive deficits in mice are improved by subsequent subchronic administration of the glycine transporter-1 inhibitor NFPS and D-serine	2008	18	414-421	Eur Neuropsychopharmacol
Hashimoto, N.; Matsumoto, T.; Mabe, H.; Hashitani, T.; Nishino, H.	Dopamine has inhibitory and accelerating effects on ischemia-induced neuronal cell damage in the rat striatum	1994	33	281-288	Brain Res Bull
Haskew-Layton, R. E.; Rudkouskaya, A.; Jin, Y.; Feustel, P. J.; Kimelberg, H. K.; Mongin, A. A.	Two distinct modes of hypoosmotic medium-induced release of excitatory amino acids and taurine in the rat brain in vivo	2008	3	e3543	PLoS One
Hassel, B.; Dahlberg, D.; Mariussen, E.; Goverud, I. L.; Antal, E. A.; Tonjum, T.; Maehlen, J.	Brain infection with Staphylococcus aureus leads to high extracellular levels of glutamate, aspartate, gamma-aminobutyric acid, and zinc	2014	92	1792-1800	J Neurosci Res
Hathway, G. J.; Emson, P. C.; Humphrey, P. P.; Kendrick, K. M.	Somatostatin potently stimulates in vivo striatal dopamine and gamma-aminobutyric acid release by a glutamate-dependent action	1998	70	1740-1749	J Neurochem
Hatzipetros, T.; Raudensky, J. G.; Soghomonian, J. J.; Yamamoto, B. K.	Haloperidol treatment after high-dose methamphetamine administration is excitotoxic to GABA cells in the substantia nigra pars reticulata	2007	27	5895-5902	J Neurosci
Hatzipetros, T.; Yamamoto, B. K.	Dopaminergic and GABAergic modulation of glutamate release from rat subthalamic nucleus efferents to the substantia nigra	2006	1076	60-67	Brain Res
Haxhiu, M. A.; Yamamoto, B.; Dreshaj, I. A.; Bedol, D.; Ferguson, D. G.	Involvement of glutamate in transmission of afferent constrictive inputs from the airways to the nucleus tractus solitarius in ferrets	2000	80	22-30	J Auton Nerv Syst
Hazell, A. S.; Butterworth, R. F.; Hakim, A. M.	Cerebral vulnerability is associated with selective increase in extracellular glutamate concentration in experimental thiamine deficiency	1993	61	1155-1158	J Neurochem
Hegoburu, C.; Sevelinges, Y.; Thevenet, M.; Gervais, R.; Parrot, S.; Mouly, A. M.	Differential dynamics of amino acid release in the amygdala and olfactory cortex during odor fear acquisition as revealed with simultaneous high temporal resolution microdialysis	2009	16	687-697	Learn Mem

Glutamate					
**Authors**	**Title**	**Year**	**Vol.**	**Pages**	**Journal**
Hehre, D. A.; Devia, C. J.; Bancalari, E.; Suguihara, C.	Brainstem amino acid neurotransmitters and ventilatory response to hypoxia in piglets	2008	63	46-50	Pediatr Res
Heim, C.; Zhang, J.; Lan, J.; Sieklucka, M.; Kurz, T.; Riederer, P.; Gerlach, M.; Sontag, K. H.	Cerebral oligaemia episode triggers free radical formation and late cognitive deficiencies	2000	12	715-725	Eur J Neurosci
Hemmati, P.; Shilliam, C. S.; Hughes, Z. A.; Shah, A. J.; Roberts, J. C.; Atkins, A. R.; Hunter, A. J.; Heidbreder, C. A.	In vivo characterization of basal amino acid levels in subregions of the rat nucleus accumbens: effect of a dopamine D(3)/D(2) agonist	2001	39	199-208	Neurochem Int
Henderson, J. L.; Reynolds, J. D.; Dexter, F.; Atkins, B.; Hrdy, J.; Poduska, D.; Penning, D. H.	Chronic hypoxemia causes extracellular glutamate concentration to increase in the cerebral cortex of the near-term fetal sheep	1998	105	287-293	Brain Res Dev Brain Res
Herbison, A. E.; Voisin, D. L.; Douglas, A. J.; Chapman, C.	Profile of monoamine and excitatory amino acid release in rat supraoptic nucleus over parturition	1997	138	33-40	Endocrinology
Hernandez, L. F.; Segovia, G.; Mora, F.	Chronic treatment with a dopamine uptake blocker changes dopamine and acetylcholine but not glutamate and GABA concentrations in prefrontal cortex, striatum and nucleus accumbens of the awake rat	2008	52	457-469	Neurochem Int
Hernandez, L.; Escalona, J.; Verdeguer, P.; Guzman, N. A.	In vivo monitoring of brain glutamate by microdialysis coupled to capillary electrophoresis and laser induced fluorescence detection	1993	16(9-10)	2149-2160	J Liq Chromatogr
Hernandez, L.; Tucci, S.; Guzman, N.; Paez, X.	In vivo monitoring of glutamate in the brain by microdialysis and capillary electrophoresis with laser-induced fluorescence detection	1993	652	393-398	J Chromatogr A
Heron, A.; Lasbennes, F.; Seylaz, J.	Adenosine modulation of amino acid release in rat hippocampus during ischemia and veratridine depolarization	1993	608	27-32	Brain Res
Heron, A.; Lasbennes, F.; Seylaz, J.	Effects of adenosinergic compounds on extracellular release of glutamate. [French]	1992	9	111-122	Circulation et Metabolisme du Cerveau
Heron, A.; Lasbennes, F.; Seylaz, J.	Effect of two different routes of administration of R-PIA on glutamate release during ischemia	1992	147	205-208	Neurosci Lett
Heron, A.; Lekieffre, D.; Le Peillet, E.; Lasbennes, F.; Seylaz, J.; Plotkine, M.; Boulu, R. G.	Effects of an A1 adenosine receptor agonist on the neurochemical, behavioral and histological consequences of ischemia	1994	641	217-224	Brain Res
Heron, A.; Springhetti, V.; Seylaz, J.;	Effects of a glutamate uptake inhibitor on glutamate release	1995	26	593-599	Neurochem Int

Glutamate					
**Authors**	**Title**	**Year**	**Vol.**	**Pages**	**Journal**
Lasbennes, F.	induced by veratridine and ischemia				
Herrera, D. G.; Maysinger, D.; Goiny, M.	Induction of c-FOS immunoreactivity in the hippocampus following potassium stimulation	1993	52	237-244	Neuroscience
Herrera-Marschitz, M.; Javier Meana, J.; Hokfelt, T.; You, Z. B.; Morino, P.; Brodin, E.; Ungerstedt, U.	Cholecystokinin is released from a crossed corticostriatal pathway	1992	3	905-908	Neuroreport
Herrera-Marschitz, M.; Meana, J. J.; Connor, W. T.; Goiny, M.; Reid, M. S.; Ungerstedt, U.	Neuronal dependence of extracellular dopamine, acetylcholine, glutamate, aspartate and gamma-aminobutyric acid (GABA) measured simultaneously from rat neostriatum using in vivo microdialysis: reciprocal interactions	1992	2	157-179	Amino Acids
Herrera-Marschitz, M.; You, Z. B.; Goiny, M.; Meana, J. J.; Silveira, R.; Godukhin, O. V.; Chen, Y.; Espinoza, S.; Pettersson, E.; Loidl, C. F.; Lubec, G.; Andersson, K.; Nylander, I.; Terenius, L.; Ungerstedt, U.	On the origin of extracellular glutamate levels monitored in the basal ganglia of the rat by in vivo microdialysis	1996	66	1726-1735	J Neurochem
Hershey, N. D.; Kennedy, R. T.	In vivo calibration of microdialysis using infusion of stable-isotope labeled neurotransmitters	2013	4	729-736	ACS Chem Neurosci
Herz, R. C.; Gaillard, P. J.; de Wildt, D. J.; Versteeg, D. H.	Differences in striatal extracellular amino acid concentrations between Wistar and Fischer 344 rats after middle cerebral artery occlusion	1996	715	163-171	Brain Res
Hescham, S.; Jahanshahi, A.; Schweimer, J. V.; Mitchell, S. N.; Carter, G.; Blokland, A.; Sharp, T.; Temel, Y.	Fornix deep brain stimulation enhances acetylcholine levels in the hippocampus	2016	221	4281-4286	Brain Struct Funct
Higuera-Matas, A.; Miguens, M.; Coria, S. M.; Assis, M. A.; Borcel, E.; del Olmo, N.; Ambrosio, E.	Sex-specific disturbances of the glutamate/GABA balance in the hippocampus of adult rats subjected to adolescent cannabinoid exposure	2012	62	1975-1984	Neuropharmacology
Hilgier, W.; Kocki, T.; Obara-Michlewska, M.; Turski, W. A.; Oja, S. S.; Saransaari, P.; Albrecht, J.	Modulation by kynurenine of extracellular kynurenate and glutamate in cerebral cortex of rats with acute liver failure	2014	66	466-470	Pharmacol Rep
Hilgier, W.; Lazarewicz, J. W.; Struzynska, L.; Frontczak-Baniewicz, M.; Albrecht, J.	Repeated exposure of adult rats to Aroclor 1254 induces neuronal injury and impairs the neurochemical manifestations of the NMDA receptor-mediated intracellular signaling in the hippocampus	2012	33	16-22	Neurotoxicology

Glutamate					
**Authors**	**Title**	**Year**	**Vol.**	**Pages**	**Journal**
Hilgier, W.; Zielinska, M.; Borkowska, H. D.; Gadamski, R.; Walski, M.; Oja, S. S.; Saransaari, P.; Albrecht, J.	Changes in the extracellular profiles of neuroactive amino acids in the rat striatum at the asymptomatic stage of hepatic failure	1999	56	76-84	J Neurosci Res
Hiller, A.; Loeffler, S.; Haupt, C.; Litza, M.; Hofmann, U.; Moser, A.	Electrical high frequency stimulation of the caudate nucleus induces local GABA outflow in freely moving rats	2007	159	286-290	J Neurosci Methods
Hillered, L.; Hallstrom, A.; Segersvard, S.; Persson, L.; Ungerstedt, U.	Dynamics of extracellular metabolites in the striatum after middle cerebral artery occlusion in the rat monitored by intracerebral microdialysis	1989	9	607-616	J Cereb Blood Flow Metab
Hirota, H.	Role of glutamate release in anoxic depolarization of the brain	1994	36	37-47	Nihon Univ J Med
Hirota, H.; Katayama, Y.; Kawamata, T.; Kano, T.; Tsubokawa, T.	Inhibition of the high-affinity glutamate uptake system facilitates the massive potassium flux during cerebral ischaemia in vivo	1995	17	94-96	Neurol Res
Ho, Y. J.; Chang, Y. C.; Liu, T. M.; Tai, M. Y.; Wong, C. S.; Tsai, Y. F.	Striatal glutamate release during novelty exposure-induced hyperactivity in olfactory bulbectomized rats	2000	287	117-120	Neurosci Lett
Holmer, H. K.; Keyghobadi, M.; Moore, C.; Menashe, R. A.; Meshul, C. K.	Dietary restriction affects striatal glutamate in the MPTP-induced mouse model of nigrostriatal degeneration	2005	57	100-112	Synapse
Holmer, H. K.; Keyghobadi, M.; Moore, C.; Meshul, C. K.	l-dopa-induced reversal in striatal glutamate following partial depletion of nigrostriatal dopamine with 1-methyl-4-phenyl-1,2,3,6-tetrahydropyridine	2005	136	333-341	Neuroscience
Homola, A.; Zoremba, N.; Slais, K.; Kuhlen, R.; Sykova, E.	Changes in diffusion parameters, energy-related metabolites and glutamate in the rat cortex after transient hypoxia/ischemia	2006	404	137-142	Neurosci Lett
Honma, S.; Katsuno, Y.; Shinohara, K.; Abe, H.; Honma, K.	Circadian rhythm and response to light of extracellular glutamate and aspartate in rat suprachiasmatic nucleus	1996	271	R579-R585	Am J Physiol
Hoop, B.; Beagle, J. L.; Maher, T. J.; Kazemi, H.	Brainstem amino acid neurotransmitters and hypoxic ventilatory response	1999	118	117-129	Respir Physiol
Horio, M.; Kohno, M.; Fujita, Y.; Ishima, T.; Inoue, R.; Mori, H.; Hashimoto, K.	Levels of D-serine in the brain and peripheral organs of serine racemase (Srr) knock-out mice	2011	59	853-859	Neurochem Int
Horn, T.; Bauce, L.; Landgraf, R.; Pittman, Q. J.	Microdialysis with high NaCl causes central release of amino acids and dopamine	1995	64	1632-1644	J Neurochem
Horn, T.; Smith, P. M.; McLaughlin, B. E.; Bauce, L.; Marks, G. S.; Pittman, Q. J.; Ferguson, A. V.	Nitric oxide actions in paraventricular nucleus: cardiovascular and neurochemical implications	1994	266	R306-R313	Am J Physiol
Hoshi, K.; Ma, T.; Ho, I. K.	Precipitated kappa-opioid receptor agonist withdrawal increase glutamate in rat locus coeruleus	1996	314	301-306	Eur J Pharmacol

Glutamate					
**Authors**	**Title**	**Year**	**Vol.**	**Pages**	**Journal**
Hoshi, K.; Ma, T.; Oh, S.; Ho, I. K.	Increased release of excitatory amino acids in rat locus coeruleus in kappa-opioid agonist dependent rats precipitated by nor-binaltorphimine	1997	753	63-68	Brain Res
Hoshi, K.; Yamamoto, A.; Ishizuki, S.; Fujihira, E.; Ichihara, K.	Excitatory amino acid release in the locus coeruleus during naloxone-precipitated morphine withdrawal in adjuvant arthritic rats	2000	49	36-41	Inflamm Res
Hosmann, A.; Schober, A.; Gruber, A.; Sterz, F.; Testori, C.; Warenits, A.; Weihs, W.; Hogler, S.; Scherer, T.; Janata, A.; Laggner, A.; Zeitlinger, M.	Cerebral and Peripheral Metabolism to Predict Successful Reperfusion After Cardiac Arrest in Rats: A Microdialysis Study	2016	24	283-293	Neurocrit Care
Hotsenpiller, G.; Giorgetti, M.; Wolf, M. E.	Alterations in behaviour and glutamate transmission following presentation of stimuli previously associated with cocaine exposure	2001	14	1843-1855	Eur J Neurosci
Hotsenpiller, G.; Wolf, M. E.	Extracellular glutamate levels in prefrontal cortex during the expression of associative responses to cocaine related stimuli	2002	43	1218-1229	Neuropharmacology
Howes, S. R.; Dalley, J. W.; Morrison, C. H.; Robbins, T. W.; Everitt, B. J.	Leftward shift in the acquisition of cocaine self-administration in isolation-reared rats: relationship to extracellular levels of dopamine, serotonin and glutamate in the nucleus accumbens and amygdala-striatal FOS expression	2000	151	55-63	Psychopharmacology (Berl)
Hu, M.; Watson, C. J.; Kennedy, R. T.; Becker, J. B.	Estradiol attenuates the K+-induced increase in extracellular GABA in rat striatum	2006	59	122-124	Synapse
Hu, R. Q.; Banerjee, P. K.; Snead, O. C., 3rd	Regulation of gamma-aminobutyric acid (GABA) release in cerebral cortex in the gamma-hydroxybutyric acid (GHB) model of absence seizures in rat	2000	39	427-439	Neuropharmacology
Hu, X. J.; Wang, F. H.; Stenfors, C.; Ogren, S. O.; Kehr, J.	Effects of the 5-HT1B receptor antagonist NAS-181 on extracellular levels of acetylcholine, glutamate and GABA in the frontal cortex and ventral hippocampus of awake rats: a microdialysis study	2007	17	580-586	Eur Neuropsychopharmacol
Huang, F. P.; Zhou, L. F.; Yang, G. Y.	Effects of mild hypothermia on the release of regional glutamate and glycine during extended transient focal cerebral ischemia in rats	1998	23	991-996	Neurochem Res
Huang, M.; Felix, A. R.; Flood, D. G.; Bhuvaneswaran, C.; Hilt, D.; Koenig, G.;	The novel alpha7 nicotinic acetylcholine receptor agonist EVP-6124 enhances dopamine, acetylcholine, and glutamate efflux in	2014	231	4541-4551	Psychopharmacology (Berl)

Glutamate					
**Authors**	**Title**	**Year**	**Vol.**	**Pages**	**Journal**
Meltzer, H. Y.	rat cortex and nucleus accumbens				
Huang, M.; Li, Z.; Dai, J.; Shahid, M.; Wong, E. H.; Meltzer, H. Y.	Asenapine increases dopamine, norepinephrine, and acetylcholine efflux in the rat medial prefrontal cortex and hippocampus	2008	33	2934-2945	Neuropsychopharmacology
Huang, M.; Panos, J. J.; Kwon, S.; Oyamada, Y.; Rajagopal, L.; Meltzer, H. Y.	Comparative effect of lurasidone and blonanserin on cortical glutamate, dopamine, and acetylcholine efflux: role of relative serotonin (5-HT)2A and DA D2 antagonism and 5-HT1A partial agonism	2014	128	938-949	J Neurochem
Huang, N. K.; Tseng, C. J.; Wong, C. S.; Tung, C. S.	Effects of acute and chronic morphine on DOPAC and glutamate at subcortical DA terminals in awake rats	1997	56	363-371	Pharmacol Biochem Behav
Huang, W. T.; Niu, K. C.; Chang, C. K.; Lin, M. T.; Chang, C. P.	Curcumin inhibits the increase of glutamate, hydroxyl radicals and PGE2 in the hypothalamus and reduces fever during LPS-induced systemic inflammation in rabbits	2008	593	105-111	Eur J Pharmacol
Huang, W. T.; Tsai, S. M.; Lin, M. T.	Involvement of brain glutamate release in pyrogenic fever	2001	41	811-818	Neuropharmacology
Huang, W. T.; Wang, J. J.; Lin, M. T.	Antipyretic effect of acetaminophen by inhibition of glutamate release after staphylococcal enterotoxin A fever in rabbits	2004	355	33-36	Neurosci Lett
Hugues, S.; Garcia, R.; Lena, I.	Time course of extracellular catecholamine and glutamate levels in the rat medial prefrontal cortex during and after extinction of conditioned fear	2007	61	933-937	Synapse
Huo, T.; Zhang, Y.; Li, W.; Yang, H.; Jiang, H.; Sun, G.	Effect of realgar on extracellular amino acid neurotransmitters in hippocampal CA1 region determined by online microdialysis-dansyl chloride derivatization-high-performance liquid chromatography and fluorescence detection	2014	28	1254-1262	Biomed Chromatogr
Hwabejire, J. O.; Imam, A. M.; Jin, G.; Liu, B.; Li, Y.; Sillesen, M.; Jepsen, C. H.; Lu, J.; deMoya, M. A.; Alam, H. B.	Differential effects of fresh frozen plasma and normal saline on secondary brain damage in a large animal model of polytrauma, hemorrhage and traumatic brain injury	2013	75	968-975	J Trauma Acute Care Surg
Hwabejire, J. O.; Jin, G.; Imam, A. M.; Duggan, M.; Sillesen, M.; Deperalta, D.; Jepsen, C. H.; Lu, J.; Li, Y.; deMoya, M. A.; Alam, H. B.	Pharmacologic modulation of cerebral metabolic derangement and excitotoxicity in a porcine model of traumatic brain injury and hemorrhagic shock	2013	154	234-243	Surgery
Hwabejire, J. O.; Jin, G.; Imam, A. M.; Duggan, M.; Sillesen, M.; Deperalta, D.; Jepsen, C. H.; Lu, J.; Li, Y.; DeMoya, M.	Pharmacological modulation of mitochondrial dysfunction-induced cerebral metabolic derangement and excitotoxicity in a porcine model of traumatic brain injury and hemorrhagic shock	2013	179	322	J Surg Res Conference: 8th Annual Academic Surgical Congress of

Glutamate					
Authors	Title	Year	Vol.	Pages	Journal
A.; Velmahos, G. C.; Socrate, S.; Alam, H. B.					the Association for Academic Surgery, AAS and the Society of University Surgeons, SUS. New Orleans, LA United States. Conference Start
Hylland, P.; Nilsson, G. E.	Extracellular levels of amino acid neurotransmitters during anoxia and forced energy deficiency in crucian carp brain	1999	823	49-58	Brain Res
Hylland, P.; Nilsson, G. E.; Johansson, D.	Anoxic brain failure in an ectothermic vertebrate: release of amino acids and K+ in rainbow trout thalamus	1995	269	R1077-R1084	Am J Physiol
Hyzinski-Garcia, M. C.; Vincent, M. Y.; Haskew-Layton, R. E.; Dohare, P.; Keller Jr, R. W.; Mongin, A. A.	Hypo-osmotic swelling modifies glutamate-glutamine cycle in the cerebral cortex and in astrocyte cultures	2011	118	140-152	J Neurochem
Ichord, R. N.; Johnston, M. V.; Traystman, R. J.	MK801 decreases glutamate release and oxidative metabolism during hypoglycemic coma in piglets	2001	128	139-148	Brain Res Dev Brain Res
Ichord, R. N.; Northington, F. J.; van Wylen, D.; Johnston, M. V.; Kwon, C.; Traystman, R. J.	Brain O2 consumption and glutamate release during hypoglycemic coma in piglets are temperature sensitive	1999	276	H2053-H2062	Am J Physiol
Iijima, T.; Iwao, Y.; Sankawa, H.	Amino acid release during spreading depression in a flow-compromised cortical area	1999	818	553-555	Brain Res
Iijima, T.; Shimase, C.; Iwao, Y.; Sankawa, H.	Relationships between glutamate release, blood flow and spreading depression: real-time monitoring using an electroenzymatic dialysis electrode	1998	32	201-207	Neurosci Res
Iino, T.; Katsura, M.; Kuriyama, K.	Protective effect of vinconate on ischemia-induced neuronal damage in the rat hippocampus	1992	224	117-124	Eur J Pharmacol
Ikarashi, Y.; Iizuka, S.; Imamura, S.; Yamaguchi, T.; Sekiguchi, K.; Kanno, H.; Kawakami, Z.; Yuzurihara, M.; Kase, Y.; Takeda, S.	Effects of yokukansan, a traditional Japanese medicine, on memory disturbance and behavioral and psychological symptoms of dementia in thiamine-deficient rats	2009	32	1701-1709	Biol Pharm Bull
Ikeda, Y.; Toda, S.; Kawamoto, T.; Teramoto, A.	Arginine vasopressin release inhibitor RU51599 attenuates brain oedema following transient forebrain ischaemia in rats	1997	139	1166-1172	Acta Neurochir (Wien)
Illievich, U. M.; Zornow, M. H.; Choi, K. T.; Scheller, M. S.; Strnat, M. A.	Effects of hypothermic metabolic suppression on hippocampal glutamate concentrations after transient global cerebral ischemia	1994	78	905-911	Anesth Analg
Illievich, U. M.; Zornow, M. H.; Choi, K. T.;	Effects of hypothermia or anesthetics on hippocampal glutamate	1994	80	177-186	Anesthesiology

Glutamate					
**Authors**	**Title**	**Year**	**Vol.**	**Pages**	**Journal**
Strnat, M. A.; Scheller, M. S.	and glycine concentrations after repeated transient global cerebral ischemia				
Inglefield, J. R.; Perry, J. M.; Schwartz, R. D.	Postischemic inhibition of GABA reuptake by tiagabine slows neuronal death in the gerbil hippocampus	1995	5	460-468	Hippocampus
Inoue, S.; Yamanaka, T.; Kita, T.; Nakashima, T.; Hosoi, H.	Glutamate release in the rat medial vestibular nucleus following unilateral labyrinthectomy using in vivo microdialysis	2003	991	78-83	Brain Res
Iqbal, S.; Baziany, A.; Gordon, S.; Wright, S.; Hussain, M.; Miyashita, H.; Shuaib, A.; Hasan Rajput, A.	Neuroprotective effect of tiagabine in transient forebrain global ischemia: an in vivo microdialysis, behavioral, and histological study	2002	946	162-170	Brain Res
Irifune, M.; Kikuchi, N.; Saida, T.; Takarada, T.; Shimizu, Y.; Endo, C.; Morita, K.; Dohi, T.; Sato, T.; Kawahara, M.	Riluzole, a glutamate release inhibitor, induces loss of righting reflex, antinociception, and immobility in response to noxious stimulation in mice	2007	104	1415-1421	Anesth Analg
Ishide, T.; Hara, Y.; Maher, T. J.; Ally, A.	Glutamate neurotransmission and nitric oxide interaction within the ventrolateral medulla during cardiovascular responses to muscle contraction	2000	874	107-115	Brain Res
Ishide, T.; Maher, T. J.; Ally, A.	Role of nitric oxide in the ventrolateral medulla on cardiovascular responses and glutamate neurotransmission during mechanical and thermal stimuli	2003	47	59-68	Pharmacol Res
Ishide, T.; Maher, T. J.; Pearce, W. J.; Nauli, S. M.; Chaiyakul, P.; Ally, A.	Simultaneous glutamate and gamma-aminobutyric acid release within ventrolateral medulla during skeletal muscle contraction in intact and barodenervated rats	2001	923	137-146	Brain Res
Ishide, T.; Mancini, M.; Maher, T. J.; Chayaikul, P.; Ally, A.	Rostral ventrolateral medulla opioid receptor activation modulates glutamate release and attenuates the exercise pressor reflex	2000	865	177-185	Brain Res
Ishide, T.; Nauli, S. M.; Maher, T. J.; Ally, A.	Cardiovascular responses and neurotransmitter changes following blockade of nNOS within the ventrolateral medulla during static muscle contraction	2003	977	80-89	Brain Res
Ishide, T.; Preuss, C. V.; Maher, T. J.; Ally, A.	Neurochemistry within ventrolateral medulla and cardiovascular effects during static exercise following eNOS antagonism	2005	52	21-30	Neurosci Res
Ishiwata, S.; Umino, A.; Balu, D. T.; Coyle, J. T.; Nishikawa, T.	Neuronal serine racemase regulates extracellular D-serine levels in the adult mouse hippocampus	2015	122	1099-1103	J Neural Transm(Vienna)
Ito, K.; Abekawa, T.; Koyama, T.	Valproate blocks high-dose methamphetamine-induced behavioral cross-sensitization to locomotion-inducing effect of	2006	186	525-533	Psychopharmacology(Berl)

Glutamate					
**Authors**	**Title**	**Year**	**Vol.**	**Pages**	**Journal**
	dizocilpine (MK-801), but not methamphetamine				
Ito, K.; Abekawa, T.; Koyama, T.	Relationship between development of cross-sensitization to MK-801 and delayed increases in glutamate levels in the nucleus accumbens induced by a high dose of methamphetamine	2006	187	293-302	Psychopharmacology
Itoh, T.; Saito, T.; Fujimura, M.; Watanabe, S.; Saito, K.	Restraint stress-induced changes in endogenous zinc release from the rat hippocampus	1993	618	318-322	Brain Res
Iwasaki, K.; Kitamura, Y.; Ohgami, Y.; Mishima, K.; Fujiwara, M.	The disruption of spatial cognition and changes in brain amino acid, monoamine and acetylcholine in rats with transient cerebral ischemia	1996	709	163-172	Brain Res
Jacobson, I.; Hagberg, H.; Sandberg, M.; Hamberger, A.	Ouabain-induced changes in extracellular aspartate, glutamate and GABA levels in the rabbit olfactory bulb in vivo	1986	64	211-215	Neurosci Lett
Jacobson, I.; Sandberg, M.; Hamberger, A.	Mass transfer in brain dialysis devices--a new method for the estimation of extracellular amino acids concentration	1985	15	263-268	J Neurosi Methods
Jacobsson, S. O.; Cassel, G. E.; Karlsson, B. M.; Sellstrom, A.; Persson, S. A.	Release of dopamine, GABA and EAA in rats during intrastriatal perfusion with kainic acid, NMDA and soman: a comparative microdialysis study	1997	71	756-765	Arch Toxicol
Jacobsson, S. O.; Sellstrom, A.; Persson, S. A.; Cassel, G. E.	Correlation between cortical EEG and striatal microdialysis in soman-intoxicated rats	1997	231	155-158	Neurosci Lett
Jalal, F. Y.; Bohlke, M.; Maher, T. J.	Acetyl-L-carnitine reduces the infarct size and striatal glutamate outflow following focal cerebral ischemia in rats	2010	1199	95-104	Ann N Y Acad Sci
Jamal, M.; Ono, J.; Ameno, K.; Shirakami, G.; Tanaka, N.; Takakura, A.; Kinoshita, H.	Aldehyde dehydrogenase 2 deficiency increases resting-state glutamate and expression of the GluN1 subunit of N-methyl-D-aspartate receptor in the frontal cortex of mice	2015	348	46-50	J Neurol Sci
Jay, T. M.; Zilkha, E.; Obrenovitch, T. P.	Long-term potentiation in the dentate gyrus is not linked to increased extracellular glutamate concentration	1999	81	1741-1748	J Neurophysiol
Jayaram, P.; Steketee, J. D.	Effects of repeated cocaine on medial prefrontal cortical GABAB receptor modulation of neurotransmission in the mesocorticolimbic dopamine system	2004	90	839-847	J Neurochem
Jellish, W. S.; Murdoch, J.; Kindel, G.; Zhang, X.; White, F. A.	The effect of clonidine on cell survival, glutamate, and aspartate release in normo- and hyperglycemic rats after near complete forebrain ischemia	2005	167	526-534	Exp Brain Res
Jia, J.; Hu, Y. S.; Wu, Y.; Liu, G.; Yu, H. X.; Zheng, Q. P.; Zhu, D. N.; Xia, C. M.;	Pre-ischemic treadmill training affects glutamate and gamma aminobutyric acid levels in the striatal dialysate of a rat model of	2009	84	505-511	Life Sci

Glutamate					
**Authors**	**Title**	**Year**	**Vol.**	**Pages**	**Journal**
Cao, Z. J.	cerebral ischemia				
Jiang, M. H.; Kaku, T.; Hada, J.; Hayashi, Y.	Different effects of eNOS and nNOS inhibition on transient forebrain ischemia	2002	946	139-147	Brain Res
Jiao, Y.; Wang, D. Q.; Wu, Z. E.; Fan, B.; Peng, J.; Wang, X. Y.	The effect of extracellular amino acid neurotransmitters in striatum of rats by intracerebral perfusion of 6-OHDA and Madopar intervene. [Chinese]	2009	25	1055-1059	Zhongguo yao li xue tong bao
Jimenez-Diaz, L.; Gruart, A.; Minano, F. J.; Delgado-Garcia, J. M.	An experimental study of posterior interpositus involvement in the genesis and control of conditioned eyelid responses	2002	978	106-118	Ann N Y Acad Sci
Jimenez-Sanchez, L.; Castane, A.; Perez-Caballero, L.; Grifoll-Escoda, M.; Lopez-Gil, X.; Campa, L.; Galofre, M.; Berrocoso, E.; Adell, A.	Activation of AMPA Receptors Mediates the Antidepressant Action of Deep Brain Stimulation of the Infralimbic Prefrontal Cortex	2016	26	2778-289	Cereb Cortex
Jin, Q. H.; Ueda, Y.; Ishizuka, Y.; Kunitake, T.; Kannan, H.	Cardiovascular changes induced by central hypertonic saline are accompanied by glutamate release in awake rats	2001	281	R1224-R1231	Am J Physiol Regul Integr Comp Physiol
Jin, Y.; Zhang, B. Y.; Yao, S. Y.; Liu, Z.; Liu, W. Z.; Zhao, Y. J.	Effect of activation or blocking of N-methyl-D-aspartate receptors on release of excitatory amino acids in rat brain hippocampus. [Chinese]	2005	9	141-143	Zhongguo kang fu yi xue hui
John, J.; Kodama, T.; Siegel, J. M.	Caffeine promotes glutamate and histamine release in the posterior hypothalamus	2014	307	R704-R710	Am J Physiol Regul Integr Comp Physiol
John, J.; Ramanathan, L.; Siegel, J. M.	Rapid changes in glutamate levels in the posterior hypothalamus across sleep-wake states in freely behaving rats	2008	295	R2041-R2049	Am J Physiol Regul Integr Comp Physiol
John, J.; Wu, M. F.; Kodama, T.; Siegel, J. M.	Intravenously administered hypocretin-1 alters brain amino acid release: an in vivo microdialysis study in rats	2003	548	557-562	J Physiol
Jones, N. M.; Lawrence, A. J.; Beart, P. M.	In vivo microdialysis reveals facilitatory metabotropic glutamate receptors regulating excitatory amino acid release in rat nucleus tractus solitarius	1998	32	31-38	Neurochem Int
Jonkers, N.; Sarre, S.; Ebinger, G.; Michotte, Y.	MK801 suppresses the L-DOPA-induced increase of glutamate in striatum of hemi-Parkinson rats	2002	926	149-155	Brain Res
Juarez, B. I.; Martinez, M. L.; Montante, M.; Dufour, L.; Garcia, E.; Jimenez-Capdeville, M. E.	Methylmercury increases glutamate extracellular levels in frontal cortex of awake rats	2002	24	767-771	Neurotoxicol Teratol
Juhasz, G.; Kekesi, K. A.; Nyitrai, G.; Dobolyi, A.; Krogsgaard-Larsen, P.;	Differential effects of nipecotic acid and 4,5,6,7-tetrahydroisoxazolo[4,5-c]pyridin-3-ol on extracellular gamma-	1997	331	139-144	Eur J Pharmacol

Glutamate					
**Authors**	**Title**	**Year**	**Vol.**	**Pages**	**Journal**
Schousboe, A.	aminobutyrate levels in rat thalamus				
Kahn, R. A.; Panah, M.; Kiffel, S.; Weinberger, J.	Modulation of ischemic excitatory neurotransmitter and gamma-aminobutyric acid release during global temporary cerebral ischemia by local nitric oxide synthase inhibition	1997	84	1004-1010	Anesth Analg
Kahn, R. A.; Panah, M.; Weinberger, J.	Modulation of ischemic excitatory neurotransmitter and gamma-aminobutyric acid release during global temporary cerebral ischemia by selective neuronal nitric oxide synthase inhibition	1997	84	997-1003	Anesth Analg
Kaku, T.; Hada, J.; Hayashi, Y.	Endogenous adenosine exerts inhibitory effects upon the development of spreading depression and glutamate release induced by microdialysis with high K+ in rat hippocampus	1994	658	39-48	Brain Res
Kalinine, E.; Zimmer, E. R.; Zenki, K. C.; Kalinine, I.; Kazlauckas, V.; Haas, C. B.; Hansel, G.; Zimmer, A. R.; Souza, D. O.; Muller, A. P.; Portela, L. V.	Nandrolone-induced aggressive behavior is associated with alterations in extracellular glutamate homeostasis in mice	2014	66	383-392	Horm Behav
Kalivas, P. W.; Duffy, P.	D1 receptors modulate glutamate transmission in the ventral tegmental area	1995	15	5379-5388	J Neurosci
Kalivas, P. W.; Duffy, P.	Repeated cocaine administration alters extracellular glutamate in the ventral tegmental area	1998	70	1497-1502	J Neurochem
Kalivas, P. W.; Duffy, P.	Dopamine regulation of extracellular glutamate in the nucleus accumbens	1997	761	173-177	Brain Res
Kamikawa, H.; Hori, T.; Nakane, H.; Aou, S.; Tashiro, N.	IL-1beta increases norepinephrine level in rat frontal cortex: involvement of prostanoids, NO, and glutamate	1998	275	R803-R810	Am J Physiol
Kaminski, R. M.; Nunez-Taltavull, J. F.; Budziszewska, B.; Lason, W.; Gasior, M.; Zapata, A.; Shippenberg, T. S.; Witkin, J. M.	Effects of cocaine-kindling on the expression of NMDA receptors and glutamate levels in mouse brain	2011	36	146-152	Neurochem Res
Kanamori, K.	Disinhibition reduces extracellular glutamine and elevates extracellular glutamate in rat hippocampus in vivo	2015	114	32-46	Epilepsy Res
Kanamori, K.; Kondrat, R. W.; Ross, B. D.	13C enrichment of extracellular neurotransmitter glutamate in rat brain--combined mass spectrometry and NMR studies of neurotransmitter turnover and uptake into glia in vivo	2003	49	819-836	Cell Mol Biol
Kanamori, K.; Ross, B. D.	Electrographic seizures are significantly reduced by in vivo inhibition of neuronal uptake of extracellular glutamine in rat	2013	107	20-36	Epilepsy Res

Glutamate					
**Authors**	**Title**	**Year**	**Vol.**	**Pages**	**Journal**
	hippocampus				
Kanamori, K.; Ross, B. D.	Suppression of glial glutamine release to the extracellular fluid studied in vivo by NMR and microdialysis in hyperammonemic rat brain	2005	94	74-85	J Neurochem
Kanamori, K.; Ross, B. D.	Chronic electrographic seizure reduces glutamine and elevates glutamate in the extracellular fluid of rat brain	2011	1371	180-191	Brain Res
Kanamori, K.; Ross, B. D.; Kondrat, R. W.	Glial uptake of neurotransmitter glutamate from the extracellular fluid studied in vivo by microdialysis and (13)C NMR	2002	83	682-695	J Neurochem
Kanda, T.; Kurokawa, M.; Tamura, S.; Nakamura, J.; Ishii, A.; Kuwana, Y.; Serikawa, T.; Yamada, J.; Ishihara, K.; Sasa, M.	Topiramate reduces abnormally high extracellular levels of glutamate and aspartate in the hippocampus of spontaneously epileptic rats (SER)	1996	59	1607-1616	Life Sci
Kang, L.; Dai, Z. Z.; Li, H. H.; Ma, L.	Environmental cues associated with morphine modulate release of glutamate and gamma-aminobutyric acid in ventral subiculum	2006	22	255-260	Neurosci Bull
Kang, S. W.; Choi, S. K.; Park, E.; Chae, S. J.; Choi, S.; Jin Joo, H.; Lee, G. J.; Park, H. K.	Neuroprotective effects of magnesium-sulfate on ischemic injury mediated by modulating the release of glutamate and reduced of hyperreperfusion	2011	1371	121-128	Brain Res
Kano, T.; Katayama, Y.; Kawamata, T.; Hirota, H.; Tsubokawa, T.	Propentofylline administered by microdialysis attenuates ischemia-induced hippocampal damage but not excitatory amino acid release in gerbils	1994	641	149-154	Brain Res
Kano, T.; Shimizu-Sasamata, M.; Huang, P. L.; Moskowitz, M. A.; Lo, E. H.	Effects of nitric oxide synthase gene knockout on neurotransmitter release in vivo	1998	86	695-699	Neuroscience
Kanthan, R.; Shuaib, A.; Goplen, G.; Miyashita, H.	A new method of in-vivo microdialysis of the human brain	1995	60	151-155	J Neurosci Methods
Kao, C. H.; Huang, W. T.; Lin, M. T.; Wu, W. S.	Central interleukin-10 attenuated lipopolysaccharide-induced changes in core temperature and hypothalamic glutamate, hydroxyl radicals and prostaglandin-E(2)	2011	654	187-193	Eur J Pharmacol
Kapasova, Z.; Szumlinski, K. K.	Strain differences in alcohol-induced neurochemical plasticity: a role for accumbens glutamate in alcohol intake	2008	32	617-631	Alcohol Clin Exp Res
Kapoor, V.; Minson, J.; Chalmers, J.	Ventral medulla stimulation increases blood pressure and spinal cord amino acid release	1992	3	55-58	Neuroreport
Kapoor, V.; Nakahara, D.; Blood, R. J.; Chalmers, J. P.	Preferential release of neuroactive amino acids from the ventrolateral medulla of the rat in vivo as measured by	1990	37	187-191	Neuroscience

Glutamate					
**Authors**	**Title**	**Year**	**Vol.**	**Pages**	**Journal**
	microdialysis				
Karlsson, G. A.; Chaitoff, K. A.; Hossain, S.; Bohlke, M.; Maher, T. J.; Ally, A.	Modulation of cardiovascular responses and neurotransmission during peripheral nociception following nNOS antagonism within the periaqueductal gray	2007	1143	150-160	Brain Res
Karlsson, R. M.; Adermark, L.; Molander, A.; Perreau-Lenz, S.; Singley, E.; Solomon, M.; Holmes, A.; Tanaka, K.; Lovinger, D. M.; Spanagel, R.; Heilig, M.	Reduced alcohol intake and reward associated with impaired endocannabinoid signaling in mice with a deletion of the glutamate transporter GLAST	2012	63	181-189	Neuropharmacology
Karreman, M.; Moghaddam, B.	Effect of a pharmacological stressor on glutamate efflux in the prefrontal cortex	1996	716	180-182	Brain Res
Kashkin, V. A.; De Witte, P.	Nicotine increases microdialysate brain amino acid concentrations and induces conditioned place preference	2005	15	625-632	Eur Neuropsychopharmacol
Kasper, J. M.; Booth, R. G.; Peris, J.	Serotonin-2C receptor agonists decrease potassium-stimulated GABA release in the nucleus accumbens	2015	69	78-85	Synapse
Katayama, Y.; Becker, D. P.; Tamura, T.; Hovda, D. A.	Massive increases in extracellular potassium and the indiscriminate release of glutamate following concussive brain injury	1990	73	889-900	J Neurosurg
Katayama, Y.; Kawamata, T.; Tamura, T.; Hovda, D. A.; Becker, D. P.; Tsubokawa, T.	Calcium-dependent glutamate release concomitant with massive potassium flux during cerebral ischemia in vivo	1991	558	136-140	Brain Res
Katoh, H.; Shima, K.; Nawashiro, H.; Wada, K.; Chigasaki, H.	Selective hippocampal damage to hypoxia after mild closed head injury in the rat	1998	Supplement 71	247-249	Acta neurochirurgica
Katoh, H.; Sima, K.; Nawashiro, H.; Wada, K.; Chigasaki, H.	The effect of MK-801 on extracellular neuroactive amino acids in hippocampus after closed head injury followed by hypoxia in rats	1997	758	153-162	Brain Res
Katz, L. M.; Wang, Y.; Rockoff, S.; Bouldin, T. W.	Low-dose Carbicarb improves cerebral outcome after asphyxial cardiac arrest in rats	2002	39	359-365	Ann Emerg Med
Kaul, S.; Faiman, M. D.; Lunte, C. E.	Determination of GABA, glutamate and carbamathione in brain microdialysis samples by capillary electrophoresis with fluorescence detection	2011	32	284-291	Electrophoresis
Kaura, S.; Bradford, H. F.; Young, A. M.; Croucher, M. J.; Hughes, P. D.	Effect of amygdaloid kindling on the content and release of amino acids from the amygdaloid complex: in vivo and in vitro studies	1995	65	1240-1249	J Neurochem
Kawaguchi, K.; Huerbin, M.; Simon, R. P.	Lesioning of deep prepiriform cortex protects against ischemic neuronal necrosis by attenuating extracellular glutamate	1997	69	412-417	J Neurochem

Glutamate					
**Authors**	**Title**	**Year**	**Vol.**	**Pages**	**Journal**
	concentrations				
Kawahara, N.; Ruetzler, C. A.; Klatzo, I.	Protective effect of spreading depression against neuronal damage following cardiac arrest cerebral ischaemia	1995	17	9-16	Neurol Res
Kawamata, T.; Omote, K.; Toriyabe, M.; Kawamata, M.; Namiki, A.	Involvement of capsaicin-sensitive fibers in spinal NMDA-induced glutamate release	2001	12	3447-3450	Neuroreport
Kawamoto, T.; Ikeda, Y.; Teramoto, A.	[Protective effect of L-histidine (singlet oxygen scavenger) on transient forebrain ischemia in the rat]	1997	49	612-618	No To Shinkei
Keck, M. E.; Sillaber, I.; Ebner, K.; Welt, T.; Toschi, N.; Kaehler, S. T.; Singewald, N.; Philippu, A.; Elbel, G. K.; Wotjak, C. T.; Holsboer, F.; Landgraf, R.; Engelmann, M.	Acute transcranial magnetic stimulation of frontal brain regions selectively modulates the release of vasopressin, biogenic amines and amino acids in the rat brain	2000	12	3713-3720	Eur J Neurosci
Keefe, K. A.; Sved, A. F.; Zigmond, M. J.; Abercrombie, E. D.	Stress-induced dopamine release in the neostriatum: evaluation of the role of action potentials in nigrostriatal dopamine neurons or local initiation by endogenous excitatory amino acids	1993	61	1943-1952	J Neurochem
Kehr, J.	Determination of glutamate and aspartate in microdialysis samples by reversed-phase column liquid chromatography with fluorescence and electrochemical detection	1998	708	27-38	J Chromatogr B Biomed Sci Appl
Kehr, J.; Hu, X. J.; Yoshitake, T.; Wang, F. H.; Osborne, P.; Stenfors, C.; Ogren, S. O.	The selective 5-HT(1A) receptor antagonist NAD-299 increases acetylcholine release but not extracellular glutamate levels in the frontal cortex and hippocampus of awake rat	2010	20	487-500	Eur Neuropsychopharmacol
Keith, R. A.; Mangano, T. J.; Lampe, R. A.; DeFeo, P. A.; Hyde, M. J.; Donzanti, B. A.	Comparative actions of synthetic omega-grammotoxin SIA and synthetic omega-Aga-IVA on neuronal calcium entry and evoked release of neurotransmitters in vitro and in vivo	1995	34	1515-1528	Neuropharmacology
Kekesi, K. A.; Dobolyi, A.; Salfay, O.; Nyitrai, G.; Juhasz, G.	Slow wave sleep is accompanied by release of certain amino acids in the thalamus of cats	1997	8	1183-1186	Neuroreport
Kekesi, K. A.; Szilagyi, N.; Nyitrai, G.; Dobolyi, A.; Skuban, N.; Kardos, J.	Persistent depolarization and Glu uptake inhibition operate distinct osmoregulatory mechanisms in the mammalian brain	2000	37	171-178	Neurochem Int
Kelly, C. J.; Huang, M.; Meltzer, H.; Martina, M.	Reduced Glutamatergic Currents and Dendritic Branching of Layer 5 Pyramidal Cells Contribute to Medial Prefrontal Cortex Deactivation in a Rat Model of Neuropathic Pain	2016	10	133	Front Cell Neurosci
Kemppainen, H.; Nurmi, H.; Raivio, N.; Kiianmaa, K.	Enhanced Extracellular Glutamate and Dopamine in the Ventral Pallidum of Alcohol-Preferring AA and Alcohol-Avoiding ANA Rats after Morphine	2015	6	1	Front Psychiatry

Glutamate					
**Authors**	**Title**	**Year**	**Vol.**	**Pages**	**Journal**
Kemppainen, H.; Raivio, N.; Nurmi, H.; Kiianmaa, K.	GABA and glutamate overflow in the VTA and ventral pallidum of alcohol-preferring AA and alcohol-avoiding ANA rats after ethanol	2010	45	111-118	Alcohol Alcohol
Kendrick, K. M.; Fabre-Nys, C.; Blache, D.; Goode, J. A.; Broad, K. D.	The role of oxytocin release in the mediobasal hypothalamus of the sheep in relation to female sexual receptivity	1993	5	13-21	J Neuroendocrinol
Kendrick, K. M.; Guevara-Guzman, R.; de la Riva, C.; Christensen, J.; Ostergaard, K.; Emson, P. C.	NMDA and kainate-evoked release of nitric oxide and classical transmitters in the rat striatum: in vivo evidence that nitric oxide may play a neuroprotective role	1996	8	2619-2634	Eur J Neurosci
Kendrick, K. M.; Guevara-Guzman, R.; Zorrilla, J.; Hinton, M. R.; Broad, K. D.; Mimmack, M.; Ohkura, S.	Formation of olfactory memories mediated by nitric oxide	1997	388	670-674	Nature
Kendrick, K. M.; Hinton, M. R.	Microdialysis sampling of in vivo neurotransmitter release in the brain of the conscious sheep: Measurement of acetylcholine, amino acid and monoamine concentrations by high performance liquid chromatography (HPLC)	1992	446	66P	J Physiol
Kendrick, K. M.; Keverne, E. B.; Chapman, C.; Baldwin, B. A.	Microdialysis measurement of oxytocin, aspartate, gamma-aminobutyric acid and glutamate release from the olfactory bulb of the sheep during vaginocervical stimulation	1988	442	171-174	Brain Res
Kendrick, K. M.; Keverne, E. B.; Hinton, M. R.; Goode, J. A.	Oxytocin, amino acid and monoamine release in the region of the medial preoptic area and bed nucleus of the stria terminalis of the sheep during parturition and suckling	1992	569	199-209	Brain Res
Kennedy, R. T.; Thompson, J. E.; Vickroy, T. W.	In vivo monitoring of amino acids by direct sampling of brain extracellular fluid at ultralow flow rates and capillary electrophoresis	2002	114	39-49	J Neurosci Methods
Keverne, E. B.; Levy, F.; Guevara-Guzman, R.; Kendrick, K. M.	Influence of birth and maternal experience on olfactory bulb neurotransmitter release	1993	56	557-565	Neuroscience
Khan, G. M.; Smolders, I.; Ebinger, G.; Michotte, Y.	Anticonvulsant effect and neurotransmitter modulation of focal and systemic 2-chloroadenosine against the development of pilocarpine-induced seizures	2000	39	2418-2432	Neuropharmacology
Khan, G. M.; Smolders, I.; Ebinger, G.; Michotte, Y.	Flumazenil prevents diazepam-elicited anticonvulsant action and concomitant attenuation of glutamate overflow	2000	407	139-144	Eur J Pharmacol
Khan, G. M.; Smolders, I.; Lindekens, H.; Manil, J.; Ebinger, G.; Michotte, Y.	Effects of diazepam on extracellular brain neurotransmitters in pilocarpine-induced seizures in rats	1999	373	153-161	Eur J Pharmacol
Kim, H. K.; Zornow, M. H.; Strnat, M. A.;	Dexmedetomidine does not attenuate increases in excitatory	1996	8	230-236	J Neurosurg Anesthesiol

Glutamate					
**Authors**	**Title**	**Year**	**Vol.**	**Pages**	**Journal**
Maze, M.	amino acids after transient global ischemia in the rabbit				
Kim, J. H.; Austin, J. D.; Tanabe, L.; Creekmore, E.; Vezina, P.	Activation of group II mGlu receptors blocks the enhanced drug taking induced by previous exposure to amphetamine	2005	21	295-300	Eur J Neurosci
Kippin, T. E.; Szumlinski, K. K.; Kapasova, Z.; Rezner, B.; See, R. E.	Prenatal stress enhances responsiveness to cocaine	2008	33	769-782	Neuropsychopharmacology
Kirschner, D. L.; Jaramillo, M.; Green, T. K.	Enantioseparation and stacking of Cyanobenz[f]isoindole-amino acids by reverse polarity capillary electrophoresis and sulfated beta-cyclodextrin	2007	79	736-743	Anal Chem
Kishi, T.; Hirooka, Y.; Sakai, K.; Shigematsu, H.; Shimokawa, H.; Takeshita, A.	Overexpression of eNOS in the RVLM causes hypotension and bradycardia via GABA release	2001	38	896-901	Hypertension
Kishi, T.; Hirooka, Y.; Sunagawa, K.	Telmisartan inhibits sympathetic nerve activity through the inhibition of oxidative stress and increase in NO and GABA in the brain of hypertensive rats	2010	22	S60	Nitric Oxide
Kitaichi, Y.; Hashimoto, R.; Inoue, T.; Abekawa, T.; Kakuta, A.; Hattori, S.; Koyama, T.	Abnormalities in extracellular glycine and glutamate levels in the striatum of sandy mice	2013	25	215-220	Acta Neuropsychiatr
Kitta, T.; Matsumoto, M.; Tanaka, H.; Mitsui, T.; Yoshioka, M.; Nonomura, K.	GABAergic mechanism mediated via D receptors in the rat periaqueductal gray participates in the micturition reflex: an in vivo microdialysis study	2008	27	3216-3225	Eur J Neurosci
Kling, A. S.; Tachiki, K.; Lloyd, R.	Neurochemical correlates of the Kluver-Bucy syndrome by in vivo microdialysis in monkey	1993	56	161-170	Behav Brain Res
Klinker, C. C.; Bowser, M. T.	4-fluoro-7-nitro-2,1,3-benzoxadiazole as a fluorogenic labeling reagent for the in vivo analysis of amino acid neurotransmitters using online microdialysis-capillary electrophoresis	2007	79	8747-8754	Anal Chem
Kobayashi, M.; Takeda, Y.; Taninishi, H.; Takata, K.; Aoe, H.; Morita, K.	Quantitative evaluation of the neuroprotective effects of thiopental sodium, propofol, and halothane on brain ischemia in the gerbil: effects of the anesthetics on ischemic depolarization and extracellular glutamate concentration	2007	19	171-178	J Neurosurg Anesthesiol
Kodama, T.; Hikosaka, K.; Honda, Y.; Kojima, T.; Tsutsui, K.; Watanabe, M.	Dopamine and glutamate release in the anterior default system during rest: A monkey microdialysis study	2015	294	194-197	Behav Brain Res
Kodama, T.; Hikosaka, K.; Watanabe, M.	Differential changes in glutamate concentration in the primate prefrontal cortex during spatial delayed alternation and sensory-	2002	145	133-141	Exp Brain Res

Glutamate					
**Authors**	**Title**	**Year**	**Vol.**	**Pages**	**Journal**
	guided tasks				
Kodama, T.; Honda, Y.	Acetylcholine and glutamate release during sleep-wakefulness in the pedunculopontine tegmental nucleus and norepinephrine changes regulated by nitric oxide	1999	53	109-111	Psychiatry Clin Neurosci
Kodama, T.; Kimura, M.	Arousal effects of orexin-A correlate with GLU release from the locus coeruleus in rats	2002	23	1673-1681	Peptides
Kodama, T.; Lai, Y. Y.; Siegel, J. M.	Enhanced glutamate release during REM sleep in the rostromedial medulla as measured by in vivo microdialysis	1998	780	178-181	Brain Res
Koetzner, L.; Hua, X. Y.; Lai, J.; Porreca, F.; Yaksh, T.	Nonopioid actions of intrathecal dynorphin evoke spinal excitatory amino acid and prostaglandin E2 release mediated by cyclooxygenase-1 and -2	2004	24	1451-1458	J Neurosci
Kohno, K.; Ohta, S.; Kohno, K.; Kumon, Y.; Mitani, A.; Sakaki, S.; Kataoka, K.	Nitric oxide synthase inhibitor reduces delayed neuronal death in gerbil hippocampal CA1 neurons after transient global ischemia without reduction of brain temperature or extracellular glutamate concentration	1996	738	257-280	Brain Res
Kohno, T.; Asai, S.; Iribe, Y.; Hosoi, I.; Shibata, K.; Ishikawa, K.	An improved method for the detection of changes in brain extracellular glutamate levels	1998	81	199-205	J Neurosci Methods
Koinig, H.; Morimoto, Y.; Zornow, M. H.	The combination of lamotrigine and mild hypothermia prevents ischemia-induced increase in hippocampal glutamate	2001	13	106-112	J Neurosurg Anesthesiol
Koinig, H.; Vornik, V.; Rueda, C.; Zornow, M. H.	Lubeluzole inhibits accumulation of extracellular glutamate in the hippocampus during transient global cerebral ischemia	2001	898	297-302	Brain Res
Koizumi, H.; Fujisawa, H.; Ito, H.; Maekawa, T.; Di, X.; Bullock, R.	Effects of mild hypothermia on cerebral blood flow-independent changes in cortical extracellular levels of amino acids following contusion trauma in the rat	1997	747	304-312	Brain Res
Koizumi, H.; Fujisawa, H.; Suehiro, E.; Shirao, S.; Suzuki, M.	Neuroprotective effects of ebselen following forebrain ischemia: involvement of glutamate and nitric oxide	2011	51	337-343	Neurol Med Chir (Tokyo)
Kolosowska, K.; Maciejak, P.; Szyndler, J.; Turzynska, D.; Sobolewska, A.; Plaznik, A.	The role of IL-1beta and glutamate in the effects of lipopolysaccharide on the hippocampal electrical kindling of seizures	2016	298	146-152	J Neuroimmunol
Kondrat, R. W.; Kanamori, K.; Ross, B. D.	In vivo microdialysis and gas-chromatography/mass-spectrometry for 13C-enrichment measurement of extracellular glutamate in rat brain	2002	120	179-192	J Neurosci Methods
Konradsson-Geuken, A.; Wu, H. Q.; Gash,	Cortical kynurenic acid bi-directionally modulates prefrontal	2010	169	1848-	Neuroscience

Glutamate					
**Authors**	**Title**	**Year**	**Vol.**	**Pages**	**Journal**
C. R.; Alexander, K. S.; Campbell, A.; Sozeri, Y.; Pellicciari, R.; Schwarcz, R.; Bruno, J. P.	glutamate levels as assessed by microdialysis and rapid electrochemistry			1859	
Kovacs, A.; Mihaly, A.; Komaromi, A.; Gyengesi, E.; Szente, M.; Weiczner, R.; Krisztin-Peva, B.; Szabo, G.; Telegdy, G.	Seizure, neurotransmitter release, and gene expression are closely related in the striatum of 4-aminopyridine-treated rats	2003	55	117-129	Epilepsy Res
Kretschmer, B. D.; Goiny, M.; Herrera-Marschitz, M.	Effect of intracerebral administration of NMDA and AMPA on dopamine and glutamate release in the ventral pallidum and on motor behavior	2000	74	2049-2057	J Neurochem
Kreuter, J. D.; Mattson, B. J.; Wang, B.; You, Z. B.; Hope, B. T.	Cocaine-induced Fos expression in rat striatum is blocked by chloral hydrate or urethane	2004	127	233-242	Neuroscience
Kroppenstedt, S. N.; Sakowitz, O. W.; Thomale, U. W.; Unterberg, A. W.; Stover, J. F.	Influence of norepinephrine and dopamine on cortical perfusion, EEG activity, extracellular glutamate, and brain edema in rats after controlled cortical impact injury	2002	19	1421-1432	J Neurotrauma
Krugel, U.; Schraft, T.; Regenthal, R.; Illes, P.; Kittner, H.	Purinergic modulation of extracellular glutamate levels in the nucleus accumbens in vivo	2004	22	565-570	Int J Dev Neurosci
Kumar, N.; Cherkas, P. S.; Varathan, V.; Miyamoto, M.; Chiang, C. Y.; Dostrovsky, J. O.; Sessle, B. J.; Coderre, T. J.	Systemic pregabalin attenuates facial hypersensitivity and noxious stimulus-evoked release of glutamate in medullary dorsal horn in a rodent model of trigeminal neuropathic pain	2013	62	831-835	Neurochem Int
Kunimatsu, T.; Asai, S.; Kanematsu, K.; Zhao, H.; Kohno, T.; Misaki, T.; Ishikawa, K.	Transient in vivo membrane depolarization and glutamate release before anoxic depolarization in rat striatum	1999	831	273-282	Brain Res
Kuntz, A.; Clement, H. W.; Lehnert, W.; van Calker, D.; Hennighausen, K.; Gerlach, M.; Schulz, E.	Effects of secretin on extracellular amino acid concentrations in rat hippocampus	2004	111	931-939	J Neural Transm (Vienna)
Kuo, J. R.; Lo, C. J.; Chio, C. C.; Chang, C. P.; Lin, M. T.	Resuscitation from experimental traumatic brain injury by agmatine therapy	2007	75	506-514	Resuscitation
Kuo, J. S.; Li, H. T.; Lin, N. N.; Yang, C. S.; Cheng, F. C.	Dorsal facial area of cat medulla; 5-HT2 action on glutamate release in regulating common carotid blood flow	1999	266	137-140	Neurosci Lett
Kurtz, P.; Vargas-Lopes, C.; Madeira, C.; Mello, I.; Panizzutti, R.; Azevedo, L. C.; Bozza, F. A.	Pathophysiology of sepsis-associated brain dysfunction: An experimental study of cerebral microdialysis and mitochondrial function	2013	17	S8-S9	Crit Care
Kusaka, T.; Matsuura, S.; Fujikawa, Y.;	Relationship between cerebral interstitial levels of amino acids	2004	55	273-279	Pediatr Res

Glutamate					
**Authors**	**Title**	**Year**	**Vol.**	**Pages**	**Journal**
Okubo, K.; Kawada, K.; Namba, M.; Okada, H.; Imai, T.; Isobe, K.; Itoh, S.	and phosphorylation potential during secondary energy failure in hypoxic-ischemic newborn piglets				
Kuzmin, A.; Chefer, V.; Bazov, I.; Meis, J.; Ogren, S. O.; Shippenberg, T.; Bakalkin, G.	Upregulated dynorphin opioid peptides mediate alcohol-induced learning and memory impairment	2013	3	e310	Transl Psychiatry
Kwon, J. Y.; Bacher, A.; Zornow, M. H.	Riluzole does not attenuate increases in hippocampal glutamate concentrations in a rabbit model of repeated transient global cerebral ischemia	1998	86	128-133	Anesth Analg
La Bella, V.; Piccoli, F.	Differential effect of beta-N-oxalylamino-L-alanine, the Lathyrus sativus neurotoxin, and (+/-)-alpha-amino-3-hydroxy-5-methylisoxazole-4-propionate on the excitatory amino acid and taurine levels in the brain of freely moving rats	2000	36	523-530	Neurochem Int
LaCrosse, A. L.; Hill, K.; Knackstedt, L. A.	Ceftriaxone attenuates cocaine relapse after abstinence through modulation of nucleus accumbens AMPA subunit expression	2016	26	186-194	Eur Neuropsychopharmacol
Lada, M. W.; Kennedy, R. T.	Quantitative in vivo monitoring of primary amines in rat caudate nucleus using microdialysis coupled by a flow-gated interface to capillary electrophoresis with laser-induced fluorescence detection	1996	68	2790-2797	Anal Chem
Lada, M. W.; Vickroy, T. W.; Kennedy, R. T.	Evidence for neuronal origin and metabotropic receptor-mediated regulation of extracellular glutamate and aspartate in rat striatum in vivo following electrical stimulation of the prefrontal cortex	1998	70	617-625	J Neurochem
Lada, M. W.; Vickroy, T. W.; Kennedy, R. T.	High temporal resolution monitoring of glutamate and aspartate in vivo using microdialysis on-line with capillary electrophoresis with laser-induced fluorescence detection	1997	69	4560-4565	Anal Chem
Lai, Y. J.; Shen, E. Y.; Pan, W. H.	Effects of ascorbate in microdialysis perfusion medium on the extracellular basal concentration of glutamate in rat's striatum	2000	279	145-148	Neurosci Lett
Lallemand, F.; Ward, R. J.; De Witte, P.	The influence of chronic nicotine administration on behavioural and neurochemical parameters in male and female rats after repeated binge drinking exposure	2009	44	535-546	Alcohol Alcohol
Lallemand, F.; Ward, R. J.; De Witte, P.; Verbanck, P.	Binge drinking +/- chronic nicotine administration alters extracellular glutamate and arginine levels in the nucleus accumbens of adult male and female Wistar rats	2011	46	373-382	Alcohol Alcohol
Lallemand, F.; Ward, R. J.; De Witte, P.;	Binge drinking and/or chronic nicotine administration alters	2011	46	i44	Alcohol Alcohol

Glutamate					
**Authors**	**Title**	**Year**	**Vol.**	**Pages**	**Journal**
Verbank, P.	extracellular glutamate and arginine levels in the nucleus accumbens of adult male and female wistar rats				
Lallemand, F.; Ward, R. J.; Dravolina, O.; De Witte, P.	Nicotine-induced changes of glutamate and arginine in naive and chronically alcoholized rats: an in vivo microdialysis study	2006	1111	48-60	Brain Res
Lallement, G.; Carpentier, P.; Collet, A.; Pernot-Marino, I.; Baubichon, D.; Blanchet, G.	Effects of soman-induced seizures on different extracellular amino acid levels and on glutamate uptake in rat hippocampus	1991	563	234-240	Brain Res
Lallement, G.; Carpentier, P.; Collet, A.; Pernot-Marino, I.; Baubichon, D.; Sentenac-Roumanou, H.; Blanchet, G.	[Involvement of glutamatergic system of amygdala in generalized seizures induced by soman: comparison with the hippocampus]	1991	313	421-426	C R Acad Sci III
LaLumiere, R. T.; Kalivas, P. W.	Glutamate release in the nucleus accumbens core is necessary for heroin seeking	2008	28	3170-3177	J Neurosci
Lang, D.; Kiewert, C.; Mdzinarishvili, A.; Schwarzkopf, T. M.; Sumbria, R.; Hartmann, J.; Klein, J.	Neuroprotective effects of bilobalide are accompanied by a reduction of ischemia-induced glutamate release in vivo	2011	1425	155-163	Brain Res
Lang, D.; Schwarzkopf, T.; Kiewert, C.; Mdzinarishvili, A.; Klein, J.	Metabolic changes in the brain after stroke: Neuroprotective effect of bilobalide	2011	383	50	Naunyn-Schmiedeberg's Archives of Pharmacology
Langlais, P. J.; Shu Xing, Zhang	Extracellular glutamate is increased in thalamus during thiamine deficiency-induced lesions and is blocked by MK-801	1993	61	2175-2182	J Neurochem
Langlais, P. J.; Zhang, S. X.; Weilersbacher, G.; Hough, L. B.; Barke, K. E.	Histamine-mediated neuronal death in a rat model of Wernicke's encephalopathy	1994	38	565-574	J Neurosci Res
Largo, C.; Cuevas, P.; Herreras, O.	Is glia disfunction the initial cause of neuronal death in ischemic penumbra?	1996	18	445-448	Neurol Res
Largo, C.; Cuevas, P.; Somjen, G. G.; Martin del Rio, R.; Herreras, O.	The effect of depressing glial function in rat brain in situ on ion homeostasis, synaptic transmission, and neuron survival	1996	16	1219-1229	J Neurosci
Lasley, S. M.; Gilbert, M. E.	Rat hippocampal glutamate and GABA release exhibit biphasic effects as a function of chronic lead exposure level	2002	66	139-47	Toxicol Sci
Lasley, S. M.; Gilbert, M. E.	Presynaptic glutamatergic function in dentate gyrus in vivo is diminished by chronic exposure to inorganic lead	1996	736	125-134	Brain Res
Lasley, S. M.; Green, M. C.; Gilbert, M. E.	Influence of exposure period on in vivo hippocampal glutamate and GABA release in rats chronically exposed to lead	1999	20	619-629	Neurotoxicology
Lasley, S. M.; Yan, Q. S.	Diminished potassium-stimulated GABA release in vivo in	1994	175	145-148	Neurosci Lett

Glutamate					
Authors	Title	Year	Vol.	Pages	Journal
	genetically epilepsy-prone rats				
Lawrence, A. J.	Neurotransmitter mechanisms of rat vagal afferent neurons	1995	22	869-873	Clin Exp Pharmacol Physiol
Lawrence, A. J.; Jarrott, B.	L-glutamate as a neurotransmitter at baroreceptor afferents: evidence from in vivo microdialysis	1994	58	585-591	Neuroscience
Lawrence, A. J.; Jarrott, B.	Nitric oxide increases interstitial excitatory amino acid release in the rat dorsomedial medulla oblongata	1993	151	126-129	Neurosci Lett
Lazarewicz, J. W.; Salinska, E.; Puka, M.	Glycine enhances extracellular 45Ca2+ response to NMDA application investigated with microdialysis of rabbit hippocampus in vivo	1992	52	83-91	Acta Neurobiol Exp (Wars)
Lee, T. F.; Jantzie, L. L.; Todd, K. G.; Cheung, P. Y.	Postresuscitation N-acetylcysteine treatment reduces cerebral hydrogen peroxide in the hypoxic piglet brain	2008	34	190-197	Intensive Care Med
Lehmann, A.	Alterations in hippocampal extracellular amino acids and purine catabolites during limbic seizures induced by folate injections into the rabbit amygdala	1987	22	573-578	Neuroscience
Lehmann, A.	Evidence for a direct action of N-methylaspartate on non-neuronal cells	1987	411	95-101	Brain Res
Lehmann, A.	Effects of microdialysis-perfusion with anisoosmotic media on extracellular amino acids in the rat hippocampus and skeletal muscle	1989	53	525-535	J Neurochem
Lehmann, A.	Abnormalities in the levels of extracellular and tissue amino acids in the brain of the seizure-susceptible rat	1989	3	130-137	Epilepsy Res
Lehmann, A.; Hagberg, H.; Huxtable, R. J.; Sandberg, M.	Reduction of brain taurine: Effects on neurotoxic and metabolic actions of kainate	1987	10	265-274	Neurochem Int
Lekieffre, D.; Callebert, J.; Plotkine, M.; Allix, M.; Boulu, R. G.	Enhancement of endogenous excitatory amino acids by theophylline does not modify the behavioral and histological consequences of forebrain ischemia	1991	565	353-357	Brain Research
Lekieffre, D.; Callebert, J.; Plotkine, M.; Boulu, R. G.	Concomitant increases in the extracellular concentrations of excitatory and inhibitory amino acids in the rat hippocampus during forebrain ischemia	1992	137	78-82	Neurosci Lett
Lekieffre, D.; Ghribi, O.; Callebert, J.; Allix, M.; Plotkine, M.; Boulu, R. G.	Inhibition of glutamate release in rat hippocampus by kynurenic acid does not protect CA1 cells from forebrain ischemia	1992	592	333-337	Brain Research
Lekieffre, D.; Meldrum, B. S.	The pyrimidine-derivative, BW1003C87, protects CA1 and striatal neurons following transient severe forebrain ischaemia in rats. A	1993	56	93-99	Neuroscience

Glutamate					
**Authors**	**Title**	**Year**	**Vol.**	**Pages**	**Journal**
	microdialysis and histological study				
Len, W. B.; Chan, J. Y.	GABAergic neurotransmission at the nucleus tractus solitarii in the suppression of reflex bradycardia by parabrachial nucleus	2001	42	27-39	Synapse
Len, W.; Chan, S. H.; Chan, J. Y.	Parabrachial nucleus induces suppression of baroreflex bradycardia by the release of glutamate in the rostral ventrolateral medulla of the rat	2000	7	401-411	J Biomed Sci
Lena, I.; Chessel, A.; Le Pen, G.; Krebs, M. O.; Garcia, R.	Alterations in prefrontal glutamatergic and noradrenergic systems following MK-801 administration in rats prenatally exposed to methylazoxymethanol at gestational day 17	2007	192	373-383	Psychopharmacology(Berl)
Lena, I.; Parrot, S.; Deschaux, O.; Muffat-Joly, S.; Sauvinet, V.; Renaud, B.; Suaud-Chagny, M. F.; Gottesmann, C.	Variations in extracellular levels of dopamine, noradrenaline, glutamate, and aspartate across the sleep--wake cycle in the medial prefrontal cortex and nucleus accumbens of freely moving rats	2005	81	891-899	J Neurosci Res
Leng, G.; Brown, C. H.; Bull, P. M.; Brown, D.; Scullion, S.; Currie, J.; Blackburn-Munro, R. E.; Feng, J.; Onaka, T.; Verbalis, J. G.; Russell, J. A.; Ludwig, M.	Responses of magnocellular neurons to osmotic stimulation involves coactivation of excitatory and inhibitory input: an experimental and theoretical analysis	2001	21	6967-6977	J Neurosci
Lerma, J.; Herranz, A. S.; Herreras, O.	In vivo determination of extracellular concentration of amino acids in the rat hippocampus. A method based on brain dialysis and computerized analysis	1986	384	145-155	Brain Research
Lesser, J. B.; Koorn, R.; Vloka, J. D.; Kuroda, M. M.; Thys, D. M.	The interaction of temperature with thiopental and etomidate on extracellular dopamine and glutamate levels in Wistar-Kyoto rats subjected to forebrain ischemia	1999	43	989-998	Acta Anaesthesiol Scand
Leung, L. Y.; Tong, K. Y.; Zhang, S. M.; Zeng, X. H.; Zhang, K. P.; Zheng, X. X.	Neurochemical effects of exercise and neuromuscular electrical stimulation on brain after stroke: a microdialysis study using rat model	2006	397	135-139	Neurosci Lett
Levy, F.; Kendrick, K. M.; Goode, J. A.; Guevara-Guzman, R.; Keverne, E. B.	Oxytocin and vasopressin release in the olfactory bulb of parturient ewes: changes with maternal experience and effects on acetylcholine, gamma-aminobutyric acid, glutamate and noradrenaline release	1995	669	197-206	Brain Res
Li, H. T.; Chen, W. Y.; Liu, L.; Yang, C. S.; Cheng, F. C.; Chai, C. Y.; Kuo, J. S.	The dorsal facial area of the medulla in cats: inhibitory action of serotonin on glutamate release in regulating common carotid blood flow	1996	210	193-196	Neurosci Lett

Glutamate					
**Authors**	**Title**	**Year**	**Vol.**	**Pages**	**Journal**
Li, H.; Li, C.; Yan, Z. Y.; Yang, J.; Chen, H.	Simultaneous monitoring multiple neurotransmitters and neuromodulators during cerebral ischemia/reperfusion in rats by microdialysis and capillary electrophoresis	2010	189	162-168	J Neurosci Methods
Li, H.; Yan, Z. Y.	Analysis of amino acid neurotransmitters in hypothalamus of rats during cerebral ischemia-reperfusion by microdialysis and capillary electrophoresis	2010	24	1185-1192	Biomed Chromatogr
Li, J.; Mitchell, J.	Effect of nitric oxide on release of glutamate in the subretrofacial nucleus (SRF) during the exercise pressor reflex in cats	2002	950	195-202	Brain Res
Li, J.; Mitchell, J. H.	Glutamate release in midbrain periaqueductal gray by activation of skeletal muscle receptors and arterial baroreceptors	2003	285	H137-H144	Am J Physiol Heart Circ Physiol
Li, J.; Potts, J. T.; Kramer, G. L.; Petty, F.; Mitchell, J. H.	Activation of skeletal muscle afferents evokes release of glutamate in the subretrofacial nucleus (SRF) of cats	2001	894	249-254	Brain Res
Li, J.; von Pfostl, V.; Zaldivar, D.; Zhang, X.; Logothetis, N.; Rauch, A.	Measuring multiple neurochemicals and related metabolites in blood and brain of the rhesus monkey by using dual microdialysis sampling and capillary hydrophilic interaction chromatography-mass spectrometry	2012	402	2545-2554	Anal Bioanal Chem
Li, J.; Yang, Q.; Hu, S.	The effect of Wuto Granules on the levels of excitatory amino acids in extracellular fluid of rat hippocampus following cerebral ischemia. [Chinese]	1997	22	19-21	Hunan Yi Ke Da Xue Xue Bao
Li, P. A.; He, Q. P.; Miyashita, H.; Howllet, W.; Siesjo, B. K.; Shuaib, A.	Hypothermia ameliorates ischemic brain damage and suppresses the release of extracellular amino acids in both normo- and hyperglycemic subjects	1999	158	242-253	Exp Neurol
Li, P.; Zhang, M. Y.; Wang, D. Q.; Xu, S.; Xu, X. J.	Effects of sinomenine on analgesia and exciting amino acid neurotransmitters in brain of SSNI rat model. [Chinese]	2012	28	1365-1369	Zhongguo yao li xue tong bao
Li, T. F.; Xue, W.; Li, M.; Shi, A. X.; Li, K. X.	Establishment of an LC-MS/MS method to determine content of six amino acids in rat brain dialysate. [Chinese]	2015	24	1659-1664	Zhongguo xin yao za zhi
Li, X. L.; An, Y.; Jin, Q. H.; Kim, M. S.; Park, B. R.; Jin, Y. Z.	Changes of some amino acid concentrations in the medial vestibular nucleus of conscious rats following acute hypotension	2010	447	11-14	Neurosci Lett
Li, X.; Gardner, E. L.; Xi, Z. X.	The metabotropic glutamate receptor 7 (mGluR7) allosteric agonist AMN082 modulates nucleus accumbens GABA and glutamate, but not dopamine, in rats	2008	54	542-551	Neuropharmacology
Li, X.; Li, J.; Gardner, E. L.; Xi, Z. X.	Activation of mGluR7s inhibits cocaine-induced reinstatement of drug-seeking behavior by a nucleus accumbens glutamate-	2010	114	1368-1380	J Neurochem

Glutamate					
Authors	Title	Year	Vol.	Pages	Journal
	mGluR2/3 mechanism in rats				
Li, X.; Li, J.; Peng, X. Q.; Spiller, K.; Gardner, E. L.; Xi, Z. X.	Metabotropic glutamate receptor 7 modulates the rewarding effects of cocaine in rats: involvement of a ventral pallidal GABAergic mechanism	2009	34	1783-1796	Neuropsychopharmacology
Li, Y. F.; Cornish, K. G.; Patel, K. P.	Alteration of NMDA NR1 receptors within the paraventricular nucleus of hypothalamus in rats with heart failure	2003	93	990-997	Circ Res
Li, Y. M.; Qu, Y.; Vandenbussche, E.; Arckens, L.; Vandesande, F.	Analysis of extracellular gamma-aminobutyric acid, glutamate and aspartate in cat visual cortex by in vivo microdialysis and capillary electrophoresis-laser induced fluorescence detection	2001	105	211-215	J Neurosci Methods
Li, Z. P.; Zhang, X. Y.; Lu, X.; Zhong, M. K.; Ji, Y. H.	Dynamic release of amino acid transmitters induced by valproate in PTZ-kindled epileptic rat hippocampus	2004	44	263-270	Neurochem Int
Li, Z. Q.; Yamamoto, Y.; Morimoto, T.; Ono, J.; Okada, S.; Yamatodani, A.	The effect of pentylenetetrazole-kindling on the extracellular glutamate and taurine levels in the frontal cortex of rats	2000	282	117-119	Neurosci Lett
Li, Z.; Boules, M.; Williams, K.; Peris, J.; Richelson, E.	The novel neurotensin analog NT69L blocks phencyclidine (PCP)-induced increases in locomotor activity and PCP-induced increases in monoamine and amino acids levels in the medial prefrontal cortex	2010	1311	28-36	Brain Res
Li, Z.; Boules, M.; Williams, K.; Peris, J.; Richelson, E.	The novel neurotensin analog NT69L blocks phencyclidine-induced increases in locomotor activity and increases in monoamine and amino acids levels in the mPFC	2009	1	71S-72S	Biol Psychiatry
Li, Z.; Gao, L.; Liu, Q.; Cao, C.; Sun, X. L.; Ding, J. H.; Hu, G.	Aquaporin-4 knockout regulated cocaine-induced behavior and neurochemical changes in mice	2006	403	294-248	Neurosci Lett
Li, Z.; Zharikova, A.; Vaughan, C. H.; Bastian, J.; Zandy, S.; Esperon, L.; Axman, E.; Rowland, N. E.; Peris, J.	Intermittent high-dose ethanol exposures increase motivation for operant ethanol self-administration: possible neurochemical mechanism	2010	1310	142-153	Brain Res
Liang, M. Y.; Chen, G. X.; Tang, Z. X.; Rong, J.; Yao, J. P.; Wu, Z. K.	Retrograde Cerebral Perfusion Results in Better Perfusion to the Striatum Than the Cerebral Cortex During Deep Hypothermic Circulatory Arrest: A Microdialysis Study	2016	40	270-277	Artif Organs
Lillaney, R.; Maher, T. J.; Chaiyakul, P.; Ally, A.	Changes in extracellular glutamate and pressor response during muscle contraction following AMPA-receptor blockade in the RVLM and CVLM	1999	844	164-173	Brain Res
Lillrank, S. M.; Connor, W. T.; Oja, S. S.; Ungerstedt, U.	Systemic phencyclidine administration is associated with increased dopamine, GABA, and 5-HIAA levels in the	1994	95	145-155	J Neural Transm Gen Sect

Glutamate					
**Authors**	**Title**	**Year**	**Vol.**	**Pages**	**Journal**
	dorsolateral striatum of conscious rats: an in vivo microdialysis study				
Lin, B.; Globus, M. Y.; Dietrich, W. D.; Busto, R.; Martinez, E.; Ginsberg, M. D.	Differing neurochemical and morphological sequelae of global ischemia: comparison of single- and multiple-insult paradigms	1992	59	2213-2223	J Neurochem
Lin, H. C.; Kang, B. H.; Wan, F. J.; Huang, S. T.; Tseng, C. J.	Reciprocal regulation of nitric oxide and glutamate in the nucleus tractus solitarii of rats	2000	407	83-89	Eur J Pharmacol
Lin, H. C.; Wan, F. J.; Kang, B. H.; Wu, C. C.; Tseng, C. J.	Systemic administration of lipopolysaccharide induces release of nitric oxide and glutamate and c-fos expression in the nucleus tractus solitarii of rats	1999	33	1218-1224	Hypertension
Lin, W. H.; Huang, H. P.; Lin, M. X.; Chen, S. G.	[The effect of high frequency stimulation of epileptic foci on the release of glutamate and gamma-aminobutyric acid in hippocampus of the kainic acid-kindled rats]	2011	27	88-92	Zhongguo Ying Yong Sheng Li Xue Za Zhi
Lindefors, N.; Ungerstedt, U.	Bilateral regulation of glutamate tissue and extracellular levels in caudate-putamen by midbrain dopamine neurons	1990	115	248-252	Neurosci Lett
Lindekens, H.; Smolders, I.; Khan, G. M.; Bialer, M.; Ebinger, G.; Michotte, Y.	In vivo study of the effect of valpromide and valnoctamide in the pilocarpine rat model of focal epilepsy	2000	17	1408-1413	Pharm Res
Linge, R.; Jimenez-Sanchez, L.; Campa, L.; Pilar-Cuellar, F.; Vidal, R.; Pazos, A.; Adell, A.; Diaz, A.	Cannabidiol induces rapid-acting antidepressant-like effects and enhances cortical 5-HT/glutamate neurotransmission: role of 5-HT1A receptors	2016	103	16-26	Neuropharmacology
Liu, H. F.; Zhou, W. H.; Xie, X. H.; Cao, J. L.; Gu, J.; Yang, G. D.	[Muscarinic receptors modulate the mRNA expression of NMDA receptors in brainstem and the release of glutamate in periaqueductal grey during morphine withdrawal in rats]	2004	56	95-100	Sheng Li Xue Bao
Liu, H. G.; Yang, A. C.; Meng, D. W.; Chen, N.; Zhang, J. G.	Stimulation of the anterior nucleus of the thalamus induces changes in amino acids in the hippocampi of epileptic rats	2012	1477	37-44	Brain Res
Liu, H.; Yao, S.	Thiopental sodium reduces glutamate extracellular levels in rat intact prefrontal cortex	2005	167	666-669	Exp Brain Res
Liu, N.; Ho, I. K.; Rockhold, R. W.	Contribution of glutamatergic systems in locus coeruleus to nucleus paragigantocellularis stimulation-evoked behavior	1999	63	555-567	Pharmacol Biochem Behav
Liu, P.; He, X. R.; Zhou, W. B.; Shen, R. R.; Feng, F.	[Research for dependablity of administration of platycodi radix in Tianwang Buxinwan decoction with change of brain inhibitive neurotransmitte in rats by microdialysis]	2008	33	2830-2833	Zhongguo Zhong Yao Za Zhi
Liu, P.; He, X.; Guo, M.	[Single and combining effects of Calculus Bovis and zolpidem on inhibitive neurotransmitter of rat striatum corpora]	2010	35	904-907	Zhongguo Zhong Yao Za Zhi

Glutamate					
**Authors**	**Title**	**Year**	**Vol.**	**Pages**	**Journal**
Liu, Q. F.; Jin, Q. H.; Jiang, H. Y.; Cui, G. Y.; Sun, J. B.; Jin, X. J.	Changes of amino acids in hippocampal dentate gyrus during learning-dependent long-term potentiation in free-moving conscious rats. [Chinese]	2006	10	71-75	Zhongguo kang fu yi xue hui
Liu, Q.; Li, Z.; Ding, J. H.; Liu, S. Y.; Wu, J.; Hu, G.	Iptakalim inhibits nicotine-induced enhancement of extracellular dopamine and glutamate levels in the nucleus accumbens of rats	2006	1085	138-143	Brain Res
Liu, Y. X.; Zhang, M.; Liu, L. Z.; Cui, X.; Hu, Y. Y.; Li, W. B.	The role of glutamate transporter-1a in the induction of brain ischemic tolerance in rats	2012	60	112-124	Glia
Liu, Z.; Zhang, L.; He, Q.; Liu, X.; Chukwunweike Ikechukwu, O.; Tong, L.; Guo, L.; Yang, H.; Zhang, Q.; Zhao, H.; Gu, X.	Effect of Baicalin-loaded PEGylated cationic solid lipid nanoparticles modified by OX26 antibody on regulating the levels of baicalin and amino acids during cerebral ischemia-reperfusion in rats	2015	489	131-138	Int J Pharm
Lo, E. H.; Bosque-Hamilton, P.; Meng, W.	Inhibition of poly(ADP-ribose) polymerase: reduction of ischemic injury and attenuation of N-methyl-D-aspartate-induced neurotransmitter dysregulation	1998	29	830-836	Stroke
Lo, E. H.; Steinberg, G. K.; Panahian, N.; Maidment, N. T.; Newcomb, R.	Profiles of extracellular amino acid changes in focal cerebral ischaemia: effects of mild hypothermia	1993	15	281-287	Neurol Res
Loeliger, M.; Watson, C. S.; Reynolds, J. D.; Penning, D. H.; Harding, R.; Bocking, A. D.; Rees, S. M.	Extracellular glutamate levels and neuropathology in cerebral white matter following repeated umbilical cord occlusion in the near term fetal sheep	2003	116	705-714	Neuroscience
Loidl, C. F.; Herrera-Marschitz, M.; Andersson, K.; You, Z. B.; Goiny, M.; Connor, W. T.; Silveira, R.; Rawal, R.; Bjelke, B.; Chen, Y.; et al.	Long-term effects of perinatal asphyxia on basal ganglia neurotransmitter systems studied with microdialysis in rat	1994	175	9-12	Neurosci Lett
Lombardi, G.; Moroni, F.	GM1 ganglioside reduces ischemia-induced excitatory amino acid output: a microdialysis study in the gerbil hippocampus	1992	134	171-174	Neurosci Lett
Lominac, K. D.; Quadir, S. G.; Barrett, H. M.; McKenna, C. L.; Schwartz, L. M.; Ruiz, P. N.; Wroten, M. G.; Campbell, R. R.; Miller, B. W.; Holloway, J. J.; Travis, K. O.; Rajasekar, G.; Maliniak, D.; Thompson, A. B.; Urman, L. E.; Kippin, T. E.; Phillips, T. J.; Szumlinski, K. K.	Prefrontal glutamate correlates of methamphetamine sensitization and preference	2016	43	689-702	Eur J Neurosci
Lominac, K. D.; Sacramento, A. D.;	Distinct neurochemical adaptations within the nucleus	2012	37	707-722	Neuropsychopharmacology

Glutamate					
**Authors**	**Title**	**Year**	**Vol.**	**Pages**	**Journal**
Szumlinski, K. K.; Kippin, T. E.	accumbens produced by a history of self-administered vs non-contingently administered intravenous methamphetamine				
Lopez-Gil, X.; Artigas, F.; Adell, A.	Role of different monoamine receptors controlling MK-801-induced release of serotonin and glutamate in the medial prefrontal cortex: relevance for antipsychotic action	2009	12	487-499	Int J Neuropsychopharmacol
Lopez-Gil, X.; Babot, Z.; Amargos-Bosch, M.; Sunol, C.; Artigas, F.; Adell, A.	Clozapine and haloperidol differently suppress the MK-801-increased glutamatergic and serotonergic transmission in the medial prefrontal cortex of the rat	2007	32	2087-2097	Neuropsychopharmacology
Lopez-Perez, S. J.; Morales-Villagran, A.; Ventura-Valenzuela, J.; Medina-Ceja, L.	Short- and long-term changes in extracellular glutamate and acetylcholine concentrations in the rat hippocampus following hypoxia	2012	61	258-265	Neurochem Int
Lorenc-Koci, E.; Golembiowska, K.; Pietraszek, M.; Wardas, J.	Treatment with 1,2,3,4,-tetrahydroisoquinoline affects glutamate release in the striatum but not the binding of [3H]MK-801 to NMDA receptors in the dopaminergic structures of the rat brain	2009	61	789-806	Pharmacol Rep
Lorrain, D. S.; Baccei, C. S.; Bristow, L. J.; Anderson, J. J.; Varney, M. A.	Effects of ketamine and N-methyl-D-aspartate on glutamate and dopamine release in the rat prefrontal cortex: modulation by a group II selective metabotropic glutamate receptor agonist LY379268	2003	117	697-706	Neuroscience
Lorrain, D. S.; Schaffhauser, H.; Campbell, U. C.; Baccei, C. S.; Correa, L. D.; Rowe, B.; Rodriguez, D. E.; Anderson, J. J.; Varney, M. A.; Pinkerton, A. B.; Vernier, J. M.; Bristow, L. J.	Group II mGlu receptor activation suppresses norepinephrine release in the ventral hippocampus and locomotor responses to acute ketamine challenge	2003	28	1622-1632	Neuropsychopharmacology
Lowy, M. T.; Gault, L.; Yamamoto, B. K.	Adrenalectomy attenuates stress-induced elevations in extracellular glutamate concentrations in the hippocampus	1993	61	1957-1960	J Neurochem
Lu, L.; Mamiya, T.; Lu, P.; Toriumi, K.; Mouri, A.; Hiramatsu, M.; Zou, L. B.; Nabeshima, T.	Prenatal exposure to PCP produces behavioral deficits accompanied by the overexpression of GLAST in the prefrontal cortex of postpubertal mice	2011	220	132-139	Behav Brain Res
Lu, Y. M.; Zhang, J. T.; Zhao, F. Q.; Qin, Y. F.	Effects of Ca2+ antagonists on glutamate release and Ca2+ influx in the hippocampus with in vivo intracerebral microdialysis	1991	104	222-226	Br J Pharmacol
Lu, Y.; Zhang, L.; Shou, C.; Li, H.; Zheng, X.	Application of overexcitation model induced by penicillin sodium in the study of inhibitory effect of sedative-hypnotic drugs	2005	43	308-312	Pharm Biol
Luczynski, P.; Moquin, L.; Gratton, A.	Chronic stress alters the dendritic morphology of callosal neurons	2015	18	654-667	Stress

Glutamate					
**Authors**	**Title**	**Year**	**Vol.**	**Pages**	**Journal**
	and the acute glutamate stress response in the rat medial prefrontal cortex				
Ludvig, N.; Mishra, P. K.; Yan, Q. S.; Lasley, S. M.; Burger, R. L.; Jobe, P. C.	The paradoxical effect of NMDA receptor stimulation on electrical activity of the sensorimotor cortex in freely behaving rats: analysis by combined EEG-intracerebral microdialysis	1992	12	87-98	Synapse
Ludvig, N.; Potter, P. E.; Fox, S. E.	Simultaneous single-cell recording and microdialysis within the same brain site in freely behaving rats: a novel neurobiological method	1994	55	31-40	J Neurosci Methods
Luna-Munguia, H.; Meneses, A.; Pena-Ortega, F.; Gaona, A.; Rocha, L.	Effects of hippocampal high-frequency electrical stimulation in memory formation and their association with amino acid tissue content and release in normal rats	2012	22	98-105	Hippocampus
Lupinsky, D.; Moquin, L.; Gratton, A.	Interhemispheric regulation of the rat medial prefrontal cortical glutamate stress response: role of local GABA- and dopamine-sensitive mechanisms	2016	234	1-11	Psychopharmacology
Lupinsky, D.; Moquin, L.; Gratton, A.	Interhemispheric regulation of the rat medial prefrontal cortical glutamate stress response: role of local GABA- and dopamine-sensitive mechanisms	2017	234	353-363	Psychopharmacology(Berl)
Lupinsky, D.; Moquin, L.; Gratton, A.	Interhemispheric regulation of the medial prefrontal cortical glutamate stress response in rats	2010	30	7624-7633	J Neurosci
Lutgen, V.; Resch, J.; Qualmann, K.; Raddatz, N. J.; Panhans, C.; Olander, E. M.; Kong, L.; Choi, S.; Mantsch, J. R.; Baker, D. A.	Behavioral assessment of acute inhibition of system xc (-) in rats	2014	231	4637-4647	Psychopharmacology(Berl)
Mabrouk, O. S.; Marti, M.; Morari, M.	Endogenous nociceptin/orphanin FQ (N/OFQ) contributes to haloperidol-induced changes of nigral amino acid transmission and parkinsonism: a combined microdialysis and behavioral study in naive and nociceptin/orphanin FQ receptor knockout mice	2010	166	40-48	Neuroscience
Mabrouk, O. S.; Marti, M.; Salvadori, S.; Morari, M.	The novel delta opioid receptor agonist UFP-512 dually modulates motor activity in hemiparkinsonian rats via control of the nigro-thalamic pathway	2009	164	360-369	Neuroscience
Mabrouk, O. S.; Mela, F.; Calcagno, M.; Budri, M.; Viaro, R.; Dekundy, A.;	GluN2A and GluN2B NMDA receptor subunits differentially modulate striatal output pathways and contribute to levodopa-	2013	4	808-816	ACS Chem Neurosci

Glutamate					
**Authors**	**Title**	**Year**	**Vol.**	**Pages**	**Journal**
Parsons, C. G.; Auberson, Y. P.; Morari, M.	induced abnormal involuntary movements in dyskinetic rats				
Mabrouk, O. S.; Semaan, D. Z.; Mikelman, S.; Gnegy, M. E.; Kennedy, R. T.	Amphetamine stimulates movement through thalamocortical glutamate release	2014	128	152-161	J Neurochem
Mabrouk, O. S.; Viaro, R.; Volta, M.; Ledonne, A.; Mercuri, N.; Morari, M.	Stimulation of delta opioid receptor and blockade of nociceptin/orphanin FQ receptor synergistically attenuate parkinsonism	2014	34	12953-12962	J Neurosci
Mabrouk, O. S.; Volta, M.; Marti, M.; Morari, M.	Stimulation of delta opioid receptors located in substantia nigra reticulata but not globus pallidus or striatum restores motor activity in 6-hydroxydopamine lesioned rats: new insights into the role of delta receptors in parkinsonism	2008	107	1647-1659	J Neurochem
Maciejak, P.; Lehner, M.; Turzynska, D.; Szyndler, J.; Bidzinski, A.; Taracha, E.; Sobolewska, A.; Walkowiak, J.; Skorzewska, A.; Wislowska, A.; Hamed, A.; Plaznik, A.	The opposite role of hippocampal mGluR1 in fear conditioning in kindled and non-kindled rats	2008	1187	184-193	Brain Res
Maciejak, P.; Szyndler, J.; Turzynska, D.; Sobolewska, A.; Plaznik, A.	Kynurenic acid: A new effector of valproate action?	2011	63	1569-1573	Pharmacological Reports
Maciejak, P.; Szyndler, J.; Turzynska, D.; Sobolewska, A.; Taracha, E.; Skorzewska, A.; Lehner, M.; Bidzinski, A.; Hamed, A.; Wislowska-Stanek, A.; Plaznik, A.	The effects of group III mGluR ligands on pentylenetetrazol-induced kindling of seizures and hippocampal amino acids concentration	2009	1282	20-27	Brain Res
MacKay, K. B.; Patel, T. R.; Galbraith, S. L.; Woodruff, G. N.; McCulloch, J.	The relationship between glutamate release and cerebral blood flow after focal cerebral ischaemia in the cat: effect of pretreatment with enadoline (a kappa receptor agonist)	1996	712	329-334	Brain Res
Madayag, A.; Kau, K. S.; Lobner, D.; Mantsch, J. R.; Wisniewski, S.; Baker, D. A.	Drug-induced plasticity contributing to heightened relapse susceptibility: Neurochemical changes and augmented reinstatement in high-intake rats	2010	30	210-217	J Neurosci
Madayag, A.; Lobner, D.; Kau, K. S.; Mantsch, J. R.; Abdulhameed, O.; Hearing, M.; Grier, M. D.; Baker, D. A.	Repeated N-acetylcysteine administration alters plasticity-dependent effects of cocaine	2007	27	13968-13976	J Neurosci
Maeda, T.	Pre-synaptic release of excitatory amino acid in contused brain tissue: Effects of hypothermia and in situ administration of Co2+	1997	23	293-305	J Brain Sci

Glutamate					
**Authors**	**Title**	**Year**	**Vol.**	**Pages**	**Journal**
	on extracellular levels of glutamate				
Maeda, T.; Katayama, Y.; Kawamata, T.; Yamamoto, T.	Mechanisms of excitatory amino acid release in contused brain tissue: effects of hypothermia and in situ administration of Co2+ on extracellular levels of glutamate	1998	15	655-664	J Neurotrauma
Magnusson, O.; Serrano-Lundkvist, G.; Henriksson, I.; Mohringe, B.; Ogren, S. O.	Prolonged treatment with the atypical antipsychotic agent remoxipride reduces extracellular glutamate levels in the striatum of freely moving rats	1997	81	53-56	Pharm and Toxicol
Mahler, S. V.; Hensley-Simon, M.; Tahsili-Fahadan, P.; LaLumiere, R. T.; Thomas, C.; Fallon, R. V.; Kalivas, P. W.; Aston-Jones, G.	Modafinil attenuates reinstatement of cocaine seeking: role for cystine-glutamate exchange and metabotropic glutamate receptors	2014	19	49-60	Addict Biol
Maione, S.; Marabese, I.; Oliva, P.; De Novellis, V.; Stella, L.; Filippelli, A.; Rossi, F.	Periaqueductal gray matter glutamate and GABA decrease following subcutaneous formalin injection in rat	1999	10	1403-1407	NeuroReport
Maione, S.; Marabese, I.; Rossi, F.; Berrino, L.; Palazzo, E.; Trabace, L.	Effects of persistent nociception on periaqueductal gray glycine release	2000	97	311-316	Neuroscience
Malmberg, A. B.; Hamberger, A.; Hedner, T.	Effects of prostaglandin E2 and capsaicin on behavior and cerebrospinal fluid amino acid concentrations of unanesthetized rats: a microdialysis study	1995	65	2185-2193	J Neurochem
Mannaioni, G.; Carpenedo, R.; Moroni, F.	5-hydroxyindole causes convulsions and increases transmitter release in the CA1 region of the rat hippocampus	2003	138	245-253	Br J Pharmacol
Manto, M.; Laute, M. A.	A possible mechanism for the beneficial effect of ethanol in essential tremor	2008	15	697-705	Eur J Neurol
Marabese, I.; de Novellis, V.; Palazzo, E.; Mariani, L.; Siniscalco, D.; Rodella, L.; Rossi, F.; Maione, S.	Differential roles of mGlu8 receptors in the regulation of glutamate and gamma-aminobutyric acid release at periaqueductal grey level	2005	49 Suppl1	157-166	Neuropharmacology
Margaill, I.; Parmentier, S.; Callebert, J.; Allix, M.; Boulu, R. G.; Plotkine, M.	Short therapeutic window for MK-801 in transient focal cerebral ischemia in normotensive rats	1996	16	107-113	J Cereb Blood Flow Metab
Mark, K. A.; Soghomonian, J. J.; Yamamoto, B. K.	High-dose methamphetamine acutely-activates the striatonigral pathway to increase striatal glutamate and mediate long-term dopamine toxicity	2004	24	11449-11456	J Neurosci
Markou, A.; Amitai, N.; Behrens, M.; Kuczenski, R.	Repeated phencyclidine administration alters glutamate release and decreases gaba markers in the prefrontal cortex of rats	2011	37	109-110	Schizophr Bull

Glutamate					
**Authors**	**Title**	**Year**	**Vol.**	**Pages**	**Journal**
Marquez de Prado, B.; Castaneda, T. R.; Galindo, A.; del Arco, A.; Segovia, G.; Reiter, R. J.; Mora, F.	Melatonin disrupts circadian rhythms of glutamate and GABA in the neostriatum of the aware rat: a microdialysis study	2000	29	209-216	J Pineal Res
Marsala, M.; Sorkin, L. S.; Yaksh, T. L.	Transient spinal ischemia in rat: characterization of spinal cord blood flow, extracellular amino acid release, and concurrent histopathological damage	1994	14	604-614	J Cereb Blood Flow Metab
Marti, M.; Guerrini, R.; Beani, L.; Bianchi, C.; Morari, M.	Nociceptin/orphanin FQ receptors modulate glutamate extracellular levels in the substantia nigra pars reticulata. A microdialysis study in the awake freely moving rat	2002	112	153-160	Neuroscience
Marti, M.; Manzalini, M.; Fantin, M.; Bianchi, C.; Della Corte, L.; Morari, M.	Striatal glutamate release evoked in vivo by NMDA is dependent upon ongoing neuronal activity in the substantia nigra, endogenous striatal substance P and dopamine	2005	93	195-205	J Neurochem
Marti, M.; Mela, F.; Bianchi, C.; Beani, L.; Morari, M.	Striatal dopamine-NMDA receptor interactions in the modulation of glutamate release in the substantia nigra pars reticulata in vivo: opposite role for D1 and D2 receptors	2002	83	635-644	J Neurochem
Marti, M.; Mela, F.; Guerrini, R.; Calo, G.; Bianchi, C.; Morari, M.	Blockade of nociceptin/orphanin FQ transmission in rat substantia nigra reverses haloperidol-induced akinesia and normalizes nigral glutamate release	2004	91	1501-1504	J Neurochem
Marti, M.; Rodi, D.; Li, Q.; Guerrini, R.; Fasano, S.; Morella, I.; Tozzi, A.; Brambilla, R.; Calabresi, P.; Simonato, M.; Bezard, E.; Morari, M.	Nociceptin/orphanin FQ receptor agonists attenuate L-DOPA-induced dyskinesias	2012	32	16106-16119	J Neurosci
Marti, M.; Sbrenna, S.; Fuxe, K.; Bianchi, C.; Beani, L.; Morari, M.	Increased responsivity of glutamate release from the substantia nigra pars reticulata to striatal NMDA receptor blockade in a model of Parkinson's disease. A dual probe microdialysis study in hemiparkinsonian rats	2000	12	1848-1850	Eur J Neurosci
Marti, M.; Trapella, C.; Viaro, R.; Morari, M.	The nociceptin/orphanin FQ receptor antagonist J-113397 and L-DOPA additively attenuate experimental parkinsonism through overinhibition of the nigrothalamic pathway	2007	27	1297-1307	J Neurosci
Martinez-Tica, J. F.; Zornow, M. H.	Effects of adenosine agonists and an antagonist on excitatory transmitter release from the ischemic rabbit hippocampus	2000	872	110-115	Brain Res
Mascarucci, P.; Perego, C.; Terrazzino, S.; De Simoni, M. G.	Glutamate release in the nucleus tractus solitarius induced by peripheral lipopolysaccharide and interleukin-1 beta	1998	86	1285-1290	Neuroscience

Glutamate					
**Authors**	**Title**	**Year**	**Vol.**	**Pages**	**Journal**
Masoero, E.; Frattini, P.; Favalli, L.; Rozza, A.; Scelsi, R.; Govoni, S.	Effect of acute alcohol on ischemia-induced glutamate release and brain damage	2000	22	173-177	Alcohol
Mason, P. A.; Escarciga, R.; Doyle, J. M.; Romano, W. F.; Berger, R. E.; Donnellan, J. P.	Amino acid concentrations in hypothalamic and caudate nuclei during microwave-induced thermal stress: analysis by microdialysis	1997	18	277-283	Bioelectromagnetics
Massie, A.; Cnops, L.; Jacobs, S.; Van Damme, K.; Vandenbussche, E.; Eysel, U. T.; Vandesande, F.; Arckens, L.	Glutamate levels and transport in cat (Felis catus) area 17 during cortical reorganization following binocular retinal lesions	2003	84	1387-1397	J Neurochem
Massie, A.; Cnops, L.; Smolders, I.; Van Damme, K.; Vandenbussche, E.; Vandesande, F.; Eysel, U. T.; Arckens, L.	Extracellular GABA concentrations in area 17 of cat visual cortex during topographic map reorganization following binocular central retinal lesioning	2003	976	100-108	Brain Res
Massieu, L.; Gomez-Roman, N.; Montiel, T.	In vivo potentiation of glutamate-mediated neuronal damage after chronic administration of the glycolysis inhibitor iodoacetate	2000	165	257-267	Exp Neurol
Massieu, L.; Morales-Villagran, A.; Tapia, R.	Accumulation of extracellular glutamate by inhibition of its uptake is not sufficient for inducing neuronal damage: an in vivo microdialysis study	1995	64	2262-2272	J Neurochem
Massieu, L.; Tapia, R.	Glutamate uptake impairment and neuronal damage in young and aged rats in vivo	1997	69	1151-1160	J Neurochem
Mataga, N.; Imamura, K.; Watanabe, Y.	6R-tetrahydrobiopterin perfusion enhances dopamine, serotonin, and glutamate outputs in dialysate from rat striatum and frontal cortex	1991	551	64-71	Brain Res
Matarredona, E. R.; Santiago, M.; Machado, A.; Cano, J.	Lack of involvement of glutamate-induced excitotoxicity in MPP+ toxicity in striatal dopaminergic terminals: possible involvement of ascorbate	1997	121	1038-1044	Br J Pharmacol
Matsumoto, K.; Graf, R.; Rosner, G.; Shimada, N.; Heiss, W. D.	Flow thresholds for extracellular purine catabolite elevation in cat focal ischemia	1992	579	309-314	Brain Res
Matsumoto, K.; Graf, R.; Rosner, G.; Taguchi, J.; Heiss, W. D.	Elevation of neuroactive substances in the cortex of cats during prolonged focal ischemia	1993	13	586-594	J Cereb Blood Flow Metab
Matsumoto, K.; Lo, E. H.; Pierce, A. R.; Halpern, E. F.; Newcomb, R.	Secondary elevation of extracellular neurotransmitter amino acids in the reperfusion phase following focal cerebral ischemia	1996	16	114-124	J Cereb Blood Flow Metab
Matsumoto, K.; Ueda, S.; Hashimoto, T.; Kuriyama, K.	Ischemic neuronal injury in the rat hippocampus following transient forebrain ischemia: evaluation using in vivo microdialysis	1991	543	236-242	Brain Res

Glutamate					
**Authors**	**Title**	**Year**	**Vol.**	**Pages**	**Journal**
Matsumoto, M.; Kanno, M.; Togashi, H.; Ueno, K.; Otani, H.; Mano, Y.; Yoshioka, M.	Involvement of GABAA receptors in the regulation of the prefrontal cortex on dopamine release in the rat dorsolateral striatum	2003	482	177-184	Eur J Pharmacol
Matsumoto, M.; Scheller, M. S.; Zornow, M. H.; Strnat, M. A.	Effect of S-emopamil, nimodipine, and mild hypothermia on hippocampal glutamate concentrations after repeated cerebral ischemia in rabbits	1993	24	1228-1234	Stroke
Matsumoto, M.; Zornow, M. H.; Scheller, M. S.; Strnat, M. A.	AMPA and NMDA receptor antagonists do not decrease hippocampal glutamate concentrations during transient global ischemia	1992	77	764-771	Anesthesiology
Matsumoto, Y.; Aihara, K.; Kamata, T.; Goto, N.	Nizofenone, a neuroprotective drug, suppresses glutamate release and lactate accumulation	1994	262	157-161	Eur J Pharmacol
Matsuo, I.; Hirooka, Y.; Hironaga, K.; Eshima, K.; Shigematsu, H.; Shihara, M.; Sakai, K.; Takeshita, A.	Glutamate release via NO production evoked by NMDA in the NTS enhances hypotension and bradycardia in vivo	2001	280	R1285-R1291	Am J Physiol Regul Integr Comp Physiol
Matsushita, Y.; Shima, K.; Katoh, H.; Nawashiro, H.	The synergistic effect of high K+ and hypoxia on extracellular concentrations of neuroactive amino acid in hippocampus	2000	76	247-249	Acta Neurochirg Suppl
Mauler, F.; Fahrig, T.; Horvath, E.; Jork, R.	Inhibition of evoked glutamate release by the neuroprotective 5-HT(1A) receptor agonist BAY x 3702 in vitro and in vivo	2001	888	150-157	Brain Res
Maya-Vetencourt, J. F.; Baroncelli, L.; Viegi, A.; Tiraboschi, E.; Castren, E.; Cattaneo, A.; Maffei, L.	IGF-1 restores visual cortex plasticity in adult life by reducing local GABA levels	2012	2012	250421	Neural Plast
Mazzuferi, M.; Binaschi, A.; Rodi, D.; Mantovani, S.; Simonato, M.	Induction of B1 bradykinin receptors in the kindled hippocampus increases extracellular glutamate levels: a microdialysis study	2005	135	979-986	Neuroscience
McArdle, P.; Penning, D. H.; Dexter, F.; Reynolds, J. D.	Flumazenil does not affect the increase in rat hippocampal extracellular glutamate concentration produced during thioacetamide-induced hepatic encephalopathy	1996	11	329-342	Metab Brain Dis
McFarland, K.; Lapish, C. C.; Kalivas, P. W.	Prefrontal glutamate release into the core of the nucleus accumbens mediates cocaine-induced reinstatement of drug-seeking behavior	2003	23	3531-3537	J Neurosci
McKee, B. L.; Keyghobadi, M.; Tozier De La Poterie, A. P.; Meshul, C. K.	Lesion of the ventromedial nucleus of the thalamus blocks acute cocaine-induced changes in striatal glutamate	2010	64	445-448	Synapse
McKenzie, J. A.; Watson, C. J.; Rostand, R. D.; German, I.; Witowski, S. R.;	Automated capillary liquid chromatography for simultaneous determination of neuroactive amines and amino acids	2002	962	105-115	J Chromatogr A

Glutamate					
Authors	Title	Year	Vol.	Pages	Journal
Kennedy, R. T.					
Mdzinarishvili, A.; Sumbria, R.; Lang, D.; Klein, J.	Ginkgo extract EGb761 confers neuroprotection by reduction of glutamate release in ischemic brain	2012	15	94-102	J Pharm Pharm Sci
Meana, J. J.; Herrera-Marschitz, M.; Brodin, E.; Hokfelt, T.; Ungerstedt, U.	Simultaneous determination of cholecystokinin, dopamine, glutamate and aspartate in cortex and striatum of the rat using in vivo microdialysis	1991	1	365-373	Amino Acids
Meana, J. J.; Johansson, B.; Herrera-Marschitz, M.; Connor, W. T.; Goiny, M.; Parkinson, F. E.; Fredholm, B. B.; Ungerstedt, U.	Effect of the neurotoxin AF64A on intrinsic and extrinsic neuronal systems of rat neostriatum measured by in vivo microdialysis	1992	596	65-72	Brain Res
Medina-Ceja, L.; Morales-Villagran, A.; Tapia, R.	Action of 4-aminopyridine on extracellular amino acids in hippocampus and entorhinal cortex: a dual microdialysis and electroencehalographic study in awake rats	2000	53	255-262	Brain Res Bull
Medina-Ceja, L.; Pardo-Pena, K.; Morales-Villagran, A.; Ortega-Ibarra, J.; Lopez-Perez, S.	Increase in the extracellular glutamate level during seizures and electrical stimulation determined using a high temporal resolution technique	2015	16	11	BMC Neurosci
Meeusen, R.; Smolders, I.; Sarre, S.; de Meirleir, K.; Keizer, H.; Serneels, M.; Ebinger, G.; Michotte, Y.	Endurance training effects on neurotransmitter release in rat striatum: an in vivo microdialysis study	1997	159	335-341	Acta Physiol Scand
Mehl, M.; Bidmon, H. J.; Hilbig, H.; Zilles, K.; Dringen, R.; Ullrich, V.	Ischemia-induced glutamate release in the dentate gyrus. A microdialysis study in the gerbil	1999	271	187-190	Neurosci Lett
Meinhardt, M. W.; Hansson, A. C.; Perreau-Lenz, S.; Bauder-Wenz, C.; Stahlin, O.; Heilig, M.; Harper, C.; Drescher, K. U.; Spanagel, R.; Sommer, W. H.	Rescue of infralimbic mGluR2 deficit restores control over drug-seeking behavior in alcohol dependence	2013	33	2794-2806	J Neurosci
Mela, F.; Marti, M.; Bido, S.; Cenci, M. A.; Morari, M.	In vivo evidence for a differential contribution of striatal and nigral D1 and D2 receptors to L-DOPA induced dyskinesia and the accompanying surge of nigral amino acid levels	2012	45	573-582	Neurobiol Dis
Melani, A.; Pantoni, L.; Bordoni, F.; Gianfriddo, M.; Bianchi, L.; Vannucchi, M. G.; Bertorelli, R.; Monopoli, A.; Pedata, F.	The selective A2A receptor antagonist SCH 58261 reduces striatal transmitter outflow, turning behavior and ischemic brain damage induced by permanent focal ischemia in the rat	2003	959	243-250	Brain Res
Melani, A.; Pantoni, L.; Corsi, C.; Bianchi,	Striatal outflow of adenosine, excitatory amino acids, gamma-	1999	30	2448-	Stroke

Glutamate					
**Authors**	**Title**	**Year**	**Vol.**	**Pages**	**Journal**
L.; Monopoli, A.; Bertorelli, R.; Pepeu, G.; Pedata, F.	aminobutyric acid, and taurine in awake freely moving rats after middle cerebral artery occlusion: correlations with neurological deficit and histopathological damage			2455	
Meldrum, B. S.; Swan, J. H.; Leach, M. J.; Millan, M. H.; Gwinn, R.; Kadota, K.; Graham, S. H.; Chen, J.; Simon, R. P.	Reduction of glutamate release and protection against ischemic brain damage by BW 1003C87	1992	593	1-6	Brain Res
Melecio, L. M.; Melendez, R. I.	Prefrontal GABA-B receptor regulation of glutamate release, learning, and alcohol drinking	2016	40	197A	Alcohol Clin Exp Res
Melendez, R. I.; Gregory, M. L.; Bardo, M. T.; Kalivas, P. W.	Impoverished rearing environment alters metabotropic glutamate receptor expression and function in the prefrontal cortex	2004	29	1980-1987	Neuropsychopharmacology
Melendez, R. I.; Hicks, M. P.; Cagle, S. S.; Kalivas, P. W.	Ethanol exposure decreases glutamate uptake in the nucleus accumbens	2005	29	326-333	Alcohol Clin Exp Res
Melendez, R. I.; Vuthiganon, J.; Kalivas, P. W.	Regulation of extracellular glutamate in the prefrontal cortex: focus on the cystine glutamate exchanger and group I metabotropic glutamate receptors	2005	314	139-147	J Pharmacol Exp Ther
Melis, M. R.; Succu, S.; Cocco, C.; Caboni, E.; Sanna, F.; Boi, A.; Ferri, G. L.; Argiolas, A.	Oxytocin induces penile erection when injected into the ventral subiculum: role of nitric oxide and glutamic acid	2010	58	1153-1160	Neuropharmacology
Melis, M. R.; Succu, S.; Mascia, M. S.; Cortis, L.; Argiolas, A.	Extracellular excitatory amino acids increase in the paraventricular nucleus of male rats during sexual activity: main role of N-methyl-d-aspartic acid receptors in erectile function	2004	19	2569-2575	Eur J Neurosci
Melon, C.; Chassain, C.; Bielicki, G.; Renou, J. P.; Kerkerian-Le Goff, L.; Salin, P.; Durif, F.	Progressive brain metabolic changes under deep brain stimulation of subthalamic nucleus in parkinsonian rats	2015	132	703-712	J Neurochem
Melone, M.; Vitellaro-Zuccarello, L.; Vallejo-Illarramendi, A.; Perez-Samartin, A.; Matute, C.; Cozzi, A.; Pellegrini-Giampietro, D. E.; Rothstein, J. D.; Conti, F.	The expression of glutamate transporter GLT-1 in the rat cerebral cortex is down-regulated by the antipsychotic drug clozapine	2001	6	380-386	Mol Psychiatry
Mena, F. V.; Baab, P. J.; Zielke, C. L.; Zielke, H. R.	In vivo glutamine hydrolysis in the formation of extracellular glutamate in the injured rat brain	2000	60	632-641	J Neurosci Res
Meng, C. H.; Ding, J. H.; He, H. R.; Yang, Y. L.; Gu, B.; Hu, G.	[Relationship between neurotoxicity of 6-hydroxydopamine and glutamate transport]	2003	38	885-888	Yao Xue Xue Bao

Glutamate					
**Authors**	**Title**	**Year**	**Vol.**	**Pages**	**Journal**
Meng, T.; Yuan, S.; Zheng, Z.; Liu, T.; Lin, L.	Effects of endogenous melatonin on glutamate and GABA rhythms in the striatum of unilateral 6-hydroxydopamine-lesioned rats	2015	286	308-315	Neuroscience
Mereu, G.; Fa, M.; Ferraro, L.; Cagiano, R.; Antonelli, T.; Tattoli, M.; Ghiglieri, V.; Tanganelli, S.; Gessa, G. L.; Cuomo, V.	Prenatal exposure to a cannabinoid agonist produces memory deficits linked to dysfunction in hippocampal long-term potentiation and glutamate release	2003	100	4915-4920	Proc Natl Acad Sci U S A
Meshul, C. K.; Buckman, J. F.; Allen, C.; Riggan, J. P.; Feller, D. J.	Activation of corticostriatal pathway leads to similar morphological changes observed following haloperidol treatment	1996	22	350-361	Synapse
Meshul, C. K.; Emre, N.; Nakamura, C. M.; Allen, C.; Donohue, M. K.; Buckman, J. F.	Time-dependent changes in striatal glutamate synapses following a 6-hydroxydopamine lesion	1999	88	1-16	Neuroscience
Meshul, C. K.; Kamel, D.; Moore, C.; Kay, T. S.; Krentz, L.	Nicotine alters striatal glutamate function and decreases the apomorphine-induced contralateral rotations in 6-OHDA-lesioned rats	2002	175	257-274	Exp Neurol
Messam, C. A.; Greene, J. G.; Greenamyre, J. T.; Robinson, M. B.	Intrastriatal injections of the succinate dehydrogenase inhibitor, malonate, cause a rise in extracellular amino acids that is blocked by MK-801	1995	684	221-224	Brain Res
Meurs, A.; Clinckers, R.; Ebinger, G.; Michotte, Y.; Smolders, I.	Seizure activity and changes in hippocampal extracellular glutamate, GABA, dopamine and serotonin	2008	78	50-59	Epilepsy Res
Meurs, A.; Clinckers, R.; Ebinger, G.; Michotte, Y.; Smolders, I.	Substantia nigra is an anticonvulsant site of action of topiramate in the focal pilocarpine model of limbic seizures	2006	47	1519-1535	Epilepsia
Meurs, A.; Portelli, J.; Balasubramaniam, A.; Clinckers, R.; Ebinger, G.; Michotte, Y.; Smolders, I.	Neuropeptide Y increases extracellular glutamate concentration in the hippocampus	2009	50	182	Epilepsia
Meurs, A.; Portelli, J.; Clinckers, R.; Balasubramaniam, A.; Michotte, Y.; Smolders, I.	Neuropeptide Y increases in vivo hippocampal extracellular glutamate levels through Y1 receptor activation	2012	510	143-147	Neurosci Lett
Meyer, P. J.; Meshul, C. K.; Phillips, T. J.	Ethanol- and cocaine-induced locomotion are genetically related to increases in accumbal dopamine	2009	8	346-355	Genes Brain Behav
Michalak, A.; Rose, C.; Butterworth, J.; Butterworth, R. F.	Neuroactive amino acids and glutamate (NMDA) receptors in frontal cortex of rats with experimental acute liver failure	1996	24	908-913	Hepatology
Miele, M.; Berners, M.; Boutelle, M. G.; Kusakabe, H.; Fillenz, M.	The determination of the extracellular concentration of brain glutamate using quantitative microdialysis	1996	707	131-133	Brain Res

Glutamate					
**Authors**	**Title**	**Year**	**Vol.**	**Pages**	**Journal**
Miele, M.; Boutelle, M. G.; Fillenz, M.	The source of physiologically stimulated glutamate efflux from the striatum of conscious rats	1996	497 (Pt3)	745-751	J Physiol
Miele, M.; Mura, M. A.; Enrico, P.; Esposito, G.; Serra, P. A.; Migheli, R.; Zangani, D.; Miele, E.; Desole, M. S.	On the mechanism of d-amphetamine-induced changes in glutamate, ascorbic acid and uric acid release in the striatum of freely moving rats	2000	129	582-588	Br J Pharmacol
Mignon, L. J.; Wolf, W. A.	8-hydroxy-2-(di-n-propylamino)tetralin reduces striatal glutamate in an animal model of Parkinson's disease	2005	16	699-703	Neuroreport
Miguens, M.; Botreau, F.; Olias, O.; Del Olmo, N.; Coria, S. M.; Higuera-Matas, A.; Ambrosio, E.	Genetic differences in the modulation of accumbal glutamate and gamma-amino butyric acid levels after cocaine-induced reinstatement	2013	18	623-632	Addict Biol
Miguens, M.; Del Olmo, N.; Higuera-Matas, A.; Torres, I.; Garcia-Lecumberri, C.; Ambrosio, E.	Glutamate and aspartate levels in the nucleus accumbens during cocaine self-administration and extinction: a time course microdialysis study	2008	196	303-313	Psychopharmacology(Berl)
Mikami, C.; Suzuki, M.; Tsuiki, K.; Ogawa, A.	Effect of nicardipine and magnesiumon cerebral infarction - brain surface perfusion technique	2001	11	44-50	Cerebrovasc Dis
Mikhailova, M. O.	Comparison of changes in glutamate levels in the nucleus accumbens of the rat brain during food consumption in conditions of blockade of dopamine D1 and D2 receptors	2003	33	431-434	Neurosci Behav Physiol
Mikhailova, M. O.	[Comparison in changes of glutamate level in the rat nucleus accumbens induced by D1- and D2-dopamine receptors during feeding]	2002	88	3-7	Ross Fiziol Zh Im I M Sechenova
Millan, M. H.; Chapman, A. G.; Meldrum, B. S.	Dual inhibitory action of enadoline (CI977) on release of amino acids in the rat hippocampus	1995	279	75-81	Eur J Pharmacol
Millan, M. H.; Chapman, A. G.; Meldrum, B. S.	Contrasting effects of D- and L-(E)-4-(3-phosphono-2-propenyl)piperazine-2-carboxylic acid as anticonvulsants and as inhibitors of potassium-evoked increases in hippocampal extracellular glutamate and aspartate levels in freely moving rats	1994	62	217-222	J Neurochem
Millan, M. H.; Chapman, A. G.; Meldrum, B. S.	Extracellular amino acid levels in hippocampus during pilocarpine-induced seizures	1993	14	139-148	Epilepsy Res
Millan, M. H.; Obrenovitch, T. P.; Sarna, G. S.; Lok, S. Y.; Symon, L.; Meldrum, B. S.	Changes in rat brain extracellular glutamate concentration during seizures induced by systemic picrotoxin or focal bicuculline injection: An in vivo dialysis study with on-line enzymatic detection	1991	9	86-91	Epilepsy Res

Glutamate					
**Authors**	**Title**	**Year**	**Vol.**	**Pages**	**Journal**
Milton, S. L.; Thompson, J. W.; Lutz, P. L.	Mechanisms for maintaining extracellular glutamate levels in the anoxic turtle striatum	2002	282	R1317-R1323	Am J Physiol Regul Integr Comp Physiol
Minamoto, Y.; Itano, T.; Tokuda, M.; Matsui, H.; Janjua, N. A.; Hosokawa, K.; Okada, Y.; Murakami, T. H.; Negi, T.; Hatase, O.	In vivo microdialysis of amino acid neurotransmitters in the hippocampus in amygdaloid kindled rat	1992	573	345-348	Brain Res
Minkeviciene, R.; Ihalainen, J.; Malm, T.; Matilainen, O.; Keksa-Goldsteine, V.; Goldsteins, G.; Iivonen, H.; Leguit, N.; Glennon, J.; Koistinaho, J.; Banerjee, P.; Tanila, H.	Age-related decrease in stimulated glutamate release and vesicular glutamate transporters in APP/PS1 transgenic and wild-type mice	2008	105	584-594	J Neurochem
Miranda, M. I.; Ferreira, G.; Ramirez-Lugo, L.; Bermudez-Rattoni, F.	Glutamatergic activity in the amygdala signals visceral input during taste memory formation	2002	99	11417-11422	Proc Natl Acad Sci U S A
Mitani, A.; Andou, Y.; Kataoka, K.	Selective vulnerability of hippocampal CA1 neurons cannot be explained in terms of an increase in glutamate concentration during ischemia in the gerbil: brain microdialysis study	1992	48	307-313	Neuroscience
Mitani, A.; Andou, Y.; Kataoka, K.	Erratum: Selective vulnerability of hippocampal CA1 neurons cannot be explained in terms of an increase in glutamate concentration during ischemia in the gerbil: Brain microdialysis study (Neuroscience 48, 307-313)	1992	50	251	Neuroscience
Mitani, A.; Andou, Y.; Matsuda, S.; Arai, T.; Sakanaka, M.; Kataoka, K.	Origin of ischemia-induced glutamate efflux in the CA1 field of the gerbil hippocampus: an in vivo brain microdialysis study	1994	63	2152-2164	J Neurochem
Mitani, A.; Imon, H.; Iga, K.; Kubo, H.; Kataoka, K.	Gerbil hippocampal extracellular glutamate and neuronal activity after transient ischemia	1990	25	319-324	Brain Res Bull
Mitani, A.; Kataoka, K.	Critical levels of extracellular glutamate mediating gerbil hippocampal delayed neuronal death during hypothermia: brain microdialysis study	1991	42	661-670	Neuroscience
Mitani, A.; Kubo, H.; Iga, K.; Imon, H.; Kadoya, F.; Kataoka, K.	A new enzymatic cycling technique for glutamate determination in brain microdialysates	1990	54	709-711	J Neurochem
Mitani, A.; Matsuda, S.; Yamamoto, H.; Sakanaka, M.; Kataoka, K.	The role of remaining presynaptic terminals in the hippocampal CA1 after selective neuronal death: ischemia-induced glutamate efflux	1996	91	41-46	Acta Neuropathol
Mitani, A.; Tanaka, K.	Functional changes of glial glutamate transporter GLT-1 during	2003	23	7176-	J Neurosci

Glutamate					
Authors	Title	Year	Vol.	Pages	Journal
	ischemia: an in vivo study in the hippocampal CA1 of normal mice and mutant mice lacking GLT-1			7182	
Mitsuya, K.; Nitta, N.; Suzuki, F.	Persistent zinc depletion in the mossy fiber terminals in the intrahippocampal kainate mouse model of mesial temporal lobe epilepsy	2009	50	1979-1990	Epilepsia
Miyamoto, O.; Sumitani, K.; Nakamura, T.; Yamagami, S.; Miyata, S.; Itano, T.; Negi, T.; Okabe, A.	Clostridium perfringens epsilon toxin causes excessive release of glutamate in the mouse hippocampus	2000	189	109-113	FEMS Microbiol Lett
Miyashita, K.; Abe, H.; Nakajima, T.; Ishikawa, A.; Nishiura-Suzuki, M.; Naritomi, H.; Tanaka, R.; Sawada, T.	Glutamate release in the gerbil hippocampus after middle cerebral artery occlusion	1994	5	945-948	Neuroreport
Miyashita, K.; Nakajima, T.; Ishikawa, A.; Miyatake, T.	An adenosine uptake blocker, propentofylline, reduces glutamate release in gerbil hippocampus following transient forebrain ischemia	1992	17	147-150	Neurochem Res
Mizusawa, A.; Ogawa, H.; Kikuchi, Y.; Hida, W.; Kurosawa, H.; Okabe, S.; Takishima, T.; Shirato, K.	In vivo release of glutamate in nucleus tractus solitarii of the rat during hypoxia	1994	478 (Pt1)	55-66	J Physiol
Mocci, G.; Jimenez-Sanchez, L.; Adell, A.; Cortes, R.; Artigas, F.	Expression of 5-HT2A receptors in prefrontal cortex pyramidal neurons projecting to nucleus accumbens. Potential relevance for atypical antipsychotic action	2014	79	49-58	Neuropharmacology
Moghaddam, B.	Stress preferentially increases extraneuronal levels of excitatory amino acids in the prefrontal cortex: comparison to hippocampus and basal ganglia	1993	60	1650-1657	J Neurochem
Moghaddam, B.; Adams, B.; Verma, A.; Daly, D.	Activation of glutamatergic neurotransmission by ketamine: a novel step in the pathway from NMDA receptor blockade to dopaminergic and cognitive disruptions associated with the prefrontal cortex	1997	17	2921-2927	J Neurosci
Moghaddam, B.; Bolinao, M. L.	Biphasic effect of ethanol on extracellular accumulation of glutamate in the hippocampus and the nucleus accumbens	1994	178	99-102	Neurosci Lett
Moghaddam, B.; Bolinao, M. L.; Stein-Behrens, B.; Sapolsky, R.	Glucocorticoids mediate the stress-induced extracellular accumulation of glutamate	1994	655	251-254	Brain Res
Moghaddam, B.; Bunney, B. S.	Depolarization inactivation of dopamine neurons: terminal release characteristics	1993	14	195-200	Synapse

Glutamate					
**Authors**	**Title**	**Year**	**Vol.**	**Pages**	**Journal**
Moghaddamt, H. F.; Ardestani, M. S.; Saffari, M.; Navidpour, L.; Shafiee, A.; Rahmim, A.	Dopaminergic but not glutamatergic neurotransmission is increased in the striatum after selective cyclooxygenase-2 inhibition in normal and hemiparkinsonian rats	2008	103	293-296	Basic Clin Pharmacol Toxicol
Molchanova, S.; Kbi, P.; Oja, S. S.; Saransaari, P.	Interstitial concentrations of amino acids in the rat striatum during global forebrain ischemia and potassium-evoked spreading depression	2004	29	1519-1527	Neurochem Res
Molchanova, S.; Oja, S. S.; Saransaari, P.	Characteristics of basal taurine release in the rat striatum measured by microdialysis	2004	27	261-268	Amino Acids
Monda, M.; Viggiano, A.; De Luca, V.	An aversive diet as thiamine-free food blocks food-induced release of excitatory amino acids in the accumbens	2003	178	197-203	Acta Physiol Scand
Monda, M.; Viggiano, A.; Sullo, A.; De Luca, V.	Aspartic and glutamic acids increase in the frontal cortex during prostaglandin E1 hyperthermia	1998	83	1239-1243	Neuroscience
Monno, A.; Vezzani, A.; Bastone, A.; Salmona, M.; Garattini, S.	Extracellular glutamate levels in the hypothalamus and hippocampus of rats after acute or chronic oral intake of monosodium glutamate	1995	193	45-48	Neurosci Lett
Montalbetti, L.	Aminoacid recovery via microdialysis and photoinduced focal cerebral ischemia in brain cortex of rats	1995	192	153-156	Neurosci Lett
Mora, G.; Tapia, R.	Effects of retigabine on the neurodegeneration and extracellular glutamate changes induced by 4-aminopyridine in rat hippocampus in vivo	2005	30	1557-1565	Neurochem Res
Morales, I.; Dopico, J. G.; Sabate, M.; Gonzalez-Hernandez, T.; Rodriguez Diaz, M.	Substantia nigra osmoregulation: Taurine and ATP involvement	2007	292	C1934-C1941	Am J Physiol Cell Physiol
Morales, I.; Fuentes, A.; Gonzalez-Hernandez, T.; Rodriguez, M.	Osmosensitive response of glutamate in the substantia nigra	2009	220	335-340	Exp Neurol
Morales, I.; Sabate, M.; Rodriguez, M.	Striatal glutamate induces retrograde excitotoxicity and neuronal degeneration of intralaminar thalamic nuclei: their potential relevance for Parkinson's disease	2013	38	2172-2182	Eur J Neurosci
Morales-Villagran, A.; Pardo-Pena, K.; Medina-Ceja, L.; Lopez-Perez, S.	A microdialysis and enzymatic reactor sensing procedure for the simultaneous registration of online glutamate measurements at high temporal resolution during epileptiform activity	2016	139	886-896	J Neurochem
Morales-Villagran, A.; Tapia, R.	Preferential stimulation of glutamate release by 4-aminopyridine in rat striatum in vivo	1996	28	35-40	Neurochem Int

Glutamate					
**Authors**	**Title**	**Year**	**Vol.**	**Pages**	**Journal**
Moran, R. J.; Stephan, K. E.; Kiebel, S. J.; Rombach, N.; Connor, W. T.; Murphy, K. J.; Reilly, R. B.; Friston, K. J.	Bayesian estimation of synaptic physiology from the spectral responses of neural masses	2008	42	272-284	Neuroimage
Morari, M.; Connor, W. T.; Darvelid, M.; Ungerstedt, U.; Bianchi, C.; Fuxe, K.	Functional neuroanatomy of the nigrostriatal and striatonigral pathways as studied with dual probe microdialysis in the awake rat--I. Effects of perfusion with tetrodotoxin and low-calcium medium	1996	72	79-87	Neuroscience
Morari, M.; Connor, W. T.; Ungerstedt, U.; Bianchi, C.; Fuxe, K.	Functional neuroanatomy of the nigrostriatal and striatonigral pathways as studied with dual probe microdialysis in the awake rat--II. Evidence for striatal N-methyl-D-aspartate receptor regulation of striatonigral GABAergic transmission and motor function	1996	72	89-97	Neuroscience
Morari, M.; Connor, W. T.; Ungerstedt, U.; Fuxe, K.	Dopamine D1 and D2 receptor antagonism differentially modulates stimulation of striatal neurotransmitter levels by N-methyl-D-aspartic acid	1994	256	23-30	Eur J Pharmacol
Morari, M.; Connor, W. T.; Ungerstedt, U.; Fuxe, K.	N-methyl-D-aspartic acid differentially regulates extracellular dopamine, GABA, and glutamate levels in the dorsolateral neostriatum of the halothane-anesthetized rat: an in vivo microdialysis study	1993	60	1884-1893	J Neurochem
Morari, M.; Fantin, M.	Loss of the preferential control over the striato-nigral direct pathway by striatal NMDA receptors in a rat model of Parkinson's disease	2015	140	3830-3839	Analyst
Morari, M.; Sbrenna, S.; Marti, M.; Connor, W. T.; Bianchi, C.; Fuxe, K.; Beani, L.	Evidence for a striatal NMDA receptor modulation of nigral glutamate release. A dual probe microdialysis study in the awake freely moving rat	1998	10	1716-1722	Eur J Neurosci
Morgese, M. G.; Cassano, T.; Gaetani, S.; Macheda, T.; Laconca, L.; Dipasquale, P.; Ferraro, L.; Antonelli, T.; Cuomo, V.; Giuffrida, A.	Neurochemical changes in the striatum of dyskinetic rats after administration of the cannabinoid agonist WIN55,212-2	2009	54	56-64	Neurochem Int
Mori, K.; Maeda, M.; Miyazaki, M.; Iwase, H.	Effects of mild and moderate hypothermia on cerebral metabolism and glutamate in an experimental head injury	1998	71	222-224	Acta Neurochir Suppl
Mori, K.; Maeda, M.; Miyazaki, M.; Iwase, H.	Effects of mild (33 degrees C) and moderate (29 degrees C) hypothermia on cerebral blood flow and metabolism, lactate, and	1998	20	719-726	Neurol Res

Glutamate					
**Authors**	**Title**	**Year**	**Vol.**	**Pages**	**Journal**
	extracellular glutamate in experimental head injury				
Mori, T.; Tateishi, N.; Kagamiishi, Y.; Shimoda, T.; Satoh, S.; Ono, S.; Katsube, N.; Asano, T.	Attenuation of a delayed increase in the extracellular glutamate level in the peri-infarct area following focal cerebral ischemia by a novel agent ONO-2506	2004	45	381-387	Neurochem Int
Morimoto, T.; Globus, M. Y.; Busto, R.; Martinez, E.; Ginsberg, M. D.	Simultaneous measurement of salicylate hydroxylation and glutamate release in the penumbral cortex following transient middle cerebral artery occlusion in rats	1996	16	92-99	J Cereb Blood Flow Metab
Morishima, Y.; Miyakawa, T.; Furuyashiki, T.; Tanaka, Y.; Mizuma, H.; Nakanishi, S.	Enhanced cocaine responsiveness and impaired motor coordination in metabotropic glutamate receptor subtype 2 knockout mice	2005	102	4170-4175	Proc Natl Acad Sci U S A
Morita, T.; Takahashi, M.; Takeuchi, T.; Hikasa, Y.; Ikeda, S.; Sawada, M.; Sato, K.; Shibahara, T.; Shimada, A.	Changes in extracellular neurotransmitters in the cerebrum of familial idiopathic epileptic shetland sheepdogs using an intracerebral microdialysis technique and immunohistochemical study for glutamate metabolism	2005	67	1119-1126	J Vet Med Sci
Mork, A.; Witten, L. M.; Arnt, J.	Effect of sertindole on extracellular dopamine, acetylcholine, and glutamate in the medial prefrontal cortex of conscious rats: a comparison with risperidone and exploration of mechanisms involved	2009	206	39-49	Psychopharmacology(Berl)
Moroni, F.; Carpenedo, R.; Cozzi, A.; Meli, E.; Chiarugi, A.; Pellegrini-Giampietro, D. E.	Studies on the neuroprotective action of kynurenine mono-oxygenase inhibitors in post-ischemic brain damage	2003	527	127-136	Adv Exp Med Biol
Moroni, F.; Cozzi, A.; Carpendo, R.; Cipriani, G.; Veneroni, O.; Izzo, E.	Kynurenine 3-mono-oxygenase inhibitors reduce glutamate concentration in the extracellular spaces of the basal ganglia but not in those of the cortex or hippocampus	2005	48	788-795	Neuropharmacology
Moroni, F.; Cozzi, A.; Lombardi, G.; Sourtcheva, S.; Leonardi, P.; Carfi, M.; Pellicciari, R.	Presynaptic mGlu1 type receptors potentiate transmitter output in the rat cortex	1998	347	189-195	Eur J Pharmacol
Morrone, L. A.; Rombola, L.; Pelle, C.; Corasaniti, M. T.; Zappettini, S.; Paudice, P.; Bonanno, G.; Bagetta, G.	The essential oil of bergamot enhances the levels of amino acid neurotransmitters in the hippocampus of rat: implication of monoterpene hydrocarbons	2007	55	255-262	Pharmacol Res
Mosqueda-Garcia, R.; Tseng, C. J.; Appalsamy, M.; Beck, C.; Robertson, D.	Cardiovascular excitatory effects of adenosine in the nucleus of the solitary tract	1991	18	494-502	Hypertension
Mousavi, Z.; Shafaghi, B.; Kobarfard, F.;	Sex differences and role of gonadal hormones on glutamate level	2007	554	145-149	Eur J Pharmacol

Glutamate					
**Authors**	**Title**	**Year**	**Vol.**	**Pages**	**Journal**
Jorjani, M.	in the nucleus accumbens in morphine tolerant rats: a microdialysis study				
Munoz, M. D.; Herreras, O.; Herranz, A. S.	Effects of dihydrokainic acid of extracellular amino acids and neuronal excitability in the in vivo rat hippocampus	1987	26	1-8	Neuropharmacology
Mura, E.; Zappettini, S.; Preda, S.; Biundo, F.; Lanni, C.; Grilli, M.; Cavallero, A.; Olivero, G.; Salamone, A.; Govoni, S.; Marchi, M.	Dual effect of beta-amyloid on alpha7 and alpha4beta2 nicotinic receptors controlling the release of glutamate, aspartate and GABA in rat hippocampus	2012	7	e29661	PLoS One
Murakami, G.; Nakamura, M.; Takita, M.; Ishida, Y.; Ueki, T.; Nakahara, D.	Brain Rewarding Stimulation Reduces Extracellular Glutamate Through Glial Modulation in Medial Prefrontal Cortex of Rats	2015	40	2686-2695	Neuropsychopharmacology
Murata, J.; Ikeda, M.; Takazawa, A.; Kaneko, T.; Suzuki, H.	[Differential effects of ketamine enantiomers on anesthetic levels and glutamate release in the hippocampus]	2000	49	255-262	Masui
Murphy, K. J.; Ter Horst, J. P.; Cassidy, A. W.; DeSouza, I. E.; Morgunova, M.; Li, C.; Connole, L. M.; Sullivan, N. C.; Loscher, J. S.; Brady, A. T.; Rombach, N.; Connellan, J.; McGettigan, P. A.; Scully, D.; Fedriani, R.; Lukasz, B.; Moran, M. P.; McCabe, O. M.; Wantuch, C. M.; Hughes, Z. A.; Mulvany, S. K.; Higgins, D. G.; Pangalos, M. N.; Marquis, K. L.; Connor, W. T.; Ring, R. H.; von Schack, D.; Regan, C. M.	Temporal dysregulation of cortical gene expression in the isolation reared Wistar rat	2010	113	601-164	J Neurochem
Murphy, N. P.; Maidment, N. T.	Orphanin FQ/nociceptin modulation of mesolimbic dopamine transmission determined by microdialysis	1999	73	179-186	J Neurochem
Muschamp, J. W.; Regina, M. J.; Hull, E. M.; Winter, J. C.; Rabin, R. A.	Lysergic acid diethylamide and [-]-2,5-dimethoxy-4-methylamphetamine increase extracellular glutamate in rat prefrontal cortex	2004	1023	134-140	Brain Res
Naderi, A.; Asgari, A. R.; Zahed, R.; Ghanbari, A.; Samandari, R.; Jorjani, M.	Estradiol attenuates spinal cord injury-related central pain by decreasing glutamate levels in thalamic VPL nucleus in male rats	2014	29	763-770	Metal Brain Dis
Nagai, T.; Takata, N.; Shinohara, Y.; Hirase, H.	Adaptive changes of extracellular amino acid concentrations in mouse dorsal striatum by 4-AP-induced cortical seizures	2015	295	229-236	Neuroscience
Nagy, K.; Marko, B.; Zsilla, G.; Matyus, P.; Pallagi, K.; Szabo, G.; Juranyi, Z.;	Alterations in brain extracellular dopamine and glycine levels following combined administration of the glycine transporter type-	2010	35	2096-2106	Neurochem Res

Glutamate					
Authors	Title	Year	Vol.	Pages	Journal
Barkoczy, J.; Levay, G.; Harsing Jr, L. G.	1 inhibitor Org-24461 and risperidone				
Nail-Boucherie, K.; Le-Pham, B. T.; Gobaille, S.; Maitre, M.; Aunis, D.; Depaulis, A.	Evidence for a role of the parafascicular nucleus of the thalamus in the control of epileptic seizures by the superior colliculus	2005	46	141-145	Epilepsia
Nakamura, T.; Miyamoto, O.; Kawai, N.; Negi, T.; Itano, T.; Nagao, S.	Long-term activation of the glutamatergic system associated with N-methyl-D-aspartate receptors after postischemic hypothermia in gerbils	2001	49	706-714	Neurosurgery
Nakane, H.; Ooboshi, H.; Ibayashi, S.; Yao, H.; Sadoshima, S.; Fujishima, M.	Isradipine, a calcium channel blocker, attenuates the ischemia-induced release of dopamine but not glutamate in rats	1995	188	151-154	Neurosci Lett
Nakane, H.; Yao, H.; Ibayashi, S.; Kitazono, T.; Ooboshi, H.; Uchimura, H.; Fujishima, M.	Protein kinase C modulates ischemia-induced amino acids release in the striatum of hypertensive rats	1998	782	290-296	Brain Res
Nakasa, H.; Tada, T.; Eguchi, T.; Hirabayashi, H.; Morimoto, T.; Sakaki, T.	Sequential changes in content of excitatory amino acids in the epileptic focus during seizure. [Japanese]	1991	43	451-454	Brain and Nerve
Nakase, H.; Tada, T.; Eguchi, T.; Hirabayashi, H.; Mirimoto, T.; Sakaki, T.	Kindling seizure induction and excitatory amino acid	1992	46	357-360	Jpn J Psychiatry Neurol
Nakase, H.; Tada, T.; Hashimoto, H.; Kurokawa, S.; Hirabayashi, H.; Hoshida, T.; Sakaki, T.; Ohnishi, H.	Experimental study of the mechanism of seizure induction: changes in the concentrations of excitatory amino acids in the epileptic focus of the cat amygdaloid kindling model	1994	64	418-422	Neurol Med Chir (Tokyo)
Nakashima, K.; Todd, M. M.	Effects of hypothermia, pentobarbital, and isoflurane on postdepolarization amino acid release during complete global cerebral ischemia	1996	85	161-168	Anesthesiology
Nakashima, K.; Todd, M. M.	Effects of hypothermia on the rate of excitatory amino acid release after ischemic depolarization	1996	27	913-918	Stroke
Nakata, N.; Kato, H.; Kogure, K.	Ischemic tolerance and extracellular amino acid concentrations in gerbil hippocampus measured by intracerebral microdialysis	1994	35	247-251	Brain Res Bull
Nakata, N.; Kato, H.; Kogure, K.	Protective effects of serotonin reuptake inhibitors, citalopram and clomipramine, against hippocampal CA1 neuronal damage following transient ischemia in the gerbil	1992	590	48-52	Brain Res
Nakata, N.; Kato, H.; Kogure, K.; DeGraba, T. J.	Effects of repeated cerebral ischemia on extracellular amino acid concentrations measured with intracerebral microdialysis in the gerbil hippocampus	1993	24	458-464	Stroke
Nakayama, R.; Yano, T.; Ushijima, K.;	Effects of dantrolene on extracellular glutamate concentration	2002	96	705-710	Anesthesiology

Glutamate					
**Authors**	**Title**	**Year**	**Vol.**	**Pages**	**Journal**
Abe, E.; Terasaki, H.	and neuronal death in the rat hippocampal CA1 region subjected to transient ischemia				
Nam, H. W.; Lee, M. R.; Hinton, D. J.; Choi, D. S.	Reduced effect of NMDA glutamate receptor antagonist on ethanol-induced ataxia and striatal glutamate levels in mice lacking ENT1	2010	479	277-281	Neurosci Lett
Nanri, K.; Takizawa, S.; Fujita, H.; Ogawa, S.; Shinohara, Y.	Modulation of extracellular glutamate concentration by nitric oxide synthase inhibitor in rat transient forebrain ischemia	1996	738	243-248	Brain Res
Nash, J. F.; Yamamoto, B. K.	Methamphetamine neurotoxicity and striatal glutamate release: comparison to 3,4-methylenedioxymethamphetamine	1992	581	237-243	Brain Res
Nash, J. F.; Yamamoto, B. K.	Effect of D-amphetamine on the extracellular concentrations of glutamate and dopamine in iprindole-treated rats	1993	627	1-8	Brain Res
Natividad, L. A.; Buczynski, M. W.; Parsons, L. H.; Torres, O. V.; Dell, L. E.	Adolescent rats are resistant to adaptations in excitatory and inhibitory mechanisms that modulate mesolimbic dopamine during nicotine withdrawal	2012	123	578-588	J Neurochem
Neal-Perry, G. S.; Zeevalk, G. D.; Santoro, N. F.; Etgen, A. M.	Attenuation of preoptic area glutamate release correlates with reduced luteinizing hormone secretion in middle-aged female rats	2005	146	4331-4339	Endocrinology
Neal-Perry, G. S.; Zeevalk, G. D.; Shu, J.; Etgen, A. M.	Restoration of the luteinizing hormone surge in middle-aged female rats by altering the balance of GABA and glutamate transmission in the medial preoptic area	2008	79	878-888	Biol Reprod
Neill, M. J.; Bogaert, L.; Hicks, C. A.; Bond, A.; Ward, M. A.; Ebinger, G.; Ornstein, P. L.; Michotte, Y.; Lodge, D.	LY377770, a novel iGlu5 kainate receptor antagonist with neuroprotective effects in global and focal cerebral ischaemia	2000	39	1575-1588	Neuropharmacology
Nencioni, A. L. A.; Barreto, S. A.; Lebrun, I.; Florio, J. C.; Lourenco, G. A.; Dorce, V. A. C.	Neurotransmitter evaluation in the hippocampus of rats after intracerebral injection of TsTX scorpion toxin	2009	15	236-254	J Venom Anim Toxins Incl Trop Dis
Nencioni, A. L.; Lebrun, I.; Dorce, V. A.	A microdialysis study of glutamate concentration in the hippocampus of rats after TsTX toxin injection and blockade of toxin effects by glutamate receptor antagonists	2003	74	455-463	Phamarcol Biochem Behav
Nencioni, A. L.; Lourenco, G. A.; Lebrun, I.; Florio, J. C.; Dorce, V. A.	Central effects of Tityus serrulatus and Tityus bahiensis scorpion venoms after intraperitoneal injection in rats	2009	463	234-238	Neurosci Lett
Neumann, I.; Landgraf, R.; Bauce, L.; Pittman, Q. J.	Osmotic responsiveness and cross talk involving oxytocin, but not vasopressin or amino acids, between the supraoptic nuclei in virgin and lactating rats	1995	15	3408-3417	J Neurosci

Glutamate					
**Authors**	**Title**	**Year**	**Vol.**	**Pages**	**Journal**
Newcomb, R.; Palma, A.	Effects of diverse omega-conopeptides on the in vivo release of glutamic and gamma-aminobutyric acids	1994	638	95-102	Brain Res
Newcomb, R.; Pierce, A. R.; Kano, T.; Meng, W.; Bosque-Hamilton, P.; Taylor, L.; Curthoys, N.; Lo, E. H.	Characterization of mitochondrial glutaminase and amino acids at prolonged times after experimental focal cerebral ischemia	1998	813	103-111	Brain Res
Nicniocaill, B.; Haraldsson, B.; Hansson, O.; Connor, W. T.; Brundin, P.	Altered striatal amino acid neurotransmitter release monitored using microdialysis in R6/1 Huntington transgenic mice	2001	13	206-210	Eur J Neurosci
Nicol, A. U.; Sanchez-Andrade, G.; Collado, P.; Segonds-Pichon, A.; Kendrick, K. M.	Olfactory bulb encoding during learning under anesthesia	2014	8	193	Front Behav Neurosci
Nielsen, T. H.; Engell, S. I.; Johnsen, R. A.; Schulz, M. K.; Gerke, O.; Hjelmborg, J.; Toft, P.; Nordstrom, C. H.	Comparison between cerebral tissue oxygen tension and energy metabolism in experimental subdural hematoma	2011	15	585-592	Neurocrit Care
Nielsen, T. H.; Olsen, N. V.; Toft, P.; Nordstrom, C. H.	Cerebral energy metabolism during mitochondrial dysfunction induced by cyanide in piglets	2013	57	793-801	Acta Anaesthesiol Scand
Nikiforuk, A.; Golembiowska, K.; Popik, P.	Mazindol attenuates ketamine-induced cognitive deficit in the attentional set shifting task in rats	2010	20	37-48	Eur Neuropsychopharmacol
Nikiforuk, A.; Popik, P.; Drescher, K. U.; van Gaalen, M.; Relo, A. L.; Mezler, M.; Marek, G.; Schoemaker, H.; Gross, G.; Bespalov, A.	Effects of a positive allosteric modulator of group II metabotropic glutamate receptors, LY487379, on cognitive flexibility and impulsive-like responding in rats	2010	335	665-673	J Pharmacol Exp Ther
Nilsson, G. E.; Lutz, P. L.	Release of inhibitory neurotransmitters in response to anoxia in turtle brain	1991	261	R32-R37	Am J Physiol
Nilsson, P.; Hillered, L.; Ponten, U.; Ungerstedt, U.	Changes in cortical extracellular levels of energy-related metabolites and amino acids following concussive brain injury in rats	1990	10	631-637	J Cereb Blood Flow Metab
Nilsson, P.; Ronne-Engstrom, E.; Flink, R.; Ungerstedt, U.; Carlson, H.; Hillered, L.	Epileptic seizure activity in the acute phase following cortical impact trauma in rat	1994	637	227-232	Brain Res
Nitz, D.; Siegel, J.	GABA release in the dorsal raphe nucleus: role in the control of REM sleep	1997	273	R451-R455	Am J Physiol
Nitz, D.; Siegel, J. M.	GABA release in the locus coeruleus as a function of sleep/wake state	1997	78	795-801	Neuroscience

Glutamate					
**Authors**	**Title**	**Year**	**Vol.**	**Pages**	**Journal**
Nitz, D.; Siegel, J. M.	GABA release in posterior hypothalamus across sleep-wake cycle	1996	271	R1707-R1712	Am J Physiol
Nordmark, J.; Enblad, P.; Rubertsson, S.	Cerebral energy failure following experimental cardiac arrest Hypothermia treatment reduces secondary lactate/pyruvate-ratio increase	2009	80	573-579	Resuscitation
Northcott, C. A.; Burnett, R.; MacDonald, M.; Haywood, J. R.; Parsell, D.	GABA, glutamate and taurine in the paraventricular nucleus (PVN) during the onset of renal wrap hypertension	2010	24	/	FASEB J Conference: Experimental Biology
Northrop, N. A.; Smith, L. P.; Yamamoto, B. K.; Eyerman, D. J.	Regulation of glutamate release by alpha7 nicotinic receptors: differential role in methamphetamine-induced damage to dopaminergic and serotonergic terminals	2011	336	900-907	J Pharmacol Exp Ther
Noworyta-Sokolowska, K.; Gorska, A.; Golembiowska, K.	LPS-induced oxidative stress and inflammatory reaction in the rat striatum	2013	65	863-869	Pharmacological Reports
Nyitrai, G.; Emri, Z.; Crunelli, V.; Kekesi, K. A.; Dobolyi, A.; Juhasz, G.	In vivo blockade of thalamic GABA(B) receptors increases excitatory amino-acid levels	1996	318	295-300	Eur J Pharmacol
Nyitrai, G.; Kekesi, K. A.; Szilagyi, N.; Papp, A.; Juhasz, G.; Kardos, J.	Neurotoxicity of lindane and picrotoxin: neurochemical and electrophysiological correlates in the rat hippocampus in vivo	2002	27	139-145	Neurochem Res
Nyitrai, G.; Kovacs, I.; Szarics, E.; Skuban, N.; Juhasz, G.; Kardos, J.	Role of intracellular Ca(2+) stores shaping normal activity in brain	1999	57	906-915	J Neurosci Res
Nyitrai, G.; Szarics, E.; Kovacs, I.; Kekesi, K. A.; Juhasz, G.; Kardos, J.	Effect of CGP 36742 on the extracellular level of neurotransmitter amino acids in the thalamus	1999	34	391-398	Neurochem Int
Obrenovitch, T. P.	Origins of glutamate release in ischaemia	1996	66	50-55	Acta Neurochir Suppl
Obrenovitch, T. P.; Girault, J. A.	Update on the monitoring of changes in extracellular glutamate, and their significance (multiple letters)	1998	9	i	Neuroreport
Obrenovitch, T. P.; Richards, D. A.	Extracellular neurotransmitter changes in cerebral ischaemia	1995	7	1-54	Cerebrovasc Brain Metab Rev
Obrenovitch, T. P.; Urenjak, J.; Richards, D. A.; Ueda, Y.; Curzon, G.; Symon, L.	Extracellular neuroactive amino acids in the rat striatum during ischaemia: comparison between penumbral conditions and ischaemia with sustained anoxic depolarisation	1993	61	178-186	J Neurochem
Obrenovitch, T. P.; Urenjak, J.; Zilkha, E.	Effects of increased extracellular glutamate levels on the local field potential in the brain of anaesthetized rats	1997	122	372-378	Br J Pharmacol
Obrenovitch, T. P.; Urenjak, J.; Zilkha, E.	Evidence disputing the link between seizure activity and high extracellular glutamate	1996	66	2446-2454	J Neurochem
Obrenovitch, T. P.; Urenjak, J.; Zilkha, E.;	Excitotoxicity in neurological disorders--the glutamate paradox	2000	18	281-287	Int J Dev Neurosci

Glutamate					
**Authors**	**Title**	**Year**	**Vol.**	**Pages**	**Journal**
Jay, T. M.					
Obrenovitch, T. P.; Zilkha, E.; Urenjak, J.	Effects of pharmacological inhibition of glutamate-uptake on ischaemia-induced glutamate efflux and anoxic depolarization latency	1998	357	225-231	Naunyn-Schmiedeberg's Archives of Pharmacology
Obrenovitch, T. P.; Zilkha, E.; Urenjak, J.	Evidence against high extracellular glutamate promoting the elicitation of spreading depression by potassium	1996	16	923-931	J Cereb Blood Flow Metab
Obrenovitch, T. P.; Zilkha, E.; Urenjak, J.	Intracerebral microdialysis: electrophysiological evidence of a critical pitfall	1995	64	1884-1887	J Neurochem
Ochi, M.; Shiozaki, S.; Kase, H.	Adenosine A(2A) receptor-mediated modulation of GABA and glutamate release in the output regions of the basal ganglia in a rodent model of Parkinson's disease	2004	127	223-231	Neuroscience
Oda, M.; Kure, S.; Sugawara, T.; Yamaguchi, S.; Kojima, K.; Shinka, T.; Sato, K.; Narisawa, A.; Aoki, Y.; Matsubara, Y.; Omae, T.; Mizoi, K.; Kinouchi, H.	Direct correlation between ischemic injury and extracellular glycine concentration in mice with genetically altered activities of the glycine cleavage multienzyme system	2007	38	2157-2164	Stroke
Ogawa, H.; Mizusawa, A.; Kikuchi, Y.; Hida, W.; Miki, H.; Shirato, K.	Nitric oxide as a retrograde messenger in the nucleus tractus solitarii of rats during hypoxia	1995	486(Pt2)	495-504	J Physiol
Ogawa, M.; Miyakawa, T.; Nakamura, K.; Kitano, J.; Furushima, K.; Kiyonari, H.; Nakayama, R.; Nakao, K.; Moriyoshi, K.; Nakanishi, S.	Altered sensitivities to morphine and cocaine in scaffold protein tamalin knockout mice	2007	104	14789-14794	Proc Natl Acad Sci U S A
Ohashi, M.; Saitoh, A.; Oka, J. I.; Yamada, M.	Riluzole in the prelimbic medial prefrontal cortex attenuates veratrine-induced anxiety-like behaviors in mice	2015	232	391-398	Psychopharmacology
Ohmori, T.; Abekawa, T.; Koyama, T.	The role of glutamate in behavioral and neurotoxic effects of methamphetamine	1996	29	301-307	Neurochem Int
Ohoyama, K.; Yamamura, S.; Hamaguchi, T.; Nakagawa, M.; Motomura, E.; Shiroyama, T.; Tanii, H.; Okada, M.	Effect of novel atypical antipsychotic, blonanserin, on extracellular neurotransmitter level in rat prefrontal cortex	2011	653	47-57	Eur J Pharmacol
Ohta, H.; Li, X.; Talman, W. T.	Release of glutamate in the nucleus tractus solitarii in response to baroreflex activation in rats	1996	74	29-37	Neuroscience
Ohta, K.; Araki, N.; Shibata, M.; Hamada, J.; Komatsumoto, S.; Shimazu, K.;	Correlation of in vivo nitric oxide and cGMP with glutamate/glutamine metabolism in the rat striatum	1996	25	379-384	Neurosci Res

Glutamate					
**Authors**	**Title**	**Year**	**Vol.**	**Pages**	**Journal**
Fukuuchi, Y.					
Ohta, K.; Fukuuchi, Y.; Shimazu, K.; Komatsumoto, S.; Ichijo, M.; Araki, N.; Shibata, M.	Presynaptic glutamate receptors facilitate release of norepinephrine and 5-hydroxytryptamine as well as dopamine in the normal and ischemic striatum	1994	49 Suppl	S195-S202	J Auton Nerv Syst
Ohtaki, K.; Matsubara, K.; Fujimaru, S.; Shimizu, K.; Awaya, T.; Suno, M.; Chiba, K.; Hayase, N.; Shiono, H.	Cefoselis, a beta-lactam antibiotic, easily penetrates the blood-brain barrier and causes seizure independently by glutamate release	2004	111	1523-1535	J Neural Transm(Vienna)
Ojanen, S. P.; Palmen, M.; Hyytia, P.; Kiianmaa, K.	Extracellular glutamate and GABA in the ventral tegmental area of alcohol-preferring AA and alcohol-avoiding ANA rats treated repeatedly with morphine	2007	559	38-45	Eur J Pharmacol
Oka, S.; Matsumoto, M.; Ohtake, K.; Kiyoshima, T.; Nakakimura, K.; Sakabe, T.	The addition of epinephrine to tetracaine injected intrathecally sustains an increase in glutamate concentrations in the cerebrospinal fluid and worsens neuronal injury	2001	93	1050-1057	Anesth Analg
Okada, M.; Kawata, Y.; Mizuno, K.; Wada, K.; Kondo, T.; Kaneko, S.	Interaction between Ca2+, K+, carbamazepine and zonisamide on hippocampal extracellular glutamate monitored with a microdialysis electrode	1998	124	1277-1285	Br J Pharmacol
Okada, M.; Zhu, G.; Yoshida, S.; Hirose, S.; Kaneko, S.	Protein kinase associated with gating and closing transmission mechanisms in temporoammonic pathway	2004	47	485-504	Neuropharmacology
Okuda, N.; Kohara, K.; Mikami, H.; Moriguchi, A.; Yamada, K.; Higaki, J.; Ogihara, T.	Effect of propranolol on central neurotransmitter release in Wistar rats analysed by brain microdialysis	1999	26	220-224	Clin Exp Pharmacol Physiol
Oldenziel, W. H.; Dijkstra, G.; Cremers, T. I.; Westerink, B. H.	Evaluation of hydrogel-coated glutamate microsensors	2006	78	3366-3378	Anal Chem
Oliva, P.; Berrino, L.; de Novellis, V.; Palazzo, E.; Marabese, I.; Siniscalco, D.; Scafuro, M.; Mariani, L.; Rossi, F.; Maione, S.	Role of periaqueductal grey prostaglandin receptors in formalin-induced hyperalgesia	2006	530	40-47	Eur J Pharmacol
Onaka, T.; Yagi, K.	Involvement of N-methyl-D-aspartic acid receptor activation in oxytocin and vasopressin release after osmotic stimuli in rats	2001	13	166-174	J Neuroendocrinol
Ono, K.; Iwatsuki, N.; Tajima, T.; Takahashi, M.; Hashimoto, Y.	Elevation of the extracellular glutamate concentration in the hippocampus after total cerebral ischemia related to the deterioration of the recovery in EEG and evoked potentials in dogs	1993	7	334-340	J Anesth

Glutamate					
**Authors**	**Title**	**Year**	**Vol.**	**Pages**	**Journal**
Ooboshi, H.; Ibayashi, S.; Takano, K.; Sadoshima, S.; Kondo, A.; Uchimura, H.; Fujishima, M.	Hypothermia inhibits ischemia-induced efflux of amino acids and neuronal damage in the hippocampus of aged rats	2000	884	23-30	Brain Research
Ooboshi, H.; Sadoshima, S.; Yao, H.; Ibayashi, S.; Matsumoto, T.; Uchimura, H.; Fujishima, M.	Ischemia-induced release of amino acids in the hippocampus of aged hypertensive rats	1995	15	227-234	J Cereb Blood Flow Metab
Opacka-Juffry, J.; Ashworth, S.; Ahier, R. G.; Hume, S. P.	Modulatory effects of L-DOPA on D2 dopamine receptors in rat striatum, measured using in vivo microdialysis and PET	1998	105	349-364	J Neural Transm (Vienna)
Oreiro-Garcia, M. T.; Vazquez-Illanes, M. D.; Sierra-Paredes, G.; Sierra-Marcuno, G.	Changes in extracellular amino acid concentrations in the rat hippocampus after in vivo actin depolymerization with latrunculin A	2007	50	734-740	Neurochem Int
Oreiro-Garcia, M. T.; Vazquez-Illanes, M. D.; Sierra-Paredes, G.; Sierra-Marcuno, G.	Analysis of neuroactive amino acids from microdialysate samples by fluorescence detection using a modification of the 6-aminoquinolyl-N-hydroxysuccinimidyl carbamate method	2005	19	720-724	Biomed Chromatogr
Oria, M.; Chatauret, N.; Chavarria, L.; Romero-Gimenez, J.; Palenzuela, L.; Pardo-Yules, B.; Arranz, J. A.; Bodega, G.; Raguer, N.; Cordoba, J.	Motor-evoked potentials in awake rats are a valid method of assessing hepatic encephalopathy and of studying its pathogenesis	2010	52	2077-2085	Hepatology
Oria, M.; Romero-Gimenez, J.; Arranz, J. A.; Riudor, E.; Raguer, N.; Cordoba, J.	Ornithine phenylacetate prevents disturbances of motor-evoked potentials induced by intestinal blood in rats with portacaval anastomosis	2012	56	109-114	J Hepatol
Oria, M.; Romero-Gimenez, J.; Arranz, J. A.; Riudor, E.; Raguer, N.; Cordoba, J.	Ornithine phenylacetate (OP) prevents neurophysiological abnormalities in an experimental model of hepatic encephalopathy	2011	54	S376	J Hepatol
Orwar, O.; Li, X.; Andine, P.; Bergstrom, C. M.; Hagberg, H.; Folestad, S.; Sandberg, M.	Increased intra- and extracellular concentrations of gamma-glutamylglutamate and related dipeptides in the ischemic rat striatum: involvement of glutamyl transpeptidase	1994	63	1371-1376	J Neurochem
Osborne, P. G.; Hashimoto, M.	Mammalian cerebral metabolism and amino acid neurotransmission during hibernation	2008	106	1888-1899	J Neurochem
Oshinsky, M. L.; Gomonchareonsiri, S.	Episodic dural stimulation in awake rats: a model for recurrent headache	2007	47	1026-1036	Headache
Oshinsky, M. L.; Luo, J.	Neurochemistry of trigeminal activation in an animal model of migraine	2006	46 Suppl1	S39-S44	Headache

Glutamate					
**Authors**	**Title**	**Year**	**Vol.**	**Pages**	**Journal**
Oskarsson, A.; Ljungberg, T.; Stahle, L.	Behavioral and neurochemical effects after combined perinatal treatment of rats with lead and disulfiram	1986	8	591-599	Neurotoxicol Teratol
Ossanai, L. T.; Lourenco, G. A.; Nencioni, A. L.; Lebrun, I.; Yamanouye, N.; Dorce, V. A.	Effects of a toxin isolated from Tityus bahiensis scorpion venom on the hippocampus of rats	2012	91	230-236	Life Sci
Osuga, H.; Hakim, A. M.	Relationship between extracellular glutamate concentration and voltage-sensitive calcium channel function in focal cerebral ischemia in the rat	1996	16	629-636	J Cereb Blood Flow Metab
Osuga, H.; Hakim, A. M.	Relevance of interstitial glutamate to selective vulnerability in focal cerebral ischemia	1994	14	343-347	J Cereb Blood Flow Metab
Ou, J. J.; Zhang, Y.; Montine, T. J.	In vivo assessment of lipid peroxidation products associated with age-related neurodegenerative diseases	2002	175	363-369	Exp Neurol
Ovanesov, M. V.; Vogel, M. W.; Moran, T. H.; Pletnikov, M. V.	Neonatal Borna disease virus infection in rats is associated with increased extracellular levels of glutamate and neurodegeneration in the striatum	2007	13	185-194	J Neurovirol
Owen, A. J.; Ijaz, S.; Miyashita, H.; Wishart, T.; Howlett, W.; Shuaib, A.	Zonisamide as a neuroprotective agent in an adult gerbil model of global forebrain ischemia: a histological, in vivo microdialysis and behavioral study	1997	770	115-122	Brain Res
Ozyurt Bayraktar, H.; Yananli, H.; Terzioglu, B.; Oktay, S.; Kaleli, M.; Goren, M. Z.	The role of NO in the posterior hypothalamus in amygdala-generated pressor responses in conscious rats	2013	33	7-16	Auton Autacoid Pharmacol
Palazzo, E.; Marabese, I.; Soukupova, M.; Luongo, L.; Boccella, S.; Giordano, C.; de Novellis, V.; Rossi, F.; Maione, S.	Metabotropic glutamate receptor subtype 8 in the amygdala modulates thermal threshold, neurotransmitter release, and rostral ventromedial medulla cell activity in inflammatory pain	2011	31	4687-4697	J Neurosci
Palmer, A. M.; Hutson, P. H.; Lowe, S. L.; Bowen, D. M.	Extracellular concentrations of aspartate and glutamate in rat neostriatum following chemical stimulation of frontal cortex	1989	75	659-663	Exp Brain Res
Palmer, A. M.; Marion, D. W.; Botscheller, M. L.; Redd, E. E.	Therapeutic hypothermia is cytoprotective without attenuating the traumatic brain injury-induced elevations in interstitial concentrations of aspartate and glutamate	1993	10	363-372	J Neurotrauma
Palmer, A. M.; Marion, D. W.; Botscheller, M. L.; Swedlow, P. E.; Styren, S. D.; DeKosky, S. T.	Traumatic brain injury-induced excitotoxicity assessed in a controlled cortical impact model	1993	61	2015-2024	J Neurochem
Pan, X.; Sun, L.; Ma, W.; Tang, Y.; Long,	Overactivation of poly(adenosine phosphate-ribose) polymerase	2007	134	1227-	J Thorac Cardiovasc Surg

Glutamate					
**Authors**	**Title**	**Year**	**Vol.**	**Pages**	**Journal**
C.; Tian, L.; Liu, N.; Feng, Z.; Zheng, J.	1 and molecular events in neuronal injury after deep hypothermic circulatory arrest: study in a rabbit model			1233	
Panter, S. S.; Faden, A. I.	Pretreatment with NMDA antagonists limits release of excitatory amino acids following traumatic brain injury	1992	136	165-168	Neurosci Lett
Paolone, G.; Brugnoli, A.; Arcuri, L.; Mercatelli, D.; Morari, M.	Eltoprazine prevents levodopa-induced dyskinesias by reducing striatal glutamate and direct pathway activity	2015	30	1728-1738	Mov Disord
Paredes, D. A.; Cartford, M. C.; Catlow, B. J.; Samec, A.; Avilas, M.; George, A.; Schlunck, A.; Small, B.; Bickford, P. C.	Neurotransmitter release during delay eyeblink classical conditioning: role of norepinephrine in consolidation and effect of age	2009	92	267-282	Neurobiol Learn Mem
Paredes, D.; Granholm, A. C.; Bickford, P. C.	Effects of NGF and BDNF on baseline glutamate and dopamine release in the hippocampal formation of the adult rat	2007	1141	56-64	Brain Res
Park, E.; Lee, G. J.; Choi, S.; Choi, S. K.; Chae, S. J.; Kang, S. W.; Pak, Y. K.; Park, H. K.	The role of glutamate release on voltage-dependent anion channels (VDAC)-mediated apoptosis in an eleven vessel occlusion model in rats	2010	5	e15192	PloS One
Parrot, S.; Bert, L.; Renaud, B.; Denoroy, L.	Glutamate and aspartate do not exhibit the same changes in their extracellular concentrations in the rat striatum after N-methyl-D-aspartate local administration	2003	71	445-454	J Neurosci Res
Parrot, S.; Bert, L.; Renaud, B.; Denoroy, L.	Large inter-experiment variations in microdialysate aspartate and glutamate in rat striatum may reflect a circannual rhythm	2001	39	267-269	Synapse
Parrot, S.; Sauvinet, V.; Riban, V.; Depaulis, A.; Renaud, B.; Denoroy, L.	High temporal resolution for in vivo monitoring of neurotransmitters in awake epileptic rats using brain microdialysis and capillary electrophoresis with laser-induced fluorescence detection	2004	140	29-38	J Neurosci Methods
Parsegian, A.; See, R. E.	Dysregulation of dopamine and glutamate release in the prefrontal cortex and nucleus accumbens following methamphetamine self-administration and during reinstatement in rats	2014	39	811-822	Neuropsychopharmacology
Pascual, M.; Boix, J.; Felipo, V.; Guerri, C.	Repeated alcohol administration during adolescence causes changes in the mesolimbic dopaminergic and glutamatergic systems and promotes alcohol intake in the adult rat	2009	108	920-931	J Neurochem
Pasumarthi, R. K.; Fadel, J.	Stimulation of lateral hypothalamic glutamate and acetylcholine efflux by nicotine: implications for mechanisms of nicotine-induced activation of orexin neurons	2010	113	1023-1035	J Neurochem

Glutamate					
Authors	Title	Year	Vol.	Pages	Journal
Patel, D. R.; Young, A. M.; Croucher, M. J.	Presynaptic alpha-amino-3-hydroxy-5-methyl-4-isoxazole propionate receptor-mediated stimulation of glutamate and GABA release in the rat striatum in vivo: a dual-label microdialysis study	2001	12	101-111	Neuroscience
Patel, D.; Bohlke, M.; Phattanarudee, S.; Kabadi, S.; Maher, T. J.; Ally, A.	Cardiovascular responses and neurotransmitter changes during blockade of angiotensin II receptors within the ventrolateral medulla	2008	60	340-348	Neurosci Res
Patel, P. M.; Drummond, J. C.; Cole, D. J.; Goskowicz, R. L.	Isoflurane reduces ischemia-induced glutamate release in rats subjected to forebrain ischemia	1995	82	996-1003	Anesthesiology
Patel, P. M.; Drummond, J. C.; Cole, D. J.; Yaksh, T. L.	Differential temperature sensitivity of ischemia-induced glutamate release and eicosanoid production in rats	1994	650	205-211	Brain Res
Patel, P. M.; Goskowicz, R. L.; Drummond, J. C.; Cole, D. J.	Etomidate reduces ischemia-induced glutamate release in the hippocampus in rats subjected to incomplete forebrain ischemia	1995	80	933-939	Anesth Analg
Pati, D.; Kelly, K.; Stennett, B.; Frazier, C. J.; Knackstedt, L. A.	Alcohol consumption increases basal extracellular glutamate in the nucleus accumbens core of Sprague-Dawley rats without increasing spontaneous glutamate release	2016	44	1896-1905	Eur J Neurosci
Paulsen, R. E.; Fonnum, F.	Role of glial cells for the basal and Ca2+-dependent K+-evoked release of transmitter amino acids investigated by microdialysis	1989	52	1823-1829	J Neurochem
Pellegrini-Giampietro, D. E.; Peruginelli, F.; Meli, E.; Cozzi, A.; Albani-Torregrossa, S.; Pellicciari, R.; Moroni, F.	Protection with metabotropic glutamate 1 receptor antagonists in models of ischemic neuronal death: time-course and mechanisms	1999	38	1607-1619	Neuropharmacology
Pena, F.; Tapia, R.	Relationships among seizures, extracellular amino acid changes, and neurodegeneration induced by 4-aminopyridine in rat hippocampus: a microdialysis and electroencephalographic study	1999	72	2006-2014	J Neurochem
Penning, D. H.; Chestnut, D. H.; Dexter, F.; Hrdy, J.; Poduska, D.; Arkins, B.	Glutamate release from the ovine fetal brain during maternal hemorrhage: A study using chronic in utero cerebral microdialysis	1995	82	521-530	Anesthesiology
Penning, D. H.; Dexter, F.; Henderson, J. L.; Chestnut, D. H.; Reynolds, J. D.	Bolus maternal cocaine administration does not produce a large increase in fetal sheep cerebral cortical glutamate concentration	1999	21	177-180	Neurotoxicol Teratol
Perez, J.; Colasante, C.; Tucci, S.; Hernandez, L.; Rada, P.	Effects of feeding on extracellular levels of glutamate in the medial and lateral portion of the globus pallidus of freely moving rats	2000	877	91-94	Brain Res
Perez-Pinzon, M. A.; Nilsson, G. E.; Lutz, P. L.	Relationship between ion gradients and neurotransmitter release in the newborn rat striatum during anoxia	1993	602	228-233	Brain Res
Perry, V. L.; Young, R. S.; Aquila, W. J.;	Effect of experimental Escherichia coli meningitis on	1993	34	187-191	Pediatr Res

Glutamate					
**Authors**	**Title**	**Year**	**Vol.**	**Pages**	**Journal**
During, M. J.	concentrations of excitatory and inhibitory amino acids in the rabbit brain: in vivo microdialysis study				
Petkova-Kirova, P.; Rakovska, A.; Della Corte, L.; Zaekova, G.; Radomirov, R.; Mayer, A.	Neurotensin modulation of acetylcholine, GABA, and aspartate release from rat prefrontal cortex studied in vivo with microdialysis	2008	77	129-135	Brain Res Bull
Phattanarudee, S.; Towiwat, P.; Maher, T. J.; Ally, A.	Effects of medullary administration of a nitric oxide precursor on cardiovascular responses and neurotransmission during static exercise following ischemic stroke	2013	91	510-520	Can J Physiol Pharmacol
Phillis, J. W.; Song, D.; Guyot, L. L.; Regan, M. H.	Lactate reduces amino acid release and fuels recovery of function in the ischemic brain	1999	272	195-198	Neurosci Lett
Piantadosi, C. A.; Zhang, J.; Levin, E. D.; Folz, R. J.; Schmechel, D. E.	Apoptosis and delayed neuronal damage after carbon monoxide poisoning in the rat	1997	147	103-114	Exp Neurol
Piepponen, T. P.; Kiianmaa, K.; Ahtee, L.	Effects of ethanol on the accumbal output of dopamine, GABA and glutamate in alcohol-tolerant and alcohol-nontolerant rats	2002	74	21-30	Pharmacol Biochem Behav
Piepponen, T. P.; Skujins, A.	Rapid and sensitive step gradient assays of glutamate, glycine, taurine and gamma-aminobutyric acid by high-performance liquid chromatography-fluorescence detection with o-phthalaldehyde-mercaptoethanol derivatization with an emphasis on microdialysis samples	2001	757	277-283	J Chromatogr B Analyt Technol Biomed Life Sci
Pierard, C.; Lagarde, D.; Barrere, B.; Duret, P.; Cordeiro, C.; Guezennec, C. Y.; Peres, M.	Effects of a vigilance enhancing drug, modafinil, on rat brain cortex amino acids: A microdialysis study	1997	25	51-54	Med Sci Res
Pierard, C.; Peres, M.; Satabin, P.; Guezennec, C. Y.; Lagarde, D.	Effects of GABA-transaminase inhibition on brain metabolism and amino-acid compartmentation: an in vivo study by 2D 1H-NMR spectroscopy coupled with microdialysis	1999	127	321-327	Exp Brain Res
Pierce, R. C.; Bell, K.; Duffy, P.; Kalivas, P. W.	Repeated cocaine augments excitatory amino acid transmission in the nucleus accumbens only in rats having developed behavioral sensitization	1996	16	1550-1560	J Neurosci
Pierce, R. C.; Reeder, D. C.; Hicks, J.; Morgan, Z. R.; Kalivas, P. W.	Ibotenic acid lesions of the dorsal prefrontal cortex disrupt the expression of behavioral sensitization to cocaine	1998	82	1103-1114	Neuroscience
Pietraszek, M.; Golembiowska, K.; Bijak, M.; Ossowska, K.; Wolfarth, S.	Differential effects of chronic haloperidol and clozapine administration on glutamatergic transmission in the fronto-parietal cortex in rats: microdialysis and electrophysiological studies	2002	366	417-424	Naunyn Schmiedebergs Arch Pharmacol

Glutamate					
**Authors**	**Title**	**Year**	**Vol.**	**Pages**	**Journal**
Pietraszek, M.; Michaluk, J.; Romanska, I.; Wasik, A.; Golembiowska, K.; Antkiewicz-Michaluk, L.	1-Methyl-1,2,3,4-tetrahydroisoquinoline antagonizes a rise in brain dopamine metabolism, glutamate release in frontal cortex and locomotor hyperactivity produced by MK-801 but not the disruptions of prepulse inhibition, and impairment of working memory in rat	2009	16	390-407	Neurotox Res
Ping, L.; Mahesh, V. B.; Wiedmeier, V. T.; Brann, D. W.	Release of glutamate and aspartate from the preoptic area during the progesterone-induced LH surge: in vivo microdialysis studies	1994	59	318-324	Neuroendocrinology
Pintor, A.; Galluzzo, M.; Grieco, R.; Pezzola, A.; Reggio, R.; Popoli, P.	Adenosine A 2A receptor antagonists prevent the increase in striatal glutamate levels induced by glutamate uptake inhibitors	2004	89	152-156	J Neurochem
Pintor, A.; Pezzola, A.; Reggio, R.; Quarta, D.; Popoli, P.	The mGlu5 receptor agonist CHPG stimulates striatal glutamate release: possible involvement of A2A receptors	2000	11	3611-3614	Neuroreport
Pintor, A.; Quarta, D.; Pezzola, A.; Reggio, R.; Popoli, P.	SCH 58261 (an adenosine A(2A) receptor antagonist) reduces, only at low doses, K(+)-evoked glutamate release in the striatum	2001	421	177-180	Eur J Pharmacol
Pintor, A.; Tebano, M. T.; Martire, A.; Grieco, R.; Galluzzo, M.; Scattoni, M. L.; Pezzola, A.; Coccurello, R.; Felici, F.; Cuomo, V.; Piomelli, D.; Calamandrei, G.; Popoli, P.	The cannabinoid receptor agonist WIN 55,212-2 attenuates the effects induced by quinolinic acid in the rat striatum	2006	51	1004-1012	Neuropharmacology
Piroli, G. G.; Reznikov, L. R.; Grillo, C. A.; Hagar, J. M.; Fadel, J. R.; Reagan, L. P.	Tianeptine modulates amygdalar glutamate neurochemistry and synaptic proteins in rats subjected to repeated stress	2013	241	184-193	Exp Neurol
Pistis, M.; Ferraro, L.; Pira, L.; Flore, G.; Tanganelli, S.; Gessa, G. L.; Devoto, P.	Delta(9)-tetrahydrocannabinol decreases extracellular GABA and increases extracellular glutamate and dopamine levels in the rat prefrontal cortex: an in vivo microdialysis study	2002	948	155-158	Brain Res
Placenza, F. M.; Rajabi, H.; Stewart, J.	Effects of chronic buprenorphine treatment on levels of nucleus accumbens glutamate and on the expression of cocaine-induced behavioral sensitization in rats	2008	200	347-355	Psychopharmacology(Berl)
Pocivavsek, A.; Wu, H. Q.; Elmer, G. I.; Bruno, J. P.; Schwarcz, R.	Pre- and postnatal exposure to kynurenine causes cognitive deficits in adulthood	2012	35	1605-1612	Eur J Neurosci
Pocivavsek, A.; Wu, H. Q.; Okuyama, M.; Kajii, Y.; Bruno, J. P.; Schwarcz, R.	The systemically active kynurenine aminotransferase II inhibitor BFF816 attenuates contextual memory deficit induced by prenatal kynurenine elevation in rats	2015	41	S8-S9	Schizophr Bull
Poe, G. R.; Nitz, D. A.; Rector, D. M.; Kristensen, M. P.; Harper, R. M.	Concurrent reflectance imaging and microdialysis in the freely behaving cat	1996	65	143-149	J Neurosci Methods

Glutamate					
**Authors**	**Title**	**Year**	**Vol.**	**Pages**	**Journal**
Pokela, M.; Biancari, F.; Rimpilainen, J.; Romsi, P.; Hirvonen, J.; Vainionpaa, V.; Kiviluoma, K.; Anttila, V.; Juvonen, T.	The role of cerebral microdialysis in predicting the outcome after experimental hypothermic circulatory arrest	2001	35	395-402	Scand Cardiovasc J
Polissidis, A.; Chouliara, O.; Galanopoulos, A.; Naxakis, G.; Papahatjis, D.; Papadopoulou-Daifoti, Z.; Antoniou, K.	Cannabinoids negatively modulate striatal glutamate and dopamine release and behavioural output of acute D-amphetamine	2014	270	261-269	Behav Brain Res
Polissidis, A.; Galanopoulos, A.; Naxakis, G.; Papahatjis, D.; Papadopoulou-Daifoti, Z.; Antoniou, K.	The cannabinoid CB1 receptor biphasically modulates motor activity and regulates dopamine and glutamate release region dependently	2013	16	393-403	Int J Neuropsychopharmacol
Popoli, P.; Betto, P.; Reggio, R.; Ricciarello, G.	Adenosine A2A receptor stimulation enhances striatal extracellular glutamate levels in rats	1995	287	215-217	Eur J Pharmacol
Popoli, P.; Pintor, A.; Tebano, M. T.; Frank, C.; Pepponi, R.; Nazzicone, V.; Grieco, R.; Pezzola, A.; Reggio, R.; Minghetti, L.; De Berardinis, M. A.; Martire, A.; Potenza, R. L.; Domenici, M. R.; Massotti, M.	Neuroprotective effects of the mGlu5R antagonist MPEP towards quinolinic acid-induced striatal toxicity: involvement of pre- and post-synaptic mechanisms and lack of direct NMDA blocking activity	2004	89	1479-1489	J Neurochem
Portelli, J.; Aourz, N.; De Bundel, D.; Meurs, A.; Smolders, I.; Michotte, Y.; Clinckers, R.	Intrastrain differences in seizure susceptibility, pharmacological response and basal neurochemistry of Wistar rats	2009	87	234-246	Epilepsy Res
Portelli, J.; Smolders, I.	Intracerebral microdialysis in the study of limbic seizure mechanisms and antiepileptic drug action using freely moving rats	2013	75	321-337	Microdialys Tech Neurosci
Potter, M. C.; Elmer, G. I.; Bergeron, R.; Albuquerque, E. X.; Guidetti, P.; Wu, H. Q.; Schwarcz, R.	Reduction of endogenous kynurenic acid formation enhances extracellular glutamate, hippocampal plasticity, and cognitive behavior	2010	35	1734-1742	Neuropsychopharmacology
Pozzi, L.; Baviera, M.; Sacchetti, G.; Calcagno, E.; Balducci, C.; Invernizzi, R. W.; Carli, M.	Attention deficit induced by blockade of N-methyl D-aspartate receptors in the prefrontal cortex is associated with enhanced glutamate release and cAMP response element binding protein phosphorylation: role of metabotropic glutamate receptors 2/3	2011	176	336-348	Neuroscience
Prazak, J.; Laszikova, E.; Pantoflicek, T.; Ryska, O.; Koblihova, E.; Ryska, M.	Cerebral microdialysis reflects the neuroprotective effect of fractionated plasma separation and adsorption in acute liver	2013	13	98	BMC Gastroenterol

Glutamate					
**Authors**	**Title**	**Year**	**Vol.**	**Pages**	**Journal**
	failure better and earlier than intracranial pressure: a controlled study in pigs				
Pu, J.; Niu, X.; Zhao, J.	Excitatory amino acid changes in the brains of rhesus monkeys following selective cerebral deep hypothermia and blood flow occlusion	2013	8	143-148	Neural Regen Res
Puka, M.; Lehmann, A.	In vivo acidosis reduces extracellular concentrations of taurine and glutamate in the rat hippocampus	1994	37	641-646	J Neurosci Res
Puka-Sundvall, M.; Eriksson, P.; Nilsson, M.; Sandberg, M.; Lehmann, A.	Neurotoxicity of cysteine: interaction with glutamate	1995	705	65-70	Brain Res
Puka-Sundvall, M.; Sandberg, M.; Hagberg, H.	Brain injury after hypoxia-ischemia in newborn rats: relationship to extracellular levels of excitatory amino acids and cysteine	1997	750	325-328	Brain Res
Purins, K.; Enblad, P.; Wiklund, L.; Lewen, A.	Brain tissue oxygenation and cerebral perfusion pressure thresholds of ischemia in a standardized pig brain death model	2012	16	462-469	Neurocrit Care
Qi, J.; Han, W. Y.; Yang, J. Y.; Wang, L. H.; Dong, Y. X.; Wang, F.; Song, M.; Wu, C. F.	Oxytocin regulates changes of extracellular glutamate and GABA levels induced by methamphetamine in the mouse brain	2012	17	758-769	Addict Biol
Qin, S.; Evering, M.; Wahono, N.; Cremers, T. I. F. H.; Westerink, B. H. C.	Monitoring extracellular glutamate in the brain by microdialysis and microsensors	2013	Neuromethods. 80	153-177	Microelectrode Biosensors
Qu, X.; Xu, C.; Wang, H.; Xu, J.; Liu, W.; Wang, Y.; Jia, X.; Xie, Z.; Xu, Z.; Ji, C.; Wu, A.; Yue, Y.	Hippocampal glutamate level and glutamate aspartate transporter (GLAST) are up-regulated in senior rat associated with isoflurane-induced spatial learning/memory impairment	2013	38	59-73	Neurochem Res
Qu, Y.; Arckens, L.; Vandenbussche, E.; Geeraerts, S.; Vandesande, F.	Simultaneous determination of total and extracellular concentrations of the amino acid neurotransmitters in cat visual cortex by microbore liquid chromatography and electrochemical detection	1998	798	19-26	J Chromatogr A
Qu, Y.; Arckens, L.; Vandesande, F.; Vandenbussche, E.	Sampling extracellular aspartate, glutamate and gamma-aminobutyric acid in striate cortex of awake cat by in vivo microdialysis: surgical and methodological aspects	2000	866	116-127	Brain Res
Qu, Y.; Li, Y.; Vandenbussche, E.; Vandesande, F.; Arckens, L.	In vivo microdialysis in the visual cortex of awake cat. II: sample analysis by microbore HPLC-electrochemical detection and capillary electrophoresis-laser-induced fluorescence detection	2001	7	45-51	Journal Res Brain Res Protocol
Qu, Y.; Massie, A.; Van der Gucht, E.; Cnops, L.; Vandenbussche, E.; Eysel, U.	Retinal lesions affect extracellular glutamate levels in sensory-deprived and remote non-deprived regions of cat area 17 as	2003	962	199-206	Brain Res

Glutamate					
**Authors**	**Title**	**Year**	**Vol.**	**Pages**	**Journal**
T.; Vandesande, F.; Arckens, L.	revealed by in vivo microdialysis				
Qu, Y.; Saint Marie, R. L.; Breier, M. R.; Ko, D.; Stouffer, D.; Parsons, L. H.; Swerdlow, N. R.	Neural basis for a heritable phenotype: Differences in the effects of apomorphine on startle gating and ventral pallidal GABA efflux in male Sprague-Dawley and Long-Evans rats	2009	207	271-280	Psychopharmacology
Quarta, D.; Borycz, J.; Solinas, M.; Patkar, K.; Hockemeyer, J.; Ciruela, F.; Lluis, C.; Franco, R.; Woods, A. S.; Goldberg, S. R.; Ferre, S.	Adenosine receptor-mediated modulation of dopamine release in the nucleus accumbens depends on glutamate neurotransmission and N-methyl-D-aspartate receptor stimulation	2004	91	873-880	J Neurochem
Quarta, D.; Ferre, S.; Solinas, M.; You, Z. B.; Hockemeyer, J.; Popoli, P.; Goldberg, S. R.	Opposite modulatory roles for adenosine A1 and A2A receptors on glutamate and dopamine release in the shell of the nucleus accumbens. Effects of chronic caffeine exposure	2004	88	1151-1158	J Neurochem
Quertemont, E.; Dahchour, A.; Ward, R. J.; De Witte, P.	Ethanol induces taurine release in the amygdala: An in vivo microdialysis study	1999	4	47-54	Addict Biol
Quertemont, E.; de Neuville, J.; De Witte, P.	Changes in the amygdala amino acid microdialysate after conditioning with a cue associated with ethanol	1998	139	71-78	Psychopharmacology(Berl)
Quertemont, E.; Lallemand, F.; Colombo, G.; De Witte, P.	Taurine and ethanol preference: a microdialysis study using Sardinian alcohol-preferring and non-preferring rats	2000	10	377-383	Eur Neuropsychopharmacol
Quertemont, E.; Linotte, S.; de Witte, P.	Differential taurine responsiveness to ethanol in high- and low-alcohol sensitive rats: a brain microdialysis study	2002	444	143-150	Eur J Pharmacol
Quinton, M. S.; Yamamoto, B. K.	Neurotoxic effects of chronic restraint stress in the striatum of methamphetamine-exposed rats	2007	193	341-350	Psychopharmacology(Berl)
Qureshi, A. I.; Ali, Z.; Suri, M. F.; Shuaib, A.; Baker, G.; Todd, K.; Guterman, L. R.; Hopkins, L. N.	Extracellular glutamate and other amino acids in experimental intracerebral hemorrhage: an in vivo microdialysis study	2003	31	1482-1489	Crit Care Med
Rabouan, S.; Olivier, J. C.; Guillemin, H.; Barthes, D.	Validation of HPLC analysis of aspartate and glutamate neurotransmitters following o-phthaldialdehyde-mercaptoethanol derivatization	2003	26	1797-1808	J Liq Chromatogr Relat Technol
Rada, P.; Mendialdua, A.; Hernandez, L.; Hoebel, B. G.	Extracellular glutamate increases in the lateral hypothalamus during meal initiation, and GABA peaks during satiation: microdialysis measurements every 30 s	2003	117	222-227	Behav Neurosci
Rada, P.; Moreno, S. A.; Tucci, S.; Gonzalez, L. E.; Harrison, T.; Chau, D. T.; Hoebel, B. G.; Hernandez, L.	Glutamate release in the nucleus accumbens is involved in behavioral depression during the PORSOLT swim test	2003	119	557-565	Neuroscience

Glutamate					
**Authors**	**Title**	**Year**	**Vol.**	**Pages**	**Journal**
Rada, P.; Tucci, S.; Murzi, E.; Hernandez, L.	Extracellular glutamate increases in the lateral hypothalamus and decreases in the nucleus accumbens during feeding	1997	768	338-340	Brain Res
Radhakrishnan, R.; Sluka, K. A.	Increased glutamate and decreased glycine release in the rostral ventromedial medulla during induction of a pre-clinical model of chronic widespread muscle pain	2009	457	141-145	Neurosci Lett
Rahman, S.; Bardo, M. T.	Environmental enrichment increases amphetamine-induced glutamate neurotransmission in the nucleus accumbens: a neurochemical study	2008	1197	40-46	Brain Res
Rajarao, S. J.; Platt, B.; Sukoff, S. J.; Lin, Q.; Bender, C. N.; Nieuwenhuijsen, B. W.; Ring, R. H.; Schechter, L. E.; Rosenzweig-Lipson, S.; Beyer, C. E.	Anxiolytic-like activity of the non-selective galanin receptor agonist, galnon	2007	41	307-320	Neuropeptides
Rakovska, A.; Giovannini, M. G.; Corte, L. D.; Kalfin, R.; Bianchi, L.; Pepeu, G.	Neurotensin modulation of acetylcholine and GABA release from the rat hippocampus: An in vivo microdialysis study	1998	33	335-340	Neurochem Int
Ramirez-Munguia, N.; Vera, G.; Tapia, R.	Epilepsy, neurodegeneration, and extracellular glutamate in the hippocampus of awake and anesthetized rats treated with okadaic acid	2003	28	1517-1524	Neurochem Res
Rao, V. L.; Audet, R. M.; Butterworth, R. F.	Selective alterations of extracellular brain amino acids in relation to function in experimental portal-systemic encephalopathy: results of an in vivo microdialysis study	1995	65	1221-1228	J Neurochem
Rappeneau, V.; Blaker, A.; Petro, J. R.; Yamamoto, B. K.; Shimamoto, A.	Disruption of the Glutamate-Glutamine Cycle Involving Astrocytes in an Animal Model of Depression for Males and Females	2016	10	231	Front Behav Neurosci
Rasmussen, B. A.; Baron, D. A.; Kim, J. K.; Unterwald, E. M.; Rawls, S. M.	beta-Lactam antibiotic produces a sustained reduction in extracellular glutamate in the nucleus accumbens of rats	2011	40	761-764	Amino Acids
Raudensky, J.; Yamamoto, B. K.	Effects of chronic unpredictable stress and methamphetamine on hippocampal glutamate function	2007	1135	129-135	Brain Res
Raudkivi, K.; Alttoa, A.; Leito, I.; Harro, J.	Differences in extracellular glutamate levels in striatum of rats with high and low exploratory activity	2015	67	858-865	Pharmacol Rep
Ravindran, J.; Shuaib, A.; Ijaz, S.; Galazka, P.; Waqar, T.; Ishaqzay, R.; Miyashita, H.; Liu, L.	High extracellular GABA levels in hippocampus--as a mechanism of neuronal protection in cerebral ischemia in adrenalectomized gerbils	1994	176	209-211	Neurosci Lett
Rawls, S. M.; McGinty, J. F.	Delta opioid receptors regulate calcium-dependent, amphetamine-evoked glutamate levels in the rat striatum: an in	2000	861	296-304	Brain Res

Glutamate					
**Authors**	**Title**	**Year**	**Vol.**	**Pages**	**Journal**
	vivo microdialysis study				
Rawls, S. M.; McGinty, J. F.	Muscarinic receptors regulate extracellular glutamate levels in the rat striatum: an in vivo microdialysis study	1998	286	91-98	J Pharmacol Exp Ther
Rawls, S. M.; McGinty, J. F.	Kappa receptor activation attenuates L-trans-pyrrolidine-2,4-dicarboxylic acid-evoked glutamate levels in the striatum	1998	70	626-634	J Neurochem
Rawls, S. M.; McGinty, J. F.	L-trans-pyrrolidine-2,4-dicarboxylic acid-evoked striatal glutamate levels are attenuated by calcium reduction, tetrodotoxin, and glutamate receptor blockade	1997	68	1553-1563	J Neurochem
Razoux, F.; Garcia, R.; Lena, I.	Ketamine, at a dose that disrupts motor behavior and latent inhibition, enhances prefrontal cortex synaptic efficacy and glutamate release in the nucleus accumbens	2007	32	719-727	Neuropsychopharmacology
Rea, M. A.; Ferriera, S.; Randolph, W.; Glass, J. D.	Daily profile of the extracellular concentration of glutamate in the suprachiasmatic region of the Siberian hamster	1993	204	104-109	Proc Soc Exp Biol Med
Reagan, L. P.; Reznikov, L. R.; Evans, A. N.; Gabriel, C.; Mocaer, E.; Fadel, J. R.	The antidepressant agomelatine inhibits stress-mediated changes in amino acid efflux in the rat hippocampus and amygdala	2012	1466	91-98	Brain Res
Rebec, G. V.; Witowski, S. R.; Sandstrom, M. I.; Rostand, R. D.; Kennedy, R. T.	Extracellular ascorbate modulates cortically evoked glutamate dynamics in rat striatum	2005	378	166-170	Neurosci Lett
Reid, M. S.; Berger, S. P.	Evidence for sensitization of cocaine-induced nucleus accumbens glutamate release	1996	7	1325-1329	Neuroreport
Reid, M. S.; Connor, W. T.; Herrera-Marschitz, M.; Ungerstedt, U.	The effects of intranigral GABA and dynorphin A injections on striatal dopamine and GABA release: evidence that dopamine provides inhibitory regulation of striatal GABA neurons via D2 receptors	1990	519	255-260	Brain Res
Reid, M. S.; Fox, L.; Ho, L. B.; Berger, S. P.	Nicotine stimulation of extracellular glutamate levels in the nucleus accumbens: neuropharmacological characterization	2000	35	129-136	Synapse
Reid, M. S.; Herrera-Marschitz, M.; Kehr, J.; Ungerstedt, U.	Striatal dopamine and glutamate release: effects of intranigral injections of substance P	1990	140	527-537	Acta Physiol Scand
Reid, M. S.; Hsu Jr, K.; Berger, S. P.	Cocaine and amphetamine preferentially stimulate glutamate release in the limbic system: Studies on the involvement of dopamine	1997	27	95-105	Synapse
Reidman, D. A.; Maher, T. J.; Chaiyakul, P.; Ally, A.	Modulation of extracellular glutamate and pressor response to muscle contraction during NMDA-receptor blockade in the rostral	2000	36	147-156	Neurosci Res

Glutamate					
**Authors**	**Title**	**Year**	**Vol.**	**Pages**	**Journal**
	ventrolateral medulla				
Reinhoud, N. J.; Brouwer, H. J.; van Heerwaarden, L. M.; Korte-Bouws, G. A.	Analysis of glutamate, GABA, noradrenaline, dopamine, serotonin, and metabolites using microbore UHPLC with electrochemical detection	2013	4	888-894	ACS Chem Neurosci
Reisi, P.; Alaei, H.; Babri, S.; Sharifi, M. R.; Mohaddes, G.; Soleimannejad, E.	Determination of the extracellular basal levels of glutamate and GABA at dentate gyrus of streptozotocin-induced diabetic rats	2009	16	63-66	Pathophysiology
Reisi, P.; Alaei, H.; Babri, S.; Sharifi, M. R.; Mohaddes, G.; Soleimannejad, E.; Rashidi, B.	Effects of treadmill running on extracellular basal levels of glutamate and GABA at dentate gyrus of streptozotocin-induced diabetic rats	2010	15	172-174	J Res Med Sci
Renno, W. M.	Microdialysis of excitatory amino acids in the periaqueductal gray of the rat after unilateral peripheral inflammation	1998	14	319-321	Amino Acids
Renno, W. M.; Alkhalaf, M.; Mousa, A.; Kanaan, R. A.	A comparative study of excitatory and inhibitory amino acids in three different brainstem nuclei	2008	33	150-159	Neurochem Res
Renno, W. M.; Mullet, M. A.; Williams, F. G.; Beitz, A. J.	Construction of 1 mm microdialysis probe for amino acids dialysis in rats	1998	79	217-228	J Neurosci Methods
Renno, W. M.; Mullett, M. A.; Beitz, A. J.	Systemic morphine reduces GABA release in the lateral but not the medial portion of the midbrain periaqueductal gray of the rat	1992	594	221-232	Brain Res
Reyes, E.; Rossell, S.; Paredes, D.; Rada, P.; Tucci, S.; Gonzalez, L. E.; Hernandez, L.	Haloperidol abolished glutamate release evoked by photic stimulation of the visual cortex in rats	2002	327	149-152	Neurosci Lett
Richards, D. A.; Lemos, T.; Whitton, P. S.; Bowery, N. G.	Extracellular GABA in the ventrolateral thalamus of rats exhibiting spontaneous absence epilepsy: a microdialysis study	1995	65	1674-1680	J Neurochem
Richards, D. A.; Morrone, L. A.; Bagetta, G.; Bowery, N. G.	Effects of alpha-dendrotoxin and dendrotoxin K on extracellular excitatory amino acids and on electroencephalograph spectral power in the hippocampus of anaesthetised rats	2000	293	183-186	Neurosci Lett
Richards, D. A.; Morrone, L. A.; Bowery, N. G.	Hippocampal extracellular amino acids and EEG spectral analysis in a genetic rat model of absence epilepsy	2000	39	2433-2441	Neuropharmacology
Richter, D. W.; Schmidt-Garcon, P.; Pierrefiche, O.; Bischoff, A. M.; Lalley, P. M.	Neurotransmitters and neuromodulators controlling the hypoxic respiratory response in anaesthetized cats	1999	514 (Pt2)	567-578	J Physiol
Rigaud-Monnet, A. S.; Heron, A.; Seylaz, J.; Pinard, E.	Effect of inhibiting NO synthesis on hippocampal extracellular glutamate concentration in seizures induced by kainic acid	1995	673	297-303	Brain Res
Rimpilainen, J.; Pokela, M.; Kiviluoma, K.;	The N-methyl-D-aspartate antagonist memantine has no	2001	121	957-970	J Thorac Cardiovasc Surg

Glutamate					
**Authors**	**Title**	**Year**	**Vol.**	**Pages**	**Journal**
Vainionpaa, V.; Hirvonen, J.; Ohtonen, P.; Jantti, V.; Anttila, V.; Heinonen, H.; Juvonen, T.	neuroprotective effect during hypothermic circulatory arrest: a study in the chronic porcine model				
Ritz, M. F.; Curin, Y.; Mendelowitsch, A.; Andriantsitohaina, R.	Acute treatment with red wine polyphenols protects from ischemia-induced excitotoxicity, energy failure and oxidative stress in rats	2008	1239	226-234	Brain Res
Ritz, M. F.; Ratajczak, P.; Curin, Y.; Cam, E.; Mendelowitsch, A.; Pinet, F.; Andriantsitohaina, R.	Chronic treatment with red wine polyphenol compounds mediates neuroprotection in a rat model of ischemic cerebral stroke	2008	138	519-525	J Nutr
Ritz, M. F.; Schmidt, P.; Mendelowitsch, A.	Acute effects of 17beta-estradiol on the extracellular concentration of excitatory amino acids and energy metabolites during transient cerebral ischemia in male rats	2004	1022	157-163	Brain Res
Ritz, M. F.; Schmidt, P.; Mendelowitsch, A.	Effects of isoflurane on glutamate and taurine releases, brain swelling and injury during transient ischemia and reperfusion	2006	116	191-202	Int J Neurosci
Rivas-Arancibia, S.; Dorado-Martinez, C.; Colin-Barenque, L.; Kendrick, K. M.; De La Riva, C.; Guevara-Guzman, R.	Effect of acute ozone exposure on locomotor behavior and striatal function	2003	74	891-900	Pharmacol Biochem Behav
Robelet, S.; Melon, C.; Guillet, B.; Salin, P.; Kerkerian-Le Goff, L.	Chronic L-DOPA treatment increases extracellular glutamate levels and GLT1 expression in the basal ganglia in a rat model of Parkinson's disease	2004	20	1255-1266	Eur J Neurosci
Robert, F.; Bert, L.; Lambas-Senas, L.; Denoroy, L.; Renaud, B.	In vivo monitoring of extracellular noradrenaline and glutamate from rat brain cortex with 2-min microdialysis sampling using capillary electrophoresis with laser-induced fluorescence detection	1996	70	153-162	J Neurosci Methods
Robert, F.; Bert, L.; Parrot, S.; Denoroy, L.; Stoppini, L.; Renaud, B.	Coupling on-line brain microdialysis, precolumn derivatization and capillary electrophoresis for routine minute sampling of O-phosphoethanolamine and excitatory amino acids	1998	817	195-203	J Chromatogr A
Roberto, M.; Schweitzer, P.; Madamba, S. G.; Stouffer, D. G.; Parsons, L. H.; Siggins, G. R.	Acute and chronic ethanol alter glutamatergic transmission in rat central amygdala: an in vitro and in vivo analysis	2004	24	1594-1603	J Neurosci
Robinson, S. E.; Kunko, P. M.; Smith, J. A.; Wallace, M. J.; Mo, Q.; Maher, J. R.	Extracellular aspartate concentration increases in nucleus accumbens after cocaine sensitization	1997	319	31-36	Eur J Pharmacol
Robinson, S.; Freeman, P.; Moore, C.;	Acute and subchronic MPTP administration differentially affects	2003	180	74-87	Exp Neurol

Glutamate					
**Authors**	**Title**	**Year**	**Vol.**	**Pages**	**Journal**
Touchon, J. C.; Krentz, L.; Meshul, C. K.	striatal glutamate synaptic function				
Rocha, L.; Briones, M.; Ackermann, R. F.; Anton, B.; Maidment, N. T.; Evans, C. J.; Engel, J., Jr.	Pentylenetetrazol-induced kindling: early involvement of excitatory and inhibitory systems	1996	26	105-113	Epilepsy Res
Rocher, C.; Bert, L.; Robert, F.; Trouvin, J. H.; Renaud, B.; Jacquot, C.; Gardier, A. M.	Microdialysis monitoring of variations in extracellular levels of serotonin, GABA and excitatory amino acids in the frontal cortex of awake rats in response to a single peripheral or central administration of dexfenfluramine	1996	737	221-230	Brain Res
Rocher, C.; Gardier, A. M.	Effects of repeated systemic administration of d-Fenfluramine on serotonin and glutamate release in rat ventral hippocampus: comparison with methamphetamine using in vivo microdialysis	2001	363	422-428	Naunyn Schmiedebergs Arch Pharmacol
Rocher, C.; Jacquot, C.; Gardier, A. M.	Simultaneous effects of local dexfenfluramine application on extracellular glutamate and serotonin levels in rat frontal cortex: a reverse microdialysis study	1999	38	513-523	Neuropharmacology
Rockhold, R. W.; Liu, N.; Coleman, D.; Commiskey, S.; Shook, J.; Ho, I. K.	The nucleus paragigantocellularis and opioid withdrawal-like behavior	2000	7	270-276	J Biomed Sci
Rodriguez Diaz, M.; Alonso, T. J.; Perdomo Diaz, J.; Gonzalez Hernandez, T.; Castro Fuentes, R.; Sabate, M.; Garcia Dopico, J.	Glial regulation of nonsynaptic extracellular glutamate in the substantia nigra	2005	49	134-142	Glia
Rodriguez, M. J.; Robledo, P.; Andrade, C.; Mahy, N.	In vivo co-ordinated interactions between inhibitory systems to control glutamate-mediated hippocampal excitability	2005	95	651-661	J Neurochem
Rodriguez-Navarro, J. A.; Gonzalo-Gobernado, R.; Herranz, A. S.; Gonzlez-Vigueras, J. M.; Solis, J. M.	High potassium induces taurine release by osmosensitive and osmoresistant mechanisms in the rat hippocampus in vivo	2009	87	208-217	J Neurosci Res
Roehl, A. B.; Zoremba, N.; Kipp, M.; Schiefer, J.; Goetzenich, A.; Bleilevens, C.; Kuehn-Velten, N.; Tolba, R.; Rossaint, R.; Hein, M.	The effects of levosimendan on brain metabolism during initial recovery from global transient ischaemia/hypoxia	2012	12	81	BMC Neurol
Roenker, N. L.; Gudelsky, G. A.; Ahlbrand, R.; Horn, P. S.; Richtand, N. M.	Evidence for involvement of nitric oxide and GABA(B) receptors in MK-801- stimulated release of glutamate in rat prefrontal cortex	2012	63	575-581	Neuropharmacology
Roenker, N. L.; Gudelsky, G.; Ahlbrand,	Effect of paliperidone and risperidone on extracellular glutamate	2011	500	167-171	Neurosci Lett

Glutamate					
**Authors**	**Title**	**Year**	**Vol.**	**Pages**	**Journal**
R.; Bronson, S. L.; Kern, J. R.; Waterman, H.; Richtand, N. M.	in the prefrontal cortex of rats exposed to prenatal immune activation or MK-801				
Ronne Engstrom, E.; Hillered, L.; Flink, R.; Kihlstrom, L.; Lindquist, C.; Nie, J. X.; Olsson, Y.; Silander, H. C.	Extracellular amino acid levels measured with intracerebral microdialysis in the model of posttraumatic epilepsy induced by intracortical iron injection	2001	43	135-144	Epilepsy Res
Ronne-Engstrom, E.; Carlson, H.; Liu, Y.; Ungerstedt, U.; Hillered, L.	Influence of perfusate glucose concentration on dialysate lactate, pyruvate, aspartate, and glutamate levels under basal and hypoxic conditions: a microdialysis study in rat brain	1995	65	257-262	J Neurochem
Rosales, M. G.; Martinez-Fong, D.; Morales, R.; Nunez, A.; Flores, G.; Gongora-Alfaro, J. L.; Floran, B.; Aceves, J.	Reciprocal interaction between glutamate and dopamine in the pars reticulata of the rat substantia nigra: a microdialysis study	1997	80	803-810	Neuroscience
Rosdahl, H.; Samuelsson, A. C.; Ungerstedt, U.; Henriksson, J.	Influence of adrenergic agonists on the release of amino acids from rat skeletal muscle studied by microdialysis	1998	163	349-360	Acta Physiol Scand
Rose, C.; Michalak, A.; Pannunzio, M.; Chatauret, N.; Rambaldi, A.; Butterworth, R. F.	Mild hypothermia delays the onset of coma and prevents brain edema and extracellular brain glutamate accumulation in rats with acute liver failure	2000	31	872-877	Hepatology
Rose, C.; Ytrebo, L. M.; Davies, N. A.; Sen, S.; Nedredal, G. I.; Belanger, M.; Revhaug, A.; Jalan, R.	Association of reduced extracellular brain ammonia, lactate, and intracranial pressure in pigs with acute liver failure	2007	46	1883-1892	Hepatology
Rose, M. E.; Huerbin, M. B.; Melick, J.; Marion, D. W.; Palmer, A. M.; Schiding, J. K.; Kochanek, P. M.; Graham, S. H.	Regulation of interstitial excitatory amino acid concentrations after cortical contusion injury	2002	943	15-22	Brain Res
Rose, M. E.; Huerbin, M. B.; Melick, J.; Marion, D. W.; Palmer, A. M.; Schiding, J. K.; Kochanek, P. M.; Graham, S. H.	Regulation of interstitial excitatory amino acid concentrations after cortical contusion injury	2002	935	40-46	Brain Res
Rosi, S.; Giovannini, M. G.; Lestage, P. J.; Munoz, C.; Corte, L. D.; Pepeu, G.	S 18986, a positive modulator of AMPA receptors with cognition-enhancing properties, increases ACh release in the hippocampus of young and aged rat	2004	361	120-123	Neurosci Lett
Rossell, S.; Gonzalez, L. E.; Hernandez, L.	One-second time resolution brain microdialysis in fully awake rats. Protocol for the collection, separation and sorting of nanoliter dialysate volumes	2003	784	385-393	J Chromatogr B Analyt Technol Biomed Life Sci
Rossetti, Z. L.; Carboni, S.	Ethanol withdrawal is associated with increased extracellular	1995	283	177-183	Eur J Pharmacol

Glutamate					
**Authors**	**Title**	**Year**	**Vol.**	**Pages**	**Journal**
	glutamate in the rat striatum				
Rossetti, Z. L.; Carboni, S.; Fadda, F.	Glutamate-induced increase of extracellular glutamate through N-methyl-D-aspartate receptors in ethanol withdrawal	1999	83	1135-1140	Neuroscience
Rossetti, Z. L.; Marcangione, C.; Wise, R. A.	Increase of extracellular glutamate and expression of Fos-like immunoreactivity in the ventral tegmental area in response to electrical stimulation of the prefrontal cortex	1998	70	1503-1512	J Neurochem
Rowley, H. L.; Marsden, C. A.; Martin, K. F.	Generalised seizure-induced changes in rat hippocampal glutamate but not GABA release are potentiated by repeated seizures	1997	234	143-146	Neurosci Lett
Rowley, H. L.; Marsden, C. A.; Martin, K. F.	Differential effects of phenytoin and sodium valproate on seizure-induced changes in gamma-aminobutyric acid and glutamate release in vivo	1995	294	541-546	Eur J Pharmacol
Rowley, H. L.; Martin, K. F.; Marsden, C. A.	Determination of in vivo amino acid neurotransmitters by high-performance liquid chromatography with o-phthalaldehyde-sulphite derivatisation	1995	57	93-99	J Neurosci Methods
Rowley, H. L.; Martin, K. F.; Marsden, C. A.	Decreased GABA release following tonic-clonic seizures is associated with an increase in extracellular glutamate in rat hippocampus in vivo	1995	68	415-422	Neuroscience
Rozza, A.; Masoero, E.; Favalli, L.; Lanza, E.; Govoni, S.; Rizzo, V.; Montalbetti, L.	Influence of different anaesthetics on extracellular aminoacids in rat brain	2000	101	165-169	J Neurosci Methods
Ruda-Kucerova, J.; Amchova, P.; Havlickova, T.; Jerabek, P.; Babinska, Z.; Kacer, P.; Syslova, K.; Sulcova, A.; Sustkova-Fiserova, M.	Reward related neurotransmitter changes in a model of depression: An in vivo microdialysis study	2015	16	521-535	World J Biol Psychiatry
Runnerstam, M.; Bao, F.; Huang, Y.; Shi, J.; Gutierrez, E.; Hamberger, A.; Hansson, H. A.; Viano, D.; Haglid, K.	A new model for diffuse brain injury by rotational acceleration: II. Effects on extracellular glutamate, intracranial pressure, and neuronal apoptosis	2001	18	259-273	J Neurotrauma
Runnerstam, M.; Stephensen, H.; von Essen, C.; Nystrom, B.; Hamberger, A.	Subarachnoid blood infusion versus raised intracranial pressure: effects on the amino acid pattern in the extracellular fluid of the rabbit hippocampus	1999	21	404-408	Neurol Res
Saellstroem Baum, S.; Huebner, A.; Krimphove, M.; Morgenstern, R.; Badawy, A. A.; Spies, C. D.	Nicotine stimulation on extracellular glutamate levels in the nucleus accumbens of ethanol-withdrawn rats in vivo	2006	30	1414-1421	Alcohol Clin Exp Res

Glutamate					
**Authors**	**Title**	**Year**	**Vol.**	**Pages**	**Journal**
Saito, H.; Matsumoto, M.; Togashi, H.; Yoshioka, M.	Ischemia stimulates cortical 5-hydroxytryptamine efflux in rat brain: An in vivo microdialysis study	1994	10	91-97	Biog Amines
Saito, N.; Chang, C.; Kawai, K.; Joo, F.; Nowak Jr, T. S.; Mies, G.; Ikeda, J.; Nagashima, G.; Ruetzler, C.; Lohr, J.	Role of neuroexcitation in development of blood-brain barrier and oedematous changes following cerebral ischaemia and traumatic brain injury	1990	51	186-188	Acta Neurochirurgica
Saito, R.; Graf, R.; Hubel, K.; Taguchi, J.; Rosner, G.; Fujita, T.; Heiss, W. D.	Halothane, but not alpha-chloralose, blocks potassium-evoked cortical spreading depression in cats	1995	699	109-115	Brain Res
Saitoh, A.; Ohashi, M.; Suzuki, S.; Tsukagoshi, M.; Sugiyama, A.; Oka, J. I.; Inagaki, M.; Yamada, M.	Activation of the prelimbic medial prefrontal cortex induces anxiety-like behaviors via N-Methyl-D-aspartate receptor-mediated glutamatergic neurotransmission in mice	2014	92	1044-1053	J Neurosci Res
Sakowitz, O. W.; Unterberg, A. W.; Stover, J. F.	Neuronal activity determined by quantitative EEG and cortical microdialysis is increased following controlled cortical impact injury in rats	2002	81	221-223	Acta Neurochir Suppl
Salamone, A.; Mura, E.; Zappettini, S.; Grilli, M.; Olivero, G.; Preda, S.; Govoni, S.; Marchi, M.	Inhibitory effects of beta-amyloid on the nicotinic receptors which stimulate glutamate release in rat hippocampus: the glial contribution	2014	723	314-321	Eur J Pharmacol
Salazar, P.; Tapia, R.	Allopregnanolone potentiates the glutamate-mediated seizures induced by 4-aminopyridine in rat hippocampus in vivo	2012	37	596-603	Neurochem Res
Saleh, T. M.; Bauce, L. G.; Pittman, Q. J.	Glutamate release in parabrachial nucleus and baroreflex alterations after vagal afferent activation	1997	272	R1631-R1640	Am J Physiol Regul Integr Comp Physiol
Saleh, T. M.; Connell, B. J.; Legge, C.; Cribb, A. E.	Estrogen attenuates neuronal excitability in the insular cortex following middle cerebral artery occlusion	2004	1018	119-129	Brain Res
Saleh, T. M.; Connell, B. J.; McQuaid, T.; Cribb, A. E.	Estrogen-induced neurochemical and electrophysiological changes in the parabrachial nucleus of the male rat	2003	990	58-65	Brain Res
Samuel, D.; Pisano, P.; Forni, C.; Nieoullon, A.; Goff, L. K. L.	Involvement of the glutamatergic metabotropic receptors in the regulation of glutamate uptake and extracellular excitatory amino acid levels in the striatum of chloral hydrate-anesthetized rats	1996	739	156-162	Brain Re
Samuelsson, C.; Kumlien, E.; Elfving, A.; Lindholm, D.; Ronne-Engstrom, E.	The effects of PBN (phenyl-butyl-nitrone) on GLT-1 levels and on the extracellular levels of amino acids and energy metabolites in a model of iron-induced posttraumatic epilepsy	2003	56	165-173	Epilepsy Res
Sanchez-Carbente, M. D. R.; Massieu, L.	Transient inhibition of glutamate uptake in vivo induces neurodegeneration when energy metabolism is impaired	1999	72	129-138	J Neurochem
Sanchez-Huerta, K. B.; Montes, S.; Perez-	Hypothyroidism reduces glutamate-synaptic release by ouabain	2012	90	905-912	J Neurosci Res

Glutamate					
**Authors**	**Title**	**Year**	**Vol.**	**Pages**	**Journal**
Severiano, F.; Alva-Sanchez, C.; Rios, C.; Pacheco-Rosado, J.	depolarization in rat CA3-hippocampal region				
Sandberg, M.; Butcher, S. P.; Hagberg, H.	Extracellular overflow of neuroactive amino acids during severe insulin-induced hypoglycemia: in vivo dialysis of the rat hippocampus	1986	47	178-184	J Neurochem
Sandlin, Z. D.; Shou, M.; Shackman, J. G.; Kennedy, R. T.	Microfluidic electrophoresis chip coupled to microdialysis for in vivo monitoring of amino acid neurotransmitters	2005	77	7702-7708	Anal Chem
Sandusky, L. A.; Flint, R. W.; McNay, E. C.	Elevated glucose metabolism in the amygdala during an inhibitory avoidance task	2013	245	83-87	Behav Brain Res
Sano, K.; Mishima, K.; Koushi, E.; Orito, K.; Egashira, N.; Irie, K.; Takasaki, K.; Katsurabayashi, S.; Iwasaki, K.; Uchida, N.; Egawa, T.; Kitamura, Y.; Nishimura, R.; Fujiwara, M.	Delta 9-tetrahydrocannabinol-induced catalepsy-like immobilization is mediated by decreased 5-HT neurotransmission in the nucleus accumbens due to the action of glutamate-containing neurons	2008	151	320-328	Neuroscience
Santamaria, A.; Arias, H. R.	Neurochemical and behavioral effects elicited by bupropion and diethylpropion in rats	2010	211	132-139	Behav Brain Res
Santana Gomez, C.	Evaluation of the hippocampal release of gaba and glutamate as result of status epilepticus induced by lithium-pilocarpine administration in rats	2014	14	148	Epilepsy Curr
Santana-Gomez, C. E.; Alcantara-Gonzalez, D.; Luna-Munguia, H.; Banuelos-Cabrera, I.; Magdaleno-Madrigal, V.; Fernandez-Mas, R.; Besio, W.; Rocha, L.	Transcranial focal electrical stimulation reduces the convulsive expression and amino acid release in the hippocampus during pilocarpine-induced status epilepticus in rats	2015	49	33-39	Epilepsy Behav
Santos-Fandila, A.; Zafra-Gomez, A.; Barranco, A.; Navalon, A.; Rueda, R.; Ramirez, M.	Quantitative determination of neurotransmitters, metabolites and derivates in microdialysates by UHPLC-tandem mass spectrometry	2013	144	79-89	Talanta
Sanz, B.; Exposito, I.; Mora, F.	Effects of neurotensin on the release of glutamic acid in the prefrontal cortex and striatum of the rat	1993	4	1194-1196	Neuroreport
Sato, S.; Takeda, Y.; Mizoue, R.; Morimatsu, H.	Intraischemic nasopharyngeal cooling decreases extracellular glutamate level and the cerebral blood flow threshold for repolarization	2013	128	/	Circulation. Conference: American Heart Association
Satoh, M.; Asai, S.; Katayama, Y.; Kohno,	Real-time monitoring of glutamate transmitter release with anoxic	1999	822	142-148	Brain Res

Glutamate					
**Authors**	**Title**	**Year**	**Vol.**	**Pages**	**Journal**
T.; Ishikawa, K.	depolarization during anoxic insult in rat striatum				
Saul; skaia, N. B.; Mikhailova, M. O.	[Vesicular and nonvesicular glutamate release in the nucleus accumbens during a forced switch in behavioral strategy]	2004	54	526-532	Zh Vyssh Nerv Deiat Im I P Pavlova
Saul; skaia, N. B.; Mikhailova, M. O.	[Enhancement of glutamate release in the extracellular space of nucleus accumbens during presentation of aversive or neutral stimuli instead of food reinforcement]	2003	53	170-175	Zh Vyssh Nerv Deiat Im I P Pavlova
Saul; skaia, N. B.; Mikhailova, M. O.	[Th effect of D2-dopamine receptor blockade on glutamate release into extracellular space of Nucleus accumbens during food reinforcement]	2001	51	254-255	Zh Vyssh Nerv Deiat Im I P Pavlova
Saul; skaia, N. B.; Mikhailova, M. O.	[Effects of motivational and emotional factors on glutamate release in the nucleus accumbens of rats during feeding]	2001	87	858-864	Ross Fiziol Zh Im I M Sechenova
Saul; skaia, N. B.; Mikhailova, M. O.; Pudovkina, O. L.; Gorbachevskaia, A. I.	[Changes in glutamate release in the rat nucleus accumbens during food and pain reinforcement]	2000	50	124-132	Zh Vyssh Nerv Deiat Im I P Pavlova
Saul; skaia, N. B.; Solov; eva, N. A.	[Tetrodotoxine-dependent glycine release in the rat nucleus accumbens during correction of feeding behaviour]	2004	90	833-839	Ross Fiziol Zh Im I M Sechenova
Saul; skaya, N. B.; Mikhailova, M. O.	Vesicular and non-vesicular glutamate release in the nucleus accumbens in conditions of a forced change of behavioral strategy	2005	35	677-683	Neurosci Behav Physiol
Saul; skaya, N. B.; Mikhailova, M. O.	Increased glutamate release into the intercellular space of the nucleus accumbens (N. accumbens) during substitution of food reinforcement with aversive or neutral stimuli	2004	34	109-113	Neurosci Behav Physiol
Saul; skaya, N. B.; Mikhailova, M. O.	The effects of motivational and emotional factors in glutamate release in the nucleus accumbens of the rat brain during food consumption	2003	33	151-156	Neurosci Behav Physiol
Saul; skaya, N. B.; Pudovkina, O. L.; Gorbachevskaya, A. I.	Increases in glutamate release in the nucleus accumbens in rats with lesions to the hippocampal formation during an emotional conditioned response	2000	30	407-413	Neurosci Behav Physiol
Saul; skaya, N. B.; Solov; eva, N. A.; Savel; ev, S. A.	Glutamate release in the nucleus accumbens during competitive presentation of aversive and appetitive stimuli	2006	36	247-252	Neurosci Behav Physiol
Saulskaya, N. B.; Mikhailova, M. O.	Feeding-induced decrease in extracellular glutamate level in the rat nucleus accumbens: dependence on glutamate uptake	2002	112	791-801	Neuroscience
Saulskaya, N. B.; Soloviova, N. A.	Tetrodotoxin-dependent glutamate release in the rat nucleus	2004	140	15-21	J Neurosci Methods

Glutamate					
**Authors**	**Title**	**Year**	**Vol.**	**Pages**	**Journal**
	accumbens during concurrent presentation of appetitive and conditioned aversive stimuli				
Saulskaya, N.; Marsden, C. A.	Extracellular glutamate in the nucleus accumbens during a conditioned emotional response in the rat	1995	698	114-120	Brain Res
Schaefer, F.; Vogel, M.; Kerkhoff, G.; Woitzik, J.; Daschner, M.; Mehls, O.	Experimental uremia affects hypothalamic amino acid neurotransmitter milieu	2001	12	1218-1227	J Am Soc Nephrol
Schallier, A.; Smolders, I.; Van Dam, D.; Loyens, E.; De Deyn, P. P.; Michotte, A.; Michotte, Y.; Massie, A.	Region- and age-specific changes in glutamate transport in the AbetaPP23 mouse model for Alzheimer's disease	2011	24	287-300	J Alzeihemers Dis
Schallier, A.; Vermoesen, K.; Loyens, E.; Van Liefferinge, J.; Michotte, Y.; Smolders, I.; Massie, A.	L-Theanine intake increases threshold for limbic seizures but decreases threshold for generalized seizures	2013	16	78-82	Nutr Neurosci
Schechter, L. E.; Lin, Q.; Smith, D. L.; Zhang, G.; Shan, Q.; Platt, B.; Brandt, M. R.; Dawson, L. A.; Cole, D.; Bernotas, R.; Robichaud, A.; Rosenzweig-Lipson, S.; Beyer, C. E.	Neuropharmacological profile of novel and selective 5-HT6 receptor agonists: WAY-181187 and WAY-208466	2008	33	1323-1335	Neuropsychopharmacology
Scheller, D. K.; De Ryck, M.; Kolb, J.; Szathmary, S.; van Reempts, J.; Clincke, G.; Tegtmeier, F.	Lubeluzole blocks increases in extracellular glutamate and taurine in the peri-infarct zone in rats	1997	338	243-251	Eur J Pharmacol
Scheller, D.; Szathmary, S.; Kolb, J.; Tegtmeier, F.	Observations on the relationship between the extracellular changes of taurine and glutamate during cortical spreading depression, during ischemia, and within the area surrounding a thrombotic infarct	2000	19	571-583	Amino Acids
Schilstrom, B.; Fagerquist, M. V.; Zhang, X.; Hertel, P.; Panagis, G.; Nomikos, G. G.; Svensson, T. H.	Putative role of presynaptic alpha7* nicotinic receptors in nicotine stimulated increases of extracellular levels of glutamate and aspartate in the ventral tegmental area	2000	38	375-383	Synapse
Schloesser, R.; Jimenez, D. V.; Hardy, N. F.; Paredes, D.; Catlow, B. J.; Manji, H. K.; McKay, R. D.; Martinowich, K.	Atrophy of pyramidal neurons and increased stress-induced glutamate levels in ca3 following suppression of adult neurogenesis	2013	1	150S	Biological Psychiatry
Schmidt, M.; Marx, T.; Gloggl, E.; Reinelt, H.; Schirmer, U.	Xenon attenuates cerebral damage after ischemia in pigs	2005	102	929-936	Anesthesiology
Schober, A.; Warenits, A. M.; Testori, C.;	Microdialysis Assessment of Cerebral Perfusion during Cardiac	2016	11	e0155303	PloS One

Glutamate					
**Authors**	**Title**	**Year**	**Vol.**	**Pages**	**Journal**
Weihs, W.; Hosmann, A.; Hogler, S.; Sterz, F.; Janata, A.; Scherer, T.; Magnet, I. A.; Ettl, F.; Laggner, A. N.; Herkner, H.; Zeitlinger, M.	Arrest, Extracorporeal Life Support and Cardiopulmonary Resuscitation in Rats - A Pilot Trial				
Schubert, G. A.; Poli, S.; Mendelowitsch, A.; Schilling, L.; Thome, C.	Hypothermia reduces early hypoperfusion and metabolic alterations during the acute phase of massive subarachnoid hemorrhage: a laser-Doppler-flowmetry and microdialysis study in rats	2008	25	539-548	J Neurotrauma
Schwarcz, R.	Cognitive enhancement through inhibition of kynurenic acid synthesis	2015	40	S49-S50	Neuropsychopharmacology
Schwarzkopf, T. M.; Hagl, S.; Eckert, G. P.; Klein, J.	Neuroprotection by bilobalide in ischemia: improvement of mitochondrial function	2013	68	584-589	Pharmazie
Schwarzkopf, T. M.; Klein, J.	Neuroprotection by bilobalide: What is the mode of action?	2006	372	S87-S88	Naunyn Schmiedeberg's Archives of Pharmacology. Conference: Abstracts of the 78th Annual Meeting of the Deutsche Gesellschaft fur Experimentelle und Klinische Pharmakologie und Toxikologie
Schwarzkopf, T. M.; Koch, K. A.; Klein, J.	Neurodegeneration after transient brain ischemia in aged mice: beneficial effects of bilobalide	2013	1529	178-187	Brain Res
Schwarzkopf, T. M.; Koch, K.; Klein, J.	Reduced severity of ischemic stroke and improvement of mitochondrial function after dietary treatment with the anaplerotic substance triheptanoin	2015	300	201-209	Neuroscience
Sciotti, V. M.; Roche, F. M.; Grabb, M. C.; Van Wylen, D. G.	Adenosine receptor blockade augments interstitial fluid levels of excitatory amino acids during cerebral ischemia	1992	12	646-655	J Cereb Blood Flow Metab
See, R. E.; Chapman, M. A.	Chronic haloperidol, but not clozapine, produces altered oral movements and increased extracellular glutamate in rats	1994	263	269-276	Eur J Pharmacol
See, R. E.; Lynch, A. M.	Chronic haloperidol potentiates stimulated glutamate release in caudate putamen, but not prefrontal cortex	1995	6	1795-1798	Neuroreport
See, R. E.; Lynch, A. M.	Duration-dependent increase in striatal glutamate following prolonged fluphenazine administration in rats	1996	308	279-282	Eur J Pharmacol

Glutamate					
**Authors**	**Title**	**Year**	**Vol.**	**Pages**	**Journal**
Segieth, J.; Getting, S. J.; Biggs, C. S.; Whitton, P. S.	Nitric oxide regulates excitatory amino acid release in a biphasic manner in freely moving rats	1995	200	101-104	Neurosci Lett
Segovia, G.; Del Arco, A.; Mora, F.	Effects of aging on the interaction between glutamate, dopamine, and GABA in striatum and nucleus accumbens of the awake rat	1999	73	2063-2072	J Neurochem
Segovia, G.; Del Arco, A.; Mora, F.	Endogenous glutamate increases extracellular concentrations of dopamine, GABA, and taurine through NMDA and AMPA/kainate receptors in striatum of the freely moving rat: a microdialysis study	1997	69	1476-1483	J Neurochem
Segovia, G.; Del Arco, A.; Mora, F.	Role of glutamate receptors and glutamate transporters in the regulation of the glutamate-glutamine cycle in the awake rat	1999	24	779-783	Neurochem Res
Segovia, G.; Del Arco, A.; Prieto, L.; Mora, F.	Glutamate-glutamine cycle and aging in striatum of the awake rat: effects of a glutamate transporter blocker	2001	26	37-41	Neurochem Res
Segovia, G.; Mora, F.	Role of nitric oxide in modulating the release of dopamine, glutamate, and GABA in striatum of the freely moving rat	1998	45	275-279	Brain Res Bull
Segovia, G.; Mora, F.	Involvement of NMDA and AMPA/kainate receptors in the effects of endogenous glutamate on extracellular concentrations of dopamine and GABA in the nucleus accumbens of the awake rat	2001	54	153-157	Brain Res Bull
Segovia, G.; Porras, A.; Mora, F.	Effects of 4-aminopyridine on extracellular concentrations of glutamate in striatum of the freely moving rat	1997	22	1491-1497	Neurochem Res
Segovia, G.; Yague, A. G.; Garcia-Verdugo, J. M.; Mora, F.	Environmental enrichment promotes neurogenesis and changes the extracellular concentrations of glutamate and GABA in the hippocampus of aged rats	2006	70	8-14	Brain Res Bull
Seki, Y.; Feustel, P. J.; Keller Jr, R. W.; Tranmer, B. I.; Kimelberg, H. K.	Inhibition of ischemia-induced glutamate release in rat striatum by dihydrokinate and an anion channel blocker	1999	30	433-440	Stroke
Sekiguchi, K.; Kanno, H.; Yamaguchi, T.; Ikarashi, Y.; Kase, Y.	Ameliorative effect of yokukansan on vacuous chewing movement in haloperidol-induced rat tardive dyskinesia model and involvement of glutamatergic system	2012	89	151-158	Brain Res Bull
Selim, M.; Bradberry, C. W.	Effect of ethanol on extracellular 5-HT and glutamate in the nucleus accumbens and prefrontal cortex: comparison between the Lewis and Fischer 344 rat strains	1996	716	157-164	Brain Res
Semba, J.; Miyoshi, R.; Kanazawa, M.; Kito, S.	Effect of systemic dexamethasone on extracellular amino acids, GABA and acetylcholine release in rat hippocampus using in vivo microdialysis	1995	10	17-21	Funct Neurol

Glutamate					
**Authors**	**Title**	**Year**	**Vol.**	**Pages**	**Journal**
Semba, J.; Wakuta, M. S.	Regional differences in the effects of glutamate uptake inhibitor L-trans-pyrrolidine-2,4-dicarboxylic acid on extracellular amino acids and dopamine in rat brain: an in vivo microdialysis study	1998	31	399-404	Gen Pharmacol
Sepulveda, M. J.; Hernandez, L.; Rada, P.; Tucci, S.; Contreras, E.	Effect of precipitated withdrawal on extracellular glutamate and aspartate in the nucleus accumbens of chronically morphine-treated rats: an in vivo microdialysis study	1998	60	255-262	Pharmacol Biochem Behav
Serra, P. A.; Susini, G.; Rocchitta, G.; Migheli, R.; Dessanti, G.; Miele, E.; Desole, M. S.; Miele, M.	Effects of sufentanil on the release and metabolism of dopamine and ascorbic acid and glutamate release in the striatum of freely moving rats	2003	344	9-12	Neurosci Lett
Shah, A. J.; de Biasi, V.; Taylor, S. G.; Roberts, C.; Hemmati, P.; Munton, R.; West, A.; Routledge, C.; Camilleri, P.	Development of a protocol for the automated analysis of amino acids in brain tissue samples and microdialysates	1999	735	133-140	J Chromatogr B Biomed Sci Appl
Shakil, S. S.; Holmer, H. K.; Moore, C.; Abernathy, A. T.; Jakowec, M. W.; Petzinger, G. M.; Meshul, C. K.	High and low responders to novelty show differential effects in striatal glutamate	2005	58	200-207	Synapse
Shang, Y.; Gu, P. F.; Shang, Y.; Li, Y.	[Penehyclidine hydrochloride inhibits glutamate release and related research in global brain ischemia/reperfusion rats]	2011	27	353-356	Zhongguo Ying Yong Sheng Li Xue Za Zhi
Shea, S. D.; Smith, I. M.; McCabe, O. M.; Cronin, M. M.; Walsh, D. M.; Connor, W. T.	Intracerebroventricular Administration of Amyloid beta-protein Oligomers Selectively Increases Dorsal Hippocampal Dialysate Glutamate Levels in the Awake Rat	2008	8	7428-7437	Sensors (Basel)
Shea, T. J.; Weber, P. L.; Bammel, B. P.; Lunte, C. E.; Lunte, S. M.; Smyth, M. R.	Monitoring excitatory amino acid release in vivo by microdialysis with capillary electrophoresis-electrochemistry	1992	608	189-195	J Chromatogr
Shen, D.; Wang, Y.; Zhang, R.; Wu, Z.; Yuan, W.; Su, D.; Wang, W.	Overexpression of angiotensin-converting enzyme 2 attenuates tonically acting glutamatergic inputs to the rostral ventrolateral medulla in hypertensive rats	2012	60	/	Hypertension. Conference: High Blood Pressure Research
Shen, E. Y.; Lai, Y. J.	In vivo microdialysis study of excitatory and inhibitory amino acid levels in the hippocampus following penicillin-induced seizures in mature rats	2002	43	313-318	Acta Paediatr Taiwan
Shen, H. W.; Scofield, M. D.; Boger, H.; Hensley, M.; Kalivas, P. W.	Synaptic glutamate spillover due to impaired glutamate uptake mediates heroin relapse	2014	34	5649-5657	J Neurosci
Shen, H.; Chen, G. J.; Harvey, B. K.; Bickford, P. C.; Wang, Y.	Inosine reduces ischemic brain injury in rats	2005	36	654-659	Stroke
Shen, L. M.; Chen, Y. C.	[Changes of inhibitory amino acid in the monkey frontal cortex	1999	51	681-686	Sheng Li Xue Bao

Glutamate					
Authors	Title	Year	Vol.	Pages	Journal
	during a figure recognition guided sequential movement task]				
Sheng, W.; Hang, H. W.; Ruan, D. Y.	In vivo microdialysis study of the relationship between lead-induced impairment of learning and neurotransmitter changes in the hippocampus	2005	20	233-240	Environ Toxicol Pharmacol
Shi, H.; Yang, X.; Zhao, H.; Zhang, S.; Zu, J.; Zhang, W.; Shen, X.; Cui, G.; Hua, F.; Yan, C.	Ranitidine reduced levodopa-induced dyskinesia by remodeling neurochemical changes in hemiparkinsonian model of rats	2015	11	1331-1337	Neuropsychiatr Dis Treat
Shi, L.; Yang, A. C.; Li, J. J.; Meng, D. W.; Jiang, B.; Zhang, J. G.	Favorable modulation in neurotransmitters: effects of chronic anterior thalamic nuclei stimulation observed in epileptic monkeys	2015	265	94-101	Exp Neurol
Shibanoki, S.; Kogure, M.; Sugahara, M.; Ishikawa, K.	Effect of systemic administration of N-methyl-D-aspartic acid on extracellular taurine level measured by microdialysis in the hippocampal CA1 field and striatum of rats	1993	61	1698-1704	J Neurochem
Shima, K.; Shirotani, T.; Chigasaki, H.	Delayed neuronal damage following focal ischemic injury in stroke-prone spontaneously hypertensive rats	1996	67	24-27	Acta Neurochir Suppl
Shimada, N.; Graf, R.; Rosner, G.; Heiss, W. D.	Ischemia-induced accumulation of extracellular amino acids in cerebral cortex, white matter, and cerebrospinal fluid	1993	60	66-71	J Neurochem
Shimada, N.; Graf, R.; Rosner, G.; Wakayama, A.; George, C. P.; Heiss, W. D.	Ischemic flow threshold for extracellular glutamate increase in cat cortex	1989	9	603-606	J Cereb Blood Flow Metab
Shimizu, H.; Graham, S. H.; Chang, L. H.; Mintorovitch, J.; James, T. L.; Faden, A. I.; Weinstein, P. R.	Relationship between extracellular neurotransmitter amino acids and energy metabolism during cerebral ischemia in rats monitored by microdialysis and in vivo magnetic resonance spectroscopy	1993	605	33-42	Brain Res
Shimizu, K.; Matsubara, K.; Ohtaki, K.; Fujimaru, S.; Saito, O.; Shiono, H.	Paraquat induces long-lasting dopamine overflow through the excitotoxic pathway in the striatum of freely moving rats	2003	976	243-252	Brain Res
Shimizu, K.; Matsubara, K.; Uezono, T.; Kimura, K.; Shiono, H.	Reduced dorsal hippocampal glutamate release significantly correlates with the spatial memory deficits produced by benzodiazepines and ethanol	1998	83	701-706	Neuroscience
Shimizu-Sasamata, M.	Attenuated neurotransmitter release and spreading depression-like depolarizations after focal ischemia in mutant mice with disrupted type I nitric oxide synthase gene	1998	18	9564-9571	J Neurosci
Shin, C. B.; Serchia, M. M.; Shahin, J. R.; Ruppert-Majer, M. A.; Kippin, T. E.;	Incubation of cocaine-craving relates to glutamate over-flow within ventromedial prefrontal cortex	2016	102	103-110	Neuropharmacology

Glutamate					
Authors	Title	Year	Vol.	Pages	Journal
Szumlinski, K. K.					
Shin, K. H.; Kim, H. G.; Choi, S. H.; Cho, S. H.; Chun, Y. S.; Chun, B. G.	Changes of glutamate and polyamine levels of hippocampal microdialysates in response to occlusion of both carotid arteries in Mongolian gerbils	1994	30	273-289	Korean J Pharmacol
Shin, R. S.; Anisman, H.; Merali, Z.; McIntyre, D. C.	Amygdala amino acid and monoamine levels in genetically Fast and Slow kindling rat strains during massed amygdala kindling: a microdialysis study	2004	20	185-194	Eur J Neurosci
Shin, R. S.; Anisman, H.; Merali, Z.; McIntyre, D. C.	Changes in extracellular levels of amygdala amino acids in genetically fast and slow kindling rat strains	2002	946	31-42	Brain Res
Shioda, K.; Nisijima, K.; Kasai, M.; Yoshino, T.; Kato, S.	Risperidone attenuates the increase of extracellular nitric oxide and glutamate levels in serotonin syndrome animal models	2012	528	22-26	Neurosci Lett
Shioda, K.; Nisijima, K.; Yoshino, T.; Kato, S.	Effect of risperidone on acute methamphetamine-induced hyperthermia in rats	2010	111	241-249	Drug Alcohol Depend
Shioda, K.; Nisijima, K.; Yoshino, T.; Kato, S.	Extracellular serotonin, dopamine and glutamate levels are elevated in the hypothalamus in a serotonin syndrome animal model induced by tranylcypromine and fluoxetine	2004	28	633-640	Prog Neuropsychopharmacol Biol Psychiatry
Shiraishi, M.; Kamiyama, Y.; Huttemeier, P. C.; Benveniste, H.	Extracellular glutamate and dopamine measured by microdialysis in the rat striatum during blockade of synaptic transmission in anesthetized and awake rats	1997	759	221-227	Brain Res
Shiraishi, N.; Oi, K.	The influence of intravenous anesthetics on nitric oxide production in the rat dorsal hippocampus. [Japanese]	2000	28	27-35	J Jpn Dent Soc Anesthesiol
Shirotani, T.; Shima, K.; Chigasaki, H.	In vivo studies of extracellular metabolites in the striatum after distal middle cerebral artery occlusion in stroke-prone spontaneously hypertensive rats	1995	26	878-384	Stroke
Shoblock, J. R.; Sullivan, E. B.; Maisonneuve, I. M.; Glick, S. D.	Neurochemical and behavioral differences between d-methamphetamine and d-amphetamine in rats	2003	165	359-369	Psychopharmacology(Berl)
Shou, M.; Smith, A. D.; Shackman, J. G.; Peris, J.; Kennedy, R. T.	In vivo monitoring of amino acids by microdialysis sampling with on-line derivatization by naphthalene-2,3-dicarboxyaldehyde and rapid micellar electrokinetic capillary chromatography	2004	138	189-197	J Neurosci Methods
Shuaib, A.	The role of taurine in cerebral ischemia: studies in transient forebrain ischemia and embolic focal ischemia in rodents	2003	526	421-431	Adv Exp Med Biol
Shuaib, A.; Ijaz, M. S.; Miyashita, H.; Hussain, S.; Kanthan, R.	GABA and glutamate levels in the substantia nigra reticulata following repetitive cerebral ischemia in gerbils	1997	147	311-315	Exp Neurol

Glutamate					
**Authors**	**Title**	**Year**	**Vol.**	**Pages**	**Journal**
Shuaib, A.; Ijaz, M. S.; Waqar, T.; Voll, C.; Kanthan, R.; Miyashita, H.; Liu, L.	Insulin elevates hippocampal GABA levels during ischemia. This is independent of its hypoglycemic effect	1995	67	809-814	Neuroscience
Shuaib, A.; Ijaz, S.; Hemmings, S.; Galazka, P.; Ishaqzay, R.; Liu, L.; Rabindran, J.; Miyashita, H.	Decreased glutamate release during hypothyroidism may contribute to protection in cerebral ischemia	1994	128	260-265	Exp Neurol
Shuaib, A.; Mahmood, R. H.; Wishart, T.; Kanthan, R.; Murabit, M. A.; Ijaz, S.; Miyashita, H.; Howlett, W.	Neuroprotective effects of lamotrigine in global ischemia in gerbils. A histological, in vivo microdialysis and behavioral study	1995	702	199-206	Brain Res
Sierra-Paredes, G.; Galan-Valiente, J.; Vazquez-Illanes, M. D.; Aguilar-Veiga, E.; Sierra-Marcuno, G.	Effect of ionotropic glutamate receptors antagonists on the modifications in extracellular glutamate and aspartate levels during picrotoxin seizures: a microdialysis study in freely moving rats	2000	37	377-386	Neurochem Int
Sierra-Paredes, G.; Galan-Valiente, J.; Vazquez-Illanes, M. D.; Aguilar-Veiga, E.; Soto-Otero, R.; Mendez-Alvarez, E.; Sierra-Marcuno, G.	Extracellular amino acids in the rat hippocampus during picrotoxin threshold seizures in chronic microdialysis experiments	1998	248	53-56	Neurosci Lett
Sierra-Paredes, G.; Loureiro, A. I.; Wright, L. C.; Sierra-Marcuno, G.; Soares-da-Silva, P.	Effects of eslicarbazepine acetate on acute and chronic latrunculin A-induced seizures and extracellular amino acid levels in the mouse hippocampus	2014	15	134	BMC Neurosci
Sierra-Paredes, G.; Oreiro-Garcia, M. T.; Vazquez-Illanes, M. D.; Sierra-Marcuno, G.	Effect of eslicarbazepine acetate (BIA 2-093) on latrunculin A-induced seizures and extracellular amino acid concentrations in the rat hippocampus	2007	77	36-43	Epilepsy Res
Silva, E.; Hernandez, L.	[Extracellular aminoacids in the amygdala and nucleus accumbens in the rat during acute pain]	2007	48	213-224	Invest Clin
Silva, E.; Hernandez, L.; Contreras, Q.; Guerrero, F.; Alba, G.	Noxious stimulation increases glutamate and arginine in the periaqueductal gray matter in rats: a microdialysis study	2000	87	131-135	Pain
Silva, E.; Hernandez, L.; Quinonez, B.; Gonzalez, L. E.; Colasante, C.	Selective amino acids changes in the medial and lateral preoptic area in the formalin test in rats	2004	124	395-404	Neuroscience
Silva, E.; Quinones, B.; Freund, N.; Gonzalez, L. E.; Hernandez, L.	Extracellular glutamate, aspartate and arginine increase in the ventral posterolateral thalamic nucleus during nociceptive stimulation	2001	923	45-49	Brain Res
Silva, E.; Quinones, B.; Paez, X.; Hernandez, L.	[Effect of a simple morphine system injection in some aminoacids in the anterior cingulate cortex during acute pain]	2008	49	511-522	Invest Clin

Glutamate					
**Authors**	**Title**	**Year**	**Vol.**	**Pages**	**Journal**
Silverstein, F. S.; Naik, B.	Effect of depolarization on striatal amino acid efflux in perinatal rats: an in vivo microdialysis study	1991	128	133-136	Neurosci Lett
Silverstein, F. S.; Naik, B.; Simpson, J.	Hypoxia-ischemia stimulates hippocampal glutamate efflux in perinatal rat brain: an in vivo microdialysis study	1991	30	587-590	Pediatr Res
Silverstein, F. S.; Simpson, J.; Gordon, K. E.	Hypoglycemia alters striatal amino acid efflux in perinatal rats: an in vivo microdialysis study	1990	28	516-521	Ann Neurol
Simon, R. P.; Chen, J.; Graham, S. H.	GM1 ganglioside treatment of focal ischemia: a dose-response and microdialysis study	1993	265	24-29	J Pharmacol Exp Ther
Sizemore, G. M.; Co, C.; Smith, J. E.	Ventral pallidal extracellular fluid levels of dopamine, serotonin, gamma amino butyric acid, and glutamate during cocaine self-administration in rats	2000	150	391-398	Psychopharmacology(Berl)
Skorzewska, A.; Bidzinski, A.; Hamed, A.; Lehner, M.; Turzynska, D.; Sobolewska, A.; Szyndler, J.; Maciejak, P.; Wislowska-Stanek, A.; Plaznik, A.	The effect of CRF and alpha-helical CRF((9-41)) on rat fear responses and amino acids release in the central nucleus of the amygdala	2009	57	148-156	Neuropharmacology
Skorzewska, A.; Bidzinski, A.; Hamed, A.; Lehner, M.; Turzynska, D.; Sobolewska, A.; Walkowiak, J.; Plaznik, A.	Changes in hippocampal amino acid concentrations after chronic administration of corticosterone	2007	59	763-772	Pharmacol Rep
Slais, K.; Vorisek, I.; Zoremba, N.; Homola, A.; Dmytrenko, L.; Sykova, E.	Brain metabolism and diffusion in the rat cerebral cortex during pilocarpine-induced status epilepticus	2008	209	145-154	Exp Neurol
Slaney, T. R.; Mabrouk, O. S.; Porter-Stransky, K. A.; Aragona, B. J.; Kennedy, R. T.	Chemical gradients within brain extracellular space measured using low flow push-pull perfusion sampling in vivo	2013	4	321-329	ACS Chem Neurosci
Slaney, T. R.; Nie, J.; Hershey, N. D.; Thwar, P. K.; Linderman, J.; Burns, M. A.; Kennedy, R. T.	Push-pull perfusion sampling with segmented flow for high temporal and spatial resolution in vivo chemical monitoring	2011	83	5207-5213	Anal Chem
Slezia, A.; Kekesi, A. K.; Szikra, T.; Papp, A. M.; Nagy, K.; Szente, M.; Magloczky, Z.; Freund, T. F.; Juhasz, G.	Uridine release during aminopyridine-induced epilepsy	2004	16	490-499	Neurobiol Dis
Smith, A.; Watson, C. J.; Frantz, K. J.; Eppler, B.; Kennedy, R. T.; Peris, J.	Differential increase in taurine levels by low-dose ethanol in the dorsal and ventral striatum revealed by microdialysis with on-line capillary electrophoresis	2004	28	1028-1038	Alcohol Clin Exp Res
Smith, J. A.; Mo, Q.; Guo, H.; Kunko, P.	Cocaine increases extraneuronal levels of aspartate and	1995	683	264-269	Brain Res

Glutamate					
**Authors**	**Title**	**Year**	**Vol.**	**Pages**	**Journal**
M.; Robinson, S. E.	glutamate in the nucleus accumbens				
Smith, S. E.; Lekieffre, D.; Sowinski, P.; Meldrum, B. S.	Cerebroprotective effect of BW619C89 after focal or global cerebral ischaemia in the rat	1993	4	1339-1342	Neuroreport
Smith, S. L.; Thompson, K. S. J.; Sargent, B. J.; Heal, D. J.	BTS 72664 - A novel CNS drug with potential anticonvulsant, neuroprotective, and antimigraine properties	2001	7	146-171	CNS Drug Review
Smolders, I.; Bogaert, L.; Ebinger, G.; Michotte, Y.	Muscarinic modulation of striatal dopamine, glutamate, and GABA release, as measured with in vivo microdialysis	1997	68	1942-1948	J Neurochem
Smolders, I.; Gousseau, C.; Marchand, S.; Couet, W.; Ebinger, G.; Michotte, Y.	Convulsant and subconvulsant doses of norfloxacin in the presence and absence of biphenylacetic acid alter extracellular hippocampal glutamate but not gamma-aminobutyric acid levels in conscious rats	2002	46	471-477	Antimicrob Agents Chemother
Smolders, I.; Khan, G. M.; Lindekens, H.; Prikken, S.; Marvin, C. A.; Manil, J.; Ebinger, G.; Michotte, Y.	Effectiveness of vigabatrin against focally evoked pilocarpine-induced seizures and concomitant changes in extracellular hippocampal and cerebellar glutamate, gamma-aminobutyric acid and dopamine levels, a microdialysis-electrocorticography study in freely moving rats	1997	283	1239-1248	J Pharmacol Exp Ther
Smolders, I.; Khan, G. M.; Manil, J.; Ebinger, G.; Michotte, Y.	NMDA receptor-mediated pilocarpine-induced seizures: characterization in freely moving rats by microdialysis	1997	121	1171-1179	Br J Pharmacol
Smolders, I.; Lindekens, H.; Clinckers, R.; Meurs, A.; Neill, M. J.; Lodge, D.; Ebinger, G.; Michotte, Y.	In vivo modulation of extracellular hippocampal glutamate and GABA levels and limbic seizures by group I and II metabotropic glutamate receptor ligands	2004	88	1068-1077	J Neurochem
Smolders, I.; Sarre, S.; Ebinger, G.; Michotte, Y.	Microbore liquid chromatography analysis of amino acid transmitters	1997	72	197-204	Methods Mol Biol
Smolders, I.; Sarre, S.; Michotte, Y.; Ebinger, G.	The analysis of excitatory, inhibitory and other amino acids in rat brain microdialysates using microbore liquid chromatography	1995	57	47-53	J Neurosci Methods
Smolders, I.; Sarre, S.; Vanhaesendonck, C.; Ebinger, G.; Michotte, Y.	Extracellular striatal dopamine and glutamate after decortication and kainate receptor stimulation, as measured by microdialysis	1996	66	2373-2380	J Neurochem
Smolders, I.; Van Belle, K.; Ebinger, G.; Michotte, Y.	Hippocampal and cerebellar extracellular amino acids during pilocarpine-induced seizures in freely moving rats	1997	319	21-29	Eur J Pharmacol
Solas, A. B.; Kutzsche, S.; Vinje, M.; Saugstad, O. D.	Cerebral hypoxemia-ischemia and reoxygenation with 21% or 100% oxygen in newborn piglets: effects on extracellular levels of excitatory amino acids and microcirculation	2001	2	340-345	Pediatr Crit Care Med
Solas, A. B.; Munkeby, B. H.; Saugstad,	Comparison of short- and long-duration oxygen treatment after	2004	56	125-131	Pediatr Res

Glutamate					
**Authors**	**Title**	**Year**	**Vol.**	**Pages**	**Journal**
O. D.	cerebral asphyxia in newborn piglets				
Solinas, M.; Ferre, S.; You, Z. B.; Karcz-Kubicha, M.; Popoli, P.; Goldberg, S. R.	Caffeine induces dopamine and glutamate release in the shell of the nucleus accumbens	2002	22	6321-6324	J Neurosci
Solis, J. M.; Herranz, A. S.; Herreras, O.; Lerma, J.; Martin Del Rio, R.	Low chloride-dependent release of taurine by a furosemide-sensitive process in the in vivo rat hippocampus	1988	24	885-891	Neuroscience
Solis, J. M.; Herranz, A. S.; Herreras, O.; Lerma, J.; Martin del Rio, R.	Does taurine act as an osmoregulatory substance in the rat brain?	1988	91	53-58	Neurosci Lett
Solis, J. M.; Herranz, A. S.; Herreras, O.; Munoz, M. D.; Martin del Rio, R.; Lerma, J.	Variation of potassium ion concentrations in the rat hippocampus specifically affects extracellular taurine levels	1986	66	263-268	Neurosci Lett
Song, P.; Mabrouk, O. S.; Hershey, N. D.; Kennedy, R. T.	In vivo neurochemical monitoring using benzoyl chloride derivatization and liquid chromatography-mass spectrometry	2012	84	412-419	Anal Chem
Soukupova, M.; Binaschi, A.; Falcicchia, C.; Palma, E.; Roncon, P.; Zucchini, S.; Simonato, M.	Increased extracellular levels of glutamate in the hippocampus of chronically epileptic rats	2015	301	246-253	Neuroscience
Srkalovic, G.; Selim, M.; Rea, M. A.; Glass, J. D.	Serotonergic inhibition of extracellular glutamate in the suprachiasmatic nuclear region assessed using in vivo brain microdialysis	1994	656	302-308	Brain Res
Stanley, E. M.; Fadel, J. R.	Aging-related alterations in orexin/hypocretin modulation of septo-hippocampal amino acid neurotransmission	2011	195	70-9	Neuroscience
Stanley, E. M.; Fadel, J. R.; Mott, D. D.	Interneuron loss reduces dendritic inhibition and GABA release in hippocampus of aged rats	2012	33	431.e1-431.e13	Neurobiol Aging
Starowicz, K.; Maione, S.; Cristino, L.; Palazzo, E.; Marabese, I.; Rossi, F.; de Novellis, V.; Di Marzo, V.	Tonic endovanilloid facilitation of glutamate release in brainstem descending antinociceptive pathways	2007	27	13739-13749	J Neurosci
Steciuk, M.; Kram, M.; Kramer, G. L.; Petty, F.	Immobilization-induced glutamate efflux in medial prefrontal cortex: blockade by (+)-Mk-801, a selective NMDA receptor antagonist	2000	3	195-199	Stress
Stein-Behrens, B. A.; Elliott, E. M.; Miller, C. A.; Schilling, J. W.; Newcombe, R.; Sapolsky, R. M.	Glucocorticoids exacerbate kainic acid-induced extracellular accumulation of excitatory amino acids in the rat hippocampus	1992	58	1730-1735	J Neurochem
Stein-Behrens, B. A.; Lin, W. J.; Sapolsky, R. M.	Physiological elevations of glucocorticoids potentiate glutamate accumulation in the hippocampus	1994	63	596-602	J Neurochem

Glutamate					
**Authors**	**Title**	**Year**	**Vol.**	**Pages**	**Journal**
Stephans, S. E.; Yamamoto, B. K.	Effect of repeated methamphetamine administrations on dopamine and glutamate efflux in rat prefrontal cortex	1995	700	99-106	Brain Research
Stephans, S. E.; Yamamoto, B. K.	Methamphetamine-induced neurotoxicity: roles for glutamate and dopamine efflux	1994	17	203-209	Synapse
Stephens, M. L.; Pomerleau, F.; Huettl, P.; Gerhardt, G. A.; Zhang, Z.	Real-time glutamate measurements in the putamen of awake rhesus monkeys using an enzyme-based human microelectrode array prototype	2010	185	264-272	J Neurosci Methods
Stier, C.; Skorka, G.; Sohr, R.; Ott, T.	Time course and role of extracellular Ca2+ in veratridine-induced glutamate release	1996	7	401-404	Neuroreport
Stiller, C. O.; Linderoth, B.; Connor, W. T.; Franck, J.; Falkenberg, T.; Ungerstedt, U.; Brodin, E.	Repeated spinal cord stimulation decreases the extracellular level of gamma-aminobutyric acid in the periaqueductal gray matter of freely moving rats	1995	699	231-241	Brain Res
Stoffel, M.; Eriskat, J.; Plesnila, M.; Aggarwal, N.; Baethmann, A.	The penumbra zone of a traumatic cortical lesion: a microdialysis study of excitatory amino acid release	1997	70	91-93	Acta Neurochir Suppl
Stoffel, M.; Plesnila, N.; Eriskat, J.; Furst, M.; Baethmann, A.	Release of excitatory amino acids in the penumbra of a focal cortical necrosis	2002	19	467-477	J Neurotrauma
Storozheva, Z. I.; Proshin, A. T.	Selective involvement of the neurotransmitter systems of the cerebellum in the mechanisms forming different types of defensive behavior	2011	41	964-972	Neurosci Behav Physiol
Storozheva, Z. I.; Proshin, A. T.	[Selective involvement of cerebellar neurotransmitter systems in mechanisms of various types of defensive behavior]	2010	60	474-485	Zh Vyssh Nerv Deiat Im I P Pavlova
Stover, J. F.; Sakowitz, O. W.; Beyer, T. F.; Dohse, N. K.; Kroppenstedt, S. N.; Thomale, U. W.; Schaser, K. D.; Unterberg, A. W.	Effects of LY379268, a selective group II metabotropic glutamate receptor agonist on EEG activity, cortical perfusion, tissue damage, and cortical glutamate, glucose, and lactate levels in brain-injured rats	2003	20	315-326	J Neurotrauma
Stover, J. F.; Sakowitz, O. W.; Kroppenstedt, S. N.; Thomale, U. W.; Kempski, O. S.; Flugge, G.; Unterberg, A. W.	Differential effects of prolonged isoflurane anesthesia on plasma, extracellular, and CSF glutamate, neuronal activity, 125I-Mk801 NMDA receptor binding, and brain edema in traumatic brain-injured rats	2004	146	819-830	Acta Neurochir (Wien)
Suarez, L. M.; Munoz, M. D.; Gonzalez, J. C.; Bustamante, J.; Del Rio, R. M.; Solis, J. M.	The taurine transporter substrate guanidinoethyl sulfonate mimics the action of taurine on long-term synaptic potentiation	2016	48	2647-2656	Amino Acids
Succu, S.; Mascia, M. S.; Sanna, F.;	The cannabinoid CB1 receptor antagonist SR 141716A induces	2006	169	274-281	Behav Brain Res

Glutamate					
**Authors**	**Title**	**Year**	**Vol.**	**Pages**	**Journal**
Melis, T.; Argiolas, A.; Melis, M. R.	penile erection by increasing extra-cellular glutamic acid in the paraventricular nucleus of male rats				
Sugahara, M.; Asai, S.; Zhao, H.; Nagata, T.; Kunimatsu, T.; Ishii, Y.; Kohno, T.; Ishikawa, K.	Extracellular glutamate changes in rat striatum during ischemia determined by a novel dialysis electrode and conventional microdialysis	2001	39	65-73	Neurochem Int
Sullivan, M. E.; Hall, S. R.; Milne, B.; Jhamandas, K.	Suppression of acute and chronic opioid withdrawal by a selective soluble guanylyl cyclase inhibitor	2000	859	45-56	Brain Res
Sumbria, R. K.; Klein, J.; Bickel, U.	Acute depression of energy metabolism after microdialysis probe implantation is distinct from ischemia-induced changes in mouse brain	2011	36	109-116	Neurochem Res
Sun, J. Y.; Yang, J. Y.; Wang, F.; Wang, J. Y.; Song, W.; Su, G. Y.; Dong, Y. X.; Wu, C. F.	Lesions of nucleus accumbens affect morphine-induced release of ascorbic acid and GABA but not of glutamate in rats	2011	16	540-550	Addict Biol
Sun, Z.; Jia, J.; Gong, X.; Jia, Y.; Deng, J.; Wang, X.	Inhibition of glutamate and acetylcholine release in behavioral improvement induced by electroacupuncture in parkinsonian rats	2012	520	32-37	Neurosci Lett
Sustkova-Fiserova, M.; Vavrova, J.; Krsiak, M.	Brain levels of GABA, glutamate and aspartate in sociable, aggressive and timid mice: an in vivo microdialysis study	2009	30	79-84	Neuro Endocrinol Lett
Suto, N.; Ecke, L. E.; You, Z. B.; Wise, R. A.	Extracellular fluctuations of dopamine and glutamate in the nucleus accumbens core and shell associated with lever-pressing during cocaine self-administration, extinction, and yoked cocaine administration	2010	211	267-275	Psychopharmacology(Berl)
Suto, N.; Elmer, G. I.; Wang, B.; You, Z. B.; Wise, R. A.	Bidirectional modulation of cocaine expectancy by phasic glutamate fluctuations in the nucleus accumbens	2013	33	9050-9055	J Neurosci
Suto, T.; Severino, A. L.; Eisenach, J. C.; Hayashida, K.	Gabapentin increases extracellular glutamatergic level in the locus coeruleus via astroglial glutamate transporter-dependent mechanisms	2014	81	95-100	Neuropharmacology
Suzuki, K.; Matsuo, N.; Moriguchi, T.; Takeyama, N.; Kitazawa, Y.; Tanaka, T.	Changes in brain ECF amino acids in rats with experimentally induced hyperammonemia	1992	7	63-75	Metab Brain Dis
Suzuki, S.; Saitoh, A.; Ohashi, M.; Oka, J. I.; Yamada, M.	The infralimbic and prelimbic medial prefrontal cortices have differential functions in the expression of anxiety-like behaviors in mice	2016	304	120-124	Behav Brain Res
Sved, A. F.; Curtis, J. T.	Amino acid neurotransmitters in nucleus tractus solitarius: an in vivo microdialysis study	1993	61	2089-2098	J Neurochem

Glutamate					
**Authors**	**Title**	**Year**	**Vol.**	**Pages**	**Journal**
Swanson, C. J.; Baker, D. A.; Carson, D.; Worley, P. F.; Kalivas, P. W.	Repeated cocaine administration attenuates group I metabotropic glutamate receptor-mediated glutamate release and behavioral activation: a potential role for Homer	2001	21	9043-9052	J Neurosci
Swanson, R. A.; Chen, J.; Graham, S. H.	Glucose can fuel glutamate uptake in ischemic brain	1994	14	1-6	J Cereb Blood Flow Metab
Szeg, E. M.; Kekesi, K. A.; Szabo, Z.; Janaky, T.; Juhasz, G. D.	Estrogen regulates cytoskeletal flexibility, cellular metabolism and synaptic proteins: A proteomic study	2010	35	807-819	Psychoeneuroendocrinology
Szerb, J. C.	Glutamate release and spreading depression in the fascia dentata in response to microdialysis with high K+: role of glia	1991	542	259-265	Brain Res
Szumlinski, K. K.; Abernathy, K. E.; Oleson, E. B.; Klugmann, M.; Lominac, K. D.; He, D. Y.; Ron, D.; During, M.; Kalivas, P. W.	Homer isoforms differentially regulate cocaine-induced neuroplasticity	2006	31	768-777	Neuropsychopharmacology
Szumlinski, K. K.; Diab, M. E.; Friedman, R.; Henze, L. M.; Lominac, K. D.; Bowers, M. S.	Accumbens neurochemical adaptations produced by binge-like alcohol consumption	2007	190	415-431	Psychopharmacology (Berl)
Szumlinski, K. K.; Frys, K. A.; Kalivas, P. W.	Dissociable roles for the dorsal and median raphe in the facilitatory effect of 5-HT1A receptor stimulation upon cocaine-induced locomotion and sensitization	2004	29	1675-1687	Neuropsychopharmacology
Szumlinski, K. K.; Liu, A.; Penzner, J. H.; Lominac, K. D.	Protracted 'pro-addictive' phenotype produced in mice by pre-adolescent phenylpropanolamine	2007	32	1760-1773	Neuropsychopharmacology
Szumlinski, K. K.; Lominac, K. D.; Campbell, R. R.; Cohen, M.; Fultz, E. K.; Brown, C. N.; Miller, B. W.; Quadir, S. G.; Martin, D.; Thompson, A. B.; von Jonquieres, G.; Klugmann, M.; Phillips, T. J.; Kippin, T. E.	Methamphetamine Addiction Vulnerability: The Glutamate, the Bad, and the Ugly	2016	81	959-970	Biol Psychiatry
Szumlinski, K. K.; Lominac, K. D.; Kleschen, M. J.; Oleson, E. B.; Dehoff, M. H.; Schwartz, M. K.; Seeberg, P. H.; Worley, P. F.; Kalivas, P. W.	Behavioral and neurochemical phenotyping of Homer1 mutant mice: Possible relevance to schizophrenia	2005	4	273-288	Genes Brain Behav
Szyndler, J.; Maciejak, P.; Turzynska, D.; Sobolewska, A.; Lehner, M.; Taracha, E.; Walkowiak, J.; Skorzewska, A.;	Changes in the concentration of amino acids in the hippocampus of pentylenetetrazole-kindled rats	2008	439	245-249	Neurosci Lett

Glutamate					
**Authors**	**Title**	**Year**	**Vol.**	**Pages**	**Journal**
Wislowska-Stanek, A.; Hamed, A.; Bidzinski, A.; Plaznik, A.					
Taguchi, J.; Graf, R.; Rosner, G.; Heiss, W. D.	Prolonged transient ischemia results in impaired CBF recovery and secondary glutamate accumulation in cats	1996	16	271-279	J Cereb Blood Flow Metab
Takagi, K.; Ginsberg, M. D.; Globus, M. Y.; Dietrich, W. D.; Martinez, E.; Kraydieh, S.; Busto, R.	Changes in amino acid neurotransmitters and cerebral blood flow in the ischemic penumbral region following middle cerebral artery occlusion in the rat: correlation with histopathology	1993	13	575-585	J Cereb Blood Flow Metab
Takagi, K.; Ginsberg, M. D.; Globus, M. Y.; Martinez, E.; Busto, R.	Effect of hyperthermia on glutamate release in ischemic penumbra after middle cerebral artery occlusion in rats	1994	267	H1770-H1776	Am J Physiol
Takahashi, A.; Lee, R. X.; Iwasato, T.; Itohara, S.; Arima, H.; Bettler, B.; Miczek, K. A.; Koide, T.	Glutamate input in the dorsal raphe nucleus as a determinant of escalated aggression in male mice	2015	35	6452-6463	J Neurosci
Takahashi, K.; Murasawa, H.; Yamaguchi, K.; Nakatani, A.; Yoshida, M.; Iwai, T.; Inagaki, M.; Yamada, M.; Saitoh, A.	Riluzole rapidly attenuates hyperemotional responses in olfactory bulbectomized rats, an animal model of depression	2011	216	46-52	Behav Brain Res
Takaki, M.; Ueda, Y.; Doi, T.; Nagatomo, K.; Murashima, Y. L.; Kannan, H.	Molecular regulation of antioxidant ability in the hippocampus of EL mice	2008	1228	1-5	Brain Res
Takatsuru, Y.; Eto, K.; Kaneko, R.; Masuda, H.; Shimokawa, N.; Koibuchi, N.; Nabekura, J.	Critical role of the astrocyte for functional remodeling in contralateral hemisphere of somatosensory cortex after stroke	2013	33	4683-4692	J Neurosci
Takazawa, A.; Murashima, Y. L.; Minatogawa, Y.; Kojima, T.; Tanaka, K.; Yamauchi, T.	In vivo microdialysis monitoring for extracellular glutamate and GABA in the ventral hippocampus of the awake rat during kainate-induced seizures	1995	49	S275-S277	Psychiatry Clin Neurosci
Takeda, A.; Ando, M.; Kanno, S.; Oku, N.	Unique response of zinc in the hippocampus to behavioral stress and attenuation of subsequent mossy fiber long-term potentiation	2009	30	712-717	Neuro Toxicology
Takeda, A.; Hirate, M.; Tamano, H.; Nisibaba, D.; Oku, N.	Susceptibility to kainate-induced seizures under dietary zinc deficiency	2003	85	1575-1580	J Neurochem
Takeda, A.; Hirate, M.; Tamano, H.; Oku, N.	Release of glutamate and GABA in the hippocampus under zinc deficiency	2003	72	537-542	J Neurosci Res
Takeda, A.; Hirate, M.; Tamano, H.; Oku, N.	Zinc movement in the brain under kainate-induced seizures	2003	54	123-129	Epilepsy Res
Takeda, A.; Itoh, H.; Imano, S.; Oku, N.	Impairment of GABAergic neurotransmitter system in the amygdala of young rats after 4-week zinc deprivation	2006	49	746-750	Neurochem Int

Glutamate					
**Authors**	**Title**	**Year**	**Vol.**	**Pages**	**Journal**
Takeda, A.; Itoh, H.; Tamano, H.; Yuzurihara, M.; Oku, N.	Suppressive effect of Yokukansan on excessive release of glutamate and aspartate in the hippocampus of zinc-deficient rats	2008	11	41-46	Nutr Neurosci
Takeda, A.; Minami, A.; Sakurada, N.; Nakajima, S.; Oku, N.	Response of hippocampal mossy fiber zinc to excessive glutamate release	2007	50	322-327	Neurochem Int
Takeda, A.; Minami, A.; Seki, Y.; Nakajima, S.; Oku, N.	Release of amino acids by zinc in the hippocampus	2004	63	253-257	Brain Res Bull
Takeda, A.; Minami, A.; Seki, Y.; Oku, N.	Differential effects of zinc on glutamatergic and GABAergic neurotransmitter systems in the hippocampus	2004	75	225-229	J Neurosci Res
Takeda, A.; Minami, A.; Seki, Y.; Oku, N.	Inhibitory function of zinc against excitation of hippocampal glutamatergic neurons	2003	57	169-174	Epilepsy Res
Takeda, A.; Minami, A.; Yamaide, R.; Oku, N.	Involvement of amygdalar extracellular zinc in rat behavior for passive avoidance	2004	358	119-122	Neurosci Lett
Takeda, A.; Sakurada, N.; Kanno, S.; Ando, M.; Oku, N.	Vulnerability to seizures induced by potassium dyshomeostasis in the hippocampus in aged rats	2008	54	37-42	J Health Sci
Takeda, A.; Sakurada, N.; Kanno, S.; Minami, A.; Oku, N.	Response of extracellular zinc in the ventral hippocampus against novelty stress	2006	99	670-676	J Neurochem
Takeda, A.; Sotogaku, N.; Oku, N.	Influence of manganese on the release of neurotransmitters in rat striatum	2003	965	279-282	Brain Res
Takeda, A.; Sotogaku, N.; Oku, N.	Manganese influences the levels of neurotransmitters in synapses in rat brain	2002	114	669-674	Neuroscience
Takeda, A.; Tamano, H.; Itoh, H.; Oku, N.	Attenuation of abnormal glutamate release in zinc deficiency by zinc and Yokukansan	2008	53	230-235	Neurochem Int
Takeda, A.; Tamano, H.; Kan, F.; Hanajima, T.; Yamada, K.; Oku, N.	Enhancement of social isolation-induced aggressive behavior of young mice by zinc deficiency	2008	82	909-914	Life Sci
Takeda, A.; Tamano, H.; Oku, N.	Involvement of unusual glutamate release in kainate-induced seizures in zinc-deficient adult rats	2005	66	137-143	Epilepsy Res
Takita, M.; Kaneko, H.; Suzuki, S. S.; Akamatsu, M.	Lasting effect of NO on glutamate release in rat striatum revealed by continuous brain dialysis	1997	8	567-570	Neuroreport
Takita, M.; Kawashima, T.; Kaneko, H.; Suzuki, S. S.; Yokoi, H.	Sensitization of glutamate release and N-methyl-D-aspartate receptor response by transient dopamine pretreatment in prefrontal cortex of rats	2002	317	97-100	Neurosci Lett
Tamano, H.; Takeda, A.	Suppressive effect of Saiko-ka-ryukotsu-borei-to, a herbal medicine, on excessive release of glutamate in the hippocampus	2004	64	273-277	Brain Res Bull

Glutamate					
**Authors**	**Title**	**Year**	**Vol.**	**Pages**	**Journal**
Tan, W. K.; Williams, C. E.; During, M. J.; Mallard, C. E.; Gunning, M. I.; Gunn, A. J.; Gluckman, P. D.	Accumulation of cytotoxins during the development of seizures and edema after hypoxic-ischemic injury in late gestation fetal sheep	1996	39	791-797	Pediatr Res
Tanahashi, S.; Ueda, Y.; Nakajima, A.; Yamamura, S.; Nagase, H.; Okada, M.	Novel delta1-receptor agonist KNT-127 increases the release of dopamine and L-glutamate in the striatum, nucleus accumbens and median pre-frontal cortex	2012	62	2057-2067	Neuropharmacology
Tanahashi, S.; Yamamura, S.; Nakagawa, M.; Motomura, E.; Okada, M.	Clozapine, but not haloperidol, enhances glial D-serine and L-glutamate release in rat frontal cortex and primary cultured astrocytes	2012	165	1543-1555	Br J Pharmacol
Tanaka, H.; Katayama, Y.; Kawamata, T.; Tsubokawa, T.	Excitatory amino acid release from contused brain tissue into surrounding brain areas	1994	60	524-527	Acta Neurochir Suppl (Wien)
Tanaka, K.; Graham, S. H.; Simon, R. P.	The role of excitatory neurotransmitters in seizure-induced neuronal injury in rats	1996	737	59-63	Brain Res
Tanaka, S.; Kiuchi, Y.; Tsuchida, A.; Yamamoto, T.; Oguchi, K.; Yoshida, T.; Kuroiwa, Y.	Possible involvement of an increase in brain extracellular concentration of excitatory amino acids in convulsions in phenobarbital-withdrawn rats	1996	17	91-102	Res Commun Alcohol Substances
Tanaka, Y.; Han, H.; Hagishita, T.; Fukui, F.; Liu, G.; Ando, S.	alpha-Sialylcholesterol enhances the depolarization-induced release of acetylcholine and glutamate in rat hippocampus: in vivo microdialysis study	2004	357	9-12	Neurosci Lett
Tang, Y. B.; Sun, F.; Teng, L.; Li, W. B.; An, S. M.; Zhang, C.; Yang, X. J.; Lv, H. Y.; Ding, X. P.; Zhu, L.; Chen, H. Z.	Simultaneous determination of the repertoire of classical neurotransmitters released from embryonal carcinoma stem cells using online microdialysis coupled with hydrophilic interaction chromatography-tandem mass spectrometry	2014	849	70-79	Anal Chim Acta
Tanganelli, S.; Sandager Nielsen, K.; Ferraro, L.; Antonelli, T.; Kehr, J.; Franco, R.; Ferre, S.; Agnati, L. F.; Fuxe, K.; Scheel-Kruger, J.	Striatal plasticity at the network level. Focus on adenosine A2A and D2 interactions in models of Parkinson's Disease	2004	10	273-280	Parkinsonism Relat Disord
Taninishi, H.; Takeda, Y.; Kobayashi, M.; Arai, M.; Sasaki, T.; Morita, K.	Nitrous oxide administration increased neuronal damage during cerebral ischemia without affecting extracellular glutamate concentration in gerbils	2007	27	BP57-BP03U	J Cereb Blood Flow Metab
Taninishi, H.; Takeda, Y.; Kobayashi, M.; Sasaki, T.; Arai, M.; Morita, K.	Effect of nitrous oxide on neuronal damage and extracellular glutamate concentration as a function of mild, moderate, or severe ischemia in halothane-anesthetized gerbils	2008	108	1063-1070	Anesthesiology

Glutamate					
**Authors**	**Title**	**Year**	**Vol.**	**Pages**	**Journal**
Taninishi, H.; Takeda, Y.; Morita, K.	Effect of continuous intravenous Ketamine on dynamic change in extracellular glutamate concentration during cerebral ischemia by use of microdialysis in gerbils	2010	22(4)	424-425	J Neurosurg Anesthesiol
Taylor, D. L.; Davies, S. E.; Obrenovitch, T. P.; Doheny, M. H.; Patsalos, P. N.; Clark, J. B.; Symon, L.	Investigation into the role of N-acetylaspartate in cerebral osmoregulation	1995	65	275-281	J Neurochem
Tebano, M. T.; Pintor, A.; Frank, C.; Domenici, M. R.; Martire, A.; Pepponi, R.; Potenza, R. L.; Grieco, R.; Popoli, P.	Adenosine A2A receptor blockade differentially influences excitotoxic mechanisms at pre- and postsynaptic sites in the rat striatum	2004	77	100-107	J Neurosci Res
Tedesco, A.; Ally, A.	Angiotensin II type-2 (AT2) receptor antagonism alters cardiovascular responses to static exercise and simultaneously changes glutamate/GABA levels within the ventrolateral medulla	2009	64	372-379	Neurosci Res
Teismann, P.; Ferger, B.	Effects of ensaculin on dopamine metabolite levels and K(+)-induced glutamate release	2000	398	247-250	Eur J Pharmacol
Tejeda, H. A.; Counotte, D. S.; Oh, E.; Ramamoorthy, S.; Schultz-Kuszak, K. N.; Backman, C. M.; Chefer, V.; Donnell, P.; Shippenberg, T. S.	Prefrontal cortical kappa-opioid receptor modulation of local neurotransmission and conditioned place aversion	2013	38	1770-1779	Neuropsychopharmacology
Terzioglu, B.; Aypak, C.; Onat, F. Y.; Kucukibrahimoglu, E.; Ozkaynakci, A. E.; Goren, M. Z.	The effects of ethosuximide on amino acids in genetic absence epilepsy rat model	2006	100	227-233	J Pharmacol Sci
Terzioglu, B.; Karaalp, A.; Zafer Goren, M.	A linear relationship between lamotrigine and GABA in cerebrospinal fluid	2011	15	1-6	Marmara Pharm J
Thomas, L. S.; Jane, D. E.; Gasparini, F.; Croucher, M. J.	Glutamate release inhibiting properties of the novel mGlu(5) receptor antagonist 2-methyl-6-(phenylethynyl)-pyridine (MPEP): complementary in vitro and in vivo evidence	2001	41	523-527	Neuropharmacology
Timaru-Kast, R.; Meissner, A.; Heimann, A.; Hoelper, B.; Kempski, O.; Alessandri, B.	Acute subdural hematoma in pigs: Role of volume on multiparametric neuromonitoring and histology	2008	25	1107-1119	J Neurotrauma
Timmer, K. M.; Steketee, J. D.	Group I metabotropic glutamate receptors in the medial prefrontal cortex: role in mesocorticolimbic glutamate release in cocaine sensitization	2013	67	887-896	Synapse
Timmerman, W.; Cisci, G.; Nap, A.; de	Effects of handling on extracellular levels of glutamate and other	1999	833	150-160	Brain Res

Glutamate					
**Authors**	**Title**	**Year**	**Vol.**	**Pages**	**Journal**
Vries, J. B.; Westerink, B. H.	amino acids in various areas of the brain measured by microdialysis				
Tin Tin Win, Shwe; Mitsushima, D.; Yamamoto, S.; Fukushima, A.; Funabashi, T.; Kobayashi, T.; Fujimaki, H.	Changes in neurotransmitter levels and proinflammatory cytokine mRNA expressions in the mice olfactory bulb following nanoparticle exposure	2008	226	192-198	Toxicol Appl Pharmacol
Todd, K. G.; Butterworth, R. F.	In vivo microdialysis in an animal model of neurological disease: thiamine deficiency (Wernicke) encephalopathy	2001	23	55-61	Methods
Todd, K. G.; Butterworth, R. F.	Evaluation of the role of NMDA-mediated excitotoxicity in the selective neuronal loss in experimental Wernicke encephalopathy	1998	149	130-138	Exp Neurol
Togashi, H.; Nakamura, K.; Matsumoto, M.; Ueno, K.; Ohashi, S.; Saito, H.; Yoshioka, M.	Aniracetam enhances glutamatergic transmission in the prefrontal cortex of stroke-prone spontaneously hypertensive rats	2002	320	109-112	Neurosci Lett
Torp, R.; Arvin, B.; Le Peillet, E.; Chapman, A. G.; Ottersen, O. P.; Meldrum, B. S.	Effect of ischaemia and reperfusion on the extra- and intracellular distribution of glutamate, glutamine, aspartate and GABA in the rat hippocampus, with a note on the effect of the sodium channel blocker BW1003C87	1993	96	365-376	Exp Brain Res
Toshinai, K.; Saito, T.; Yamaguchi, H.; Sasaki, K.; Tsuchimochi, W.; Minamino, N.; Ueta, Y.; Nakazato, M.	Neuroendocrine regulatory peptide-1 and -2 (NERPs) inhibit the excitability of magnocellular neurosecretory cells in the hypothalamus	2014	1563	52-60	Brain Res
Tossman, U.; Delin, A.; Eriksson, L. S.; Ungerstedt, U.	Brain cortical amino acids measured by intracerebral dialysis in portacaval shunted rats	1987	12	265-269	Neurochem Res
Tossman, U.; Eriksson, S.; Delin, A.; Hagenfeldt, L.; Law, D.; Ungerstedt, U.	Brain amino acids measured by intracerebral dialysis in portacaval shunted rats	1983	41	1046-1051	J Neurochem
Tossman, U.; Jonsson, G.; Ungerstedt, U.	Regional distribution and extracellular levels of amino acids in rat central nervous system	1986	127	533-545	Acta Physiol Scand
Tossman, U.; Segovia, J.; Ungerstedt, U.	Extracellular levels of amino acids in striatum and globus pallidus of 6-hydroxydopamine-lesioned rats measured with microdialysis	1986	127	547-551	Acta Physiol Scand
Toth, E.	Effect of nicotine on the level of extracellular amino acids in the hippocampus of rat	1996	21	903-907	Neurochem Res
Toth, E.; Harsing Jr, L. G.; Sershen, H.; Ramacci, M. T.; Lajtha, A.	Effect of acetyl-L-carnitine on extracellular amino acid levels in vivo in rat brain regions	1993	18	573-578	Neurochem Res
Toth, E.; Sershen, H.; Hashim, A.; Vizi, E. S.; Lajtha, A.	Effect of nicotine on extracellular levels of neurotransmitters assessed by microdialysis in various brain regions: role of	1992	17	265-271	Neurochem Res

Glutamate					
Authors	Title	Year	Vol.	Pages	Journal
	glutamic acid				
Toth, E.; Vizi, E. S.; Lajtha, A.	Effect of nicotine on levels of extracellular amino acids in regions of the rat brain in vivo	1993	32	827-832	Neuropharmacology
Touchon, J. C.; Holmer, H. K.; Moore, C.; McKee, B. L.; Frederickson, J.; Meshul, C. K.	Apomorphine-induced alterations in striatal and substantia nigra pars reticulata glutamate following unilateral loss of striatal dopamine	2005	193	131-140	Exp Neurol
Touchon, J. C.; Moore, C.; Frederickson, J.; Meshul, C. K.	Lesion of Subthalamic or Motor Thalamic Nucleus in 6-Hydroxydopamine-Treated Rats: Effects on Striatal Glutamate and Apomorphine-Induced Contralateral Rotations	2004	51	287-298	Synapse
Touret, M.; Parrot, S.; Denoroy, L.; Belin, M. F.; Didier-Bazes, M.	Glutamatergic alterations in the cortex of genetic absence epilepsy rats	2007	8	69	BMC Neurosci
Tovar-y-Romo, L. B.; Tapia, R.	Cerebral neurons of transgenic ALS mice are vulnerable to glutamate release stimulation but not to increased extracellular glutamate due to transport blockade	2006	199	281-290	Exp Neurol
Toya, S.; Takatsuru, Y.; Kokubo, M.; Amano, I.; Shimokawa, N.; Koibuchi, N.	Early-life-stress affects the homeostasis of glutamatergic synapses	2014	40	3627-3634	Eur J Neurosci
Toyota, S.; Graf, R.; Dohmen, C.; Valentino, M.; Grond, M.; Wienhard, K.; Heiss, W. D.	Elevation of extracellular glutamate in the final, ischemic stage of progressive epidural mass lesion in cats	2001	18	1349-1357	J Neurotrauma
Toyota, S.; Graf, R.; Valentino, M.; Yoshimine, T.; Heiss, W. D.	Malignant infarction in cats after prolonged middle cerebral artery occlusion: glutamate elevation related to decrease of cerebral perfusion pressure	2002	33	1383-1391	Stroke
Toyota, S.; Graf, R.; Valentino, M.; Yoshimine, T.; Heiss, W. D.	Prediction of malignant infarction: perifocal neurochemical monitoring following prolonged MCA occlusion in cats	2003	86	153-157	Acta Neurochir Suppl
Trabace, L.; Cassano, T.; Cagiano, R.; Tattoli, M.; Pietra, C.; Steardo, L.; Kendrick, K. M.; Cuomo, V.	Effects of ENA713 and CHF2819, two anti-Alzheimer's disease drugs, on rat amino acid levels	2001	910	182-186	Brain Res
Trabace, L.; Kendrick, K. M.	Nitric oxide can differentially modulate striatal neurotransmitter concentrations via soluble guanylate cyclase and peroxynitrite formation	2000	75	1664-1674	J Neurochem
Traficante, A.; Riozzi, B.; Cannella, M.; Rampello, L.; Squitieri, F.; Battaglia, G.	Reduced activity of cortico-striatal fibres in the R6/2 mouse model of Huntington's disease	2007	18	1997-2000	Neuroreport
Trantham-Davidson, H.; LaLumiere, R. T.;	Ceftriaxone normalizes nucleus accumbens synaptic	2012	32	12406-	J Neurosci

Glutamate					
**Authors**	**Title**	**Year**	**Vol.**	**Pages**	**Journal**
Reissner, K. J.; Kalivas, P. W.; Knackstedt, L. A.	transmission, glutamate transport, and export following cocaine self-administration and extinction training			12410	
Tsai, C. C.; Lin, M. T.; Wang, J. J.; Liao, J. F.; Huang, W. T.	The antipyretic effects of baicalin in lipopolysaccharide-evoked fever in rabbits	2006	51	709-717	Neuropharmacology
Tsai, G.; Stauch, B. L.; Vornov j, J.; Deshpande, J. K.; Coyle, J. T.	Selective release of N-acetylaspartylglutamate from rat optic nerve terminals in vivo	1990	518	313-316	Brain Res
Tsai, T. H.; Matthews, K.; Dalley, J. W.	Determination of glutamate in rat brain microdialysates by microbore liquid chromatography with electrochemical detection	1997	20	3039-3047	J Liq Chromatogr Relat Technol
Tseng, E. E.; Brock, M. V.; Kwon, C. C.; Annanata, M.; Lange, M. S.; Troncoso, J. C.; Johnston, M. V.; Baumgartner, W. A.	Increased intracerebral excitatory amino acids and nitric oxide after hypothermic circulatory arrest	1999	67	371-376	Ann Thorac Surg
Tseng, E. E.; Brock, M. V.; Lange, M. S.; Troncoso, J. C.; Blue, M. E.; Lowenstein, C. J.; Johnston, M. V.; Baumgartner, W. A.	Monosialoganglioside GM1 inhibits neurotoxicity after hypothermic circulatory arrest	1998	124	298-306	Surgery
Tseng, E. E.; Brock, M. V.; Lange, M. S.; Troncoso, J. C.; Blue, M. E.; Lowenstein, C. J.; Johnston, M. V.; Baumgartner, W. A.	Glutamate excitotoxicity mediates neuronal apoptosis after hypothermic circulatory arrest	2010	89	440-445	Ann Thorac Surg
Tsuchida, E.; Harms, J. F.; Woodward, J. J.; Bullock, R.	A use-dependent sodium channel antagonist, 619C89, in reduction of ischemic brain damage and glutamate release after acute subdural hematoma in the rat	1996	85	104-111	J Neurosurg
Tsukada, H.; Miyasato, K.; Nishiyama, S.; Fukumoto, D.; Kakiuchi, T.; Domino, E. F.	Nicotine normalizes increased prefrontal cortical dopamine D1 receptor binding and decreased working memory performance produced by repeated pretreatment with MK-801: a PET study in conscious monkeys	2005	30	2144-2153	Neuropsychopharmacology
Tsukada, Y.; Kanamatsu, T.; Takahara, H.	Neurotransmitter release from the medial hyperstriatum ventrale of the chick forebrain accompanying filial imprinting behavior, measured by in vivo microdialysis	1999	24	315-320	Neurochem Res
Tsuru, N.; Ueda, Y.	A seizure triggering mechanism of spontaneous epileptiform potential in the hippocampus	1997	23	61-78	J Brain Sci
Tucci, P.; Mhillaj, E.; Morgese, M. G.; Colaianna, M.; Zotti, M.; Schiavone, S.;	Memantine prevents memory consolidation failure induced by soluble beta amyloid in rats	2014	8	/	Front Behav Neurosci

Glutamate					
**Authors**	**Title**	**Year**	**Vol.**	**Pages**	**Journal**
Cicerale, M.; Trezza, V.; Campolongo, P.; Cuomo, V.; Trabace, L.					
Tucci, S.; Contreras, Q.; Paez, X.; Gonzalez, L.; Rada, P.; Hernandez, L.	Medial prefrontal transection enhances social interaction. II: neurochemical studies	2000	887	259-265	Brain Res
Tuma, P.; Sustkova-Fiserova, M.; Opekar, F.; Pavlicek, V.; Malkova, K.	Large-volume sample stacking for in vivo monitoring of trace levels of gamma-aminobutyric acid, glycine and glutamate in microdialysates of periaqueductal gray matter by capillary electrophoresis with contactless conductivity detection	2013	1303	94-99	J Chromatogr A
Uchiyama-Tsuyuki, Y.; Araki, H.; Yae, T.; Otomo, S.	Changes in the extracellular concentrations of amino acids in the rat striatum during transient focal cerebral ischemia	1994	62	1074-1078	J Neurochem
Ueda, Y.; Doi, T.; Nagatomo, K.; Tokumaru, J.; Takaki, M.; Willmore, L. J.	Effect of levetiracetam on molecular regulation of hippocampal glutamate and GABA transporters in rats with chronic seizures induced by amygdalar FeCl3 injection	2007	1151	55-61	Brain Res
Ueda, Y.; Doi, T.; Tokumaru, J.; Mitsuyama, Y.; James Willmore, L.	Kindling phenomena induced by the repeated short-term high potassium stimuli in the ventral hippocampus of rats: On-line monitoring of extracellular glutamate overflow	2000	135	199-203	Exp Brain Res
Ueda, Y.; Doi, T.; Tokumaru, J.; Yokoyama, H.; Nakajima, A.; Mitsuyama, Y.; Ohya-Nishiguchi, H.; Kamada, H.; James Willmore, L.	Collapse of extracellular glutamate regulation during epileptogenesis: Down-regulation and functional failure of glutamate transporter function in rats with chronic seizures induced by kainic acid	2001	76	892-900	J Neurochem
Ueda, Y.; Mitsuyama, Y.	Nitroprusside inhibits kindling development and potentiates excitatory amino acid exocytosis in the kindling rat	1997	23	49-60	Journal of Brain Science
Ueda, Y.; Obrenovitch, T. P.; Lok, S. Y.; Sarna, G. S.; Symon, L.	Efflux of glutamate produced by short ischemia of varied severity in rat striatum	1992	23	253-259	Stroke
Ueda, Y.; Obrenovitch, T. P.; Lok, S. Y.; Sarna, G. S.; Symon, L.; DeLorenzo, R. J.	Changes in extracellular glutamate concentration produced in the rat striatum by repeated ischemia	1992	23	1125-1131	Stroke
Ueda, Y.; Tsuru, N.	Simultaneous monitoring of the seizure-related changes in extracellular glutamate and gamma-aminobutyric acid concentration in bilateral hippocampi following development of amygdaloid kindling	1995	20	213-219	Epilepsy Res
Ueda, Y.; Tsuru, N.	Bilateral seizure-related changes of extracellular glutamate concentration in hippocampi during development of amygdaloid kindling	1994	18	85-88	Epilepsy Res

Glutamate					
**Authors**	**Title**	**Year**	**Vol.**	**Pages**	**Journal**
Ueda, Y.; Yokoyama, H.; Nakajima, A.; Tokumaru, J.; Doi, T.; Mitsuyama, Y.	Glutamate excess and free radical formation during and following kainic acid-induced status epilepticus	2002	147	219-226	Exp Brain Res
Ueda, Y.; Yokoyama, H.; Tokumaru, J.; Doi, T.; Nakajima, A.	Kinetics of extracellular nitroxide radical and glutamate levels in the hippocampus of conscious rats: Cautionary note to the application of nitroxide radical on clinical arena	2004	29	1695-1701	Neurochemical Research
Uezono, T.; Matsubara, K.; Shimizu, K.; Mizukami, H.; Ogawa, K.; Saito, O.; Hayase, N.; Eto, H.; Kimura, K.; Shiono, H.	Glutamate is not involved in the MPP+-induced dopamine overflow in the striatum of freely moving C57BL/6 mice	2001	108	899-908	J Neural Transm (Vienna)
Umbrain, V. J.; Lauwers, M. H.; Shi, L.; Smolders, I.; Michotte, Y.; Poelaert, J.	Comparison of the effects of intrathecal administration of levobupivacaine and lidocaine on the prostaglandin E2 and glutamate increases in cerebrospinal fluid: a microdialysis study in freely moving rats	2009	102	540-545	Br J Anaesth
Uutela, P.; Ketola, R. A.; Piepponen, P.; Kostiainen, R.	Comparison of different amino acid derivatives and analysis of rat brain microdialysates by liquid chromatography tandem mass spectrometry	2009	633	223-231	Anal Chim Acta
Vaccari, A.; Ferraro, L.; Saba, P.; Ruiu, S.; Mocci, I.; Antonelli, T.; Tanganelli, S.	Differential mechanisms in the effects of disulfiram and diethyldithiocarbamate intoxication on striatal release and vesicular transport of glutamate	1998	285	961-967	J Pharmacol Exp Ther
Valentino, K.; Newcomb, R.; Gadbois, T.; Singh, T.; Bowersox, S.; Bitner, S.; Justice, A.; Yamashiro, D.; Hoffman, B. B.; Ciaranello, R.; et al.	A selective N-type calcium channel antagonist protects against neuronal loss after global cerebral ischemia	1993	90	7894-7897	Proc Natl Acad Sci U S A
Valle-Dorado, M. G.; Santana-Gomez, C. E.; Orozco-Suarez, S. A.; Rocha, L.	The mast cell stabilizer sodium cromoglycate reduces histamine release and status epilepticus-induced neuronal damage in the rat hippocampus	2015	92	49-55	Neuropharmacology
Vallee, N.; Rostain, J. C.; Boussuges, A.; Risso, J. J.	Comparison of nitrogen narcosis and helium pressure effects on striatal amino acids: a microdialysis study in rats	2009	34	835-844	Neurochem Res
Vallee, N.; Rostain, J. C.; Risso, J. J.	A pressurized nitrogen counterbalance to cortical glutamatergic pathway stimulation	2010	35	718-726	Neurochem Res
Vallee, N.; Rostain, J. C.; Risso, J. J.	How can an inert gas counterbalance a NMDA-induced glutamate release?	2009	107	1951-1958	J Appl Physiol (1985)
Valtonen, P.; Haapalinna, A.; Riekkinen,	Effect of alpha 2-adrenergic drugs dexmedetomidine and	1995	285	239-246	Eur J Pharmacol

Glutamate					
**Authors**	**Title**	**Year**	**Vol.**	**Pages**	**Journal**
P., Sr.; Halonen, T.	atipamezole on extracellular amino acid levels in vivo				
Van Hemelrijck, A.; Hachimi-Idrissi, S.; Sarre, S.; Ebinger, G.; Michotte, Y.	Post-ischaemic mild hypothermia inhibits apoptosis in the penumbral region by reducing neuronal nitric oxide synthase activity and thereby preventing endothelin-1-induced hydroxyl radical formation	2005	22	1327-1337	Eur J Neurosci
Van Hemelrijck, A.; Hachimi-Idrissi, S.; Sarre, S.; Ebinger, G.; Michotte, Y.	Neuroprotective effect of N-acetyl-aspartyl-glutamate in combination with mild hypothermia in the endothelin-1 rat model of focal cerebral ischaemia	2005	95	1287-1297	J Neurochem
Van Hemelrijck, A.; Sarre, S.; Smolders, I.; Michotte, Y.	Determination of amino acids associated with cerebral ischaemia in rat brain microdialysates using narrowbore liquid chromatography and fluorescence detection	2005	144	63-71	J Neurosci Methods
Van Hemelrijck, A.; Vermijlen, D.; Hachimi-Idrissi, S.; Sarre, S.; Ebinger, G.; Michotte, Y.	Effect of resuscitative mild hypothermia on glutamate and dopamine release, apoptosis and ischaemic brain damage in the endothelin-1 rat model for focal cerebral ischaemia	2003	87	66-75	J Neurochem
van Os, S.; Ruitenbeek, W.; Hopman, J.; Klaessens, J.; van de Bor, M.	Cortical excitatory amino acid release and cell function during hypotension in near-term born lambs	2006	90	128-134	Biol Neonate
Varatharajan, R.; Joseph, K.; Neto, S. C.; Hofmann, U. G.; Moser, A.; Tronnier, V.	Electrical high frequency stimulation modulates GABAergic activity in the nucleus accumbens of freely moving rats	2015	90	255-260	Neurochem Int
Varga, V.; Kekesi, A.; Juhasz, G.; Kocsis, B.	Reduction of the extracellular level of glutamate in the median raphe nucleus associated with hippocampal theta activity in the anaesthetized rat	1998	84	49-57	Neuroscience
Vasylieva, N.; Maucler, C.; Meiller, A.; Viscogliosi, H.; Lieutaud, T.; Barbier, D.; Marinesco, S.	Immobilization method to preserve enzyme specificity in biosensors: consequences for brain glutamate detection	2013	85	2507-2515	Anal Chem
Vazquez, C. A.; Melecio, L. F.; Rivera, C. A.; Acosta, L. A.; Rodriguez, E. P.; Melendez, R. I.	Aniracetam treatment enhances spatial learning but not prefrontal glutamate release or ethanol consumption in adolescent C57BL/6J (B6) mice	2015	39	56A	Alcohol Clin Exp Res
Vazquez-DeRose, J.; Schwartz, M. D.; Nguyen, A. T.; Warrier, D. R.; Gulati, S.; Mathew, T. K.; Neylan, T. C.; Kilduff, T. S.	Hypocretin/orexin antagonism enhances sleep-related adenosine and GABA neurotransmission in rat basal forebrain	2016	221	923-940	Brain Struct Funct
Venero, C.; Borrell, J.	Rapid glucocorticoid effects on excitatory amino acid levels in the hippocampus: a microdialysis study in freely moving rats	1999	11	2465-2473	Eur J Neurosci
Venton, B. J.; Robinson, T. E.; Kennedy,	Transient changes in nucleus accumbens amino acid	2006	96	236-246	J Neurochem

Glutamate					
**Authors**	**Title**	**Year**	**Vol.**	**Pages**	**Journal**
R. T.	concentrations correlate with individual responsivity to the predator fox odor 2,5-dihydro-2,4,5-trimethylthiazoline				
Venton, B. J.; Robinson, T. E.; Kennedy, R. T.; Maren, S.	Dynamic amino acid increases in the basolateral amygdala during acquisition and expression of conditioned fear	2006	23	3391-3398	Eur J Neurosci
Vera, G.; Tapia, R.	Activation of group III metabotropic glutamate receptors by endogenous glutamate protects against glutamate-mediated excitotoxicity in the hippocampus in vivo	2012	90	1055-1066	J Neurosci Res
Vezzani, A.; Ungerstedt, U.; French, E. D.; Schwarcz, R.	In vivo brain dialysis of amino acids and simultaneous EEG measurements following intrahippocampal quinolinic acid injection: evidence for a dissociation between neurochemical changes and seizures	1985	45	335-344	J Neurochem
Vogels, B. A.; Karlsen, O. T.; Mass, M. A.; Bovee, W. M.; Chamuleau, R. A.	L-ornithine vs. L-ornithine-L-aspartate as a treatment for hyperammonemia-induced encephalopathy in rats	1997	26	174-182	J Hepatol
Voisin, D. L.; Herbison, A. E.; Chapman, C.; Poulain, D. A.	Effects of central GABAB receptor modulation upon the milk ejection reflex in the rat	1996	63	368-376	Neuroendocrinology
Volta, M.; Mabrouk, O. S.; Bido, S.; Marti, M.; Morari, M.	Further evidence for an involvement of nociceptin/orphanin FQ in the pathophysiology of Parkinson's disease: a behavioral and neurochemical study in reserpinized mice	2010	115	1543-1555	J Neurochem
Volta, M.; Marti, M.; McDonald, J.; Molinari, S.; Camarda, V.; Pela, M.; Trapella, C.; Morari, M.	Pharmacological profile and antiparkinsonian properties of the novel nociceptin/orphanin FQ receptor antagonist 1-[1-cyclooctylmethyl-5-(1-hydroxy-1-methyl-ethyl)-1,2,3,6-tetrahydro-pyridin-4-y l]-3-ethyl-1,3-dihydro-benzoimidazol-2-one (GF-4)	2010	31	1194-1204	Peptides
Volta, M.; Viaro, R.; Trapella, C.; Marti, M.; Morari, M.	Dopamine-nociceptin/orphanin FQ interactions in the substantia nigra reticulata of hemiparkinsonian rats: Involvement of D2/D3 receptors and impact on nigro-thalamic neurons and motor activity	2011	228	126-137	Exp Neurol
Vondrakova, K.; Syslova, K.; Mikoska, M.; Kacer, P.; Burchfiel, J.; Kubova, H.; Vales, K.; Tsenov, G.	The role of endothelin receptors in neurochemical changes during focal cerebral ischemia induced by intrahippocampal injection of the endothelin-1 in immature rats	2016	36	477	J Cereb Blood Flow Metab
Wade, J. V.; Olson, J. P.; Samson, F. E.; Nelson, S. R.; Pazdernik, T. L.	A possible role for taurine in osmoregulation within the brain	1988	51	740-745	J Neurochem
Wade, J. V.; Samson, F. E.; Nelson, S. R.; Pazdernik, T. L.	Changes in extracellular amino acids during soman- and kainic acid-induced seizures	1987	49	645-650	J Neurochem

Glutamate					
**Authors**	**Title**	**Year**	**Vol.**	**Pages**	**Journal**
Waeiss, R. A.; Deehan Jr, G. A.; Hauser, S. R.; Knight, C. P.; Engleman, E. A.; Rodd, Z. A.	Co-abuse of ethanol and nicotine increases basal glutamate levels within the nucleus accumbens shell	2015	39	112A	Alcohol Clin Exp Res
Wagner, Z.; Tabi, T.; Zachar, G.; Csillag, A.; Szoko, E.	Comparison of quantitative performance of three fluorescence labels in CE/LIF analysis of aspartate and glutamate in brain microdialysate	2011	32	2816-2822	Electrophoresis
Wahl, F.; Obrenovitch, T. P.; Hardy, A. M.; Plotkine, M.; Boulu, R.; Symon, L.	Extracellular glutamate during focal cerebral ischaemia in rats: time course and calcium dependency	1994	63	1003-1011	J Neurochem
Waldmeier, P. C.; Martin, P.; Stocklin, K.; Portet, C.; Schmutz, M.	Effect of carbamazepine, oxcarbazepine and lamotrigine on the increase in extracellular glutamate elicited by veratridine in rat cortex and striatum	1996	354	164-172	Naunyn Schmiedebergs Arch Pharmacol
Waldmeier, P. C.; Stocklin, K.; Feldtrauer, J. J.	Systemic administration of baclofen and the GABAB antagonist, CGP 35348, does not affect GABA, glutamate or aspartate in microdialysates of the striatum of conscious rats	1992	345	548-552	Naunyn Schmiedebergs Arch Pharmacol
Walker, R. H.; Davies, G.; Koch, R. J.; Haack, A. K.; Moore, C.; Meshul, C. K.	Effects of zona incerta lesions upon striatal neurochemistry and behavioural asymmetry in 6OHDA-lesioned rats	2010	25	S457	Mov Disord
Walker, R. H.; Davies, G.; Koch, R. J.; Haack, A. K.; Moore, C.; Meshul, C. K.	Effects of zona incerta lesions on striatal neurochemistry and behavioral asymmetry in 6-hydroxydopamine-lesioned rats	2010	88	2964-2975	J Neurosci Res
Walker, R. H.; Koch, R. J.; Sweeney, J. E.; Moore, C.; Meshul, C. K.	Effects of subthalamic nucleus lesions and stimulation upon glutamate levels in the dopamine-depleted rat striatum	2009	20	770-775	NeuroReport
Walker, R.; Sweeney, J.; Koch, R.; Moore, C.; Meshul, C.	Effects of subthalamic nucleus lesions and short-term stimulation upon striatal glutamate levels in awake intact and 6-hydroxy/dopamine-lesioned rats	2009	15	S104	Parkinsonism Relat Disord
Wang, B.; Shaham, Y.; Zitzman, D.; Azari, S.; Wise, R. A.; You, Z. B.	Cocaine experience establishes control of midbrain glutamate and dopamine by corticotropin-releasing factor: a role in stress-induced relapse to drug seeking	2005	25	5389-5396	J Neurosci
Wang, F.; Chen, H.; Sharp, B. M.	Neuroadaptive changes in the mesocortical glutamatergic system during chronic nicotine self-administration and after extinction in rats	2008	106	943-956	J Neurochem
Wang, J.; Peng, Y. J.; Zhu, D. N.	Amino acids modulate the hypotensive effect of angiotensin-(1-7) at the caudal ventrolateral medulla in rats	2005	129	1-7	Regul Pept
Wang, J.; Shen, L. L.; Cao, Y. X.; Sun, Z. J.; Wang, Q.; Zhu, D. N.	[Role of angiotensin-(1-7) in amino-acid-neurotransmitter-mediated blood pressure regulation in rat rostral ventrolateral	2001	53	1-6	Sheng Li Xue Bao

Glutamate					
Authors	Title	Year	Vol.	Pages	Journal
	medulla]				
Wang, J.; Shen, L. L.; Cao, Y. X.; Zhu, D. N.	Effects of electroacupuncture on pressor response to angiotensin-(1-7) by amino acid release in the rostral ventrolateral medulla	2003	28	25-34	Acupunct Electrother Res
Wang, L.; Huang, Y.; Wu, J.; Lv, G.; Zhou, L.; Jia, J.	Effect of Buyang Huanwu decoction on amino acid content in cerebrospinal fluid of rats during ischemic/reperfusion injury	2013	86	143-150	J Pharm Biomed Anal
Wang, L.; Maher, T. J.; Wurtman, R. J.	Oral L-glutamine increases GABA levels in striatal tissue and extracellular fluid	2007	21	1227-1232	Faseb J
Wang, S.; Fu, F. H.; Zhu, H.	The effects of chronic exercise on the autonomic balance of the cardiovascular system in hypertensive rats	2009	168	273-281	Gazz Med Ital Arch Sci Med (Torino)
Wang, S.; Hu, L. F.; Zhang, Y.; Sun, T.; Sun, Y. H.; Liu, S. Y.; Ding, J. H.; Wu, J.; Hu, G.	Effects of systemic administration of iptakalim on extracellular neurotransmitter levels in the striatum of unilateral 6-hydroxydopamine-lesioned rats	2006	31	933-940	Neuropsychopharmacology
Wang, S.; Pan, D. X.; Wang, D.; Wan, P.; Qiu, D. L.; Jin, Q. H.	Nitric oxide facilitates active avoidance learning via enhancement of glutamate levels in the hippocampal dentate gyrus	2014	271	177-183	Behav Brain Res
Wang, X.; Ai, J.; Hampson, D. R.; Snead, Iii O. C.	Altered glutamate and GABA release within thalamocortical circuitry in metabotropic glutamate receptor 4 knockout mice	2005	134	1195-1203	Neuroscience
Wang, X.; Shimizu-Sasamata, M.; Moskowitz, M. A.; Newcomb, R.; Lo, E. H.	Profiles of glutamate and GABA efflux in core versus peripheral zones of focal cerebral ischemia in mice	2001	313	121-124	Neurosci Lett
Wang, Y. K.; Shen, D.; Hao, Q.; Yu, Q.; Wu, Z. T.; Deng, Y.; Chen, Y. F.; Yuan, W. J.; Hu, Q. K.; Su, D. F.; Wang, W. Z.	Overexpression of angiotensin-converting enzyme 2 attenuates tonically active glutamatergic input to the rostral ventrolateral medulla in hypertensive rats	2014	307	H182-H190	Am J Physiol Heart Circ Physiol
Wang, Y. L.; He, R. R.	Effects of dipfluzine on cortical somatosensory evoked potentials and amino acid contents in ischemic rat brain	1996	17	41-44	Zhongguo Yao Li Xue Bao
Wang, Y.; Wang, D. Q.; Cui, Y.; Zhang, Y.; Sun, D. D.; Zhao, X. L.; Liu, Y.; Zhang, M. Y.; Jiao, Y.; Xu, X. J.; Xu, S.	[Analgesic effect of CQM on prosopalgia model rats and its impact on exciting amino acid neurotransmitters]	2013	38	3554-3559	Zhongguo Zhong Yao Za Zhi
Ward, R. J.; Colantuoni, C.; Dahchour, A.; Quertemont, E.; De Witte, P.	Acetaldehyde-induced changes in monoamine and amino acid extracellular microdialysate content of the nucleus accumbens	1997	36	225-232	Neuropharmacology
Ward, R. J.; Colivicchi, M. A.; Allen, R.; Schol, F.; Lallemand, F.; de Witte, P.; Ballini, C.; Corte, L. D.; Dexter, D.	Neuro-inflammation induced in the hippocampus of 'binge drinking'rats may be mediated by elevated extracellular glutamate content	2009	111	1119-1128	J Neurochem
Watson, C. J.	Insular balance of glutamatergic and GAB Aergic signaling	2016	157	2194-	Pain

Glutamate					
**Authors**	**Title**	**Year**	**Vol.**	**Pages**	**Journal**
	modulates pain processing			2207	
Watson, C. J.; Lydic, R.; Baghdoyan, H. A.	Sleep duration varies as a function of glutamate and GABA in rat pontine reticular formation	2011	118	571-580	J Neurochem
Watts, J.; Fowler, L.; Whitton, P. S.; Pearce, B.	Release of arginine, glutamate and glutamine in the hippocampus of freely moving rats: Involvement of nitric oxide	2005	65	521-528	Brain Res Bull
Wei, J.; Quast, M. J.	Effect of nitric oxide synthase inhibitor on a hyperglycemic rat model of reversible focal ischemia: detection of excitatory amino acids release and hydroxyl radical formation	1998	791	146-156	Brain Res
Wei, J.; Yao, L.; Yang, L.; Zhao, W.; Shi, S.; Cai, Q.; Chen, D.; Li, W.; Wang, Q.	Alteration of glutamate/GABA balance during acute alcohol intoxication in rats: effect of Xingnaojing injection	2015	166	333-339	J Ethnopharmacol
Wei, N.; Wang, Y.; Wang, X.; He, Z.; Zhang, M.; Zhang, X.; Pan, Y.; Zhang, J.; Qin, Z.; Zhang, K.	The different effects of high-frequency stimulation of the nucleus accumbens shell and core on food consumption are possibly associated with different neural responses in the lateral hypothalamic area	2015	301	312-322	Neuroscience
Welsch-Kunze, S.; Kleim, J.; Dietze, S.; Kuschinsky, K.	Recordings of extracellular glutamate and gamma-aminobutyric acid in the striatum of non-anesthetized rats. K(+)-stimulation, its Ca(2+)-dependence and lack of effects of drugs acting on dopamine receptors	1993	43	85-91	Arzneimittelforschung
Welty, N.; Shoblock, J. R.	The effects of thioperamide on extracellular levels of glutamate and GABA in the rat prefrontal cortex	2009	207	433-438	Psychopharmacology (Berl)
Wen, Z. H.; Wu, G. J.; Chang, Y. C.; Wang, J. J.; Wong, C. S.	Dexamethasone modulates the development of morphine tolerance and expression of glutamate transporters in rats	2005	133	807-817	Neuroscience
West, A. R.; Galloway, M. P.	Inhibition of glutamate reuptake potentiates endogenous nitric oxide-facilitated dopamine efflux in the rat striatum: an in vivo microdialysis study	1997	230	21-24	Neurosci Lett
West, A. R.; Galloway, M. P.	Endogenous nitric oxide facilitates striatal dopamine and glutamate efflux in vivo: role of ionotropic glutamate receptor-dependent mechanisms	1997	36	1571-1581	Neuropharmacology
Westergren, I.; Nystrom, B.; Hamberger, A.; Johansson, B. B.	Intracerebral dialysis and the blood-brain barrier	1995	64	229-234	J Neurochem
Westergren, I.; Nystrom, B.; Hamberger, A.; Johansson, B. B.	Amino acids in extracellular fluid in vasogenic brain edema	1994	60	124-127	Acta Neurochir Suppl (Wien)
Westergren, I.; Nystrom, B.; Hamberger,	Concentrations of amino acids in extracellular fluid after opening	1994	62	159-165	J Neurochem

Glutamate					
**Authors**	**Title**	**Year**	**Vol.**	**Pages**	**Journal**
A.; Nordborg, C.; Johansson, B. B.	of the blood-brain barrier by intracarotid infusion of protamine sulfate				
Westerink, B. H.; Damsma, G.; de Vries, J. B.	Effect of ouabain applied by intrastriatal microdialysis on the in vivo release of dopamine, acetylcholine, and amino acids in the brain of conscious rats	1989	52	705-712	J Neurochem
Westerink, B. H.; Hofsteede, H. M.; Damsma, G.; de Vries, J. B.	The significance of extracellular calcium for the release of dopamine, acetylcholine and amino acids in conscious rats, evaluated by brain microdialysis	1988	337	373-378	Naunyn Schmiedebergs Arch Pharmacol
Westermaier, T.; Jauss, A.; Eriskat, J.; Kunze, E.; Roosen, K.	The temporal profile of cerebral blood flow and tissue metabolites indicates sustained metabolic depression after experimental subarachnoid hemorrhage in rats	2011	68	223-230	Neurosurgery
Williams, J. M.; Steketee, J. D.	Cocaine increases medial prefrontal cortical glutamate overflow in cocaine-sensitized rats: a time course study	2004	20	1639-1646	Eur J Neurosci
Wilson, C. L.; Maidment, N. T.; Shomer, M. H.; Behnke, E. J.; Ackerson, L.; Fried, I.; Engel, J., Jr.	Comparison of seizure related amino acid release in human epileptic hippocampus versus a chronic, kainate rat model of hippocampal epilepsy	1996	26	245-254	Epilepsy Res
Windels, F.; Bruet, N.; Poupard, A.; Feuerstein, C.; Bertrand, A.; Savasta, M.	Influence of the frequency parameter on extracellular glutamate and gamma-aminobutyric acid in substantia nigra and globus pallidus during electrical stimulation of subthalamic nucleus in rats	2003	72	259-267	J Neurosci Res
Windels, F.; Bruet, N.; Poupard, A.; Urbain, N.; Chouvet, G.; Feuerstein, C.; Savasta, M.	Effects of high frequency stimulation of subthalamic nucleus on extracellular glutamate and GABA in substantia nigra and globus pallidus in the normal rat	2000	12	4141-4146	Eur J Neurosci
Windels, F.; Carcenac, C.; Poupard, A.; Savasta, M.	Pallidal origin of GABA release within the substantia nigra pars reticulata during high-frequency stimulation of the subthalamic nucleus	2005	25	5079-5086	J Neurosci
Winfree, C. J.; Baker, C. J.; Connolly, E. S., Jr.; Fiore, A. J.; Solomon, R. A.	Mild hypothermia reduces penumbral glutamate levels in the rat permanent focal cerebral ischemia model	1996	38	1216-1222	Neurosurgery
Win-Shwe, T. T.; Mitsushima, D.; Nakajima, D.; Ahmed, S.; Yamamoto, S.; Tsukahara, S.; Kakeyama, M.; Goto, S.; Fujimaki, H.	Toluene induces rapid and reversible rise of hippocampal glutamate and taurine neurotransmitter levels in mice	2007	168	75-82	Toxicol Lett
Win-Shwe, T. T.; Mitsushima, D.;	Extracellular glutamate level and NMDA receptor subunit	2009	21	828-836	Inhal Toxicol

Glutamate					
**Authors**	**Title**	**Year**	**Vol.**	**Pages**	**Journal**
Yamamoto, S.; Fujitani, Y.; Funabashi, T.; Hirano, S.; Fujimaki, H.	expression in mouse olfactory bulb following nanoparticle-rich diesel exhaust exposure				
Win-Shwe, T. T.; Mitsushima, D.; Yamamoto, S.; Funabashi, T.; Fujimaki, H.	Strain differences in extracellular amino acid neurotransmitter levels in the hippocampi of major histocompatibility complex congenic mice in response to toluene exposure	2009	16	185-190	Neuroimmunomodulation
Wise, R. A.; Wang, B.; You, Z. B.	Cocaine serves as a peripheral interoceptive conditioned stimulus for central glutamate and dopamine release	2008	3	e2846	PloS One
Wislowska-Stanek, A.; Hamed, A.; Lehner, M.; Bidzinski, A.; Turzynska, D.; Sobolewska, A.; Walkowiak, J.; Plaznik, A.	Effects of midazolam and buspirone on in vivo concentration of amino acids and monoamine metabolites in the rat hippocampus	2008	60	209-218	Pharmacol Rep
Wolf, M. E.; Xue, C. J.	Amphetamine and D1 dopamine receptor agonists produce biphasic effects on glutamate efflux in rat ventral tegmental area: modification by repeated amphetamine administration	1998	70	198-209	J Neurochem
Wolf, M. E.; Xue, C. J.	Amphetamine-induced glutamate efflux in the rat ventral tegmental area is prevented by MK-801, SCH 23390, and ibotenic acid lesions of the prefrontal cortex	1999	73	1529-1538	J Neurochem
Wolf, M. E.; Xue, C. J.; Li, Y.; Wavak, D.	Amphetamine increases glutamate efflux in the rat ventral tegmental area by a mechanism involving glutamate transporters and reactive oxygen species	2000	75	1634-1644	J Neurochem
Wolfram-Aduan, A.; Altemus, M.; Wickwire, J. H.; Sandstrom, M. I.	Presymptomatic glutamate levels in prefrontal cortex in the Hdh(CAG150) mouse model of Huntington's disease	2014	3	387-399	J Huntingtons Dis
Wong, J. M.; Malec, P. A.; Mabrouk, O. S.; Ro, J.; Dus, M.; Kennedy, R. T.	Benzoyl chloride derivatization with liquid chromatography-mass spectrometry for targeted metabolomics of neurochemicals in biological samples	2016	1446	78-90	J Chromatogr A
Wozniak, M.; Golembiowska, K.; Noworyta-Sokolowska, K.; Acher, F.; Cieslik, P.; Kusek, M.; Tokarski, K.; Pilc, A.; Wieronska, J. M.	Neurochemical and behavioral studies on the 5-HT1A-dependent antipsychotic action of the mGlu4 receptor agonist LSP4-2022	2016	14	413-426	Neuropharmacology
Wu, G.; Kim, H. K.; Zornow, M. H.	Transient brain ischemia in rabbits: the effect of omega-conopeptide MVIIC on hippocampal excitatory amino acids	1995	692	118-122	Brain Res
Wu, H. Q.; Okuyama, M.; Kajii, Y.; Pocivavsek, A.; Bruno, J. P.; Schwarcz, R.	Targeting kynurenine aminotransferase II in psychiatric diseases: promising effects of an orally active enzyme inhibitor	2014	40 Suppl 2	S152-S158	Schizophr Bull
Wu, H. Q.; Pereira, E. F.; Bruno, J. P.;	The astrocyte-derived alpha7 nicotinic receptor antagonist	2010	40	204-210	J Mol Neurosci

Glutamate					
**Authors**	**Title**	**Year**	**Vol.**	**Pages**	**Journal**
Pellicciari, R.; Albuquerque, E. X.; Schwarcz, R.	kynurenic acid controls extracellular glutamate levels in the prefrontal cortex				
Wu, Z. E.; Wang, D. Q.; Li, P.; Sun, X. F.; Wang, W.	The method of simultaneous measurement of the plasma concentration of L-DOPA and extracellular amino acids in striatum of the Parkinson's disease rats induced by 6-OHDA. [Chinese]	2010	26	1395-1399	Zhongguo yao li xue tong bao
Wydra, K.; Golembiowska, K.; Suder, A.; Kaminska, K.; Fuxe, K.; Filip, M.	On the role of adenosine (A)2A receptors in cocaine-induced reward: A pharmacological and neurochemical analysis in rats	2015	232	421-435	Psychopharmacology
Wydra, K.; Golembiowska, K.; Zaniewska, M.; Kaminska, K.; Ferraro, L.; Fuxe, K.; Filip, M.	Accumbal and pallidal dopamine, glutamate and GABA overflow during cocaine self-administration and its extinction in rats	2013	18	307-324	Addict Biol
Xi, Z. X.; Baker, D. A.; Shen, H.; Carson, D. S.; Kalivas, P. W.	Group II metabotropic glutamate receptors modulate extracellular glutamate in the nucleus accumbens	2002	300	162-171	J Pharmacol Exp Ther
Xi, Z. X.; Li, X.; Peng, X. Q.; Li, J.; Chun, L.; Gardner, E. L.; Thomas, A. G.; Slusher, B. S.; Ashby, C. R., Jr.	Inhibition of NAALADase by 2-PMPA attenuates cocaine-induced relapse in rats: a NAAG-mGluR2/3-mediated mechanism	2010	112	564-576	J Neurochem
Xi, Z. X.; Ramamoorthy, S.; Baker, D. A.; Shen, H.; Samuvel, D. J.; Kalivas, P. W.	Modulation of group II metabotropic glutamate receptor signaling by chronic cocaine	2002	303	608-615	J Pharmacol Exp Ther
Xi, Z. X.; Shen, H.; Baker, D. A.; Kalivas, P. W.	Inhibition of non-vesicular glutamate release by group III metabotropic glutamate receptors in the nucleus accumbens	2003	87	1204-1212	J Neurochem
Xia, C. M.; Shao, C. H.; Xin, L.; Wang, Y. R.; Ding, C. N.; Wang, J.; Shen, L. L.; Li, L.; Cao, Y. X.; Zhu, D. N.	Effects of melatonin on blood pressure in stress-induced hypertension in rats	2008	35	1258-1264	Clin Exp Pharmacol Physiol
Xie, F.; Li, X.; Bao, M.; Shi, R.; Yue, Y.; Guan, Y.; Wang, Y.	Anesthetic propofol normalized the increased release of glutamate and gamma-amino butyric acid in hippocampus after paradoxical sleep deprivation in rats	2015	37	1102-1107	Neurol Res
Xie, H. S.; Song, W. M.; Shangguan, D. H.; Li, X. P.; Wang, W.; Wang, L.; Zhu, L.	[Level of glutamate on the auditory pathway of inferior colliculus after exposure to noise observed by microdialysis in vivo]	2007	39	409-411	Beijing Da Xue Xue Bao
Xie, X.; Steketee, J. D.	Repeated exposure to cocaine alters the modulation of mesocorticolimbic glutamate transmission by medial prefrontal cortex Group II metabotropic glutamate receptors	2008	107	186-196	J Neurochem
Xu, F.; Wang, L.; Gao, M.; Jin, L.; Jin, J.	Amperometric sensor for glucose and hypoxanthine based on a PdIrO(2) modified electrode by a co-crosslinking bienzymic	2002	57	365-373	Talanta

Glutamate					
**Authors**	**Title**	**Year**	**Vol.**	**Pages**	**Journal**
	system				
Xu, G. Y.; McAdoo, D. J.; Hughes, M. G.; Robak, G.; De Castro Jr, R.	Considerations in the determination by microdialysis of resting extracellular amino acid concentrations and release upon spinal cord injury	1998	86	1011-1021	Neuroscience
Xu, P.; Li, J.; Chen, B.; Wang, X.; Cai, X.; Jiang, H.; Wang, C.; Zhang, H.	The real-time neurotoxicity analysis of Fe3O4 nanoparticles combined with daunorubicin for rat brain in vivo	2012	8	417-423	J Biomed Nanotechnol
Xu, X. H.; Zheng, X. X.; Li, H.	Effects of puerarin on excitatory amino acid levels in cerebral ischemic striatum of rat. [Chinese]	2008	43	675-679	Zhongguo yao li xue tong bao
Xue, C. J.; Ng, J. P.; Li, Y.; Wolf, M. E.	Acute and repeated systemic amphetamine administration: effects on extracellular glutamate, aspartate, and serine levels in rat ventral tegmental area and nucleus accumbens	1996	67	352-363	J Neurochem
Yablonsky-Alter, E.; Agovic, M. S.; Gashi, E.; Lidsky, T. I.; Friedman, E.; Banerjee, S. P.	Cocaine challenge enhances release of neuroprotective amino acid taurine in the striatum of chronic cocaine treated rats: a microdialysis study	2009	79	215-218	Brain Res Bull
Yager, J. Y.; Armstrong, E. A.; Miyashita, H.; Wirrell, E. C.	Prolonged neonatal seizures exacerbate hypoxic-ischemic brain damage: correlation with cerebral energy metabolism and excitatory amino acid release	2002	24	367-381	Dev Neurosci
Yamada, K.; Moriguchi, A.; Mikami, H.; Okuda, N.; Higaki, J.; Ogihara, T.	The effect of central amino acid neurotransmitters on the antihypertensive response to angiotensin blockade in spontaneous hypertension	1995	13	1624-1630	J Hypertens
Yamada, T.; Terashima, T.; Kawano, S.; Furuno, R.; Okubo, T.; Juneja, L. R.; Yokogoshi, H.	Theanine, gamma-glutamylethylamide, a unique amino acid in tea leaves, modulates neurotransmitter concentrations in the brain striatum interstitium in conscious rats	2009	36	21-27	Amino Acids
Yamada, T.; Terashima, T.; Okubo, T.; Juneja, L. R.; Yokogoshi, H.	Effects of theanine, r-glutamylethylamide, on neurotransmitter release and its relationship with glutamic acid neurotransmission	2005	8	219-226	Nutr Neurosci
Yamada, T.; Zuo, D.; Yamamoto, T.; Olszewski, R. T.; Bzdega, T.; Moffett, J. R.; Neale, J. H.	NAAG peptidase inhibition in the periaqueductal gray and rostral ventromedial medulla reduces flinching in the formalin model of inflammation	2012	8	/	Mol Pain
Yamagami, S.; Miyamoto, O.; Nakamura, T.; Okada, Y.; Negi, T.; Hayashida, Y.; Nagao, S.; Itano, T.	Suppression of hyperemia after brain ischemia by L-threo-3,4-dihydroxyphenylserine	1998	9	2939-2943	Neuroreport
Yamamoto, B. K.; Cooperman, M. A.	Differential effects of chronic antipsychotic drug treatment on extracellular glutamate and dopamine concentrations	1994	14	4159-4166	J Neurosci

Glutamate					
**Authors**	**Title**	**Year**	**Vol.**	**Pages**	**Journal**
Yamamoto, B. K.; Davy, S.	Dopaminergic modulation of glutamate release in striatum as measured by microdialysis	1992	58	1736-1742	J Neurochem
Yamamoto, H.; Mitani, A.; Cui, Y.; Takechi, S.; Irita, J.; Suga, T.; Arai, T.; Kataoka, K.	Neuroprotective effect of mild hypothermia cannot be explained in terms of a reduction of glutamate release during ischemia	1999	91	501-509	Neuroscience
Yamamoto, K.; Chan, S. W.; Rudd, J. A.; Lin, G.; Asano, K.; Yamatodan, A.	Involvement of hypothalamic glutamate in cisplatin-induced emesis in suncus murinus (house musk shrew)	2009	109	631-834	J Pharmacol Sci
Yamamoto, Y.; Kakigi, T.; Maeda, K.	Intra-striatal phencyclidine inhibits N-methyl-D-aspartic acid-stimulated increase in glutamate levels of freely moving rats	1999	23	161-174	Prog Neuropsychopharmacol Biol Psychiatry
Yamamura, S.; Hamaguchi, T.; Ohoyama, K.; Sugiura, Y.; Suzuki, D.; Kanehara, S.; Nakagawa, M.; Motomura, E.; Matsumoto, T.; Tanii, H.; Shiroyama, T.; Okada, M.	Topiramate and zonisamide prevent paradoxical intoxication induced by carbamazepine and phenytoin	2009	84	172-186	Epilepsy Res
Yamamura, S.; Hoshikawa, M.; Dai, K.; Saito, H.; Suzuki, N.; Niwa, O.; Okada, M.	ONO-2506 inhibits spike-wave discharges in a genetic animal model without affecting traditional convulsive tests via gliotransmission regulation	2013	168	1088-1100	Br J Pharmacol
Yamamura, S.; Ohoyama, K.; Hamaguchi, T.; Kashimoto, K.; Nakagawa, M.; Kanehara, S.; Suzuki, D.; Matsumoto, T.; Motomura, E.; Shiroyama, T.; Okada, M.	Effects of quetiapine on monoamine, GABA, and glutamate release in rat prefrontal cortex	2009	206	243-258	Psychopharmacology (Berl)
Yamamura, S.; Ohoyama, K.; Hamaguchi, T.; Nakagawa, M.; Suzuki, D.; Matsumoto, T.; Motomura, E.; Tanii, H.; Shiroyama, T.; Okada, M.	Effects of zotepine on extracellular levels of monoamine, GABA and glutamate in rat prefrontal cortex	2009	157	656-665	Br J Pharmacol
Yamamura, S.; Ohoyama, K.; Nagase, H.; Okada, M.	Zonisamide enhances delta receptor-associated neurotransmitter release in striato-pallidal pathway	2009	57	322-331	Neuropharmacology
Yamamura, S.; Saito, H.; Suzuki, N.; Kashimoto, S.; Hamaguchi, T.; Ohoyama, K.; Suzuki, D.; Kanehara, S.; Nakagawa, M.; Shiroyama, T.; Okada, M.	Effects of zonisamide on neurotransmitter release associated with inositol triphosphate receptors	2009	454	91-96	Neurosci Lett
Yamanaka, T.	[Excitatory effects of glucocorticoids on neuronal activity in the medial vestibular nucleus--mediation by glucocorticoid receptor on the membrane]	1994	97	855-867	Nihon Jibiinkoka Gakkai Kaiho

Glutamate					
**Authors**	**Title**	**Year**	**Vol.**	**Pages**	**Journal**
Yamanaka, T.; Sasa, M.; Matsunaga, T.	Glutamate as a primary afferent neurotransmitter in the medial vestibular nucleus as detected by in vivo microdialysis	1997	762	243-246	Brain Res
Yamanaka, T.; Sasa, M.; Matsunaga, T.	Release of glutamate from the vestibular nerve in the medial vestibular nucleus as a neurotransmitter: in vivo microdialysis study	1995	520Pt1	92-93	Acta Otolaryngol Suppl
Yamashita, A.; Hamada, A.; Suhara, Y.; Kawabe, R.; Yanase, M.; Kuzumaki, N.; Matsui, R.; Okano, H.; Narita, M.	Astrocytic activation in the anterior cingulate cortex is critical for sleep disorder under neuropathic pain	2014	68	235-247	Synapse
Yamashita, A.; Matsumoto, M.; Matsumoto, S.; Itoh, M.; Kawai, K.; Sakabe, T.	A comparison of the neurotoxic effects on the spinal cord of tetracaine, lidocaine, bupivacaine, and ropivacaine administered intrathecally in rabbits	2003	97	512-519	Anesth Analg
Yan, E. B.; Hellewell, S. C.; Bellander, B. M.; Agyapomaa, D. A.; Morganti-Kossmann, M. C.	Post-traumatic hypoxia exacerbates neurological deficit, neuroinflammation and cerebral metabolism in rats with diffuse traumatic brain injury	2011	8	147	J Neuroinflammation
Yan, H. T.; Wu, N.; Lu, X. Q.; Su, R. B.; Zheng, J. Q.; Li, J.	Aquaporin-4 deficiency attenuates opioid dependence through suppressing glutamate transporter-1 down-regulation and maintaining glutamate homeostasis	2013	19	12-19	CNS Neurosci Ther
Yan, N.; Chen, N.; Zhu, H.; Zhang, J.; Sim, M.; Ma, Y.; Wang, W.	High-frequency stimulation of nucleus accumbens changes in dopaminergic reward circuit	2013	8	e79318	Plos One
Yan, Q. S.; Reith, M. E.; Yan, S. G.; Jobe, P. C.	Effect of systemic ethanol on basal and stimulated glutamate releases in the nucleus accumbens of freely moving Sprague-Dawley rats: a microdialysis study	1998	258	29-32	Neurosci Lett
Yananli, H. R.; Terzioglu, B.; Goren, M. Z.; Aker, R. G.; Aypak, C.; Onat, F. Y.	Extracellular hypothalamic gamma-aminobutyric acid (GABA) and L-glutamic acid concentrations in response to bicuculline in a genetic absence epilepsy rat model	2008	106	301-309	J Pharmacol Sci
Yang, C. S.; Lin, N. N.; Liu, L.; Tsai, P. J.; Kuo, J. S.	Lowered brain glutathione by diethylmaleate decreased the glutamate release induced by cerebral ischemia in anesthetized rats	1995	698	237-240	Brain Res
Yang, C. S.; Lin, N. N.; Tsai, P. J.; Liu, L.; Kuo, J. S.	In vivo evidence of hydroxyl radical formation induced by elevation of extracellular glutamate after cerebral ischemia in the cortex of anesthetized rats	1996	20	245-250	Free Radic Biol Med
Yang, C. S.; Tsai, P. J.; Chen, W. Y.; Tsai, W. J.; Kuo, J. S.	On-line derivatization for continuous and automatic monitoring of brain extracellular glutamate levels in anesthetized rats: a	1999	734	1-6	J Chromatogr B Biomed Sci Appl

Glutamate					
Authors	Title	Year	Vol.	Pages	Journal
	microdialysis study				
Yang, C. S.; Tsai, P. J.; Lin, N. N.; Kuo, J. S.	Diethylmaleate decreased ascorbic acid release induced by cerebral ischemia in cerebral cortex of the anesthetized rat	2000	43	49-53	Chin J Physiol
Yang, C. S.; Tsai, P. J.; Lin, N. N.; Kuo, J. S.	Elevated extracellular glutamate concentrations increased malondialdehyde production in anesthetized rat brain cortex	1998	243	33-36	Neurosci Lett
Yang, D. Y.; Tsai, T. H.; Cheng, C. H.; Lee, C. W.; Chen, S. H.; Cheng, F. C.	Simultaneous monitoring of extracellular glucose, pyruvate, lactate and glutamate in gerbil cortex during focal cerebral ischemia by dual probe microdialysis	2001	913	349-354	J Chromatogr A
Yang, J.; Hu, L. F.; Liu, X.; Zhou, F.; Ding, J. H.; Hu, G.	Effects of iptakalim on extracellular glutamate and dopamine levels in the striatum of unilateral 6-hydroxydopamine-lesioned rats: a microdialysis study	2006	78	1940-1944	Life Sci
Yang, P. S.; Kim, H.; Lee, W.; Bohlke, M.; Park, S.; Maher, T. J.; Yoo, S. S.	Transcranial focused ultrasound to the thalamus is associated with reduced extracellular GABA levels in rats	2010	65	153-160	Neuropsychobiology
Yang, X.; He, Z.; Zhang, Q.; Wu, Y.; Hu, Y.; Wang, X.; Li, M.; Wu, Z.; Guo, Z.; Guo, J.; Jia, J.	Pre-ischemic treadmill training for prevention of ischemic brain injury via regulation of glutamate and its transporter GLT-1	2012	13	9447-9459	Int J Mol Sci
Yang, Y.; Li, Q.; Miyashita, H.; Yang, T.; Shuaib, A.	Different dynamic patterns of extracellular glutamate release in rat hippocampus after permanent or 30-min transient cerebral ischemia and histological correlation	2001	21	181-187	Neuropathology
Yang, Z. J.; Xie, Y.; Bosco, G. M.; Chen, C.; Camporesi, E. M.	Hyperbaric oxygenation alleviates MCAO-induced brain injury and reduces hydroxyl radical formation and glutamate release	2010	108	513-522	Eur J Appl Physiol
Yang, Z. Z.; Li, J.; Li, S. X.; Feng, W.; Wang, H.	Effect of ginkgolide B on striatal extracellular amino acids in middle cerebral artery occluded rats	2011	136	117-122	J Ethnopharmacol
Yano, T.; Nakayama, R.; Ushijima, K.	Intracerebroventricular propofol is neuroprotective against transient global ischemia in rats: extracellular glutamate level is not a major determinant	2000	883	69-76	Brain Res
Yao, H.; Matsumoto, T.; Hirano, M.; Uchimura, H.; Ooboshi, H.; Sadoshima, S.; Fujishima, M.	Striatal glutamic acid and gamma-aminobutyric acid in transient cerebral ischemia in spontaneously hypertensive rats	1990	31	385-392	Jpn Heart J
Yao, H.; Ooboshi, H.; Ibayashi, S.; Uchimura, H.; Fujishima, M.	Cerebral blood flow and ischemia-induced neurotransmitter release in the striatum of aged spontaneously hypertensive rats	1993	24	577-580	Stroke
Yasumatsu, M.; Yazawa, T.; Otokawa, M.; Kuwasawa, K.; Hasegawa, H.; Aihara, Y.	Monoamines, amino acids and acetylcholine in the preoptic area and anterior hypothalamus of rats: measurements of tissue	1998	21	13-23	Comp Biochem Physiol A Mol Integr Physiol

Glutamate					
**Authors**	**Title**	**Year**	**Vol.**	**Pages**	**Journal**
	extracts and in vivo microdialysates				
Yelkenli, I. H.; Ulupinar, E.; Korkmaz, O. T.; Sener, E.; Kus, G.; Filiz, Z.; Tuncel, N.	Modulation of Corpus Striatal Neurochemistry by Astrocytes and Vasoactive Intestinal Peptide (VIP) in Parkinsonian Rats	2016	59	280-289	J Mol Neurosci
Yin, P. B.; Li, B. M.; Ye, W. L.; Mei, Z. T.	[A microdialysis study of excitatory amino acid levels of the monkeys'caudate nucleus during the delayed go/no-go task]	1997	49	128-134	Sheng Li Xue Bao
Yokoyama, M.; Suzuki, E.; Sato, T.; Maruta, S.; Watanabe, S.; Miyaoka, H.	Amygdalic levels of dopamine and serotonin rise upon exposure to conditioned fear stress without elevation of glutamate	2005	379	37-41	Neurosci Lett
Yonezawa, Y.; Hondo, H.; Hashimoto, K.; Matsumoto, T.; Hirano, M.; Uchimura, H.; Kuroki, T.	Effect of phencyclidine on endogenous excitatory amino acid release from the rat anterior cingulate cortex--an in vivo microdialysis study	1993	94	235-240	J Neural Transm Gen Sect
Yoshida, S.; Okada, M.; Zhu, G.; Kaneko, S.	Carbamazepine prevents breakdown of neurotransmitter release induced by hyperactivation of ryanodine receptor	2007	52	1538-1546	Neuropharmacology
Yoshida, S.; Okada, M.; Zhu, G.; Kaneko, S.	Effects of zonisamide on neurotransmitter exocytosis associated with ryanodine receptors	2005	67	153-162	Epilepsy Res
Yoshioka, H.; Sugita, M.; Kinouchi, H.	Neuroprotective effects of group II metabotropic glutamate receptor agonist DCG-IV on hippocampal neurons in transient forebrain ischemia	2009	461	266-270	Neurosci Lett
Yoshioka, H.; Sugita, M.; Yagi, T.; Horikoshi, T.; Kinouchi, H.	Effects of group II metabotropic glutamate receptor activation on neuroprotection and extracellular glutamate concentration after transient forebrain ischemia	2007	27	BO09-BO02	J Cereb Blood Flow Metab
You, Z. B.; Chen, Y. Q.; Wise, R. A.	Dopamine and glutamate release in the nucleus accumbens and ventral tegmental area of rat following lateral hypothalamic self-stimulation	2001	107	629-639	Neuroscience
You, Z. B.; Godukhin, O.; Goiny, M.; Nylander, I.; Ungerstedt, U.; Terenius, L.; Hokfelt, T.; Herrera-Marschitz, M.	Cholecystokinin-8S increases dynorphin B, aspartate and glutamate release in the fronto-parietal cortex of the rat via different receptor subtypes	1997	355	576-581	Naunyn Schmiedebergs Arch Pharmacol
You, Z. B.; Herrera-Marschitz, M.; Brodin, E.; Meana, J. J.; Morino, P.; Hokfelt, T.; Silveira, R.; Goiny, M.; Ungerstedt, U.	On the origin of striatal cholecystokinin release: studies with in vivo microdialysis	1994	62	76-85	J Neurochem
You, Z. B.; Herrera-Marschitz, M.; Nylander, I.; Goiny, M.; Kehr, J.; Ungerstedt, U.; Terenius, L.	Effect of morphine on dynorphin B and GABA release in the basal ganglia of rats	1996	710	241-248	Brain Res
You, Z. B.; Herrera-Marschitz, M.;	Modulation of neurotransmitter release by cholecystokinin in the	1996	74	793-804	Neuroscience

Glutamate					
**Authors**	**Title**	**Year**	**Vol.**	**Pages**	**Journal**
Pettersson, E.; Nylander, I.; Goiny, M.; Shou, H. Z.; Kehr, J.; Godukhin, O.; Hokfelt, T.; Terenius, L.; Ungerstedt, U.	neostriatum and substantia nigra of the rat: regional and receptor specificity				
You, Z. B.; Herrera-Marschitz, M.; Terenius, L.	Modulation of neurotransmitter release in the basal ganglia of the rat brain by dynorphin peptides	1999	290	1307-1315	J Pharmacol Exp Ther
You, Z. B.; Pettersson, E.; Herrera-Marschitz, M.; Hokfelt, T.; Terenius, L.; Nylander, I.; Goiny, M.; Hughes, J.; Connor, W. T.; Ungerstedt, U.	Modulation of striatal aspartate and dynorphin B release by cholecystokinin (CCK-8) studied in vivo with microdialysis	1994	5	2301-2304	Neuroreport
You, Z. B.; Saria, A.; Fischer-Colbrie, R.; Terenius, L.; Goiny, M.; Herrera-Marschitz, M.	Effects of secretogranin II-derived peptides on the release of neurotransmitters monitored in the basal ganglia of the rat with in vivo microdialysis	1996	354	717-724	Naunyn Schmiedebergs Arch Pharmacol
You, Z. B.; Tzschentke, T. M.; Brodin, E.; Wise, R. A.	Electrical stimulation of the prefrontal cortex increases cholecystokinin, glutamate, and dopamine release in the nucleus accumbens: an in vivo microdialysis study in freely moving rats	1998	18	6492-6500	J Neurosci
You, Z. B.; Wang, B.; Zitzman, D.; Azari, S.; Wise, R. A.	A role for conditioned ventral tegmental glutamate release in cocaine seeking	2007	27	10546-10555	J Neurosci
Young, A. M. J.; Crowder, J. M.; Bradford, H. F.	Kainate potentiates excitatory amino acid release in striatum	1987	15	635-636	Biochem Soc Trans
Young, A. M.; Bradford, H. F.	N-methyl-D-aspartate releases excitatory amino acids in rat corpus striatum in vivo	1991	56	1677-1683	J Neurochem
Young, A. M.; Bradford, H. F.	Excitatory amino acid neurotransmitters in the corticostriate pathway: studies using intracerebral microdialysis in vivo	1986	47	1399-1404	J Neurochem
Young, A. M.; Bradford, H. F.	N-methyl-D-aspartate releases gamma-aminobutyric acid from rat striatum in vivo: a microdialysis study using a novel preloading method	1993	60	487-492	J Neurochem
Young, A. M.; Foley, P. M.; Bradford, H. F.	Preloading in vivo: a rapid and reliable method for measuring gamma-aminobutyric acid and glutamate fluxes by microdialysis	1990	55	1060-1063	J Neurochem
Young, R. S.; During, M. J.; Aquila, W. J.; Tendler, D.; Ley, E.	Hypoxia increases extracellular concentrations of excitatory and inhibitory neurotransmitters in subsequently induced seizure: in vivo microdialysis study in the rabbit	1992	117	204-209	Exp Neurol
Yu, H. L.; An, Y.; Bing, Y. H.; Jin, Q. H.; Cui, X.; Jin, Y. Z.	[Changes of glutamate and taurine released in the medial vestibular nucleus following acute hypotension]	2006	58	117-182	Sheng Li Xue Bao

Glutamate					
**Authors**	**Title**	**Year**	**Vol.**	**Pages**	**Journal**
Yu, H. L.; An, Y.; Jiang, H. Y.; Jin, Q. H.; Jin, Y. Z.	Changes of amino acid concentrations in the rat medial vestibular nucleus following unilateral labyrinthectomy	2007	59	71-78	Sheng Li Xue Bao
Yu, Y.; Liu, X.; Jiang, D.; Sun, Q.; Zhou, T.; Zhu, M.; Jin, L.; Shi, G.	[C3(OH)2mim][BF4]-Au/Pt biosensor for glutamate sensing in vivo integrated with on-line microdialysis system	2011	26	3227-3232	Biosens Bioelectron
Yu, Y.; Sun, Q.; Zhou, T.; Zhu, M.; Jin, L.; Shi, G.	On-line microdialysis system with poly(amidoamine)-encapsulated Pt nanoparticles biosensor for glutamate sensing in vivo	2011	81	53-57	Bioelectrochemistry
Yusa, T.	Increased extracellular ascorbate release reflects glutamate re-uptake during the early stage of reperfusion after forebrain ischemia in rats	2001	897	104-113	Brain Res
Zachar, G.; Wagner, Z.; Tabi, T.; Balint, E.; Szoko, E.; Csillag, A.	Differential changes of extracellular aspartate and glutamate in the striatum of domestic chicken evoked by high potassium or distress: an in vivo microdialysis study	2012	37	1730-1737	Neurochem Res
Zangen, A.; Hyodo, K.	Transcranial magnetic stimulation induces increases in extracellular levels of dopamine and glutamate in the nucleus accumbens	2002	13	2401-2405	Neuroreport
Zappettini, S.; Mura, E.; Grilli, M.; Preda, S.; Salamone, A.; Olivero, G.; Govoni, S.; Marchi, M.	Different presynaptic nicotinic receptor subtypes modulate in vivo and in vitro the release of glycine in the rat hippocampus	2011	59	729-738	Neurochem Int
Zayara, A. E.; McIver, G.; Valdivia, P. N.; Lominac, K. D.; McCreary, A. C.; Szumlinski, K. K.	Blockade of nucleus accumbens 5-HT2A and 5-HT2C receptors prevents the expression of cocaine-induced behavioral and neurochemical sensitization in rats	2011	213	321-335	Psychopharmacology(Berl)
Zeng, L. H.; Ouyang, Y.; Gazit, V.; Cirrito, J. R.; Jansen, L. A.; Ess, K. C.; Yamada, K. A.; Wozniak, D. F.; Holtzman, D. M.; Gutmann, D. H.; Wong, M.	Abnormal glutamate homeostasis and impaired synaptic plasticity and learning in a mouse model of tuberous sclerosis complex	2007	28	184-196	Neurobiol Dis
Zeng, X.; Zhang, Y.; Zhang, S.; Zheng, X.	A microdialysis study of effects of gastrodin on neurochemical changes in the ischemic/reperfused rat cerebral hippocampus	2007	30	801-804	Bio Pharm Bull
Zestos, A. G.; Mikelman, S. R.; Kennedy, R. T.; Gnegy, M. E.	PKCbeta Inhibitors Attenuate Amphetamine-Stimulated Dopamine Efflux	2016	7	757-766	ASC Chem Neurosci
Zhang, B.; Chu, J.; Zhang, J.; Ma, Y.	Change of extracellular glutamate and gamma-aminobutyric acid in substantia nigra and globus pallidus during electrical stimulation of subthalamic nucleus in epileptic rats	2008	86	208-215	Stereotact Funct Neurosurg

Glutamate					
**Authors**	**Title**	**Year**	**Vol.**	**Pages**	**Journal**
Zhang, C. Y.; Du, G. Y.; Wang, W.; Ye, Z. G.; Wang, D. Q.; Sun, X. F.; Zhao, D. Z.	[Effects of tianma gouteng fang on transmitter amino acids in the hippocampus extracellular liquids in freely moving rats subjected to brain ischemia]	2004	29	1061-1065	Zhongguo Zhong Yao Za Zhi
Zhang, D.; Zhang, J.; Ma, W.; Chen, D.; Han, H.; Shu, H.; Liu, G.	Analysis of trace amino acid neurotransmitters in hypothalamus of rats after exhausting exercise using microdialysis	2001	758	277-282	J Chromatogr B Biomed Sci Appl
Zhang, F. F.; Wan, Q.; Li, C. X.; Wang, X. L.; Zhu, Z. Q.; Xian, Y. Z.; Jin, L. T.; Yamamoto, K.	Simultaneous assay of glucose, lactate, L-glutamate and hypoxanthine levels in a rat striatum using enzyme electrodes based on neutral red-doped silica nanoparticles	2004	380	637-642	Anal Bioanal Chem
Zhang, F.; Wu, Y.; Jia, J.; Hu, Y. S.	Pre-ischemic treadmill training induces tolerance to brain ischemia: Involvement of glutamate and ERK1/2	2010	15	5246-5257	Molecules
Zhang, G.; Cinalli, D.; Cohen, S. J.; Knapp, K. D.; Rios, L. M.; Martinez-Hernandez, J.; Lujan, R.; Stackman, R. W., Jr.	Examination of the hippocampal contribution to serotonin 5-HT2A receptor-mediated facilitation of object memory in C57BL/6J mice	2016	109	332-340	Neuropharmacology
Zhang, J.; Benveniste, H.; Klitzman, B.; Piantadosi, C. A.	Nitric oxide synthase inhibition and extracellular glutamate concentration after cerebral ischemia/reperfusion	1995	26	289-304	Stroke
Zhang, J.; Benveniste, H.; Piantadosi, C. A.	Inhibition of nitric oxide synthase increases extracellular cerebral glutamate concentration after global ischemia	1993	157	179-182	Neurosci Lett
Zhang, J.; Niu, X.	Changes of monoamines, purines and amino acids in rat striatum as measured by intercerebral microdialysis during ischemia/reperfusion	1994	9	225-229	Chin Med Sci J
Zhang, M.; Ning, G.; Shou, C.; Lu, Y.; Hong, D.; Zheng, X.	Inhibitory effect of jujuboside A on glutamate-mediated excitatory signal pathway in hippocampus	2003	69	692-695	Planta Med
Zhang, N.; Liu, P.; He, X.	[Effect of single-used borneol and combining it with diazepam on content of neurotransmitter in corpus striatum of rats]	2011	36	3180-3183	Zhongguo Zhong Yao Za Zhi
Zhang, N.; Liu, P.; He, X.	Effect of single-use versus combined-use moschus and diazepam on expression of amino acid neurotransmitters in the rat corpus striatum	2012	7	182-186	Neural Regen Res
Zhang, N.; Liu, P.; He, X.	Effect of borneol, moschus, storax, and acorus tatarinowii on expression levels of four amino acid neurotransmitters in the rat corpus striatum	2012	7	440-444	Neural Regen Res
Zhang, S.; Takeda, Y.; Hagioka, S.; Goto, K.; Morita, K.	The close relationship between decreases in extracellular GABA concentrations and increases in the incidence of hyperbaric	2004	58	91-95	Acta Med Okayama

Glutamate					
**Authors**	**Title**	**Year**	**Vol.**	**Pages**	**Journal**
	oxygen-induced electrical discharge				
Zhang, S.; Takeda, Y.; Hagioka, S.; Takata, K.; Aoe, H.; Nakatsuka, H.; Yokoyama, M.; Morita, K.	Measurement of GABA and glutamate in vivo levels with high sensitivity and frequency	2005	14	61-66	Brain Res Brain Protoc
Zhang, T.; Feng, Y.; Rockhold, R. W.; Ho, I. K.	Naloxone-precipitated morphine withdrawal increases pontine glutamate levels in the rat	1994	55	PI25-PI31	Life Sci
Zhang, W. Q.; Hudson, P. M.; Sobotka, T. J.; Hong, J. S.; Tilson, H. A.	Extracellular concentrations of amino acid transmitters in ventral hippocampus during and after the development of kindling	1991	540	315-318	Brain Res
Zhang, W. Q.; Mundy, W. R.; Thai, L.; Hudson, P. M.; Gallagher, M.; Tilson, H. A.; Hong, J. S.	Decreased glutamate release correlates with elevated dynorphin content in the hippocampus of aged rats with spatial learning deficits	1991	1	391-397	Hippocampus
Zhang, W. Q.; Rogers, B. C.; Tandon, P.; Hudson, P. M.; Sobotka, T. J.; Hong, J. S.; Tilson, H. A.	Systemic administration of kainic acid increases GABA levels in perfusate from the hippocampus of rats in vivo	1990	11	593-600	Neurotoxicology
Zhang, W.; Wei, L.; Yu, W.; Cui, X.; Liu, X.; Wang, Q.; Wang, S.	Effect of Jian-Pi-Zhi-Dong Decoction on striatal glutamate and gamma-aminobutyric acid levels detected using microdialysis in a rat model of Tourette syndrome	2016	12	1233-1242	Neuropsychiatr Dis Treat
Zhang, X. J.; Chen, H. L.; Li, Z.; Zhang, H. Q.; Xu, H. X.; Sung, J. J.; Bian, Z. X.	Analgesic effect of paeoniflorin in rats with neonatal maternal separation-induced visceral hyperalgesia is mediated through adenosine A(1) receptor by inhibiting the extracellular signal-regulated protein kinase (ERK) pathway	2009	94	88-97	Pharmacol Biochem Behav
Zhang, X.; Rauch, A.; Lee, H.; Xiao, H.; Rainer, G.; Logothetis, N. K.	Capillary hydrophilic interaction chromatography/mass spectrometry for simultaneous determination of multiple neurotransmitters in primate cerebral cortex	2007	21	3621-3628	Rapid Commun Mass Spectrom
Zhang, X.; Shi, M.; Bjoras, M.; Wang, W.; Zhang, G.; Han, J.; Liu, Z.; Zhang, Y.; Wang, B.; Chen, J.; Zhu, Y.; Xiong, L.; Zhao, G.	Ginsenoside Rd promotes glutamate clearance by up-regulating glial glutamate transporter GLT-1 via PI3K/AKT and ERK1/2 pathways	2013	4	152	Front Pharmacol
Zhang, Y.; Zhang, H.; Feustel, P. J.; Kimelberg, H. K.	DCPIB, a specific inhibitor of volume regulated anion channels (VRACs), reduces infarct size in MCAo and the release of glutamate in the ischemic cortical penumbra	2008	210	514-520	Exp Neurol
Zhao, H.; Asai, S.; Kanematsu, K.; Kunimatsu, T.; Kohno, T.; Ishikawa, K.	Real-time monitoring of the effects of normothermia and hypothermia on extracellular glutamate re-uptake in the rat	1997	8	2389-2393	Neuroreport

Glutamate					
Authors	Title	Year	Vol.	Pages	Journal
	following global brain ischemia				
Zhao, P.; Cheng, J.	Effects of electroacupuncture on extracellular contents of amino acid neurotransmitters in rat striatum following transient focal cerebral ischemia	1997	22	119-126	Acupunct Electrother Res
Zhao, W. H.; Huang, H. L.; Huang, Y.; Xu, X.; Mo, L. D.	Experimental study on the correlation between the cerebral energy metabolism and cerebral blood flow changes in rats with subarachnoid hemorrhage-induced delayed cerebral vasospasm. [Chinese]	2007	7	146-151	Zhongguo Xian Dai Shen Jing Ji Bing Za Zhi
Zhao, X. D.; Zhou, X. P.; Liu, H. H.; Li, B. M.; Hu, X. W.; Li, F. Q.; You, B. M.	[Changes of amino acids neurotransmitters in striatum of hemi-parkinsonian rhesus monkey after high frequency stimulation of subthalamic nucleus]	2007	45	1682-1684	Zhonghua Wai Ke Za Zhi
Zheng, L.; Wu, X.; Dong, X.; Ding, X.; Song, C.	Effects of Chronic Alcohol Exposure on the Modulation of Ischemia-Induced Glutamate Release via Cannabinoid Receptors in the Dorsal Hippocampus	2015	39	1908-1916	Alcohol Clin Exp Res
Zhong, C.; Zhao, X.; Van, K. C.; Bzdega, T.; Smyth, A.; Zhou, J.; Kozikowski, A. P.; Jiang, J.; Connor, W. T.; Berman, R. F.; Neale, J. H.; Lyeth, B. G.	NAAG peptidase inhibitor increases dialysate NAAG and reduces glutamate, aspartate and GABA levels in the dorsal hippocampus following fluid percussion injury in the rat	2006	97	1015-1025	J Neurochem
Zhong, M.; Chen, H.; Jiang, Y.; Tu, P. F.; Liu, C. L.; Zhang, W. X.; Ma, J. Y.; Ding, H.	Effects of echinacoside on striatal extracellular levels of amino acid neurotransmitter in cerebral ischemia rats. [Chinese]	2012	28	361-365	Zhongguo yao li xue tong bao
Zhou, F.; Braddock, J. F.; Hu, Y.; Zhu, X.; Castellani, R. J.; Smith, M. A.; Drew, K. L.	Microbial origin of glutamate, hibernation and tissue trauma: an in vivo microdialysis study	2002	119	121-128	J Neurosci Methods
Zhou, J.; Lunte, S. M.	Direct determination of amino acids by capillary electrophoresis/electrochemistry using a copper microelectrode and zwitterionic buffers	1995	16	498-503	Electrophoresis
Zhou, Q. M.; Lu, Y. L.; Hu, G.; Zeng, Y. M.; Tu, W. F.	Effect of aquaporin-4 deficiency on intravenous anaesthetic induced hypnotic effects in mice	2013	65	569-576	Sheng Li Xue Bao
Zhou, S. Y.; Zuo, H.; Stobaugh, J. F.; Lunte, C. E.; Lunte, S. M.	Continuous in vivo monitoring of amino acid neurotransmitters by microdialysis sampling with on-line derivatization and capillary electrophoresis separation	1995	67	594-599	Anal Chem
Zhou, W.; Fu, L. W.; Guo, Z. L.; Longhurst, J. C.	Role of glutamate in the rostral ventrolateral medulla in acupuncture-related modulation of visceral reflex	2007	292	H1868-H1875	Am J Physiol Heart Circ Physiol

Glutamate					
**Authors**	**Title**	**Year**	**Vol.**	**Pages**	**Journal**
	sympathoexcitation				
Zhou, W.; Fu, L. W.; Tjen, A. Looi S. C.; Guo, Z. L.; Longhurst, J. C.	Role of glutamate in a visceral sympathoexcitatory reflex in rostral ventrolateral medulla of cats	2006	291	H1309-H1318	Am J Physiol Heart Circ Physiol
Zhu, D. N.; Moriguchi, A.; Mikami, H.; Higaki, J.; Ogihara, T.	Central amino acids mediate cardiovascular response to angiotensin II in the rat	1998	45	189-197	Brain Res Bull
Zhu, G.; Okada, M.; Murakami, T.; Kamata, A.; Kawata, Y.; Wada, K.; Kaneko, S.	Dysfunction of M-channel enhances propagation of neuronal excitability in rat hippocampus monitored by multielectrode dish and microdialysis systems	2000	294	53-57	Neurosci Lett
Zhu, G.; Okada, M.; Uchiyama, D.; Ohkubo, T.; Yoshida, S.; Kaneko, S.	Hyperactivity of endoplasmic reticulum associated exocytosis mechanism contributes to acute phencyclidine intoxication	2004	95	214-227	J Pharmacol Sci
Zhu, G.; Okada, M.; Yoshida, S.; Hirose, S.; Kaneko, S.	Determination of exocytosis mechanisms of DOPA in rat striatum using in vivo microdialysis	2004	367	241-245	Neurosci Lett
Zhu, M. Y.; Wang, W. P.; Huang, J.; Feng, Y. Z.; Regunathan, S.; Bissette, G.	Repeated immobilization stress alters rat hippocampal and prefrontal cortical morphology in parallel with endogenous agmatine and arginine decarboxylase levels	2008	53	346-354	Neurochem Int
Zhu, X.; Ye, G.; Wang, Z.; Luo, J.; Hao, X.	Sub-anesthetic doses of ketamine exert antidepressant-like effects and upregulate the expression of glutamate transporters in the hippocampus of rats	2016	639	132-137	Neurosci Lett
Zielinska, M.; Ruszkiewicz, J.; Hilgier, W.; Fresko, I.; Albrecht, J.	Hyperammonemia increases the expression and activity of the glutamine/arginine transporter y+ LAT2 in rat cerebral cortex: implications for the nitric oxide/cGMP pathway	2011	58	190-195	Neurochem Int
Zietlow, R.; Sinclair, S. R.; Schwiening, C. J.; Dunnett, S. B.; Fawcett, J. W.	The release of excitatory amino acids, dopamine, and potassium following transplantation of embryonic mesencephalic dopaminergic grafts to the rat striatum, and their effects on dopaminergic neuronal survival in vitro	2002	11	637-652	Cell Transplantation
Zilkha, E.; Obrenovitch, T. P.; Koshy, A.; Kusakabe, H.; Bennetto, H. P.	Extracellular glutamate: on-line monitoring using microdialysis coupled to enzyme-amperometric analysis	1995	60	1-9	J Neurosci Methods
Zimmer, E. R.; Parent, M. J.; Leuzy, A.; Aliaga, A.; Aliaga, A.; Moquin, L.; Schirrmacher, E. S.; Soucy, J. P.; Skelin, I.; Gratton, A.; Gauthier, S.; Rosa-Neto, P.	Imaging in vivo glutamate fluctuations with [(11)C]ABP688: a GLT-1 challenge with ceftriaxone	2015	35	1169-1174	J Cereb Blood Flow Metab
Zoremba, N.; Homola, A.; Rossaint, R.; Sykova, E.	Brain metabolism and extracellular space diffusion parameters during and after transient global hypoxia in the rat cortex	2007	203	34-41	Exp Neurol

Glutamate					
**Authors**	**Title**	**Year**	**Vol.**	**Pages**	**Journal**
Zoremba, N.; Schnoor, J.; Berens, M.; Kuhlen, R.; Rossaint, R.	Brain metabolism during a decrease in cerebral perfusion pressure caused by an elevated intracranial pressure in the porcine neocortex	2007	105	744-750	Anesth Analg
Zuiderwijk, M.; Ghijsen, W. E.	Monitoring amino acid neurotransmitter release in the brain by in vivo microdialysis	1997	72	239-50	Methods Mol Biol
Zuiderwijk, M.; Veenstra, E.; Lopes da Silva, F. H.; Ghijsen, W. E.	Effects of uptake carrier blockers SK & F 89976-A and L-trans-PDC on in vivo release of amino acids in rat hippocampus	1996	307	275-282	Eur J Pharmacol
Zuo, D. Y.; Zhang, Y. H.; Cao, Y.; Wu, C. F.; Tanaka, M.; Wu, Y. L.	Effect of acute and chronic MK-801 administration on extracellular glutamate and ascorbic acid release in the prefrontal cortex of freely moving mice on line with open-field behavior	2006	78	2172-2178	Life Sci
Zweckberger, K.; Hackenberg, K.; Jung, C. S.; Hertle, D. N.; Kiening, K. L.; Unterberg, A. W.; Sakowitz, O. W.	Cerebral metabolism after early decompression craniotomy following controlled cortical impact injury in rats	2011	33	875-880	Neurol Res
Zweckberger, K.; Simunovic, F.; Kiening, K. L.; Unterberg, A. W.; Sakowitz, O. W.	Effects of lisuride hydrogen maleate on pericontusional tissue metabolism, brain edema formation, and contusion volume development after experimental traumatic brain injury in rats	2011	499	189-193	Neurosci Lett

Appendix 6: Studies describing baseline dialysate concentrations for glycine

Glycine					
**Authors**	**Title**	**Year**	**Vol**	**Pages**	**Journal**
Abraham, I.; Juhasz, G.; Kekesi, K. A.; Kovacs, K. J.	Effect of intrahippocampal dexamethasone on the levels of amino acid transmitters and neuronal excitability	1996	733	56-63	Brain Res
Abraham, I.; Juhasz, G.; Kekesi, K. A.; Kovacs, K. J.	Corticosterone peak is responsible for stress-induced elevation of glutamate in the hippocampus	1998	2	171-181	Stress
Acworth, I. N.; Yu, J.; Ryan, E.; Gariepy, K. C.; Gamache, P.; Hull, K.; Maher, T.	Simultaneous measurement of monoamine, amino acid, and drug levels, using high performance liquid chromatography and coulometric array technology: Application to in vivo microdialysis perfusate analysis	1994	17	685-705	J Liq Chromatogr
Adachi, N.; Chen, J.; Liu, K.; Nagaro, T.; Arai, T.	Metyrapone alleviates ischemic neuronal damage in the gerbil hippocampus	1999	373	147-152	Eur J Pharmacol
Adachi, N.; Chen, J.; Nakanishi, K.; Arai, T.	Pre-ischaemic administration of procaine suppresses ischaemic glutamate release and reduces neuronal damage in the gerbil hippocampus	1999	83	472-474	Br J Anaesth
Adachi, N.; Seyfried, F. J.; Arai, T.	Blockade of central histaminergic H2 receptors aggravates ischemic neuronal damage in gerbil hippocampus	2001	29	1189-1194	Crit Care Med
Almaguer-Melian, W.; Cruz-Aguado, R.; Riva Cde, L.; Kendrick, K. M.; Frey, J. U.; Bergado, J.	Effect of LTP-reinforcing paradigms on neurotransmitter release in the dentate gyrus of young and aged rats	2005	327	877-883	Biochem Biophys Res Commun
Amakawa, K.; Adachi, N.; Liu, K.; Ikemune, K.; Fujitani, T.; Arai, T.	Effects of pre- and postischemic administration of thiopental on transmitter amino acid release and histologic outcome in gerbils	1996	85	1422-1430	Anesthesiology
Amantea, D.; Fratto, V.; Maida, S.; Rotiroti, D.; Ragusa, S.; Nappi, G.; Bagetta, G.; Corasaniti, M. T.	Prevention of Glutamate Accumulation and Upregulation of Phospho-Akt may Account for Neuroprotection Afforded by Bergamot Essential Oil against Brain Injury Induced by Focal Cerebral Ischemia in Rat	2009	85	389-405	Int Rev Neurobiol
Anderson, J. J.; DiMicco, J. A.	The use of microdialysis for studying the regional effects of pharmacological manipulation on extracellular levels of amino acids--some	1992	51	623-630	Life Sci

Glycine					
**Authors**	**Title**	**Year**	**Vol**	**Pages**	**Journal**
	methodological aspects				
Anderson, J. J.; DiMicco, J. A.	Effect of local inhibition of gamma-aminobutyric acid uptake in the dorsomedial hypothalamus on extracellular levels of gamma-aminobutyric acid and on stress-induced tachycardia: a study using microdialysis	1990	255	1399-1407	J Pharmacol Exp Ther
Anderzhanova, E.; Saransaari, P.; Oja, S. S.	Neuroprotective mechanisms of taurine in vivo	2006	583	377-387	Adv Exp Med Biol
Andine, P.; Orwar, O.; Jacobson, I.; Sandberg, M.; Hagberg, H.	Changes in extracellular amino acids and spontaneous neuronal activity during ischemia and extended reflow in the CA1 of the rat hippocampus	1991	57	222-229	J Neurochem
Baker, A. J.; Zornow, M. H.; Grafe, M. R.; Scheller, M. S.; Skilling, S. R.; Smullin, D. H.; Larson, A. A.	Hypothermia prevents ischemia-induced increases in hippocampal glycine concentrations in rabbits	1991	22	666-673	Stroke
Baker, A. J.; Zornow, M. H.; Scheller, M. S.; Yaksh, T. L.; Skilling, S. R.; Smullin, D. H.; Larson, A. A.; Kuczenski, R.	Changes in extracellular concentrations of glutamate, aspartate, glycine, dopamine, serotonin, and dopamine metabolites after transient global ischemia in the rabbit brain	1991	57	1370-1379	J Neurochem
Behrens, P. F.; Langemann, H.; Strohschein, R.; Draeger, J.; Hennig, J.	Extracellular glutamate and other metabolites in and around RG2 rat glioma: an intracerebral microdialysis study	2000	47	11-22	J Neurooncol
Beitz, A. J.	Relationship of glutamate and aspartate to the periaqueductal gray-raphe magnus projection: analysis using immunocytochemistry and microdialysis	1990	38	1755-1765	J Histochem Cytochem
Bereiter, D. A.; Shen, S.; Benetti, A. P.	Sex differences in amino acid release from rostral trigeminal subnucleus caudalis after acute injury to the TMJ region	2002	98	89-99	Pain
Bergquist, F.; Ludwig, M.; Dutia, M. B.	Neuroscience I: Commissural GABA release during vestibular compensation in the rat	2009	37	S28	Amino Acids
Bergquist, F.; Ludwig, M.; Dutia, M. B.	Role of the commissural inhibitory system in vestibular compensation in the rat	2008	586	4441-4452	J Physiol
Bergquist, J.; Vona, M. J.; Stiller, C. O.; Connor, W. T.; Falkenberg, T.; Ekman, R.	Capillary electrophoresis with laser-induced fluorescence detection: a sensitive method for monitoring extracellular concentrations of amino acids	1996	65	33-42	J Neurosci Methods

Glycine					
**Authors**	**Title**	**Year**	**Vol**	**Pages**	**Journal**
	in the periaqueductal grey matter				
Bledsoe Jr, S. C.; Nagase, S.; Miller, J. M.; Altschuler, R. A.	Deafness-induced plasticity in the mature central auditory system	1995	7	225-229	NeuroReport
Boatell, M. L.; Bendahan, G.; Mahy, N.	Time-related cortical amino acid changes after basal forebrain lesion: a microdialysis study	1995	64	285-291	J Neurochem
Bosman, D. K.; Deutz, N. E.; Maas, M. A.; van Eijk, H. M.; Smit, J. J.; de Haan, J. G.; Chamuleau, R. A.	Amino acid release from cerebral cortex in experimental acute liver failure, studied by in vivo cerebral cortex microdialysis	1992	59	591-599	J Neurochem
Bottcher, T.; Ren, H.; Goiny, M.; Gerber, J.; Lykkesfeldt, J.; Kuhn, U.; Lotz, M.; Bunkowski, S.; Werner, C.; Schau, I.; Spreer, A.; Christen, S.; Nau, R.	Clindamycin is neuroprotective in experimental Streptococcus pneumoniae meningitis compared with ceftriaxone	2004	91	1450-1460	J Neurochem
Bowser, M. T.; Kennedy, R. T.	In vivo monitoring of amine neurotransmitters using microdialysis with on-line capillary electrophoresis	2001	22	3668-3676	Electrophoresis
Boyd, B. W.; Witowski, S. R.; Kennedy, R. T.	Trace-level amino acid analysis by capillary liquid chromatography and application to in vivo microdialysis sampling with 10-s temporal resolution	2000	72	865-871	Anal Chem
Brennan, P. A.; Kendrick, K. M.; Keverne, E. B.	Neurotransmitter release in the accessory olfactory bulb during and after the formation of an olfactory memory in mice	1995	69	1075-1086	Neuroscience
Brodie, M. E.; Opacka-Juffry, J.; Peterson, D. W.; Brown, A. W.	Neurochemical changes in hippocampal and caudate dialysates associated with early trimethyltin neurotoxicity in rats	1990	11	35-46	NeuroToxicology
Bruhn, T.; Cobo, M.; Berg, M.; Diemer, N. H.	Limbic seizure-induced changes in extracellular amino acid levels in the hippocampal formation: a microdialysis study of freely moving rats	1992	86	455-461	Acta Neurol Scand
Brust, P.; Christensen, T.; Diemer, N. H.	Decrease of extracellular taurine in the rat dorsal hippocampus after central nervous administration of vasopressin	1992	58	1427-1431	J Neurochem
Buratta, S.; Hamberger, A.; Ryberg, H.; Nystrom, B.; Sandberg, M.; Mozzi, R.	Effect of serine and ethanolamine administration on phospholipid-related compounds and neurotransmitter amino acids in the rabbit hippocampus	1998	71	2145-2150	J Neurochem

Glycine					
**Authors**	**Title**	**Year**	**Vol**	**Pages**	**Journal**
Cabrera-Pastor, A.; Taoro-Gonzalez, L.; Felipo, V.	Hyperammonemia alters glycinergic neurotransmission and modulation of the glutamate-nitric oxide-cGMP pathway by extracellular glycine in cerebellum in vivo	2016	137	539-548	J Neurochem
Camacho, A.; Montiel, T.; Massieu, L.	The anion channel blocker, 4,4'-dinitrostilbene-2,2'-disulfonic acid prevents neuronal death and excitatory amino acid release during glycolysis inhibition in the hippocampus in vivo	2006	142	1005-1017	Neuroscience
Cantor, S. L.; Zornow, M. H.; Miller, L. P.; Yaksh, T. L.	The effect of cyclohexyladenosine on the periischemic increases of hippocampal glutamate and glycine in the rabbit	1992	59	1884-1892	J Neurochem
Cassel, G. E.; Fosbraey, P.	Measurement of the oxime HI-6 after peripheral administration in tandem with neurotransmitter levels in striatal dialysates: effects of soman intoxication	1996	35	159-166	J Pharmacol Toxicol Methods
Chapman, M. A.; See, R. E.	Differential effects of unique profile antipsychotic drugs on extracellular amino acids in the ventral pallidum and globus pallidus of rats	1996	277	1586-1594	J Pharmacol Exp Ther
Chau, P.; Soderpalm, B.; Ericson, M.	The mGluR5 antagonist MPEP elevates accumbal dopamine and glycine levels; interaction with strychnine-sensitive glycine receptors	2011	16	591-599	Addict Biol
Chen, J.; Adachi, N.; Tsubota, S.; Nagaro, T.; Arai, T.	Dexamethasone augments ischemia-induced extracellular accumulation of glutamate in gerbil hippocampus	1998	347	67-70	Eur J Pharmacol
Chen, J.; Xu, W.; Jiang, H.	17 beta-estradiol protects neurons from ischemic damage and attenuates accumulation of extracellular excitatory amino acids	2001	92	1520-1523	Anesth Analg
Chen, R.; Deng, Y.; Yao, J.; Kamal, G. M.; Wang, J.; Xu, F.	Assessment of amino acid neurotransmitters in rat brain microdialysis samples by high-performance liquid chromatography with coulometric detection	2015	38	1439-1447	J Liq Chromatogr Relat Technol
Choi, K. T.; Chung, J. K.; Kwak, C. S.; Kim, H. K.	Effect of hypocapnia on extracellular glutamate and glycine concentrations during the periischemic period in rabbit hippocampus	1995	765	86-97	Ann N Y Acad Sci
Choi, K. T.; Chung, J. K.; Kwak, C. S.; Kim, H. K.	Effect of hypocapnia on extracellular glutamate and glycine concentrations during peri-ischemic period in	1994	9	394-401	J Korean Med Sci

Glycine					
**Authors**	**Title**	**Year**	**Vol**	**Pages**	**Journal**
	the rabbit hippocampus				
Choi, K. T.; Illievich, U. M.; Zornow, M. H.; Scheller, M. S.; Strnat, M. A.	Effect of hyperglycemia on peri-ischemic neurotransmitter levels in the rabbit hippocampus	1994	642	104-110	Brain Res
Choi, Y. H.; Chang, N.; Fletcher, P. J.; Anderson, G. H.	Dietary protein content affects the profiles of extracellular amino acids in the medial preoptic area of freely moving rats	2000	66	1105-1118	Life Sci
Choi, Y. H.; Fletcher, P. J.; Anderson, G. H.	Extracellular amino acid profiles in the paraventricular nucleus of the rat hypothalamus are influenced by diet composition	2001	892	320-328	Brain Res
Choi, Y. H.; Fletcher, P. J.; Wong, C. C.; Anderson, G. H.	Measurement of blood-brain barrier permeability of rats with alpha-aminoisobutyric acid during microdialysis: possible application to behavioral studies	1999	67	587-598	Physiol Behav
Cioffi, C. L.; Liu, S.; Wolf, M. A.; Guzzo, P. R.; Sadalapure, K.; Parthasarathy, V.; Loong, D. T.; Maeng, J. H.; Carulli, E.; Fang, X.; Karunakaran, K.; Matta, L.; Choo, S. H.; Panduga, S.; Buckle, R. N.; Davis, R. N.; Sakwa, S. A.; Gupta, P.; Sargent, B. J.; Moore, N. A.; Luche, M. M.; Carr, G. J.; Khmelnitsky, Y. L.; Ismail, J.; Chung, M.; Bai, M.; Leong, W. Y.; Sachdev, N.; Swaminathan, S.; Mhyre, A. J.	Synthesis and Biological Evaluation of N-((1-(4-(Sulfonyl)piperazin-1-yl)cycloalkyl)methyl)benzamide Inhibitors of Glycine Transporter-1	2016	59	8473-8494	J Med Chem
Cobo, M.; Bruhn, T.; Berg, M.; Diemer, N. H.	Phenylsuccinate reduces KCL-induced release of GABA evidence for the participation of the ketodicarboxylate carrier in the biosynthesis of transmitter-GABA	1993	5	377-388	Amino Acids
Currie, P. J.; Chang, N.; Luo, S.; Anderson, G. H.	Microdialysis as a tool to measure dietary and regional effects on the complete profile of extracellular amino acids in the hypothalamus of rats	1995	57	1911-1923	Life Sci
Czell, D.; Efe, T.; Preuss, M.; Schofer, M. D.; Becker, R.	Influence of Intraventricular Application of Baclofen on Arterial Blood Pressure and Neurotransmitter	2011	/	1-6	Neurochem Res

Glycine					
**Authors**	**Title**	**Year**	**Vol**	**Pages**	**Journal**
	Concentrations in the Hypothalamic Paraventricular Nucleus of Rats				
Dahchour, A.; Hoffman, A.; Deitrich, R.; de Witte, P.	Effects of ethanol on extracellular amino acid levels in high-and low-alcohol sensitive rats: a microdialysis study	2000	35	548-553	Alcohol Alcohol
Daisley, J. N.; Gruss, M.; Rose, S. P.; Braun, K.	Passive avoidance training and recall are associated with increased glutamate levels in the intermediate medial hyperstriatum ventrale of the day-old chick	1998	6	53-61	Neural Plast
David, C. N.; Frias, E. S.; Szu, J. I.; Vieira, P. A.; Hubbard, J. A.; Lovelace, J.; Michael, M.; Worth, D.; McGovern, K. E.; Ethell, I. M.; Stanley, B. G.; Korzus, E.; Fiacco, T. A.; Binder, D. K.; Wilson, E. H.	GLT-1-Dependent Disruption of CNS Glutamate Homeostasis and Neuronal Function by the Protozoan Parasite Toxoplasma gondii	2016	12	e1005643	PLoS Pathog
Dawson, L. A.; Organ, A. J.; Winter, P.; Lacroix, L. P.; Shilliam, C. S.; Heidbreder, C.; Shah, A. J.	Rapid high-throughput assay for the measurement of amino acids from microdialysates and brain tissue using monolithic C18-bonded reversed-phase columns	2004	807	235-241	J Chromatogr B Analyt Technol Biomed Life Sci
de Novellis, V.; Marabese, I.; Uliano, R.; Palazzo, E.; Scafuro, A.; sca Rossi, F.; Maione, S.	Type I and II metabotropic glutamate receptors modulate periaqueductal grey glycine release: interaction between mGlu2/3 and A1 adenosine receptors	2002	43	1061-1069	Neuropharmacology
Delgado, J. M.; DeFeudis, F. V.; Roth, R. H.; Ryugo, D. K.; Mitruka, B. M.	Dialytrode for long term intracerebral perfusion in awake monkeys	1972	198	9-21	Arch Int Pharmacodyn Ther
Delgado, J. M.; Lerma, J.; Martin del Rio, R.; Solis, J. M.	Dialytrode technology and local profiles of amino acids in the awake cat brain	1984	42	1218-1228	J Neurochem
Devall, A. J.; Blake, R.; Langman, N.; Smith, C. G.; Richards, D. A.; Whitehead, K. J.	Monolithic column-based reversed-phase liquid chromatography separation for amino acid assay in microdialysates and cerebral spinal fluid	2007	848	323-328	J Chromatogr B Analyt Technol Biomed Life Sci
Doheny, M. H.; Nagaki, S.; Patsalos, P. N.	A microdialysis study of glycinamide, glycine and other amino acid neurotransmitters in rat frontal cortex and hippocampus after the administration of milacemide, a glycine pro-drug	1996	354	157-163	Naunyn Schmiedebergs Arch Pharmacol

Glycine					
**Authors**	**Title**	**Year**	**Vol**	**Pages**	**Journal**
Dolgodilina, E.; Imobersteg, S.; Laczko, E.; Welt, T.; Verrey, F.; Makrides, V.	Brain interstitial fluid glutamine homeostasis is controlled by blood-brain barrier SLC7A5/LAT1 amino acid transporter	2016	36	1929-1941	J Cereb Blood Flow Metab
Doretto, M. C.; Burger, R. L.; Mishra, P. K.; Garcia-Cairasco, N.; Dailey, J. W.; Jobe, P. C.	A microdialysis study of amino acid concentrations in the extracellular fluid of the substantia nigra of freely behaving GEPR-9s: relationship to seizure predisposition	1994	17	157-165	Epilepsy Res
During, M. J.; Craig, J. S.; Hernandez, T. D.; Anderson, G. M.; Gallager, D. W.	Effect of amygdala kindling on the in vivo release of GABA and 5-HT in the dorsal raphe nucleus in freely moving rats	1992	584	36-44	Brain Res
Engel, M.; Snikeris, P.; Jenner, A.; Karl, T.; Huang, X. F.; Frank, E.	Neuregulin 1 prevents phencyclidine-induced behavioral impairments and disruptions to GABAergic signaling in mice	2015	18	1-11	Int J Neuropsychopharmacol
Fabre-Nys, C.; Blache, D.; Hinton, M. R.; Goode, J. A.; Kendrick, K. M.	Microdialysis measurement of neurochemical changes in the mediobasal hypothalamus of ovariectomized ewes during oestrus	1994	649	282-296	Brain Res
Fabricius, M.; Jensen, L. H.; Lauritzen, M.	Microdialysis of interstitial amino acids during spreading depression and anoxic depolarization in rat neocortex	1993	612	61-69	Brain Res
Fedele, E.; Foster, A. C.	An evaluation of the role of extracellular amino acids in the delayed neurodegeneration induced by quinolinic acid in the rat striatum	1993	52	911-917	Neuroscience
Fordahl, S. C.; Anderson, J. G.; Cooney, P. T.; Weaver, T. L.; Colyer, C. L.; Erikson, K. M.	Manganese exposure inhibits the clearance of extracellular GABA and influences taurine homeostasis in the striatum of developing rats	2010	31	639-646	NeuroToxicology
Foster, S. B.; Tang, H.; Miller, K. E.; Dryhurst, G.	Increased extracellular glutamate evoked by 1-methyl-4-phenylpyridinium [MPP(+)] in the rat striatum is not essential for dopaminergic neurotoxicity and is not derived from released glutathione	2005	7	251-263	Neurotox Res
Fraser, M.; Bennet, L.; Van Zijl, P. L.; Mocatta, T. J.; Williams, C. E.; Gluckman, P. D.; Winterbourn, C. C.; Gunn, A. J.	Extracellular amino acids and lipid peroxidation products in periventricular white matter during and after cerebral ischemia in preterm fetal sheep	2008	105	2214-2223	J Neurochem

Glycine					
**Authors**	**Title**	**Year**	**Vol**	**Pages**	**Journal**
Fujihira, T.; Kanematsu, S.; Umino, A.; Yamamoto, N.; Nishikawa, T.	Selective increase in the extracellular D-serine contents by D-cycloserine in the rat medial frontal cortex	2007	51	233-236	Neurochem Int
Fujikawa, D. G.; Kim, J. S.; Daniels, A. H.; Alcaraz, A. F.; Sohn, T. B.	In vivo elevation of extracellular potassium in the rat amygdala increases extracellular glutamate and aspartate and damages neurons	1996	74	695-706	Neuroscience
Fujitani, T.; Adachi, N.; Miyazaki, H.; Liu, K.; Nakamura, Y.; Kataoka, K.; Arai, T.	Lidocaine protects hippocampal neurons against ischemic damage by preventing increase of extracellular excitatory amino acids: a microdialysis study in Mongolian gerbils	1994	179	91-94	Neurosci Lett
Ganguly, P. K.; Maddaford, T. G.; Edel, A. L.; O, K.; Smeda, J. S.; Pierce, G. N.	Increased homocysteine-induced release of excitatory amino acids in the striatum of spontaneously hypertensive stroke-prone rats	2008	1226	192-198	Brain Res
Ge, J.; Long, S. K.; Kilpatrick, I. C.	Preferential blockade of cholecystokinin-8S-induced increases in aspartate and glutamate levels by the CCK(B) receptor antagonist, L-365,260, in rat brain	1998	345	163-170	Eur J Pharmacol
Gemba, T.; Matsunaga, K.; Ueda, M.	Changes in extracellular concentration of amino acids in the hippocampus during cerebral ischemia in stroke-prone SHR, stroke-resistant SHR and normotensive rats	1992	135	184-188	Neurosci Lett
Gemba, T.; Ninomiya, M.; Matsunaga, K.; Ueda, M.	Effects of a novel calcium antagonist, S-(+)-methyl-4,7-dihydro-3-isobutyl-6- methyl-4-(3-nitrophenyl)thieno[2,3-b]pyridine-5-carboxylate (S-312-d), on ischemic amino acid release and neuronal injury in stroke-prone spontaneously hypertensive rats	1993	265	463-467	J Pharmacol Exp Ther
Globus, M. Y.; Busto, R.; Martinez, E.; Valdes, I.; Dietrich, W. D.; Ginsberg, M. D.	Comparative effect of transient global ischemia on extracellular levels of glutamate, glycine, and gamma-aminobutyric acid in vulnerable and nonvulnerable brain regions in the rat	1991	57	470-478	J Neurochem
Globus, M. Y.; Ginsberg, M. D.; Busto, R.	Excitotoxic index--a biochemical marker of selective vulnerability	1991	127	39-42	Neurosci Lett
Gobert, A.; Rivet, J. M.; Billiras, R.; Parsons, F.; Millan, M. J.	Simultaneous quantification of D- vs. L-serine, taurine, kynurenate, phosphoethanolamine and diverse amino	2011	202	143-157	J Neurosci Methods

Glycine					
Authors	Title	Year	Vol	Pages	Journal
	acids in frontocortical dialysates of freely-moving rats: differential modulation by N-methyl-D-aspartate (NMDA) and other pharmacological agents				
Goldsmith, J. D.; Kujawa, S. G.; McLaren, J. D.; Bledsoe Jr, S. C.	In vivo release of neuroactive amino acids from the inferior colliculus of the guinea pig using brain microdialysis	1995	83	80-88	Hear Res
Gordon, K. E.; Simpson, J.; Statman, D.; Silverstein, F. S.	Effects of perinatal stroke on striatal amino acid efflux in rats studied with in vivo microdialysis	1991	22	928-932	Stroke
Grabb, M. C.; Sciotti, V. M.; Gidday, J. M.; Cohen, S. A.; van Wylen, D. G.	Neurochemical and morphological responses to acutely and chronically implanted brain microdialysis probes	1998	82	25-34	J Neurosci Methods
Graham, S. H.; Chen, J.; Lan, J.; Leach, M. J.; Simon, R. P.	Neuroprotective effects of a use-dependent blocker of voltage-dependent sodium channels, BW619C89, in rat middle cerebral artery occlusion	1994	269	854-859	J Pharmacol Exp Ther
Graham, S. H.; Shimizu, H.; Newman, A.; Weinstein, P.; Faden, A. I.	Opioid receptor antagonist nalmefene stereospecifically inhibits glutamate release during global cerebral ischemia	1993	632	346-350	Brain Res
Graham, S. H.; Shiraishi, K.; Panter, S. S.; Simon, R. P.; Faden, A. I.	Changes in extracellular amino acid neurotransmitters produced by focal cerebral ischemia	1990	110	124-130	Neurosci Lett
Grobin, A. C.; Deutch, A. Y.	Dopaminergic regulation of extracellular gamma-aminobutyric acid levels in the prefrontal cortex of the rat	1998	285	350-357	J Pharmacol Exp Ther
Guerra-Romero, L.; Tureen, J. H.; Fournier, M. A.; Makrides, V.; Tauber, M. G.	Amino acids in cerebrospinal and brain interstitial fluid in experimental pneumococcal meningitis	1993	33	510-513	Pediatr Res
Hamann, M.; Sohr, R.; Morgenstern, R.; Richter, A.	Extracellular amino acid levels in the striatum of the dt(sz) mutant, a model of paroxysmal dystonia	2008	157	188-195	Neuroscience
Harsing, L. G., Jr.; Matyus, P.	Mechanisms of glycine release, which build up synaptic and extrasynaptic glycine levels: the role of synaptic and non-synaptic glycine transporters	2013	93	110-119	Brain Res Bull
Hasegawa, T.; Azum, S.; Inoue, S.	Amino acid release from the rat oral pontine reticular nucleus across the sleep-wakefulness cycle	2000	47	87-93	J Med Dent Sci
Hashimoto, A.; Oka, T.; Nishikawa,	Extracellular concentration of endogenous free D-	1995	66	635-643	Neuroscience

Glycine					
**Authors**	**Title**	**Year**	**Vol**	**Pages**	**Journal**
T.	serine in the rat brain as revealed by in vivo microdialysis				
Hashimoto, K.; Fujita, Y.; Ishima, T.; Chaki, S.; Iyo, M.	Phencyclidine-induced cognitive deficits in mice are improved by subsequent subchronic administration of the glycine transporter-1 inhibitor NFPS and D-serine	2008	18	414-421	Eur Neuropsychopharmacol
Hassel, B.; Dahlberg, D.; Mariussen, E.; Goverud, I. L.; Antal, E. A.; Tonjum, T.; Maehlen, J.	Brain infection with Staphylococcus aureus leads to high extracellular levels of glutamate, aspartate, gamma-aminobutyric acid, and zinc	2014	92	1792-1800	J Neurosci Res
Hathway, G. J.; Emson, P. C.; Humphrey, P. P.; Kendrick, K. M.	Somatostatin potently stimulates in vivo striatal dopamine and gamma-aminobutyric acid release by a glutamate-dependent action	1998	70	1740-1749	J Neurochem
Hazell, A. S.; Butterworth, R. F.; Hakim, A. M.	Cerebral vulnerability is associated with selective increase in extracellular glutamate concentration in experimental thiamine deficiency	1993	61	1155-1158	J Neurochem
Hehre, D. A.; Devia, C. J.; Bancalari, E.; Suguihara, C.	Brainstem amino acid neurotransmitters and ventilatory response to hypoxia in piglets	2008	63	46-50	Pediatr Res
Heron, A.; Lasbennes, F.; Seylaz, J.	Adenosine modulation of amino acid release in rat hippocampus during ischemia and veratridine depolarization	1993	608	27-32	Brain Res
Hillered, L.; Hallstrom, A.; Segersvard, S.; Persson, L.; Ungerstedt, U.	Dynamics of extracellular metabolites in the striatum after middle cerebral artery occlusion in the rat monitored by intracerebral microdialysis	1989	9	607-616	J Cereb Blood Flow Metab
Hoop, B.; Beagle, J. L.; Maher, T. J.; Kazemi, H.	Brainstem amino acid neurotransmitters and hypoxic ventilatory response	1999	118	117-129	Respir Physiol
Horio, M.; Kohno, M.; Fujita, Y.; Ishima, T.; Inoue, R.; Mori, H.; Hashimoto, K.	Levels of D-serine in the brain and peripheral organs of serine racemase (Srr) knock-out mice	2011	59	853-859	Neurochem Int
Horn, T.; Bauce, L.; Landgraf, R.; Pittman, Q. J.	Microdialysis with high NaCl causes central release of amino acids and dopamine	1995	64	1632-1644	J Neurochem
Huang, F. P.; Zhou, L. F.; Yang, G. Y.	Effects of mild hypothermia on the release of regional glutamate and glycine during extended transient focal cerebral ischemia in rats	1998	23	991-996	Neurochem Res
Huang, M.; Panos, J. J.; Kwon, S.;	Comparative effect of lurasidone and blonanserin on	2014	128	938-949	J Neurochem

Glycine					
**Authors**	**Title**	**Year**	**Vol**	**Pages**	**Journal**
Oyamada, Y.; Rajagopal, L.; Meltzer, H. Y.	cortical glutamate, dopamine, and acetylcholine efflux: role of relative serotonin (5-HT)2A and DA D2 antagonism and 5-HT1A partial agonism				
Huo, T.; Zhang, Y.; Li, W.; Yang, H.; Jiang, H.; Sun, G.	Effect of realgar on extracellular amino acid neurotransmitters in hippocampal CA1 region determined by online microdialysis-dansyl chloride derivatization-high-performance liquid chromatography and fluorescence detection	2014	28	1254-1262	Biomed Chromatogr
Hwa, L. S.; Nathanson, A. J.; Shimamoto, A.; Tayeh, J. K.; Wilens, A. R.; Holly, E. N.; Newman, E. L.; DeBold, J. F.; Miczek, K. A.	Aggression and increased glutamate in the mPFC during withdrawal from intermittent alcohol in outbred mice	2015	232	2889-2902	Psychopharmacology (Berl)
Hwabejire, J. O.; Jin, G.; Imam, A. M.; Duggan, M.; Sillesen, M.; Deperalta, D.; Jepsen, C. H.; Lu, J.; Li, Y.; DeMoya, M. A.; Velmahos, G. C.; Socrate, S.; Alam, H. B.	Pharmacological modulation of mitochondrial dysfunction-induced cerebral metabolic derangement and excitotoxicity in a porcine model of traumatic brain injury and hemorrhagic shock	2013	179	322	J Surg Res Conference: 8th Annual Academic Surgical Congress of the Association for Academic Surgery, AAS and the Society of University Surgeons, SUS. New Orleans, LA United States. Conference Start
Hylland, P.; Nilsson, G. E.	Extracellular levels of amino acid neurotransmitters during anoxia and forced energy deficiency in crucian carp brain	1999	823	49-58	Brain Res
Hylland, P.; Nilsson, G. E.; Johansson, D.	Anoxic brain failure in an ectothermic vertebrate: release of amino acids and K+ in rainbow trout thalamus	1995	269	R1077-R1084	Am J Physiol
Ichord, R. N.; Johnston, M. V.; Traystman, R. J.	MK801 decreases glutamate release and oxidative metabolism during hypoglycemic coma in piglets	2001	128	139-148	Brain Res Dev Brain Res
Iino, T.; Katsura, M.; Kuriyama, K.	Protective effect of vinconate on ischemia-induced neuronal damage in the rat hippocampus	1992	224	117-124	Eur J Pharmacol

Glycine					
**Authors**	**Title**	**Year**	**Vol**	**Pages**	**Journal**
Illievich, U. M.; Zornow, M. H.; Choi, K. T.; Strnat, M. A.; Scheller, M. S.	Effects of hypothermia or anesthetics on hippocampal glutamate and glycine concentrations after repeated transient global cerebral ischemia	1994	80	177-186	Anesthesiology
Irimia, C.; Buczynski, M.; Laredo, S.; Alvaros, N.; Parsons, L.	Dysregulated NMDA NR1 signaling in the infralimbic cortex contributes to increased impulsivity during protracted alcohol abstinence	2015	40	S600-S601	Neuropsychopharmacology
Ishiwata, S.; Umino, A.; Balu, D. T.; Coyle, J. T.; Nishikawa, T.	Neuronal serine racemase regulates extracellular D-serine levels in the adult mouse hippocampus	2015	122	1099-1103	J Neural Transm (Vienna)
Itoh, T.; Saito, T.; Fujimura, M.; Watanabe, S.; Saito, K.	Restraint stress-induced changes in endogenous zinc release from the rat hippocampus	1993	618	318-322	Brain Res
Jacobson, I.; Hagberg, H.; Sandberg, M.; Hamberger, A.	Ouabain-induced changes in extracellular aspartate, glutamate and GABA levels in the rabbit olfactory bulb in vivo	1986	64	211-215	Neurosci Lett
Jacobsson, S. O.; Cassel, G. E.; Karlsson, B. M.; Sellstrom, A.; Persson, S. A.	Release of dopamine, GABA and EAA in rats during intrastriatal perfusion with kainic acid, NMDA and soman: a comparative microdialysis study	1997	71	756-765	Arch Toxicol
Jacobsson, S. O.; Sellstrom, A.; Persson, S. A.; Cassel, G. E.	Correlation between cortical EEG and striatal microdialysis in soman-intoxicated rats	1997	231	155-158	Neurosci Lett
Jimenez-Diaz, L.; Gruart, A.; Minano, F. J.; Delgado-Garcia, J. M.	An experimental study of posterior interpositus involvement in the genesis and control of conditioned eyelid responses	2002	978	106-118	Ann N Y Acad Sci
Juhasz, G.; Kekesi, K. A.; Nyitrai, G.; Dobolyi, A.; Krogsgaard-Larsen, P.; Schousboe, A.	Differential effects of nipecotic acid and 4,5,6,7-tetrahydroisoxazolo[4,5-c]pyridin-3-ol on extracellular gamma-aminobutyrate levels in rat thalamus	1997	331	139-144	Eur J Pharmacol
Kahn, R. A.; Panah, M.; Kiffel, S.; Weinberger, J.	Modulation of ischemic excitatory neurotransmitter and gamma-aminobutyric acid release during global temporary cerebral ischemia by local nitric oxide synthase inhibition	1997	84	1004-1010	Anesth Analg
Kahn, R. A.; Panah, M.; Weinberger, J.	Modulation of ischemic excitatory neurotransmitter and gamma-aminobutyric acid release during global temporary cerebral ischemia by selective neuronal nitric oxide synthase inhibition	1997	84	997-1003	Anesth Analg
Kapoor, V.; Minson, J.; Chalmers, J.	Ventral medulla stimulation increases blood pressure	1992	3	55-58	Neuroreport

Glycine					
**Authors**	**Title**	**Year**	**Vol**	**Pages**	**Journal**
	and spinal cord amino acid release				
Kapoor, V.; Nakahara, D.; Blood, R. J.; Chalmers, J. P.	Preferential release of neuroactive amino acids from the ventrolateral medulla of the rat in vivo as measured by microdialysis	1990	37	187-191	Neuroscience
Kasper, J. M.; Booth, R. G.; Peris, J.	Serotonin-2C receptor agonists decrease potassium-stimulated GABA release in the nucleus accumbens	2015	69	78-85	Synapse
Katoh, H.; Sima, K.; Nawashiro, H.; Wada, K.; Chigasaki, H.	The effect of MK-801 on extracellular neuroactive amino acids in hippocampus after closed head injury followed by hypoxia in rats	1997	758	153-621	Brain Res
Kaura, S.; Bradford, H. F.; Young, A. M.; Croucher, M. J.; Hughes, P. D.	Effect of amygdaloid kindling on the content and release of amino acids from the amygdaloid complex: in vivo and in vitro studies	1995	65	1240-1249	J Neurochem
Kekesi, K. A.; Dobolyi, A.; Salfay, O.; Nyitrai, G.; Juhasz, G.	Slow wave sleep is accompanied by release of certain amino acids in the thalamus of cats	1997	8	1183-1186	Neuroreport
Kekesi, K. A.; Szilagyi, N.; Nyitrai, G.; Dobolyi, A.; Skuban, N.; Kardos, J.	Persistent depolarization and Glu uptake inhibition operate distinct osmoregulatory mechanisms in the mammalian brain	2000	37	171-178	Neurochem Int
Kennedy, R. T.; Thompson, J. E.; Vickroy, T. W.	In vivo monitoring of amino acids by direct sampling of brain extracellular fluid at ultralow flow rates and capillary electrophoresis	2002	114	39-49	J Neurosci Methods
Khandelwal, P.; Beyer, C. E.; Lin, Q.; McGonigle, P.; Schechter, L. E.; Bach, A. C., 2nd	Nanoprobe NMR spectroscopy and in vivo microdialysis: new analytical methods to study brain neurochemistry	2004	133	181-189	J Neurosci Methods
Khandelwal, P.; Beyer, C. E.; Lin, Q.; Schechter, L. E.; Bach, A. C., 2nd	Studying rat brain neurochemistry using nanoprobe NMR spectroscopy: a metabonomics approach	2004	76	4123-4127	Anal Chem
Kim, H. K.; Zornow, M. H.; Strnat, M. A.; Maze, M.	Dexmedetomidine does not attenuate increases in excitatory amino acids after transient global ischemia in the rabbit	1996	8	230-236	J Neurosurg Anesthesiol
Kitaichi, Y.; Hashimoto, R.; Inoue, T.; Abekawa, T.; Kakuta, A.; Hattori, S.; Koyama, T.	Abnormalities in extracellular glycine and glutamate levels in the striatum of sandy mice	2013	25	215-220	Acta Neuropsychiatr
Klinker, C. C.; Bowser, M. T.	4-fluoro-7-nitro-2,1,3-benzoxadiazole as a fluorogenic labeling reagent for the in vivo analysis of amino acid	2007	79	8747-8754	Anal Chem

Glycine					
**Authors**	**Title**	**Year**	**Vol**	**Pages**	**Journal**
	neurotransmitters using online microdialysis-capillary electrophoresis				
Kodama, T.; Lai, Y. Y.; Siegel, J. M.	Changes in inhibitory amino acid release linked to pontine-induced atonia: an in vivo microdialysis study	2003	23	1548-1554	J Neurosci
Koizumi, H.; Fujisawa, H.; Ito, H.; Maekawa, T.; Di, X.; Bullock, R.	Effects of mild hypothermia on cerebral blood flow-independent changes in cortical extracellular levels of amino acids following contusion trauma in the rat	1997	747	304-312	Brain Res
Kuntz, A.; Clement, H. W.; Lehnert, W.; van Calker, D.; Hennighausen, K.; Gerlach, M.; Schulz, E.	Effects of secretin on extracellular amino acid concentrations in rat hippocampus	2004	111	931-939	J Neural Transm (Vienna)
Lallement, G.; Carpentier, P.; Collet, A.; Pernot-Marino, I.; Baubichon, D.; Blanchet, G.	Effects of soman-induced seizures on different extracellular amino acid levels and on glutamate uptake in rat hippocampus	1991	563	234-240	Brain Res
Langlais, P. J.; Shu Xing, Zhang	Extracellular glutamate is increased in thalamus during thiamine deficiency-induced lesions and is blocked by MK-801	1993	61	2175-2182	J Neurochem
Largo, C.; Cuevas, P.; Somjen, G. G.; Martin del Rio, R.; Herreras, O.	The effect of depressing glial function in rat brain in situ on ion homeostasis, synaptic transmission, and neuron survival	1996	16	1219-1229	J Neurosci
Lazarewicz, J. W.; Salinska, E.; Puka, M.	Glycine enhances extracellular 45Ca2+ response to NMDA application investigated with microdialysis of rabbit hippocampus in vivo	1992	52	83-91	Acta Neurobiol Exp (Wars)
Lee, T. F.; Jantzie, L. L.; Todd, K. G.; Cheung, P. Y.	Postresuscitation N-acetylcysteine treatment reduces cerebral hydrogen peroxide in the hypoxic piglet brain	2008	34	190-197	Intensive Care Med
Leonetti, M.; Desvignes, C.; Bougault, I.; Souilhac, J.; Oury-Donat, F.; Steinberg, R.	2-Chloro-N-[(S)-phenyl [(2S)-piperidin-2-yl] methyl]-3-trifluoromethyl benzamide, monohydrochloride, an inhibitor of the glycine transporter type 1, increases evoked-dopamine release in the rat nucleus accumbens in vivo via an enhanced glutamatergic neurotransmission	2006	137	555-564	Neuroscience
Li, H.; Li, C.; Yan, Z. Y.; Yang, J.; Chen, H.	Simultaneous monitoring multiple neurotransmitters and neuromodulators during cerebral ischemia/reperfusion in rats by microdialysis and	2010	189	162-168	J Neurosci Methods

Glycine					
**Authors**	**Title**	**Year**	**Vol**	**Pages**	**Journal**
	capillary electrophoresis				
Li, H.; Yan, Z. Y.	Analysis of amino acid neurotransmitters in hypothalamus of rats during cerebral ischemia-reperfusion by microdialysis and capillary electrophoresis	2010	24	1185-1192	Biomed Chromatogr
Li, P. A.; He, Q. P.; Miyashita, H.; Howllet, W.; Siesjo, B. K.; Shuaib, A.	Hypothermia ameliorates ischemic brain damage and suppresses the release of extracellular amino acids in both normo- and hyperglycemic subjects	1999	158	242-253	Exp Neurol
Li, T. F.; Xue, W.; Li, M.; Shi, A. X.; Li, K. X.	Establishment of an LC-MS/MS method to determine content of six amino acids in rat brain dialysate. [Chinese]	2015	24	1659-1664	Zhongguo xin yao za zhi
Li, X.; Li, J.; Peng, X. Q.; Spiller, K.; Gardner, E. L.; Xi, Z. X.	Metabotropic glutamate receptor 7 modulates the rewarding effects of cocaine in rats: involvement of a ventral pallidal GABAergic mechanism	2009	34	1783-1796	Neuropsychopharmacology
Li, Z. P.; Zhang, X. Y.; Lu, X.; Zhong, M. K.; Ji, Y. H.	Dynamic release of amino acid transmitters induced by valproate in PTZ-kindled epileptic rat hippocampus	2004	44	263-270	Neurochem Int
Li, Z.; Boules, M.; Williams, K.; Peris, J.; Richelson, E.	The novel neurotensin analog NT69L blocks phencyclidine (PCP)-induced increases in locomotor activity and PCP-induced increases in monoamine and amino acids levels in the medial prefrontal cortex	2010	1311	28-36	Brain Res
Li, Z.; Boules, M.; Williams, K.; Peris, J.; Richelson, E.	The novel neurotensin analog NT69L blocks phencyclidine-induced increases in locomotor activity and increases in monoamine and amino acids levels in the mPFC	2009	1	71S-72S	Biol Psychiatry
Li, Z.; Zharikova, A.; Bastian, J.; Esperon, L.; Hebert, N.; Mathes, C.; Rowland, N. E.; Peris, J.	High temporal resolution of amino acid levels in rat nucleus accumbens during operant ethanol self-administration: involvement of elevated glycine in anticipation	2008	106	170-181	J Neurochem
Li, Z.; Zharikova, A.; Vaughan, C. H.; Bastian, J.; Zandy, S.; Esperon, L.; Axman, E.; Rowland, N. E.; Peris, J.	Intermittent high-dose ethanol exposures increase motivation for operant ethanol self-administration: possible neurochemical mechanism	2010	1310	142-153	Brain Res
Lido, H. H.; Marston, H.; Ericson, M.; Soderpalm, B.	The glycine reuptake inhibitor Org24598 and acamprosate reduce ethanol intake in the rat; tolerance	2012	17	897-907	Addict Biol

Glycine					
Authors	Title	Year	Vol	Pages	Journal
	development to acamprosate but not to Org24598				
Lido, H. H.; Stomberg, R.; Fagerberg, A.; Ericson, M.; Soderpalm, B.	The glycine reuptake inhibitor org 25935 interacts with basal and ethanol-induced dopamine release in rat nucleus accumbens	2009	33	1151-1157	Alcohol Clin Exp Res
Lin, B.; Globus, M. Y.; Dietrich, W. D.; Busto, R.; Martinez, E.; Ginsberg, M. D.	Differing neurochemical and morphological sequelae of global ischemia: comparison of single- and multiple-insult paradigms	1992	59	2213-2223	J Neurochem
Liu, H.; Zhang, G.; Bie, X.; Liu, M.; Yang, J.; Wan, H.; Zhang, Y.	[Effect of polydatin on dynamic changes of excitatory amino acids in cerebrospinal fluid of cerebral hemorrhage rats]	2010	35	3038-3042	Zhongguo Zhong Yao Za Zhi
Liu, P.; He, X. R.; Zhou, W. B.; Shen, R. R.; Feng, F.	[Research for dependablity of administration of platycodi radix in Tianwang Buxinwan decoction with change of brain inhibitive neurotransmitte in rats by microdialysis]	2008	33	2830-2833	Zhongguo Zhong Yao Za Zhi
Liu, Z.; Zhang, L.; He, Q.; Liu, X.; Chukwunweike Ikechukwu, O.; Tong, L.; Guo, L.; Yang, H.; Zhang, Q.; Zhao, H.; Gu, X.	Effect of Baicalin-loaded PEGylated cationic solid lipid nanoparticles modified by OX26 antibody on regulating the levels of baicalin and amino acids during cerebral ischemia-reperfusion in rats	2015	489	131-138	Int J Pharm
Lo, E. H.; Bosque-Hamilton, P.; Meng, W.	Inhibition of poly(ADP-ribose) polymerase: reduction of ischemic injury and attenuation of N-methyl-D-aspartate-induced neurotransmitter dysregulation	1998	29	830-836	Stroke
Luna-Munguia, H.; Meneses, A.; Pena-Ortega, F.; Gaona, A.; Rocha, L.	Effects of hippocampal high-frequency electrical stimulation in memory formation and their association with amino acid tissue content and release in normal rats	2012	22	98-105	Hippocampus
Maciejak, P.; Szyndler, J.; Turzynska, D.; Sobolewska, A.; Plaznik, A.	Kynurenic acid: A new effector of valproate action?	2011	63	1569-1573	Pharmacol Rep
Maekawa, M.; Ohnishi, T.; Hashimoto, K.; Watanabe, A.; Iwayama, Y.; Ohba, H.; Hattori, E.; Yamada, K.; Yoshikawa, T.	Analysis of strain-dependent prepulse inhibition points to a role for Shmt1 (SHMT1) in mice and in schizophrenia	2010	115	1374-1385	J Neurochem
Maione, S.; Marabese, I.; Rossi, F.;	Effects of persistent nociception on periaqueductal gray	2000	97	311-316	Neuroscience

Glycine					
Authors	Title	Year	Vol	Pages	Journal
Berrino, L.; Palazzo, E.; Trabace, L.	glycine release				
Marchi, M.; Zappettini, S.; Mura, E.; Grilli, M.; Preda, S.; Salamone, A.; Pittaluga, A.; Govoni, S.	Different presynaptic nicotinic receptor subtypes modulate in vivo and in vitro the release of glycine in the rat hippocampus	2011	82(8)	1032-1033	Biochem Pharmacol
Marsala, M.; Sorkin, L. S.; Yaksh, T. L.	Transient spinal ischemia in rat: characterization of spinal cord blood flow, extracellular amino acid release, and concurrent histopathological damage	1994	14	604-614	J Cereb Blood Flow Metab
Mason, P. A.; Escarciga, R.; Doyle, J. M.; Romano, W. F.; Berger, R. E.; Donnellan, J. P.	Amino acid concentrations in hypothalamic and caudate nuclei during microwave-induced thermal stress: analysis by microdialysis	1997	18	277-283	Bioelectromagnetics
Massieu, L.; Gomez-Roman, N.; Montiel, T.	In vivo potentiation of glutamate-mediated neuronal damage after chronic administration of the glycolysis inhibitor iodoacetate	2000	165	257-267	Exp Neurol
Massieu, L.; Morales-Villagran, A.; Tapia, R.	Accumulation of extracellular glutamate by inhibition of its uptake is not sufficient for inducing neuronal damage: an in vivo microdialysis study	1995	64	2262-2272	J Neurochem
Massieu, L.; Tapia, R.	Glutamate uptake impairment and neuronal damage in young and aged rats in vivo	1997	69	1151-1160	J Neurochem
Matsui, T.; Sekiguchi, M.; Hashimoto, A.; Tomita, U.; Nishikawa, T.; Wada, K.	Functional comparison of D-serine and glycine in rodents: the effect on cloned NMDA receptors and the extracellular concentration	1995	65	454-458	J Neurochem
Matsumoto, K.; Graf, R.; Rosner, G.; Taguchi, J.; Heiss, W. D.	Elevation of neuroactive substances in the cortex of cats during prolonged focal ischemia	1993	13	586-594	J Cereb Blood Flow Metab
Matsumoto, M.; Scheller, M. S.; Zornow, M. H.; Strnat, M. A.	Effect of S-emopamil, nimodipine, and mild hypothermia on hippocampal glutamate concentrations after repeated cerebral ischemia in rabbits	1993	24	1228-1234	Stroke
Matsumoto, M.; Zornow, M. H.; Scheller, M. S.; Strnat, M. A.	AMPA and NMDA receptor antagonists do not decrease hippocampal glutamate concentrations during transient global ischemia	1992	77	764-771	Anesthesiology
McKenzie, J. A.; Watson, C. J.; Rostand, R. D.; German, I.; Witowski, S. R.; Kennedy, R. T.	Automated capillary liquid chromatography for simultaneous determination of neuroactive amines and amino acids	2002	962	105-115	J Chromatogr A
Medina-Ceja, L.; Morales-Villagran,	Action of 4-aminopyridine on extracellular amino acids	2000	53	255-262	Brain Res Bull

Glycine					
**Authors**	**Title**	**Year**	**Vol**	**Pages**	**Journal**
A.; Tapia, R.	in hippocampus and entorhinal cortex: a dual microdialysis and electroencehalographic study in awake rats				
Mena, F. V.; Baab, P. J.; Zielke, C. L.; Zielke, H. R.	In vivo glutamine hydrolysis in the formation of extracellular glutamate in the injured rat brain	2000	60	632-641	J Neurosci Res
Michalak, A.; Rose, C.; Butterworth, J.; Butterworth, R. F.	Neuroactive amino acids and glutamate (NMDA) receptors in frontal cortex of rats with experimental acute liver failure	1996	24	908-913	Hepatology
Mileykovskiy, B. Y.; Kiyashchenko, L. I.; Kodama, T.; Lai, Y. Y.; Siegel, J. M.	Activation of pontine and medullary motor inhibitory regions reduces discharge in neurons located in the locus coeruleus and the anatomical equivalent of the midbrain locomotor region	2000	20	8551-8558	J Neurosci
Millan, M. H.; Chapman, A. G.; Meldrum, B. S.	Contrasting effects of D- and L-(E)-4-(3-phosphono-2-propenyl)piperazine-2-carboxylic acid as anticonvulsants and as inhibitors of potassium-evoked increases in hippocampal extracellular glutamate and aspartate levels in freely moving rats	1994	62	217-222	J Neurochem
Millan, M. H.; Chapman, A. G.; Meldrum, B. S.	Extracellular amino acid levels in hippocampus during pilocarpine-induced seizures	1993	14	139-148	Epilepsy Res
Miyashita, K.; Abe, H.; Nakajima, T.; Ishikawa, A.; Nishiura-Suzuki, M.; Naritomi, H.; Tanaka, R.; Sawada, T.	Glutamate release in the gerbil hippocampus after middle cerebral artery occlusion	1994	5	945-948	Neuroreport
Miyashita, K.; Nakajima, T.; Ishikawa, A.; Miyatake, T.	An adenosine uptake blocker, propentofylline, reduces glutamate release in gerbil hippocampus following transient forebrain ischemia	1992	17	147-150	Neurochem Res
Molchanova, S.; Oja, S. S.; Saransaari, P.	Characteristics of basal taurine release in the rat striatum measured by microdialysis	2004	27	261-268	Amino Acids
Montiel, T.; Camacho, A.; Estrada-Sanchez, A. M.; Massieu, L.	Differential effects of the substrate inhibitor l-trans-pyrrolidine-2,4-dicarboxylate (PDC) and the non-substrate inhibitor DL-threo-beta-benzyloxyaspartate (DL-TBOA) of glutamate transporters on neuronal damage and extracellular amino acid levels in rat brain in vivo	2005	133	667-678	Neuroscience

Glycine					
**Authors**	**Title**	**Year**	**Vol**	**Pages**	**Journal**
Morales-Villagran, A.; Tapia, R.	Preferential stimulation of glutamate release by 4-aminopyridine in rat striatum in vivo	1996	28	35-40	Neurochem Int
Morrone, L. A.; Rombola, L.; Pelle, C.; Corasaniti, M. T.; Zappettini, S.; Paudice, P.; Bonanno, G.; Bagetta, G.	The essential oil of bergamot enhances the levels of amino acid neurotransmitters in the hippocampus of rat: implication of monoterpene hydrocarbons	2007	55	255-262	Pharmacol Res
Muere, C.; Neumueller, S.; Miller, J.; Olesiak, S.; Hodges, M. R.; Pan, L.; Forster, H. V.	Atropine microdialysis within or near the pre-Botzinger Complex increases breathing frequency more during wakefulness than during NREM sleep	2013	114	694-704	J Appl Physiol (1985)
Nagy, K.; Marko, B.; Zsilla, G.; Matyus, P.; Pallagi, K.; Szabo, G.; Juranyi, Z.; Barkoczy, J.; Levay, G.; Harsing Jr, L. G.	Alterations in brain extracellular dopamine and glycine levels following combined administration of the glycine transporter type-1 inhibitor Org-24461 and risperidone	2010	35	2096-2106	Neurochem Res
Nakashima, K.; Todd, M. M.	Effects of hypothermia, pentobarbital, and isoflurane on postdepolarization amino acid release during complete global cerebral ischemia	1996	85	161-168	Anesthesiology
Nakashima, K.; Todd, M. M.	Effects of hypothermia on the rate of excitatory amino acid release after ischemic depolarization	1996	27	913-918	Stroke
Nakata, N.; Kato, H.; Kogure, K.	Ischemic tolerance and extracellular amino acid concentrations in gerbil hippocampus measured by intracerebral microdialysis	1994	35	247-251	Brain Res Bull
Nakata, N.; Kato, H.; Kogure, K.; DeGraba, T. J.	Effects of repeated cerebral ischemia on extracellular amino acid concentrations measured with intracerebral microdialysis in the gerbil hippocampus	1993	24	458-464	Stroke
Nencioni, A. L. A.; Barreto, S. A.; Lebrun, I.; Florio, J. C.; Lourenco, G. A.; Dorce, V. A. C.	Neurotransmitter evaluation in the hippocampus of rats after intracerebral injection of TsTX scorpion toxin	2009	15	236-254	J Venom Anim Toxins Incl Trop Dis
Nencioni, A. L.; Lourenco, G. A.; Lebrun, I.; Florio, J. C.; Dorce, V. A.	Central effects of Tityus serrulatus and Tityus bahiensis scorpion venoms after intraperitoneal injection in rats	2009	463	234-238	Neurosci Lett
Nilsson, G. E.; Lutz, P. L.	Release of inhibitory neurotransmitters in response to anoxia in turtle brain	1991	261	R32-R37	Am J Physiol
Nilsson, P.; Hillered, L.; Ponten, U.; Ungerstedt, U.	Changes in cortical extracellular levels of energy-related metabolites and amino acids following	1990	10	631-637	J Cereb Blood Flow Metab

Glycine					
Authors	Title	Year	Vol	Pages	Journal
	concussive brain injury in rats				
Nilsson, P.; Ronne-Engstrom, E.; Flink, R.; Ungerstedt, U.; Carlson, H.; Hillered, L.	Epileptic seizure activity in the acute phase following cortical impact trauma in rat	1994	637	227-232	Brain Res
Nitz, D.; Siegel, J.	GABA release in the dorsal raphe nucleus: role in the control of REM sleep	1997	273	R451-R455	Am J Physiol
Nyitrai, G.; Kekesi, K. A.; Szilagyi, N.; Papp, A.; Juhasz, G.; Kardos, J.	Neurotoxicity of lindane and picrotoxin: neurochemical and electrophysiological correlates in the rat hippocampus in vivo	2002	27	139-145	Neurochem Res
Nyitrai, G.; Kovacs, I.; Szarics, E.; Skuban, N.; Juhasz, G.; Kardos, J.	Role of intracellular Ca(2+) stores shaping normal activity in brain	1999	57	906-915	J Neurosci Res
Obrenovitch, T. P.; Urenjak, J.; Richards, D. A.; Ueda, Y.; Curzon, G.; Symon, L.	Extracellular neuroactive amino acids in the rat striatum during ischaemia: comparison between penumbral conditions and ischaemia with sustained anoxic depolarisation	1993	61	178-186	J Neurochem
Oda, M.; Kure, S.; Sugawara, T.; Yamaguchi, S.; Kojima, K.; Shinka, T.; Sato, K.; Narisawa, A.; Aoki, Y.; Matsubara, Y.; Omae, T.; Mizoi, K.; Kinouchi, H.	Direct correlation between ischemic injury and extracellular glycine concentration in mice with genetically altered activities of the glycine cleavage multienzyme system	2007	38	2157-2164	Stroke
Okuda, N.; Kohara, K.; Mikami, H.; Moriguchi, A.; Yamada, K.; Higaki, J.; Ogihara, T.	Effect of propranolol on central neurotransmitter release in Wistar rats analysed by brain microdialysis	1999	26	220-224	Clin Exp Pharmacol Physiol
Ooboshi, H.; Ibayashi, S.; Takano, K.; Sadoshima, S.; Kondo, A.; Uchimura, H.; Fujishima, M.	Hypothermia inhibits ischemia-induced efflux of amino acids and neuronal damage in the hippocampus of aged rats	2000	884	23-30	Brain Research
Ooboshi, H.; Sadoshima, S.; Yao, H.; Ibayashi, S.; Matsumoto, T.; Uchimura, H.; Fujishima, M.	Ischemia-induced release of amino acids in the hippocampus of aged hypertensive rats	1995	15	227-234	J Cereb Blood Flow Metab
Oreiro-Garcia, M. T.; Vazquez-Illanes, M. D.; Sierra-Paredes, G.; Sierra-Marcuno, G.	Changes in extracellular amino acid concentrations in the rat hippocampus after in vivo actin depolymerization with latrunculin A	2007	50	734-740	Neurochem Int
Oreiro-Garcia, M. T.; Vazquez-	Analysis of neuroactive amino acids from	2005	19	720-724	Biomed Chromatogr

Glycine					
**Authors**	**Title**	**Year**	**Vol**	**Pages**	**Journal**
Illanes, M. D.; Sierra-Paredes, G.; Sierra-Marcuno, G.	microdialysate samples by fluorescence detection using a modification of the 6-aminoquinolyl-N-hydroxysuccinimidyl carbamate method				
Orwar, O.; Li, X.; Andine, P.; Bergstrom, C. M.; Hagberg, H.; Folestad, S.; Sandberg, M.	Increased intra- and extracellular concentrations of gamma-glutamylglutamate and related dipeptides in the ischemic rat striatum: involvement of glutamyl transpeptidase	1994	63	1371-1376	J Neurochem
Osborne, P. G.; Hashimoto, M.	Mammalian cerebral metabolism and amino acid neurotransmission during hibernation	2008	106	1888-1899	J Neurochem
Oshinsky, M. L.; Murphy, A. L.; Hekierski, H., Jr.; Cooper, M.; Simon, B. J.	Noninvasive vagus nerve stimulation as treatment for trigeminal allodynia	2014	155	1037-1042	Pain
Oskarsson, A.; Ljungberg, T.; Stahle, L.	Behavioral and neurochemical effects after combined perinatal treatment of rats with lead and disulfiram	1986	8	591-599	Neurotoxicol Teratol
Ossanai, L. T.; Lourenco, G. A.; Nencioni, A. L.; Lebrun, I.; Yamanouye, N.; Dorce, V. A.	Effects of a toxin isolated from Tityus bahiensis scorpion venom on the hippocampus of rats	2012	91	230-236	Life Sci
Patel, P. M.; Drummond, J. C.; Cole, D. J.; Goskowicz, R. L.	Isoflurane reduces ischemia-induced glutamate release in rats subjected to forebrain ischemia	1995	82	996-1003	Anesthesiology
Patel, P. M.; Goskowicz, R. L.; Drummond, J. C.; Cole, D. J.	Etomidate reduces ischemia-induced glutamate release in the hippocampus in rats subjected to incomplete forebrain ischemia	1995	80	933-939	Anesth Analg
Pena, F.; Tapia, R.	Relationships among seizures, extracellular amino acid changes, and neurodegeneration induced by 4-aminopyridine in rat hippocampus: a microdialysis and electroencephalographic study	1999	72	2006-2014	J Neurochem
Piepponen, T. P.; Skujins, A.	Rapid and sensitive step gradient assays of glutamate, glycine, taurine and gamma-aminobutyric acid by high-performance liquid chromatography-fluorescence detection with o-phthalaldehyde-mercaptoethanol derivatization with an emphasis on microdialysis samples	2001	757	277-283	J Chromatogr B Biomed Sci Appl
Pierard, C.; Lagarde, D.; Barrere, B.;	Effects of a vigilance enhancing drug, modafinil, on rat	1997	25	51-54	Med Sci Res

Glycine					
**Authors**	**Title**	**Year**	**Vol**	**Pages**	**Journal**
Duret, P.; Cordeiro, C.; Guezennec, C. Y.; Peres, M.	brain cortex amino acids: A microdialysis study				
Pierard, C.; Peres, M.; Satabin, P.; Guezennec, C. Y.; Lagarde, D.	Effects of GABA-transaminase inhibition on brain metabolism and amino-acid compartmentation: an in vivo study by 2D 1H-NMR spectroscopy coupled with microdialysis	1999	127	321-327	Exp Brain Res
Qu, Y.; Arckens, L.; Vandenbussche, E.; Geeraerts, S.; Vandesande, F.	Simultaneous determination of total and extracellular concentrations of the amino acid neurotransmitters in cat visual cortex by microbore liquid chromatography and electrochemical detection	1998	798	19-26	J Chromatogr A
Qu, Y.; Li, Y.; Vandenbussche, E.; Vandesande, F.; Arckens, L.	In vivo microdialysis in the visual cortex of awake cat. II: sample analysis by microbore HPLC-electrochemical detection and capillary electrophoresis-laser-induced fluorescence detection	2001	7	45-51	Brain Res Brain Res Protoc
Qureshi, A. I.; Ali, Z.; Suri, M. F.; Shuaib, A.; Baker, G.; Todd, K.; Guterman, L. R.; Hopkins, L. N.	Extracellular glutamate and other amino acids in experimental intracerebral hemorrhage: an in vivo microdialysis study	2003	31	1482-1489	Crit Care Med
Radhakrishnan, R.; Sluka, K. A.	Increased glutamate and decreased glycine release in the rostral ventromedial medulla during induction of a pre-clinical model of chronic widespread muscle pain	2009	457	141-145	Neurosci Lett
Renno, W. M.; Alkhalaf, M.; Mousa, A.; Kanaan, R. A.	A comparative study of excitatory and inhibitory amino acids in three different brainstem nuclei	2008	33	150-159	Neurochem Res
Renno, W. M.; Mullet, M. A.; Williams, F. G.; Beitz, A. J.	Construction of 1 mm microdialysis probe for amino acids dialysis in rats	1998	79	217-228	J Neurosci Methods
Renno, W. M.; Mullett, M. A.; Beitz, A. J.	Systemic morphine reduces GABA release in the lateral but not the medial portion of the midbrain periaqueductal gray of the rat	1992	594	221-232	Brain Res
Richards, D. A.; Morrone, L. A.; Bowery, N. G.	Hippocampal extracellular amino acids and EEG spectral analysis in a genetic rat model of absence epilepsy	2000	39	2433-2441	Neuropharmacology
Rocha, L.; Briones, M.; Ackermann, R. F.; Anton, B.; Maidment, N. T.; Evans, C. J.; Engel, J., Jr.	Pentylenetetrazol-induced kindling: early involvement of excitatory and inhibitory systems	1996	26	105-113	Epilepsy Res

Glycine					
**Authors**	**Title**	**Year**	**Vol**	**Pages**	**Journal**
Ronne Engstrom, E.; Hillered, L.; Flink, R.; Kihlstrom, L.; Lindquist, C.; Nie, J. X.; Olsson, Y.; Silander, H. C.	Extracellular amino acid levels measured with intracerebral microdialysis in the model of posttraumatic epilepsy induced by intracortical iron injection	2001	43	135-144	Epilepsy Res
Rose, M. E.; Huerbin, M. B.; Melick, J.; Marion, D. W.; Palmer, A. M.; Schiding, J. K.; Kochanek, P. M.; Graham, S. H.	Regulation of interstitial excitatory amino acid concentrations after cortical contusion injury	2002	935	40-46	Brain Res
Rozza, A.; Masoero, E.; Favalli, L.; Lanza, E.; Govoni, S.; Rizzo, V.; Montalbetti, L.	Influence of different anaesthetics on extracellular aminoacids in rat brain	2000	101	165-169	J Neurosci Methods
Runnerstam, M.; Bao, F.; Huang, Y.; Shi, J.; Gutierrez, E.; Hamberger, A.; Hansson, H. A.; Viano, D.; Haglid, K.	A new model for diffuse brain injury by rotational acceleration: II. Effects on extracellular glutamate, intracranial pressure, and neuronal apoptosis	2001	18	259-273	J Neurotrauma
Runnerstam, M.; Stephensen, H.; von Essen, C.; Nystrom, B.; Hamberger, A.	Subarachnoid blood infusion versus raised intracranial pressure: effects on the amino acid pattern in the extracellular fluid of the rabbit hippocampus	1999	21	404-408	Neurol Res
Saleh, T. M.; Connell, B. J.; Legge, C.; Cribb, A. E.	Estrogen attenuates neuronal excitability in the insular cortex following middle cerebral artery occlusion	2004	1018	119-129	Brain Res
Saleh, T. M.; Connell, B. J.; McQuaid, T.; Cribb, A. E.	Estrogen-induced neurochemical and electrophysiological changes in the parabrachial nucleus of the male rat	2003	990	58-65	Brain Res
Sanchez-Carbente, M. D. R.; Massieu, L.	Transient inhibition of glutamate uptake in vivo induces neurodegeneration when energy metabolism is impaired	1999	72	129-138	J Neurochem
Saul; skaya, N. B.; Mikhailova, M. O.; Gorbachevskaya, A. I.	Dopamine-dependent inhibition of glycine release in the nucleus accumbens of the rat brain during food consumption	2001	31	317-321	Neurosci Behav Physiol
Saul; skaya, N. B.; Solov; eva, N. A.	Tetrodotoxin-dependent glycine release in the rat nucleus accumbens during correction of feeding behavior	2005	35	815-819	Neurosci Behav Physiol
Semba, J.; Miyoshi, R.; Kanazawa, M.; Kito, S.	Effect of systemic dexamethasone on extracellular amino acids, GABA and acetylcholine release in rat	1995	10	17-21	Funct Neurol

Glycine					
**Authors**	**Title**	**Year**	**Vol**	**Pages**	**Journal**
	hippocampus using in vivo microdialysis				
Semba, J.; Wakuta, M. S.	Regional differences in the effects of glutamate uptake inhibitor L-trans-pyrrolidine-2,4-dicarboxylic acid on extracellular amino acids and dopamine in rat brain: an in vivo microdialysis study	1998	31	399-404	Gen Pharmacol
Shah, A. J.; de Biasi, V.; Taylor, S. G.; Roberts, C.; Hemmati, P.; Munton, R.; West, A.; Routledge, C.; Camilleri, P.	Development of a protocol for the automated analysis of amino acids in brain tissue samples and microdialysates	1999	735	133-140	J Chromatogr B Biomed Sci Appl
Shen, E. Y.; Lai, Y. J.	In vivo microdialysis study of excitatory and inhibitory amino acid levels in the hippocampus following penicillin-induced seizures in mature rats	2002	43	313-318	Acta Paediatr Taiwan
Sheng, W.; Hang, H. W.; Ruan, D. Y.	In vivo microdialysis study of the relationship between lead-induced impairment of learning and neurotransmitter changes in the hippocampus	2005	20	233-340	Environ Toxicol Pharmacol
Shimizu, H.; Graham, S. H.; Chang, L. H.; Mintorovitch, J.; James, T. L.; Faden, A. I.; Weinstein, P. R.	Relationship between extracellular neurotransmitter amino acids and energy metabolism during cerebral ischemia in rats monitored by microdialysis and in vivo magnetic resonance spectroscopy	1993	605	33-42	Brain Res
Shou, M.; Smith, A. D.; Shackman, J. G.; Peris, J.; Kennedy, R. T.	In vivo monitoring of amino acids by microdialysis sampling with on-line derivatization by naphthalene-2,3-dicarboxyaldehyde and rapid micellar electrokinetic capillary chromatography	2004	138	189-197	J Neurosci Methods
Sierra-Paredes, G.; Galan-Valiente, J.; Vazquez-Illanes, M. D.; Aguilar-Veiga, E.; Soto-Otero, R.; Mendez-Alvarez, E.; Sierra-Marcuno, G.	Extracellular amino acids in the rat hippocampus during picrotoxin threshold seizures in chronic microdialysis experiments	1998	248	53-56	Neurosci Lett
Sierra-Paredes, G.; Loureiro, A. I.; Wright, L. C.; Sierra-Marcuno, G.; Soares-da-Silva, P.	Effects of eslicarbazepine acetate on acute and chronic latrunculin A-induced seizures and extracellular amino acid levels in the mouse hippocampus	2014	15	134	BMC Neurosci
Sierra-Paredes, G.; Oreiro-Garcia, M. T.; Vazquez-Illanes, M. D.; Sierra-Marcuno, G.	Effect of eslicarbazepine acetate (BIA 2-093) on latrunculin A-induced seizures and extracellular amino acid concentrations in the rat hippocampus	2007	77	36-43	Epilepsy Res

Glycine					
**Authors**	**Title**	**Year**	**Vol**	**Pages**	**Journal**
Silverstein, F. S.; Naik, B.; Simpson, J.	Hypoxia-ischemia stimulates hippocampal glutamate efflux in perinatal rat brain: an in vivo microdialysis study	1991	30	587-590	Pediatr Res
Silverstein, F. S.; Simpson, J.; Gordon, K. E.	Hypoglycemia alters striatal amino acid efflux in perinatal rats: an in vivo microdialysis study	1990	28	516-521	Ann Neurol
Simon, R. P.; Chen, J.; Graham, S. H.	GM1 ganglioside treatment of focal ischemia: a dose-response and microdialysis study	1993	265	24-29	J Pharmacol Exp Ther
Simpson Jr, R. K.; Robertson, C. S.; Goodman, J. C.	Segmental release of amino acid neurotransmitters from transcranial stimulation	1991	16	89-94	Neurochem Res
Slaney, T. R.; Mabrouk, O. S.; Porter-Stransky, K. A.; Aragona, B. J.; Kennedy, R. T.	Chemical gradients within brain extracellular space measured using low flow push-pull perfusion sampling in vivo	2013	4	321-329	ACS Chem Neurosci
Smith, A.; Watson, C. J.; Frantz, K. J.; Eppler, B.; Kennedy, R. T.; Peris, J.	Differential increase in taurine levels by low-dose ethanol in the dorsal and ventral striatum revealed by microdialysis with on-line capillary electrophoresis	2004	28	1028-1038	Alcohol Clin Exp Res
Smith, S. L.; Thompson, K. S. J.; Sargent, B. J.; Heal, D. J.	BTS 72664 - A novel CNS drug with potential anticonvulsant, neuroprotective, and antimigraine properties	2001	7	146-171	CNS Drug Reviews
Smolders, I.; Sarre, S.; Michotte, Y.; Ebinger, G.	The analysis of excitatory, inhibitory and other amino acids in rat brain microdialysates using microbore liquid chromatography	1995	57	47-53	J Neurosci Methods
Solas, A. B.; Munkeby, B. H.; Saugstad, O. D.	Comparison of short- and long-duration oxygen treatment after cerebral asphyxia in newborn piglets	2004	56	125-131	Pediatr Res
Song, P.; Mabrouk, O. S.; Hershey, N. D.; Kennedy, R. T.	In vivo neurochemical monitoring using benzoyl chloride derivatization and liquid chromatography-mass spectrometry	2012	84	412-419	Anal Chem
Soukupova, M.; Dolezal, T.; Prochazkova, M.; Janovsky, M.; Tuma, P.; Kubicek, V.; Krsiak, M.	The effect of paracetamol on periaqueductal gray release of glycine in the inflammatory pain	2009	13	S84	Eur J Pain
Stoffel, M.; Eriskat, J.; Plesnila, M.; Aggarwal, N.; Baethmann, A.	The penumbra zone of a traumatic cortical lesion: a microdialysis study of excitatory amino acid release	1997	70	91-93	Acta Neurochir Suppl
Stoffel, M.; Plesnila, N.; Eriskat, J.; Furst, M.; Baethmann, A.	Release of excitatory amino acids in the penumbra of a focal cortical necrosis	2002	19	467-477	J Neurotrauma

**Glycine**					
**Authors**	**Title**	**Year**	**Vol**	**Pages**	**Journal**
Storozheva, Z. I.; Proshin, A. T.	Selective involvement of the neurotransmitter systems of the cerebellum in the mechanisms forming different types of defensive behavior	2011	41	964-972	Neurosci Behav Physiol
Storozheva, Z. I.; Proshin, A. T.	[Selective involvement of cerebellar neurotransmitter systems in mechanisms of various types of defensive behavior]	2010	60	474-485	Zh Vyssh Nerv Deiat Im I P Pavlova
Suarez, L. M.; Munoz, M. D.; Gonzalez, J. C.; Bustamante, J.; Del Rio, R. M.; Solis, J. M.	The taurine transporter substrate guanidinoethyl sulfonate mimics the action of taurine on long-term synaptic potentiation	2016	48	2647-2656	Amino Acids
Sved, A. F.; Curtis, J. T.	Amino acid neurotransmitters in nucleus tractus solitarius: an in vivo microdialysis study	1993	61	2089-2098	J Neurochem
Swanson, R. A.; Chen, J.; Graham, S. H.	Glucose can fuel glutamate uptake in ischemic brain	1994	14	1-6	J Cereb Blood Flow Metab
Szeg, E. M.; Kekesi, K. A.; Szabo, Z.; Janaky, T.; Juhasz, G. D.	Estrogen regulates cytoskeletal flexibility, cellular metabolism and synaptic proteins: A proteomic study	2010	35	807-819	Psychoneuroendocrinology
Szyndler, J.; Maciejak, P.; Turzynska, D.; Sobolewska, A.; Lehner, M.; Taracha, E.; Walkowiak, J.; Skorzewska, A.; Wislowska-Stanek, A.; Hamed, A.; Bidzinski, A.; Plaznik, A.	Changes in the concentration of amino acids in the hippocampus of pentylenetetrazole-kindled rats	2008	439	245-249	Neurosci Lett
Takagi, K.; Ginsberg, M. D.; Globus, M. Y.; Dietrich, W. D.; Martinez, E.; Kraydieh, S.; Busto, R.	Changes in amino acid neurotransmitters and cerebral blood flow in the ischemic penumbral region following middle cerebral artery occlusion in the rat: correlation with histopathology	1993	13	575-585	J Cereb Blood Flow Metab
Takeda, A.; Hirate, M.; Tamano, H.; Oku, N.	Release of glutamate and GABA in the hippocampus under zinc deficiency	2003	72	537-542	J Neurosci Res
Takeda, A.; Minami, A.; Seki, Y.; Nakajima, S.; Oku, N.	Release of amino acids by zinc in the hippocampus	2004	63	253-257	Brain Res Bull
Takeda, A.; Sakurada, N.; Kanno, S.; Ando, M.; Oku, N.	Vulnerability to seizures induced by potassium dyshomeostasis in the hippocampus in aged rats	2008	54	37-42	J Health Sci
Takeda, A.; Sotogaku, N.; Oku, N.	Influence of manganese on the release of neurotransmitters in rat striatum	2003	965	279-282	Brain Res

Glycine					
**Authors**	**Title**	**Year**	**Vol**	**Pages**	**Journal**
Takeda, A.; Sotogaku, N.; Oku, N.	Manganese influences the levels of neurotransmitters in synapses in rat brain	2002	114	669-674	Neuroscience
Takeda, A.; Tamano, H.; Kan, F.; Hanajima, T.; Yamada, K.; Oku, N.	Enhancement of social isolation-induced aggressive behavior of young mice by zinc deficiency	2008	82	909-914	Life Sci
Tan, W. K.; Williams, C. E.; During, M. J.; Mallard, C. E.; Gunning, M. I.; Gunn, A. J.; Gluckman, P. D.	Accumulation of cytotoxins during the development of seizures and edema after hypoxic-ischemic injury in late gestation fetal sheep	1996	39	791-797	Pediatr Res
Tanaka, S.; Kiuchi, Y.; Tsuchida, A.; Yamamoto, T.; Oguchi, K.; Yoshida, T.; Kuroiwa, Y.	Possible involvement of an increase in brain extracellular concentration of excitatory amino acids in convulsions in phenobarbital-withdrawn rats	1996	17	91-102	Res Commun Alcohol S
Tang, Y. B.; Sun, F.; Teng, L.; Li, W. B.; An, S. M.; Zhang, C.; Yang, X. J.; Lv, H. Y.; Ding, X. P.; Zhu, L.; Chen, H. Z.	Simultaneous determination of the repertoire of classical neurotransmitters released from embryonal carcinoma stem cells using online microdialysis coupled with hydrophilic interaction chromatography-tandem mass spectrometry	2014	849	70-79	Anal Chim Acta
Taylor, D. L.; Davies, S. E.; Obrenovitch, T. P.; Doheny, M. H.; Patsalos, P. N.; Clark, J. B.; Symon, L.	Investigation into the role of N-acetylaspartate in cerebral osmoregulation	1995	65	275-281	J Neurochem
Tin Tin Win, Shwe; Mitsushima, D.; Yamamoto, S.; Fukushima, A.; Funabashi, T.; Kobayashi, T.; Fujimaki, H.	Changes in neurotransmitter levels and proinflammatory cytokine mRNA expressions in the mice olfactory bulb following nanoparticle exposure	2008	226	192-198	Toxicol Appl Pharmacol
Togashi, H.; Nakamura, K.; Matsumoto, M.; Ueno, K.; Ohashi, S.; Saito, H.; Yoshioka, M.	Aniracetam enhances glutamatergic transmission in the prefrontal cortex of stroke-prone spontaneously hypertensive rats	2002	320	109-112	Neurosci Lett
Tossman, U.; Delin, A.; Eriksson, L. S.; Ungerstedt, U.	Brain cortical amino acids measured by intracerebral dialysis in portacaval shunted rats	1987	12	265-269	Neurochem Res
Tossman, U.; Eriksson, S.; Delin, A.; Hagenfeldt, L.; Law, D.; Ungerstedt, U.	Brain amino acids measured by intracerebral dialysis in portacaval shunted rats	1983	41	1046-1051	J Neurochem
Tossman, U.; Jonsson, G.; Ungerstedt, U.	Regional distribution and extracellular levels of amino acids in rat central nervous system	1986	127	533-545	Acta Physiol Scand

Glycine					
**Authors**	**Title**	**Year**	**Vol**	**Pages**	**Journal**
Toth, E.	Effect of nicotine on the level of extracellular amino acids in the hippocampus of rat	1996	21	903-907	Neurochem Res
Toth, E.; Harsing Jr, L. G.; Sershen, H.; Ramacci, M. T.; Lajtha, A.	Effect of acetyl-L-carnitine on extracellular amino acid levels in vivo in rat brain regions	1993	18	573-578	Neurochem Res
Toth, E.; Vizi, E. S.; Lajtha, A.	Effect of nicotine on levels of extracellular amino acids in regions of the rat brain in vivo	1993	32	827-832	Neuropharmacology
Toya, S.; Takatsuru, Y.; Kokubo, M.; Amano, I.; Shimokawa, N.; Koibuchi, N.	Early-life-stress affects the homeostasis of glutamatergic synapses	2014	40	3627-3634	Eur J Neurosci
Tseng, E. E.; Brock, M. V.; Kwon, C. C.; Annanata, M.; Lange, M. S.; Troncoso, J. C.; Johnston, M. V.; Baumgartner, W. A.	Increased intracerebral excitatory amino acids and nitric oxide after hypothermic circulatory arrest	1999	67	371-376	Ann Thorac Surg
Tseng, E. E.; Brock, M. V.; Lange, M. S.; Troncoso, J. C.; Blue, M. E.; Lowenstein, C. J.; Johnston, M. V.; Baumgartner, W. A.	Monosialoganglioside GM1 inhibits neurotoxicity after hypothermic circulatory arrest	1998	124	298-306	Surgery
Tseng, E. E.; Brock, M. V.; Lange, M. S.; Troncoso, J. C.; Blue, M. E.; Lowenstein, C. J.; Johnston, M. V.; Baumgartner, W. A.	Glutamate excitotoxicity mediates neuronal apoptosis after hypothermic circulatory arrest	2010	89	440-445	Ann Thorac Surg
Tsukada, Y.; Kanamatsu, T.; Takahara, H.	Neurotransmitter release from the medial hyperstriatum ventrale of the chick forebrain accompanying filial imprinting behavior, measured by in vivo microdialysis	1999	24	315-320	Neurochem Res
Tuma, P.; Soukupova, M.; Samcova, E.; Stulik, K.	A determination of submicromolar concentrations of glycine in periaqueductal gray matter microdialyzates using capillary zone electrophoresis with contactless conductivity detection	2009	30	3436-3441	Electrophoresis
Tuma, P.; Sustkova-Fiserova, M.; Opekar, F.; Pavlicek, V.; Malkova, K.	Large-volume sample stacking for in vivo monitoring of trace levels of gamma-aminobutyric acid, glycine and glutamate in microdialysates of periaqueductal gray matter by capillary electrophoresis with contactless conductivity detection	2013	1303	94-99	J Chromatogr A

Glycine					
**Authors**	**Title**	**Year**	**Vol**	**Pages**	**Journal**
Ueda, Y.; Mitsuyama, Y.	Nitroprusside inhibits kindling development and potentiates excitatory amino acid exocytosis in the kindling rat	1997	23	49-60	J Brain Sci
Uutela, P.; Ketola, R. A.; Piepponen, P.; Kostiainen, R.	Comparison of different amino acid derivatives and analysis of rat brain microdialysates by liquid chromatography tandem mass spectrometry	2009	633	223-231	Anal Chim Acta
Varga, V.; Kekesi, A.; Juhasz, G.; Kocsis, B.	Reduction of the extracellular level of glutamate in the median raphe nucleus associated with hippocampal theta activity in the anaesthetized rat	1998	84	49-57	Neuroscience
Vasylieva, N.; Maucler, C.; Meiller, A.; Viscogliosi, H.; Lieutaud, T.; Barbier, D.; Marinesco, S.	Immobilization method to preserve enzyme specificity in biosensors: consequences for brain glutamate detection	2013	85	2507-2515	Anal Chem
Venton, B. J.; Robinson, T. E.; Kennedy, R. T.	Transient changes in nucleus accumbens amino acid concentrations correlate with individual responsivity to the predator fox odor 2,5-dihydro-2,4,5-trimethylthiazoline	2006	96	236-246	J Neurochem
Venton, B. J.; Robinson, T. E.; Kennedy, R. T.; Maren, S.	Dynamic amino acid increases in the basolateral amygdala during acquisition and expression of conditioned fear	2006	23	3391-3398	Eur J Neurosci
Vezzani, A.; Ungerstedt, U.; French, E. D.; Schwarcz, R.	In vivo brain dialysis of amino acids and simultaneous EEG measurements following intrahippocampal quinolinic acid injection: evidence for a dissociation between neurochemical changes and seizures	1985	45	335-344	J Neurochem
Voehringer, P.; Fuertig, R.; Ferger, B.	A novel liquid chromatography/tandem mass spectrometry method for the quantification of glycine as biomarker in brain microdialysis and cerebrospinal fluid samples within 5min	2013	939	92-97	J Chromatogr B Analyt Technol Biomed Life Sci
Wade, J. V.; Olson, J. P.; Samson, F. E.; Nelson, S. R.; Pazdernik, T. L.	A possible role for taurine in osmoregulation within the brain	1988	51	740-745	J Neurochem
Wang, J.; Shen, L. L.; Cao, Y. X.; Zhu, D. N.	Effects of electroacupuncture on pressor response to angiotensin-(1-7) by amino acid release in the rostral ventrolateral medulla	2003	28	25-34	Acupunct Electrother Res
Wang, L.; Huang, Y.; Wu, J.; Lv, G.;	Effect of Buyang Huanwu decoction on amino acid	2013	86	143-150	J Pharm Biomed Anal

Glycine					
**Authors**	**Title**	**Year**	**Vol**	**Pages**	**Journal**
Zhou, L.; Jia, J.	content in cerebrospinal fluid of rats during ischemic/reperfusion injury				
Wang, M.; Hershey, N. D.; Mabrouk, O. S.; Kennedy, R. T.	Collection, storage, and electrophoretic analysis of nanoliter microdialysis samples collected from awake animals in vivo	2011	400	2013-2023	Anal Bioanal Chem
Wang, Y. L.; He, R. R.	Effects of dipfluzine on cortical somatosensory evoked potentials and amino acid contents in ischemic rat brain	1996	17	41-44	Zhongguo Yao Li Xue Bao
Westergren, I.; Nystrom, B.; Hamberger, A.; Johansson, B. B.	Amino acids in extracellular fluid in vasogenic brain edema	1994	60	124-127	Acta Neurochir Suppl (Wien)
Westergren, I.; Nystrom, B.; Hamberger, A.; Nordborg, C.; Johansson, B. B.	Concentrations of amino acids in extracellular fluid after opening of the blood-brain barrier by intracarotid infusion of protamine sulfate	1994	62	159-165	J Neurochem
Westerink, B. H.; Damsma, G.; de Vries, J. B.	Effect of ouabain applied by intrastriatal microdialysis on the in vivo release of dopamine, acetylcholine, and amino acids in the brain of conscious rats	1989	52	705-712	J Neurochem
Westerink, B. H.; Hofsteede, H. M.; Damsma, G.; de Vries, J. B.	The significance of extracellular calcium for the release of dopamine, acetylcholine and amino acids in conscious rats, evaluated by brain microdialysis	1988	377	373-378	Naunyn Schmiedebergs Arch Pharmacol
Wilson, C. L.; Maidment, N. T.; Shomer, M. H.; Behnke, E. J.; Ackerson, L.; Fried, I.; Engel, J., Jr.	Comparison of seizure related amino acid release in human epileptic hippocampus versus a chronic, kainate rat model of hippocampal epilepsy	1996	26	245-254	Epilepsy Res
Wong, J. M.; Malec, P. A.; Mabrouk, O. S.; Ro, J.; Dus, M.; Kennedy, R. T.	Benzoyl chloride derivatization with liquid chromatography-mass spectrometry for targeted metabolomics of neurochemicals in biological samples	2016	1446	78-90	J Chromatogr A
Xu, G. Y.; McAdoo, D. J.; Hughes, M. G.; Robak, G.; De Castro Jr, R.	Considerations in the determination by microdialysis of resting extracellular amino acid concentrations and release upon spinal cord injury	1998	86	1011-1021	Neuroscience
Xu, P.; Li, J.; Chen, B.; Wang, X.; Cai, X.; Jiang, H.; Wang, C.; Zhang, H.	The real-time neurotoxicity analysis of Fe3O4 nanoparticles combined with daunorubicin for rat brain in vivo	2012	8	417-423	J Biomed Nanotechnol
Yager, J. Y.; Armstrong, E. A.; Miyashita, H.; Wirrell, E. C.	Prolonged neonatal seizures exacerbate hypoxic-ischemic brain damage: correlation with cerebral energy metabolism and excitatory amino acid release	2002	24	367-381	Dev Neurosci

Glycine					
**Authors**	**Title**	**Year**	**Vol**	**Pages**	**Journal**
Yamada, K.; Moriguchi, A.; Mikami, H.; Okuda, N.; Higaki, J.; Ogihara, T.	The effect of central amino acid neurotransmitters on the antihypertensive response to angiotensin blockade in spontaneous hypertension	1995	13	1624-1630	J Hypertens
Yamada, T.; Terashima, T.; Kawano, S.; Furuno, R.; Okubo, T.; Juneja, L. R.; Yokogoshi, H.	Theanine, gamma-glutamylethylamide, a unique amino acid in tea leaves, modulates neurotransmitter concentrations in the brain striatum interstitium in conscious rats	2009	36	21-27	Amino Acids
Yamada, T.; Terashima, T.; Okubo, T.; Juneja, L. R.; Yokogoshi, H.	Effects of theanine, r-glutamylethylamide, on neurotransmitter release and its relationship with glutamic acid neurotransmission	2005	8	219-226	Nutr Neurosci
Yamamoto, S.; Morinobu, S.; Iwamoto, Y.; Ueda, Y.; Takei, S.; Fujita, Y.; Yamawaki, S.	Alterations in the hippocampal glycinergic system in an animal model of posttraumatic stress disorder	2010	44	1069-1074	J Psychiatr Res
Yamanaka, T.; Sasa, M.; Matsunaga, T.	Glutamate as a primary afferent neurotransmitter in the medial vestibular nucleus as detected by in vivo microdialysis	1997	762	243-246	Brain Res
Yang, C. S.; Tsai, P. J.; Chen, W. Y.; Tsai, W. J.; Kuo, J. S.	On-line derivatization for continuous and automatic monitoring of brain extracellular glutamate levels in anesthetized rats: a microdialysis study	1999	734	1-6	J Chromatogr B Biomed Sci Appl
Yang, Z. Z.; Li, J.; Li, S. X.; Feng, W.; Wang, H.	Effect of ginkgolide B on striatal extracellular amino acids in middle cerebral artery occluded rats	2011	136	117-122	J Ethnopharmacol
Yu, H. L.; An, Y.; Jiang, H. Y.; Jin, Q. H.; Jin, Y. Z.	Changes of amino acid concentrations in the rat medial vestibular nucleus following unilateral labyrinthectomy	2007	59	71-78	Sheng Li Xue Bao
Zappettini, S.; Grilli, M.; Olivero, G.; Mura, E.; Preda, S.; Govoni, S.; Salamone, A.; Marchi, M.	Beta Amyloid Differently Modulate Nicotinic and Muscarinic Receptor Subtypes which Stimulate in vitro and in vivo the Release of Glycine in the Rat Hippocampus	2012	3	146	Front Pharmacol
Zappettini, S.; Mura, E.; Grilli, M.; Preda, S.; Salamone, A.; Olivero, G.; Govoni, S.; Marchi, M.	Different presynaptic nicotinic receptor subtypes modulate in vivo and in vitro the release of glycine in the rat hippocampus	2011	59	729-738	Neurochem Int
Zhang, D.; Zhang, J.; Ma, W.; Chen, D.; Han, H.; Shu, H.; Liu, G.	Analysis of trace amino acid neurotransmitters in hypothalamus of rats after exhausting exercise using microdialysis	2001	758	277-282	J Chromatogr B Biomed Sci Appl

Glycine					
**Authors**	**Title**	**Year**	**Vol**	**Pages**	**Journal**
Zhang, N.; Liu, P.; He, X.	[Effect of single-used borneol and combining it with diazepam on content of neurotransmitter in corpus striatum of rats]	2011	36	3180-3183	Zhongguo Zhong Yao Za Zhi
Zhang, N.; Liu, P.; He, X.	Effect of single-use versus combined-use moschus and diazepam on expression of amino acid neurotransmitters in the rat corpus striatum	2012	7	182-186	Neural Regen Res
Zhang, N.; Liu, P.; He, X.	Effect of borneol, moschus, storax, and acorus tatarinowii on expression levels of four amino acid neurotransmitters in the rat corpus striatum	2012	7	440-444	Neural Regen Res
Zhang, W. Q.; Hudson, P. M.; Sobotka, T. J.; Hong, J. S.; Tilson, H. A.	Extracellular concentrations of amino acid transmitters in ventral hippocampus during and after the development of kindling	1991	540	315-318	Brain Res
Zhang, W. Q.; Rogers, B. C.; Tandon, P.; Hudson, P. M.; Sobotka, T. J.; Hong, J. S.; Tilson, H. A.	Systemic administration of kainic acid increases GABA levels in perfusate from the hippocampus of rats in vivo	1990	11	593-600	Neurotoxicology
Zhao, P.; Cheng, J.	Effects of electroacupuncture on extracellular contents of amino acid neurotransmitters in rat striatum following transient focal cerebral ischemia	1997	22	119-126	Acupunct Electrother Res
Zhou, J.; Lunte, S. M.	Direct determination of amino acids by capillary electrophoresis/electrochemistry using a copper microelectrode and zwitterionic buffers	1995	16	498-503	Electrophoresis
Zhu, D. N.; Moriguchi, A.; Mikami, H.; Higaki, J.; Ogihara, T.	Central amino acids mediate cardiovascular response to angiotensin II in the rat	1998	45	189-197	Brain Res Bull
Zwingmann, C.; Desjardins, P.; Hazell, A.; Chatauret, N.; Michalak, A.; Butterworth, R. F.	Reduced expression of astrocytic glycine transporter (Glyt-1) in acute liver failure	2002	17	263-273	Metab Brain Dis

Appendix 7: Studies describing baseline dialysate concentrations for histamine

**Histamine**					
**Authors**	**Title**	**Year**	**Vol.**	**Pages**	**Journal**
Acworth, I. N.; Yu, J.; Ryan, E.; Gariepy, K. C.; Gamache, P.; Hull, K.; Maher, T.	Simultaneous measurement of monoamine, amino acid, and drug levels, using high performance liquid chromatography and coulometric array technology: Application to in vivo microdialysis perfusate analysis	1994	17	685-705	J Liq Chromatogr
Akins, V. F.; Bealer, S. L.	Hypothalamic histamine release, neuroendocrine and cardiovascular responses during tuberomammillary nucleus stimulation in the conscious rat	1993	57	849-855	Neuroendocrinology
Akins, V. F.; Bealer, S. L.	Brain histamine regulates pressor responses to peripheral hyperosmolality	1990	259	R507-R513	Am J Physiol
Altinbas, B.; Topuz, B. B.; Ilhan, T.; Yilmaz, M. S.; Erdost, H.; Yalcin, M.	Activation of the central histaminergic system mediates arachidonic-acid-induced cardiovascular effects	2014	92	645-654	Can J Physiol Pharmacol
Altinbas, B.; Topuz, B. B.; Yilmaz, M. S.; Aydin, C.; Savci, V.; Jochem, J.; Aydin, S.; Yalcin, M.	The mediation of the central histaminergic system in the pressor effect of intracerebroventricularly injected melittin, a phospholipase A2 activator, in normotensive rats	2012	87	153-158	Prostaglandins Leukot Essent Fatty Acids
Barke, K. E.; Hough, L. B.	Characterization of basal and morphine-induced histamine release in the rat periaqueductal gray	1994	63	238-244	J Neurochem
Barke, K. E.; Hough, L. B.	Simultaneous measurement of opiate-induced histamine release in the periaqueductal gray and opiate antinociception: an in vivo microdialysis study	1993	266	934-942	J Pharmacol Exp Ther
Barke, K. E.; Hough, L. B.	Morphine-induced increases of extracellular histamine levels in the periaqueductal grey in vivo: a microdialysis study	1992	572	146-153	Brain Res
Bernsand, M.; Hakanson, R.; Norlen, P.	Tachyphylaxis of the ECL-cell response to PACAP: receptor desensitization and/or depletion of secretory products	2007	152	240-248	Br J Pharmacol
Blandina, P.	Functional implications of histaminergic neuron heterogeneity	2010	59	S313	Inflamm Res
Bourgogne, E.; Mathy, F. X.; Boucaut, D.; Boekens, H.; Laprevote, O.	Simultaneous quantitation of histamine and its major metabolite 1-methylhistamine in brain dialysates by using precolumn derivatization prior to HILIC-MS/MS analysis	2012	402	449-459	Anal Bioanal Chem
Boyd, B. W.; Witowski, S. R.; Kennedy, R. T.	Trace-level amino acid analysis by capillary liquid chromatography and application to in vivo microdialysis sampling with 10-s temporal resolution	2000	72	865-871	Anal Chem
Chikai, T.; Oishi, R.; Saeki, K.	Microdialysis study of the effects of sedative drugs on	1993	266	1277-	J Pharmacol Exp Ther

Histamine					
**Authors**	**Title**	**Year**	**Vol.**	**Pages**	**Journal**
	extracellular histamine in the striatum of freely moving rats			1281	
Chikai, T.; Saeki, K.	Effect of minute amounts of [D-Ala2,MePhe4,Gly(ol)5]enkephalin injected into the tuberomammillary nucleus of rats on histamine release from the cerebral cortex	1995	196	137-139	Neurosci Lett
Choi, Y. H.; Chang, N.; Fletcher, P. J.; Anderson, G. H.	Dietary protein content affects the profiles of extracellular amino acids in the medial preoptic area of freely moving rats	2000	66	1105-1118	Life Sci
Chu, M.; Huang, Z. L.; Qu, W. M.; Eguchi, N.; Yao, M. H.; Urade, Y.	Extracellular histamine level in the frontal cortex is positively correlated with the amount of wakefulness in rats	2004	49	417-420	Neurosci Res
Cumming, P.; Damsma, G.; Fibiger, H. C.; Vincent, S. R.	Characterization of extracellular histamine in the striatum and bed nucleus of the stria terminalis of the rat: an in vivo microdialysis study	1991	56	1797-1803	J Neurochem
Dolgodilina, E.; Imobersteg, S.; Laczko, E.; Welt, T.; Verrey, F.; Makrides, V.	Brain interstitial fluid glutamine homeostasis is controlled by blood-brain barrier SLC7A5/LAT1 amino acid transporter	2016	36	1929-1941	J Cereb Blood Flow Metab
Dugovic, C.; Shelton, J.; Aluisio, L.; Fraser, I.; Sutton, S.; Pascal, B.; Yun, S.; Li, X.; Dvorak, C.; Lovenberg, T.	Blockade of orexin-1 receptors attenuates orexin-2 receptor antagonism induced sleep promotion in the rat	2009	32	A1	Sleep
Endou, M.; Yanai, K.; Sakurai, E.; Fukudo, S.; Hongo, M.; Watanabe, T.	Food-deprived activity stress decreased the activity of the histaminergic neuron system in rats	2001	891	32-41	Brain Research
Fell, M. J.; Flik, G.; Dijkman, U.; Folgering, J. H.; Perry, K. W.; Johnson, B. J.; Westerink, B. H.; Svensson, K. A.	Glutamatergic regulation of brain histamine neurons: In vivo microdialysis and electrophysiology studies in the rat	2015	99	1-8	Neuropharmacology
Fell, M. J.; Katner, J. S.; Johnson, B. G.; Khilevich, A.; Schkeryantz, J. M.; Perry, K. W.; Svensson, K. A.	Activation of metabotropic glutamate (mGlu)2 receptors suppresses histamine release in limbic brain regions following acute ketamine challenge	2010	58	632-639	Neuropharmacology
Fell, M. J.; Katner, J. S.; Rasmussen, K.; Nikolayev, A.; Kuo, M. S.; Nelson, D. L.; Perry, K. W.; Svensson, K. A.	Typical and atypical antipsychotic drugs increase extracellular histamine levels in the rat medial prefrontal cortex: contribution of histamine h(1) receptor blockade	2012	3	49	Front Psychiatry
Flik, G.; Dremencov, E.; Cremers, T. I.; Folgering, J. H.; Westerink, B. H.	The role of cortical and hypothalamic histamine-3 receptors in the modulation of central histamine neurotransmission: an in vivo electrophysiology and microdialysis study	2011	34	1747-1755	Eur J Neurosci
Fujimoto, Y.; Funao, T.; Suehiro, K.; Takahashi, R.; Mori, T.; Nishikawa, K.	Brain serotonin content regulates the manifestation of tramadol-induced seizures in rats: disparity between tramadol-induced	2015	122	178-189	Anesthesiology

Histamine					
Authors	Title	Year	Vol.	Pages	Journal
	seizure and serotonin syndrome				
Giannoni, P.; Medhurst, A. D.; Passani, M. B.; Giovannini, M. G.; Ballini, C.; Corte, L. D.; Blandina, P.	Regional differential effects of the novel histamine H3 receptor antagonist 6-[(3-cyclobutyl-2,3,4,5-tetrahydro-1H-3-benzazepin-7-yl)oxy]-N-methyl-3-pyridine carboxamide hydrochloride (GSK189254) on histamine release in the central nervous system of freely moving rats	2010	332	164-172	J Pharmacol Exp Ther
Giannoni, P.; Passani, M. B.; Nosi, D.; Chazot, P. L.; Shenton, F. C.; Medhurst, A. D.; Munari, L.; Blandina, P.	Heterogeneity of histaminergic neurons in the tuberomammillary nucleus of the rat	2009	29	2363-2374	Eur J Neurosci
Harusawa, S.; Imazu, T.; Takashima, S.; Araki, L.; Ohishi, H.; Kurihara, T.; Yamamoto, Y.; Atsushi Yamatodani, B.	Synthesis of imifuramine and its stereoisomers exhibiting histamine H3- agonistic activity	1999	40	2561-2564	Tetrahedron Lett
Hong, Z. Y.; Huang, Z. L.; Qu, W. M.; Eguchi, N.	Orexin A promotes histamine, but not norepinephrine or serotonin, release in frontal cortex of mice	2005	26	155-159	Acta Pharmacol Sin
Hong, Z. Y.; Huang, Z. L.; Qu, W. M.; Eguchi, N.; Urade, Y.; Hayaishi, O.	An adenosine A receptor agonist induces sleep by increasing GABA release in the tuberomammillary nucleus to inhibit histaminergic systems in rats	2005	92	1542-1549	J Neurochem
Horii, A.; Takeda, N.; Matsunaga, T.; Yamatodani, A.; Mochizuki, T.; Okakura- Mochizuki, K.; Wada, H.	Effect of unilateral vestibular stimulation on histamine release from the hypothalamus of rats in vivo	1993	70	1822-1826	J Neurophysiol
Horii, A.; Takeda, N.; Mochizuki, T.; Okakura-Mochizuki, K.; Yamamoto, Y.; Yamatodani, A.	Effects of vestibular stimulation on acetylcholine release from rat hippocampus: an in vivo microdialysis study	1994	72	605-611	J Neurophysiol
Horner, W. E.; Johnson, D. E.; Schmidt, A. W.; Rollema, H.	Methylphenidate and atomoxetine increase histamine release in rat prefrontal cortex	2007	558	96-97	Eur J Pharmacol
Huang, L.; Adachi, N.; Nagaro, T.; Liu, K.; Arai, T.	Histaminergic involvement in neuropathic pain produced by partial ligation of the sciatic nerve in rats	2007	32	124-129	Reg Anesth Pain Med
Huang, Z. L.; Mochizuki, T.; Qu, W. M.; Hong, Z. Y.; Watanabe, T.; Urade, Y.; Hayaishi, O.	Altered sleep-wake characteristics and lack of arousal response to H3 receptor antagonist in histamine H1 receptor knockout mice	2006	103	4687-4692	Proc Natl Acad Sci U S A
Huang, Z. L.; Qu, W. M.; Li, W. D.; Mochizuki, T.; Eguchi, N.; Watanabe, T.; Urade, Y.; Hayaishi, O.	Arousal effect of orexin A depends on activation of the histaminergic system	2001	98	9965-9970	Proc Natl Acad Sci U S A

Histamine					
**Authors**	**Title**	**Year**	**Vol.**	**Pages**	**Journal**
Huang, Z. L.; Sato, Y.; Mochizuki, T.; Okada, T.; Qu, W. M.; Yamatodani, A.; Urade, Y.; Hayaishi, O.	Prostaglandin E2 activates the histaminergic system via the EP4 receptor to induce wakefulness in rats	2003	23	5975-5983	J Neurosci
Hutson, P. H.; Heins, M. S.; Folgering, J. H.	Effects of lisdexamfetamine alone and in combination with s-citalopram on acetylcholine and histamine efflux in the rat pre-frontal cortex and ventral hippocampus	2015	134	693-703	J Neurochem
Irisawa, Y.; Adachi, N.; Liu, K.; Arai, T.; Nagaro, T.	Alleviation of ischemia-induced brain edema by activation of the central histaminergic system in rats	2008	108	112-123	J Pharmacol Sci
Ishizuka, T.; Hatano, K.; Murotani, T.; Yamatodani, A.	Comparison of the effect of an H(3)-inverse agonist on energy intake and hypothalamic histamine release in normal mice and leptin resistant mice with high fat diet-induced obesity	2008	188	250-254	Behav Brain Res
Ishizuka, T.; Murotani, T.; Yamatodani, A.	Modanifil activates the histaminergic system through the orexinergic neurons	2010	483	193-196	Neurosci Lett
Ishizuka, T.; Nomura, S.; Hosoda, H.; Kangawa, K.; Watanabe, T.; Yamatodani, A.	A role of the histaminergic system for the control of feeding by orexigenic peptides	2006	89	295-300	Physiol Behav
Ishizuka, T.; Sakamoto, Y.; Sakurai, T.; Yamatodani, A.	Modafinil increases histamine release in the anterior hypothalamus of rats	2003	339	143-146	Neurosci Lett
Ishizuka, T.; Sako, N.; Murotani, T.; Morimoto, A.; Yamatodani, A.; Ohura, K.	The effect of hardness of food on amygdalar histamine release in rats	2010	1313	97-102	Brain Res
Ishizuka, T.; Yamamoto, Y.; Yamatodani, A.	The effect of orexin-A and -B on the histamine release in the anterior hypothalamus in rats	2002	323	93-96	Neurosci Lett
Itoh, Y.; Oishi, R.; Adachi, N.; Saeki, K.	A highly sensitive assay for histamine using ion-pair HPLC coupled with postcolumn fluorescent derivatization: its application to biological specimens	1992	58	884-889	J Neurochem
Itoh, Y.; Oishi, R.; Nishibori, M.; Saeki, K.	Characterization of histamine release from the rat hypothalamus as measured by in vivo microdialysis	1991	56	769-774	J Neurochem
Itoh, Y.; Oishi, R.; Saeki, K.	Feeding-induced increase in the extracellular concentration of histamine in rat hypothalamus as measured by in vivo microdialysis	1991	125	235-257	Neurosci Lett
Jansen, F. P.; Mochizuki, T.; Yamamoto, Y.; Timmerman, H.; Yamatodani, A.	In vivo modulation of rat hypothalamic histamine release by the histamine H3 receptor ligands, immepip and clobenpropit. Effects of intrahypothalamic and peripheral application	1998	362	149-155	Eur J Pharmacol

Histamine					
**Authors**	**Title**	**Year**	**Vol.**	**Pages**	**Journal**
Jochem, J.; Altinbas, B.; Yalcin, M.; Ottani, A.; Giuliani, D.; Savci, V.; Kasperska-Zajac, A.; Guarini, S.	Involvement of the histaminergic system in the resuscitating effect of centrally acting leptin in haemorrhagic shock in rats	2016	67	67-74	J Physiol Pharmacol
Jochem, J.; Savci, V.; Filiz, N.; Rybus-Kalinowska, B.; Fogel, W. A.; Yalcin, M.	Involvement of the histaminergic system in cytidine 5'-diphosphocholine-induced reversal of critical haemorrhagic hypotension in rats	2010	61	37-43	J Physiol Pharmacol
Johnson, D. E.; Drummond, E.; Grimwood, S.; Sawant-Basak, A.; Miller, E.; Tseng, E.; McDowell, L. L.; Vanase-Frawley, M. A.; Fisher, K. E.; Rubitski, D. M.; Stutzman-Engwall, K. J.; Nelson, R. T.; Horner, W. E.; Gorczyca, R. R.; Hajos, M.; Siok, C. J.	The 5-hydroxytryptamine4 receptor agonists prucalopride and PRX-03140 increase acetylcholine and histamine levels in the rat prefrontal cortex and the power of stimulated hippocampal theta oscillations	2012	341	681-691	J Pharmacol Exp Ther
Johnson, D. E.; Gorczyca, R. R.; Horner, W. E.; Abitoye, P. A.; Connor, R. E.; Salafia, M. A.; Hughes, Z. A.	Dopamine d1 agonists modulate the release of multiple neurotransmitters associated with cognition	2011	1	284S	Biol Psychiatry
Kanamaru, M.; Iwase, M.; Homma, I.	Autoregulation of histamine release in medulla oblongata via H3-receptors in rabbits	1998	31	53-60	Neurosci Res
Laitinen, K. S.; Tuomisto, L.; Laitinen, J. T.	Endogenous serotonin modulates histamine release in the rat hypothalamus as measured by in vivo microdialysis	1995	285	159-164	Eur J Pharmacol
Laitinen, K. S.; Tuomisto, L.; MacDonald, E.	Effects of a selective alpha 2-adrenoceptor antagonist, atipamezole, on hypothalamic histamine and noradrenaline release in vivo	1995	285	255-260	Eur J Pharmacol
Lamberty, Y.; Margineanu, D. G.; Dassesse, D.; Klitgaard, H.	H3 agonist immepip markedly reduces cortical histamine release, but only weakly promotes sleep in the rat	2003	48	193-198	Pharmacol Res
Langlais, P. J.; Zhang, S. X.; Weilersbacher, G.; Hough, L. B.; Barke, K. E.	Histamine-mediated neuronal death in a rat model of Wernicke's encephalopathy	1994	38	565-574	J Neurosci Res
Liu, L. L.; Yang, J.; Lei, G. F.; Wang, G. J.; Wang, Y. W.; Sun, R. P.	Atomoxetine increases histamine release and improves learning deficits in an animal model of attention-deficit hyperactivity disorder: the spontaneously hypertensive rat	2008	102	527-532	Basic Clin Pharmacol Toxicol
Lo, E. H.; Pierce, A. R.; Matsumoto, K.; Kano, T.; Evans, C. J.; Newcomb, R.	Alterations in K+ evoked profiles of neurotransmitter and neuromodulator amino acids after focal ischemia-reperfusion	1998	83	449-458	Neuroscience

Histamine					
Authors	Title	Year	Vol.	Pages	Journal
Lozeva, V.; Attila, M.; Anttila, E.; Laitinen, K.; Hippelainen, M.; Tuomisto, L.	Brain histamine H3 receptors in rats with portacaval anastomosis: in vitro and in vivo studies	1998	358	574-581	Naunyn Schmiedebergs Arch Pharmacol
Malmlof, K.; Zaragoza, F.; Golozoubova, V.; Refsgaard, H. H.; Cremers, T.; Raun, K.; Wulff, B. S.; Johansen, P. B.; Westerink, B.; Rimvall, K.	Influence of a selective histamine H3 receptor antagonist on hypothalamic neural activity, food intake and body weight	2005	29	1402-1412	Int J Obes
Mammoto, T.; Yamamoto, Y.; Kagawa, K.; Hayashi, Y.; Mashimo, T.; Yoshiya, I.; Yamatodani, A.	Interactions between neuronal histamine and halothane anesthesia in rats	1997	69	406-411	J Neurochem
McRee, R. C.; Terry-Ferguson, M.; Langlais, P. J.; Chen, Y.; Nalwalk, J. W.; Blumenstock, F. A.; Hough, L. B.	Increased histamine release and granulocytes within the thalamus of a rat model of Wernicke's encephalopathy	2000	858	227-236	Brain Res
Mochizuki, T.; Yamatodani, A.; Okakura, K.; Horii, A.; Inagaki, N.; Wada, H.	Circadian rhythm of histamine release from the hypothalamus of freely moving rats	1992	51	391-394	Physiol Behav
Mochizuki, T.; Yamatodani, A.; Okakura, K.; Takemura, M.; Inagaki, N.; Wada, H.	In vivo release of neuronal histamine in the hypothalamus of rats measured by microdialysis	1991	343	190-195	Naunyn Schmiedebergs Arch Pharmacol
Morimoto, T.; Yamamoto, Y.; Yamatodani, A.	Leptin facilitates histamine release from the hypothalamus in rats	2000	868	367-369	Brain Res
Morimoto-Ishizuka, T.; Treesukosol, Y.; Yamamoto, Y.; Yamamoto, K.; Hashimoto, T.; Yamatodani, A.	Possible involvement of the chorda tympani nerve in stimulation of the histaminergic system by leptin	2002	51 Suppl1	S35-S36	Inflamm Res
Morimoto-Ishizuka, T.; Yamamoto, Y.; Yamatodani, A.	The role of the chorda tympani nerve in the activation of the rat hypothalamic histaminergic system by leptin	2001	300	107-110	Neurosci Lett
Motoki, A.; Adachi, N.; Semba, K.; Liu, K.; Arai, T.	Reduction in brain infarction by augmentation of central histaminergic activity in rats	2005	1066	172-178	Brain Res
Munari, L.; Galeotti, N.; Ohtsu, H.; Blandina, P.; Passani, M. B.	Oleoylethanolamide and brain histamine interact to regulate feeding behaviour	2010	59	s318	Inflamm Res
Munari, L.; Provensi, G.; Passani, M. B.; Galeotti, N.; Cassano, T.; Benetti, F.; Corradetti, R.; Blandina, P.	Brain histamine is crucial for selective serotonin reuptake inhibitors' behavioral and neurochemical effects	2015	18	1-10	Int J Neuropsychopharmacol
Munari, L.; Provensi, G.; Passani, M. B.;	Is brain histamine involved in depression?	2011	60	S339	Inflamm Res

Histamine					
**Authors**	**Title**	**Year**	**Vol.**	**Pages**	**Journal**
Galeotti, N.; Cassano, T.; Blandina, P.					
Murotani, T.; Ishizuka, T.; Isogawa, Y.; Karashima, M.; Yamatodani, A.	Possible involvement of serotonin 5-HT2 receptor in the regulation of feeding behavior through the histaminergic system	2011	61	228-233	Neuropharmacology
Niimi, M.; Mochizuki, T.; Yamamoto, Y.; Yamatodani, A.	Interleukin-1 beta induces histamine release in the rat hypothalamus in vivo	1994	181	87-90	Neurosci Lett
Nishida, N.; Huang, Z. L.; Mikuni, N.; Miura, Y.; Urade, Y.; Hashimoto, N.	Deep brain stimulation of the posterior hypothalamus activates the histaminergic system to exert antiepileptic effect in rat pentylenetetrazol model	2007	205	132-144	Exp Neurol
Nuutinen, S.; Maki, T.; Rozov, S.; Backstrom, P.; Hyytia, P.; Piepponen, P.; Panula, P.	Histamine H3 receptor antagonist decreases cue-induced alcohol reinstatement in mice	2016	106	156-163	Neuropharmacology
Okakura, K.; Yamatodani, A.; Mochizuki, T.; Horii, A.; Wada, H.	Glutamatergic regulation of histamine release from rat hypothalamus	1992	213	189-192	Eur J Pharmacol
Okakura-Mochizuki, K.; Mochizuki, T.; Yamamoto, Y.; Horii, A.; Yamatodani, A.	Endogenous GABA modulates histamine release from the anterior hypothalamus of the rat	1996	67	171-176	J Neurochem
Ortega, J. E.; Katner, J.; Davis, R.; Wade, M.; Nisenbaum, L.; Nomikos, G. G.; Svensson, K. A.; Perry, K. W.	Modulation of neurotransmitter release in orexin/hypocretin-2 receptor knockout mice: a microdialysis study	2012	90	588-596	J Neurosci Res
Provensi, G.; Munari, L.; Baldi, E.; Buccherelli, C.; Blandina, P.; Passani, M. B.	Histamine in the basolateral amygdala contributes to oleoylethanolamideinduced procognitive effects	2015	1	S19-S20	Inflamm Res
Qu, Y.; Li, Y.; Vandenbussche, E.; Vandesande, F.; Arckens, L.	In vivo microdialysis in the visual cortex of awake cat. II: sample analysis by microbore HPLC-electrochemical detection and capillary electrophoresis-laser-induced fluorescence detection	2001	7	45-51	Brain Res Brain Res Protocol
Rozov, S. V.; Zant, J. C.; Karlstedt, K.; Porkka-Heiskanen, T.; Panula, P.	Periodic properties of the histaminergic system of the mouse brain	2014	39	218-228	Eur J Neurosci
Russell, W. L.; Henry, D. P.; Phebus, L. A.; Clemens, J. A.	Release of histamine in rat hypothalamus and corpus striatum in vivo	1990	512	95-101	Brain Res
Seth, R.; Terry, D. E.; Parrish, B.; Bhatt, R.; Overton, J. M.	Amylin-leptin coadministration stimulates central histaminergic signaling in rats	2012	1442	15-24	Brain Res
Smagin, G.; Song, D.; Budac, D. P.; Perch, A.; Li, Y.; Sanchez, C.	Chronic treatment with vortioxetine activates central histaminergic system: A microdialysis study in rats	2014	1	391S	Biol Psychiatry
Song, P.; Mabrouk, O. S.; Hershey, N.	In vivo neurochemical monitoring using benzoyl chloride	2012	84	412-419	Anal Chem

Histamine					
**Authors**	**Title**	**Year**	**Vol.**	**Pages**	**Journal**
D.; Kennedy, R. T.	derivatization and liquid chromatography-mass spectrometry				
Strecker, R. E.; Nalwalk, J.; Dauphin, L. J.; Thakkar, M. M.; Chen, Y.; Ramesh, V.; Hough, L. B.; McCarley, R. W.	Extracellular histamine levels in the feline preoptic/anterior hypothalamic area during natural sleep-wakefulness and prolonged wakefulness: an in vivo microdialysis study	2002	113	663-670	Neuroscience
Tedford, C. E.; Yates, S. L.; Pawlowski, G. P.; Nalwalk, J. W.; Hough, L. B.; Khan, M. A.; Phillips, J. G.; Durant, G. J.; Frederickson, R. C.	Pharmacological characterization of GT-2016, a non-thiourea-containing histamine H3 receptor antagonist: in vitro and in vivo studies	1995	275	598-604	J Pharmacol Exp Ther
Treesukosol, Y.; Ishizuka, T.; Yamamoto, C.; Senda, K.; Tsutsumi, S.; Yamatodani, A.; Yamamoto, T.	Hypothalamic histamine release by taste stimuli in freely moving rats: possible implication of palatability	2005	164	67-72	Behav Brain Res
Treesukosol, Y.; Ishizuka, T.; Yamamoto, T.; Yamatodani, A.	The effect of taste stimuli on histamine release in the anterior hypothalamus of rats	2003	964	51-55	Brain Res
Uno, A.; Takeda, N.; Horii, A.; Morita, M.; Yamamoto, Y.; Yamatodani, A.; Kubo, T.	Histamine release from the hypothalamus induced by gravity change in rats and space motion sickness	1997	61	883-887	Physiol Behav
Valle-Dorado, M. G.; Santana-Gomez, C. E.; Orozco-Suarez, S. A.; Rocha, L.	The mast cell stabilizer sodium cromoglycate reduces histamine release and status epilepticus-induced neuronal damage in the rat hippocampus	2015	92	49-55	Neuropharmacology
Washington, B.; Shaw, J. B.; Li, J.; Fisher, B.; Gwathmey, J.	In vivo histamine release from brain cortex: the effects of modulating cellular and extracellular sodium and calcium channels	2000	407	117-122	Eur J Pharmacol
Westerink, B. H.; Cremers, T. I.; De Vries, J. B.; Liefers, H.; Tran, N.; De Boer, P.	Evidence for activation of histamine H3 autoreceptors during handling stress in the prefrontal cortex of the rat	2002	43	238-243	Synapse
Wong, J. M.; Malec, P. A.; Mabrouk, O. S.; Ro, J.; Dus, M.; Kennedy, R. T.	Benzoyl chloride derivatization with liquid chromatography-mass spectrometry for targeted metabolomics of neurochemicals in biological samples	2016	1446	78-90	J Chromatogr A
Yamamoto, Y.; Mochizuki, T.; Okakura-Mochizuki, K.; Uno, A.; Yamatodani, A.	Thioperamide, a histamine H3 receptor antagonist, increases GABA release from the rat hypothalamus	1997	19	289-298	Methods Find Exp Clin Pharmacol
Yoshikawa, T.; Nakamura, T.; Shibakusa, T.; Sugita, M.; Naganuma, F.; Iida, T.; Miura, Y.; Mohsen, A.;	Insufficient intake of L-histidine reduces brain histamine and causes anxiety-like behaviors in male mice	2014	144	1637-1641	J Nutr

Histamine					
**Authors**	**Title**	**Year**	**Vol.**	**Pages**	**Journal**
Harada, R.; Yanai, K.					
Yoshitake, S.; Ijiri, S.; Kehr, J.; Yoshitake, T.	Inhibition of histamine release by local and intracerebroventricular infusion of galanin in hypothalamus, hippocampus and prefrontal cortex of awake rat: A microdialysis study	2013	534	58-63	Neurosci Lett
Yoshitake, T.; Yamaguchi, M.; Nohta, H.; Ichinose, F.; Yoshida, H.; Yoshitake, S.; Fuxe, K.; Kehr, J.	Determination of histamine in microdialysis samples from rat brain by microbore column liquid chromatography following intramolecular excimer-forming derivatization with pyrene-labeling reagent	2003	127	11-17	J Neurosci Methods
Zant, J. C.; Rozov, S.; Wigren, H. K.; Panula, P.; Porkka-Heiskanen, T.	Histamine release in the basal forebrain mediates cortical activation through cholinergic neurons	2012	32	13244-13254	J Neurosci

Appendix 8: Studies describing baseline dialysate concentrations for proline

**Proline**					
**Authors**	**Title**	**Year**	**Vol**	**Pages**	**Journal**
Wong, J. M.; Malec, P. A.; Mabrouk, O. S.; Ro, J.; Dus, M.; Kennedy, R. T.	Benzoyl chloride derivatization with liquid chromatography-mass spectrometry for targeted metabolomics of neurochemicals in biological samples	2016	1446	78-79	J Chromatogr A
David, C. N.; Frias, E. S.; Szu, J. I.; Vieira, P. A.; Hubbard, J. A.; Lovelace, J.; Michael, M.; Worth, D.; McGovern, K. E.; Ethell, I. M.; Stanley, B. G.; Korzus, E.; Fiacco, T. A.; Binder, D. K.; Wilson, E. H.	GLT-1-Dependent Disruption of CNS Glutamate Homeostasis and Neuronal Function by the Protozoan Parasite Toxoplasma gondii	2016	12	e1005643	PLoS Pathog
Dolgodilina, E.; Imobersteg, S.; Laczko, E.; Welt, T.; Verrey, F.; Makrides, V.	Brain interstitial fluid glutamine homeostasis is controlled by blood-brain barrier SLC7A5/LAT1 amino acid transporter	2016	36	1929-1941	J Cereb Blood Flow Metab
Ganguly, P. K.; Maddaford, T. G.; Edel, A. L.; O, K.; Smeda, J. S.; Pierce, G. N.	Increased homocysteine-induced release of excitatory amino acids in the striatum of spontaneously hypertensive stroke-prone rats	2008	1226	192-198	Brain Res
Li, H.; Yan, Z. Y.	Analysis of amino acid neurotransmitters in hypothalamus of rats during cerebral ischemia-reperfusion by microdialysis and capillary electrophoresis	2010	21	1185-1192	Biomed Chromatogr
Yoneyama, D.; Shinozaki, Y.; Lu, W. L.; Tomi, M.; Tachikawa, M.; Hosoya, K.	Involvement of system A in the retina-to-blood transport of l-proline across the inner blood-retinal barrier	2010	90	507-513	Exp Eye Res

Appendix 9: Studies describing baseline dialysate concentrations for taurine

Taurine					
**Authors**	**Title**	**Year**	**Vol**	**Pages**	**Journal**
Abraham, I.; Juhasz, G.; Kekesi, K. A.; Kovacs, K. J.	Effect of intrahippocampal dexamethasone on the levels of amino acid transmitters and neuronal excitability	1996	733	56-63	Brain Res
Abraham, I.; Juhasz, G.; Kekesi, K. A.; Kovacs, K. J.	Corticosterone peak is responsible for stress-induced elevation of glutamate in the hippocampus	1998	2	171-181	Stress
Acworth, I. N.; Yu, J.; Ryan, E.; Gariepy, K. C.; Gamache, P.; Hull, K.; Maher, T.	Simultaneous measurement of monoamine, amino acid, and drug levels, using high performance liquid chromatography and coulometric array technology: Application to in vivo microdialysis perfusate analysis	1994	17	685-705	J Liq Chromatogr
Ahmad, S.; Fowler, L. J.; Whitton, P. S.	Lamotrigine, carbamazepine and phenytoin differentially alter extracellular levels of 5-hydroxytryptamine, dopamine and amino acids	2005	63	141-149	Epilepsy Res
Ahmad, S.; Fowler, L. J.; Whitton, P. S.	Effects of combined lamotrigine and valproate on basal and stimulated extracellular amino acids and monoamines in the hippocampus of freely moving rats	2005	371	1-8	Naunyn Schmiedebergs Arch Pharmacol
Ahmad, S.; Fowler, L. J.; Whitton, P. S.	Effects of acute and chronic lamotrigine treatment on basal and stimulated extracellular amino acids in the hippocampus of freely moving rats	2004	1029	41-47	Brain Res
Albrecht, J.; Hilgier, W.; Zielinska, M.; Januszewski, S.; Hesselink, M.; Quack, G.	Extracellular concentrations of taurine, glutamate, and aspartate in the cerebral cortex of rats at the asymptomatic stage of thioacetamide-induced hepatic failure: modulation by ketamine anesthesia	2000	25	1497-1502	Neurochem Res
Almaguer-Melian, W.; Cruz-Aguado, R.; Riva Cde, L.; Kendrick, K. M.; Frey, J. U.; Bergado, J.	Effect of LTP-reinforcing paradigms on neurotransmitter release in the dentate gyrus of young and aged rats	2005	327	877-883	Biochem Biophys Res Commun
Amakawa, K.; Adachi, N.; Liu, K.; Ikemune, K.; Fujitani, T.; Arai, T.	Effects of pre- and postischemic administration of thiopental on transmitter amino acid release and histologic outcome in gerbils	1996	85	1422-1430	Anesthesiology
Amantea, D.; Fratto, V.; Maida, S.; Rotiroti, D.; Ragusa, S.; Nappi, G.; Bagetta, G.; Corasaniti, M. T.	Prevention of Glutamate Accumulation and Upregulation of Phospho-Akt may Account for Neuroprotection Afforded by Bergamot Essential Oil against Brain Injury Induced by Focal Cerebral Ischemia in Rat	2009	85	389-405	Int Rev Neurobiol

Taurine					
**Authors**	**Title**	**Year**	**Vol**	**Pages**	**Journal**
Anderson, J. J.; DiMicco, J. A.	The use of microdialysis for studying the regional effects of pharmacological manipulation on extracellular levels of amino acids--some methodological aspects	1992	51	623-630	Life Sci
Anderson, J. J.; DiMicco, J. A.	Effect of local inhibition of gamma-aminobutyric acid uptake in the dorsomedial hypothalamus on extracellular levels of gamma-aminobutyric acid and on stress-induced tachycardia: a study using microdialysis	1990	255	1399-1407	J Pharmacol Exp Ther
Anderzhanova, E.; Rayevsky, K. S.; Saransaari, P.; Riitamaa, E.; Oja, S. S.	Effects of acute toxic doses of psychostimulants on extracellular levels of excitatory amino acids and taurine in rats: comparison of d-amphetamine and sydnocarb	2002	965	193-203	Ann N Y Acad Sci
Anderzhanova, E.; Rayevsky, K. S.; Saransaari, P.; Riitamaa, E.; Oja, S. S.	Effects of sydnocarb and D-amphetamine on the extracellular levels of amino acids in the rat caudate-putamen	2001	428	87-95	Eur J Pharmacol
Anderzhanova, E.; Saransaari, P.; Oja, S. S.	Neuroprotective mechanisms of taurine in vivo	2006	583	377-387	Adv Exp Med Biol
Andiarzhanova, E. A.; Saransaari, R.; Riitamaa, E.; Oiia, S. S.; Raevskii, K. S.	[Extracellular level of neuroactive amino acids in the rat neostriatum after treatment with psychostimulants (microdialysis study)]	2002	65	7-13	Eksp Klin Farmakol
Andine, P.; Orwar, O.; Jacobson, I.; Sandberg, M.; Hagberg, H.	Changes in extracellular amino acids and spontaneous neuronal activity during ischemia and extended reflow in the CA1 of the rat hippocampus	1991	57	222-229	J Neurochem
Bakkelund, A. H.; Fonnum, F.; Paulsen, R. E.	Evidence using in vivo microdialysis that aminotransferase activities are important in the regulation of the pools of transmitter amino acids	1993	18	411-415	Neurochem Res
Baldauf, K.; Braun, K.; Gruss, M.	Opiate modulation of monoamines in the chick forebrain: possible role in emotional regulation?	2005	62	149-163	J Neurobiol
Baldwin, H. A.; Williams, J. L.; Snares, M.; Ferreira, T.; Cross, A. J.; Green, A. R.	Attenuation by chlormethiazole administration of the rise in extracellular amino acids following focal ischaemia in the cerebral cortex of the rat	1994	112	188-194	Br J Pharmacol
Behrens, P. F.; Franz, P.; Woodman, B.; Lindenberg, K. S.; Landwehrmeyer, G. B.	Impaired glutamate transport and glutamate-glutamine cycling: downstream effects of the Huntington mutation	2002	125	1908-1922	Brain
Behrens, P. F.; Langemann, H.; Strohschein, R.; Draeger, J.; Hennig, J.	Extracellular glutamate and other metabolites in and around RG2 rat glioma: an intracerebral microdialysis	2000	47	11-22	J Neurooncol

Taurine					
**Authors**	**Title**	**Year**	**Vol**	**Pages**	**Journal**
	study				
Beitz, A. J.	Relationship of glutamate and aspartate to the periaqueductal gray-raphe magnus projection: analysis using immunocytochemistry and microdialysis	1990	38	1755-1765	J Histochem Cytochem
Beitz, A. J.; Saxon, D.	Harmaline-induced climbing fiber activation causes amino acid and peptide release in the rodent cerebellar cortex and a unique temporal pattern of Fos expression in the olivo-cerebellar pathway	2004	33	49-74	J Neurocytol
Benveniste, H.; Drejer, J.; Schousboe, A.; Diemer, N. H.	Elevation of the extracellular concentrations of glutamate and aspartate in rat hippocampus during transient cerebral ischemia monitored by intracerebral microdialysis	1984	43	1369-1374	J Neurochem
Bereiter, D. A.; Benetti, A. P.	Excitatory amino release within spinal trigeminal nucleus after mustard oil injection into the temporomandibular joint region of the rat	1996	67	451-459	Pain
Bereiter, D. A.; Shen, S.; Benetti, A. P.	Sex differences in amino acid release from rostral trigeminal subnucleus caudalis after acute injury to the TMJ region	2002	98	89-99	Pain
Bergquist, F.; Ludwig, M.; Dutia, M. B.	Role of the commissural inhibitory system in vestibular compensation in the rat	2008	586	4441-4452	J Physiol
Bianchi, L.; Colivicchi, M. A.; Bolam, J. P.; Della Corte, L.	The release of amino acids from rat neostriatum and substantia nigra in vivo: a dual microdialysis probe analysis	1998	87	171-180	Neuroscience
Bianchi, L.; Della Corte, L.; Tipton, K. F.	Simultaneous determination of basal and evoked output levels of aspartate, glutamate, taurine and 4-aminobutyric acid during microdialysis and from superfused brain slices	1999	723	47-59	J Chromatogr B Biomed Sci Appl
Bie, X.; Chen, Y.; Han, J.; Dai, H.; Wan, H.; Zhao, T.	Effects of gastrodin on amino acids after cerebral ischemia-reperfusion injury in rat striatum	2007	16 Suppl 1	305-308	Asia Pac J Clin Nutr
Biggs, C. S.; Fowler, L. J.; Whitton, P. S.; Starr, M. S.	Impulse-dependent and tetrodotoxin-sensitive release of GABA in the rat's substantia nigra measured by microdialysis	1995	684	172-178	Brain Res
Biggs, C. S.; Pearce, B. R.; Fowler, L. J.;	Effect of isonicotinic acid hydrazide on extracellular	1994	63	2197-	J Neurochem

Taurine					
**Authors**	**Title**	**Year**	**Vol**	**Pages**	**Journal**
Whitton, P. S.	amino acids and convulsions in the rat: Reversal of neurochemical and behavioural deficits by sodium valproate			2201	
Biggs, C. S.; Pearce, B. R.; Fowler, L. J.; Whitton, P. S.	The effect of sodium valproate on extracellular GABA and other amino acids in the rat ventral hippocampus: an in vivo microdialysis study	1992	594	138-142	Brain Res
Bland, S. T.; Gonzales, R. A.; Schallert, T.	Movement-related glutamate levels in rat hippocampus, striatum, and sensorimotor cortex	1999	277	119-122	Neurosci Lett
Boatell, M. L.; Bendahan, G.; Mahy, N.	Time-related cortical amino acid changes after basal forebrain lesion: a microdialysis study	1995	64	285-291	J Neurochem
Bockelmann, R.; Reiser, M.; Wolf, G.	Potassium-stimulated taurine release and nitric oxide synthase activity during quinolinic acid lesion of the rat striatum	1998	23	469-475	Neurochem Res
Bolea, I.; Colivicchi, M. A.; Ballini, C.; Fattori, M.; Marco-Contelles, J. L.; Unzeta, M.; Della Corte, L.	Protective effect of the MAO-B inhibitor PF9601N in a microdialysis model of excitotoxicity	2011	8	/	Neurodegener Dis Conference: 10th International Conference AD/PD Alzheimer's and Parkinson's Diseases: Advances, Concepts and New Challenges. Barcelona Spain. Conference Start
Bolea, I.; Colivicchi, M. A.; Ballini, C.; Marco-Contelles, J.; Tipton, K. F.; Unzeta, M.; Della Corte, L.	Neuroprotective effects of the MAO-B inhibitor, PF9601N, in an in vivo model of excitotoxicity	2014	20	641-650	CNS Neurosci Ther
Bosch, O. J.; Sartori, S. B.; Singewald, N.; Neumann, I. D.	Extracellular amino acid levels in the paraventricular nucleus and the central amygdala in high- and low-anxiety dams rats during maternal aggression: regulation by oxytocin	2007	10	261-270	Stress
Bosman, D. K.; Deutz, N. E.; Maas, M. A.; van Eijk, H. M.; Smit, J. J.; de Haan, J. G.; Chamuleau, R. A.	Amino acid release from cerebral cortex in experimental acute liver failure, studied by in vivo cerebral cortex microdialysis	1992	59	591-599	J Neurochem
Boyd, B. W.; Witowski, S. R.; Kennedy,	Trace-level amino acid analysis by capillary liquid	2000	72	865-871	Anal Chem

Taurine					
**Authors**	**Title**	**Year**	**Vol**	**Pages**	**Journal**
R. T.	chromatography and application to in vivo microdialysis sampling with 10-s temporal resolution				
Brennan, P. A.; Kendrick, K. M.; Keverne, E. B.	Neurotransmitter release in the accessory olfactory bulb during and after the formation of an olfactory memory in mice	1995	69	1075-1086	Neuroscience
Britton, P.; Whitton, P. S.; Fowler, L. J.; Bowery, N. G.	Tetanus toxin-induced effects on extracellular amino acid levels in rat hippocampus: an in vivo microdialysis study	1996	67	324-329	J Neurochem
Bruhn, T.; Christensen, T.; Diemer, N. H.	In vivo cellular uptake of glutamate is impaired in the rat hippocampus during and after transient cerebral ischemia: a microdialysis extraction study	2001	66	1118-1126	J Neurosci Res
Brust, P.; Christensen, T.; Diemer, N. H.	Decrease of extracellular taurine in the rat dorsal hippocampus after central nervous administration of vasopressin	1992	58	1427-1431	J Neurochem
Buisson, A.; Callebert, J.; Mathieu, E.; Plotkine, M.; Boulu, R. G.	Striatal protection induced by lesioning the substantia nigra of rats subjected to focal ischemia	1992	59	1153-1157	J Neurochem
Buratta, S.; Hamberger, A.; Ryberg, H.; Nystrom, B.; Sandberg, M.; Mozzi, R.	Effect of serine and ethanolamine administration on phospholipid-related compounds and neurotransmitter amino acids in the rabbit hippocampus	1998	71	2145-2150	J Neurochem
Butcher, S. P.; Bullock, R.; Graham, D. I.; McCulloch, J.	Correlation between amino acid release and neuropathologic outcome in rat brain following middle cerebral artery occlusion	1990	21	1727-1733	Stroke
Butcher, S. P.; Cameron, D.; Kendall, L.; Griffiths, R.	Homocysteine-induced alterations in extracellular amino acids in rat hippocampus	1992	20	75-80	Neurochem Int
Butcher, S. P.; Hamberger, A.	In vivo studies on the extracellular, and veratrine-releasable, pools of endogenous amino acids in the rat striatum: effects of corticostriatal deafferentation and kainic acid lesion	1987	48	713-721	J Neurochem
Butcher, S. P.; Jacobson, I.; Hamberger, A.	On the epileptogenic effects of kainic acid and dihydrokainic acid in the dentate gyrus of the rat	1988	27	375-381	Neuropharmacology
Butcher, S. P.; Lazarewicz, J. W.; Hamberger, A.	In vivo microdialysis studies on the effects of decortication and excitotoxic lesions on kainic acid-induced calcium fluxes, and endogenous amino acid	1987	49	1355-1360	J Neurochem

Taurine					
**Authors**	**Title**	**Year**	**Vol**	**Pages**	**Journal**
	release, in the rat striatum				
Butcher, S. P.; Sandberg, M.; Hagberg, H.; Hamberger, A.	Cellular origins of endogenous amino acids released into the extracellular fluid of the rat striatum during severe insulin-induced hypoglycemia	1987	48	722-728	J Neurochem
Camacho, A.; Montiel, T.; Massieu, L.	The anion channel blocker, 4,4'-dinitrostilbene-2,2'-disulfonic acid prevents neuronal death and excitatory amino acid release during glycolysis inhibition in the hippocampus in vivo	2006	142	1005-1017	Neuroscience
Campos, F.; Alfonso, M.; Duran, R.	In vivo modulation of alpha7 nicotinic receptors on striatal glutamate release induced by anatoxin-A	2010	56	850-855	Neurochem Int
Cassel, G. E.; Fosbraey, P.	Measurement of the oxime HI-6 after peripheral administration in tandem with neurotransmitter levels in striatal dialysates: effects of soman intoxication	1996	35	159-166	J Pharmacol Toxicol Methods
Chau, P.; Soderpalm, B.; Ericson, M.	The mGluR5 antagonist MPEP elevates accumbal dopamine and glycine levels; interaction with strychnine-sensitive glycine receptors	2011	16	591-599	Addict Biol
Chen, J.; Xu, W.; Jiang, H.	17 beta-estradiol protects neurons from ischemic damage and attenuates accumulation of extracellular excitatory amino acids	2001	92	1520-1523	Anesth Analg
Chen, R.; Deng, Y.; Yao, J.; Kamal, G. M.; Wang, J.; Xu, F.	Assessment of amino acid neurotransmitters in rat brain microdialysis samples by high-performance liquid chromatography with coulometric detection	2015	38	1439-1447	J Liq Chromatogr Relat Technol
Choi, Y. H.; Chang, N.; Fletcher, P. J.; Anderson, G. H.	Dietary protein content affects the profiles of extracellular amino acids in the medial preoptic area of freely moving rats	2000	66	1105-1118	Life Sci
Choi, Y. H.; Fletcher, P. J.; Anderson, G. H.	Extracellular amino acid profiles in the paraventricular nucleus of the rat hypothalamus are influenced by diet composition	2001	892	320-328	Brain Res
Choi, Y. H.; Fletcher, P. J.; Wong, C. C.; Anderson, G. H.	Measurement of blood-brain barrier permeability of rats with alpha-aminoisobutyric acid during microdialysis: possible application to behavioral studies	1999	67	587-598	Physiol Behav
Ciriacks, C. M.; Bowser, M. T.	Measuring the effect of glutamate receptor agonists on extracellular D-serine concentrations in the rat striatum	2006	393	200-205	Neurosci Lett

Taurine					
**Authors**	**Title**	**Year**	**Vol**	**Pages**	**Journal**
	using online microdialysis-capillary electrophoresis				
Corsi, C.; Pazzagli, M.; Bianchi, L.; Della Corte, L.; Pepeu, G.; Pedata, F.	In vivo amino acid release from the striatum of aging rats: adenosine modulation	1997	18	243-250	Neurobiol Aging
Currie, P. J.; Chang, N.; Luo, S.; Anderson, G. H.	Microdialysis as a tool to measure dietary and regional effects on the complete profile of extracellular amino acids in the hypothalamus of rats	1995	57	1911-1923	Life Sci
Dahchour, A.; De Witte, P.	Effect of repeated ethanol withdrawal on glutamate microdialysate in the hippocampus	1999	23	1698-1703	Alcohol Clin Exp Res
Dahchour, A.; De Witte, P.	Taurine blocks the glutamate increase in the nucleus accumbens microdialysate of ethanol-dependent rats	2000	65	345-350	Pharmacol Biochem Behav
Dahchour, A.; De Witte, P.	Acamprosate decreases the hypermotility during repeated ethanol withdrawal	1999	18	77-81	Alcohol
Dahchour, A.; De Witte, P.	Excitatory and inhibitory amino acid changes during repeated episodes of ethanol withdrawal: an in vivo microdialysis study	2003	459	171-178	Eur J Pharmacol
Dahchour, A.; Hoffman, A.; Deitrich, R.; de Witte, P.	Effects of ethanol on extracellular amino acid levels in high-and low-alcohol sensitive rats: a microdialysis study	2000	35	548-553	Alcohol Alcohol
Dahchour, A.; Quertemont, E.; De Witte, P.	Taurine increases in the nucleus accumbens microdialysate after acute ethanol administration to naive and chronically alcoholised rats	1996	735	9-19	Brain Res
Daisley, J. N.; Gruss, M.; Rose, S. P.; Braun, K.	Passive avoidance training and recall are associated with increased glutamate levels in the intermediate medial hyperstriatum ventrale of the day-old chick	1998	6	53-61	Neural Plast
Davies, S. E.; Gotoh, M.; Richards, D. A.; Obrenovitch, T. P.	Hypoosmolarity induces an increase of extracellular N-acetylaspartate concentration in the rat striatum	1998	23	1021-1025	Neurochem Res
Dawson, L. A.; Organ, A. J.; Winter, P.; Lacroix, L. P.; Shilliam, C. S.; Heidbreder, C.; Shah, A. J.	Rapid high-throughput assay for the measurement of amino acids from microdialysates and brain tissue using monolithic C18-bonded reversed-phase columns	2004	807	235-241	J Chromatogr B Analyt Technol Biomed Life Sci
Del Arco, A.; Castaneda, T. R.; Mora, F.	Amphetamine releases GABA in striatum of the freely moving rat: involvement of calcium and high affinity transporter mechanisms	1998	37	199-205	Neuropharmacology
Del Arco, A.; Gonzalez-Mora, J. L.; Armas, V. R.; Mora, F.	Amphetamine increases the extracellular concentration of glutamate in striatum of the awake rat: involvement of	1999	38	943-954	Neuropharmacology

Taurine					
**Authors**	**Title**	**Year**	**Vol**	**Pages**	**Journal**
	high affinity transporter mechanisms				
Del Arco, A.; Martinez, R.; Mora, F.	Amphetamine increases extracellular concentrations of glutamate in the prefrontal cortex of the awake rat: a microdialysis study	1998	23	1153-1158	Neurochem Res
Del Arco, A.; Mora, F.	Effects of endogenous glutamate on extracellular concentrations of GABA, dopamine, and dopamine metabolites in the prefrontal cortex of the freely moving rat: involvement of NMDA and AMPA/KA receptors	1999	24	1027-1035	Neurochem Res
Del Arco, A.; Segovia, G.; Mora, F.	Effects of endogenous glutamate on extracellular concentrations of taurine in striatum and nucleus accumbens of the awake rat: involvement of NMDA and AMPA/kainate receptors	2000	19	729-738	Amino Acids
Del Arco, A.; Segovia, G.; Prieto, L.; Mora, F.	Endogenous glutamate-taurine interaction in striatum and nucleus accumbens of the freely moving rat: studies during the normal process of aging	2001	122	401-414	Mech Ageing Dev
Delgado, J. M.; DeFeudis, F. V.; Roth, R. H.; Ryugo, D. K.; Mitruka, B. M.	Dialytrode for long term intracerebral perfusion in awake monkeys	1972	198	9-21	Arch Int Pharmacodyn Ther
Delgado, J. M.; Lerma, J.; Martin del Rio, R.; Solis, J. M.	Dialytrode technology and local profiles of amino acids in the awake cat brain	1984	42	1218-1228	J Neurochem
Devall, A. J.; Blake, R.; Langman, N.; Smith, C. G.; Richards, D. A.; Whitehead, K. J.	Monolithic column-based reversed-phase liquid chromatography separation for amino acid assay in microdialysates and cerebral spinal fluid	2007	848	323-328	J Chromatogr B Analyt Technol Biomed Life Sci
Doheny, M. H.; Nagaki, S.; Patsalos, P. N.	A microdialysis study of glycinamide, glycine and other amino acid neurotransmitters in rat frontal cortex and hippocampus after the administration of milacemide, a glycine pro-drug	1996	354	157-163	Naunyn Schmiedebergs Arch Pharmacol
Dolgodilina, E.; Imobersteg, S.; Laczko, E.; Welt, T.; Verrey, F.; Makrides, V.	Brain interstitial fluid glutamine homeostasis is controlled by blood-brain barrier SLC7A5/LAT1 amino acid transporter	2016	36	1929-1941	J Cereb Blood Flow Metab
Donzanti, B. A.; Yamamoto, B. K.	An improved and rapid HPLC-EC method for the isocratic separation of amino acid neurotransmitters from brain tissue and microdialysis perfusates	1988	43	913-922	Life Sci
Doretto, M. C.; Burger, R. L.; Mishra, P.	A microdialysis study of amino acid concentrations in the	1994	17	157-165	Epilepsy Res

Taurine					
**Authors**	**Title**	**Year**	**Vol**	**Pages**	**Journal**
K.; Garcia-Cairasco, N.; Dailey, J. W.; Jobe, P. C.	extracellular fluid of the substantia nigra of freely behaving GEPR-9s: relationship to seizure predisposition				
During, M. J.; Craig, J. S.; Hernandez, T. D.; Anderson, G. M.; Gallager, D. W.	Effect of amygdala kindling on the in vivo release of GABA and 5-HT in the dorsal raphe nucleus in freely moving rats	1992	584	36-44	Brain Res
Ebner, K.; Bosch, O. J.; Kromer, S. A.; Singewald, N.; Neumann, I. D.	Release of oxytocin in the rat central amygdala modulates stress-coping behavior and the release of excitatory amino acids	2005	30	223-230	Neuropsychopharmacology
Ehlen, J. C.; Albers, H. E.; Breyer, E. D.	MEKC-LIF of gamma-amino butyric acid in microdialysate: Systematic optimization of the separation conditions by factorial analysis	2005	147	36-47	J Neurosci Methods
Engelmann, M.; Bull, P. M.; Brown, C. H.; Landgraf, R.; Horn, T. F.; Singewald, N.; Ludwig, M.; Wotjak, C. T.	GABA selectively controls the secretory activity of oxytocin neurons in the rat supraoptic nucleus	2004	19	601-608	Eur J Neurosci
Engelmann, M.; Ludwig, M.; Singewald, N.; Ebner, K.; Sabatier, N.; Lubec, G.; Landgraf, R.; Wotjak, C. T.	Taurine selectively modulates the secretory activity of vasopressin neurons in conscious rats	2001	14	1047-1055	Eur J Neurosci
Engelmann, M.; Wolf, G.; Horn, T. F.	Release patterns of excitatory and inhibitory amino acids within the hypothalamic supraoptic nucleus in response to direct nitric oxide administration during forced swimming in rats	2002	324	252-254	Neurosci Lett
Espey, M. G.; Kustova, Y.; Sei, Y.; Basile, A. S.	Extracellular glutamate levels are chronically elevated in the brains of LP-BM5-infected mice: a mechanism of retrovirus-induced encephalopathy	1998	71	2079-2087	J Neurochem
Exposito, I.; Del Arco, A.; Segovia, G.; Mora, F.	Endogenous dopamine increases extracellular concentrations of glutamate and GABA in striatum of the freely moving rat: involvement of D1 and D2 dopamine receptors	1999	24	849-856	Neurochem Res
Fabre-Nys, C.; Blache, D.; Hinton, M. R.; Goode, J. A.; Kendrick, K. M.	Microdialysis measurement of neurochemical changes in the mediobasal hypothalamus of ovariectomized ewes during oestrus	1994	649	282-296	Brain Res
Fabricius, M.; Jensen, L. H.; Lauritzen,	Microdialysis of interstitial amino acids during spreading	1993	612	61-69	Brain Res

Taurine					
**Authors**	**Title**	**Year**	**Vol**	**Pages**	**Journal**
M.	depression and anoxic depolarization in rat neocortex				
Fallgren, A. B.; Paulsen, R. E.	A microdialysis study in rat brain of dihydrokainate, a glutamate uptake inhibitor	1996	21	19-25	Neurochem Res
Feet, B. A.; Gilland, E.; Groenendaal, F.; Brun, N. C.; Hellstrom-Westas, L.; Hagberg, H.; Saugstad, O. D.	Cerebral excitatory amino acids and Na+/K+- ATPase activity during resuscitation of severely hypoxic newborn piglets	1998	87	889-895	Acta Paediatr
Fordahl, S. C.; Anderson, J. G.; Cooney, P. T.; Weaver, T. L.; Colyer, C. L.; Erikson, K. M.	Manganese exposure inhibits the clearance of extracellular GABA and influences taurine homeostasis in the striatum of developing rats	2010	31	639-646	Neurotoxicology
Foster, S. B.; Tang, H.; Miller, K. E.; Dryhurst, G.	Increased extracellular glutamate evoked by 1-methyl-4-phenylpyridinium [MPP(+)] in the rat striatum is not essential for dopaminergic neurotoxicity and is not derived from released glutathione	2005	7	251-263	Neurotox Res
Fraser, M.; Bennet, L.; Van Zijl, P. L.; Mocatta, T. J.; Williams, C. E.; Gluckman, P. D.; Winterbourn, C. C.; Gunn, A. J.	Extracellular amino acids and lipid peroxidation products in periventricular white matter during and after cerebral ischemia in preterm fetal sheep	2008	105	2214-2223	J Neurochem
Freinbichler, W.; Colivicchi, M. A.; Fattori, M.; Ballini, C.; Tipton, K. F.; Linert, W.; Della Corte, L.	Validation of a robust and sensitive method for detecting hydroxyl radical formation together with evoked neurotransmitter release in brain microdialysis	2008	105	738-749	J Neurochem
Fujikawa, D. G.	Effects of N-methyl-D-aspartate-receptor blockade on high-potassium-induced neuronal death and glutamate release	1997	226	25-28	Neurosci Lett
Fujikawa, D. G.; Kim, J. S.; Daniels, A. H.; Alcaraz, A. F.; Sohn, T. B.	In vivo elevation of extracellular potassium in the rat amygdala increases extracellular glutamate and aspartate and damages neurons	1996	74	695-706	Neuroscience
Fujitani, T.; Adachi, N.; Miyazaki, H.; Liu, K.; Nakamura, Y.; Kataoka, K.; Arai, T.	Lidocaine protects hippocampal neurons against ischemic damage by preventing increase of extracellular excitatory amino acids: a microdialysis study in Mongolian gerbils	1994	179	91-94	Neurosci Lett
Galeffi, F.; Bianchi, L.; Bolam, J. P.; Della Corte, L.	The effect of 6-hydroxydopamine lesions on the release of amino acids in the direct and indirect pathways of the basal ganglia: a dual microdialysis probe analysis	2003	18	856-868	Eur J Neurosci

Taurine					
**Authors**	**Title**	**Year**	**Vol**	**Pages**	**Journal**
Garcia Dopico, J.; Perdomo Diaz, J.; Alonso, T. J.; Gonzalez Hernandez, T.; Castro Fuentes, R.; Rodriguez Diaz, M.	Extracellular taurine in the substantia nigra: taurine-glutamate interaction	2004	76	528-538	J Neurosci Res
Ge, J.; Long, S. K.; Kilpatrick, I. C.	Preferential blockade of cholecystokinin-8S-induced increases in aspartate and glutamate levels by the CCK(B) receptor antagonist, L-365,260, in rat brain	1998	345	163-170	Eur J Pharmacol
Geddes, J. W.; Chang, N. G.; Ackley, D. C.; Soultanian, N. S.; McGillis, J. P.; Yokel, R. A.	Postmortem elevation in extracellular glutamate in the rat hippocampus when brain temperature is maintained at physiological levels: implications for the use of human brain autopsy tissues	1999	831	104-112	Brain Res
Gemba, T.; Matsunaga, K.; Ueda, M.	Changes in extracellular concentration of amino acids in the hippocampus during cerebral ischemia in stroke-prone SHR, stroke-resistant SHR and normotensive rats	1992	135	184-188	Neurosci Lett
Gemba, T.; Ninomiya, M.; Matsunaga, K.; Ueda, M.	Effects of a novel calcium antagonist, S-(+)-methyl-4,7-dihydro-3-isobutyl-6- methyl-4-(3-nitrophenyl)thieno[2,3-b]pyridine-5-carboxylate (S-312-d), on ischemic amino acid release and neuronal injury in stroke-prone spontaneously hypertensive rats	1993	265	463-467	J Pharmacol Exp Ther
Gobert, A.; Rivet, J. M.; Billiras, R.; Parsons, F.; Millan, M. J.	Simultaneous quantification of D- vs. L-serine, taurine, kynurenate, phosphoethanolamine and diverse amino acids in frontocortical dialysates of freely-moving rats: differential modulation by N-methyl-D-aspartate (NMDA) and other pharmacological agents	2011	202	143-157	J Neurosci Methods
Goda, H.; Ooboshi, H.; Nakane, H.; Ibayashi, S.; Sadoshima, S.; Fujishima, M.	Modulation of ischemia-evoked release of excitatory and inhibitory amino acids by adenosine A1 receptor agonist	1998	357	149-155	Eur J Pharmacol
Goldsmith, J. D.; Kujawa, S. G.; McLaren, J. D.; Bledsoe Jr, S. C.	In vivo release of neuroactive amino acids from the inferior colliculus of the guinea pig using brain microdialysis	1995	83	80-88	Hear Res
Gordon, K. E.; Simpson, J.; Statman, D.; Silverstein, F. S.	Effects of perinatal stroke on striatal amino acid efflux in rats studied with in vivo microdialysis	1991	22	928-932	Stroke
Grabb, M. C.; Sciotti, V. M.; Gidday, J. M.; Cohen, S. A.; van Wylen, D. G.	Neurochemical and morphological responses to acutely and chronically implanted brain microdialysis probes	1998	82	25-34	J Neurosci Methods

Taurine					
**Authors**	**Title**	**Year**	**Vol**	**Pages**	**Journal**
Graham, S. H.; Shimizu, H.; Newman, A.; Weinstein, P.; Faden, A. I.	Opioid receptor antagonist nalmefene stereospecifically inhibits glutamate release during global cerebral ischemia	1993	632	346-350	Brain Res
Graham, S. H.; Shiraishi, K.; Panter, S. S.; Simon, R. P.; Faden, A. I.	Changes in extracellular amino acid neurotransmitters produced by focal cerebral ischemia	1990	110	124-130	Neurosci Lett
Gruss, M.; Bock, J.; Braun, K.	Haloperidol impairs auditory filial imprinting and modulates monoaminergic neurotransmission in an imprinting-relevant forebrain area of the domestic chick	2003	87	686-696	J Neurochem
Gruss, M.; Braun, K.	Stimulus-evoked increase of glutamate in the mediorostral neostriatum/hyperstriatum ventrale of domestic chick after auditory filial imprinting: an in vivo microdialysis study	1996	66	1167-1173	J Neurochem
Gruss, M.; Bredenkotter, M.; Braun, K.	N-methyl-D-aspartate receptor-mediated modulation of monoaminergic metabolites and amino acids in the chick forebrain: an in vivo microdialysis and electrophysiology study	1999	40	116-135	J Neurobiol
Guerra-Romero, L.; Tureen, J. H.; Fournier, M. A.; Makrides, V.; Tauber, M. G.	Amino acids in cerebrospinal and brain interstitial fluid in experimental pneumococcal meningitis	1993	33	510-513	Pediatr Res
Guevara-Guzman, R.; Barrera-Mera, B.; De La Riva, C.; Kendrick, K. M.	Release of classical transmitters and nitric oxide in the rat olfactory bulb, evoked by vaginocervical stimulation and potassium, varies with the oestrus cycle	2000	12	80-88	Eur J Neurosci
Guevara-Guzman, R.; Emson, P. C.; Kendrick, K. M.	Modulation of in vivo striatal transmitter release by nitric oxide and cyclic GMP	1994	62	807-810	J Neurochem
Hada, J.; Kaku, T.; Jiang, M. H.; Morimoto, K.; Hayashi, Y.; Nagai, K.	Sodium nitroprusside-induced seizure and taurine release from rat hippocampus	2000	19	547-559	Amino Acids
Hada, J.; Kaku, T.; Morimoto, K.; Hayashi, Y.; Nagai, K.	Adenosine transport inhibitors enhance high K(+)-evoked taurine release from rat hippocampus	1996	305	101-107	Eur J Pharmacol
Hada, J.; Kaku, T.; Morimoto, K.; Hayashi, Y.; Nagai, K.	Activation of adenosine A2 receptors enhances high K(+)-evoked taurine release from rat hippocampus: a microdialysis study	1998	15	43-52	Amino Acids
Hamann, M.; Sohr, R.; Morgenstern, R.; Richter, A.	Extracellular amino acid levels in the striatum of the dt(sz) mutant, a model of paroxysmal dystonia	2008	157	188-195	Neuroscience

Taurine					
**Authors**	**Title**	**Year**	**Vol**	**Pages**	**Journal**
Han, J.; Wan, H. T.; Yang, J. H.; Zhang, Y. Y.; Ge, L. J.; Bie, X. D.	Effect of ligustrazine on levels of amino acid neurotransmitters in rat striatum after cerebral ischemia-reperfusion injury	2014	16	1060-1067	J Asian Nat Prod Res
Harkany, T.; Abraham, I.; Laskay, G.; Timmerman, W.; Jost, K.; Zarandi, M.; Penke, B.; Nyakas, C.; Luiten, P. G.	Propionyl-IIGL tetrapeptide antagonizes beta-amyloid excitotoxicity in rat nucleus basalis	1999	10	1693-1698	Neuroreport
Harkany, T.; Abraham, I.; Timmerman, W.; Laskay, G.; Toth, B.; Sasvari, M.; Konya, C.; Sebens, J. B.; Korf, J.; Nyakas, C.; Zarandi, M.; Soos, K.; Penke, B.; Luiten, P. G.	beta-amyloid neurotoxicity is mediated by a glutamate-triggered excitotoxic cascade in rat nucleus basalis	2000	12	2735-2745	Eur J Neurosci
Haskew-Layton, R. E.; Rudkouskaya, A.; Jin, Y.; Feustel, P. J.; Kimelberg, H. K.; Mongin, A. A.	Two distinct modes of hypoosmotic medium-induced release of excitatory amino acids and taurine in the rat brain in vivo	2008	3	e3543	PLoS One
Hathway, G. J.; Emson, P. C.; Humphrey, P. P.; Kendrick, K. M.	Somatostatin potently stimulates in vivo striatal dopamine and gamma-aminobutyric acid release by a glutamate-dependent action	1998	70	1740-1749	J Neurochem
Hazell, A. S.; Butterworth, R. F.; Hakim, A. M.	Cerebral vulnerability is associated with selective increase in extracellular glutamate concentration in experimental thiamine deficiency	1993	61	1155-1158	J Neurochem
Hehre, D. A.; Devia, C. J.; Bancalari, E.; Suguihara, C.	Brainstem amino acid neurotransmitters and ventilatory response to hypoxia in piglets	2008	63	46-50	Pediatr Res
Hemmati, P.; Shilliam, C. S.; Hughes, Z. A.; Shah, A. J.; Roberts, J. C.; Atkins, A. R.; Hunter, A. J.; Heidbreder, C. A.	In vivo characterization of basal amino acid levels in subregions of the rat nucleus accumbens: effect of a dopamine D(3)/D(2) agonist	2001	39	199-208	Neurochem Int
Heron, A.; Lasbennes, F.; Seylaz, J.	Adenosine modulation of amino acid release in rat hippocampus during ischemia and veratridine depolarization	1993	608	27-32	Brain Res
Herranz, A. S.; Solis, J. M.; Herreras, O.; Menendez, N.; Ambrosio, E.; Orensanz, L. M.; Martin del Rio, R.	The epileptogenic action of the taurine analogue guanidinoethane sulfonate may be caused by a blockade of GABA receptors	1990	26	98-104	J Neurosci Res
Herz, R. C.; Gaillard, P. J.; de Wildt, D. J.; Versteeg, D. H.	Differences in striatal extracellular amino acid concentrations between Wistar and Fischer 344 rats	1996	715	163-171	Brain Res

Taurine					
**Authors**	**Title**	**Year**	**Vol**	**Pages**	**Journal**
	after middle cerebral artery occlusion				
Hilgier, W.; Zielinska, M.; Borkowska, H. D.; Gadamski, R.; Walski, M.; Oja, S. S.; Saransaari, P.; Albrecht, J.	Changes in the extracellular profiles of neuroactive amino acids in the rat striatum at the asymptomatic stage of hepatic failure	1999	56	76-84	J Neurosci Res
Hillered, L.; Hallstrom, A.; Segersvard, S.; Persson, L.; Ungerstedt, U.	Dynamics of extracellular metabolites in the striatum after middle cerebral artery occlusion in the rat monitored by intracerebral microdialysis	1989	9	607-616	J Cereb Blood Flow Metab
Hoop, B.; Beagle, J. L.; Maher, T. J.; Kazemi, H.	Brainstem amino acid neurotransmitters and hypoxic ventilatory response	1999	118	117-129	Respir Physiol
Horn, T.; Bauce, L.; Landgraf, R.; Pittman, Q. J.	Microdialysis with high NaCl causes central release of amino acids and dopamine	1995	64	1632-1644	J Neurochem
Horn, T.; Smith, P. M.; McLaughlin, B. E.; Bauce, L.; Marks, G. S.; Pittman, Q. J.; Ferguson, A. V.	Nitric oxide actions in paraventricular nucleus: cardiovascular and neurochemical implications	1994	266	R306-R313	Am J Physiol
Hu, M.; Watson, C. J.; Kennedy, R. T.; Becker, J. B.	Estradiol attenuates the K+-induced increase in extracellular GABA in rat striatum	2006	59	122-124	Synapse
Huo, T.; Zhang, Y.; Li, W.; Yang, H.; Jiang, H.; Sun, G.	Effect of realgar on extracellular amino acid neurotransmitters in hippocampal CA1 region determined by online microdialysis-dansyl chloride derivatization-high-performance liquid chromatography and fluorescence detection	2014	28	1254-1262	Biomed Chromatogr
Hwa, L. S.; Nathanson, A. J.; Shimamoto, A.; Tayeh, J. K.; Wilens, A. R.; Holly, E. N.; Newman, E. L.; DeBold, J. F.; Miczek, K. A.	Aggression and increased glutamate in the mPFC during withdrawal from intermittent alcohol in outbred mice	2015	232	2889-2902	Psychopharmacology (Berl)
Hylland, P.; Nilsson, G. E.	Extracellular levels of amino acid neurotransmitters during anoxia and forced energy deficiency in crucian carp brain	1999	823	49-58	Brain Res
Hylland, P.; Nilsson, G. E.; Johansson, D.	Anoxic brain failure in an ectothermic vertebrate: release of amino acids and K+ in rainbow trout thalamus	1995	269	R1077-R1084	Am J Physiol
Hyzinski-Garcia, M. C.; Vincent, M. Y.; Haskew-Layton, R. E.; Dohare, P.; Keller Jr, R. W.; Mongin, A. A.	Hypo-osmotic swelling modifies glutamate-glutamine cycle in the cerebral cortex and in astrocyte cultures	2011	118	140-152	J Neurochem

Taurine					
**Authors**	**Title**	**Year**	**Vol**	**Pages**	**Journal**
Iijima, T.; Iwao, Y.; Sankawa, H.	Amino acid release during spreading depression in a flow-compromised cortical area	1999	818	553-555	Brain Res
Iino, T.; Katsura, M.; Kuriyama, K.	Protective effect of vinconate on ischemia-induced neuronal damage in the rat hippocampus	1992	224	117-124	Eur J Pharmacol
Ishiwata, S.; Umino, A.; Balu, D. T.; Coyle, J. T.; Nishikawa, T.	Neuronal serine racemase regulates extracellular D-serine levels in the adult mouse hippocampus	2015	122	1099-1103	J Neural Transm (Vienna)
Itoh, T.; Saito, T.; Fujimura, M.; Watanabe, S.; Saito, K.	Restraint stress-induced changes in endogenous zinc release from the rat hippocampus	1993	618	318-322	Brain Res
Jacobson, I.; Hagberg, H.; Sandberg, M.; Hamberger, A.	Ouabain-induced changes in extracellular aspartate, glutamate and GABA levels in the rabbit olfactory bulb in vivo	1986	64	211-215	Neurosci Lett
Jacobson, I.; Sandberg, M.; Hamberger, A.	Mass transfer in brain dialysis devices--a new method for the estimation of extracellular amino acids concentration	1985	15	263-268	J Neurosci Methods
Jacobsson, S. O.; Cassel, G. E.; Karlsson, B. M.; Sellstrom, A.; Persson, S. A.	Release of dopamine, GABA and EAA in rats during intrastriatal perfusion with kainic acid, NMDA and soman: a comparative microdialysis study	1997	71	756-765	Arch Toxicol
Jiao, Y.; Wang, D. Q.; Wu, Z. E.; Fan, B.; Peng, J.; Wang, X. Y.	The effect of extracellular amino acid neurotransmitters in striatum of rats by intracerebral perfusion of 6-OHDA and Madopar intervene. [Chinese]	2009	25	1055-1059	Zhongguo yao li xue tong bao
Jimenez-Diaz, L.; Gruart, A.; Minano, F. J.; Delgado-Garcia, J. M.	An experimental study of posterior interpositus involvement in the genesis and control of conditioned eyelid responses	2002	978	106-118	Ann N Y Acad Sci
Kapoor, V.; Minson, J.; Chalmers, J.	Ventral medulla stimulation increases blood pressure and spinal cord amino acid release	1992	3	55-58	Neuroreport
Kapoor, V.; Nakahara, D.; Blood, R. J.; Chalmers, J. P.	Preferential release of neuroactive amino acids from the ventrolateral medulla of the rat in vivo as measured by microdialysis	1990	37	187-191	Neuroscience
Kashkin, V. A.; De Witte, P.	Nicotine increases microdialysate brain amino acid concentrations and induces conditioned place preference	2005	15	625-632	Eur Neuropsychopharmacol
Kasper, J. M.; Booth, R. G.; Peris, J.	Serotonin-2C receptor agonists decrease potassium-stimulated GABA release in the nucleus accumbens	2015	69	78-85	Synapse

Taurine					
**Authors**	**Title**	**Year**	**Vol**	**Pages**	**Journal**
Katoh, H.; Sima, K.; Nawashiro, H.; Wada, K.; Chigasaki, H.	The effect of MK-801 on extracellular neuroactive amino acids in hippocampus after closed head injury followed by hypoxia in rats	1997	758	153-162	Brain Res
Keck, M. E.; Sillaber, I.; Ebner, K.; Welt, T.; Toschi, N.; Kaehler, S. T.; Singewald, N.; Philippu, A.; Elbel, G. K.; Wotjak, C. T.; Holsboer, F.; Landgraf, R.; Engelmann, M.	Acute transcranial magnetic stimulation of frontal brain regions selectively modulates the release of vasopressin, biogenic amines and amino acids in the rat brain	2000	12	3713-3720	Eur J Neurosci
Kekesi, K. A.; Dobolyi, A.; Salfay, O.; Nyitrai, G.; Juhasz, G.	Slow wave sleep is accompanied by release of certain amino acids in the thalamus of cats	1997	8	1183-1186	Neuroreport
Kekesi, K. A.; Szilagyi, N.; Nyitrai, G.; Dobolyi, A.; Skuban, N.; Kardos, J.	Persistent depolarization and Glu uptake inhibition operate distinct osmoregulatory mechanisms in the mammalian brain	2000	37	171-178	Neurochem Int
Kendrick, K. M.; Guevara-Guzman, R.; Zorrilla, J.; Hinton, M. R.; Broad, K. D.; Mimmack, M.; Ohkura, S.	Formation of olfactory memories mediated by nitric oxide	1997	388	670-674	Nature
Kendrick, K. M.; Hinton, M. R.	Microdialysis sampling of in vivo neurotransmitter release in the brain of the conscious sheep: Measurement of acetylcholine, amino acid and monoamine concentrations by high performance liquid chromatography (HPLC)	1992	446	66P	J Physiol
Kendrick, K. M.; Keverne, E. B.; Hinton, M. R.; Goode, J. A.	Oxytocin, amino acid and monoamine release in the region of the medial preoptic area and bed nucleus of the stria terminalis of the sheep during parturition and suckling	1992	569	199-209	Brain Res
Klinker, C. C.; Bowser, M. T.	4-fluoro-7-nitro-2,1,3-benzoxadiazole as a fluorogenic labeling reagent for the in vivo analysis of amino acid neurotransmitters using online microdialysis-capillary electrophoresis	2007	79	8747-8754	Anal Chem
Koetzner, L.; Hua, X. Y.; Lai, J.; Porreca, F.; Yaksh, T.	Nonopioid actions of intrathecal dynorphin evoke spinal excitatory amino acid and prostaglandin E2 release mediated by cyclooxygenase-1 and -2	2004	24	1451-1458	J Neurosci
Koizumi, H.; Fujisawa, H.; Ito, H.;	Effects of mild hypothermia on cerebral blood flow-	1997	747	304-312	Brain Res

Taurine					
**Authors**	**Title**	**Year**	**Vol**	**Pages**	**Journal**
Maekawa, T.; Di, X.; Bullock, R.	independent changes in cortical extracellular levels of amino acids following contusion trauma in the rat				
Kusaka, T.; Matsuura, S.; Fujikawa, Y.; Okubo, K.; Kawada, K.; Namba, M.; Okada, H.; Imai, T.; Isobe, K.; Itoh, S.	Relationship between cerebral interstitial levels of amino acids and phosphorylation potential during secondary energy failure in hypoxic-ischemic newborn piglets	2004	55	273-279	Pediatr Res
La Bella, V.; Piccoli, F.	Differential effect of beta-N-oxalylamino-L-alanine, the Lathyrus sativus neurotoxin, and (+/-)-alpha-amino-3-hydroxy-5-methylisoxazole-4-propionate on the excitatory amino acid and taurine levels in the brain of freely moving rats	2000	36	523-530	Neurochem Int
Lada, M. W.; Kennedy, R. T.	Quantitative in vivo monitoring of primary amines in rat caudate nucleus using microdialysis coupled by a flow-gated interface to capillary electrophoresis with laser-induced fluorescence detection	1996	68	2790-2797	Anal Chem
Lallemand, F.; De Witte, P.	Taurine concentration in the brain and in the plasma following intraperitoneal injections	2004	26	111-116	Amino Acids
Lallemand, F.; Ward, R. J.; De Witte, P.; Verbanck, P.	Binge drinking +/- chronic nicotine administration alters extracellular glutamate and arginine levels in the nucleus accumbens of adult male and female Wistar rats	2011	46	373-382	Alcohol Alcohol
Lallemand, F.; Ward, R. J.; De Witte, P.; Verbank, P.	Binge drinking and/or chronic nicotine administration alters extracellular glutamate and arginine levels in the nucleus accumbens of adult male and female wistar rats	2011	46	i44	Alcohol Alcohol
Lallement, G.; Carpentier, P.; Collet, A.; Pernot-Marino, I.; Baubichon, D.; Blanchet, G.	Effects of soman-induced seizures on different extracellular amino acid levels and on glutamate uptake in rat hippocampus	1991	563	234-240	Brain Res
Langlais, P. J.; Shu Xing, Zhang	Extracellular glutamate is increased in thalamus during thiamine deficiency-induced lesions and is blocked by MK-801	1993	61	2175-2182	J Neurochem
Largo, C.; Cuevas, P.; Somjen, G. G.; Martin del Rio, R.; Herreras, O.	The effect of depressing glial function in rat brain in situ on ion homeostasis, synaptic transmission, and neuron survival	1996	16	1219-1229	J Neurosci
Lazarewicz, J. W.; Puka-Sundvall, M.;	Differential effects of N-methyl-D-aspartate on Ca2+	1995	13	685-704	Int J Dev Neurosci

Taurine					
**Authors**	**Title**	**Year**	**Vol**	**Pages**	**Journal**
Gadamski, R.; Sandberg, M.; Hagberg, H.	homeostasis in developing and adult rat striatum: in vivo microdialysis approach				
Lazarewicz, J. W.; Salinska, E.; Puka, M.	Glycine enhances extracellular 45Ca2+ response to NMDA application investigated with microdialysis of rabbit hippocampus in vivo	1992	52	83-91	Acta Neurobiol Exp (Wars)
Lee, T. F.; Jantzie, L. L.; Todd, K. G.; Cheung, P. Y.	Postresuscitation N-acetylcysteine treatment reduces cerebral hydrogen peroxide in the hypoxic piglet brain	2008	34	190-197	Intensive Care Med
Lehmann, A.	Alterations in hippocampal extracellular amino acids and purine catabolites during limbic seizures induced by folate injections into the rabbit amygdala	1987	22	573-578	Neuroscience
Lehmann, A.	Evidence for a direct action of N-methylaspartate on non-neuronal cells	1987	411	95-101	Brain Res
Lehmann, A.	Effects of microdialysis-perfusion with anisoosmotic media on extracellular amino acids in the rat hippocampus and skeletal muscle	1989	53	525-535	J Neurochem
Lehmann, A.; Carlstrom, C.; Nagelhus, E. A.; Ottersen, O. P.	Elevation of taurine in hippocampal extracellular fluid and cerebrospinal fluid of acutely hypoosmotic rats: contribution by influx from blood?	1991	56	690-697	J Neurochem
Lehmann, A.; Hagberg, H.; Huxtable, R. J.; Sandberg, M.	Reduction of brain taurine: Effects on neurotoxic and metabolic actions of kainate	1987	10	265-274	Neurochem Int
Lehmann, A.; Lazarewicz, J. W.; Zeise, M.	N-Methylaspartate-evoked liberation of taurine and phosphoethanolamine in vivo: site of release	1985	45	1172-1177	J Neurochem
Lehmann, A.; Sandberg, M.	In vivo substitution of choline for sodium evokes a selective osmoinsensitive increase of extracellular taurine in the rat hippocampus	1990	54	126-129	J Neurochem
Lekieffre, D.; Callebert, J.; Plotkine, M.; Boulu, R. G.	Concomitant increases in the extracellular concentrations of excitatory and inhibitory amino acids in the rat hippocampus during forebrain ischemia	1992	137	78-82	Neurosci Lett
Lekieffre, D.; Meldrum, B. S.	The pyrimidine-derivative, BW1003C87, protects CA1 and striatal neurons following transient severe forebrain ischaemia in rats. A microdialysis and histological study	1993	56	93-99	Neuroscience
Lerma, J.; Herranz, A. S.; Herreras, O.	In vivo determination of extracellular concentration of amino acids in the rat hippocampus. A method based on	1986	384	145-155	Brain Research

Taurine					
**Authors**	**Title**	**Year**	**Vol**	**Pages**	**Journal**
	brain dialysis and computerized analysis				
Lerma, J.; Herranz, A. S.; Herreras, O.; Munoz, D.; Solis, J. M.; del Rio, R. M.; Delgado, J. M.	gamma-Aminobutyric acid greatly increases the in vivo extracellular taurine in the rat hippocampus	1985	44	983-986	J Neurochem
Lerma, J.; Herreras, O.; Herranz, A. S.; Munoz, D.; del Rio, R. M.	In vivo effects of nipecotic acid on levels of extracellular GABA and taurine, and hippocampal excitability	1984	23	595-598	Neuropharmacology
Leung, L. Y.; Tong, K. Y.; Zhang, S. M.; Zeng, X. H.; Zhang, K. P.; Zheng, X. X.	Neurochemical effects of exercise and neuromuscular electrical stimulation on brain after stroke: a microdialysis study using rat model	2006	397	135-139	Neurosci Lett
Li, H.; Li, C.; Yan, Z. Y.; Yang, J.; Chen, H.	Simultaneous monitoring multiple neurotransmitters and neuromodulators during cerebral ischemia/reperfusion in rats by microdialysis and capillary electrophoresis	2010	189	162-168	J Neurosci Methods
Li, H.; Yan, Z. Y.	Analysis of amino acid neurotransmitters in hypothalamus of rats during cerebral ischemia-reperfusion by microdialysis and capillary electrophoresis	2010	24	1185-1192	Biomed Chromatogr
Li, P. A.; He, Q. P.; Miyashita, H.; Howllet, W.; Siesjo, B. K.; Shuaib, A.	Hypothermia ameliorates ischemic brain damage and suppresses the release of extracellular amino acids in both normo- and hyperglycemic subjects	1999	158	242-253	Exp Neurol
Li, T. F.; Xue, W.; Li, M.; Shi, A. X.; Li, K. X.	Establishment of an LC-MS/MS method to determine content of six amino acids in rat brain dialysate. [Chinese]	2015	24	1659-1664	Zhongguo xin yao za zhi
Li, X. L.; An, Y.; Jin, Q. H.; Kim, M. S.; Park, B. R.; Jin, Y. Z.	Changes of some amino acid concentrations in the medial vestibular nucleus of conscious rats following acute hypotension	2010	447	11-14	Neurosci Lett
Li, Z. P.; Zhang, X. Y.; Lu, X.; Zhong, M. K.; Ji, Y. H.	Dynamic release of amino acid transmitters induced by valproate in PTZ-kindled epileptic rat hippocampus	2004	44	263-270	Neurochem Int
Li, Z. Q.; Yamamoto, Y.; Morimoto, T.; Ono, J.; Okada, S.; Yamatodani, A.	The effect of pentylenetetrazole-kindling on the extracellular glutamate and taurine levels in the frontal cortex of rats	2000	282	117-119	Neurosci Lett
Li, Z.; Zharikova, A.; Vaughan, C. H.; Bastian, J.; Zandy, S.; Esperon, L.; Axman, E.; Rowland, N. E.; Peris, J.	Intermittent high-dose ethanol exposures increase motivation for operant ethanol self-administration: possible neurochemical mechanism	2010	1310	142-153	Brain Res

**Taurine**					
**Authors**	**Title**	**Year**	**Vol**	**Pages**	**Journal**
Lido, H. H.; Marston, H.; Ericson, M.; Soderpalm, B.	The glycine reuptake inhibitor Org24598 and acamprosate reduce ethanol intake in the rat; tolerance development to acamprosate but not to Org24598	2012	17	897-907	Addict Biol
Lido, H. H.; Stomberg, R.; Fagerberg, A.; Ericson, M.; Soderpalm, B.	The glycine reuptake inhibitor org 25935 interacts with basal and ethanol-induced dopamine release in rat nucleus accumbens	2009	33	1151-1157	Alcohol Clin Exp Res
Liu, H. G.; Yang, A. C.; Meng, D. W.; Chen, N.; Zhang, J. G.	Stimulation of the anterior nucleus of the thalamus induces changes in amino acids in the hippocampi of epileptic rats	2012	1477	37-44	Brain Res
Liu, Z.; Zhang, L.; He, Q.; Liu, X.; Chukwunweike Ikechukwu, O.; Tong, L.; Guo, L.; Yang, H.; Zhang, Q.; Zhao, H.; Gu, X.	Effect of Baicalin-loaded PEGylated cationic solid lipid nanoparticles modified by OX26 antibody on regulating the levels of baicalin and amino acids during cerebral ischemia-reperfusion in rats	2015	489	131-138	Int J Pharmaceut
Lo, E. H.; Bosque-Hamilton, P.; Meng, W.	Inhibition of poly(ADP-ribose) polymerase: reduction of ischemic injury and attenuation of N-methyl-D-aspartate-induced neurotransmitter dysregulation	1998	29	830-836	Stroke
Lo, E. H.; Steinberg, G. K.; Panahian, N.; Maidment, N. T.; Newcomb, R.	Profiles of extracellular amino acid changes in focal cerebral ischaemia: effects of mild hypothermia	1993	15	281-287	Neurol Res
Lowy, M. T.; Gault, L.; Yamamoto, B. K.	Adrenalectomy attenuates stress-induced elevations in extracellular glutamate concentrations in the hippocampus	1993	61	1957-1960	J Neurochem
Luna-Munguia, H.; Meneses, A.; Pena-Ortega, F.; Gaona, A.; Rocha, L.	Effects of hippocampal high-frequency electrical stimulation in memory formation and their association with amino acid tissue content and release in normal rats	2012	22	98-105	Hippocampus
Maciejak, P.; Szyndler, J.; Turzynska, D.; Sobolewska, A.; Plaznik, A.	Kynurenic acid: A new effector of valproate action?	2011	63	1569-1573	Pharmacol Rep
Maciejak, P.; Szyndler, J.; Turzynska, D.; Sobolewska, A.; Taracha, E.; Skorzewska, A.; Lehner, M.; Bidzinski, A.; Hamed, A.; Wislowska-Stanek, A.; Plaznik, A.	The effects of group III mGluR ligands on pentylenetetrazol-induced kindling of seizures and hippocampal amino acids concentration	2009	1282	20-27	Brain Res
Marquez de Prado, B.; Castaneda, T. R.;	Melatonin disrupts circadian rhythms of glutamate and	2000	29	209-216	J Pineal Res

Taurine					
**Authors**	**Title**	**Year**	**Vol**	**Pages**	**Journal**
Galindo, A.; del Arco, A.; Segovia, G.; Reiter, R. J.; Mora, F.	GABA in the neostriatum of the aware rat: a microdialysis study				
Marsala, M.; Sorkin, L. S.; Yaksh, T. L.	Transient spinal ischemia in rat: characterization of spinal cord blood flow, extracellular amino acid release, and concurrent histopathological damage	1994	14	604-614	J Cereb Blood Flow Metab
Massieu, L.; Gomez-Roman, N.; Montiel, T.	In vivo potentiation of glutamate-mediated neuronal damage after chronic administration of the glycolysis inhibitor iodoacetate	2000	165	257-267	Exp Neurol
Massieu, L.; Morales-Villagran, A.; Tapia, R.	Accumulation of extracellular glutamate by inhibition of its uptake is not sufficient for inducing neuronal damage: an in vivo microdialysis study	1995	64	2262-2272	J Neurochem
Massieu, L.; Tapia, R.	Glutamate uptake impairment and neuronal damage in young and aged rats in vivo	1997	69	1151-1160	J Neurochem
Matsumoto, K.; Lo, E. H.; Pierce, A. R.; Halpern, E. F.; Newcomb, R.	Secondary elevation of extracellular neurotransmitter amino acids in the reperfusion phase following focal cerebral ischemia	1996	16	114-124	J Cereb Blood Flow Metab
Matsumoto, K.; Ueda, S.; Hashimoto, T.; Kuriyama, K.	Ischemic neuronal injury in the rat hippocampus following transient forebrain ischemia: evaluation using in vivo microdialysis	1991	543	236-242	Brain Res
McKenzie, J. A.; Watson, C. J.; Rostand, R. D.; German, I.; Witowski, S. R.; Kennedy, R. T.	Automated capillary liquid chromatography for simultaneous determination of neuroactive amines and amino acids	2002	962	105-115	J Chromatogr A
Medina-Ceja, L.; Morales-Villagran, A.; Tapia, R.	Action of 4-aminopyridine on extracellular amino acids in hippocampus and entorhinal cortex: a dual microdialysis and electroencehalographic study in awake rats	2000	53	255-262	Brain Res Bull
Melani, A.; Pantoni, L.; Bordoni, F.; Gianfriddo, M.; Bianchi, L.; Vannucchi, M. G.; Bertorelli, R.; Monopoli, A.; Pedata, F.	The selective A2A receptor antagonist SCH 58261 reduces striatal transmitter outflow, turning behavior and ischemic brain damage induced by permanent focal ischemia in the rat	2003	959	243-250	Brain Res
Melani, A.; Pantoni, L.; Corsi, C.; Bianchi, L.; Monopoli, A.; Bertorelli, R.; Pepeu, G.; Pedata, F.	Striatal outflow of adenosine, excitatory amino acids, gamma-aminobutyric acid, and taurine in awake freely moving rats after middle cerebral artery occlusion: correlations with neurological deficit and	1999	30	2448-2455	Stroke

Taurine					
**Authors**	**Title**	**Year**	**Vol**	**Pages**	**Journal**
	histopathological damage				
Melon, C.; Chassain, C.; Bielicki, G.; Renou, J. P.; Kerkerian-Le Goff, L.; Salin, P.; Durif, F.	Progressive brain metabolic changes under deep brain stimulation of subthalamic nucleus in parkinsonian rats	2015	132	703-712	J Neurochem
Mena, F. V.; Baab, P. J.; Zielke, C. L.; Huang, Y.; Zielke, H. R.	Formation of extracellular glutamate from glutamine: exclusion of pyroglutamate as an intermediate	2005	1052	88-96	Brain Res
Mena, F. V.; Baab, P. J.; Zielke, C. L.; Zielke, H. R.	In vivo glutamine hydrolysis in the formation of extracellular glutamate in the injured rat brain	2000	60	632-641	J Neurosci Res
Menendez, N.; Solis, J. M.; Herreras, O.; Sanchez Herranz, A.; Martin del Rio, R.	Role of endogenous taurine on the glutamate analogue-induced neurotoxicity in the rat hippocampus in vivo	1990	55	714-717	J Neurochem
Michalak, A.; Rose, C.; Butterworth, J.; Butterworth, R. F.	Neuroactive amino acids and glutamate (NMDA) receptors in frontal cortex of rats with experimental acute liver failure	1996	24	908-913	Hepatology
Millan, M. H.; Chapman, A. G.; Meldrum, B. S.	Contrasting effects of D- and L-(E)-4-(3-phosphono-2-propenyl)piperazine-2-carboxylic acid as anticonvulsants and as inhibitors of potassium-evoked increases in hippocampal extracellular glutamate and aspartate levels in freely moving rats	1994	62	217-222	J Neurochem
Millan, M. H.; Chapman, A. G.; Meldrum, B. S.	Extracellular amino acid levels in hippocampus during pilocarpine-induced seizures	1993	14	139-148	Epilepsy Res
Millan, M. H.; Chapman, A. G.; Meldrum, B. S.	Dual inhibitory action of enadoline (CI977) on release of amino acids in the rat hippocampus	1995	279	75-81	Eur J Pharmacol
Miyamoto, K. J.; Miyamoto, M. R.; Miyamoto, T. A.	Systemically administered taurine. Part I. Central nervous system effects	2006	583	323-329	Adv Exp Med Biol
Miyamoto, T. A.; Miyamoto, K. J.	Does adenosine release taurine in the A1-receptor-rich hippocampus?	1999	13	94-98	J Anesth
Miyashita, K.; Nakajima, T.; Ishikawa, A.; Miyatake, T.	An adenosine uptake blocker, propentofylline, reduces glutamate release in gerbil hippocampus following transient forebrain ischemia	1992	17	147-150	Neurochem Res
Molchanova, S. M.; Oja, S. S.; Saransaari, P.	Mechanisms of enhanced taurine release under Ca2+ depletion	2005	47	343-349	Neurochem Int
Molchanova, S.; Oja, S. S.; Saransaari, P.	Characteristics of basal taurine release in the rat striatum measured by microdialysis	2004	27	261-268	Amino Acids

Taurine					
**Authors**	**Title**	**Year**	**Vol**	**Pages**	**Journal**
Montalbetti, L.	Aminoacid recovery via microdialysis and photoinduced focal cerebral ischemia in brain cortex of rats	1995	192	153-156	Neurosci Lett
Montiel, T.; Camacho, A.; Estrada-Sanchez, A. M.; Massieu, L.	Differential effects of the substrate inhibitor l-trans-pyrrolidine-2,4-dicarboxylate (PDC) and the non-substrate inhibitor DL-threo-beta-benzyloxyaspartate (DL-TBOA) of glutamate transporters on neuronal damage and extracellular amino acid levels in rat brain in vivo	2005	133	667-678	Neuroscience
Morales, I.; Dopico, J. G.; Sabate, M.; Gonzalez-Hernandez, T.; Rodriguez Diaz, M.	Substantia nigra osmoregulation: Taurine and ATP involvement	2007	292	C1934-C1941	Am J Physiol
Morales-Villagran, A.; Tapia, R.	Preferential stimulation of glutamate release by 4-aminopyridine in rat striatum in vivo	1996	28	35-40	Neurochem Int
Morrone, L. A.; Rombola, L.; Pelle, C.; Corasaniti, M. T.; Zappettini, S.; Paudice, P.; Bonanno, G.; Bagetta, G.	The essential oil of bergamot enhances the levels of amino acid neurotransmitters in the hippocampus of rat: implication of monoterpene hydrocarbons	2007	55	255-262	Pharmacol Res
Munoz, M. D.; Herreras, O.; Herranz, A. S.	Effects of dihydrokainic acid of extracellular amino acids and neuronal excitability in the in vivo rat hippocampus	1987	26	1-8	Neuropharmacology
Nakane, H.; Yao, H.; Ibayashi, S.; Kitazono, T.; Ooboshi, H.; Uchimura, H.; Fujishima, M.	Protein kinase C modulates ischemia-induced amino acids release in the striatum of hypertensive rats	1998	782	290-296	Brain Res
Nakata, N.; Kato, H.; Kogure, K.	Ischemic tolerance and extracellular amino acid concentrations in gerbil hippocampus measured by intracerebral microdialysis	1994	35	247-251	Brain Res Bull
Nakata, N.; Kato, H.; Kogure, K.; DeGraba, T. J.	Effects of repeated cerebral ischemia on extracellular amino acid concentrations measured with intracerebral microdialysis in the gerbil hippocampus	1993	24	458-464	Stroke
Newcomb, R.; Palma, A.	Effects of diverse omega-conopeptides on the in vivo release of glutamic and gamma-aminobutyric acids	1994	638	95-102	Brain Res
Nilsson, P.; Hillered, L.; Ponten, U.; Ungerstedt, U.	Changes in cortical extracellular levels of energy-related metabolites and amino acids following concussive brain injury in rats	1990	10	631-637	J Cereb Blood Flow Metab
Nilsson, P.; Ronne-Engstrom, E.; Flink,	Epileptic seizure activity in the acute phase following	1994	637	227-232	Brain Res

Taurine					
**Authors**	**Title**	**Year**	**Vol**	**Pages**	**Journal**
R.; Ungerstedt, U.; Carlson, H.; Hillered, L.	cortical impact trauma in rat				
Nyitrai, G.; Emri, Z.; Crunelli, V.; Kekesi, K. A.; Dobolyi, A.; Juhasz, G.	In vivo blockade of thalamic GABA(B) receptors increases excitatory amino-acid levels	1996	318	295-300	Eur J Pharmacol
Nyitrai, G.; Kekesi, K. A.; Szilagyi, N.; Papp, A.; Juhasz, G.; Kardos, J.	Neurotoxicity of lindane and picrotoxin: neurochemical and electrophysiological correlates in the rat hippocampus in vivo	2002	27	139-145	Neurochem Res
Nyitrai, G.; Kovacs, I.; Szarics, E.; Skuban, N.; Juhasz, G.; Kardos, J.	Role of intracellular Ca(2+) stores shaping normal activity in brain	1999	57	906-915	J Neurosci Res
Nyitrai, G.; Szarics, E.; Kovacs, I.; Kekesi, K. A.; Juhasz, G.; Kardos, J.	Effect of CGP 36742 on the extracellular level of neurotransmitter amino acids in the thalamus	1999	34	391-398	Neurochem Int
Obrenovitch, T. P.; Richards, D. A.	Extracellular neurotransmitter changes in cerebral ischaemia	1995	7	1-54	Cerebrovasc Brain Metab Rev
Obrenovitch, T. P.; Urenjak, J.; Richards, D. A.; Ueda, Y.; Curzon, G.; Symon, L.	Extracellular neuroactive amino acids in the rat striatum during ischaemia: comparison between penumbral conditions and ischaemia with sustained anoxic depolarisation	1993	61	178-186	J Neurochem
Okuda, N.; Kohara, K.; Mikami, H.; Moriguchi, A.; Yamada, K.; Higaki, J.; Ogihara, T.	Effect of propranolol on central neurotransmitter release in Wistar rats analysed by brain microdialysis	1999	26	220-224	Clin Exp Pharmacol Physiol
Ooboshi, H.; Ibayashi, S.; Takano, K.; Sadoshima, S.; Kondo, A.; Uchimura, H.; Fujishima, M.	Hypothermia inhibits ischemia-induced efflux of amino acids and neuronal damage in the hippocampus of aged rats	2000	884	23-30	Brain Res
Ooboshi, H.; Sadoshima, S.; Yao, H.; Ibayashi, S.; Matsumoto, T.; Uchimura, H.; Fujishima, M.	Ischemia-induced release of amino acids in the hippocampus of aged hypertensive rats	1995	15	227-234	J Cereb Blood Flow Metab
Orwar, O.; Li, X.; Andine, P.; Bergstrom, C. M.; Hagberg, H.; Folestad, S.; Sandberg, M.	Increased intra- and extracellular concentrations of gamma-glutamylglutamate and related dipeptides in the ischemic rat striatum: involvement of glutamyl transpeptidase	1994	63	1371-1376	J Neurochem
Osborne, P. G.; Hashimoto, M.	Mammalian cerebral metabolism and amino acid neurotransmission during hibernation	2008	106	1888-1899	J Neurochem
Ovanesov, M. V.; Vogel, M. W.; Moran,	Neonatal Borna disease virus infection in rats is	2007	13	185-194	J Neurovirol

Taurine					
**Authors**	**Title**	**Year**	**Vol**	**Pages**	**Journal**
T. H.; Pletnikov, M. V.	associated with increased extracellular levels of glutamate and neurodegeneration in the striatum				
Paulsen, R. E.; Fonnum, F.	Role of glial cells for the basal and Ca2+-dependent K+-evoked release of transmitter amino acids investigated by microdialysis	1989	52	1823-1829	J Neurochem
Pena, F.; Tapia, R.	Relationships among seizures, extracellular amino acid changes, and neurodegeneration induced by 4-aminopyridine in rat hippocampus: a microdialysis and electroencephalographic study	1999	72	2006-2014	J Neurochem
Perez-Pinzon, M. A.; Nilsson, G. E.; Lutz, P. L.	Relationship between ion gradients and neurotransmitter release in the newborn rat striatum during anoxia	1993	602	228-233	Brain Res
Perry, V. L.; Young, R. S.; Aquila, W. J.; During, M. J.	Effect of experimental Escherichia coli meningitis on concentrations of excitatory and inhibitory amino acids in the rabbit brain: in vivo microdialysis study	1993	34	187-191	Pediatr Res
Phillis, J. W.; Song, D.; Guyot, L. L.; Regan, M. H.	Lactate reduces amino acid release and fuels recovery of function in the ischemic brain	1999	272	195-198	Neurosci Lett
Piepponen, T. P.; Kiianmaa, K.; Ahtee, L.	Effects of ethanol on the accumbal output of dopamine, GABA and glutamate in alcohol-tolerant and alcohol-nontolerant rats	2002	74	21-30	Pharmacol Biochem Behav
Piepponen, T. P.; Skujins, A.	Rapid and sensitive step gradient assays of glutamate, glycine, taurine and gamma-aminobutyric acid by high-performance liquid chromatography-fluorescence detection with o-phthalaldehyde-mercaptoethanol derivatization with an emphasis on microdialysis samples	2001	757	277-283	J Chromatogr B Biomed Sci Appl
Pierard, C.; Lagarde, D.; Barrere, B.; Duret, P.; Cordeiro, C.; Guezennec, C. Y.; Peres, M.	Effects of a vigilance enhancing drug, modafinil, on rat brain cortex amino acids: A microdialysis study	1997	25	51-54	Med Sci Res
Pierard, C.; Peres, M.; Satabin, P.; Guezennec, C. Y.; Lagarde, D.	Effects of GABA-transaminase inhibition on brain metabolism and amino-acid compartmentation: an in vivo study by 2D 1H-NMR spectroscopy coupled with microdialysis	1999	127	321-327	Exp Brain Res
Puka, M.; Lehmann, A.	In vivo acidosis reduces extracellular concentrations of	1994	37	641-646	J Neurosci Res

Taurine					
**Authors**	**Title**	**Year**	**Vol**	**Pages**	**Journal**
	taurine and glutamate in the rat hippocampus				
Puka-Sundvall, M.; Eriksson, P.; Nilsson, M.; Sandberg, M.; Lehmann, A.	Neurotoxicity of cysteine: interaction with glutamate	1995	705	65-70	Brain Res
Qian, T.; Chen, R.; Nakamura, M.; Furukawa, T.; Kumada, T.; Akita, T.; Kilb, W.; Luhmann, H. J.; Nakahara, D.; Fukuda, A.	Activity-dependent endogenous taurine release facilitates excitatory neurotransmission in the neocortical marginal zone of neonatal rats	2014	8	33	Front Cell Neurosci
Qu, Y.; Arckens, L.; Vandenbussche, E.; Geeraerts, S.; Vandesande, F.	Simultaneous determination of total and extracellular concentrations of the amino acid neurotransmitters in cat visual cortex by microbore liquid chromatography and electrochemical detection	1998	798	19-26	J Chromatogr A
Qu, Y.; Li, Y.; Vandenbussche, E.; Vandesande, F.; Arckens, L.	In vivo microdialysis in the visual cortex of awake cat. II: sample analysis by microbore HPLC-electrochemical detection and capillary electrophoresis-laser-induced fluorescence detection	2001	7	45-51	Brain Res Brain Res Protoc
Quertemont, E.; Dahchour, A.; Ward, R. J.; De Witte, P.	Ethanol induces taurine release in the amygdala: An in vivo microdialysis study	1999	4	47-54	Addict Biol
Quertemont, E.; de Neuville, J.; De Witte, P.	Changes in the amygdala amino acid microdialysate after conditioning with a cue associated with ethanol	1998	139	71-78	Psychopharmacology (Berl)
Quertemont, E.; Devitgh, A.; De Witte, P.	Systemic osmotic manipulations modulate ethanol-induced taurine release: a brain microdialysis study	2003	29	11-19	Alcohol
Quertemont, E.; Lallemand, F.; Colombo, G.; De Witte, P.	Taurine and ethanol preference: a microdialysis study using Sardinian alcohol-preferring and non-preferring rats	2000	10	377-383	Eur Neuropsychopharmacol
Quertemont, E.; Linotte, S.; de Witte, P.	Differential taurine responsiveness to ethanol in high- and low-alcohol sensitive rats: a brain microdialysis study	2002	444	143-150	Eur J Pharmacol
Qureshi, A. I.; Ali, Z.; Suri, M. F.; Shuaib, A.; Baker, G.; Todd, K.; Guterman, L. R.; Hopkins, L. N.	Extracellular glutamate and other amino acids in experimental intracerebral hemorrhage: an in vivo microdialysis study	2003	31	1482-1489	Crit Care Med
Rao, V. L.; Audet, R. M.; Butterworth, R. F.	Selective alterations of extracellular brain amino acids in relation to function in experimental portal-systemic encephalopathy: results of an in vivo microdialysis study	1995	65	1221-1228	J Neurochem

**Taurine**					
**Authors**	**Title**	**Year**	**Vol**	**Pages**	**Journal**
Renno, W. M.; Alkhalaf, M.; Mousa, A.; Kanaan, R. A.	A comparative study of excitatory and inhibitory amino acids in three different brainstem nuclei	2008	33	150-159	Neurochem Res
Renno, W. M.; Mullet, M. A.; Williams, F. G.; Beitz, A. J.	Construction of 1 mm microdialysis probe for amino acids dialysis in rats	1998	79	217-228	J Neurosci Methods
Renno, W. M.; Mullett, M. A.; Beitz, A. J.	Systemic morphine reduces GABA release in the lateral but not the medial portion of the midbrain periaqueductal gray of the rat	1992	594	221-232	Brain Res
Richards, D. A.; Lemos, T.; Whitton, P. S.; Bowery, N. G.	Extracellular GABA in the ventrolateral thalamus of rats exhibiting spontaneous absence epilepsy: a microdialysis study	1995	65	1674-1680	J Neurochem
Ritz, M. F.; Curin, Y.; Mendelowitsch, A.; Andriantsitohaina, R.	Acute treatment with red wine polyphenols protects from ischemia-induced excitotoxicity, energy failure and oxidative stress in rats	2008	1239	226-234	Brain Res
Ritz, M. F.; Schmidt, P.; Mendelowitsch, A.	Effects of isoflurane on glutamate and taurine releases, brain swelling and injury during transient ischemia and reperfusion	2006	116	191-202	Int J Neurosci
Ritz, M. F.; Schmidt, P.; Mendelowitsch, A.	17beta-estradiol effect on the extracellular concentration of amino acids in the glutamate excitotoxicity model in the rat	2002	27	1677-1683	Neurochem Res
Rocha, L.; Briones, M.; Ackermann, R. F.; Anton, B.; Maidment, N. T.; Evans, C. J.; Engel, J., Jr.	Pentylenetetrazol-induced kindling: early involvement of excitatory and inhibitory systems	1996	26	105-113	Epilepsy Res
Rodriguez, M. J.; Robledo, P.; Andrade, C.; Mahy, N.	In vivo co-ordinated interactions between inhibitory systems to control glutamate-mediated hippocampal excitability	2005	95	651-661	J Neurochem
Rodriguez-Navarro, J. A.; Gonzalo-Gobernado, R.; Herranz, A. S.; Gonzlez-Vigueras, J. M.; Solis, J. M.	High potassium induces taurine release by osmosensitive and osmoresistant mechanisms in the rat hippocampus in vivo	2009	87	208-217	J Neurosci Res
Runnerstam, M.; Bao, F.; Huang, Y.; Shi, J.; Gutierrez, E.; Hamberger, A.; Hansson, H. A.; Viano, D.; Haglid, K.	A new model for diffuse brain injury by rotational acceleration: II. Effects on extracellular glutamate, intracranial pressure, and neuronal apoptosis	2001	18	259-273	J Neurotrauma
Runnerstam, M.; Stephensen, H.; von Essen, C.; Nystrom, B.; Hamberger, A.	Subarachnoid blood infusion versus raised intracranial pressure: effects on the amino acid pattern in the	1999	21	404-408	Neurol Res

Taurine					
**Authors**	**Title**	**Year**	**Vol**	**Pages**	**Journal**
	extracellular fluid of the rabbit hippocampus				
Saellstroem Baum, S.; Huebner, A.; Krimphove, M.; Morgenstern, R.; Badawy, A. A.; Spies, C. D.	Nicotine stimulation on extracellular glutamate levels in the nucleus accumbens of ethanol-withdrawn rats in vivo	2006	30	1414-1421	Alcohol Clin Exp Res
Salimaki, J.; Scriba, G.; Piepponen, T. P.; Rautolahti, N.; Ahtee, L.	The effects of systemically administered taurine and N-pivaloyltaurine on striatal extracellular dopamine and taurine in freely moving rats	2003	368	134-141	Naunyn Schmiedebergs Arch Pharmacol
Salinska, E.; Ziembowicz, A.; Stafiej, A.; Zieminska, E.; Lazarewicz, J. W.	Role of Ca2+ release from ryanodine stores in generation of NMDA-induced Ca2+ signal in rabbit hippocampus	1999	85	212-219	Ross Fiziol Zh Im I M Sechenova
Samuelsson, C.; Kumlien, E.; Elfving, A.; Lindholm, D.; Ronne-Engstrom, E.	The effects of PBN (phenyl-butyl-nitrone) on GLT-1 levels and on the extracellular levels of amino acids and energy metabolites in a model of iron-induced posttraumatic epilepsy	2003	56	165-173	Epilepsy Res
Sanchez-Carbente, M. D. R.; Massieu, L.	Transient inhibition of glutamate uptake in vivo induces neurodegeneration when energy metabolism is impaired	1999	72	129-138	J Neurochem
Sandberg, M.; Butcher, S. P.; Hagberg, H.	Extracellular overflow of neuroactive amino acids during severe insulin-induced hypoglycemia: in vivo dialysis of the rat hippocampus	1986	47	174-184	J Neurochem
Schaefer, F.; Vogel, M.; Kerkhoff, G.; Woitzik, J.; Daschner, M.; Mehls, O.	Experimental uremia affects hypothalamic amino acid neurotransmitter milieu	2001	12	1218-1227	J Am Soc Nephrol
Scheller, D. K.	Extracellular taurine as a parameter to monitor cerebral insults 'on-line': time courses and mechanisms as studied in vivo	2000	483	265-272	Adv Exp Med Biol
Scheller, D. K.; De Ryck, M.; Kolb, J.; Szathmary, S.; van Reempts, J.; Clincke, G.; Tegtmeier, F.	Lubeluzole blocks increases in extracellular glutamate and taurine in the peri-infarct zone in rats	1997	338	243-251	Eur J Pharmacol
Scheller, D.; de Ryck, M.; Clincke, G.; Tegtmeier, F.	Extracellular changes of taurine in the peri-infarct zone: effect of lubeluzole	1997	70	185-187	Acta Neurochir Suppl
Scheller, D.; Korte, M.; Szathmary, S.; Tegtmeier, F.	Cerebral taurine release mechanisms in vivo: pharmacological investigations in rats using microdialysis for proof of principle	2000	25	801-807	Neurochem Res
Scheller, D.; Szathmary, S.; Kolb, J.;	Observations on the relationship between the	2000	19	571-583	Amino Acids

Taurine					
**Authors**	**Title**	**Year**	**Vol**	**Pages**	**Journal**
Tegtmeier, F.	extracellular changes of taurine and glutamate during cortical spreading depression, during ischemia, and within the area surrounding a thrombotic infarct				
Schubert, G. A.; Poli, S.; Mendelowitsch, A.; Schilling, L.; Thome, C.	Hypothermia reduces early hypoperfusion and metabolic alterations during the acute phase of massive subarachnoid hemorrhage: a laser-Doppler-flowmetry and microdialysis study in rats	2008	25	539-548	J Neurotrauma
Segovia, G.; Del Arco, A.; Mora, F.	Endogenous glutamate increases extracellular concentrations of dopamine, GABA, and taurine through NMDA and AMPA/kainate receptors in striatum of the freely moving rat: a microdialysis study	1997	69	1476-1483	J Neurochem
Segovia, G.; Porras, A.; Mora, F.	Effects of 4-aminopyridine on extracellular concentrations of glutamate in striatum of the freely moving rat	1997	22	1491-1497	Neurochem Res
Semba, J.; Wakuta, M. S.	Regional differences in the effects of glutamate uptake inhibitor L-trans-pyrrolidine-2,4-dicarboxylic acid on extracellular amino acids and dopamine in rat brain: an in vivo microdialysis study	1998	31	399-404	Gen Pharmacol
Shah, A. J.; de Biasi, V.; Taylor, S. G.; Roberts, C.; Hemmati, P.; Munton, R.; West, A.; Routledge, C.; Camilleri, P.	Development of a protocol for the automated analysis of amino acids in brain tissue samples and microdialysates	1999	735	133-140	J Chromatogr B Biomed Sci Appl
Shen, E. Y.; Lai, Y. J.	In vivo microdialysis study of excitatory and inhibitory amino acid levels in the hippocampus following penicillin-induced seizures in mature rats	2002	43	313-318	Acta Paediatr Taiwan
Shi, L.; Yang, A. C.; Li, J. J.; Meng, D. W.; Jiang, B.; Zhang, J. G.	Favorable modulation in neurotransmitters: effects of chronic anterior thalamic nuclei stimulation observed in epileptic monkeys	2015	265	94-101	Exp Neurol
Shibanoki, S.; Kogure, M.; Sugahara, M.; Ishikawa, K.	Effect of systemic administration of N-methyl-D-aspartic acid on extracellular taurine level measured by microdialysis in the hippocampal CA1 field and striatum of rats	1993	61	1698-1704	J Neurochem
Shima, K.; Shirotani, T.; Chigasaki, H.	Delayed neuronal damage following focal ischemic injury in stroke-prone spontaneously hypertensive rats	1996	67	24-27	Acta Neurochir Suppl

Taurine					
**Authors**	**Title**	**Year**	**Vol**	**Pages**	**Journal**
Shimizu, H.; Graham, S. H.; Chang, L. H.; Mintorovitch, J.; James, T. L.; Faden, A. I.; Weinstein, P. R.	Relationship between extracellular neurotransmitter amino acids and energy metabolism during cerebral ischemia in rats monitored by microdialysis and in vivo magnetic resonance spectroscopy	1993	605	33-42	Brain Res
Shirotani, T.; Shima, K.; Chigasaki, H.	In vivo studies of extracellular metabolites in the striatum after distal middle cerebral artery occlusion in stroke-prone spontaneously hypertensive rats	1995	26	878-884	Stroke
Shou, M.; Smith, A. D.; Shackman, J. G.; Peris, J.; Kennedy, R. T.	In vivo monitoring of amino acids by microdialysis sampling with on-line derivatization by naphthalene-2,3-dicarboxyaldehyde and rapid micellar electrokinetic capillary chromatography	2004	138	189-197	J Neurosci Methods
Sierra-Paredes, G.; Alonso, S.; Sierra-Marcuno, G.	Extracellular taurine levels are increased in mice hippocampus during latrunculin a-induced seizures	2009	50	174-175	Epilepsia
Sierra-Paredes, G.; Galan-Valiente, J.; Vazquez-Illanes, M. D.; Aguilar-Veiga, E.; Soto-Otero, R.; Mendez-Alvarez, E.; Sierra-Marcuno, G.	Extracellular amino acids in the rat hippocampus during picrotoxin threshold seizures in chronic microdialysis experiments	1998	248	53-56	Neurosci Lett
Sierra-Paredes, G.; Loureiro, A. I.; Wright, L. C.; Sierra-Marcuno, G.; Soares-da-Silva, P.	Effects of eslicarbazepine acetate on acute and chronic latrunculin A-induced seizures and extracellular amino acid levels in the mouse hippocampus	2014	15	134	BMC Neurosci
Silverstein, F. S.; Naik, B.; Simpson, J.	Hypoxia-ischemia stimulates hippocampal glutamate efflux in perinatal rat brain: an in vivo microdialysis study	1991	30	587-590	Pediatr Res
Silverstein, F. S.; Simpson, J.; Gordon, K. E.	Hypoglycemia alters striatal amino acid efflux in perinatal rats: an in vivo microdialysis study	1990	28	516-521	Ann Neurol
Simpson Jr, R. K.; Robertson, C. S.; Goodman, J. C.	Segmental release of amino acid neurotransmitters from transcranial stimulation	1991	16	89-94	Neurochem Res
Smith, A.; Watson, C. J.; Frantz, K. J.; Eppler, B.; Kennedy, R. T.; Peris, J.	Differential increase in taurine levels by low-dose ethanol in the dorsal and ventral striatum revealed by microdialysis with on-line capillary electrophoresis	2004	28	1028-1038	Alcohol Clin Exp Res
Smolders, I.; Sarre, S.; Michotte, Y.; Ebinger, G.	The analysis of excitatory, inhibitory and other amino acids in rat brain microdialysates using microbore liquid chromatography	1995	57	47-53	J Neurosci Methods
Solas, A. B.; Kutzsche, S.; Vinje, M.;	Cerebral hypoxemia-ischemia and reoxygenation with	2001	2	340-345	Pediatr Crit Care Med

Taurine					
**Authors**	**Title**	**Year**	**Vol**	**Pages**	**Journal**
Saugstad, O. D.	21% or 100% oxygen in newborn piglets: effects on extracellular levels of excitatory amino acids and microcirculation				
Solis, J. M.; Herranz, A. S.; Herreras, O.; Lerma, J.; Martin Del Rio, R.	Low chloride-dependent release of taurine by a furosemide-sensitive process in the in vivo rat hippocampus	1988	24	885-891	Neuroscience
Solis, J. M.; Herranz, A. S.; Herreras, O.; Lerma, J.; Martin del Rio, R.	Does taurine act as an osmoregulatory substance in the rat brain?	1988	91	53-58	Neurosci Lett
Solis, J. M.; Herranz, A. S.; Herreras, O.; Menendez, N.; del Rio, R. M.	Weak organic acids induce taurine release through an osmotic-sensitive process in in vivo rat hippocampus	1990	26	159-167	J Neurosci Res
Solis, J. M.; Herranz, A. S.; Herreras, O.; Munoz, M. D.; Martin del Rio, R.; Lerma, J.	Variation of potassium ion concentrations in the rat hippocampus specifically affects extracellular taurine levels	1986	66	263-268	Neurosci Lett
Song, P.; Mabrouk, O. S.; Hershey, N. D.; Kennedy, R. T.	In vivo neurochemical monitoring using benzoyl chloride derivatization and liquid chromatography-mass spectrometry	2012	84	412-419	Anal Chem
Stein-Behrens, B. A.; Elliott, E. M.; Miller, C. A.; Schilling, J. W.; Newcombe, R.; Sapolsky, R. M.	Glucocorticoids exacerbate kainic acid-induced extracellular accumulation of excitatory amino acids in the rat hippocampus	1992	58	1730-1735	J Neurochem
Suarez, L. M.; Munoz, M. D.; Gonzalez, J. C.; Bustamante, J.; Del Rio, R. M.; Solis, J. M.	The taurine transporter substrate guanidinoethyl sulfonate mimics the action of taurine on long-term synaptic potentiation	2016	48	2647-2656	Amino Acids
Sved, A. F.; Curtis, J. T.	Amino acid neurotransmitters in nucleus tractus solitarius: an in vivo microdialysis study	1993	61	2089-2098	J Neurochem
Szeg, E. M.; Kekesi, K. A.; Szabo, Z.; Janaky, T.; Juhasz, G. D.	Estrogen regulates cytoskeletal flexibility, cellular metabolism and synaptic proteins: A proteomic study	2010	35	807-819	Psychoneuroendocrinology
Szyndler, J.; Maciejak, P.; Turzynska, D.; Sobolewska, A.; Lehner, M.; Taracha, E.; Walkowiak, J.; Skorzewska, A.; Wislowska-Stanek, A.; Hamed, A.; Bidzinski, A.; Plaznik, A.	Changes in the concentration of amino acids in the hippocampus of pentylenetetrazole-kindled rats	2008	439	245-249	Neurosci Lett
Takeda, A.; Sakurada, N.; Kanno, S.; Ando, M.; Oku, N.	Vulnerability to seizures induced by potassium dyshomeostasis in the hippocampus in aged rats	2008	54	37-42	J Health Sci

Taurine					
**Authors**	**Title**	**Year**	**Vol**	**Pages**	**Journal**
Takeda, A.; Tamano, H.; Kan, F.; Hanajima, T.; Yamada, K.; Oku, N.	Enhancement of social isolation-induced aggressive behavior of young mice by zinc deficiency	2008	82	909-914	Life Sci
Tan, W. K.; Williams, C. E.; During, M. J.; Mallard, C. E.; Gunning, M. I.; Gunn, A. J.; Gluckman, P. D.	Accumulation of cytotoxins during the development of seizures and edema after hypoxic-ischemic injury in late gestation fetal sheep	1996	39	791-797	Pediatr Res
Tanaka, S.; Kiuchi, Y.; Tsuchida, A.; Yamamoto, T.; Oguchi, K.; Yoshida, T.; Kuroiwa, Y.	Possible involvement of an increase in brain extracellular concentration of excitatory amino acids in convulsions in phenobarbital-withdrawn rats	1996	17	91-102	Res Commun Alcohol S
Taylor, D. L.; Davies, S. E.; Obrenovitch, T. P.; Doheny, M. H.; Patsalos, P. N.; Clark, J. B.; Symon, L.	Investigation into the role of N-acetylaspartate in cerebral osmoregulation	1995	65	275-281	J Neurochem
Togashi, H.; Nakamura, K.; Matsumoto, M.; Ueno, K.; Ohashi, S.; Saito, H.; Yoshioka, M.	Aniracetam enhances glutamatergic transmission in the prefrontal cortex of stroke-prone spontaneously hypertensive rats	2002	320	109-112	Neurosci Lett
Tong, X.; Patsalos, P. N.	A microdialysis study of the novel antiepileptic drug levetiracetam: extracellular pharmacokinetics and effect on taurine in rat brain	2001	133	867-874	Br J Pharmacol
Tossman, U.; Delin, A.; Eriksson, L. S.; Ungerstedt, U.	Brain cortical amino acids measured by intracerebral dialysis in portacaval shunted rats	1987	12	265-269	Neurochem Res
Tossman, U.; Eriksson, S.; Delin, A.; Hagenfeldt, L.; Law, D.; Ungerstedt, U.	Brain amino acids measured by intracerebral dialysis in portacaval shunted rats	1983	41	1046-1051	J Neurochem
Tossman, U.; Jonsson, G.; Ungerstedt, U.	Regional distribution and extracellular levels of amino acids in rat central nervous system	1986	127	533-545	Acta Physiol Scand
Tossman, U.; Segovia, J.; Ungerstedt, U.	Extracellular levels of amino acids in striatum and globus pallidus of 6-hydroxydopamine-lesioned rats measured with microdialysis	1986	127	547-551	Acta Physiol Scand
Tossman, U.; Wieloch, T.; Ungerstedt, U.	gamma-Aminobutyric acid and taurine release in the striatum of the rat during hypoglycemic coma, studied by microdialysis	1985	62	231-235	Neurosci Lett
Toth, E.	Effect of nicotine on the level of extracellular amino acids in the hippocampus of rat	1996	21	903-907	Neurochem Res
Toth, E.; Harsing Jr, L. G.; Sershen, H.; Ramacci, M. T.; Lajtha, A.	Effect of acetyl-L-carnitine on extracellular amino acid levels in vivo in rat brain regions	1993	18	573-578	Neurochem Res

Taurine					
**Authors**	**Title**	**Year**	**Vol**	**Pages**	**Journal**
Toth, E.; Vizi, E. S.; Lajtha, A.	Effect of nicotine on levels of extracellular amino acids in regions of the rat brain in vivo	1993	32	827-832	Neuropharmacology
Trabace, L.; Cassano, T.; Cagiano, R.; Tattoli, M.; Pietra, C.; Steardo, L.; Kendrick, K. M.; Cuomo, V.	Effects of ENA713 and CHF2819, two anti-Alzheimer's disease drugs, on rat amino acid levels	2001	910	182-186	Brain Res
Trabace, L.; Kendrick, K. M.	Nitric oxide can differentially modulate striatal neurotransmitter concentrations via soluble guanylate cyclase and peroxynitrite formation	2000	75	1664-1674	J Neurochem
Tsukada, Y.; Kanamatsu, T.; Takahara, H.	Neurotransmitter release from the medial hyperstriatum ventrale of the chick forebrain accompanying filial imprinting behavior, measured by in vivo microdialysis	1999	24	315-320	Neurochem Res
Uchiyama-Tsuyuki, Y.; Araki, H.; Yae, T.; Otomo, S.	Changes in the extracellular concentrations of amino acids in the rat striatum during transient focal cerebral ischemia	1994	62	1074-1078	J Neurochem
Ueda, Y.; Mitsuyama, Y.	Nitroprusside inhibits kindling development and potentiates excitatory amino acid exocytosis in the kindling rat	1997	23	49-60	J Brain Sci
Van Hemelrijck, A.; Hachimi-Idrissi, S.; Sarre, S.; Ebinger, G.; Michotte, Y.	Post-ischaemic mild hypothermia inhibits apoptosis in the penumbral region by reducing neuronal nitric oxide synthase activity and thereby preventing endothelin-1-induced hydroxyl radical formation	2005	22	1327-1337	Eur J Neurosci
Van Hemelrijck, A.; Sarre, S.; Smolders, I.; Michotte, Y.	Determination of amino acids associated with cerebral ischaemia in rat brain microdialysates using narrowbore liquid chromatography and fluorescence detection	2005	144	63-71	J Neurosci Methods
Varga, V.; Kekesi, A.; Juhasz, G.; Kocsis, B.	Reduction of the extracellular level of glutamate in the median raphe nucleus associated with hippocampal theta activity in the anaesthetized rat	1998	84	49-57	Neuroscience
Venton, B. J.; Robinson, T. E.; Kennedy, R. T.	Transient changes in nucleus accumbens amino acid concentrations correlate with individual responsivity to the predator fox odor 2,5-dihydro-2,4,5-trimethylthiazoline	2006	96	236-246	J Neurochem
Venton, B. J.; Robinson, T. E.; Kennedy, R. T.; Maren, S.	Dynamic amino acid increases in the basolateral amygdala during acquisition and expression of	2006	23	3391-3398	Eur J Neurosci

**Taurine**					
**Authors**	**Title**	**Year**	**Vol**	**Pages**	**Journal**
	conditioned fear				
Vezzani, A.; Ungerstedt, U.; French, E. D.; Schwarcz, R.	In vivo brain dialysis of amino acids and simultaneous EEG measurements following intrahippocampal quinolinic acid injection: evidence for a dissociation between neurochemical changes and seizures	1985	45	335-344	J Neurochem
Wade, J. V.; Samson, F. E.; Nelson, S. R.; Pazdernik, T. L.	Changes in extracellular amino acids during soman- and kainic acid-induced seizures	1987	49	645-650	J Neurochem
Wang, J.; Peng, Y. J.; Zhu, D. N.	Amino acids modulate the hypotensive effect of angiotensin-(1-7) at the caudal ventrolateral medulla in rats	2005	129	1-7	Regul Pept
Wang, J.; Shen, L. L.; Cao, Y. X.; Zhu, D. N.	Effects of electroacupuncture on pressor response to angiotensin-(1-7) by amino acid release in the rostral ventrolateral medulla	2003	28	25-34	Acupunct Electrother Res
Wang, L.; Huang, Y.; Wu, J.; Lv, G.; Zhou, L.; Jia, J.	Effect of Buyang Huanwu decoction on amino acid content in cerebrospinal fluid of rats during ischemic/reperfusion injury	2013	86	143-150	J Pharm Biomed Anal
Wang, M.; Hershey, N. D.; Mabrouk, O. S.; Kennedy, R. T.	Collection, storage, and electrophoretic analysis of nanoliter microdialysis samples collected from awake animals in vivo	2011	400	2013-2023	Anal Bioanal Chem
Wang, Y. L.; He, R. R.	Effects of dipfluzine on cortical somatosensory evoked potentials and amino acid contents in ischemic rat brain	1996	17	41-44	Chung Kuo Yao Li Hsueh Pao
Ward, R. J.; Colantuoni, C.; Dahchour, A.; Quertemont, E.; De Witte, P.	Acetaldehyde-induced changes in monoamine and amino acid extracellular microdialysate content of the nucleus accumbens	1997	36	225-232	Neuropharmacology
Ward, R. J.; Colivicchi, M. A.; Allen, R.; Schol, F.; Lallemand, F.; de Witte, P.; Ballini, C.; Corte, L. D.; Dexter, D.	Neuro-inflammation induced in the hippocampus of 'binge drinking 'rats may be mediated by elevated extracellular glutamate content	2009	111	1119-1128	J Neurochem
Watts, J.; Segieth, J.; Pearce, B.; Whitton, P. S.	Regulatory role of nitric oxide over extracellular taurine in the hippocampus of freely moving rats	2004	357	179-182	Neurosci Lett
Westergren, I.; Nystrom, B.; Hamberger, A.; Johansson, B. B.	Amino acids in extracellular fluid in vasogenic brain edema	1994	60	124-127	Acta Neurochir Suppl (Wien)
Westergren, I.; Nystrom, B.; Hamberger, A.; Johansson, B. B.	Intracerebral dialysis and the blood-brain barrier	1995	64	229-234	J Neurochem

Taurine					
**Authors**	**Title**	**Year**	**Vol**	**Pages**	**Journal**
Westergren, I.; Nystrom, B.; Hamberger, A.; Nordborg, C.; Johansson, B. B.	Concentrations of amino acids in extracellular fluid after opening of the blood-brain barrier by intracarotid infusion of protamine sulfate	1994	62	159-165	J Neurochem
Westerink, B. H.; Damsma, G.; de Vries, J. B.	Effect of ouabain applied by intrastriatal microdialysis on the in vivo release of dopamine, acetylcholine, and amino acids in the brain of conscious rats	1989	52	705-712	J Neurochem
Westerink, B. H.; Hofsteede, H. M.; Damsma, G.; de Vries, J. B.	The significance of extracellular calcium for the release of dopamine, acetylcholine and amino acids in conscious rats, evaluated by brain microdialysis	1988	337	373-378	Naunyn Schmiedebergs Arch Pharmacol
Wilson, C. L.; Maidment, N. T.; Shomer, M. H.; Behnke, E. J.; Ackerson, L.; Fried, I.; Engel, J., Jr.	Comparison of seizure related amino acid release in human epileptic hippocampus versus a chronic, kainate rat model of hippocampal epilepsy	1996	26	245-254	Epilepsy Res
Win-Shwe, T. T.; Mitsushima, D.; Nakajima, D.; Ahmed, S.; Yamamoto, S.; Tsukahara, S.; Kakeyama, M.; Goto, S.; Fujimaki, H.	Toluene induces rapid and reversible rise of hippocampal glutamate and taurine neurotransmitter levels in mice	2007	168	75-82	Toxicol Lett
Wong, J. M.; Malec, P. A.; Mabrouk, O. S.; Ro, J.; Dus, M.; Kennedy, R. T.	Benzoyl chloride derivatization with liquid chromatography-mass spectrometry for targeted metabolomics of neurochemicals in biological samples	2016	1446	78-90	J Chromatogr A
Xia, C. M.; Shao, C. H.; Xin, L.; Wang, Y. R.; Ding, C. N.; Wang, J.; Shen, L. L.; Li, L.; Cao, Y. X.; Zhu, D. N.	Effects of melatonin on blood pressure in stress-induced hypertension in rats	2008	35	1258-1264	Clin Exp Pharmacol Physiol
Xu, G. Y.; McAdoo, D. J.; Hughes, M. G.; Robak, G.; De Castro Jr, R.	Considerations in the determination by microdialysis of resting extracellular amino acid concentrations and release upon spinal cord injury	1998	86	1011-1021	Neuroscience
Xu, X. H.; Zheng, X. X.; Li, H.	Effects of puerarin on excitatory amino acid levels in cerebral ischemic striatum of rat. [Chinese]	2008	43	675-679	Chinese Pharmaceutical Journal
Yablonsky-Alter, E.; Agovic, M. S.; Gashi, E.; Lidsky, T. I.; Friedman, E.; Banerjee, S. P.	Cocaine challenge enhances release of neuroprotective amino acid taurine in the striatum of chronic cocaine treated rats: a microdialysis study	2009	79	215-218	Brain Res Bull
Yager, J. Y.; Armstrong, E. A.; Miyashita, H.; Wirrell, E. C.	Prolonged neonatal seizures exacerbate hypoxic-ischemic brain damage: correlation with cerebral energy metabolism and excitatory amino acid release	2002	24	367-381	Dev Neurosci

Taurine					
**Authors**	**Title**	**Year**	**Vol**	**Pages**	**Journal**
Yamada, K.; Moriguchi, A.; Mikami, H.; Okuda, N.; Higaki, J.; Ogihara, T.	The effect of central amino acid neurotransmitters on the antihypertensive response to angiotensin blockade in spontaneous hypertension	1995	13	1624-1630	J Hypertension
Yamamoto, B. K.; Davy, S.	Dopaminergic modulation of glutamate release in striatum as measured by microdialysis	1992	58	1736-1742	J Neurochem
Yang, C. S.; Tsai, P. J.; Chen, W. Y.; Tsai, W. J.; Kuo, J. S.	On-line derivatization for continuous and automatic monitoring of brain extracellular glutamate levels in anesthetized rats: a microdialysis study	1999	734	1-6	J Chromatogr B Biomed Sci Appl
Yao, H.; Ooboshi, H.; Ibayashi, S.; Uchimura, H.; Fujishima, M.	Cerebral blood flow and ischemia-induced neurotransmitter release in the striatum of aged spontaneously hypertensive rats	1993	24	577-580	Stroke
Young, R. S.; During, M. J.; Aquila, W. J.; Tendler, D.; Ley, E.	Hypoxia increases extracellular concentrations of excitatory and inhibitory neurotransmitters in subsequently induced seizure: in vivo microdialysis study in the rabbit	1992	117	204-209	Exp Neurol
Yu, H. L.; An, Y.; Bing, Y. H.; Jin, Q. H.; Cui, X.; Jin, Y. Z.	[Changes of glutamate and taurine released in the medial vestibular nucleus following acute hypotension]	2006	58	177-182	Sheng Li Xue Bao
Yu, H. L.; An, Y.; Jiang, H. Y.; Jin, Q. H.; Jin, Y. Z.	Changes of amino acid concentrations in the rat medial vestibular nucleus following unilateral labyrinthectomy	2007	59	71-78	Sheng Li Xue Bao
Zhang, J.; Niu, X.	Changes of monoamines, purines and amino acids in rat striatum as measured by intercerebral microdialysis during ischemia/reperfusion	1994	9	225-229	Chin Med Sci J
Zhang, W. Q.; Hudson, P. M.; Sobotka, T. J.; Hong, J. S.; Tilson, H. A.	Extracellular concentrations of amino acid transmitters in ventral hippocampus during and after the development of kindling	1991	540	315-318	Brain Res
Zhang, W. Q.; Rogers, B. C.; Tandon, P.; Hudson, P. M.; Sobotka, T. J.; Hong, J. S.; Tilson, H. A.	Systemic administration of kainic acid increases GABA levels in perfusate from the hippocampus of rats in vivo	1990	11	593-600	Neurotoxicology
Zhao, P.; Cheng, J.	Effects of electroacupuncture on extracellular contents of amino acid neurotransmitters in rat striatum following transient focal cerebral ischemia	1997	22	119-126	Acupunct Electrother Res
Zhao, W. H.; Huang, H. L.; Huang, Y.; Xu, X.; Mo, L. D.	Experimental study on the correlation between the cerebral energy metabolism and cerebral blood flow	2007	7	146-151	Zhongguo Xian Dai Shen Jing Ji Bing Za Zhi

Taurine					
**Authors**	**Title**	**Year**	**Vol**	**Pages**	**Journal**
	changes in rats with subarachnoid hemorrhage-induced delayed cerebral vasospasm. [Chinese]				
Zhao, X. D.; Zhou, X. P.; Liu, H. H.; Li, B. M.; Hu, X. W.; Li, F. Q.; You, B. M.	[Changes of amino acids neurotransmitters in striatum of hemi-parkinsonian rhesus monkey after high frequency stimulation of subthalamic nucleus]	2007	45	1682-1684	Zhonghua Wai Ke Za Zhi
Zhou, Q. M.; Lu, Y. L.; Hu, G.; Zeng, Y. M.; Tu, W. F.	Effect of aquaporin-4 deficiency on intravenous anaesthetic induced hypnotic effects in mice	2013	65	569-576	Sheng Li Xue Bao
Zhu, D. N.; Moriguchi, A.; Mikami, H.; Higaki, J.; Ogihara, T.	Central amino acids mediate cardiovascular response to angiotensin II in the rat	1998	45	189-197	Brain Res Bull
Zielinska, M.; Hilgier, W.; Borkowska, H. D.; Oja, S. S.; Saransaari, P.; Albrecht, J.	Lubeluzole attenuates K(+)-evoked extracellular accumulation of taurine in the striatum of healthy rats and rats with hepatic failure	2001	904	173-176	Brain Res
Zielinska, M.; Hilgier, W.; Borkowska, H. D.; Oja, S. S.; Saransaari, P.; Gorynski, P.; Albrecht, J.	Ammonia-induced extracellular accumulation of taurine in the rat striatum in vivo: role of ionotropic glutamate receptors	2002	27	37-42	Neurochem Res
Zuiderwijk, M.; Veenstra, E.; Lopes da Silva, F. H.; Ghijsen, W. E.	Effects of uptake carrier blockers SK & F 89976-A and L-trans-PDC on in vivo release of amino acids in rat hippocampus	1996	307	275-282	Eur J Pharmacol

Appendix 10: Study characteristics for microdialysis studies on circadian rhythms of aspartate, GABA, glutamine, glutamate and histamine

Study_ID	Lab Location	N	Species	Strain	Age (weeks)	Weight (g)	Sex	Flow (µL/min)	Probe length (mm)	Probe diameter (mm)	Membrane	Wash-out (hours)	Anaesthesia	Post-surgical recovery (days)	Analysis	Lights ON
**Asp**																
Honma_1996b	Sapporo	15	Rats	Wistar	12	na	m	1	1	0	Eicom - 50kDa / Cuprophan - 1kDa	2	FB	7	HPLC-ECD	06:00-18:00
Honma_1996d	Sapporo	12	Rats	Wistar	12	na	m	2	1	0	Cuprophan - 1kDa	2	urethane	7	HPLC-ECD	06:00-18:00
Glass_1993b	Kent	7	Hamsters	Home bred	8-15	~125	m	1	0,5	0	Spectra-por, Fisher - 6kDa	na	FB	na	HPLC-FD / HPLC-ECD	08:00-22:00
**GABA**																
Castaneda_2004b	Madrid	7	Rats	Wistar	8-16	250-350	m	1,3	4	na	Hospal - 5kDa	na	FB	10	HPLC-FD	20:00-8:00
Castaneda_2004e	Madrid	8	Rats	Wistar	8-16	250-350	m	1,3	2,5	na	Hospal - 5kDa	na	FB	10	HPLC-FD	20:00-8:00
MarquezdePrado_2000b	Madrid	4	Rats	Wistar	8-12	250-350	m	1,3	4	na	Hospal - 5kDa	na	FB	10-15	HPLC-FD	08:00-20:00
Meng_2015	Fuzhou	5	Rats	Sprague-Dawley	adult	200-220	m	2	4	na	CMA12 - 20kDa	1	FB	7	HPLC-FD	07:00-19:00
**Gln**																
Castaneda_2004c	Madrid	na	Rats	Wistar	8-16	250-350	m	1,3	4	na	Hospal - 5kDa	na	FB	10	HPLC-FD	20:00-8:00
Castaneda_2004f	Madrid	na	Rats	Wistar	8-16	250-350	m	1,3	2,5	na	Hospal - 5kDa	na	FB	10	HPLC-FD	20:00-8:00
MarquezdePrado_2000c	Madrid	4	Rats	Wistar	8-12	250-350	m	1,3	4	na	Hospal - 5kDa	na	FB	10-15	HPLC-FD	08:00-20:00
Glass_1993c	Kent	7	Hamsters	Home bred	8-15	~125	m	1	0,5	0	Spectra-por, Fisher - 6kDa	na	FB	na	HPLC-FD / HPLC-ECD	08:00-22:00
**Glu**																
Castaneda_2004a	Madrid	7	Rats	Wistar	8-16	250-350	m	1,3	4	na	Hospal - 5kDa	na	FB	10	HPLC-FD	20:00-8:00
Castaneda_2004d	Madrid	8	Rats	Wistar	8-16	250-350	m	1,3	2,5	na	Hospal - 5kDa	na	FB	10	HPLC-FD	20:00-8:00
Honma_1996a	Sapporo	15	Rats	Wistar	12	na	m	1	1	0	Eicom - 50kDa / Cuprophan - 1kDa	2	FB	7	HPLC-ECD	06:00-18:00

Study_ID	Lab Location	N	Species	Strain	Age (weeks)	Weight (g)	Sex	Flow (µL/min)	Probe length (mm)	Probe diameter (mm)	Membrane	Wash-out (hours)	Anaesthesia	Post-surgical recovery (days)	Analysis	Lights ON
Honma_1996c	Sapporo	12	Rats	Wistar	12	na	m	2	1	0	Cuprophan - 1kDa	2	urethane	7	HPLC-ECD	06:00-18:00
MarquezdePrado_ 2000a	Madrid	4	Rats	Wistar	8-12	250-350	m	1,3	4	na	Hospal - 5kDa	na	FB	10-15	HPLC-FD	08:00-20:00
Glass_1993a	Kent	7	Hamsters	Home bred	8-15	~125	m	1	0,5	0	Spectra-por, Fisher - 6kDa	na	FB	na	HPLC-FD / HPLC-ECD	08:00-22:00
Meng_2015	Fuzhou	5	Rats	Sprague-Dawley	adult	200-220	m	2	4	na	CMA12 - 20kDa	1	FB	7	HPLC-FD	07:00-19:00
**Hist**																
Fell_2015	Indianapolis	5-12	Rat	Sprague-Dawley	na	300-350g	na	na	na	na	na	na	na	na	HPLC-FD	24:00-12:00
Chu_2004	Osaka	5	Rats	Sprague-Dawley	8-11	260-320	m	2	3	1	CMA	20	FB	10	HPLC-FD	8:00-20:00
Hong_2005	Osaka	5-6	Rats	Sprague-Dawley	8-10	280-320	m	2	3	1	20kDa	20	FB	10	HPLC-FD	8:00-20:00
Zant_2012	Helsinki	1	Rat	Han-Wistar	12-16	300-400	m	1	2	0	CMA11	na	FB	7	HPLC-FD	? (all in ZT)
Mochizuki_1992	Osaka	8	Rats	Wistar	na	190-250	m	1	2	na	CMA12	1	FB	2	HPLC-FD	08:00-20:00
Rozov_2014	Helsinki	5	Mice	C57BL6/ J	na	na	m	1	1	na	CMA7	24	FB	2-3	HPLC-FD	20:30-8:30
MarquezdePrado_ 2000d	Madrid	4	Rats	Wistar	8-12	250-350	m	1,3	4	na	Hospal - 5kDa	na	FB	10-15	HPLC-FD	08:00-20:00

Asp: aspartate; ECD: electrochemical detection; FB: Freely Behaving; FD: fluorescent detection; GABA: gamma-aminobutyric acid; Gln: glutamine; Glu: Glutamate; Hist: histamine; HPLC: high pressure liquid chromatography; kDa: kiloDalton; ZT: zeitgeber.
